# Diastereodivergent Access to *cis*,*cis*- and *cis*,*trans*-Angular Triquinanes: Total Synthesis of (±)-Arnicenone

**DOI:** 10.1021/jacs.6c09019

**Published:** 2026-09-22

**Authors:** Chia-Hao Liu, Hung-Tse Huang, Han-Jung Li, Shubham A. Dighe, Deng-Lian Hou, Li-Yun Wu, Yu-Wen Huang

**Affiliations:** † Department of Chemistry, 34881 National Tsing Hua University, 101, section 2, Kuang-Fu Road, Hsinchu 300044, Taiwan, R.O.C.; ‡ Department of Chemistry, 34900 Chung Yuan Christian University, No. 200, Zhongbei Rd., Zhongli Dist., Taoyuan City 320314, Taiwan, R.O.C.

## Abstract

Angular triquinanes constitute an important class of polycyclic natural products characterized by compact, angularly fused ring systems and multiple contiguous stereocenters. Herein, we report a diastereodivergent strategy for stereocontrolled access to both *cis*,*trans*- and *cis*,*cis*-angular triquinanes from common allene precursors. The *cis*,*trans*-fused triquinanes were constructed through a Lewis acid-promoted intramolecular [3+2] Danheiser annulation, whereas the complementary *cis*,*cis* frameworks were accessed via photochemical [2+2] cycloaddition followed by oxidative ring expansion. Notably, substitution at the R^1^ position of the allene precursor reversed the diastereoselectivity of the [3+2] annulation, providing direct access to a *cis*,*cis*-angular triquinane. DFT calculations indicate that the initial spirocyclization governs both the reactivity and stereochemical outcome of the [3+2] pathway, with substrate-dependent steric interactions accounting for the observed diastereodivergence. The synthetic utility of this strategy was demonstrated through the total synthesis of (±)-arnicenone via the [2+2] cycloaddition/oxidative ring-expansion pathway, while an alternative synthesis from the *cis*,*cis*-[3+2] Danheiser annulation product further highlights the complementary nature of the two approaches. Together, these results establish a unified platform for stereodivergent access to angular triquinanes and their application to complex natural product synthesis.

## Graphical abstract

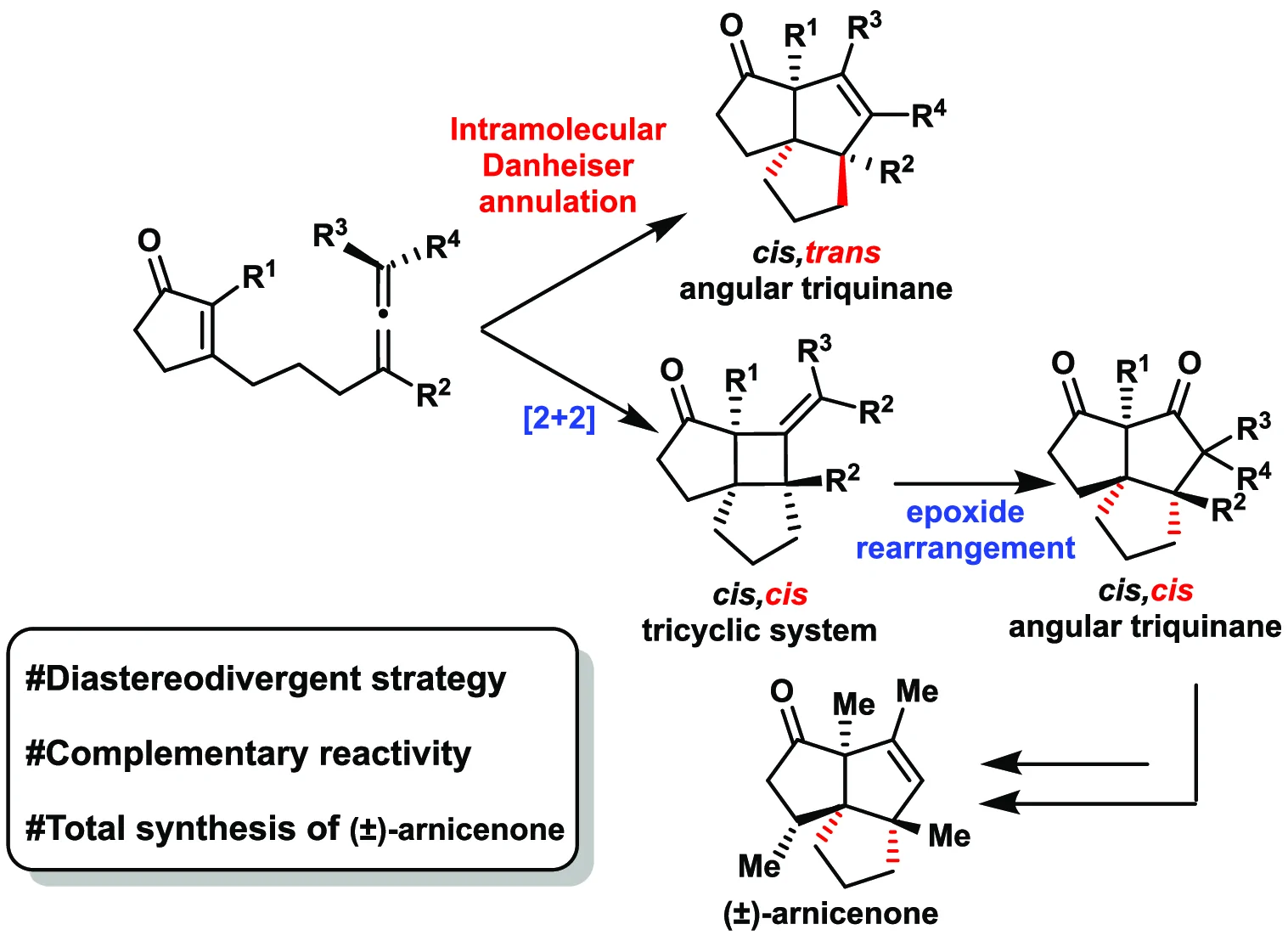

Angular triquinanes^1–3^ (**1** and **2**) constitute a prominent structural motif in natural products, particularly within terpenoid and sesquiterpenoid families (Figure 1). Their compact, angularly fused tricyclic framework—comprising three contiguous five-membered rings—features multiple stereogenic centers and significant conformational constraint. This dense stereochemical architecture renders angular triquinanes both synthetically challenging and biologically intriguing. Among these, arnicenone^4–7^ (**3**) exemplifies the structural complexity associated with this class of molecules. Other representative members include isocomene^8,9^ (**4**), pentalenene^10,11^ (**5**), retigeranic acid^12,13^ (**6**), and waihoensene^14,15^ (**7**), which collectively illustrate the structural diversity of angular triquinane natural products. These molecules differ in their oxidation states, substitution patterns, and ring fusion architectures, yet all possess highly congested polycyclic frameworks featuring multiple contiguous stereogenic centers. Their remarkable structural complexity has inspired the development of numerous synthetic strategies and continues to serve as an important driving force in angular triquinane synthesis. Considerable efforts have been devoted to the construction of *cis*,*cis*-triquinane skeletons (**1**). Representative strategies include assembly of the tricyclic core from preformed diquinane intermediates,^16^ the Pauson–Khand reaction (Scheme 1a),^17–23^ ring expansion of cyclobutane derivatives (**10**) (Scheme 1b),^24^ and various pericyclic transformations such as Nazarov reaction (Scheme 1c).^25,26^

**Figure 1:**
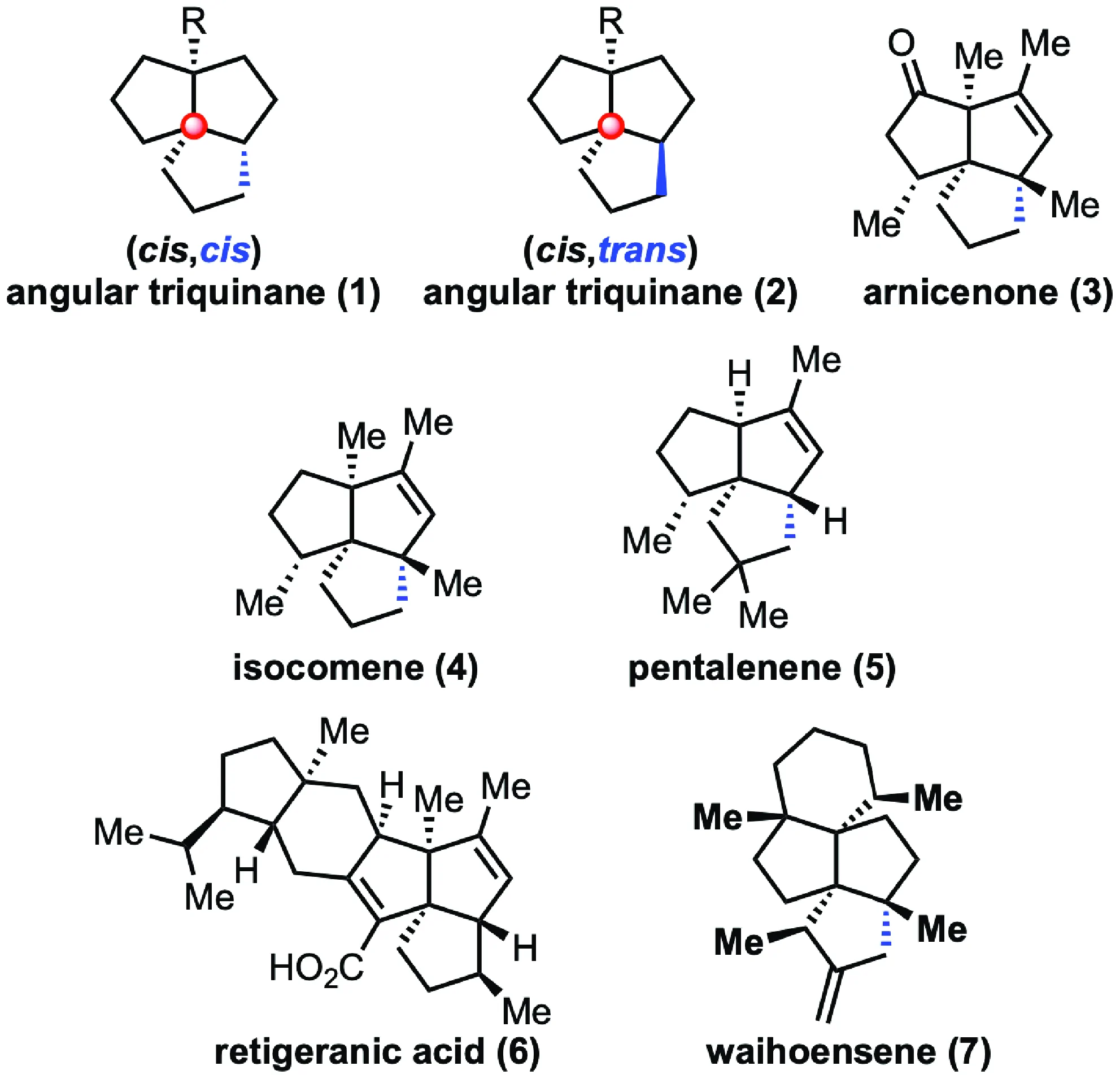
Representative stereochemistry of angular triquinanes and their related natural products.

**Scheme 1:**
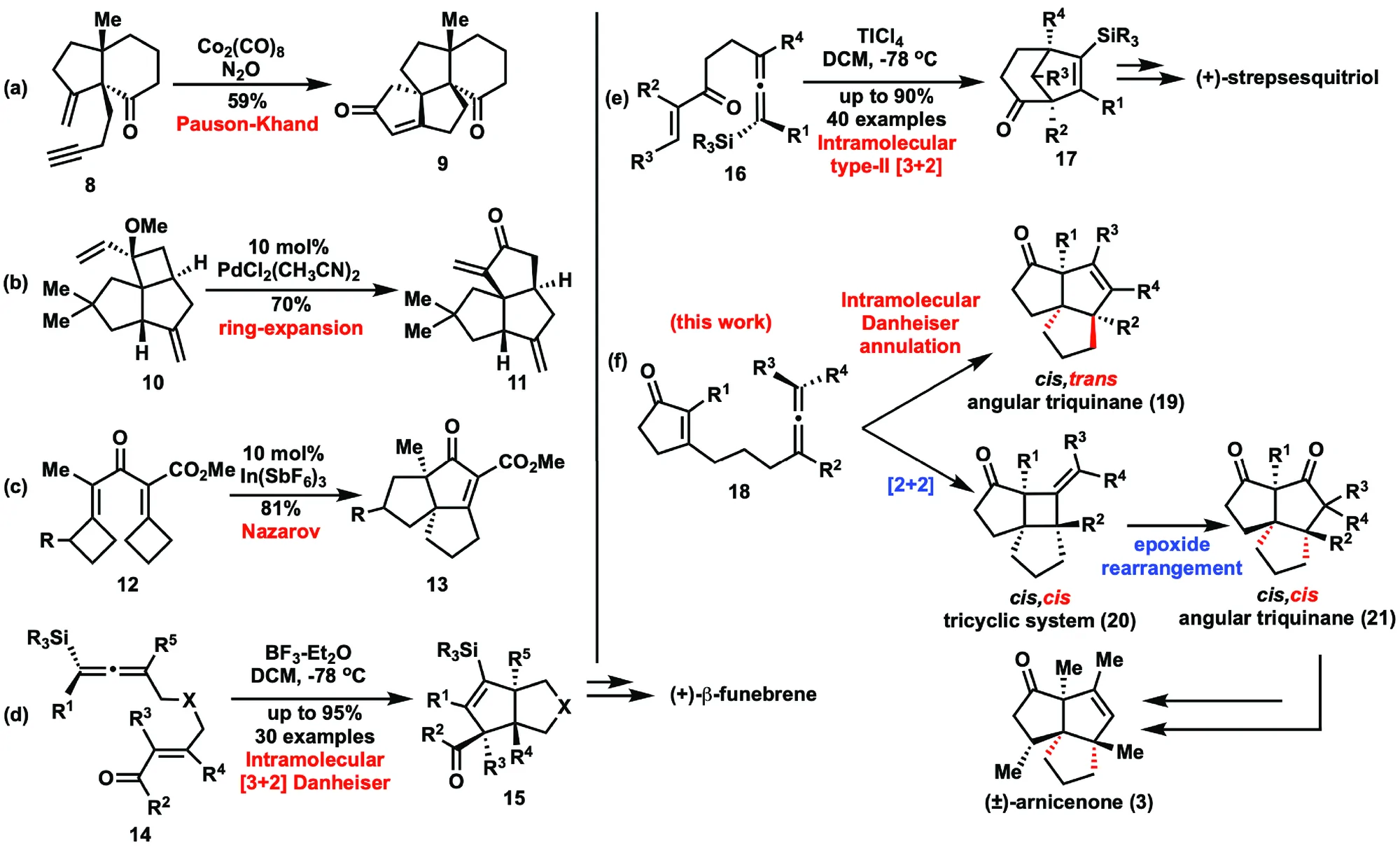
Methods for Synthesis of Angular Triquinanes and Strained Natural Products

More broadly, the construction of highly congested and stereochemically constrained molecular architectures remains a longstanding challenge in synthetic chemistry, as exemplified by *trans*-fused 5,5 ring systems,^27^ strained bridged polycycles, and atropisomeric systems.^28–32^ Within this context, angular triquinanes present a distinct synthetic challenge because their compact polycyclic frameworks require precise control over multiple stereogenic centers and ring-junction geometries. While naturally occurring angular triquinanes predominantly exhibit *cis*,*cis* ring-junction stereochemistry, the corresponding *cis*,*trans* frameworks remain largely unexplored. Despite recent advances in the construction of *cis*,*cis*-angular triquinane skeletons, methods enabling diastereodivergent^33–35^ access to both stereochemical manifolds from closely related precursors remain limited. Such strategies are highly desirable because they would provide access not only to naturally occurring frameworks but also to complementary stereochemical space beyond that represented in nature.

In 1981, Danheiser and co-workers reported the first intermolecular [3+2] annulation of allenylsilanes with alkenes, which has since emerged as a useful strategy for the construction of *cis*-fused 5/5 ring systems.^36–40^ More than four decades later, Li and co-workers developed the first intramolecular variant of this transformation, providing an efficient approach to *trans*-fused 5/5 ring systems and demonstrating its synthetic utility in the synthesis of β-funebrene (Scheme 1d).^41^ Li and co-workers subsequently extended this strategy to a Type II intramolecular [3+2] annulation, further broadening the synthetic scope of this transformation and enabling its application to the synthesis of strepsesquitriol (Scheme 1e).^42^ The synthetic utility of the Danheiser annulation was further demonstrated by Zhai and co-workers in their total synthesis of (−)-conidiogenone, in which the annulation was employed to construct a 5,5,5-*cis*,*cis*-angular tricyclic framework.^43^ Inspired by these advances, we envisioned that the intramolecular [3+2] Danheiser annulation could provide stereocontrolled access to angular triquinanes with distinct ring-junction stereochemistries. Specifically, *cis*,*trans*-angular triquinanes (**19**) were targeted via a [3+2] Danheiser annulation strategy from allenic precursor (**18**), leveraging its efficiency in constructing five-membered carbocycles with well-defined stereochemical outcomes (Scheme 1f). In contrast, *cis*,*cis*-angular triquinanes (**21**) were designed to arise from a [2+2] cycloaddition followed by oxidative ring expansion,^44–47^ offering a mechanistically orthogonal pathway to the same carbon framework. Herein, we report a diastereodivergent strategy for the synthesis of angular triquinanes that enables stereocontrolled access to both *cis*,*trans*- and *cis*,*cis*-fused systems. The synthetic utility of this approach is further demonstrated through its application to the total synthesis of (±)-arnicenone (**3**).

A series of different allenes were prepared as outlined in Scheme 2. The diketone intermediates **24**(**a-d**) were obtained efficiently via Stork-Danheiser transposition^48^ with Grignard (**23**) and sequential acidic workup in 71–82% yield. These diketones **24**(**a-d**) then underwent regioselective acetylide addition and acylation to deliver **25**(**a-k**) in 29–79% yield. The stage was set for subsequent S_N_2′ allenation proceeding cleanly to afford the desired allenes **26**(**a-n**) in 57–91% yield, establishing unified intermediates poised for divergent cyclization.

**Scheme 2:**
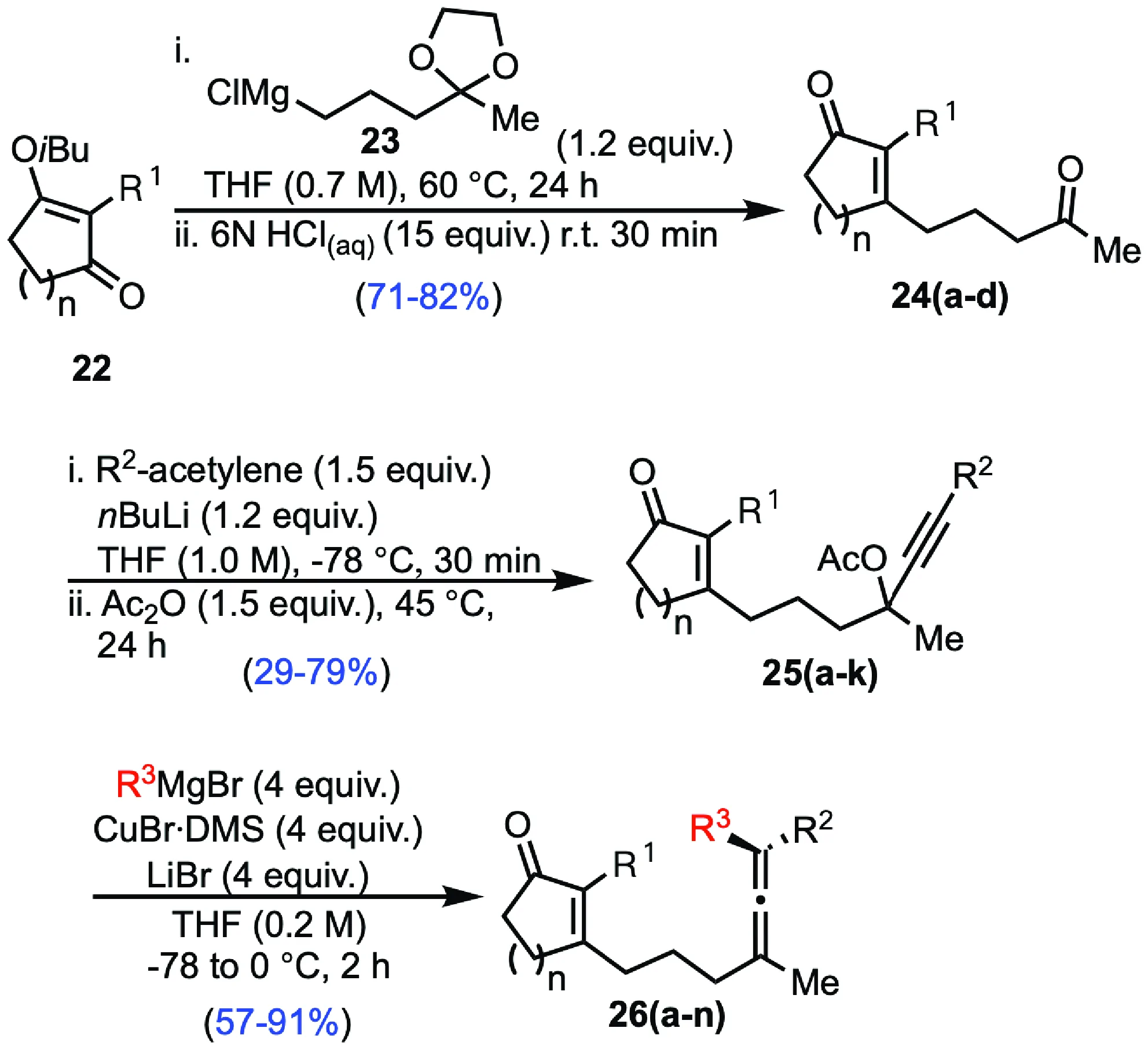
Synthesis of Allenes **26**(**a-n**)

## [3+2] Danheiser Annulation

With the allenes in hand, we first examined the intramolecular [3+2] Danheiser annulation (Table 1). Optimization^49^ of the Lewis acid, solvent, and reaction temperature identified conditions that efficiently furnished the angular triquinane products with generally high diastereoselectivity. Substrates bearing R^1^ = H readily underwent the annulation to afford the corresponding angular triquinanes **28**(**a–l**), generally favoring the *cis*,*trans*-diastereomer. The *cis*,*cis*/*cis*,*trans* ratios ranged from 1:2 to exclusive formation of the *cis*,*trans*-isomer. Notably, the cyclohexenone-derived substrates **28**(**j–l**) furnished the *cis*,*trans*-isomer exclusively. Variation at R^2^ was well tolerated, with substrates bearing R^2^ = H (**28**(**g–i**)) providing the desired products in moderate to good yields (45–82%). In contrast, increasing the steric demand at R^3^ resulted in diminished reactivity. Substrates bearing R^3^ = Et (**28f** and **28l**) preferentially afforded the *cis*,*trans*-isomer, albeit in lower yield (33% for **28l**), whereas further increasing the size of R^3^ to *n*-Bu or *i*-Pr completely suppressed the desired [3+2] annulation under the optimized conditions. The silyl substituent was also varied to examine its influence on the annulation, with TMS, TES, TBS, TIPS, and DMPS groups being evaluated. In addition, an H,H-substituted allene lacking a silyl group and an H,DMPS-substituted allene were examined, but neither underwent the desired [3+2] annulation under the optimized conditions. The lack of reactivity of the nonsilylated allene is consistent with the proposed Sakurai-type cyclization and the stabilizing β-silyl effect, whereas the failure of the H,DMPS-substituted substrate suggests that an appropriate allene substitution pattern is also required for efficient cyclization. The practicality of the [3+2] Danheiser annulation was further demonstrated by a gram-scale reaction, which furnished **28a** in 58% yield (see Supporting Information for experimental details). Interestingly, introduction of a methyl substituent at R^1^ markedly altered both the reactivity and stereochemical outcome of the annulation. Substrates bearing R^1^ = Me exhibited substantially reduced reactivity. Nevertheless, the cyclopentenone-derived substrate underwent cyclization to afford **28m** in 10% yield with reversed diastereoselectivity, favoring the *cis*,*cis*-isomer (*cis*,*cis*/*cis*,*trans* = 4:1). Desilylation of the allene precursor to give **29** was observed as the predominant competing pathway for these less reactive substrates. The substrate-dependent reversal of diastereoselectivity observed for **28m** prompted further investigation of the stereochemical origin of the [3+2] annulation by DFT calculations (vide infra). Notably, despite its modest yield, *cis*,*cis*-**28m** could be further elaborated to (±)-arnicenone (**3**), demonstrating the synthetic utility of the [3+2] annulation product (vide infra).

**Table 1:**
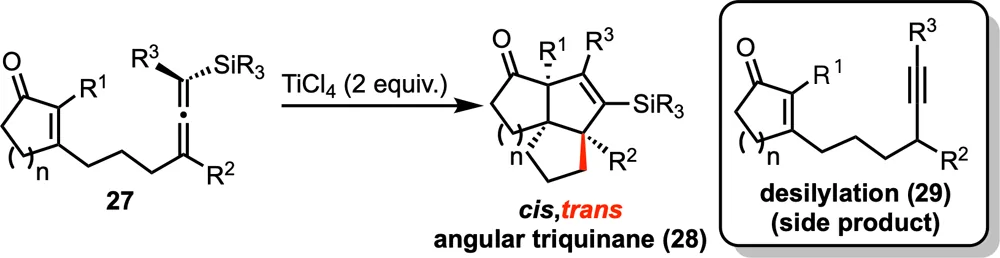

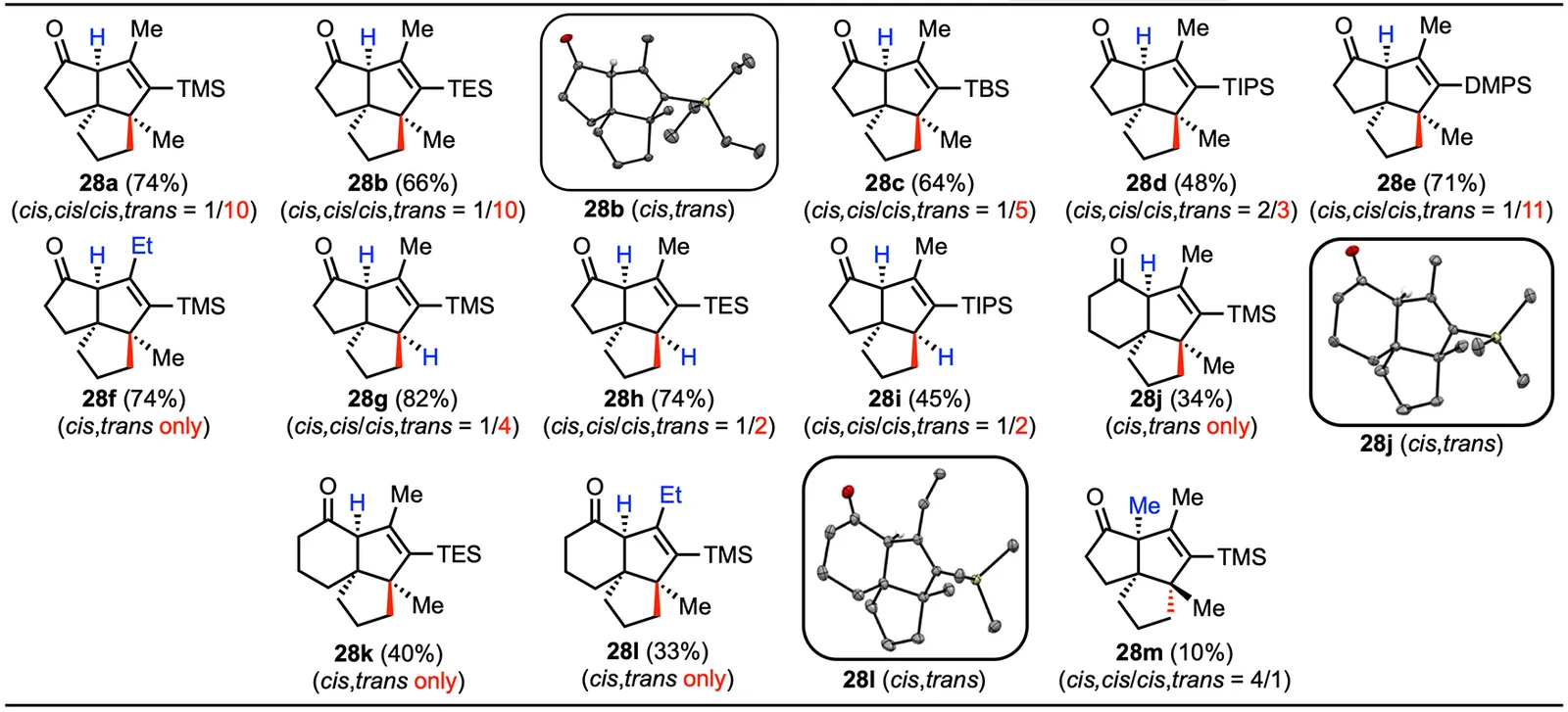
[3+2] Danheiser Annulation^a^ ^,^ ^b^

a Reaction conditions: To SM (1 equiv., 0.2–0.4 mmol) in 0.02 M DCM was added TiCl_4_ (2.0 equiv.). The reaction was stirred at 40 °C until the consumption of SM, monitoring with TLC.

b The reaction was carried out at up to 3.8 mmol (SM) scale.

## [2+2] Cycloaddition and Ring Expansion

To access the complementary *cis*,*cis*-fused framework, we next evaluated a [2+2] cycloaddition strategy (Table 2). Cycloaddition of the corresponding allene precursors (**27**) afforded cyclobutane intermediates **30**, which likely exploit the inherent rigidity of the four-membered ring to control the stereochemical outcome, delivering *cis*,*cis*-5,4,5- or *cis*,*cis*-6,4,5-tricyclic skeletons. These intermediates (**30**) subsequently underwent oxidative ring expansion to generate the *cis*,*cis*-angular triquinane core and the corresponding *cis*,*cis*-6,5,5-tricyclic frameworks, respectively. Mechanistically, the distinct stereochemical outcome arises from an orthogonal reaction manifold: the [2+2] pathway establishes an all-*cis* relationship during cyclobutane formation and preserves it through stereoselective oxidative expansion. Thus, selective choice of annulation mode enables predictable control over ring-junction geometry. After optimization, the sequence proceeded in good overall yield with exclusive diastereoselectivity. In general, the [2+2] cycloaddition exhibited broad substrate scope. In contrast to the [3+2] Danheiser annulation (Table 1), which favored substrates bearing R^1^ = H, the [2+2] cycloaddition pathway tolerated both R^1^ = H and Me. Substituents at R^2^ were also well accommodated (H or Me), while variation at R^3^ (Me, Et, and iPr) was likewise compatible. TMS, TES, and TIPS substituents were all effective under the cycloaddition conditions. Notably, allenes bearing a terminal geminal diproton (**30l**, **30m**, and **30n**) also participated efficiently, affording the desired products in 62–85% yield. The practicality of the [2+2] cycloaddition was further demonstrated by a gram-scale reaction, which furnished **30a** in 55% yield (see Supporting Information for experimental details). To further examine the effect of linker size on the two complementary cyclization pathways, we also investigated an allene precursor designed to generate a six-membered bottom ring. Under the optimized [2+2] cycloaddition conditions, however, the corresponding substrate failed to afford the desired cycloadduct. In contrast, the same substrate underwent the intramolecular [3+2] Danheiser annulation to furnish the *cis*,*trans*-5,5,6-tricyclic product in 30% yield (see Supporting Information for substrate structure and experimental details). These results demonstrate that extension of the linker to generate a six-membered bottom ring is tolerated in the [3+2] pathway, albeit with reduced efficiency, whereas the corresponding [2+2] cycloaddition is unsuccessful under the optimized conditions.

**Table 2:**
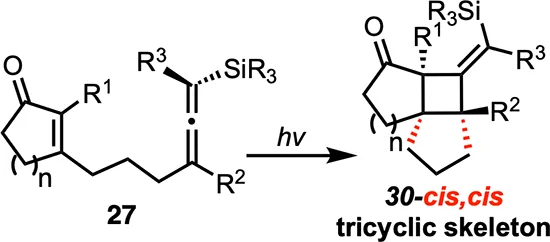

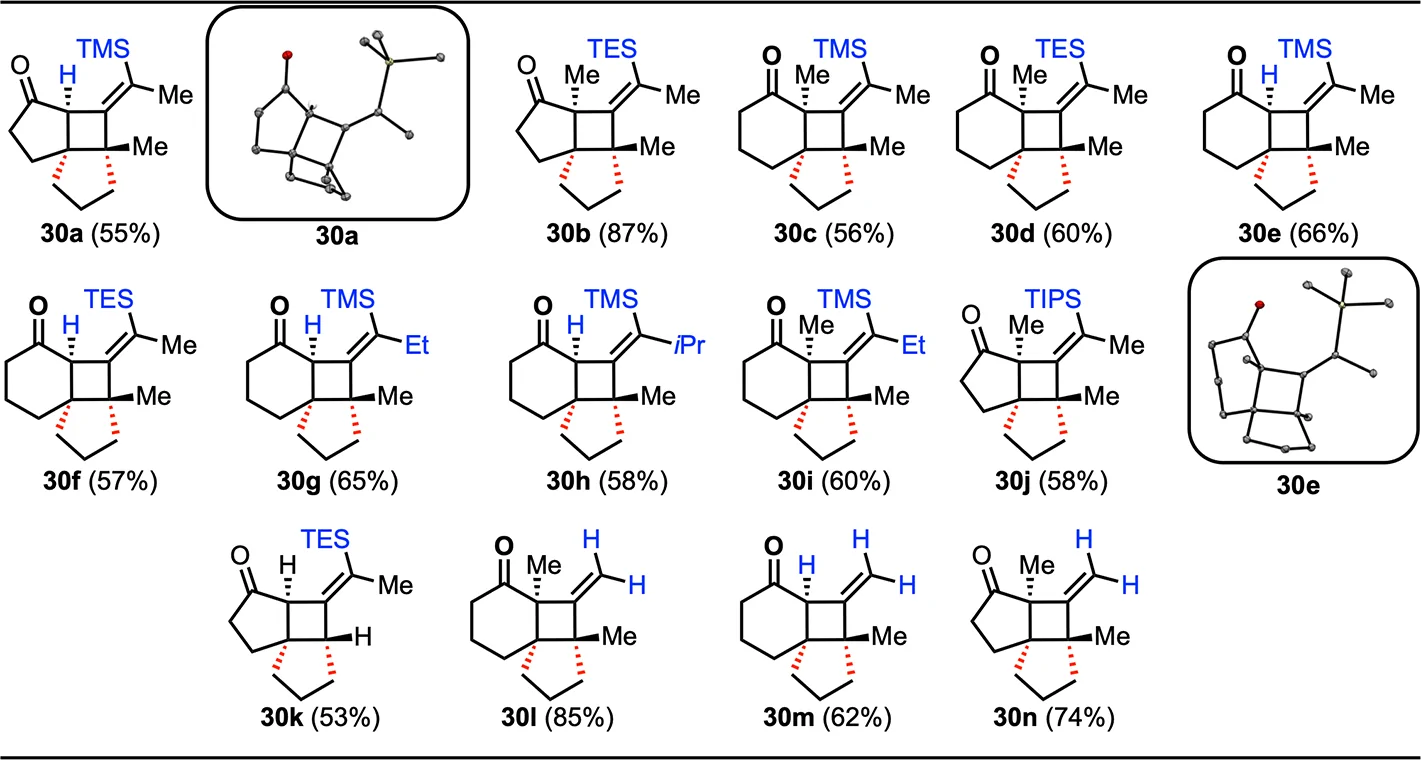
[2+2] Cycloaddition^a^ ^,^ ^b^

a Reaction conditions: SM (1 equiv., 0.5 mmol) in 0.05 M PhMe was irradiated with UV (365 nm). The reaction was stirred at room temperature until the consumption of SM, monitoring with TLC.

b The reaction was carried out at up to 2.5 mmol (SM) scale.

## Total Synthesis of (±)-Arnicenone

With the [2+2] cycloaddition-derived *cis*,*cis*-5,4,5-tricyclic intermediate **30n** in hand, we next investigated its elaboration toward (±)-arnicenone (**3**) (Scheme 3). To this end, **30n** was subjected to epoxidation with *m*CPBA to afford epoxide **31** in 87% yield with an α/β ratio of 1:4. The α-isomer was unambiguously confirmed by X-ray diffraction (XRD) analysis. Both diastereomers were then subjected to LiBr-promoted ring expansion^50^ in the presence of HMPA at elevated temperature (130 °C), furnishing the 1,4-diketone (**33**). However, further attempts to convert **33** into the corresponding α-methylated product **34** were unsuccessful. Given the unsuccessful α-methylation of **33**, we turned to an alternative strategy involving reduction of ketone **30n**, followed by hydroxyl-directed epoxidation of the *exo*-olefin with *m*CPBA, which proceeded smoothly to afford epoxide **38** with exclusive β-selectivity (Scheme 4). Treatment of this intermediate with LiBr in the presence of HMPA in toluene at 130 °C promoted rearrangement to furnish the *cis*,*cis*-angular triquinane core in 99% yield, with a diastereomeric ratio of 1:5 favoring the desired 3-hydroxyketone regioisomer (**40**) over the 4-hydroxyketone (**39**).^51^ Oxidation of **40** with IBX then delivered the corresponding 1,3-diketone (**41**) in 98% yield. Regioselective conversion of the carbonyl group into the dithiane using 1,2-ethanedithiol and BF_3_·Et_2_O proceeded efficiently to afford the protected intermediate (**42**) in 86% yield. The identity of **42** was unambiguously confirmed by X-ray diffraction (XRD) analysis. Wittig olefination employing CH_3_PPh_3_Br and *t*BuOK installed the exocyclic alkene (**43**), and subsequent TsOH-mediated olefin isomerization furnished the product (**44**) in 65% yield over two steps. Removal of the thioacetal with MeI in the presence of CaCO_3_ provided ketone (**35**) in 80% yield. Formation of the silyl enol ether followed by Pd­(OAc)_2_-mediated oxidation (Saegusa–Ito reaction)^52^ delivered the enone intermediate (**36**) in 76% yield. Finally, conjugate methyl addition using MeLi/CuI furnished the target molecule (±)-arnicenone (**3**) in 78% yield (16 steps, 9.6% overall yield). To further demonstrate the synthetic utility of the [3+2] Danheiser annulation, we also established an alternative route to (±)-arnicenone from *cis*,*cis*-**28m** (Scheme 5). Despite its modest yield (10%) in the annulation, **28m** possesses the requisite *cis*,*cis*-angular triquinane framework for direct elaboration toward the natural product. Desilylation of **28m** followed by Saegusa–Ito reaction furnished enone **36**, which underwent conjugate methylation to afford (±)-arnicenone (**3**) (7 steps, 2.1% overall yield). Thus, the *cis*,*cis*-angular triquinane generated directly through the [3+2] annulation provides an alternative entry to the natural product. In parallel, the synthetic utility of the *cis*,*trans*-[3+2] annulation product was examined through its elaboration toward the corresponding *epi*-arnicenone analogue. The sequence was successfully advanced to the late-stage *epi*-arnicenone precursor enone; however, the final conjugate methylation could not be achieved under the conditions examined (see Supporting Information for details).

**Scheme 3:**
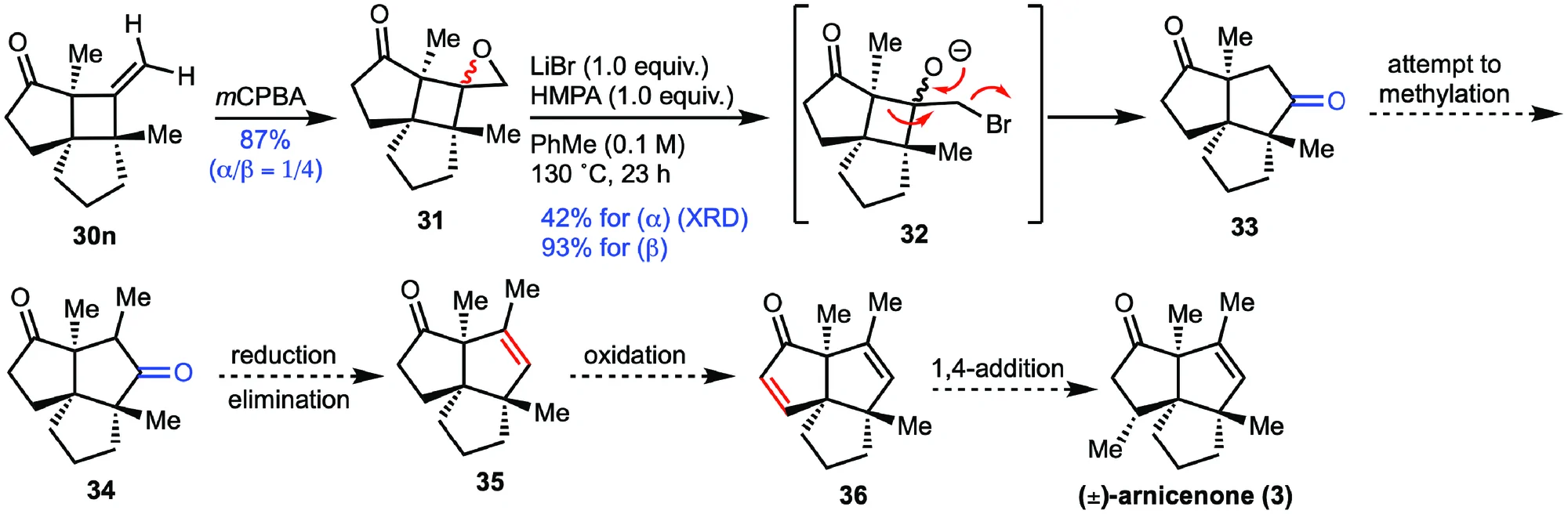
Attempt to Synthesize Methylated Ketone **34**

**Scheme 4:**
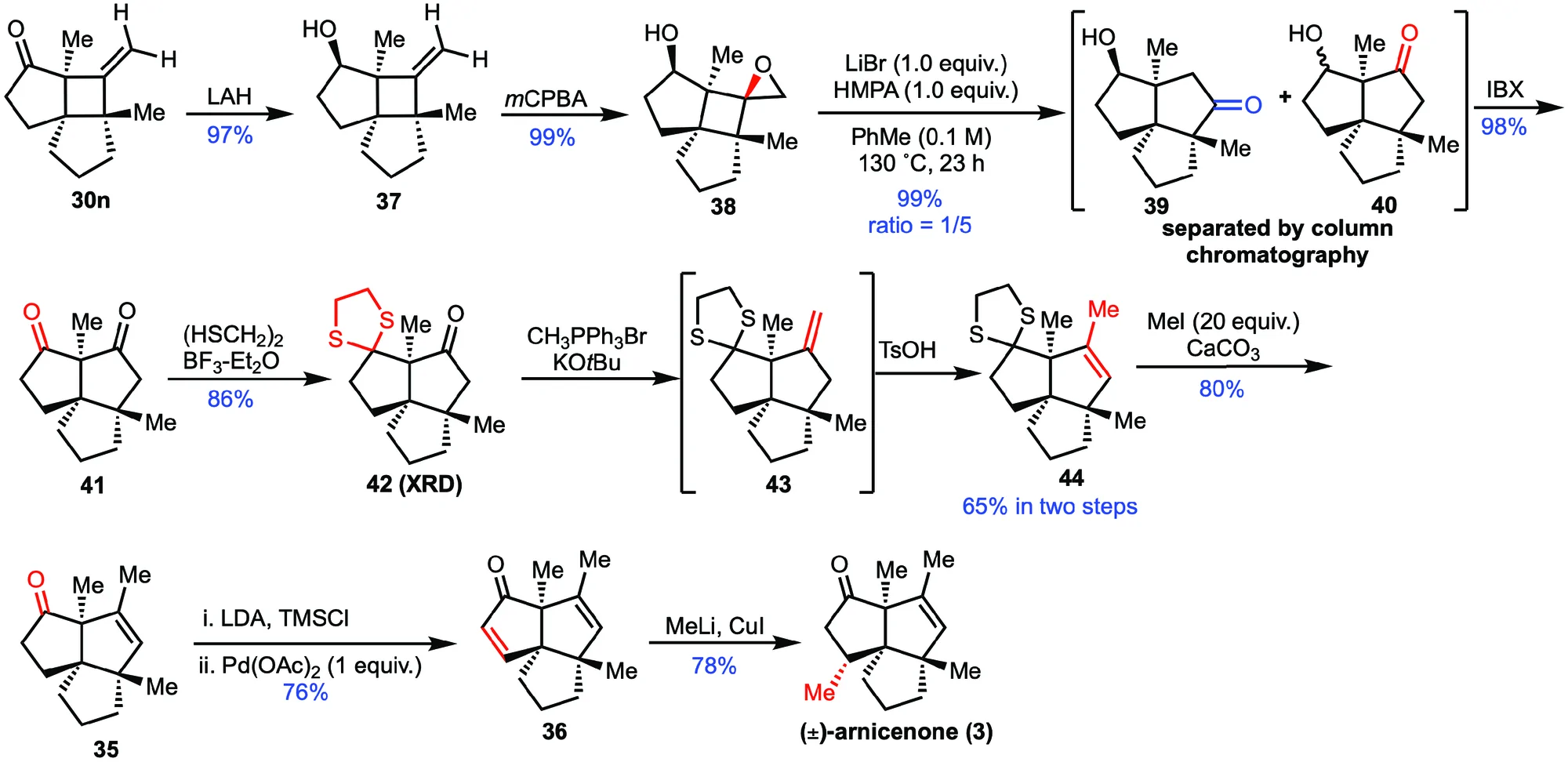
Total Synthesis of (±)-Arnicenone (**3**) via a [2+2] Cycloaddition/Ring Expansion Route

**Scheme 5:**
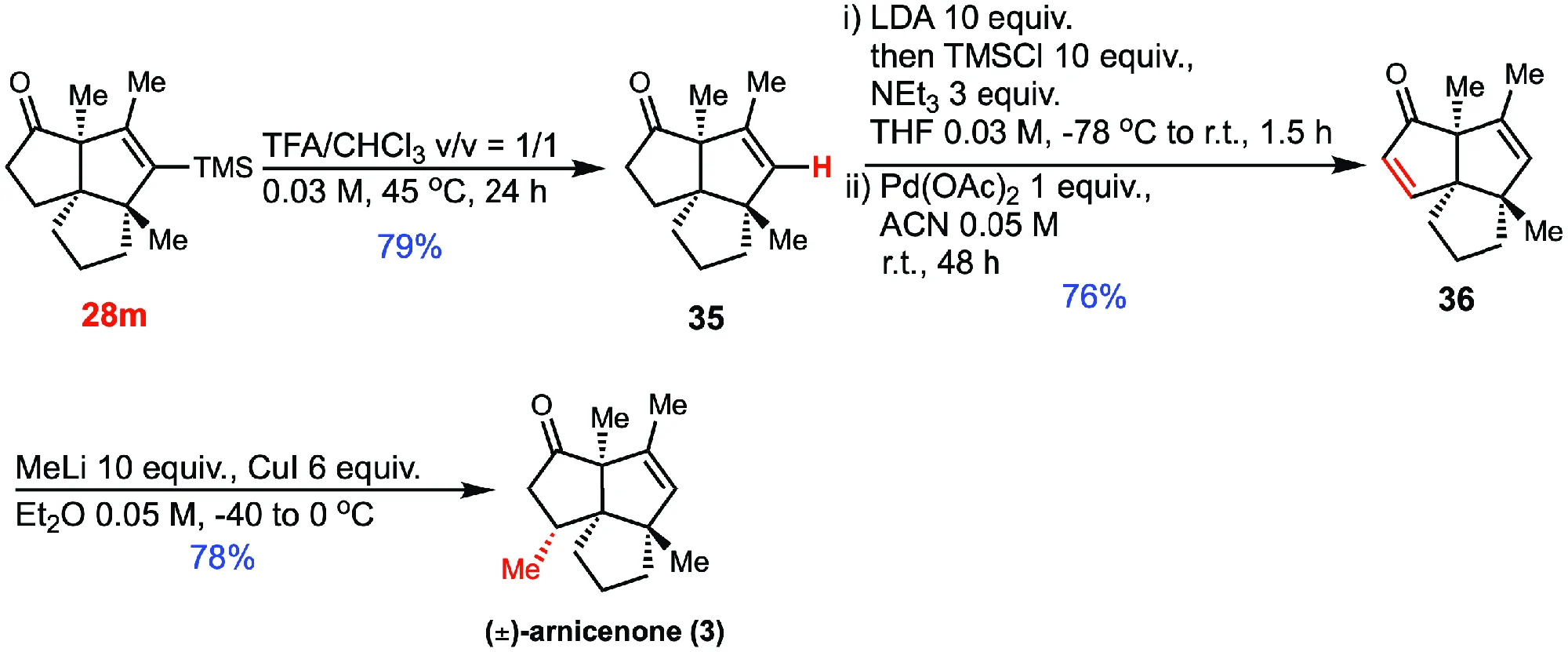
Total Synthesis of (±)-Arnicenone (**3**) via a [3+2] Annulation Route

## DFT Calculations of [3+2] Danheiser Annulation

The identity of R^1^ (Me vs H) proved crucial in controlling both the reactivity and stereochemical outcome of the [3+2] annulation. Because the present study was directed toward angular triquinane frameworks relevant to natural product synthesis, the investigation of substitution at R^1^ was focused on R^1^ = H and R^1^ = Me; larger substituents at this position were not examined. Substrates bearing R^1^ = H generally favored formation of the *cis*,*trans*-isomer, whereas introduction of R^1^ = Me resulted in diminished reactivity and reversed the diastereoselectivity, with **28m** favoring the *cis*,*cis*-isomer (*cis*,*cis*/*cis*,*trans* = 4:1). Notably, preferential formation of a *cis*,*cis*-angular tricyclic framework was also observed by Zhai and co-workers in their Danheiser annulation en route to (−)-conidiogenone. To gain insight into the origin of the stereochemical reversal observed in our system, density functional theory (DFT) calculations were performed. Mechanistically, activation of allene **45** by the Lewis acid TiCl_4_ initiates a Hosomi–Sakurai-type^53^ spirocyclization, generating vinyl cation **46** (Scheme 6). This intermediate subsequently undergoes a 1,2-silyl shift to furnish cation **47**. Finally, intramolecular enolate cyclization of **47** delivers the angular triquinane product **48**. DFT calculations support the proposal that the initial cyclization of the Lewis acid-bound starting material **45-TiCl**_**4**_ to **46** is both the rate-determining and stereochemistry-determining step of the [3+2] annulation. As shown in Figure 2, for the R^1^ = H substrate, the *trans*-forming pathway proceeds through **H_TS_** *trans* **_45–46** (20.4 kcal/mol), which is significantly lower in energy than the *cis*-forming pathway through **H_TS_** *cis* **_45–46** (23.9 kcal/mol), corresponding to ΔΔG^‡^ = 3.5 kcal/mol in favor of the *trans* pathway. This is consistent with the preferential formation of the *cis*,*trans* product. The resulting intermediates, **H_** *trans* **_46** (18.6 kcal/mol) and **H_** *cis* **_46** (18.0 kcal/mol), are close in energy, indicating that the selectivity is established primarily at the transition-state level. The subsequent 1,2-silyl shift proceeds through **H_TS_** *trans* **_46–47** (18.6 kcal/mol) and **H_TS_** *cis* **_46–47** (19.0 kcal/mol) to furnish **H_** *trans* **_47** (7.6 kcal/mol) and **H_** *cis* **_47** (4.3 kcal/mol), respectively; the small energy difference between **H_TS_** *trans* **_46–47** and **H_TS_** *cis* **_46–47** indicates that this later step does not control the product ratio.

**Scheme 6:**
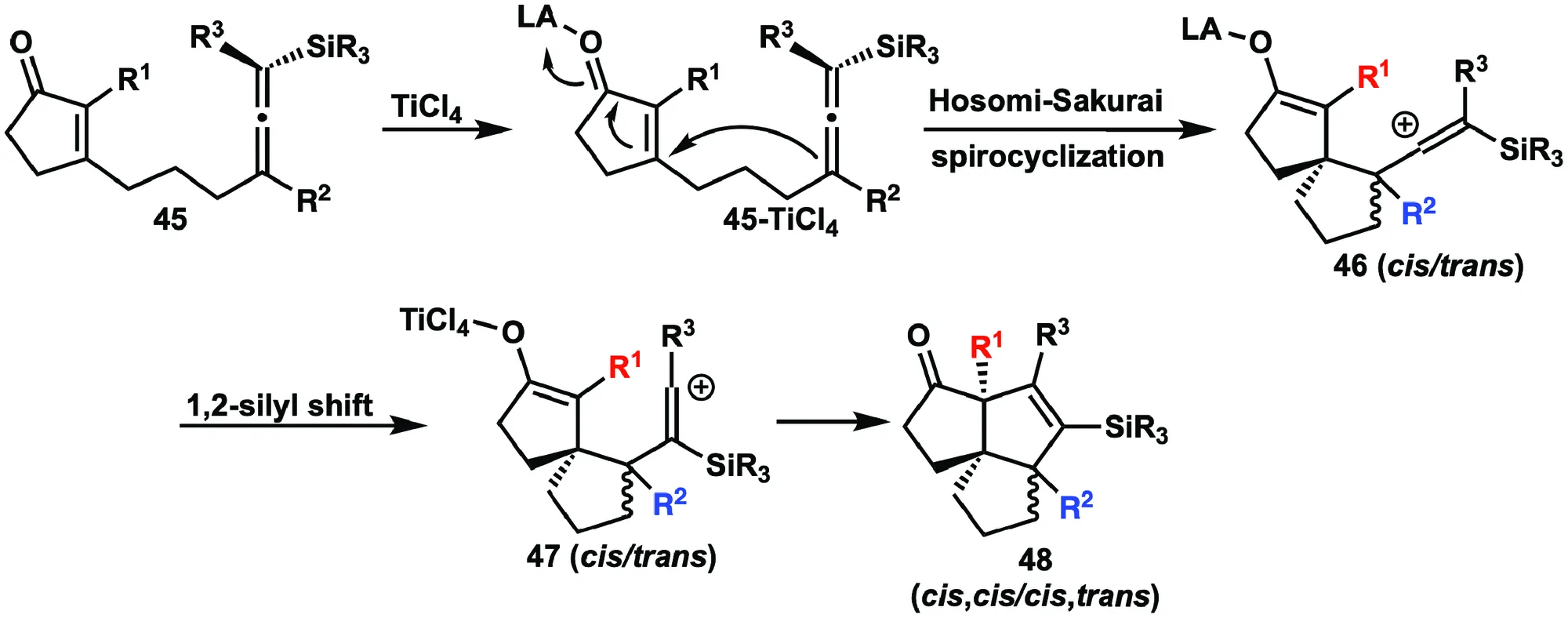
Proposed Mechanism for [3+2] Danheiser Annulation

**Figure 2:**
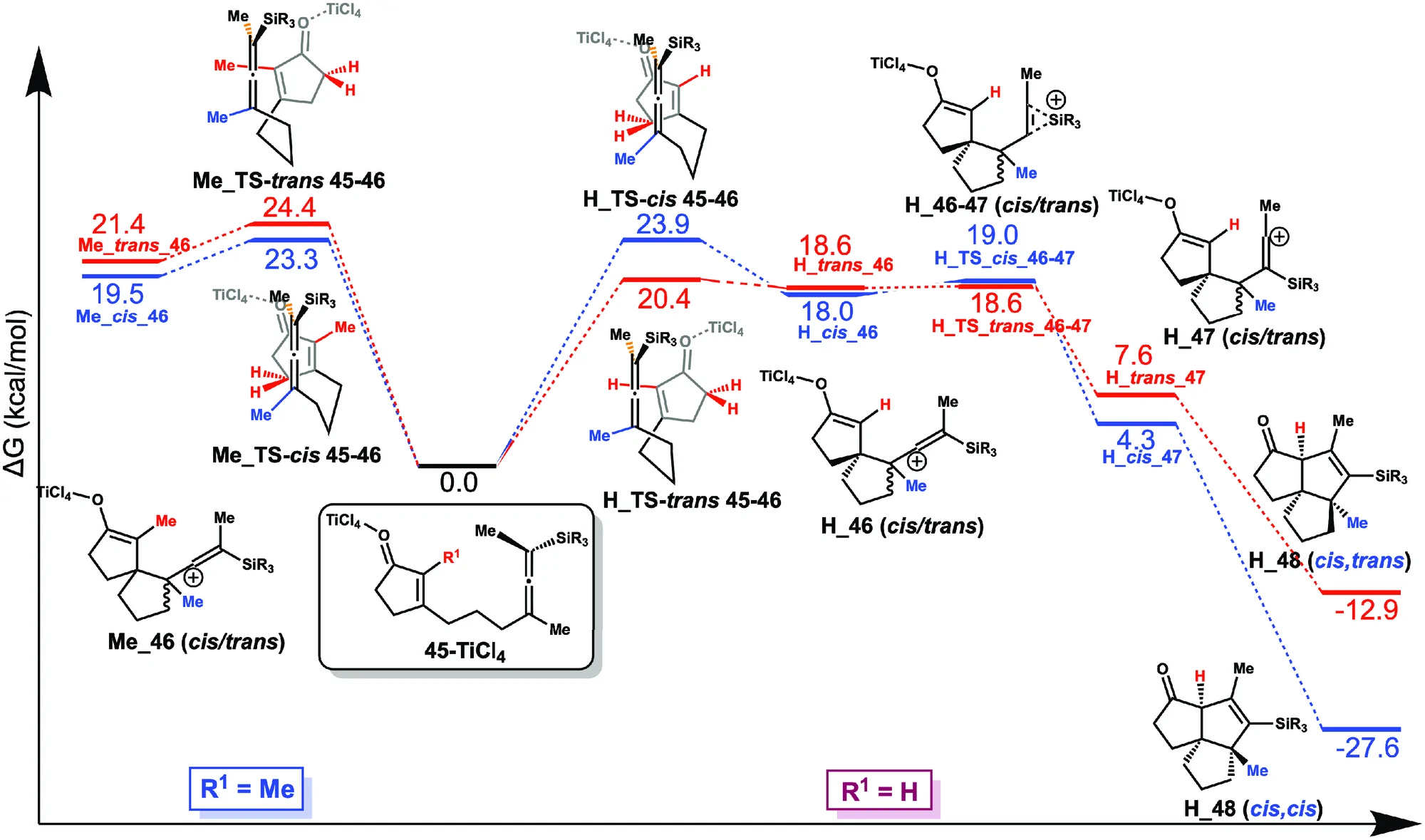
DFT calculations of [3+2] Danheiser annulation (R^1^ = H or Me, SiR_3_ = TMS).

Notably, the final product **H_48** (*cis*,*cis*)­(−27.6 kcal/mol) is substantially lower in energy than **H_48** (*cis*,*trans*)­(−12.9 kcal/mol), showing that the *cis*,*cis* isomer is thermodynamically more stable even though the *cis*,*trans* isomer is formed preferentially under kinetic control. In contrast, for the R^1^ = Me substrate, the relative ordering of the first cyclization transition states is reversed: formation of **Me_** *cis* **_46** proceeds through **Me_TS_** *cis* **_45–46** with ΔG^‡^ = 23.3 kcal/mol, whereas formation of **Me_** *trans* **_46** occurs through the slightly higher-energy **Me_TS_** *trans* **_45–46** with ΔG^‡^ = 24.4 kcal/mol, giving ΔΔG^‡^ = 1.1 kcal/mol in favor of the *cis*-forming pathway. Moreover, the lowest cyclization barrier for R^1^ = Me remains 2.9 kcal/mol higher than that for R^1^ = H, accounting for the diminished reactivity of the Me-substituted substrates. Together, these results show that changing R^1^ from H to Me both raises the barrier for cyclization of **45–TiCl_4_** and reverses the relative energies of **TS_** *cis* **_45–46** and **TS_** *trans* **_45–46**, thereby accounting for the observed differences in both reactivity and diastereoselectivity. Analysis of the computed energies provides a rationale for the observed stereochemical outcomes (Figure 3). In the **TS_** *cis* **_45–46** transition state, steric repulsion arises between the γ-CH_2_ group and R^2^, whereas in the **TS_** *trans* **_45–46** transition state, repulsion is primarily between R^1^ and R^2^. When R^1^ = Me, the Me···Me repulsion in the *trans* arrangement (24.4 kcal/mol) is greater than the γ-CH_2_···Me (R^2^) interaction in the *cis* arrangement (23.3 kcal/mol), thereby favoring formation of the *cis*,*cis* isomer. In contrast, when R^1^ = H, the R^1^···R^2^ repulsion (H···Me, 20.4 kcal/mol) is significantly lower than the γ-CH_2_···Me interaction (23.9 kcal/mol), resulting in a preference for the *cis*,*trans* isomer. Thus, the relative steric interactions in the competing transition states account for the observed diastereodivergence, with the *cis*,*trans* isomer being strongly favored when R^1^ = H.

**Figure 3:**
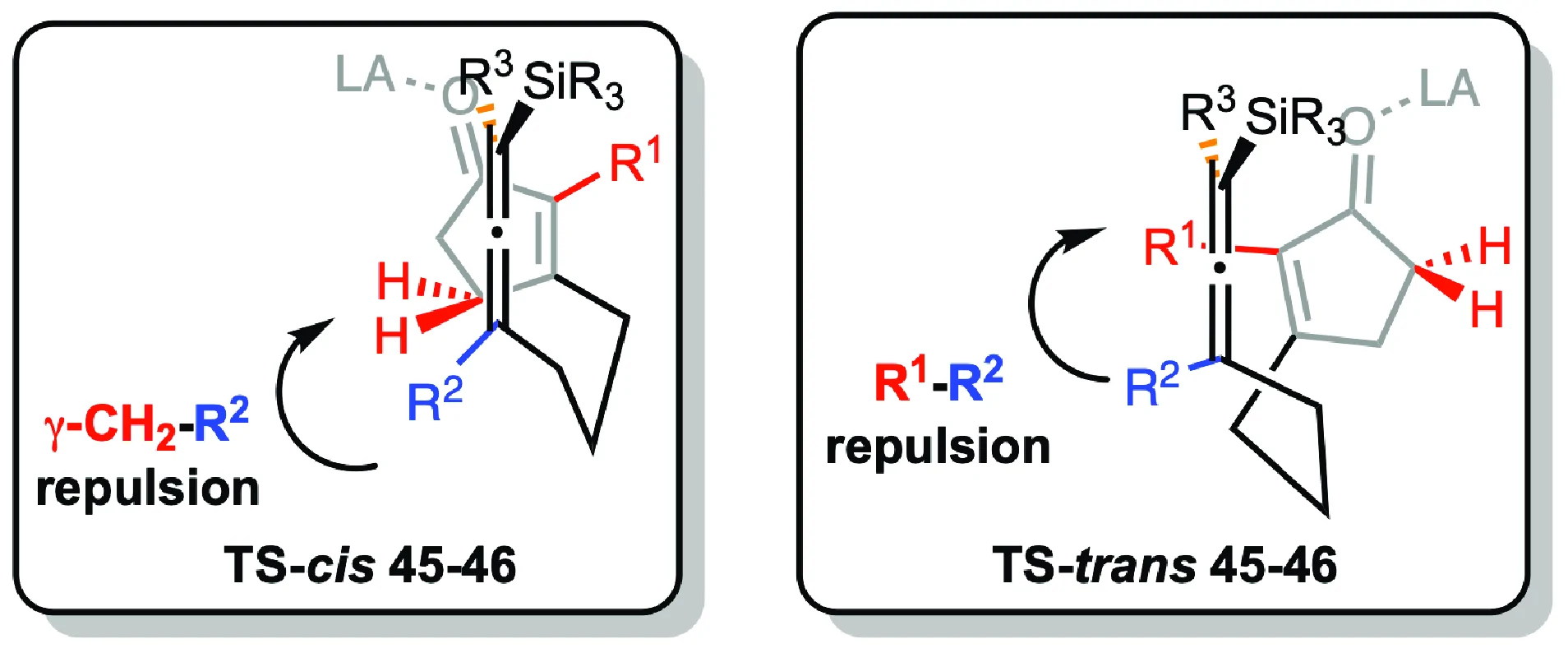
Analysis of stereochemical outcomes.

## Conclusion

In conclusion, we have developed a diastereodivergent strategy for the stereocontrolled synthesis of angular triquinanes through complementary [3+2] Danheiser annulation and [2+2] cycloaddition pathways from common allene precursors. The [3+2] annulation generally provides access to *cis*,*trans*-angular triquinanes, whereas the [2+2] cycloaddition followed by oxidative ring expansion furnishes the complementary *cis*,*cis*-fused tricyclic architectures. DFT calculations revealed that the initial spirocyclization governs both the reactivity and stereochemical outcome of the [3+2] annulation and rationalized the R^1^-dependent reversal of diastereoselectivity. The practicality of both cyclization pathways was further demonstrated on gram scale. Finally, the synthetic utility of this strategy was demonstrated through the total synthesis of (±)-arnicenone via the [2+2] cycloaddition/oxidative ring-expansion pathway, while an alternative synthesis from the *cis*,*cis*-[3+2] Danheiser annulation product **28m** further highlighted the complementary nature of the two approaches. Collectively, these results establish a versatile platform for stereodivergent access to angular triquinanes and their application to complex natural product synthesis.

## Supplementary Material















## Data Availability

The data underlying this study are available in the published Article and its Supporting Information.

## Notes

FAIR data, including the primary NMR FID files, for compounds **24**(**a-d**), **25**(**a-k**), **26**(**a-n**), **SI-2**, **SI-3**(**a-k**), **SI**(**5–12**), **28**(**a-m**), **29**(**a-d**), **30**(**a-n**), **31**(**α/β**), **33**, **35**, **36**, **37**, **38**, **39**, **40**, **41**, **42**, **43**, **44**, and **3**(**arnicenone**) (ZIP)
(ZIP)
(ZIP)
(ZIP)
(ZIP)
NMR spectra of synthesized compounds (PDF)
General information, tabulated data of synthesized compounds, and X-ray crystallographic data of synthesized compounds (PDF)
