# Supplementary material for: Diastereodivergent Access to cis,cis- and cis,trans-Angular Triquinanes: Total Synthesis of (±)-Arnicenone
Source: J Am Chem Soc. 2026 Sep 22;148(39):41724–32. doi: 10.1021/jacs.6c09019 (PMC13651531; doi:10.1021/jacs.6c09019)
Supplement: Supplementary file 1 [file ja6c09019_si_006.pdf]

III. NMR Spectra for the Synthesized Compounds

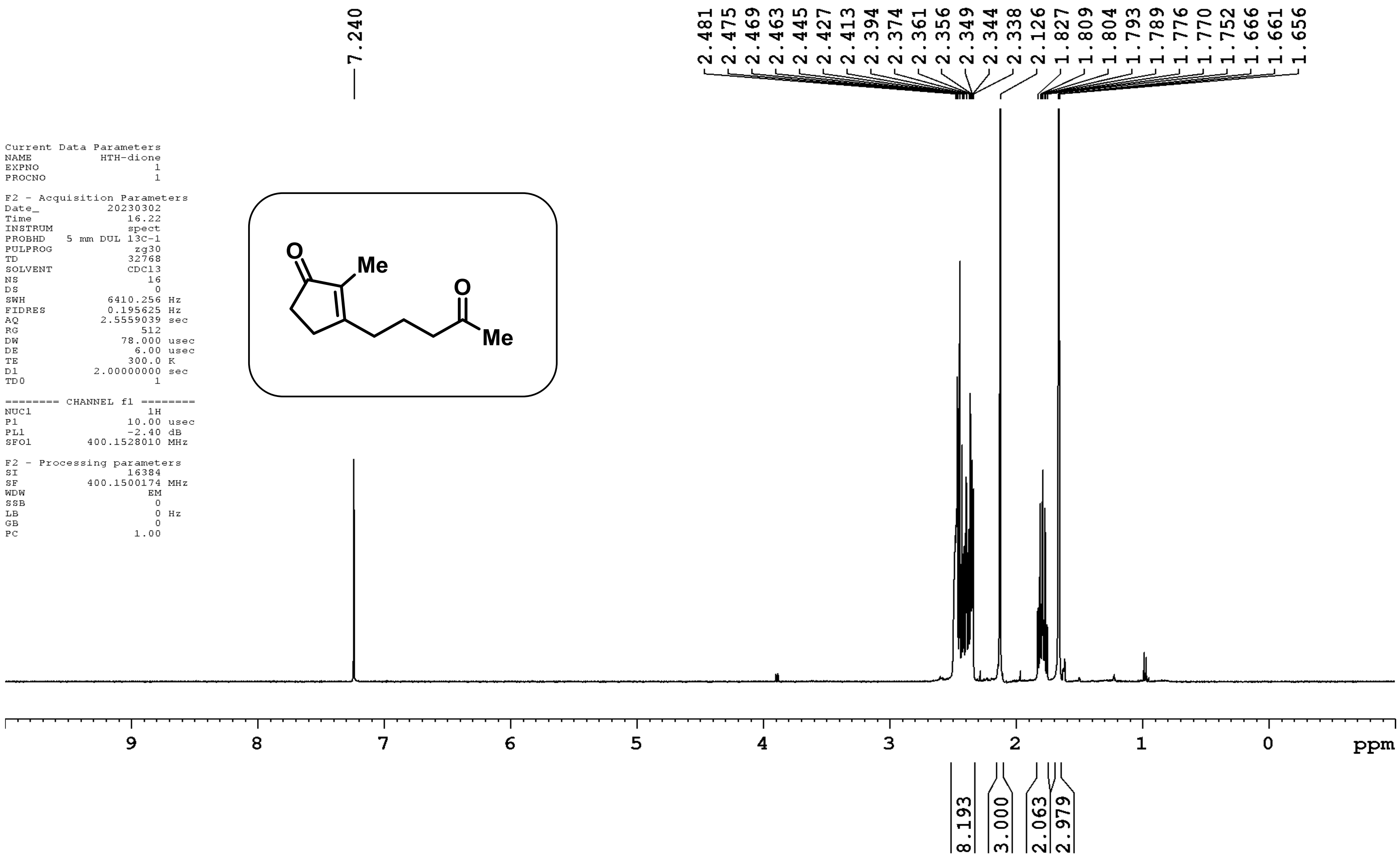

Figure 1. ^{1}H NMR (400 MHz, $CDCl_3$) Danheiser transposition (**24a**)

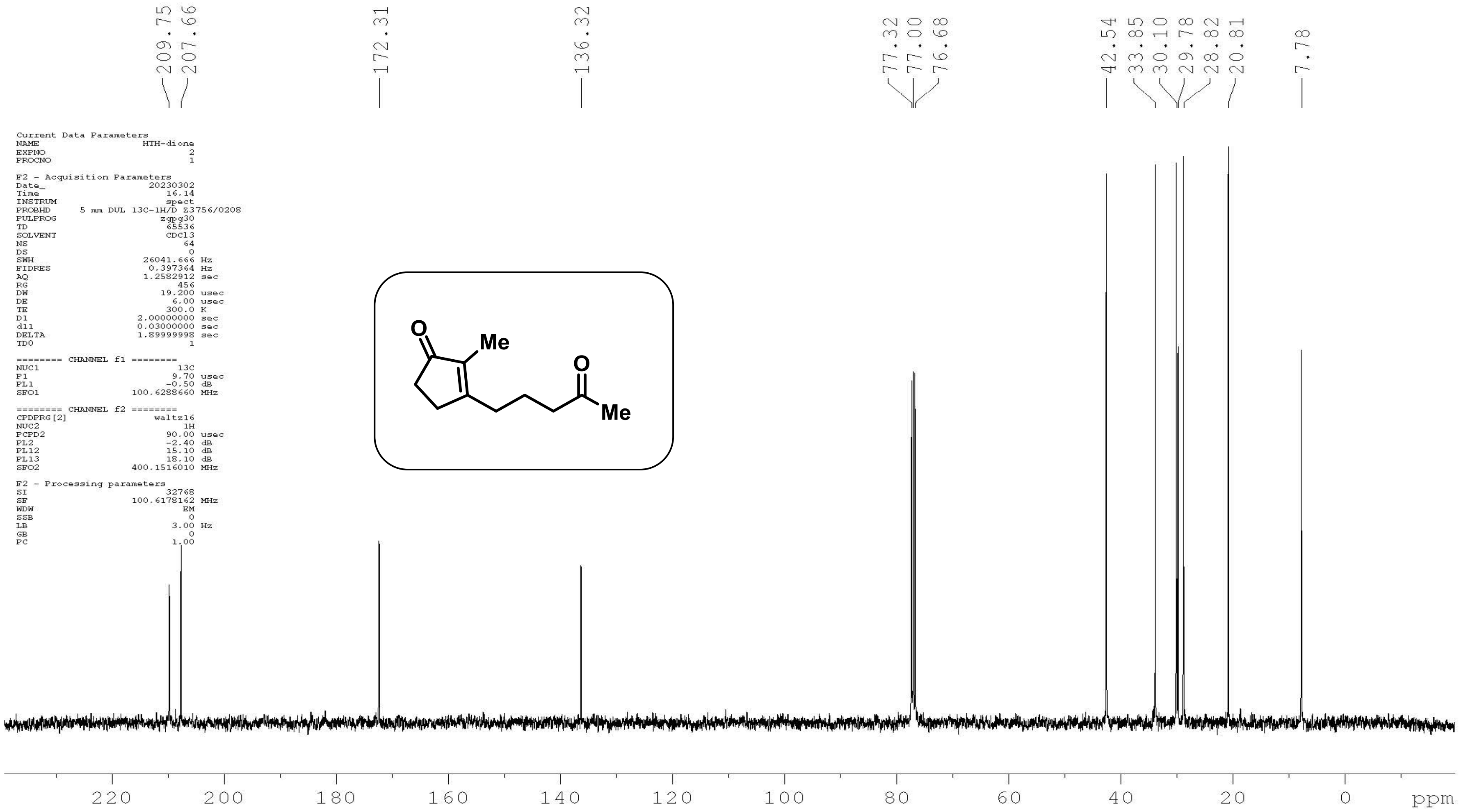

Figure 2. ^{13}C NMR (100 MHz, $CDCl_3$) Danheiser transposition (**24a**)

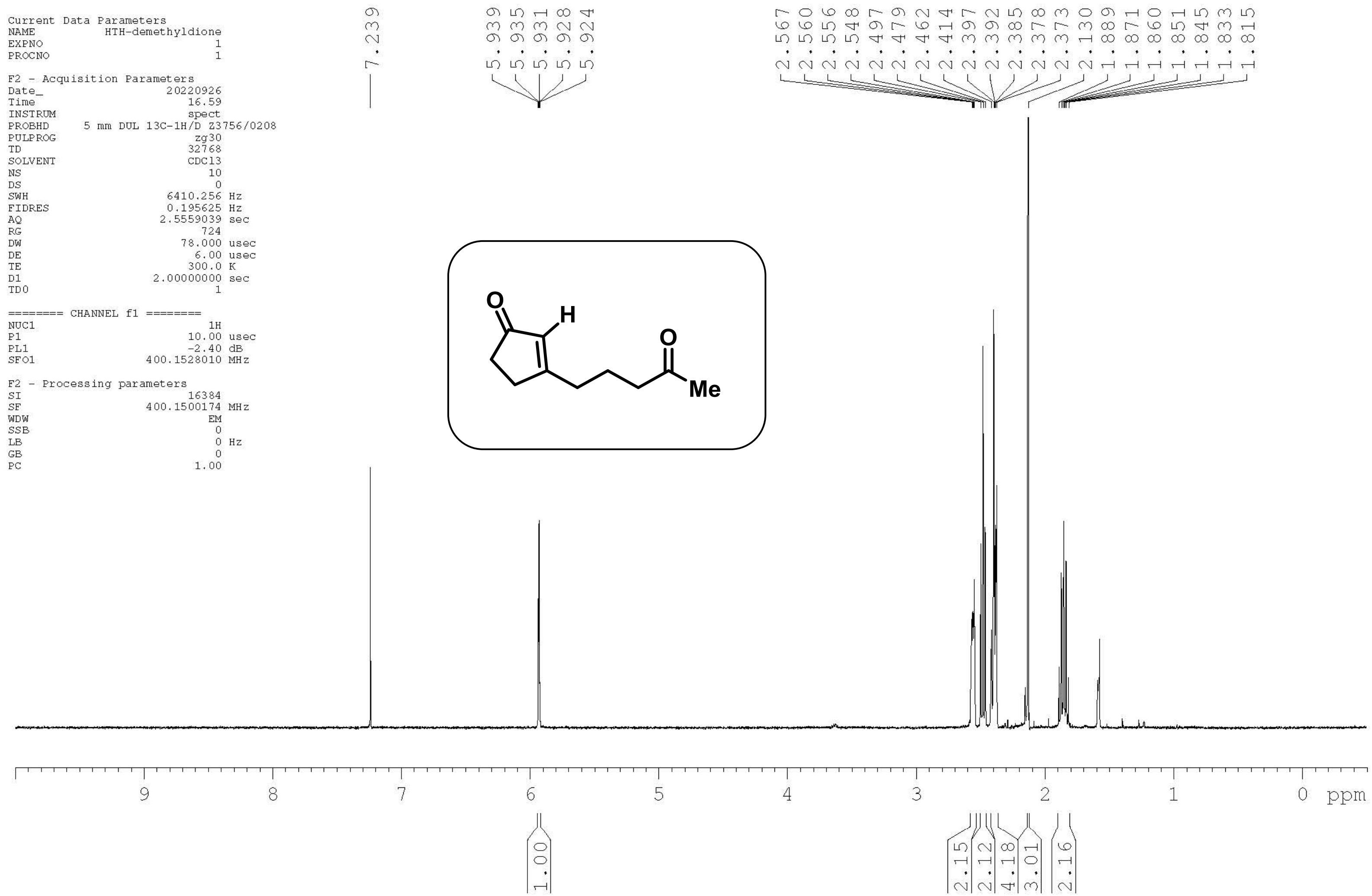

Figure 3. ^{1}H NMR (400 MHz, $CDCl_3$) Danheiser transposition (**24b**)

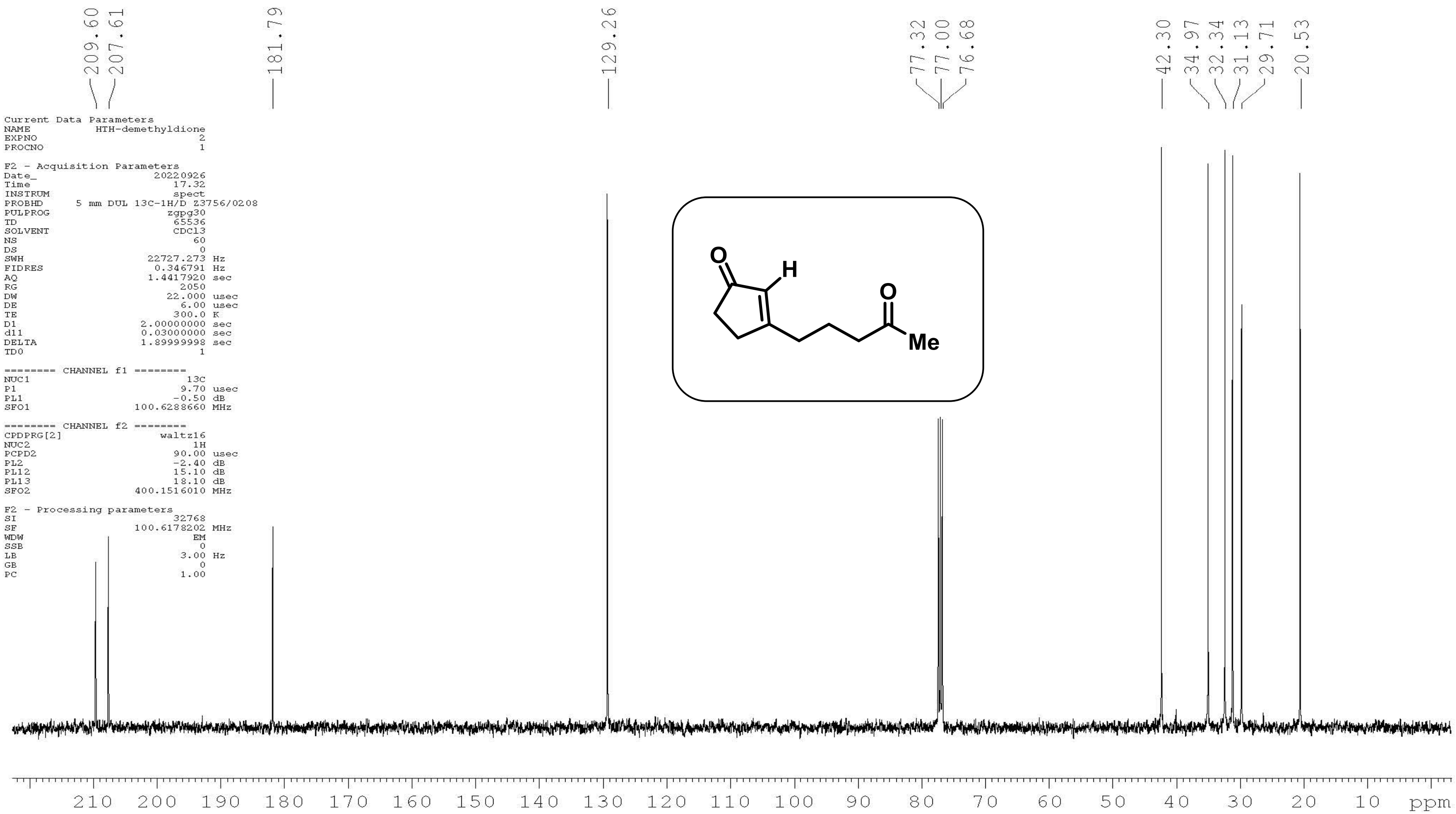

Figure 4. ^{13}C NMR (100 MHz, $CDCl_3$) Danheiser transposition (**24b**)

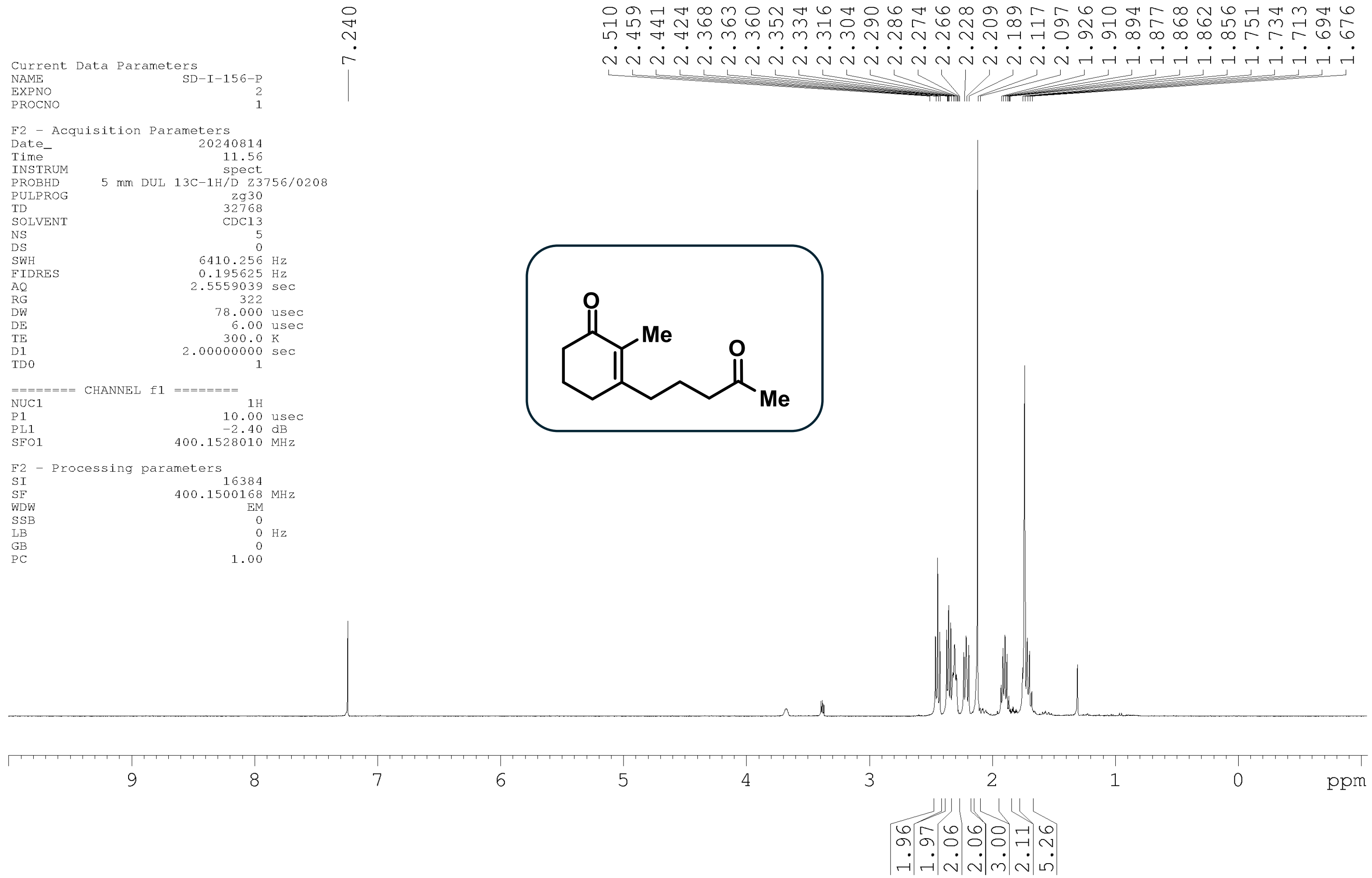

Figure 5. ^{1}H NMR (400 MHz, $CDCl_3$) Danheiser transposition (**24c**)

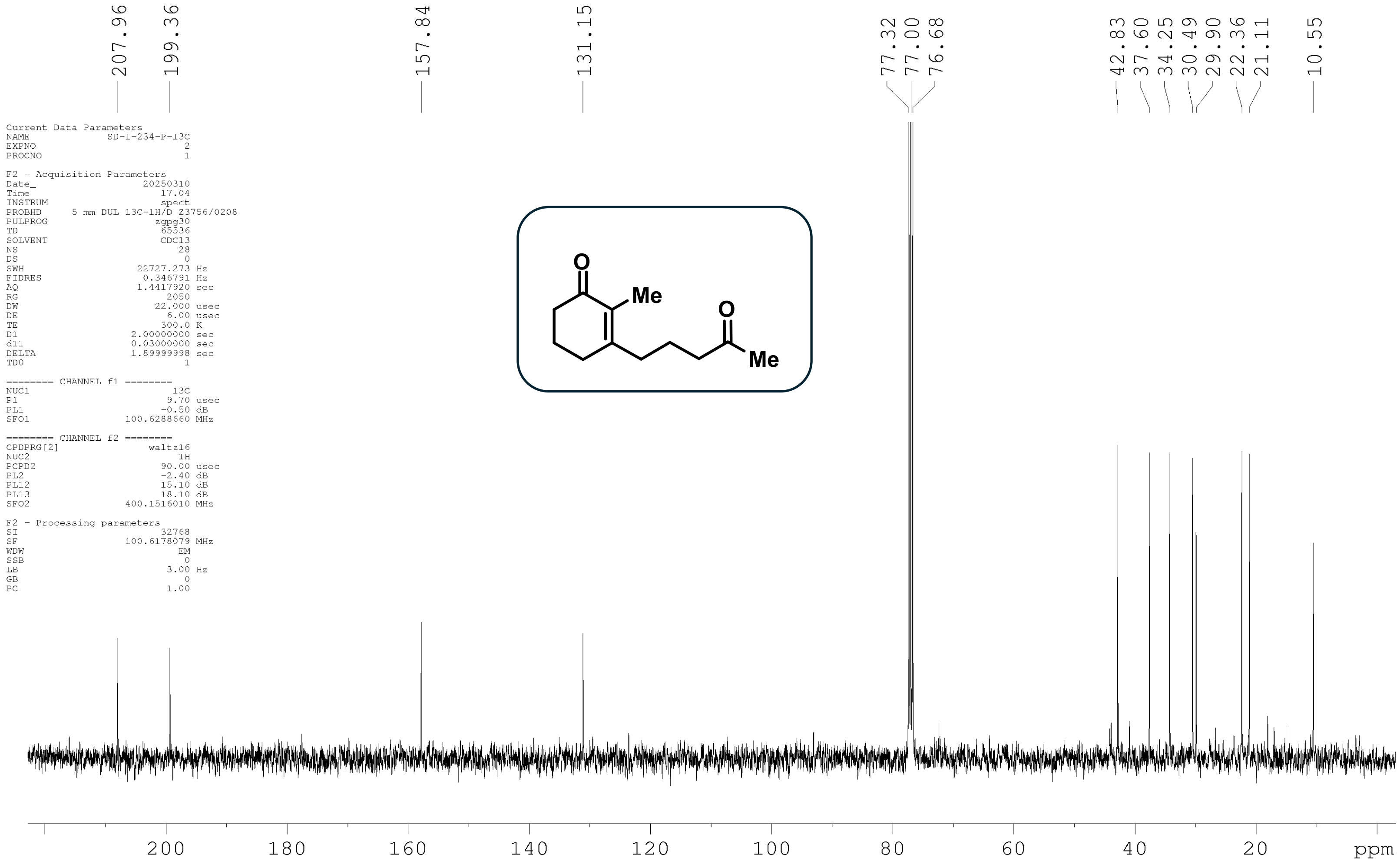

Figure 6. ^{13}C NMR (100 MHz, $CDCl_3$) Danheiser transposition (**24c**)

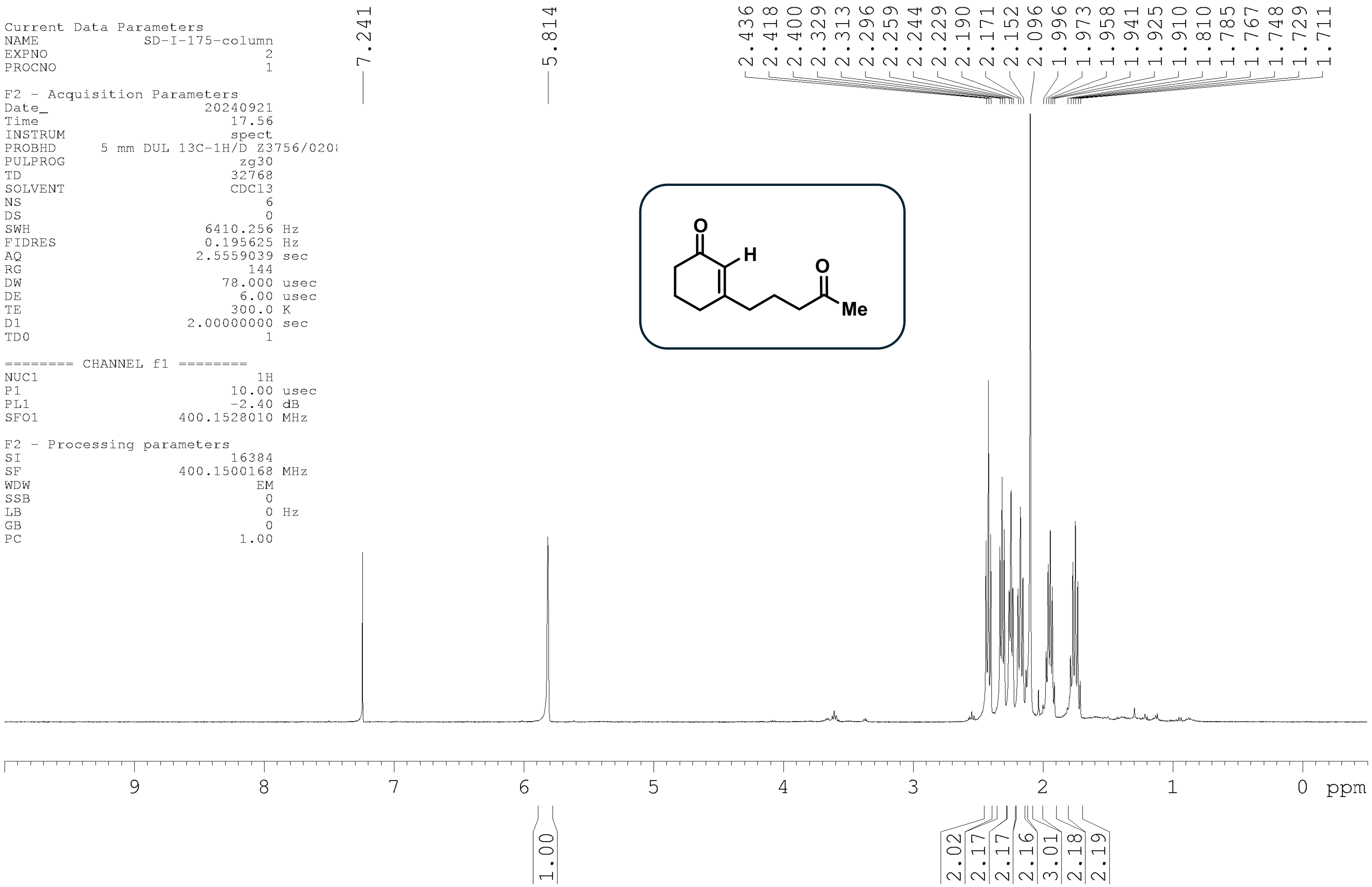

Figure 7. ^{1}H NMR (400 MHz, $CDCl_3$) Danheiser transposition (**24d**)

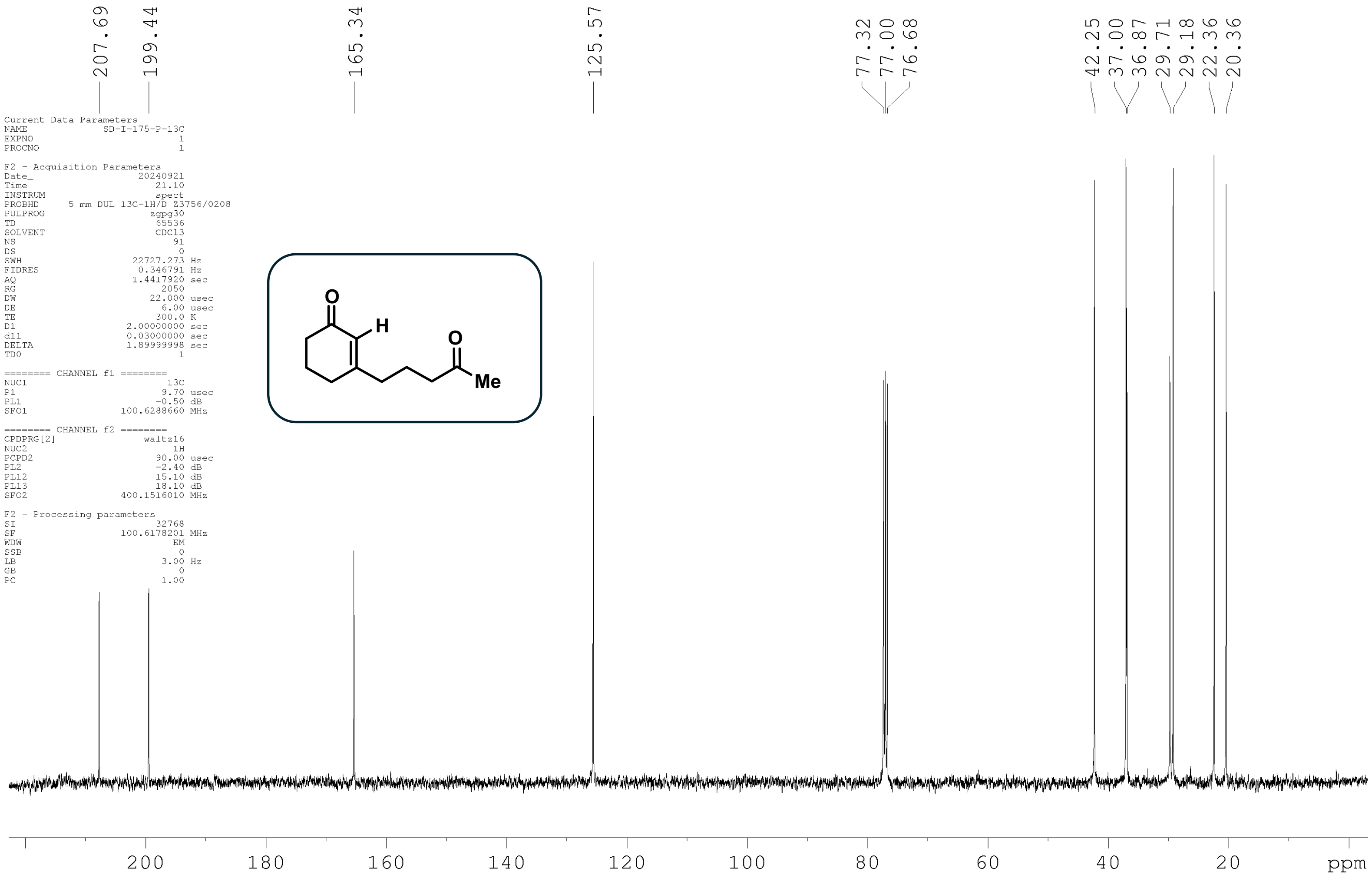

Figure 8. ^{13}C NMR (100 MHz, $CDCl_3$) Danheiser transposition (**24d**)

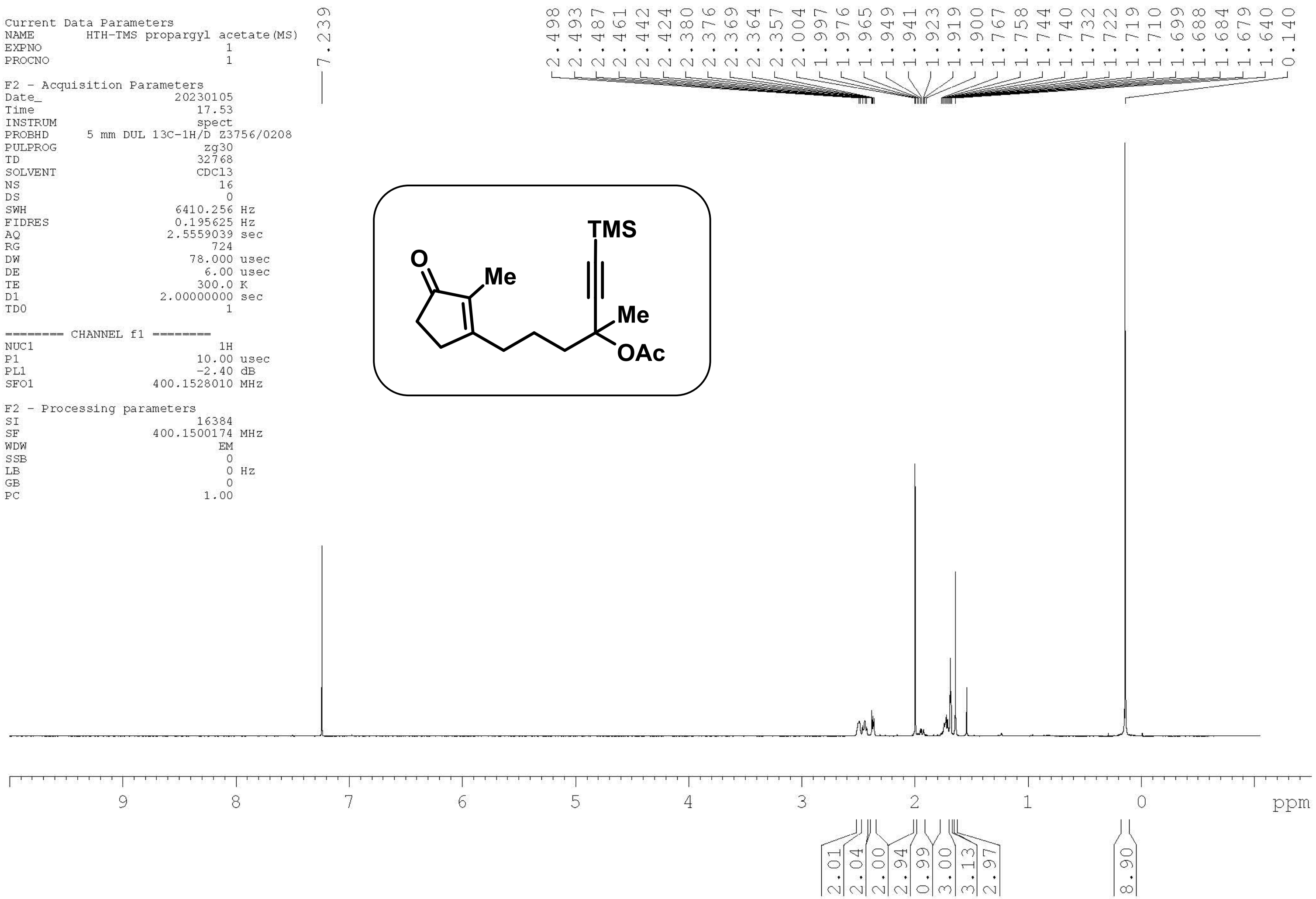

Figure 9. ^{1}H NMR (400 MHz, $CDCl_3$) (**25a**)

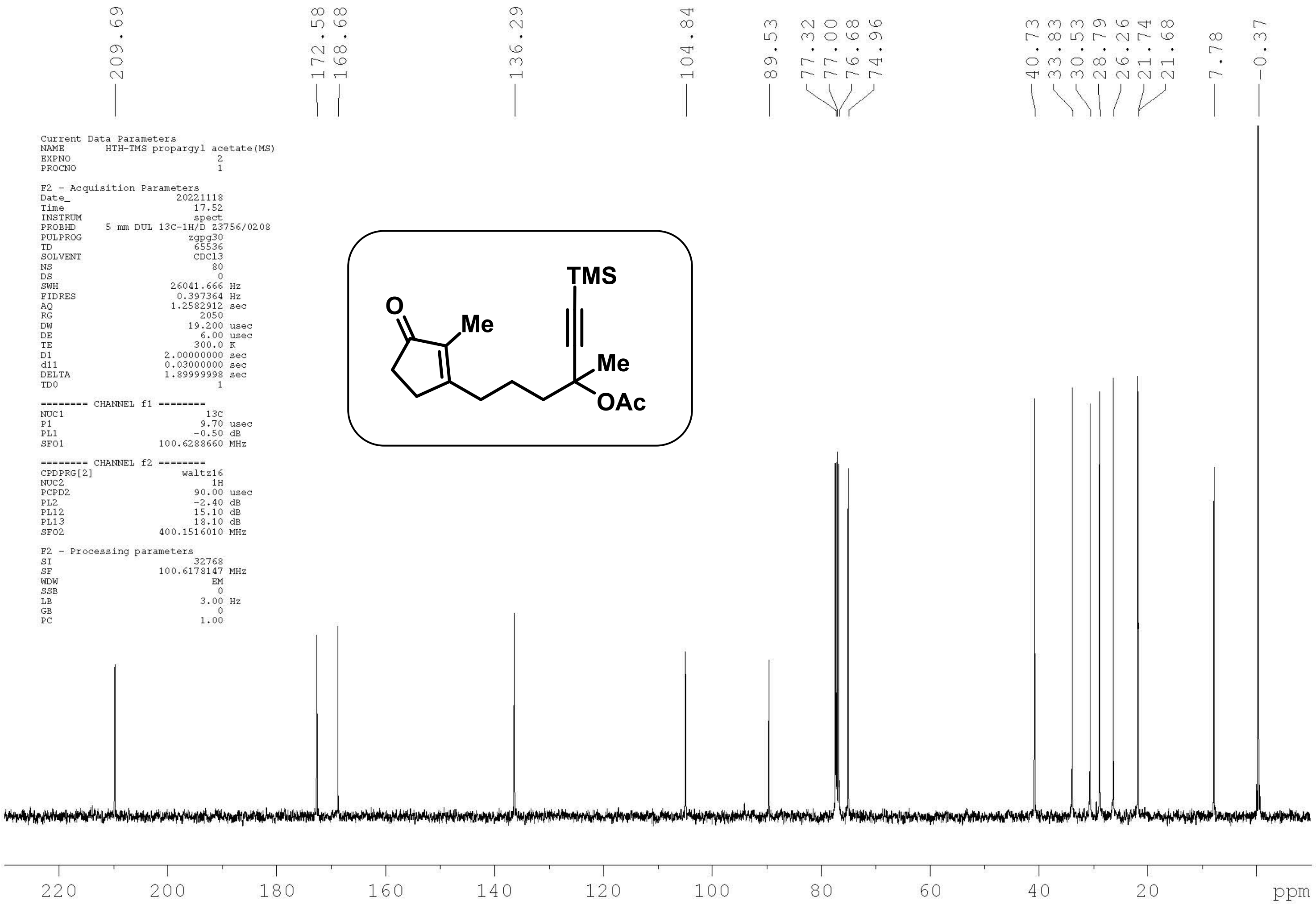

Figure 10. ^{13}C NMR (100 MHz, $CDCl_3$) (**25a**)

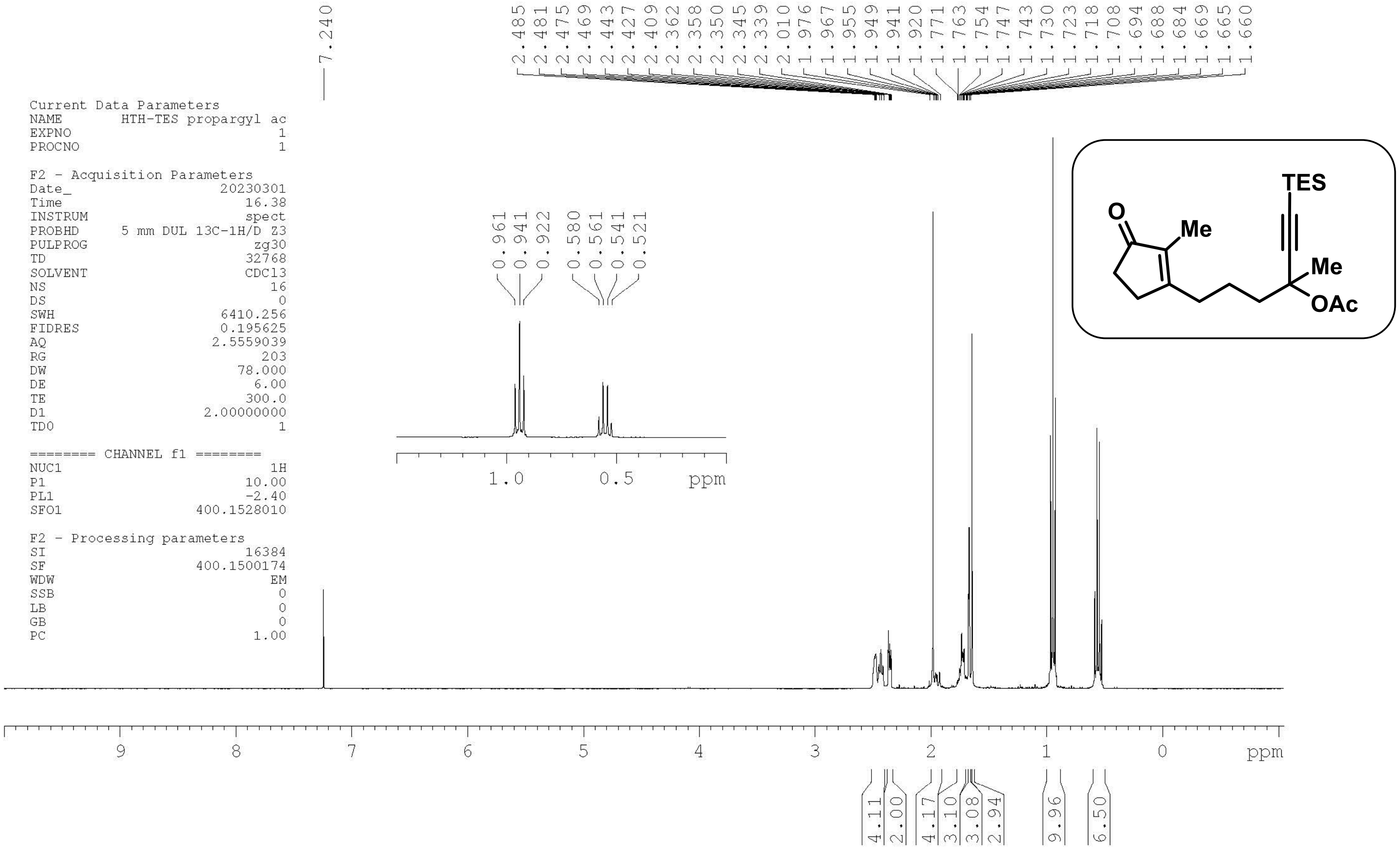

Figure 11. ^1H NMR (400 MHz, $CDCl_3$) (**25b**)

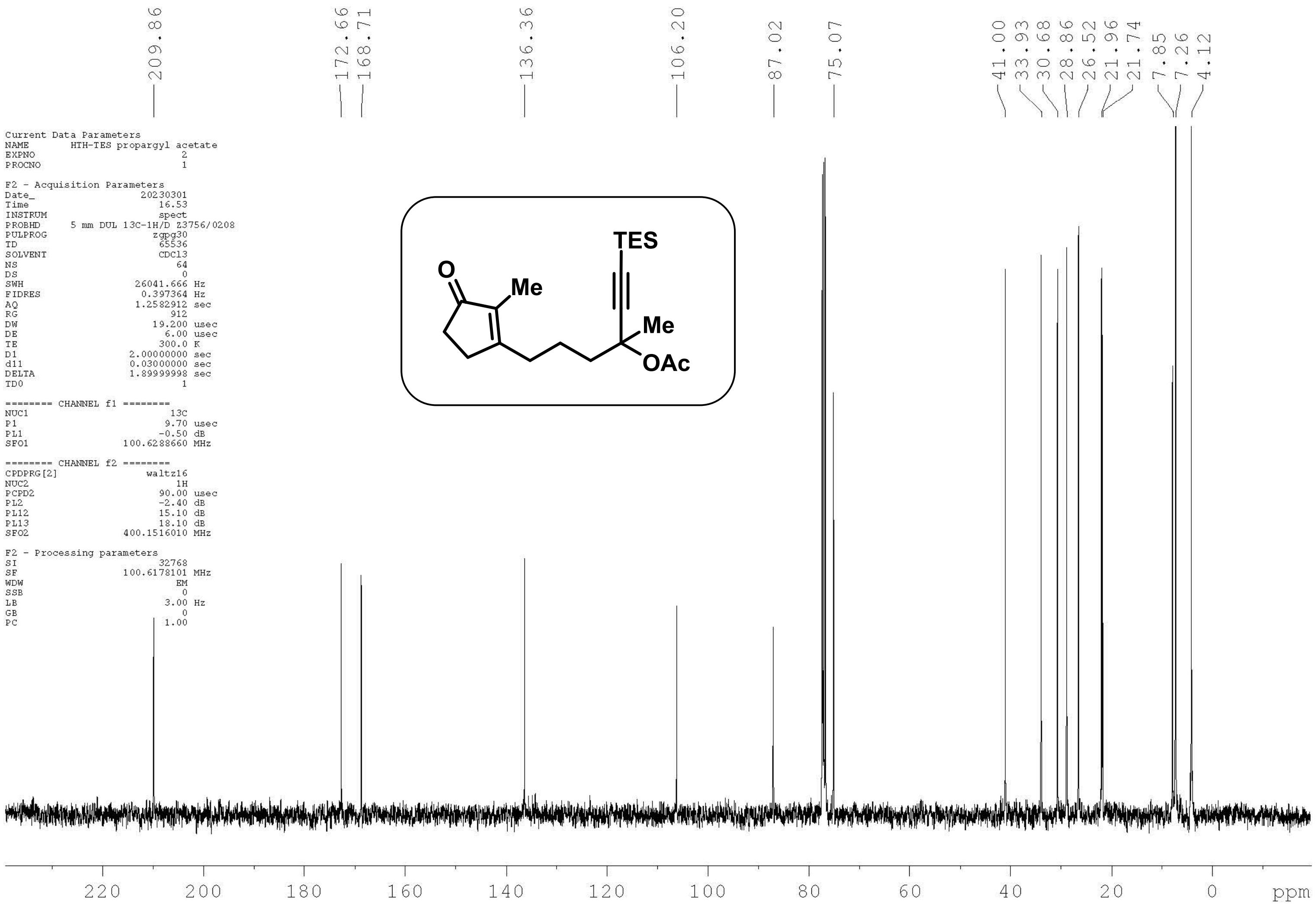

Figure 12. ^{13}C NMR (100 MHz, $CDCl_3$) (**25b**)

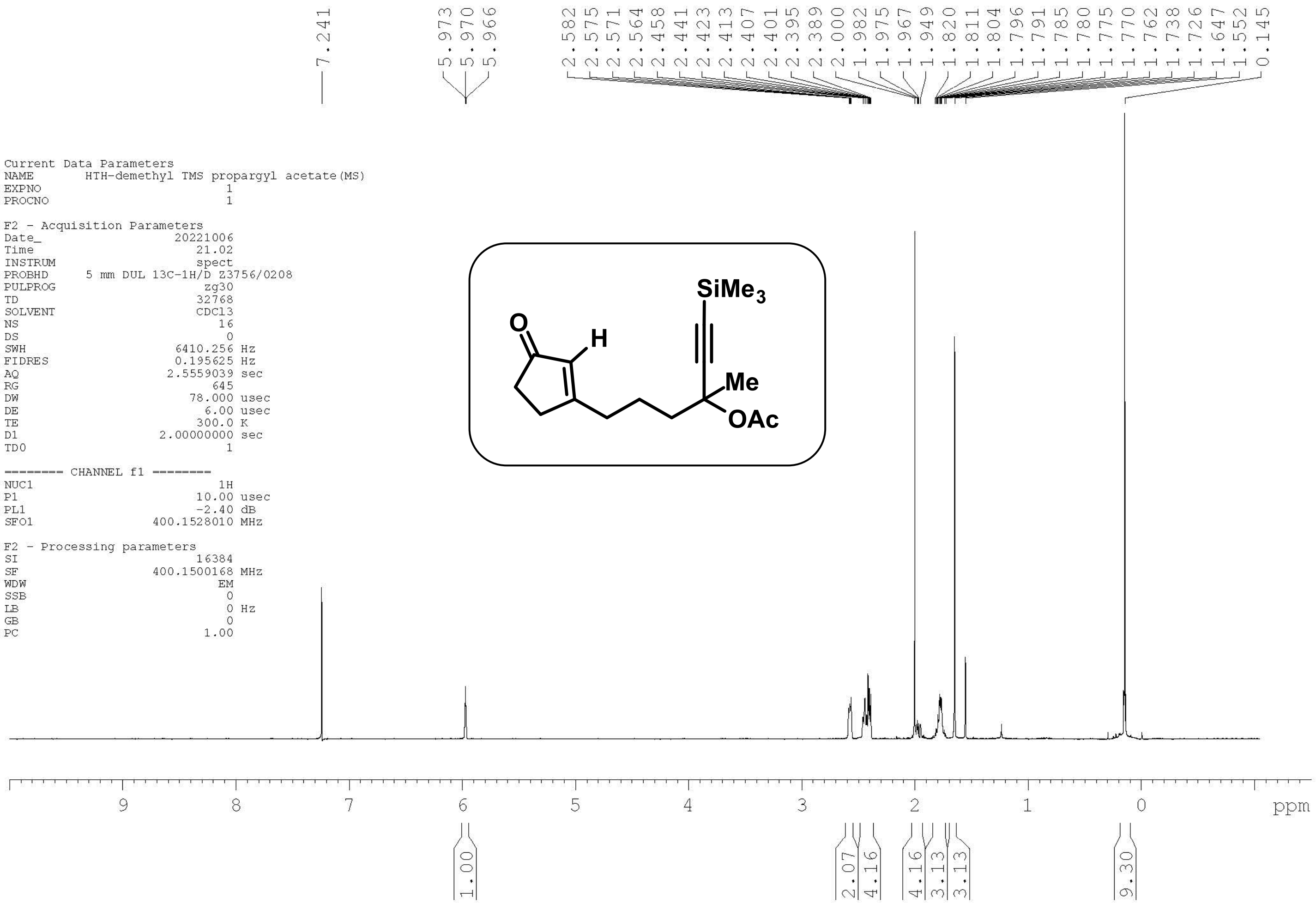

Figure 13. ^{1}H NMR (400 MHz, $CDCl_3$) (**25c**)

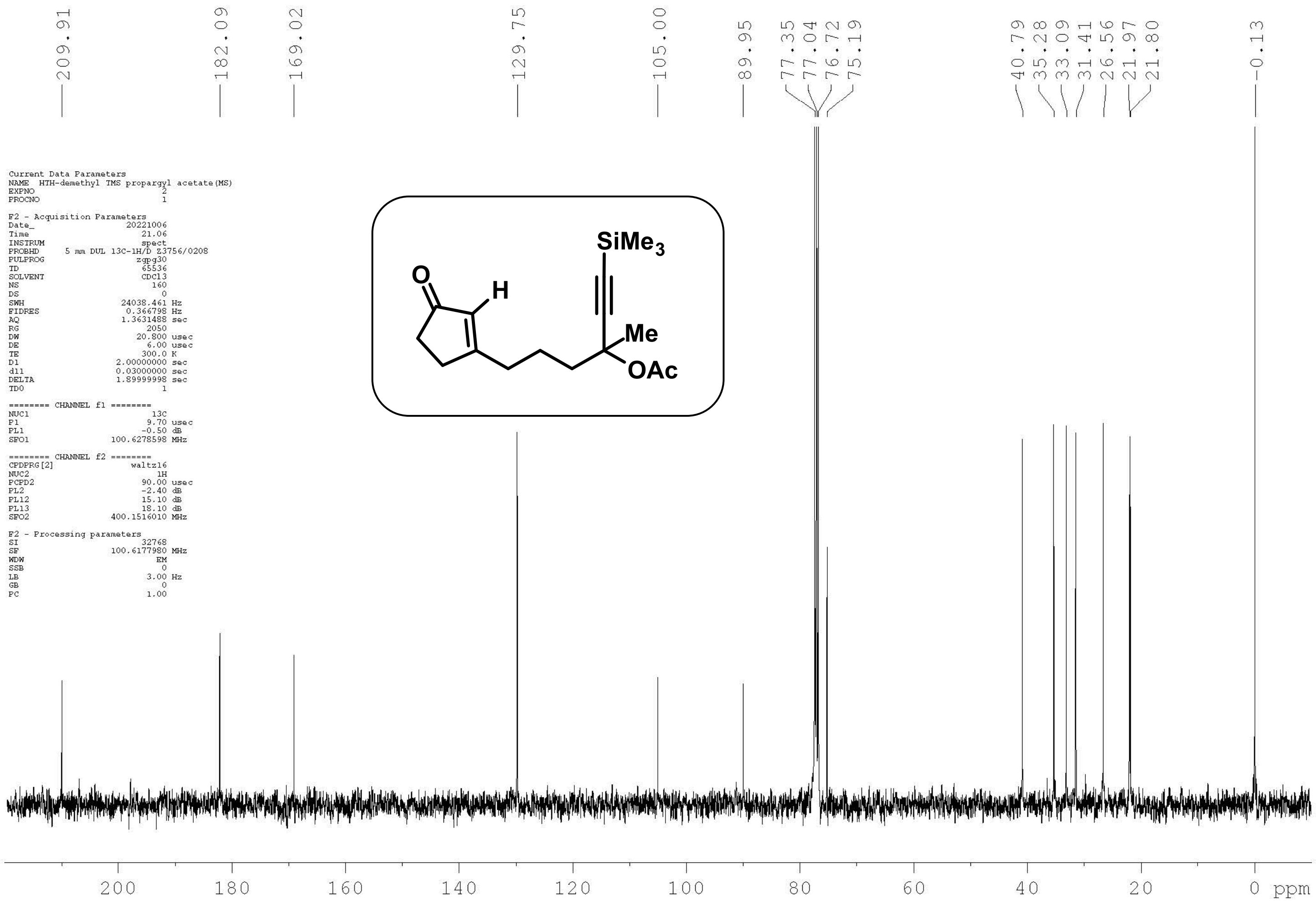

Figure 14. ^{13}C NMR (100 MHz, $CDCl_3$) (**25c**)

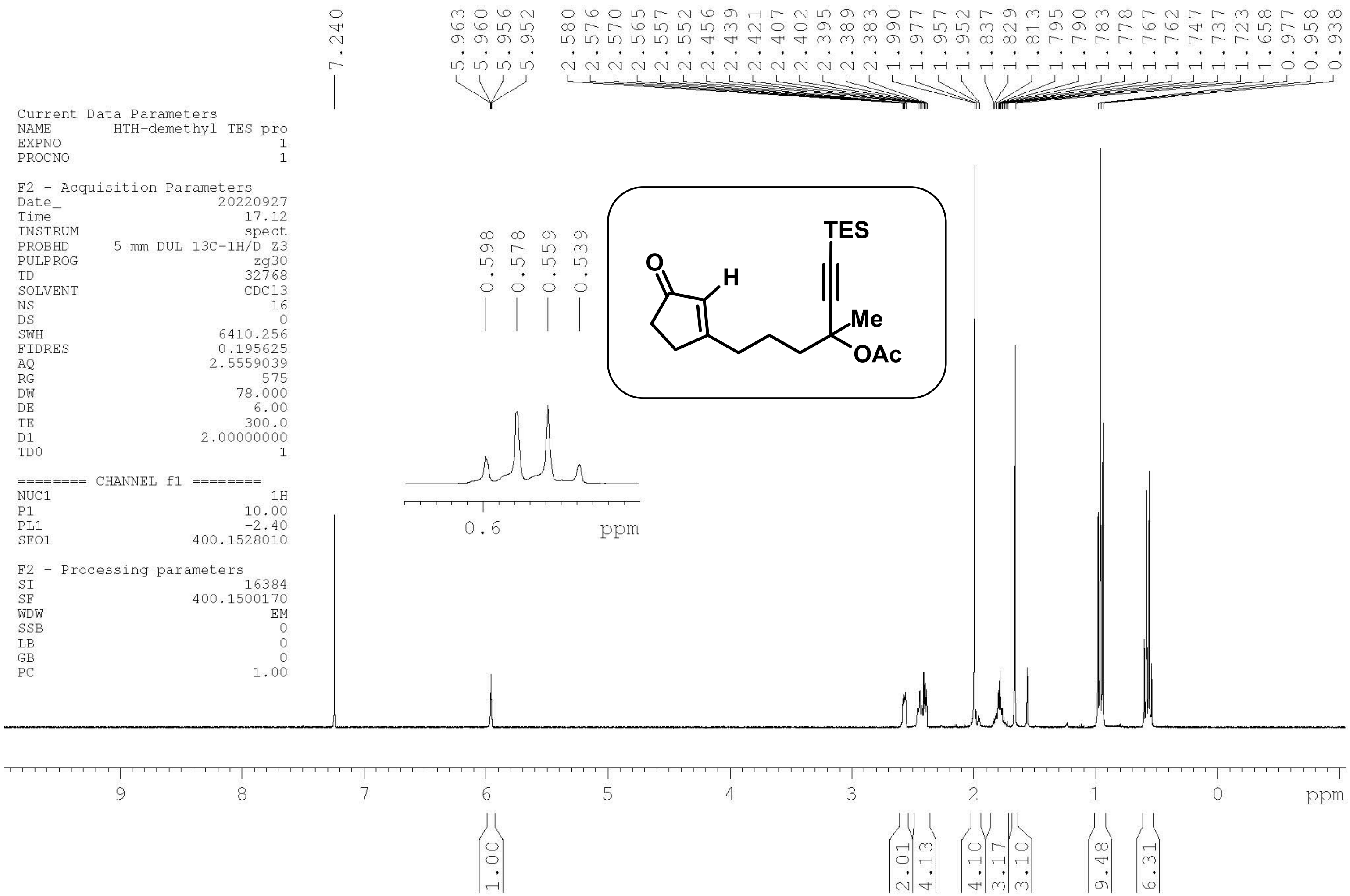

Figure 15. ^{1}H NMR (400 MHz, $CDCl_3$) (**25d**)

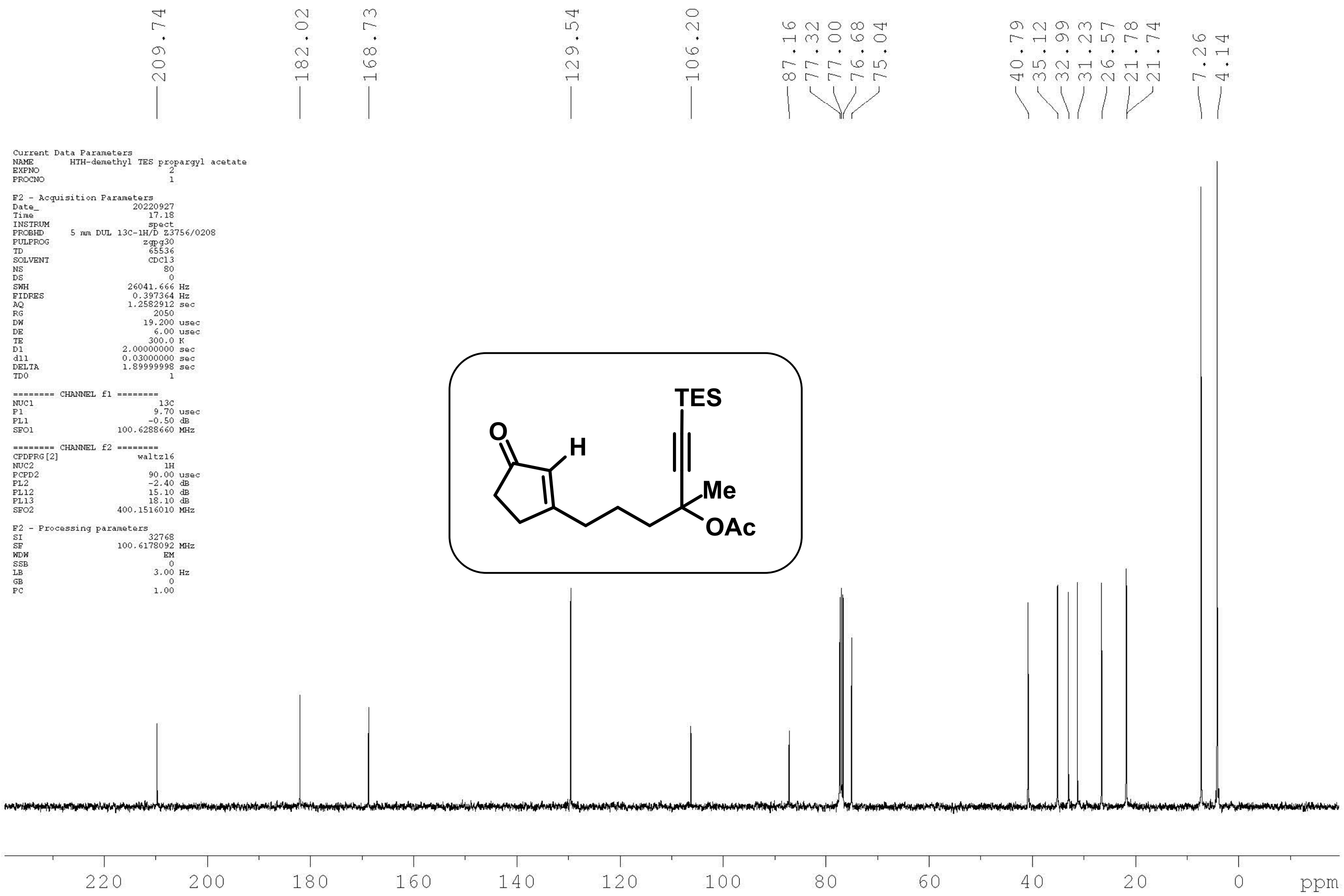

Figure 16. ^{13}C NMR (100 MHz, $CDCl_3$) (**25d**)

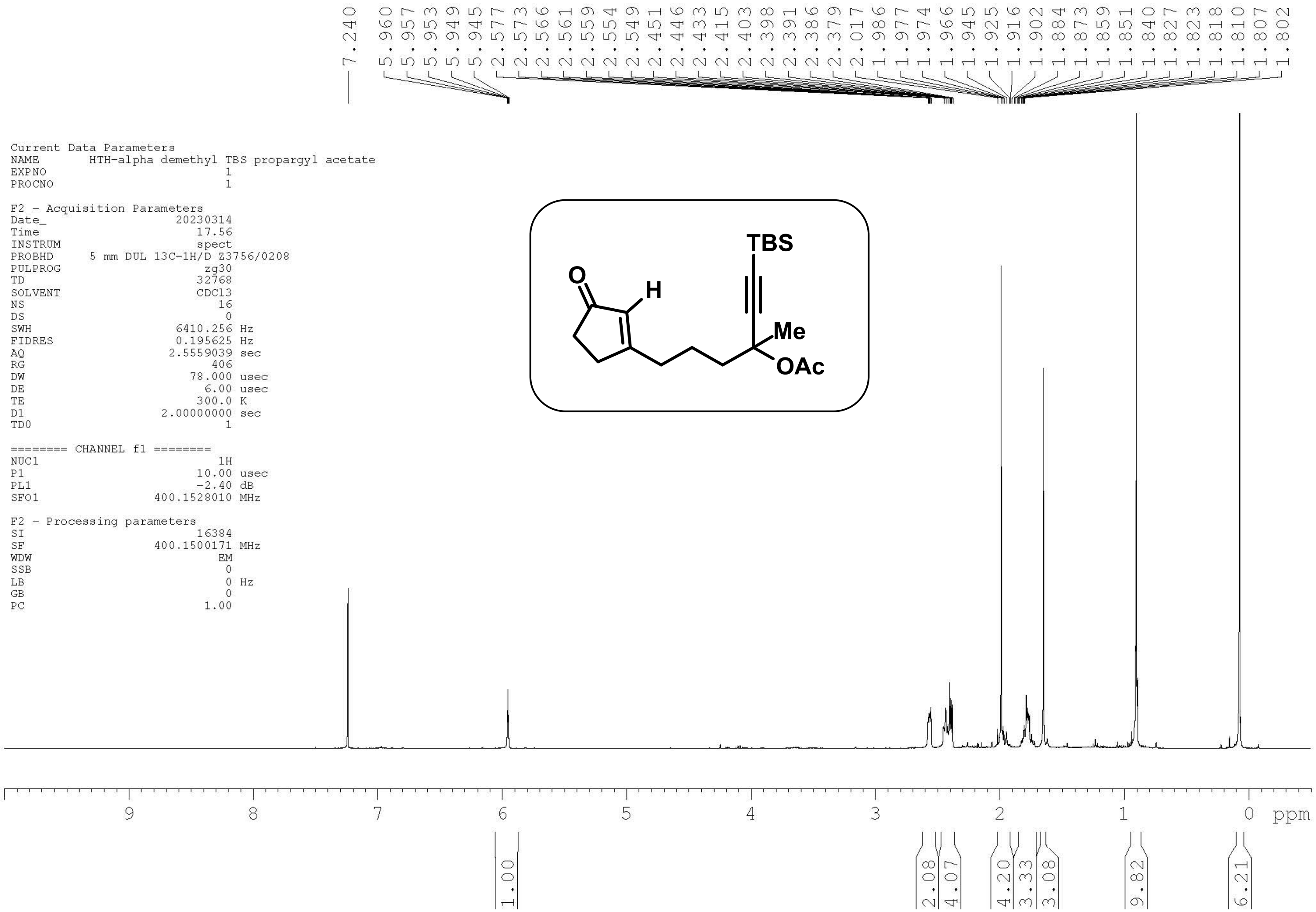

Figure 17. ^{1}H NMR (400 MHz, $CDCl_3$) (**25e**)

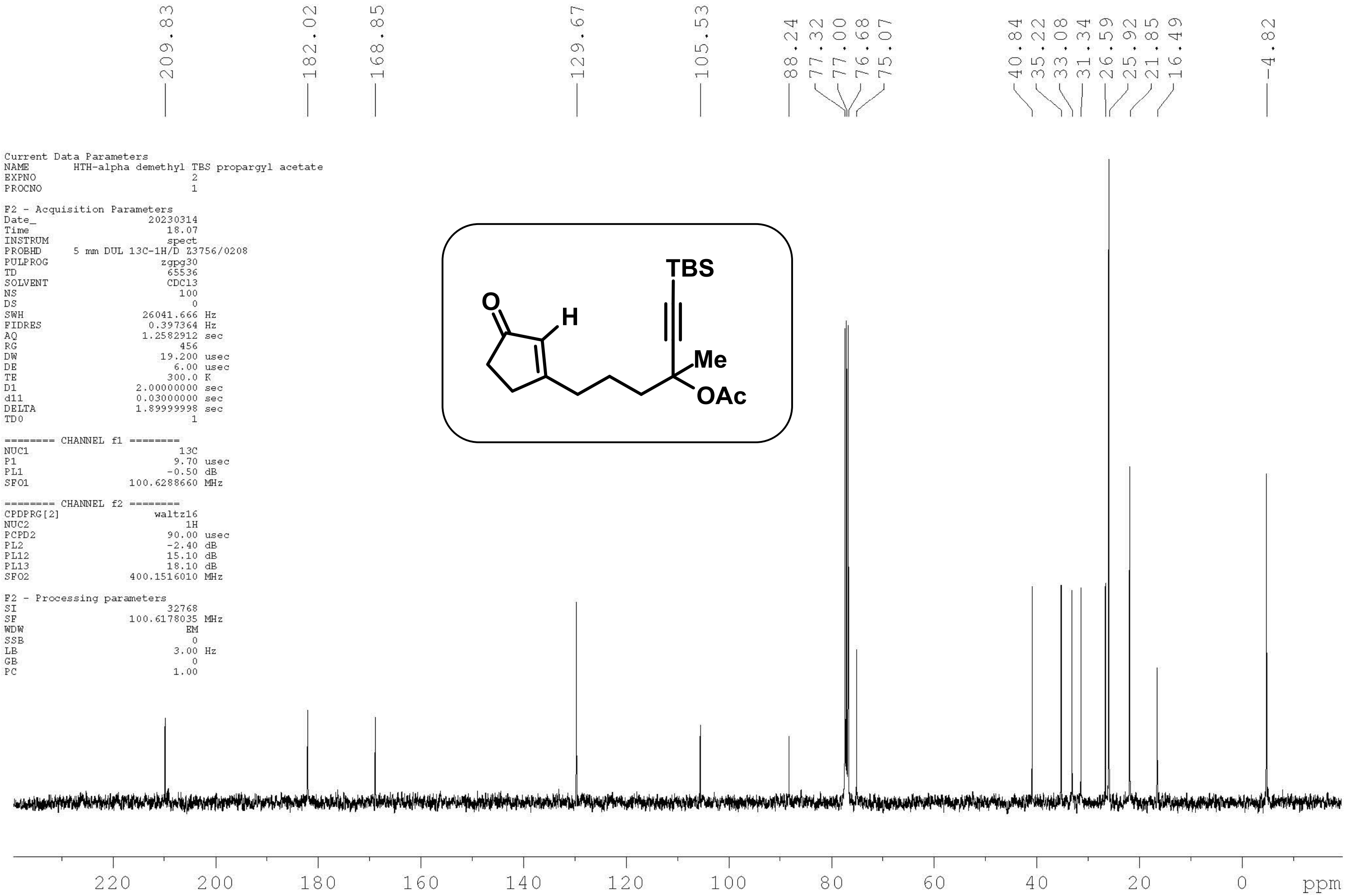

Figure 18. ^{13}C NMR (100 MHz, $CDCl_3$) (**25e**)

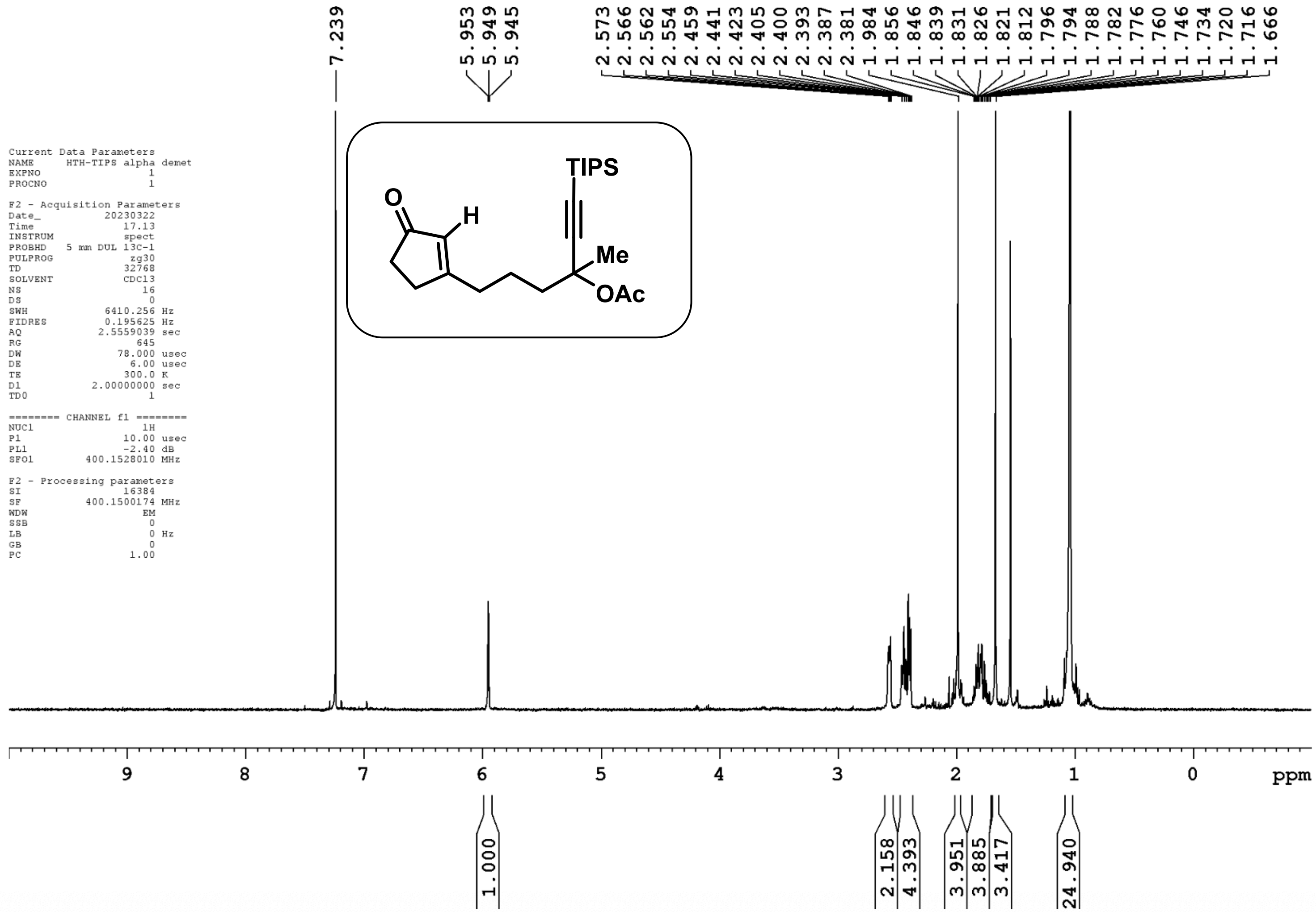

Figure 19. ^{1}H NMR (400 MHz, $CDCl_3$) (**25f**)

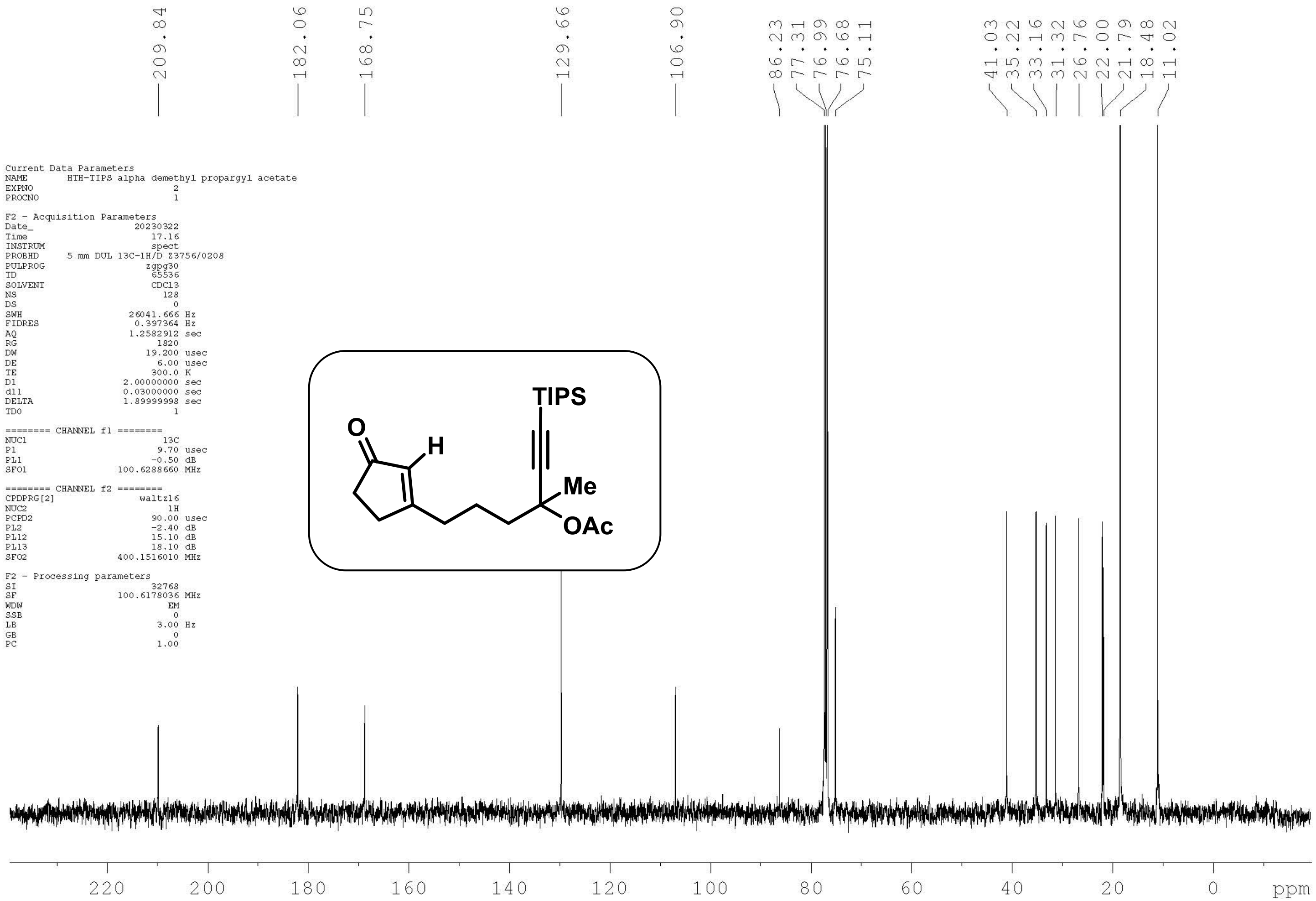

Figure 20. ^{13}C NMR (100 MHz, $CDCl_3$) (**25f**)

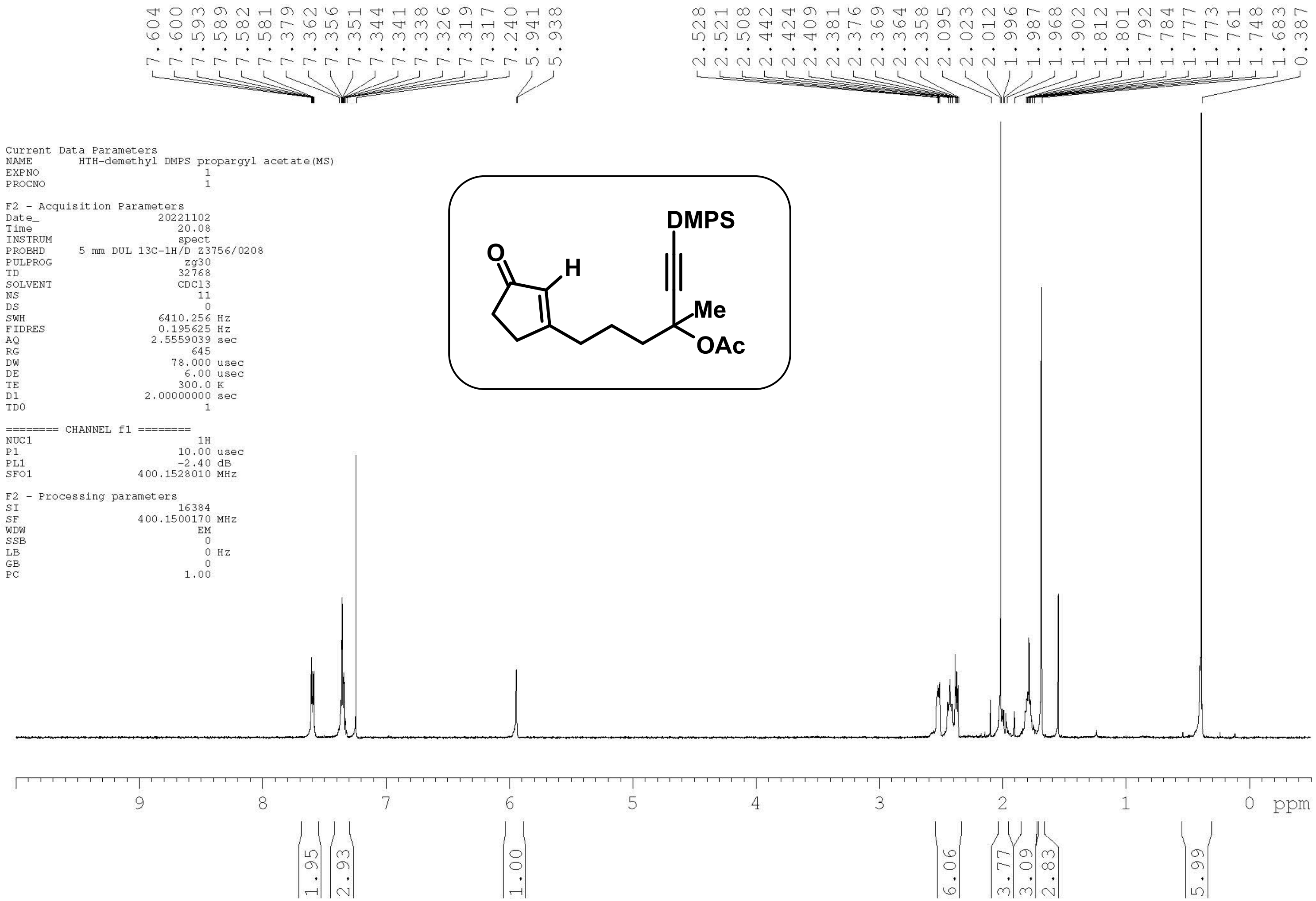

Figure 21. ^{1}H NMR (400 MHz, $CDCl_3$) (**25g**)

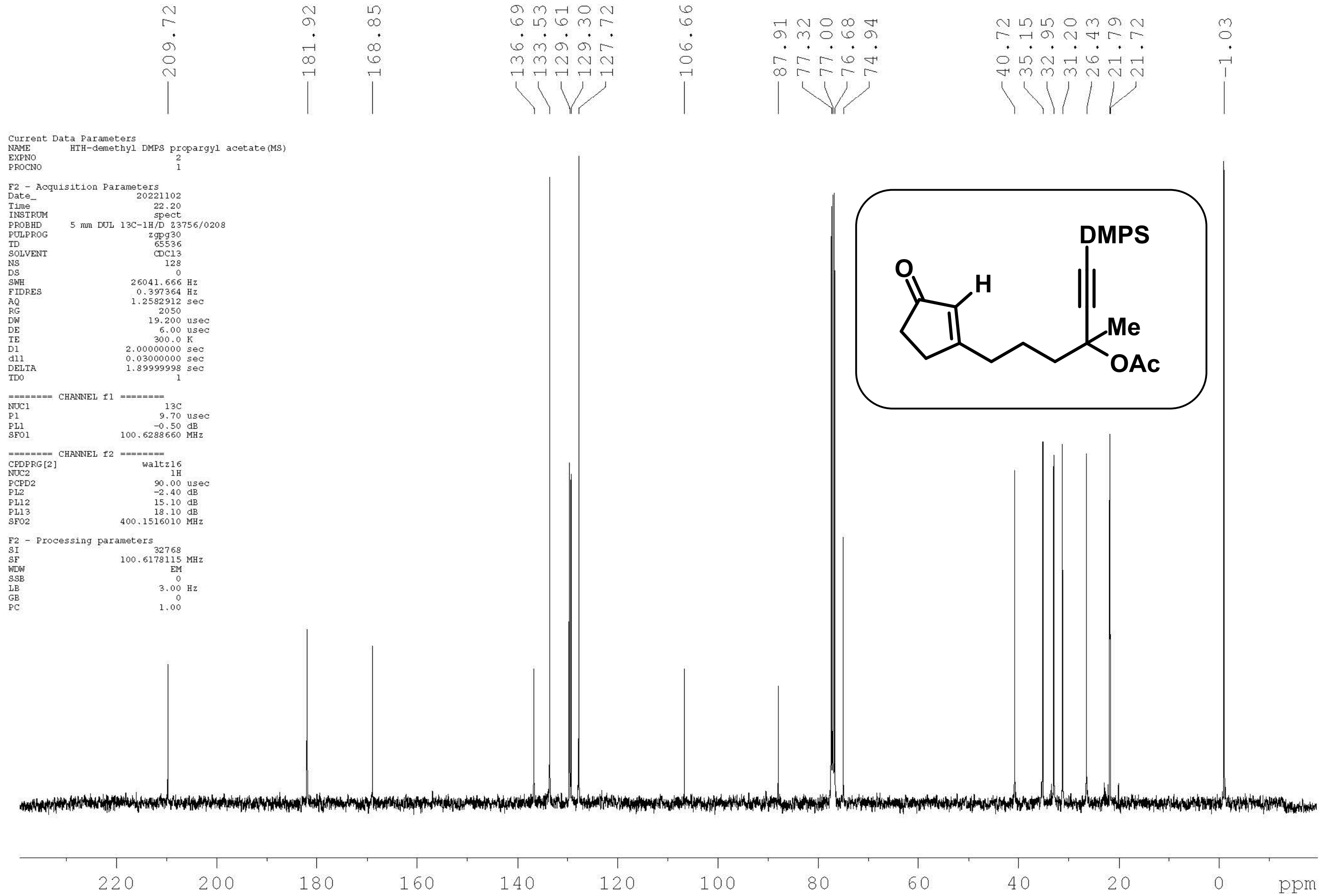

Figure 22. ^{13}C NMR (100 MHz, $CDCl_3$) (**25g**)

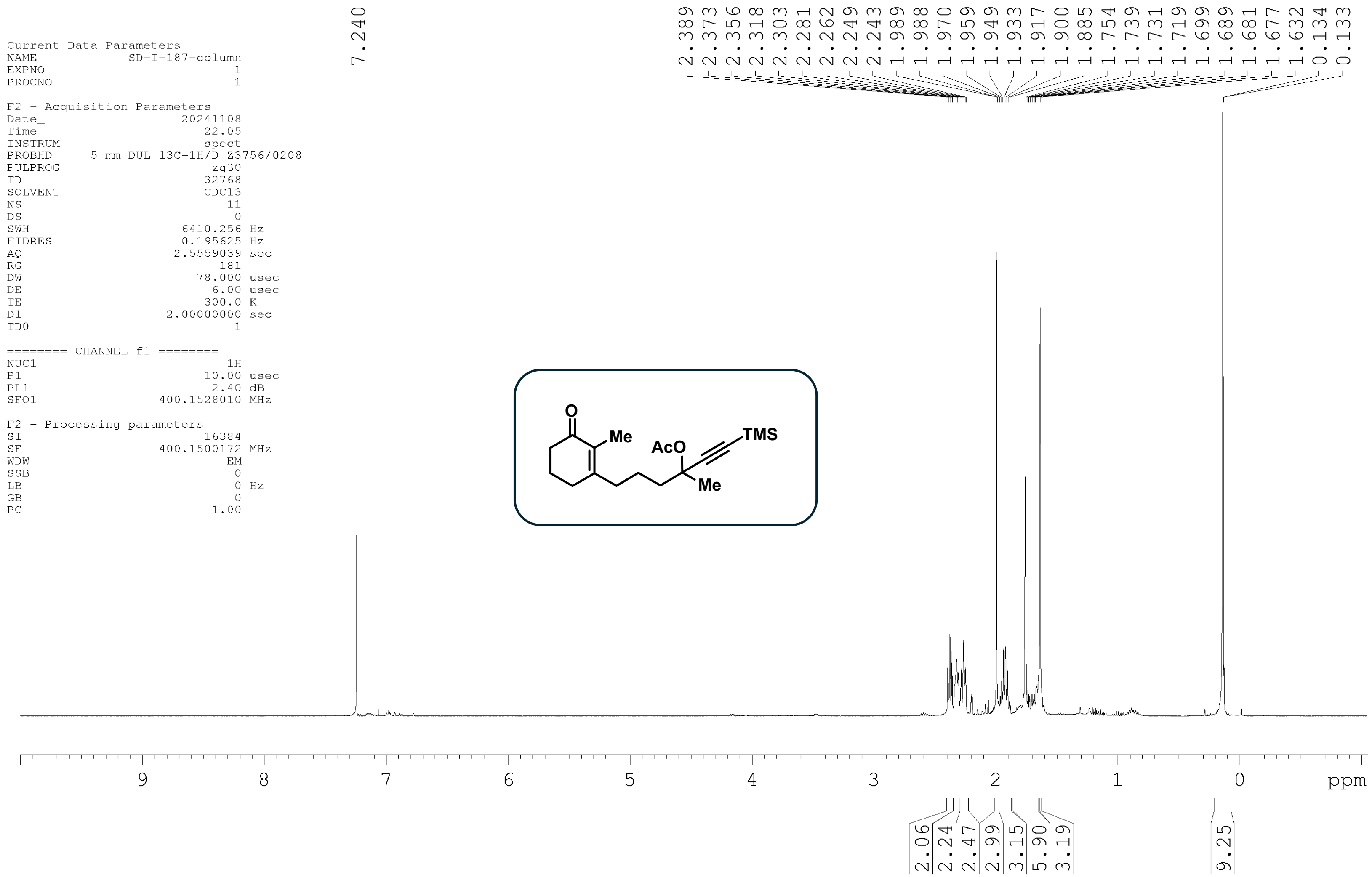

Figure 23. ^{1}H NMR (400 MHz, $CDCl_3$) (**25h**)

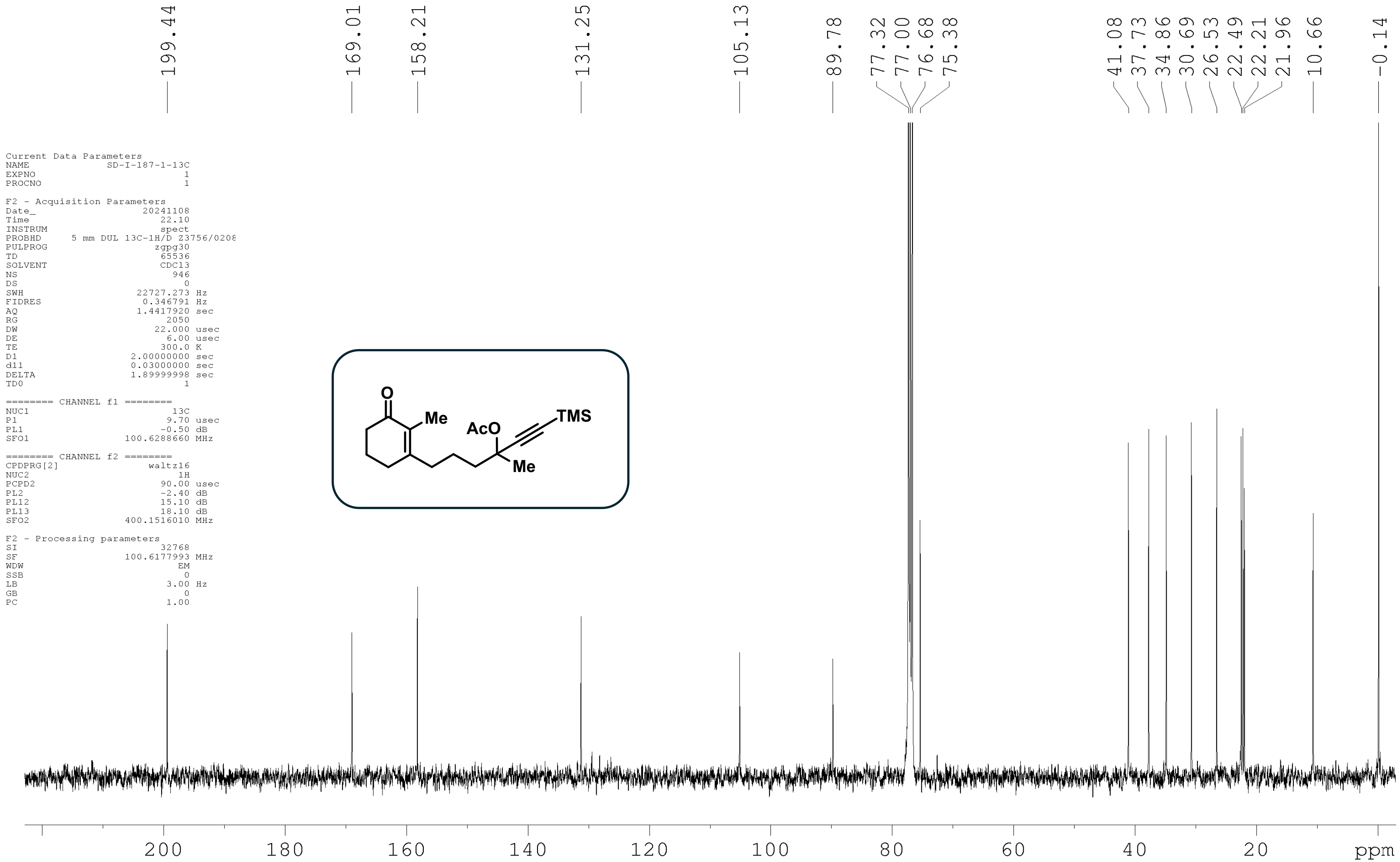

Figure 24. ^{13}C NMR (100 MHz, CDCl$_3$) (**25h**)

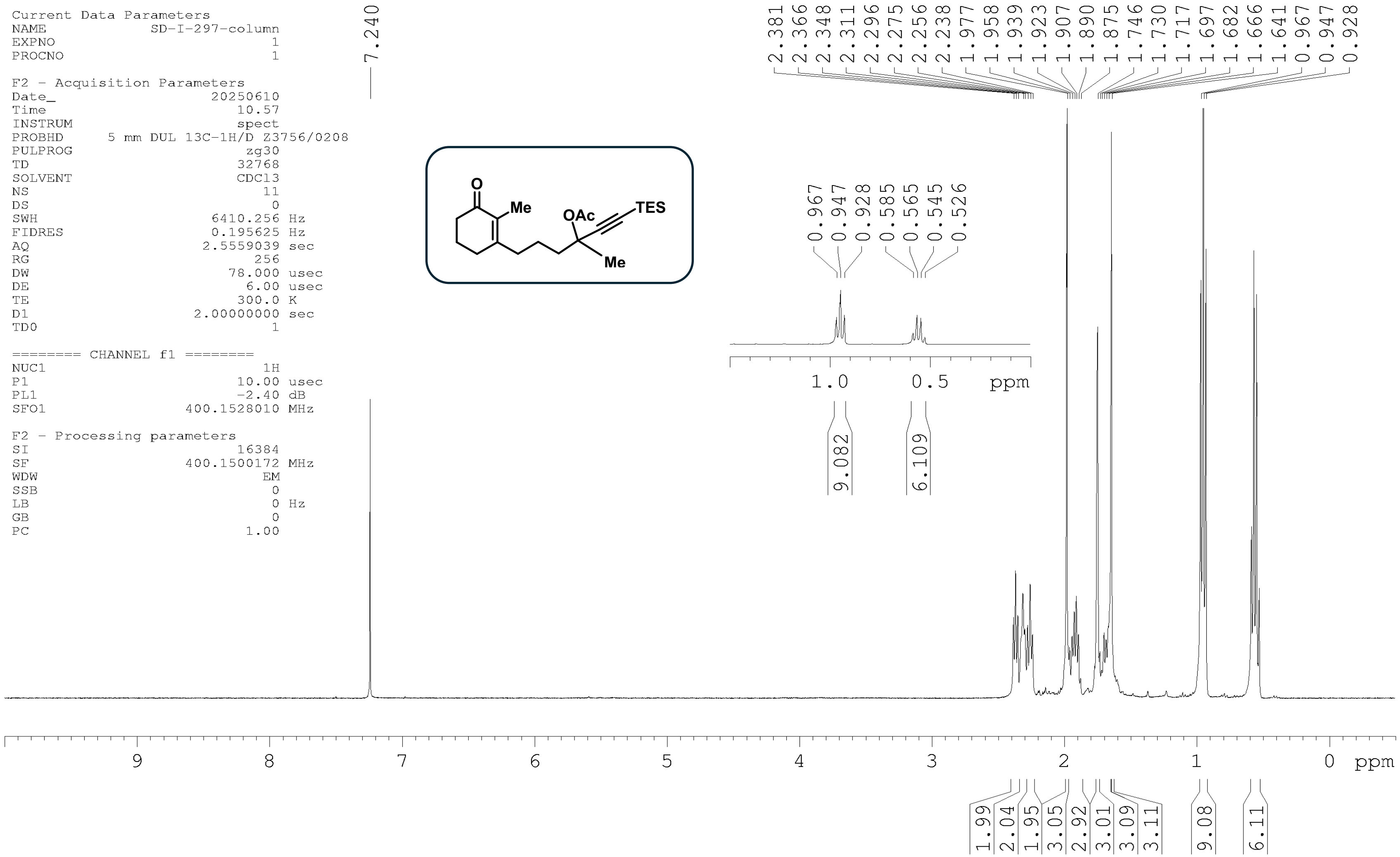

Figure 25. ^{1}H NMR (400 MHz, $CDCl_3$) (**25i**)

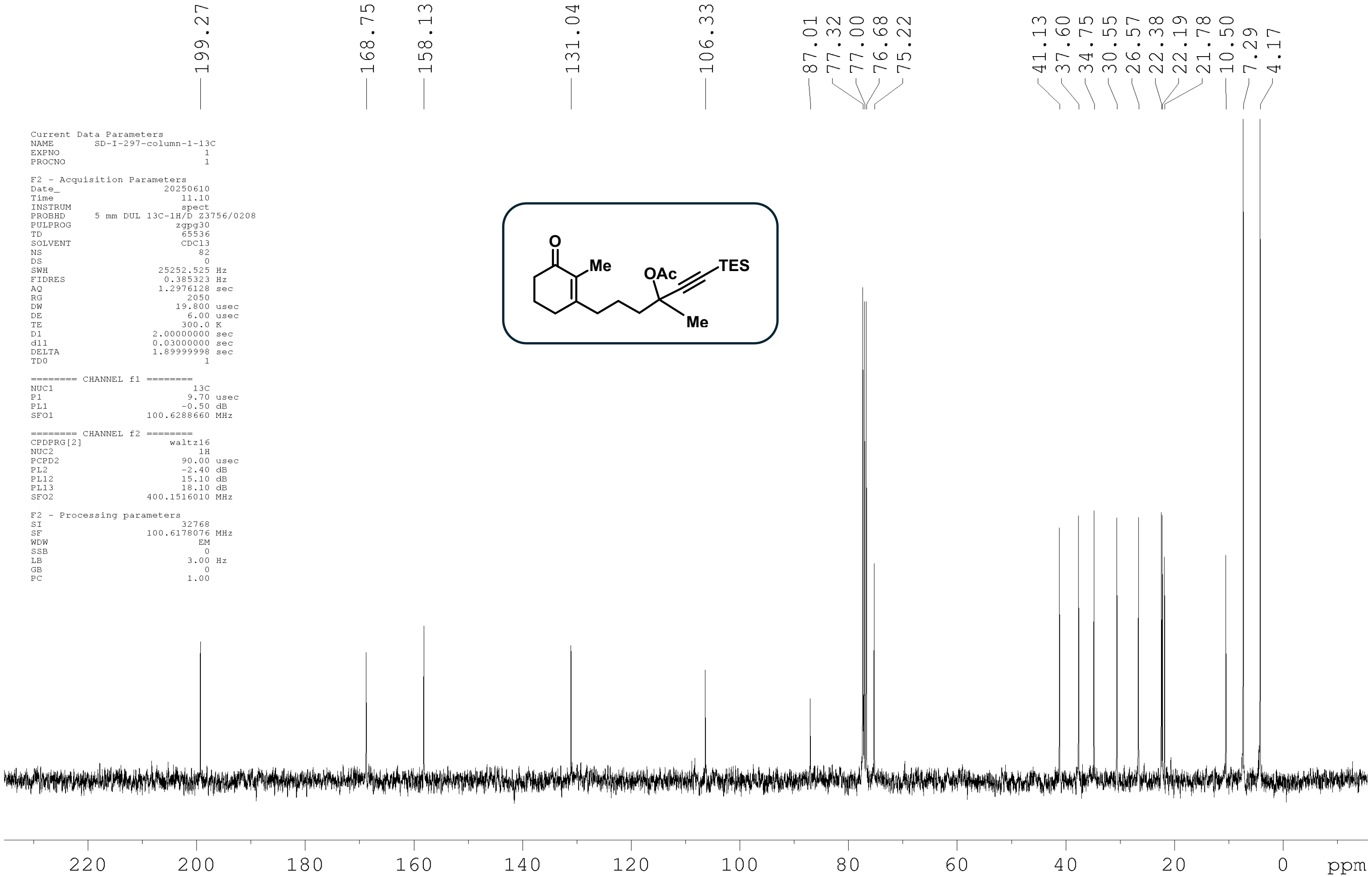

Figure 26. ^{13}C NMR (100 MHz, $CDCl_3$) (**25i**)

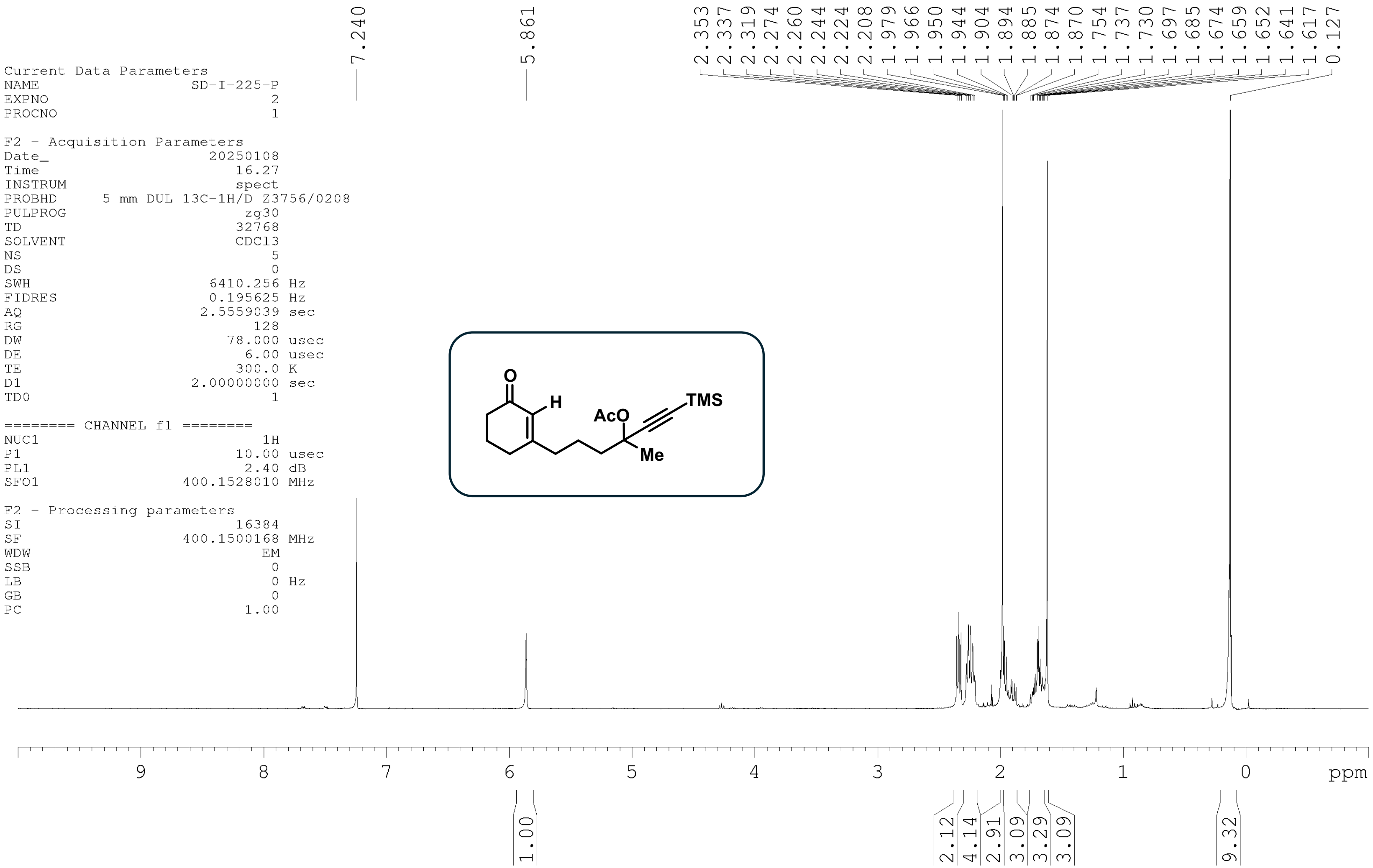

Figure 27. ^{1}H NMR (400 MHz, $CDCl_3$) (**25j**)

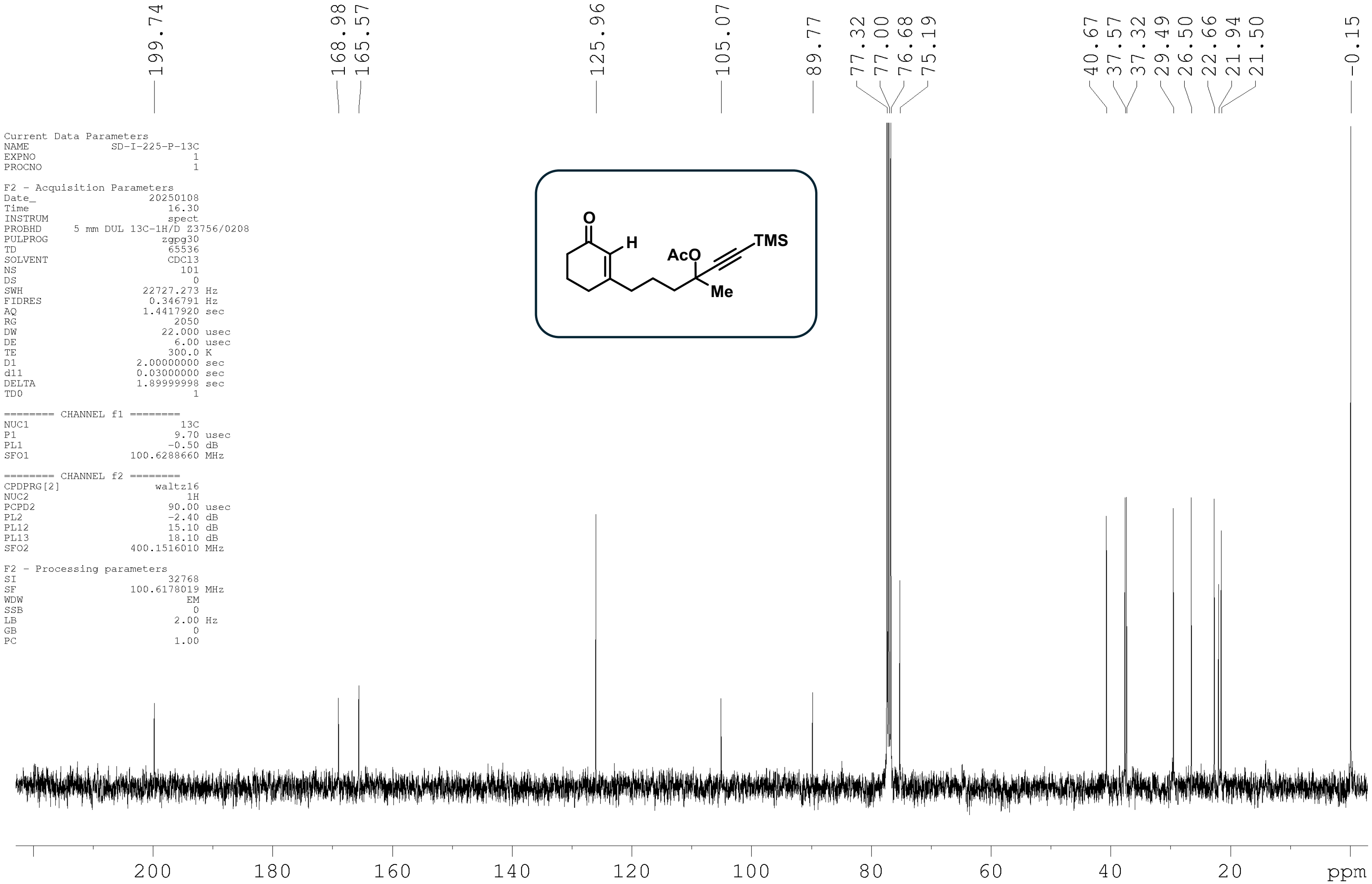

Figure 28. ^{13}C NMR (100 MHz, $CDCl_3$) (**25j**)

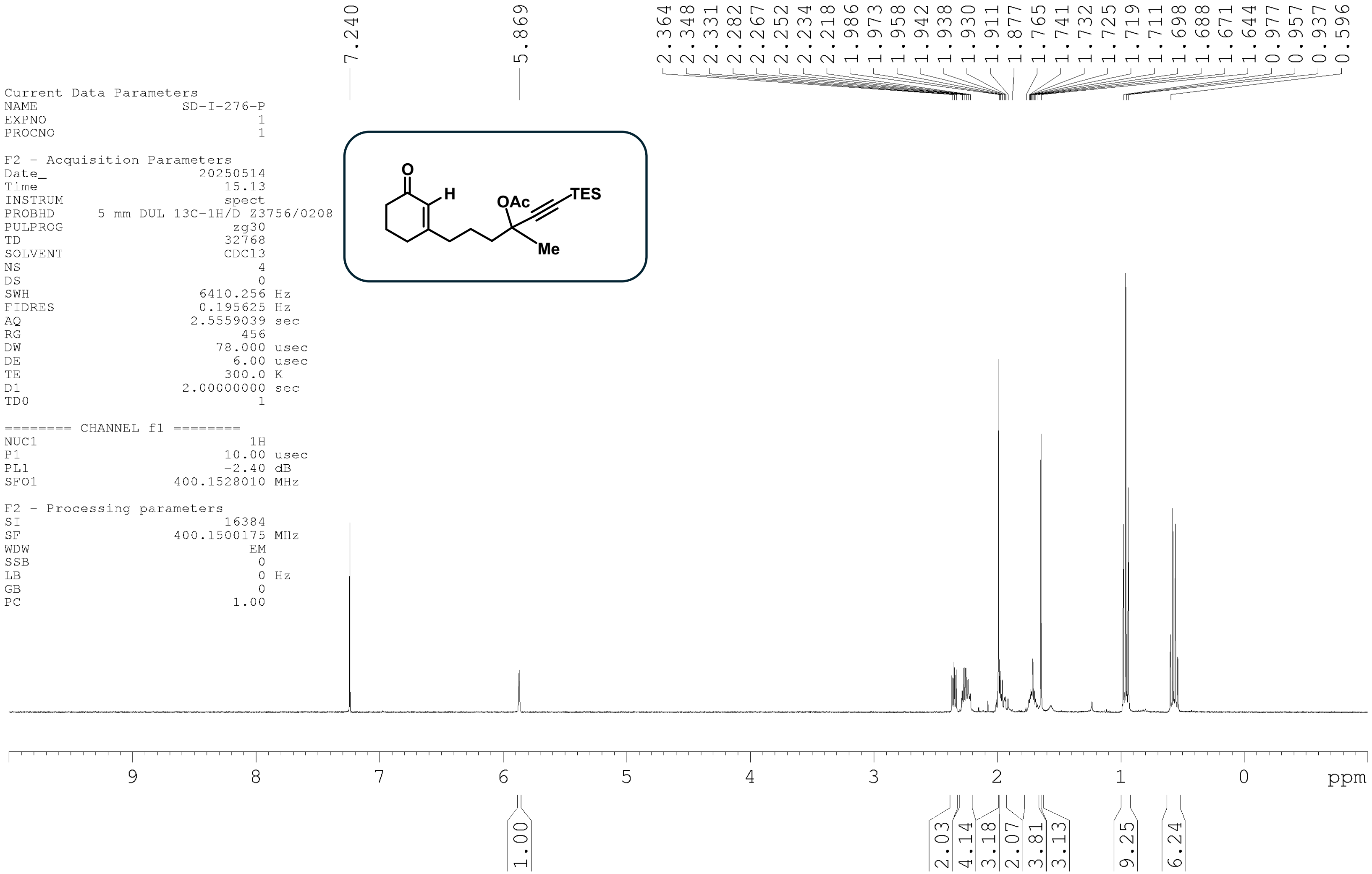

Figure 29. ^{1}H NMR (400 MHz, $CDCl_3$) (**25k**)

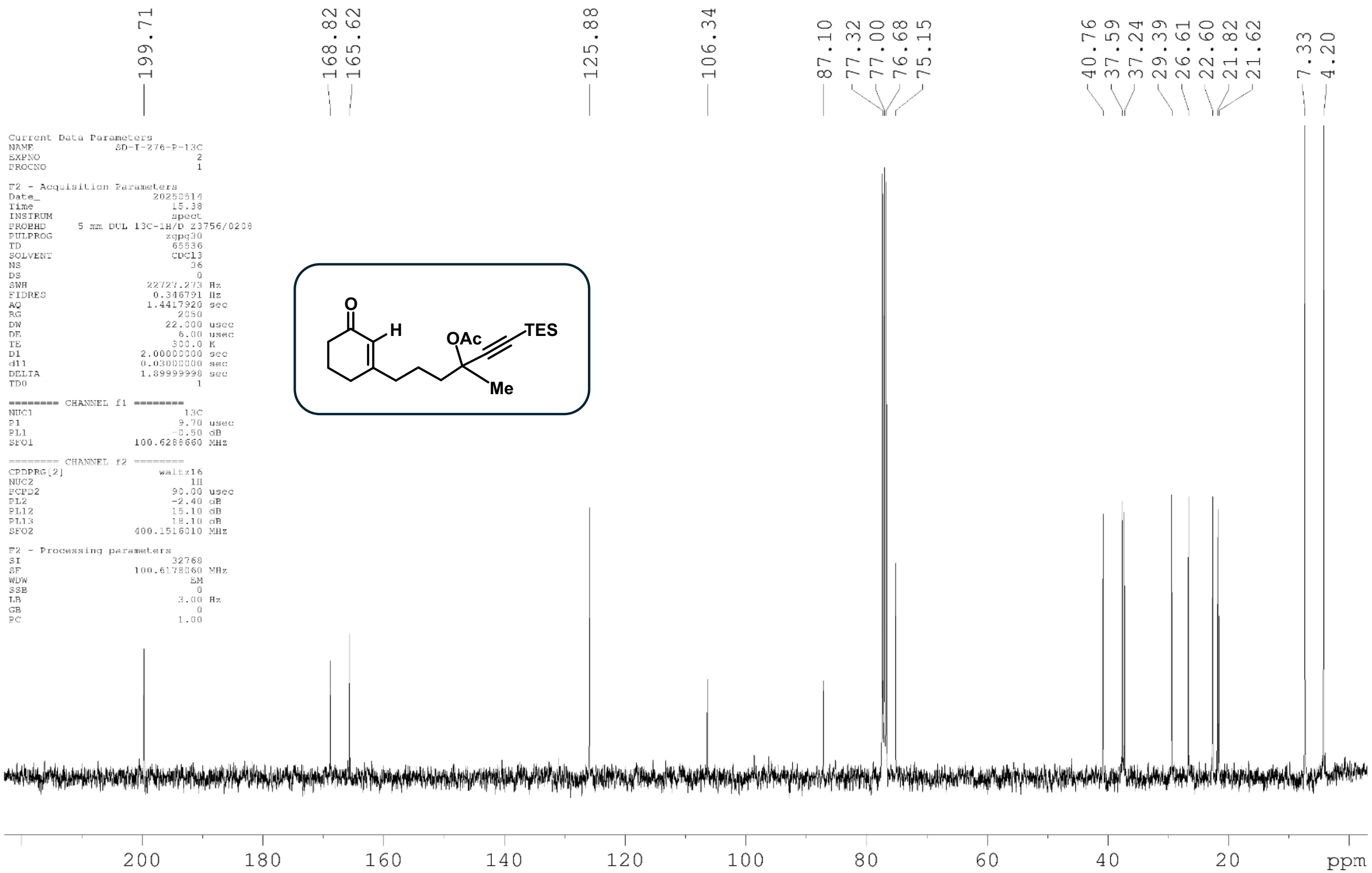

Figure 30. ^{13}C NMR (100 MHz, $CDCl_3$) (**25k**)

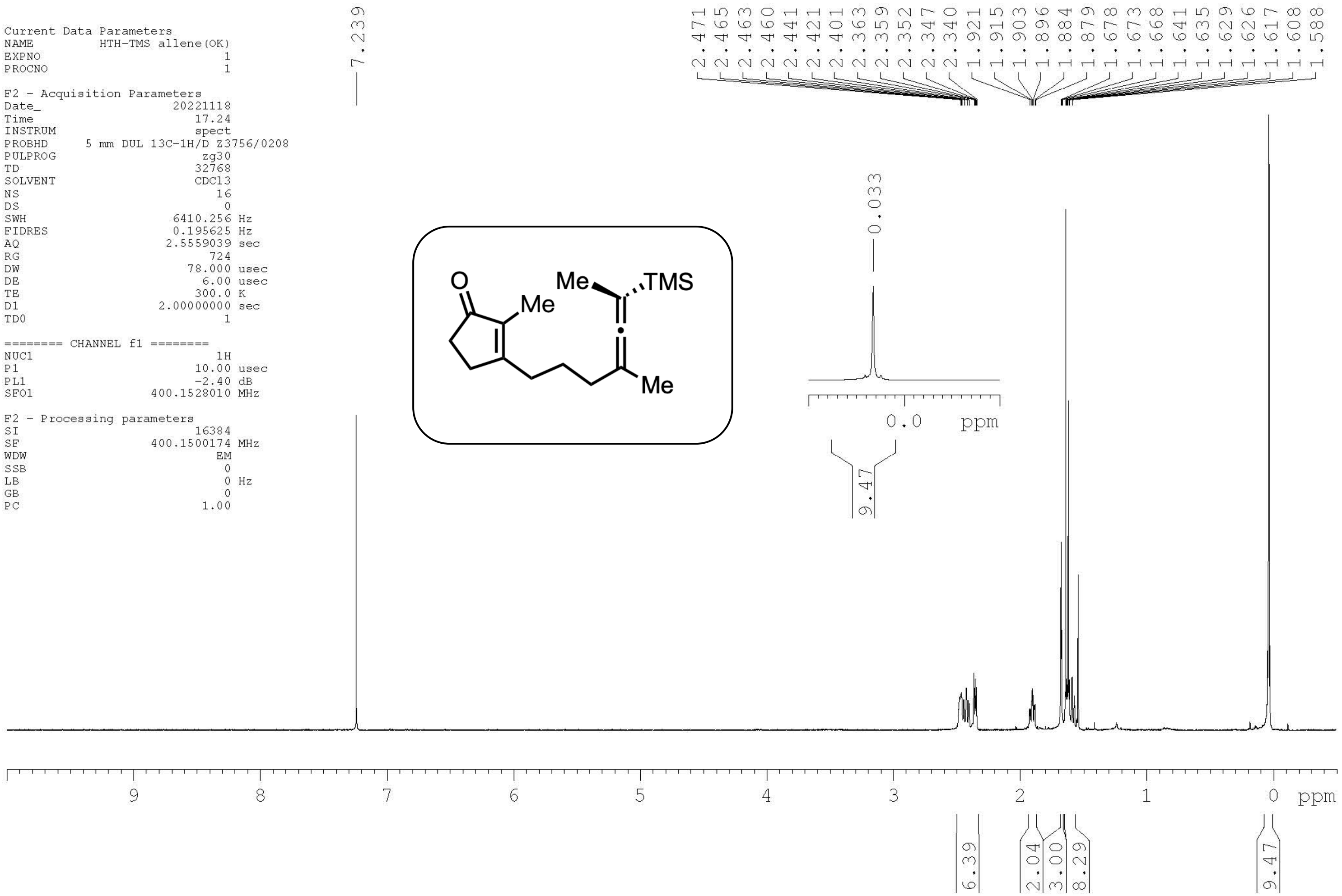

Figure 31. ^{1}H NMR (400 MHz, $CDCl_3$) (**26a**)

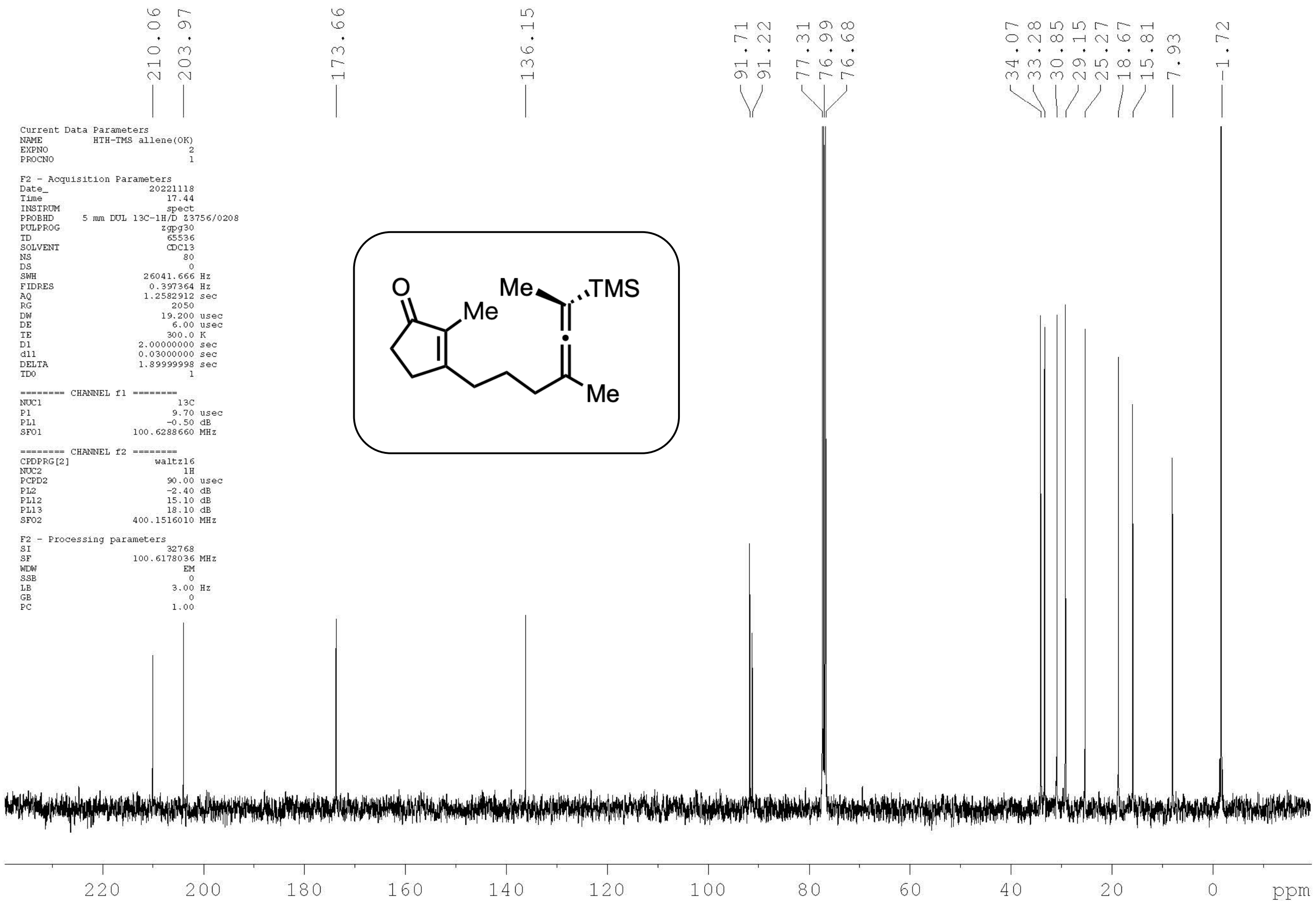

Figure 32. ^{13}C NMR (100 MHz, $CDCl_3$) (**26a**)

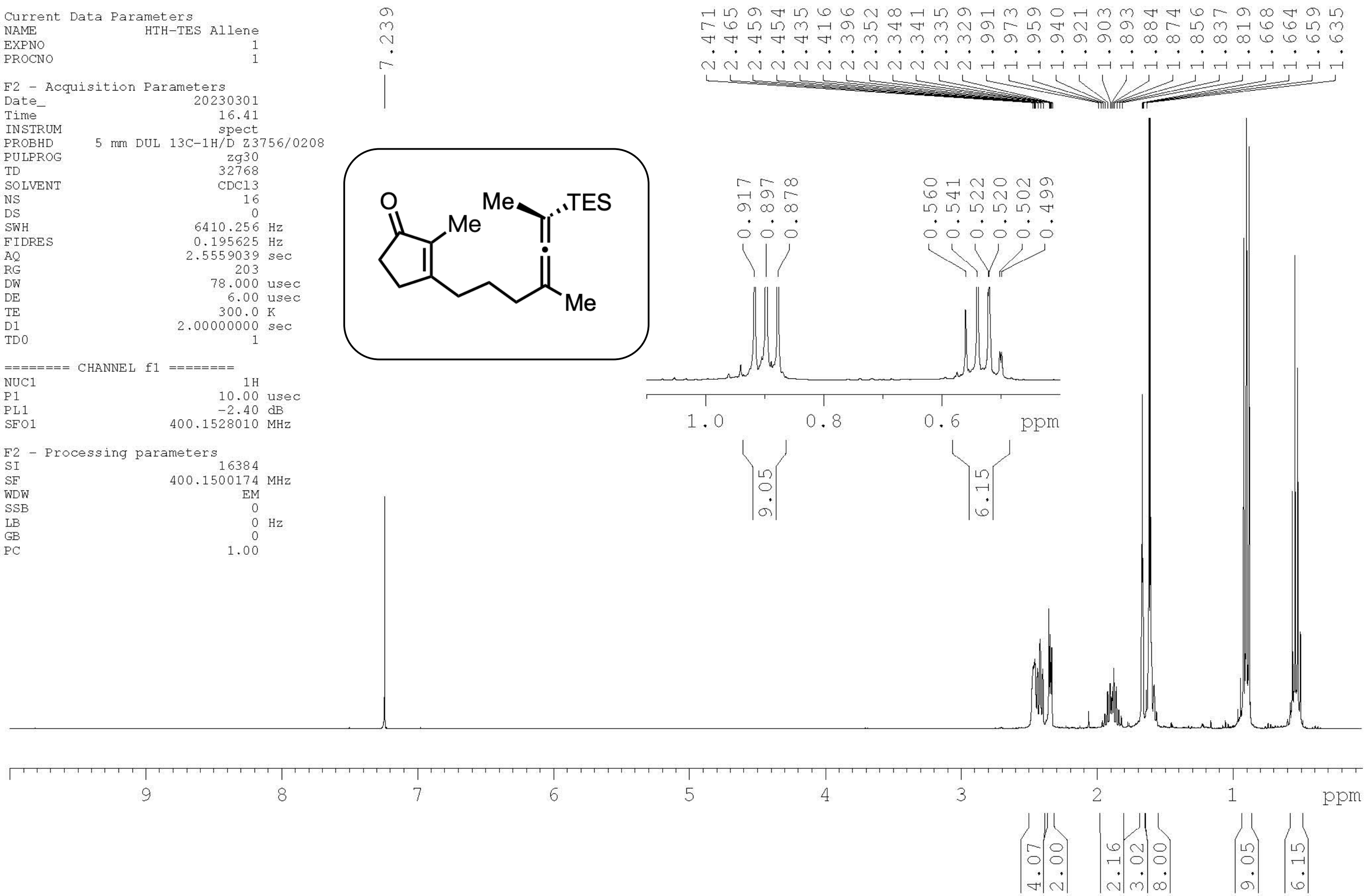

Figure 33. ^{1}H NMR (400 MHz, $CDCl_3$) (**26b**)

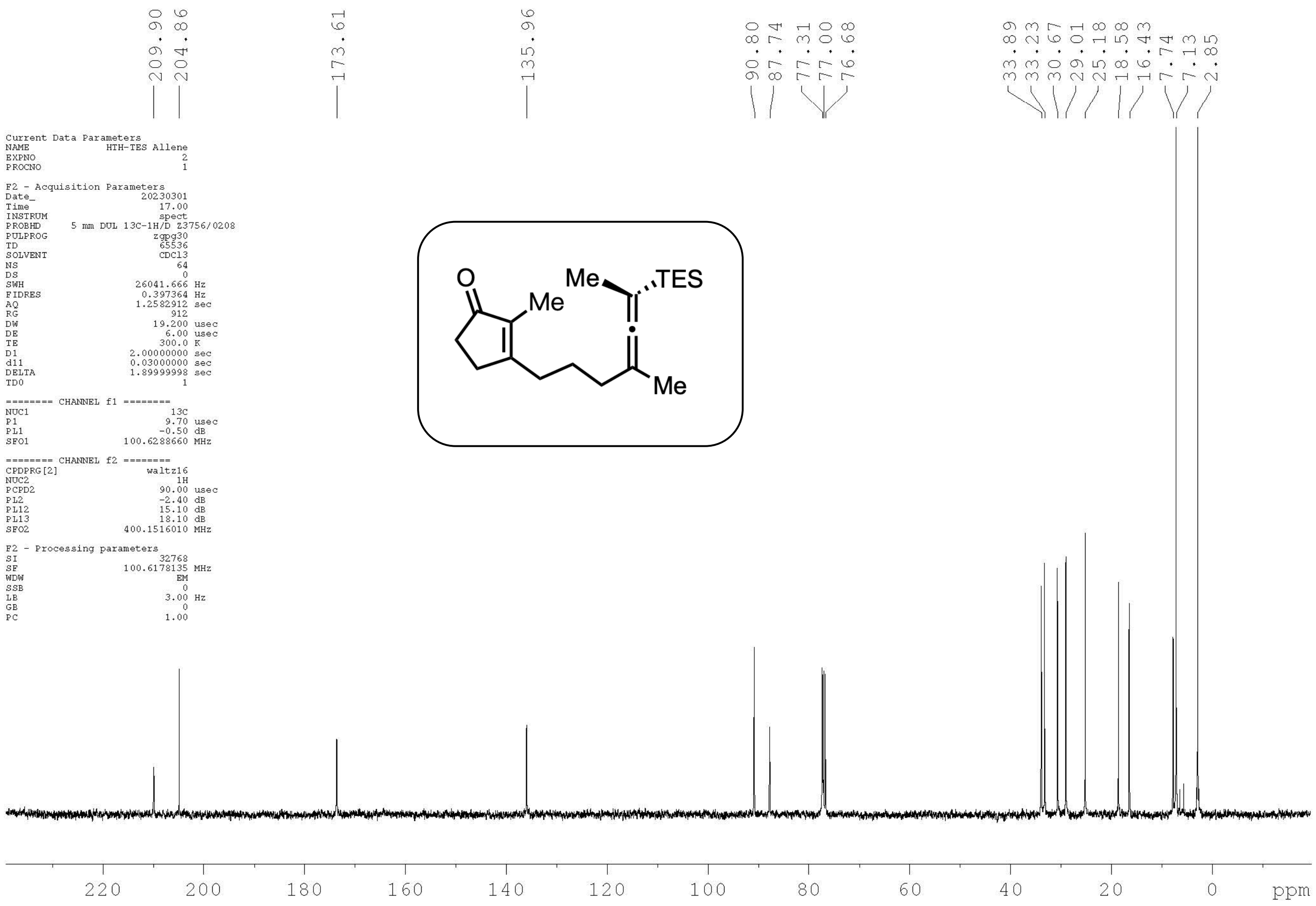

Figure 34. ^{13}C NMR (100 MHz, $CDCl_3$) (**26b**)

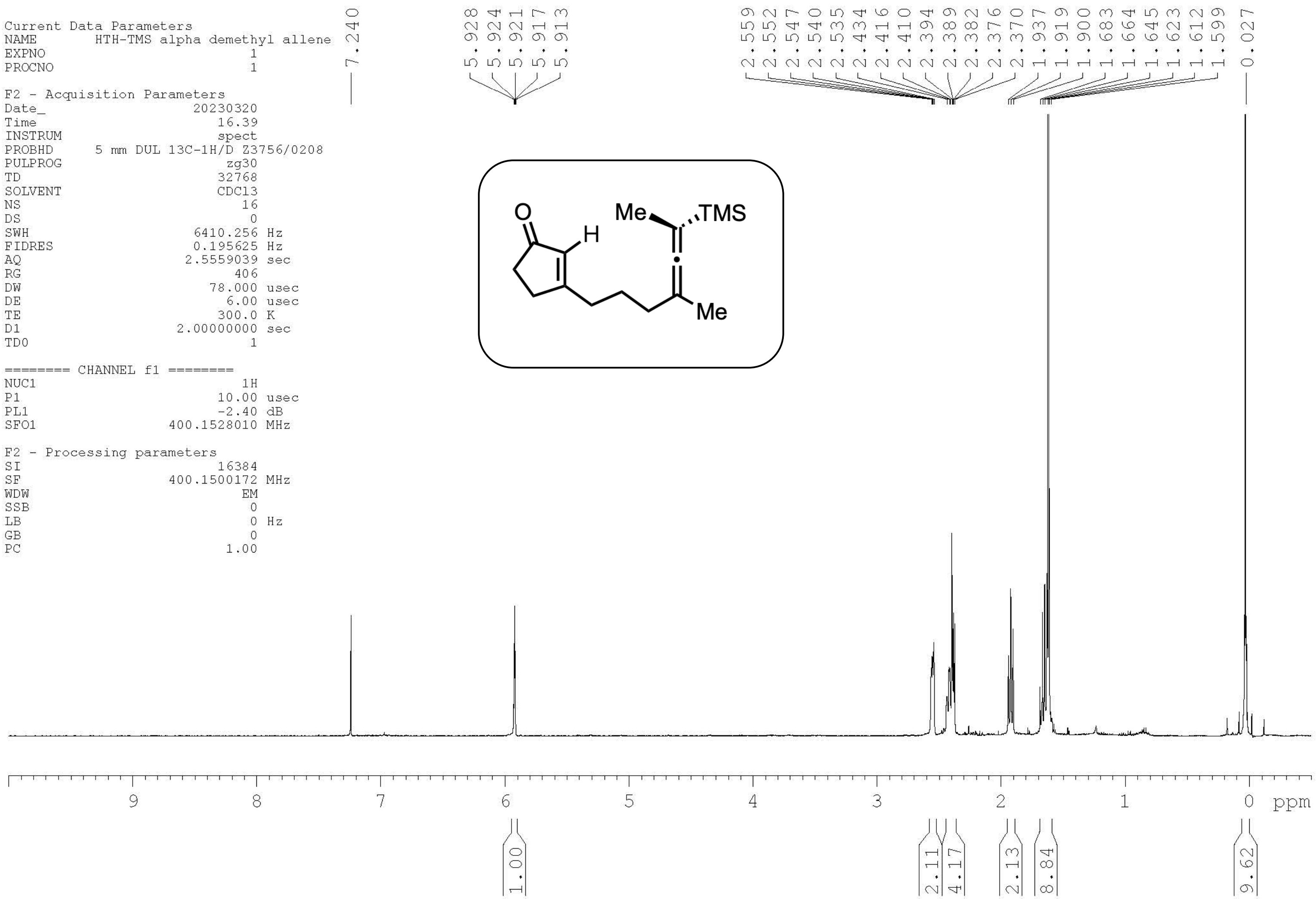

Figure 35. ^{1}H NMR (400 MHz, $CDCl_3$) (**26c**)

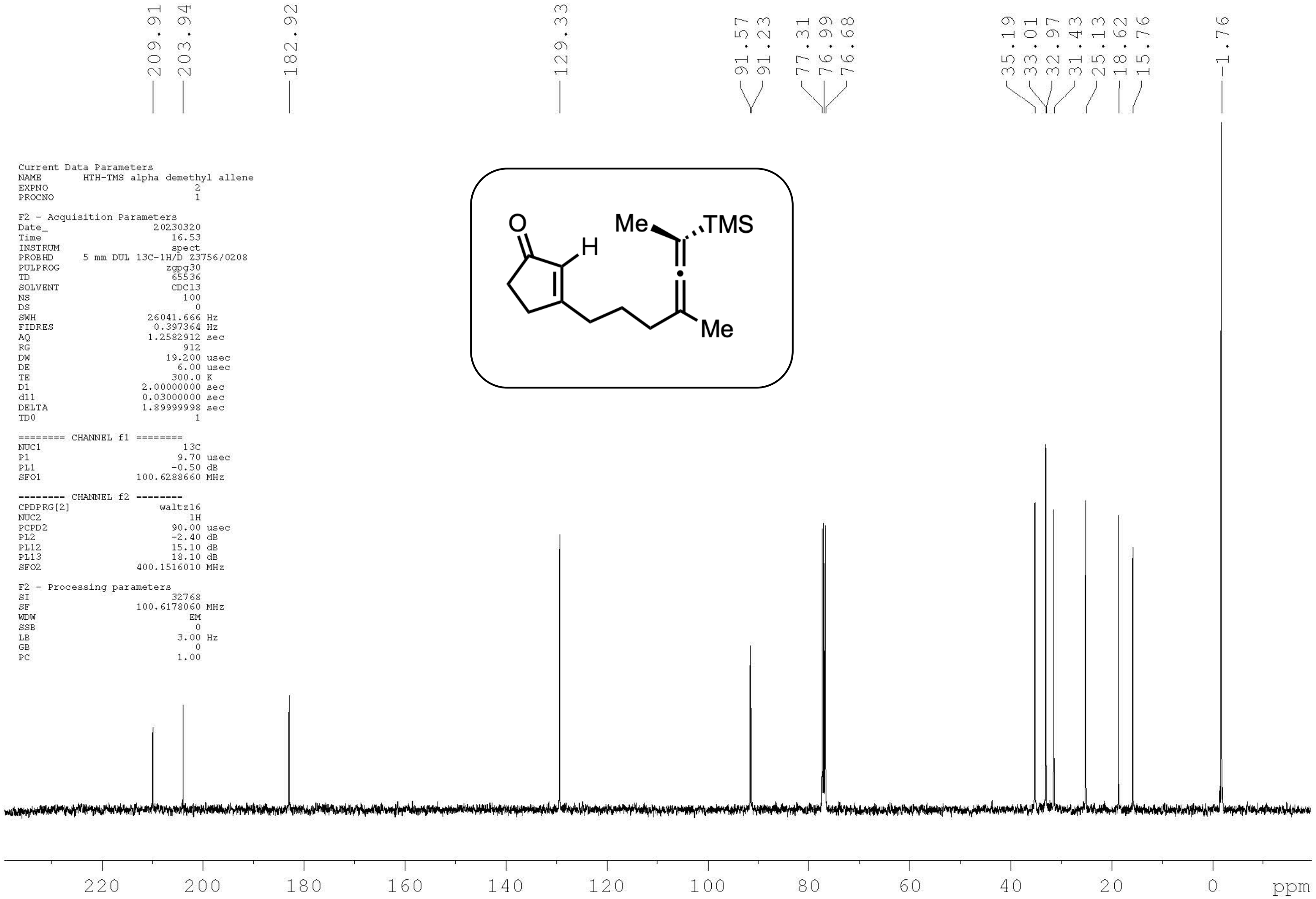

Figure 36. ^{13}C NMR (100 MHz, $CDCl_3$) (**26c**)

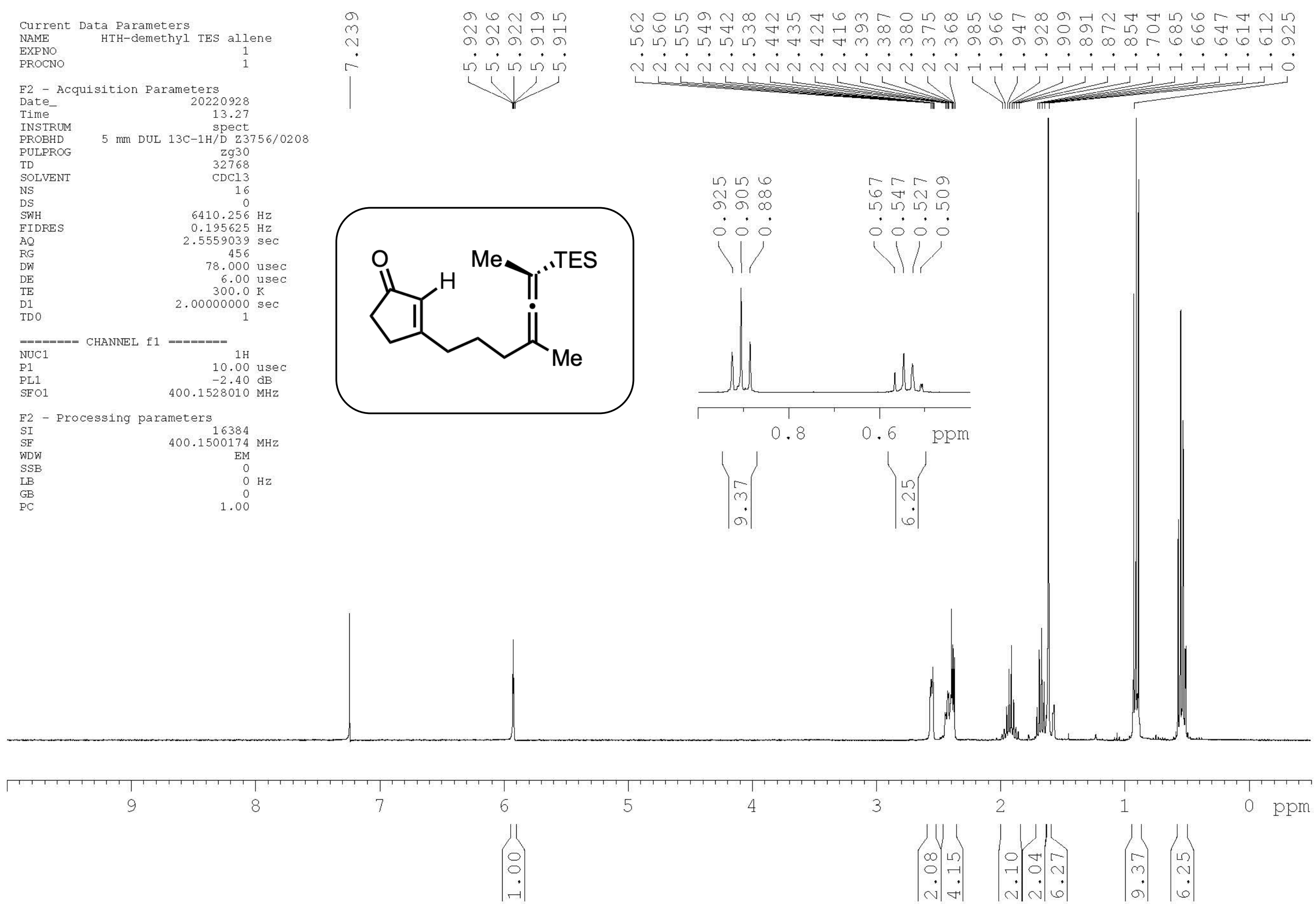

Figure 37. ^{1}H NMR (400 MHz, $CDCl_3$) (**26d**)

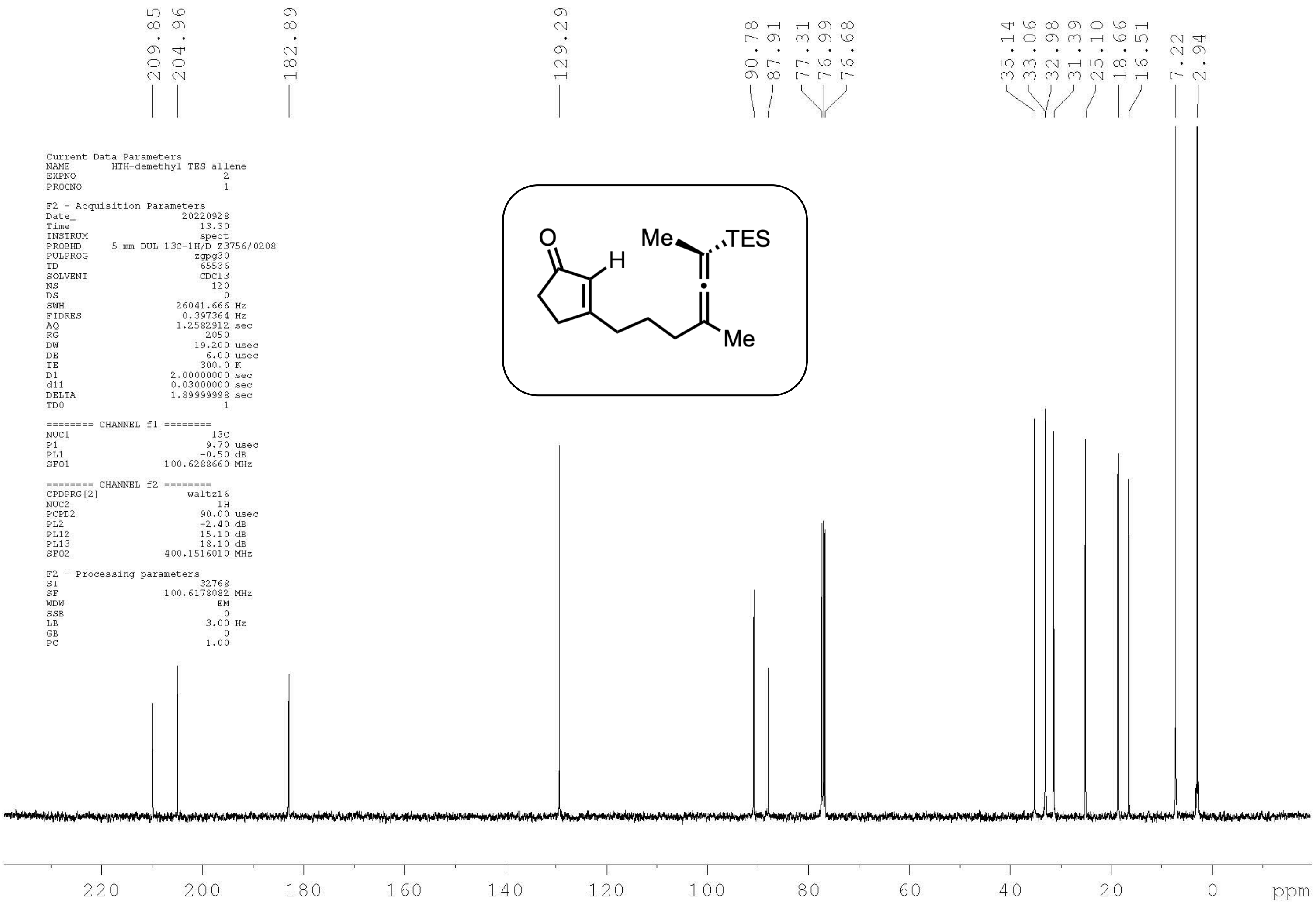

Figure 38. ^{13}C NMR (100 MHz, $CDCl_3$) (**26d**)

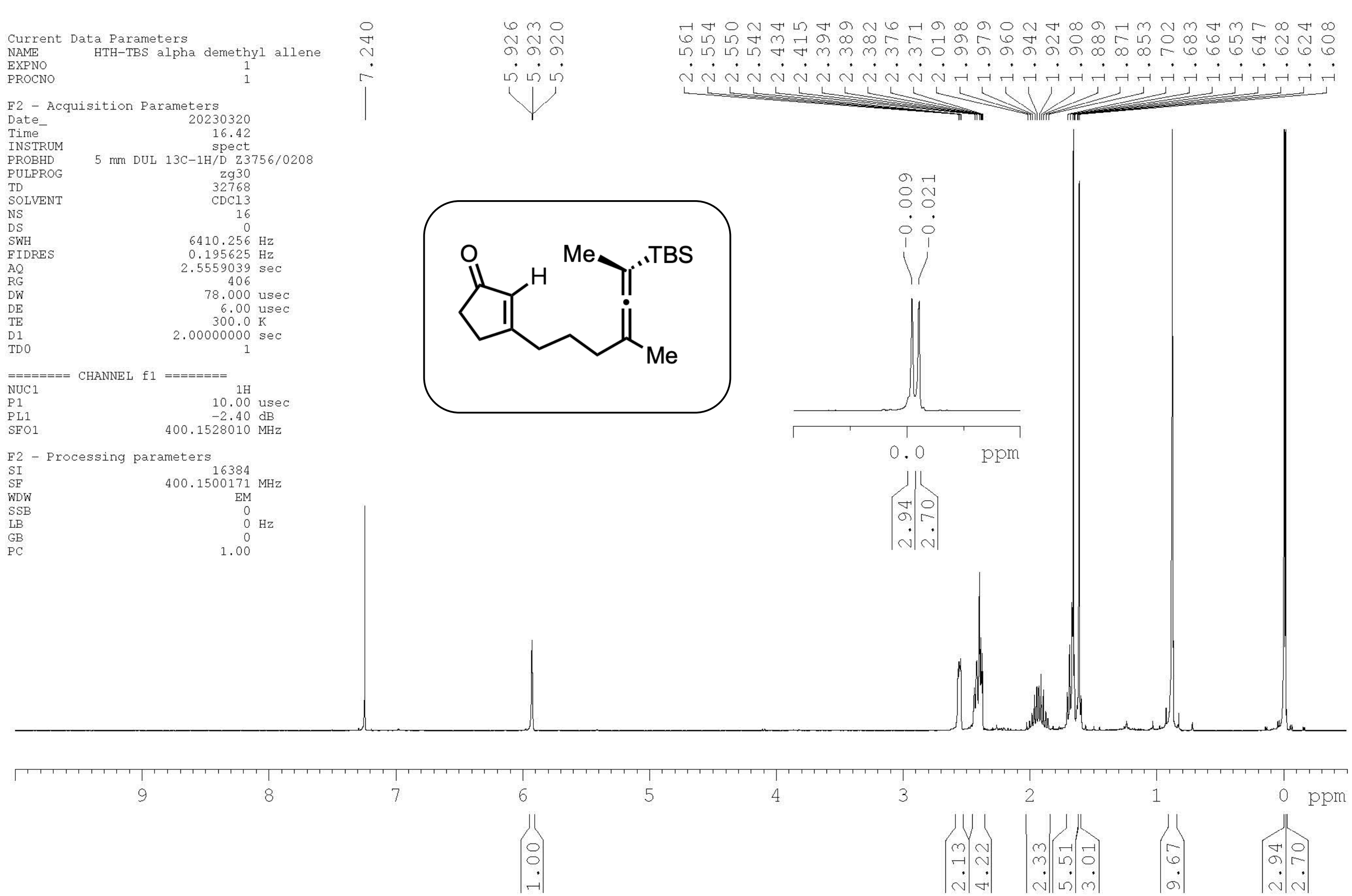

Figure 39. ^{1}H NMR (400 MHz, $CDCl_3$) (**26e**)

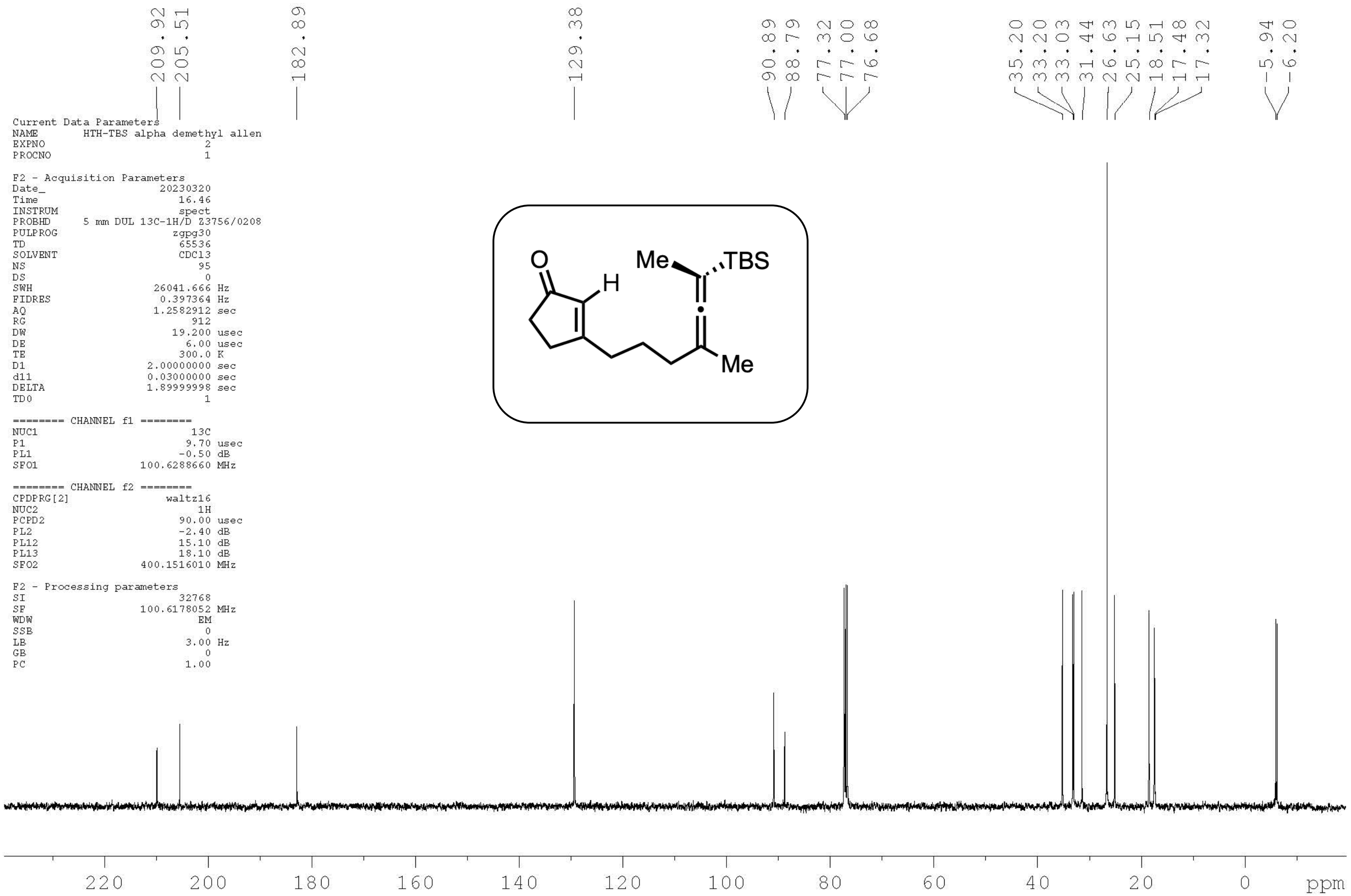

Figure 40. ^{13}C NMR (100 MHz, CDCl$_3$) (**26e**)

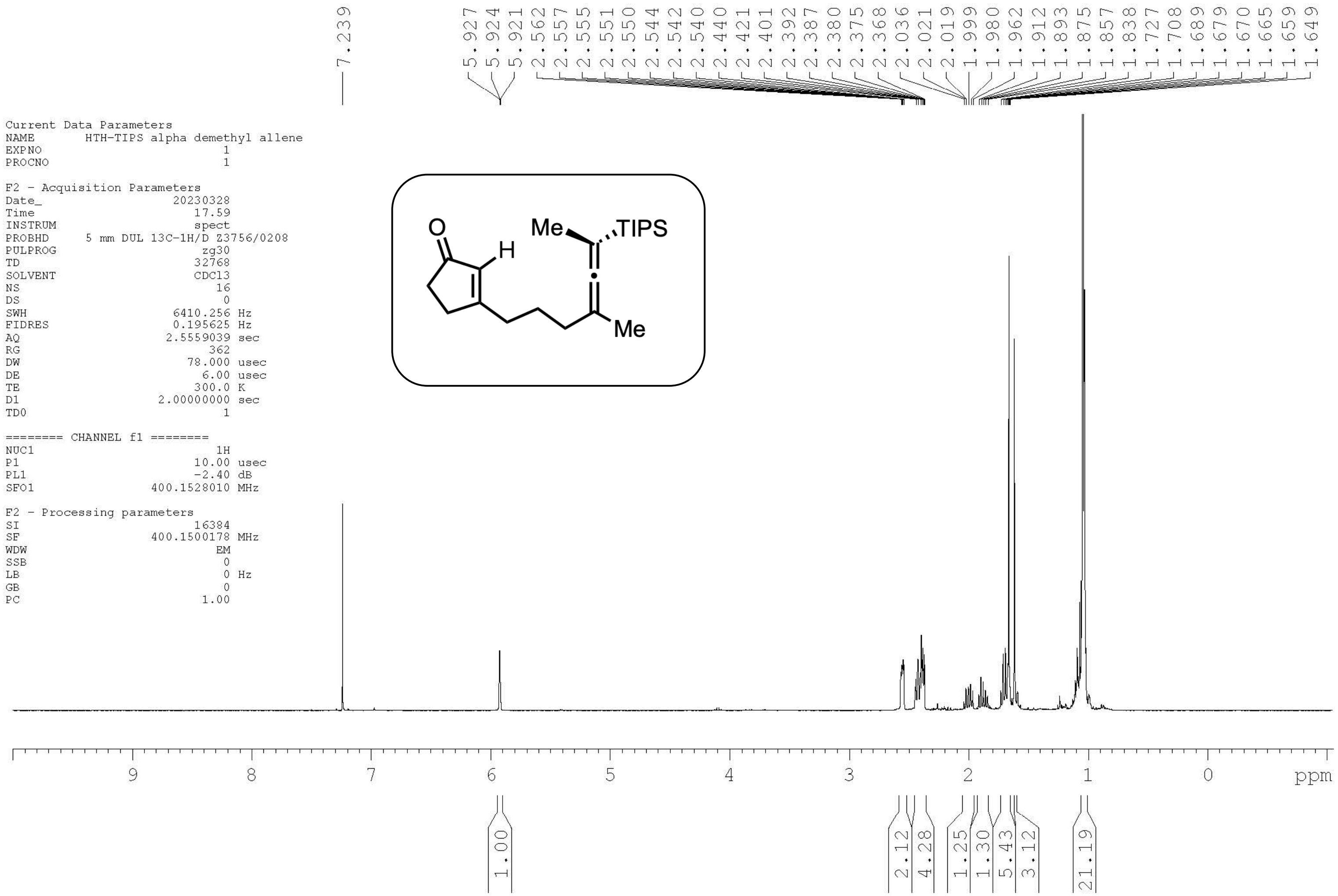

Figure 41. ^{1}H NMR (400 MHz, $CDCl_3$) (**26f**)

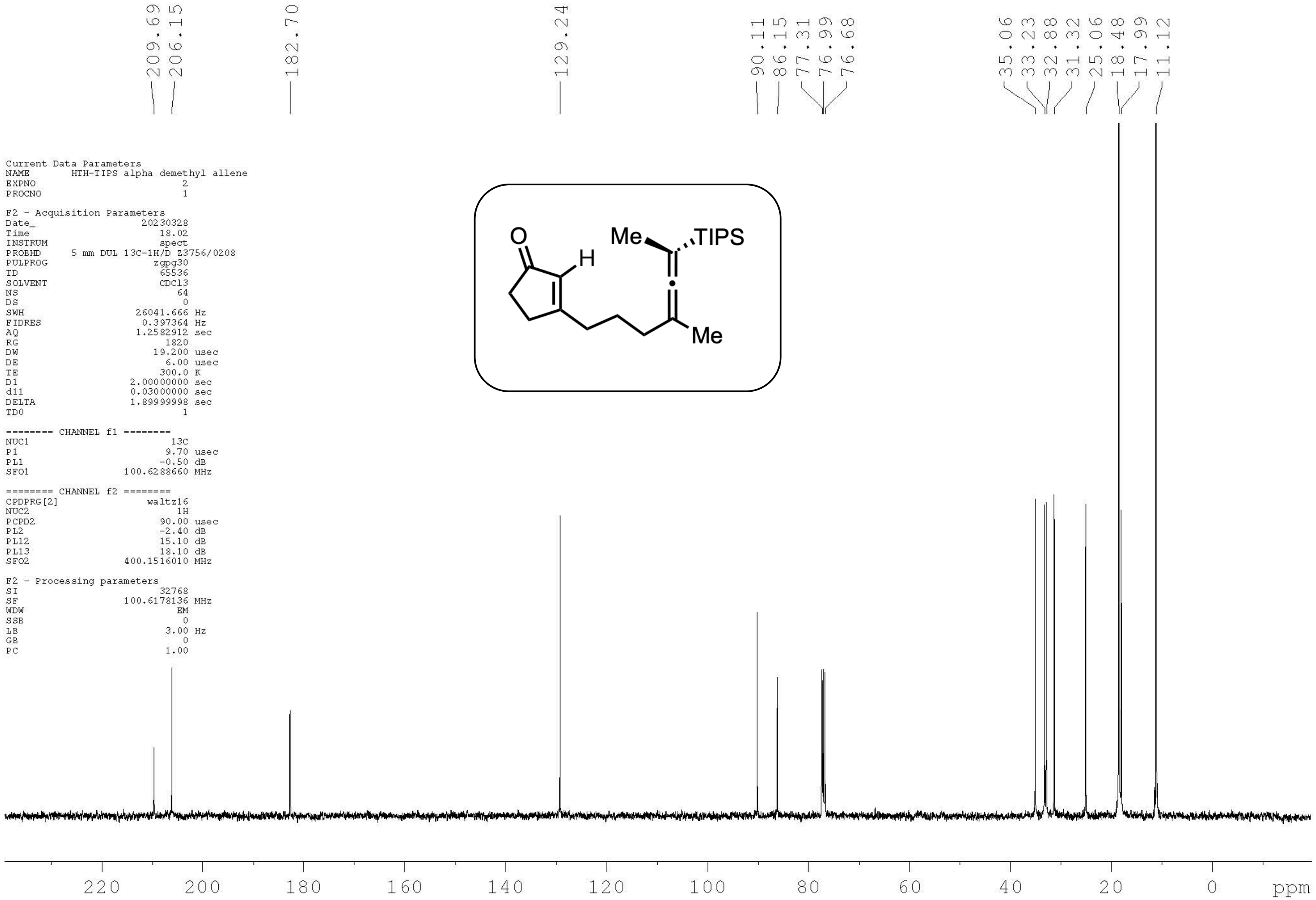

Figure 42. ^{13}C NMR (100 MHz, $CDCl_3$) (**26f**)

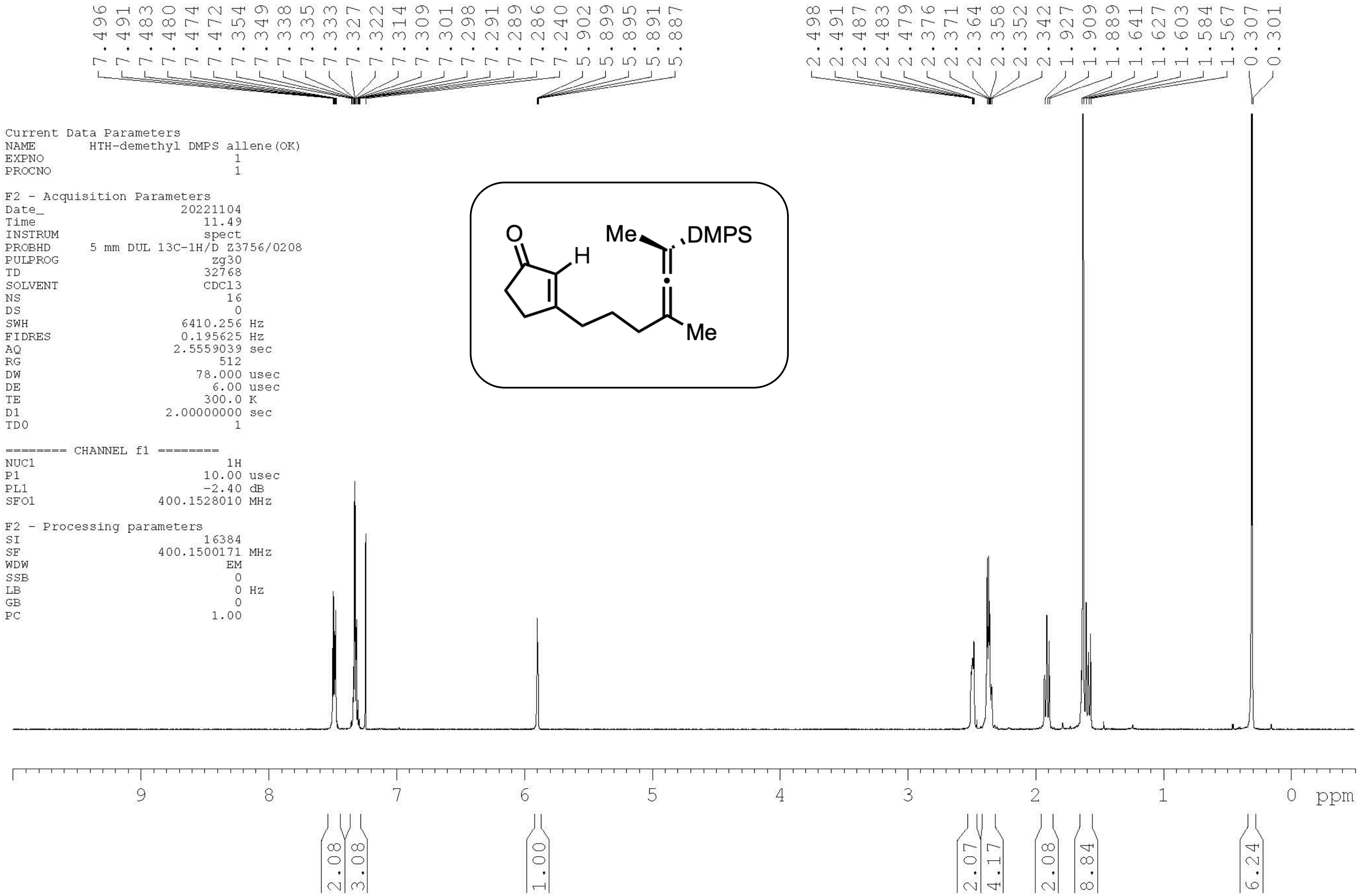

Figure 43. ^{1}H NMR (400 MHz, $CDCl_3$) (**26g**)

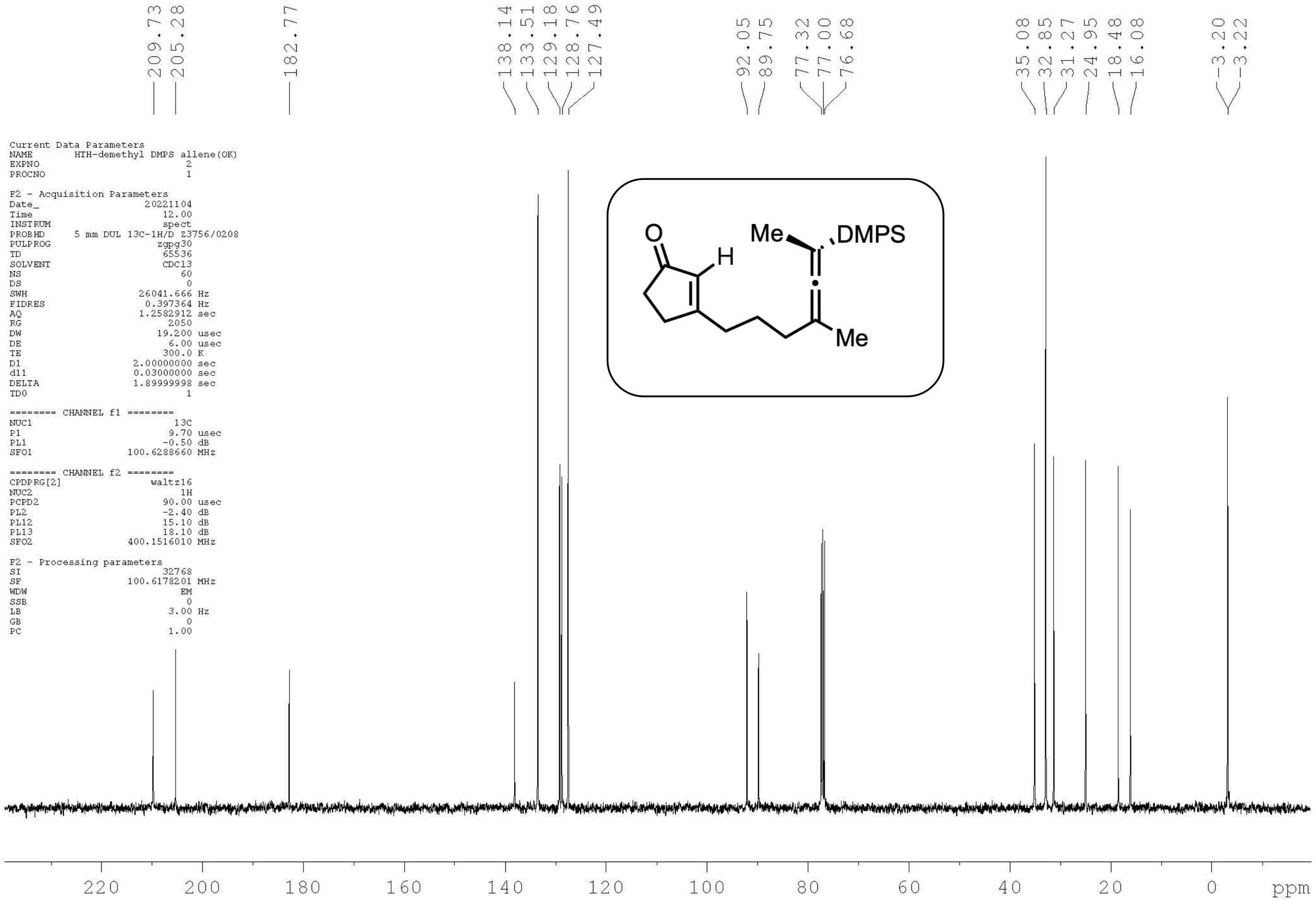

Figure 44. ^{13}C NMR (100 MHz, $CDCl_3$) (**26g**)

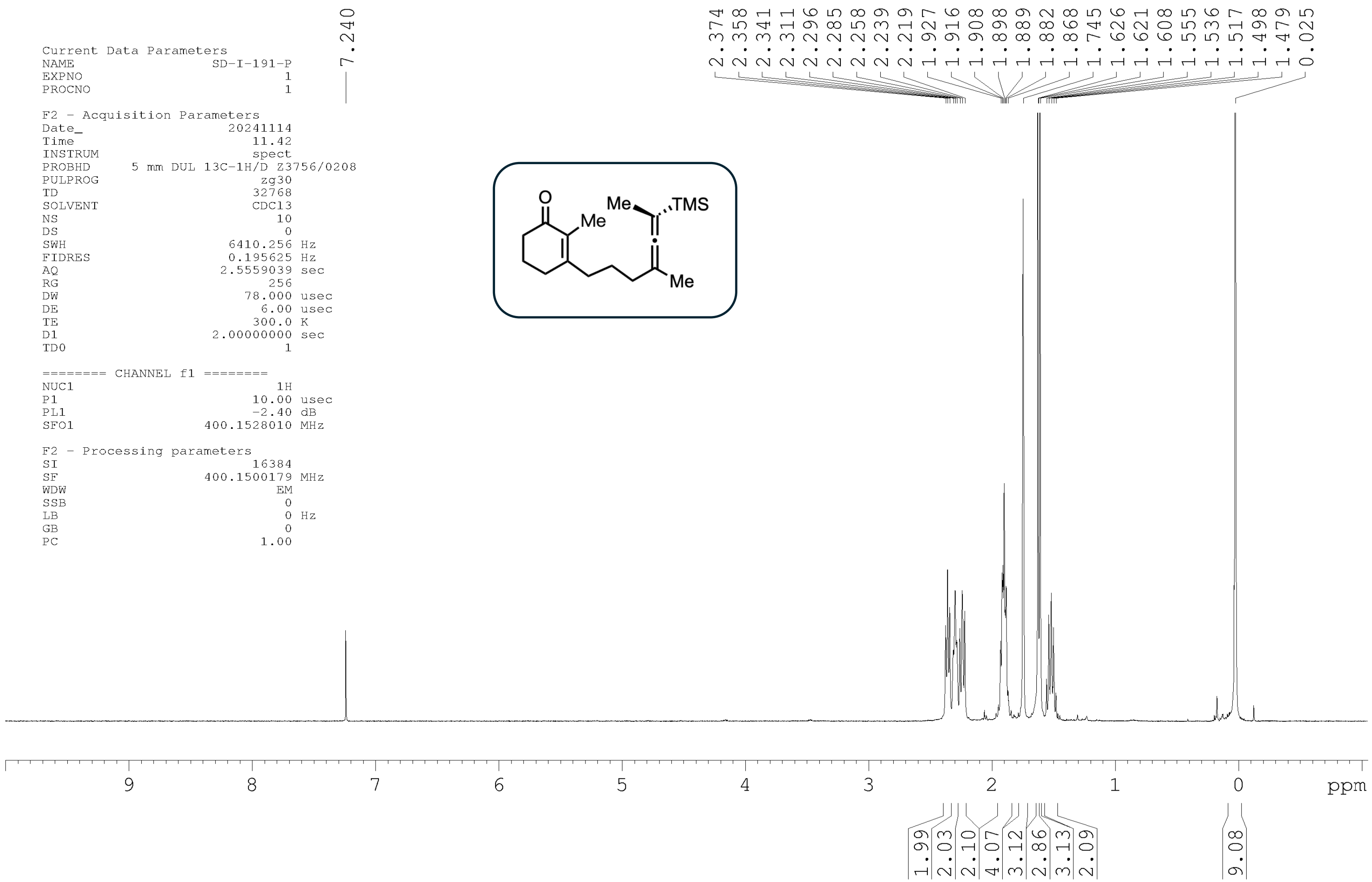

Figure 45. ^{1}H NMR (400 MHz, $CDCl_3$) (**26h**)

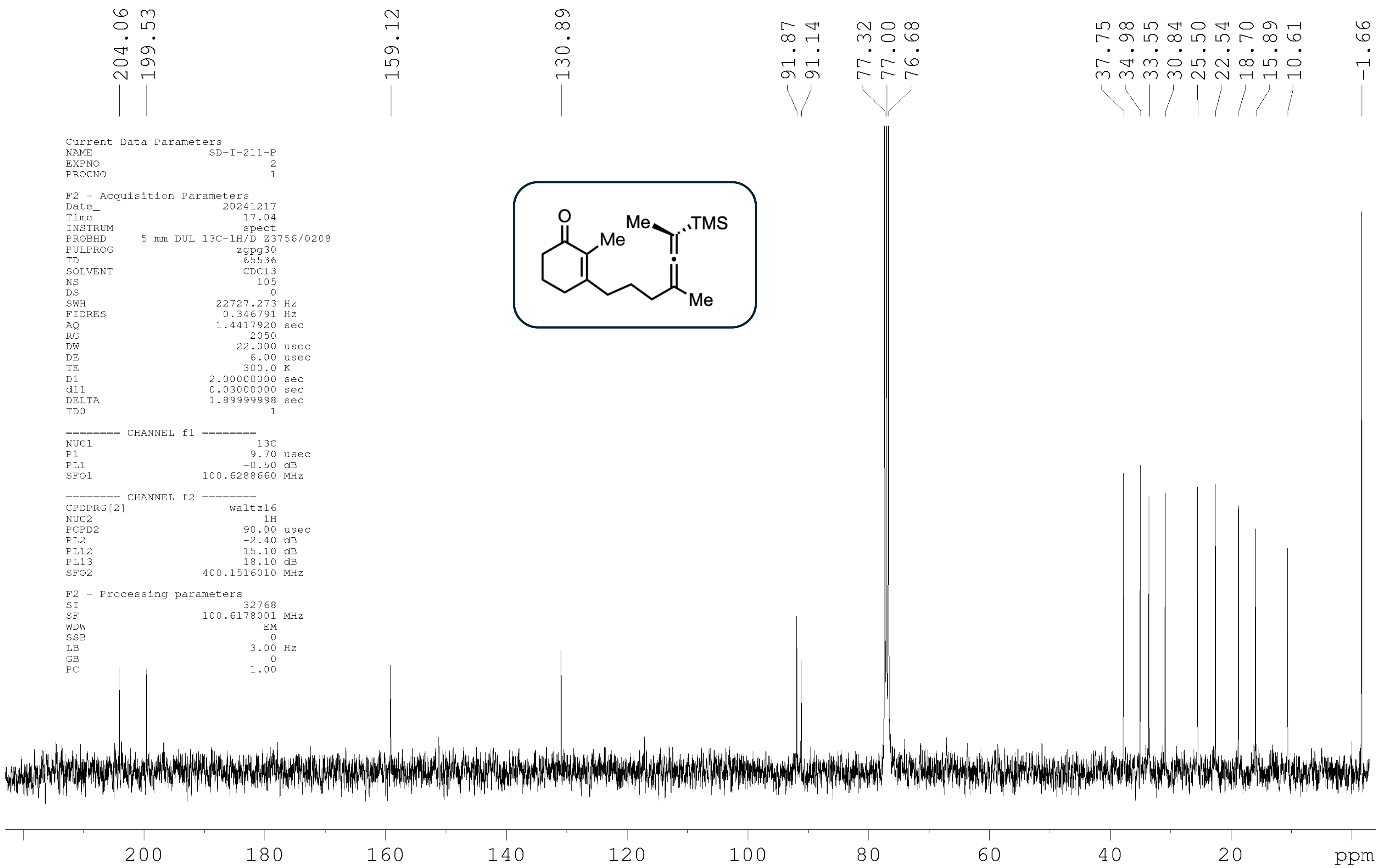

Figure 46. ^{13}C NMR (100 MHz, $CDCl_3$) (**26h**)

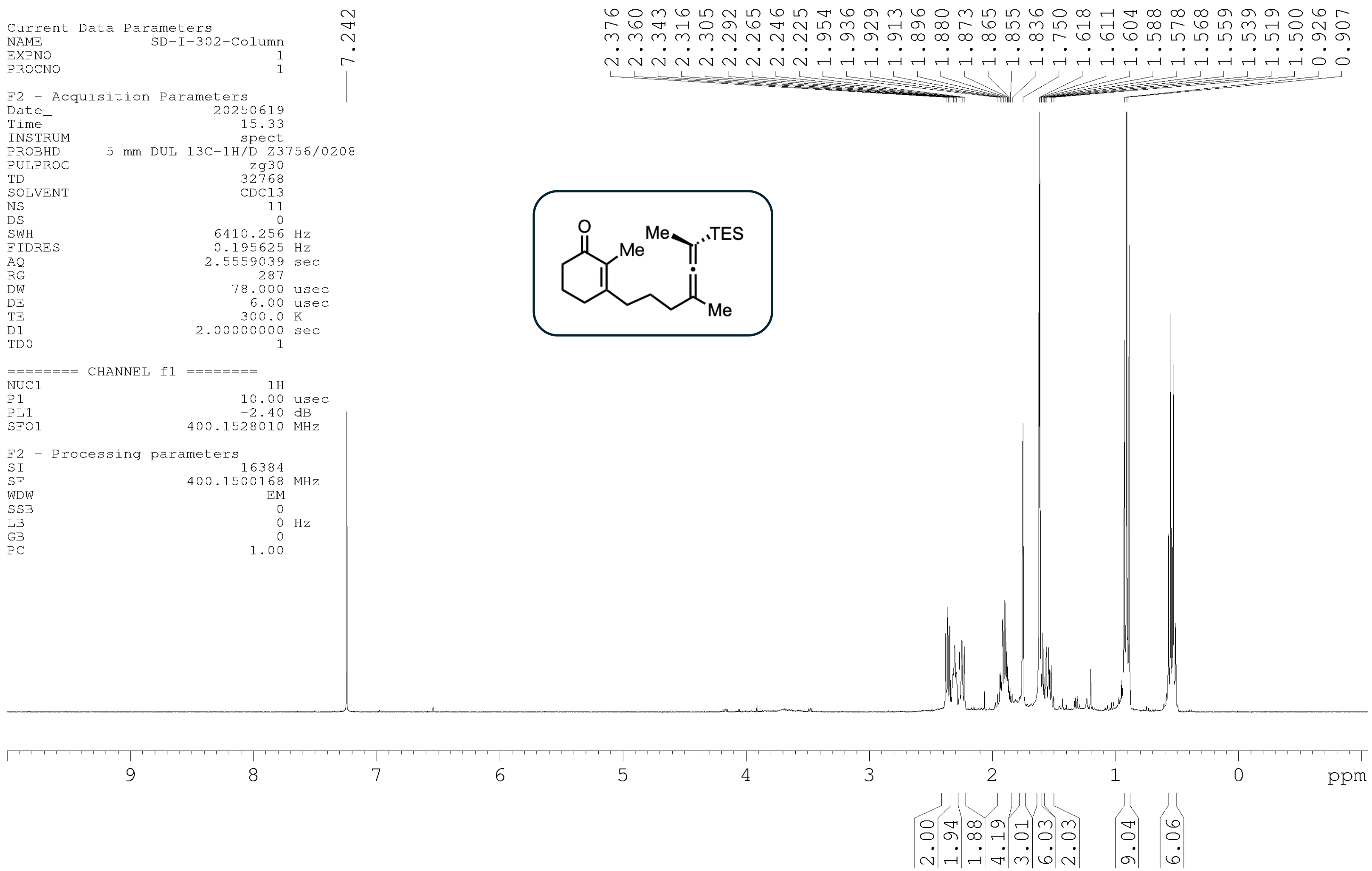

Figure 47. ^{1}H NMR (400 MHz, $CDCl_3$) (**26i**)

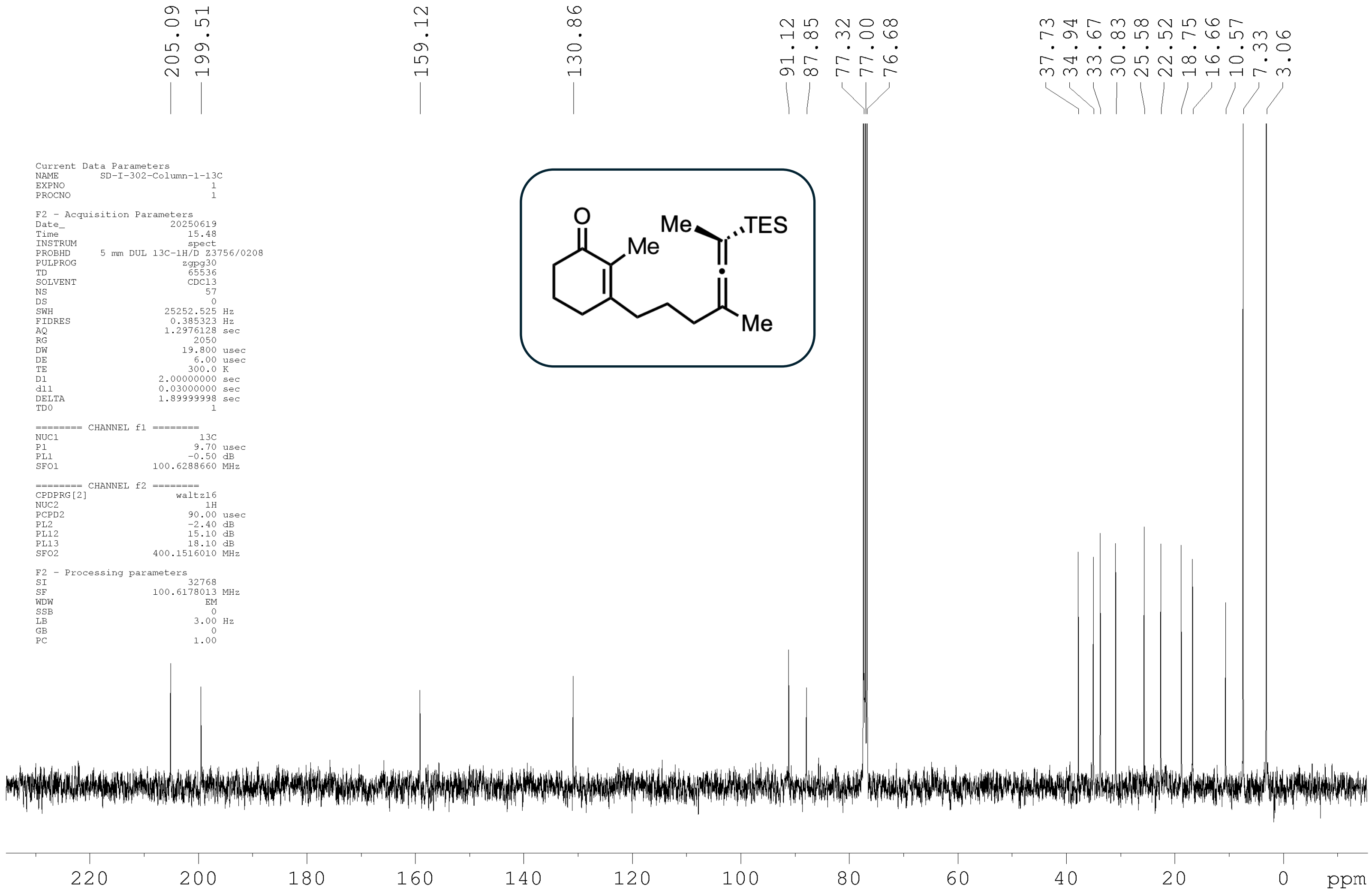

Figure 48. ^{13}C NMR (100 MHz, $CDCl_3$) (**26i**)

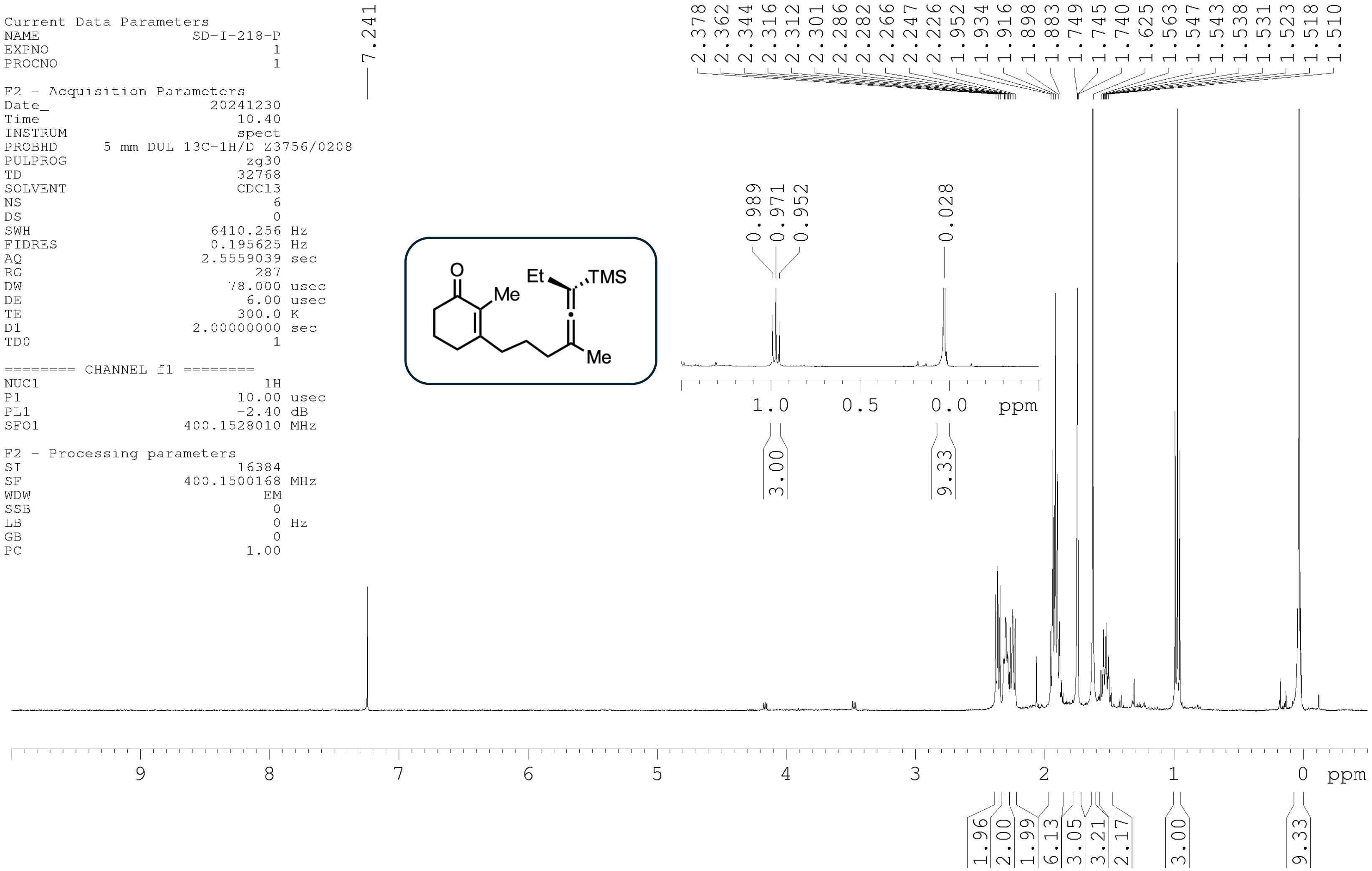

Figure 49. ^{1}H NMR (400 MHz, $CDCl_3$) (**26j**)

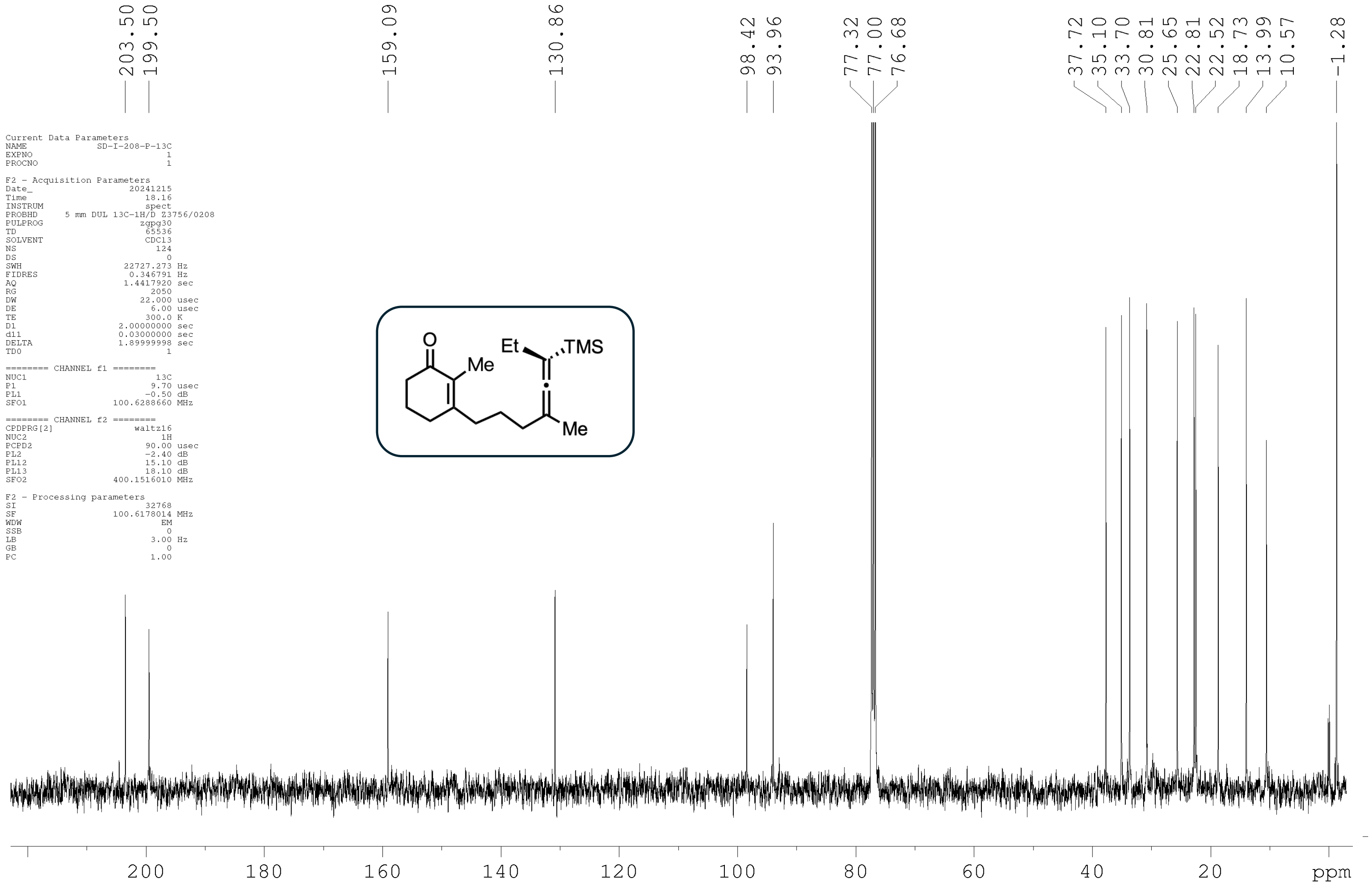

Figure 50. ^{13}C NMR (100 MHz, CDCl$_3$) (**26j**)

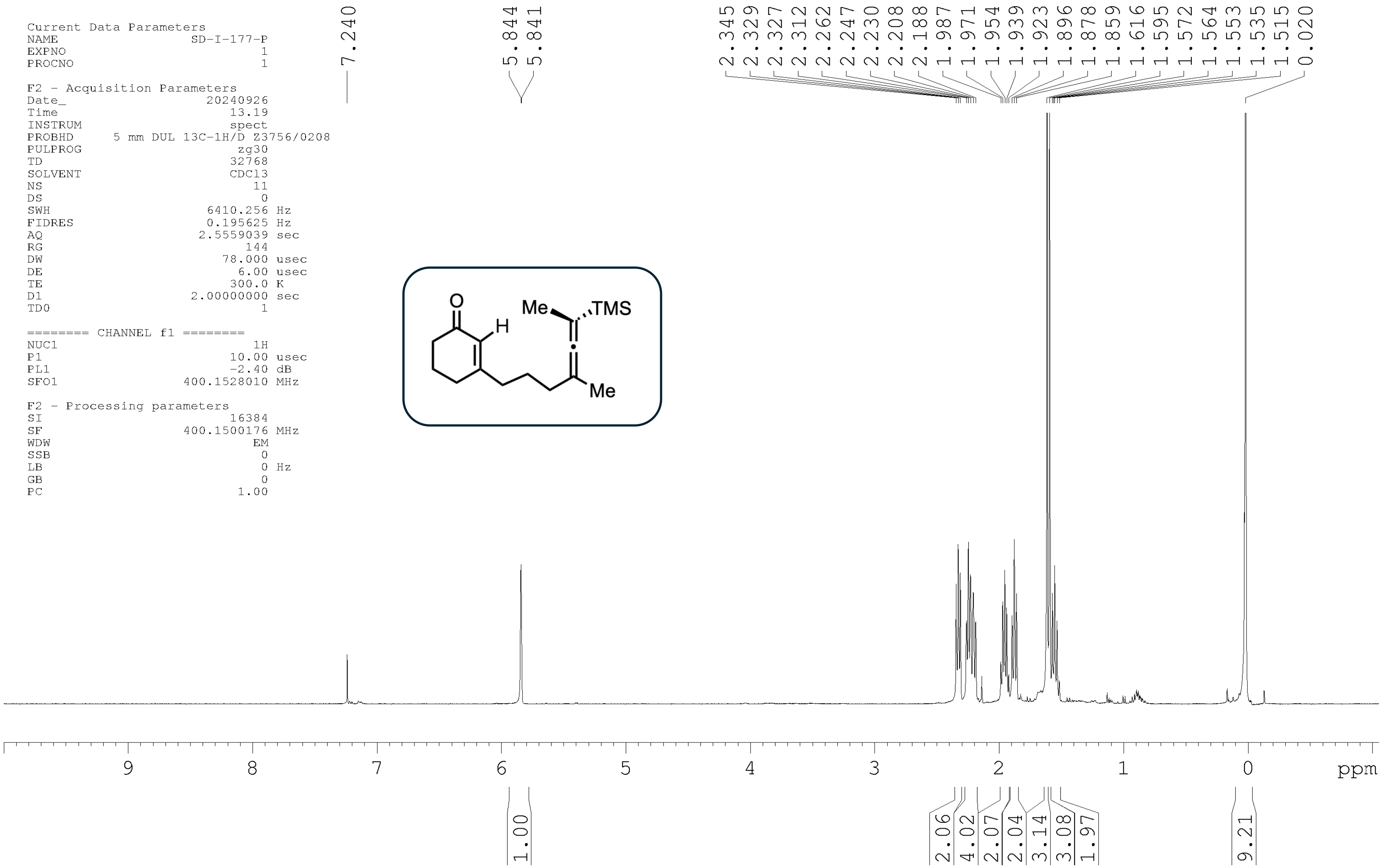

Figure 51. ^{1}H NMR (400 MHz, $CDCl_3$) (**26k**)

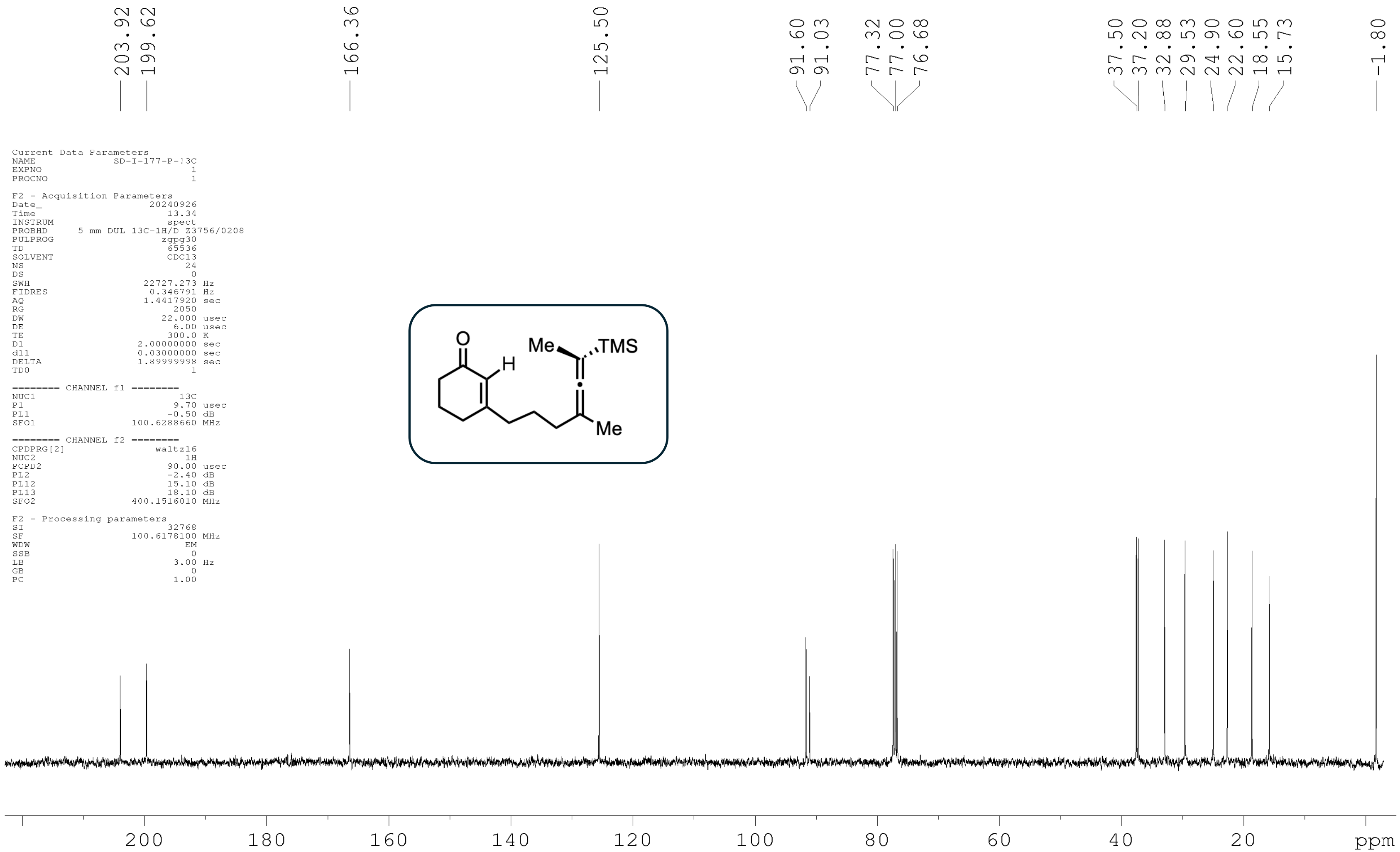

Figure 52. ^{13}C NMR (100 MHz, $CDCl_3$) (**26k**)

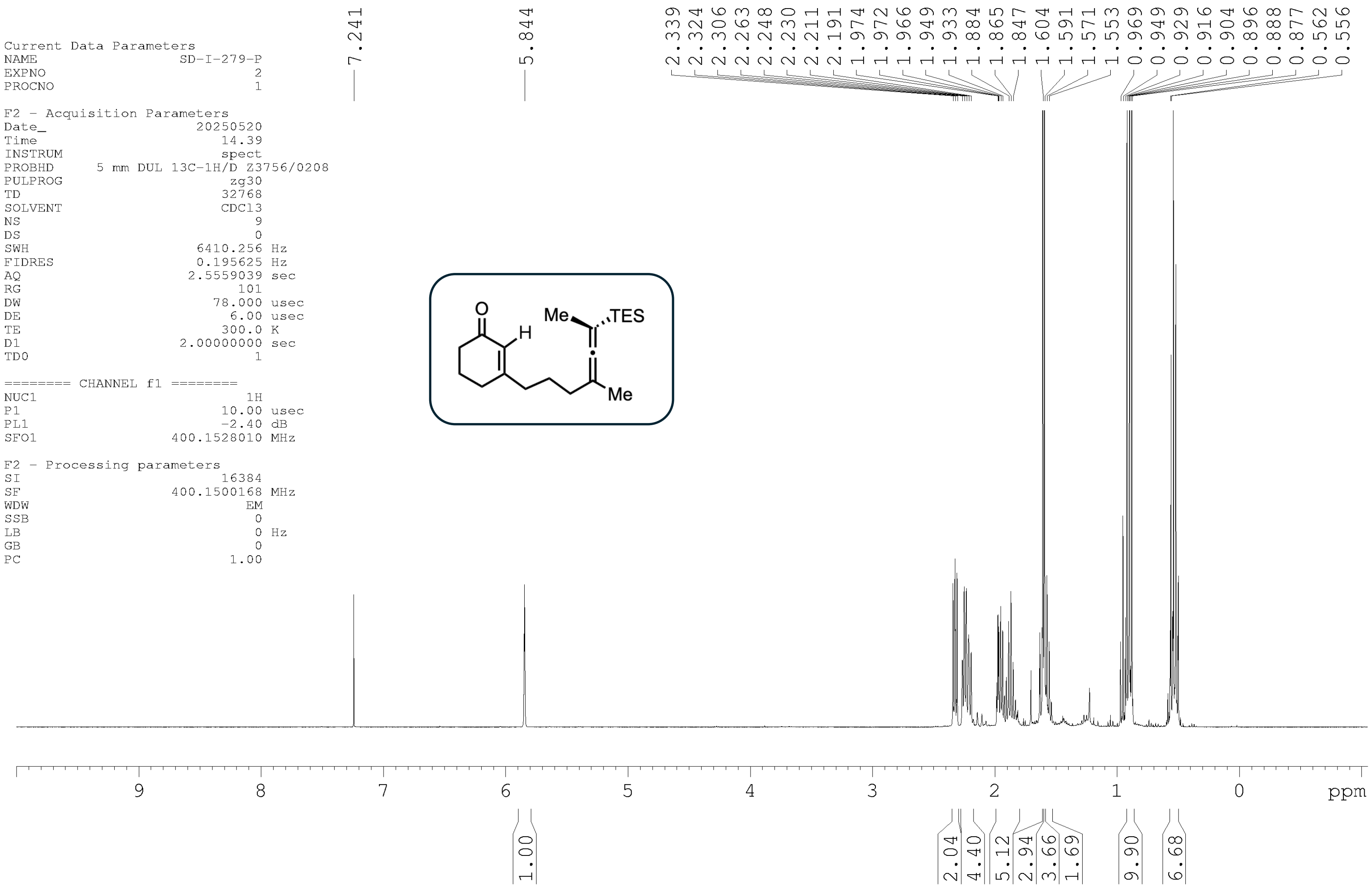

Figure 53. ^{1}H NMR (400 MHz, $CDCl_3$) (**26l**)

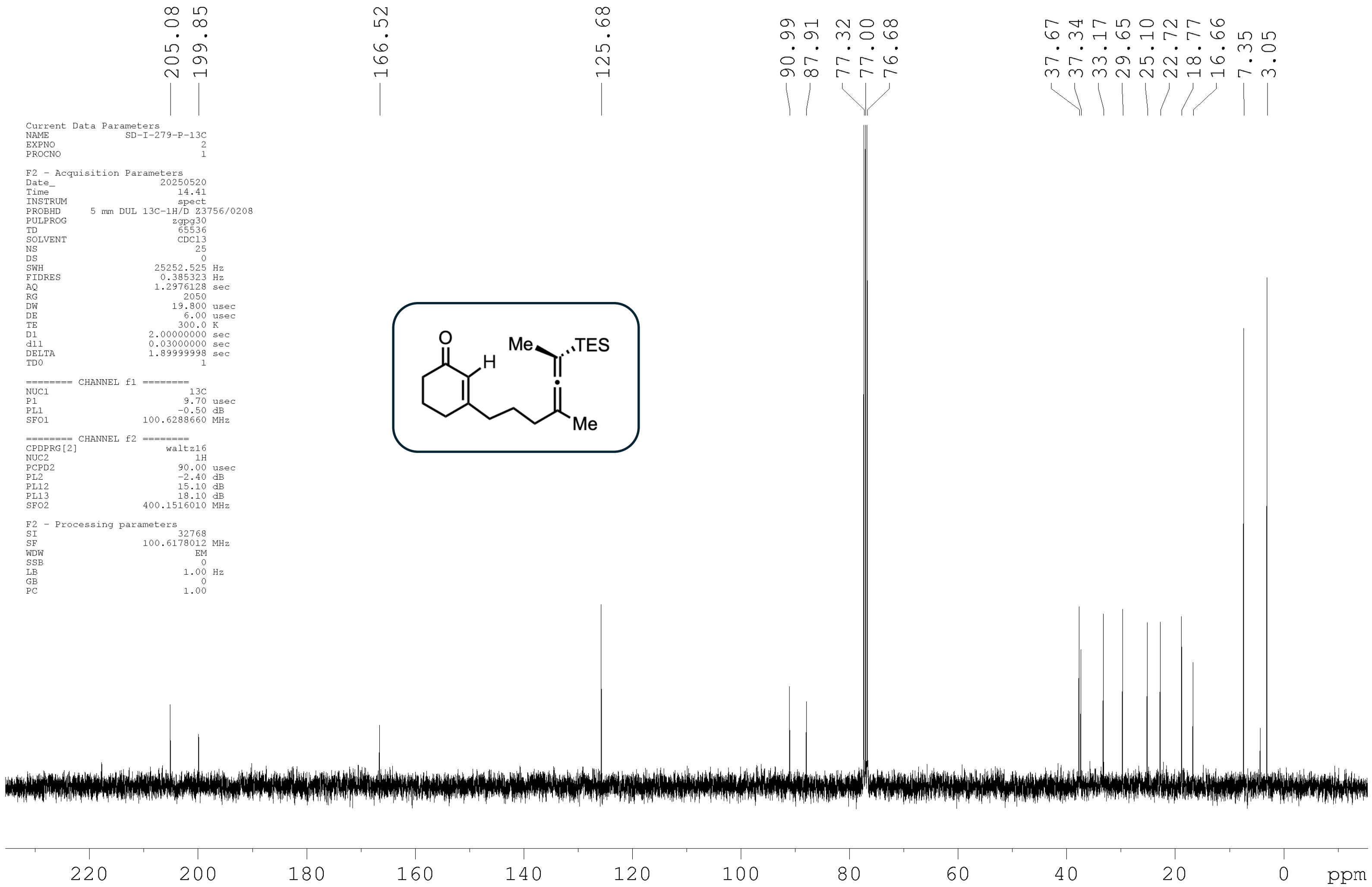

Figure 54. ^{13}C NMR (100 MHz, $CDCl_3$) (**26l**)

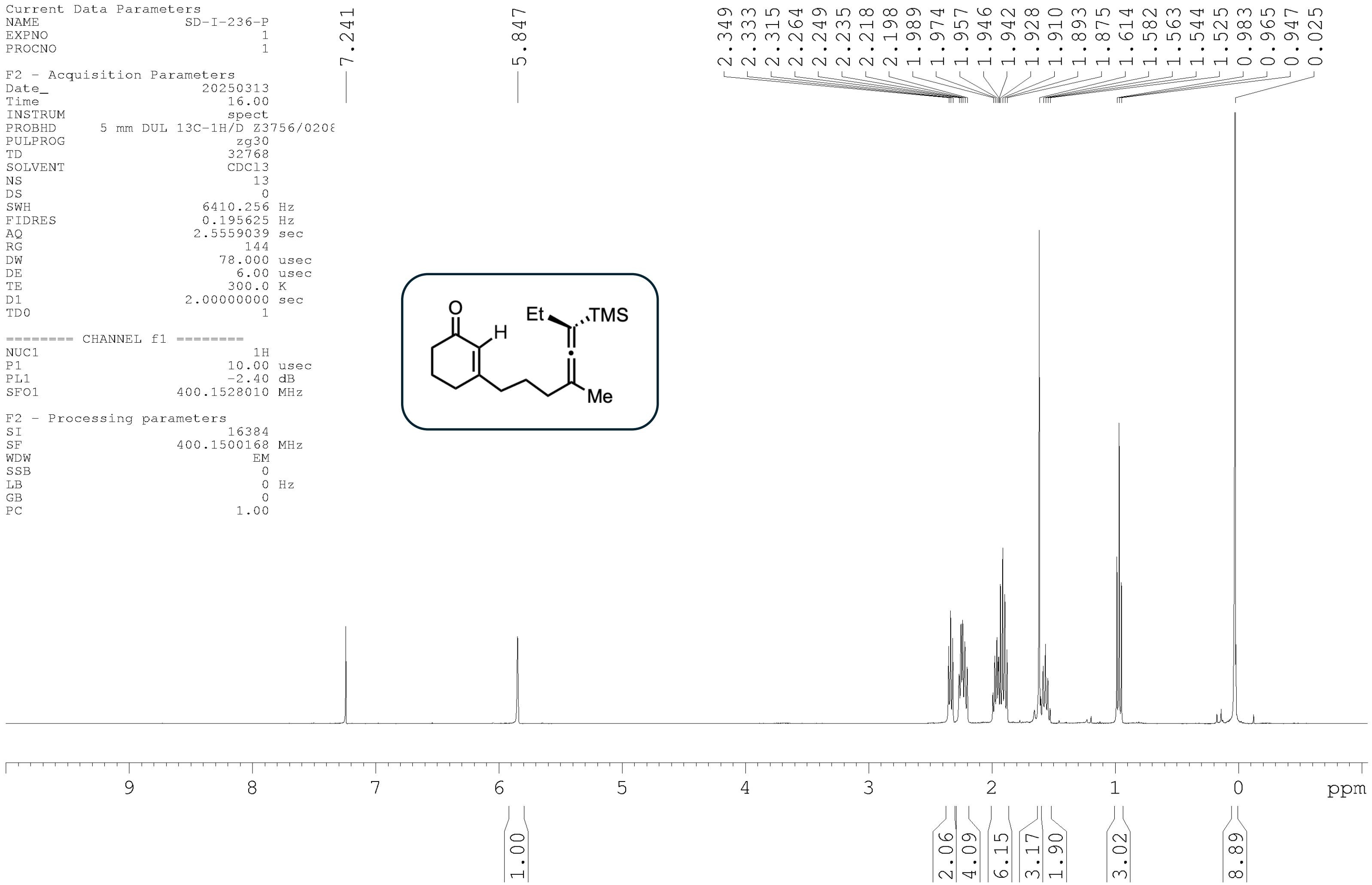

Figure 55. ^{1}H NMR (400 MHz, $CDCl_3$) (**26m**)

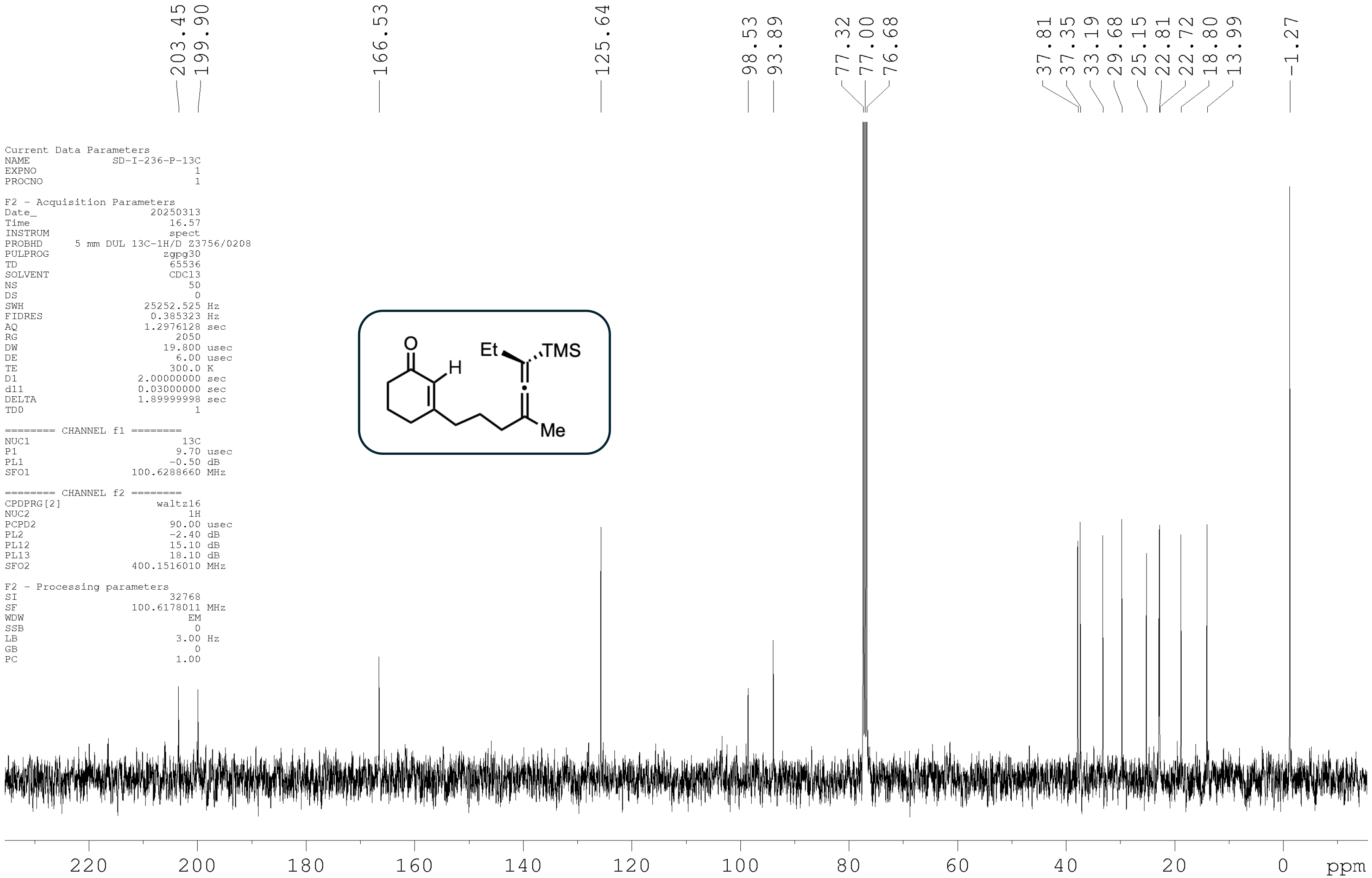

Figure 56. ^{13}C NMR (100 MHz, $CDCl_3$) (**26m**)

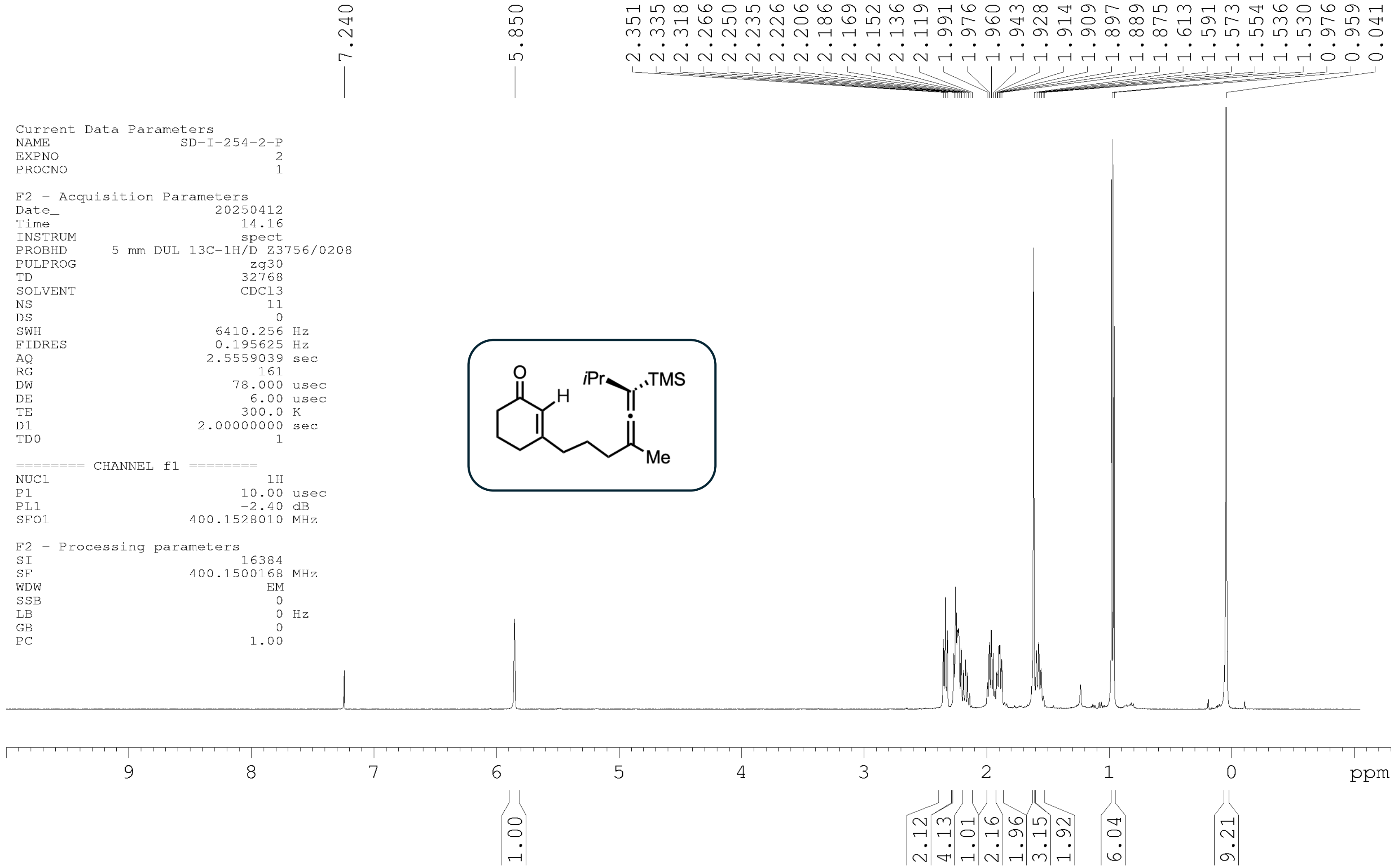

Figure 57. ^{1}H NMR (400 MHz, $CDCl_3$) (**26n**)

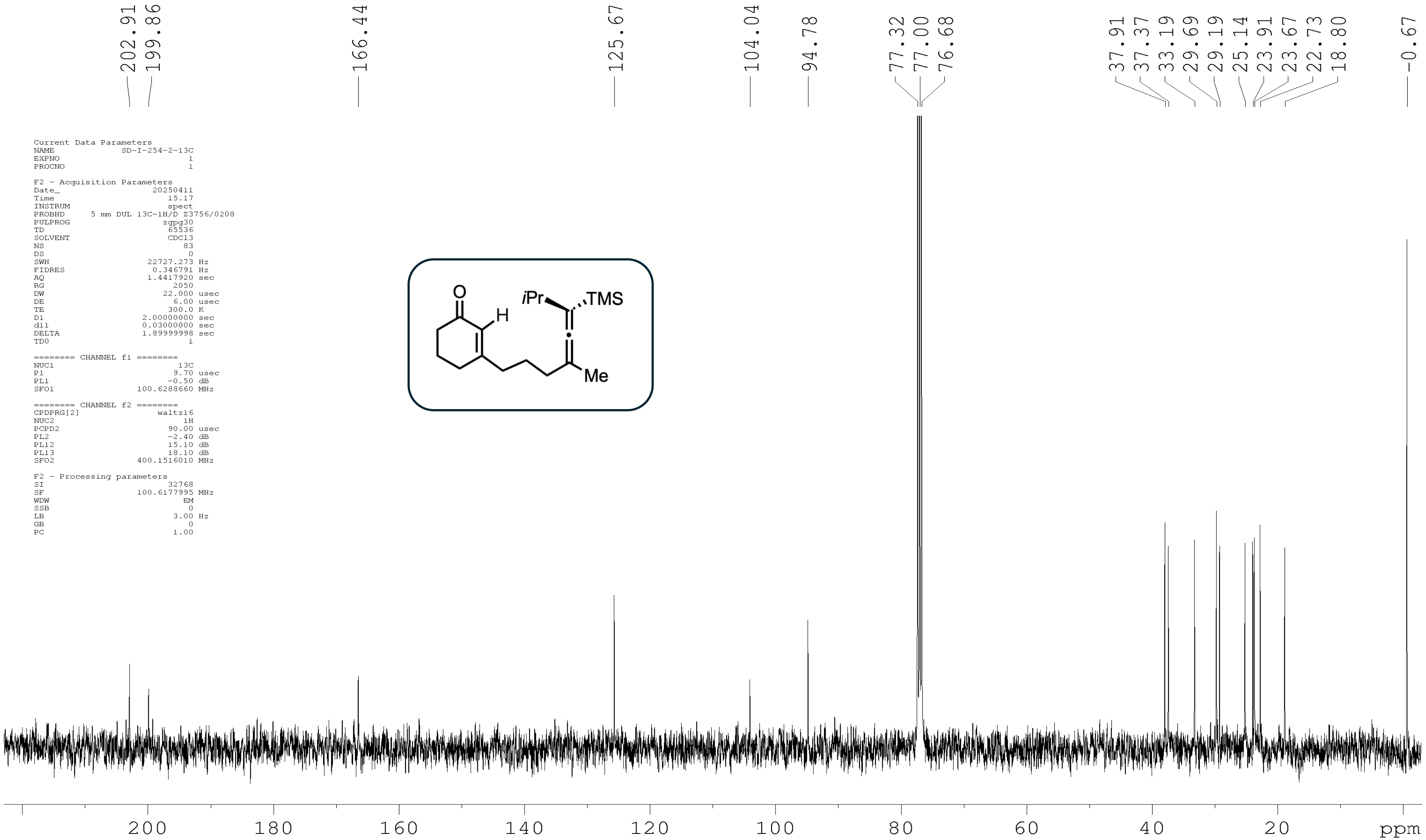

Figure 58. ^{13}C NMR (100 MHz, $CDCl_3$) (**26n**)

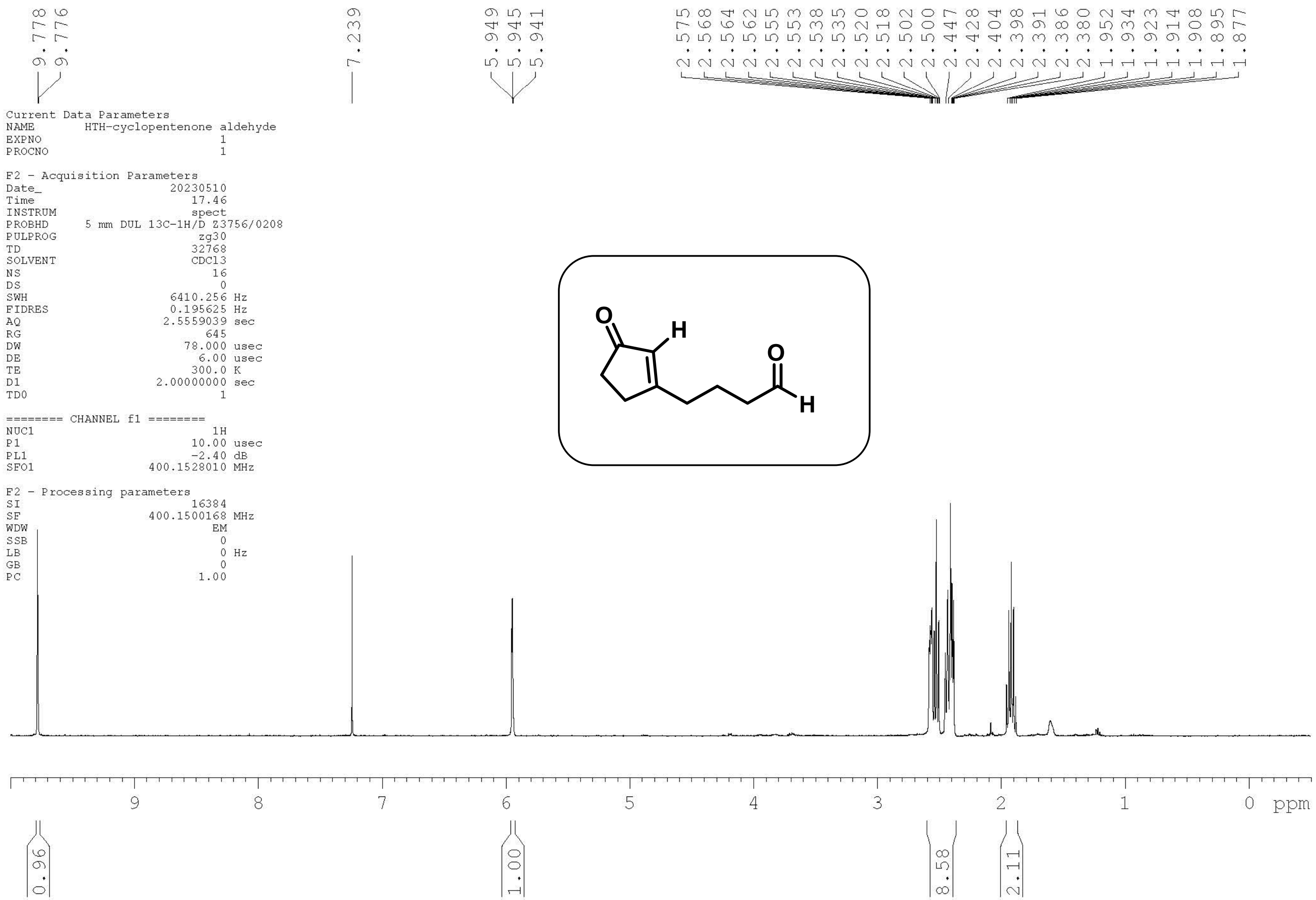

Figure 59. ^{1}H NMR (400 MHz, $CDCl_3$) Danheiser transposition (**SI-2**)

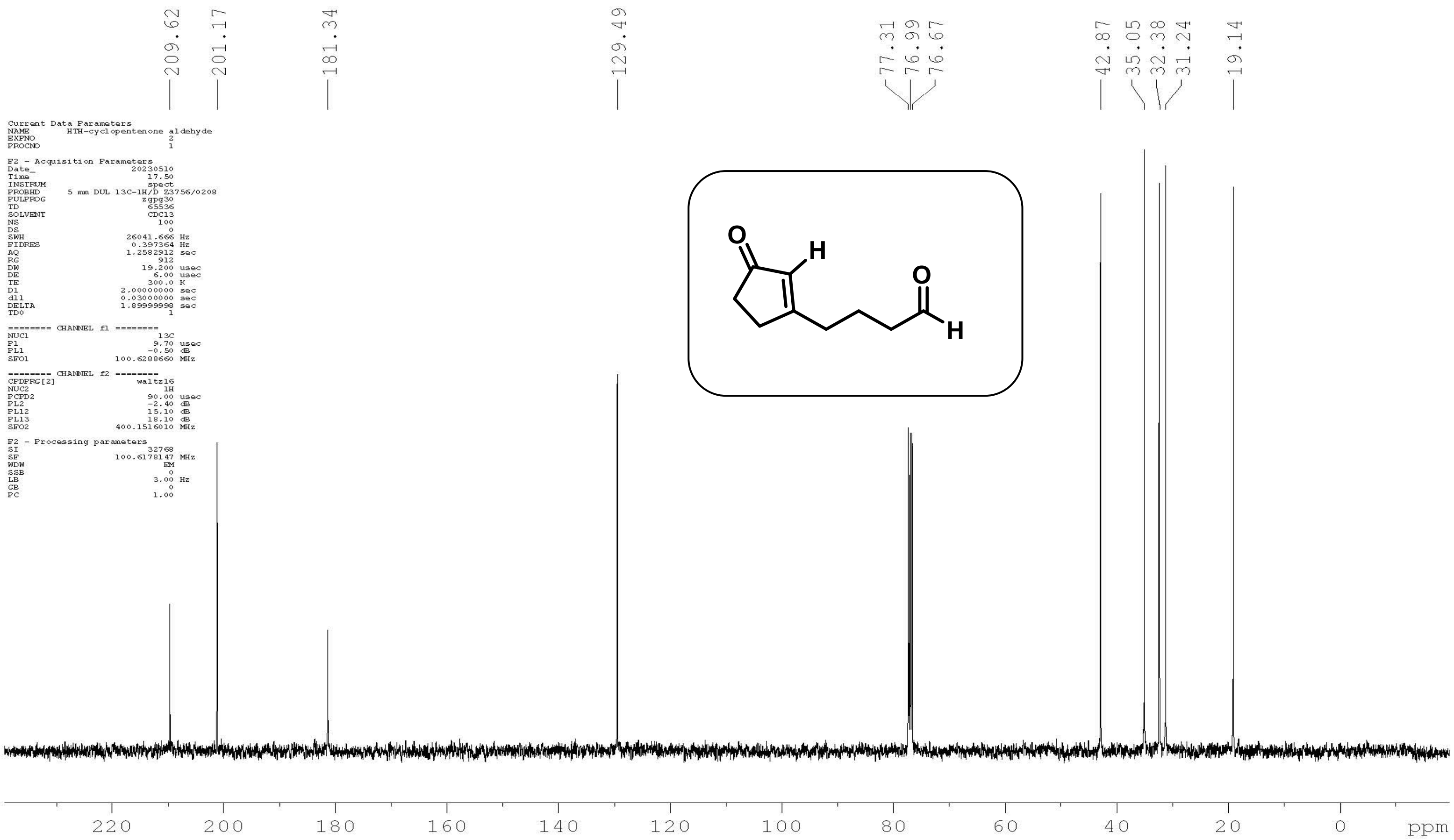

Figure 60. ^{13}C NMR (100 MHz, $CDCl_3$) Danheiser transposition (**SI-2**)

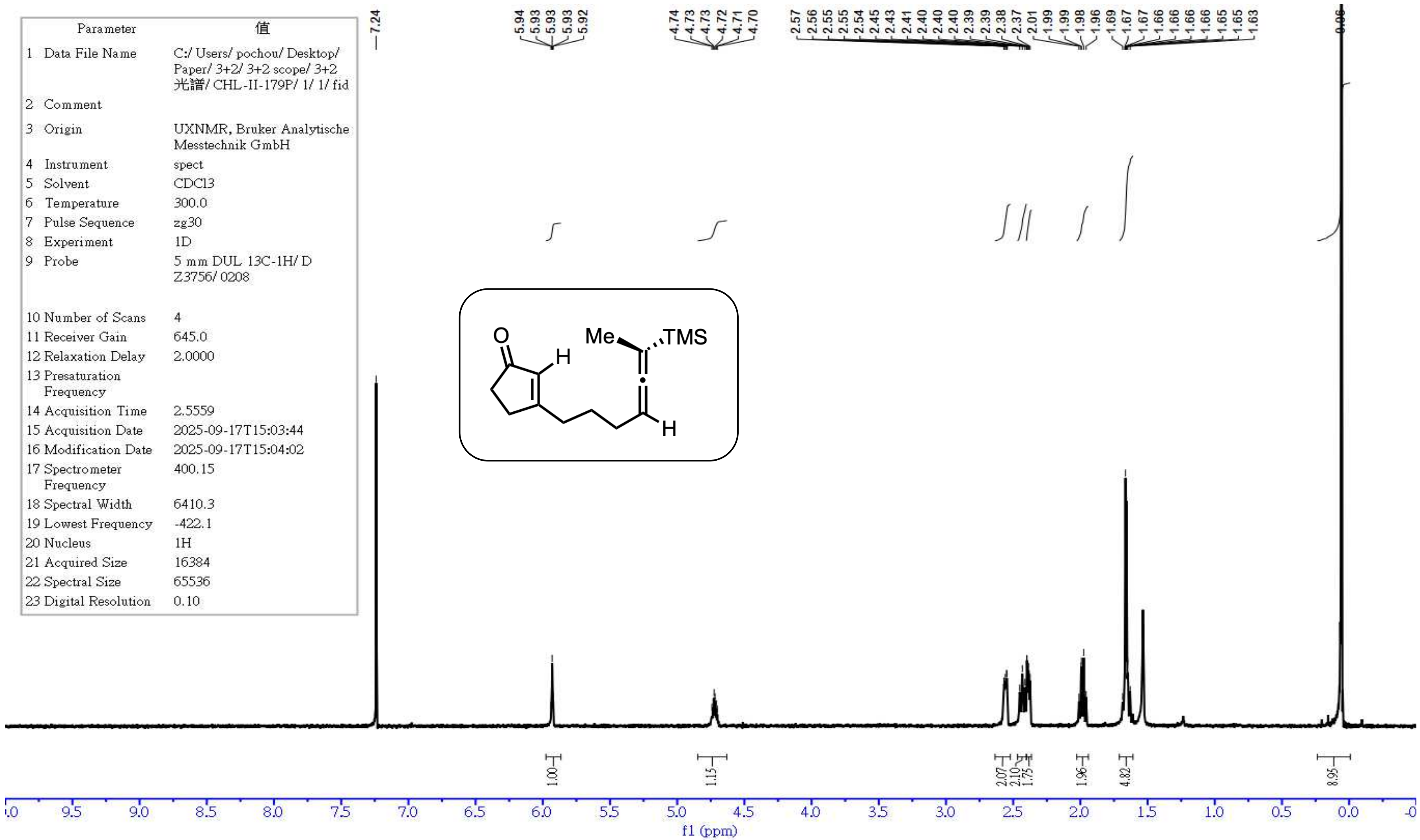

Figure 61. ^{1}H NMR (400 MHz, $CDCl_3$) (**SI-3a**)

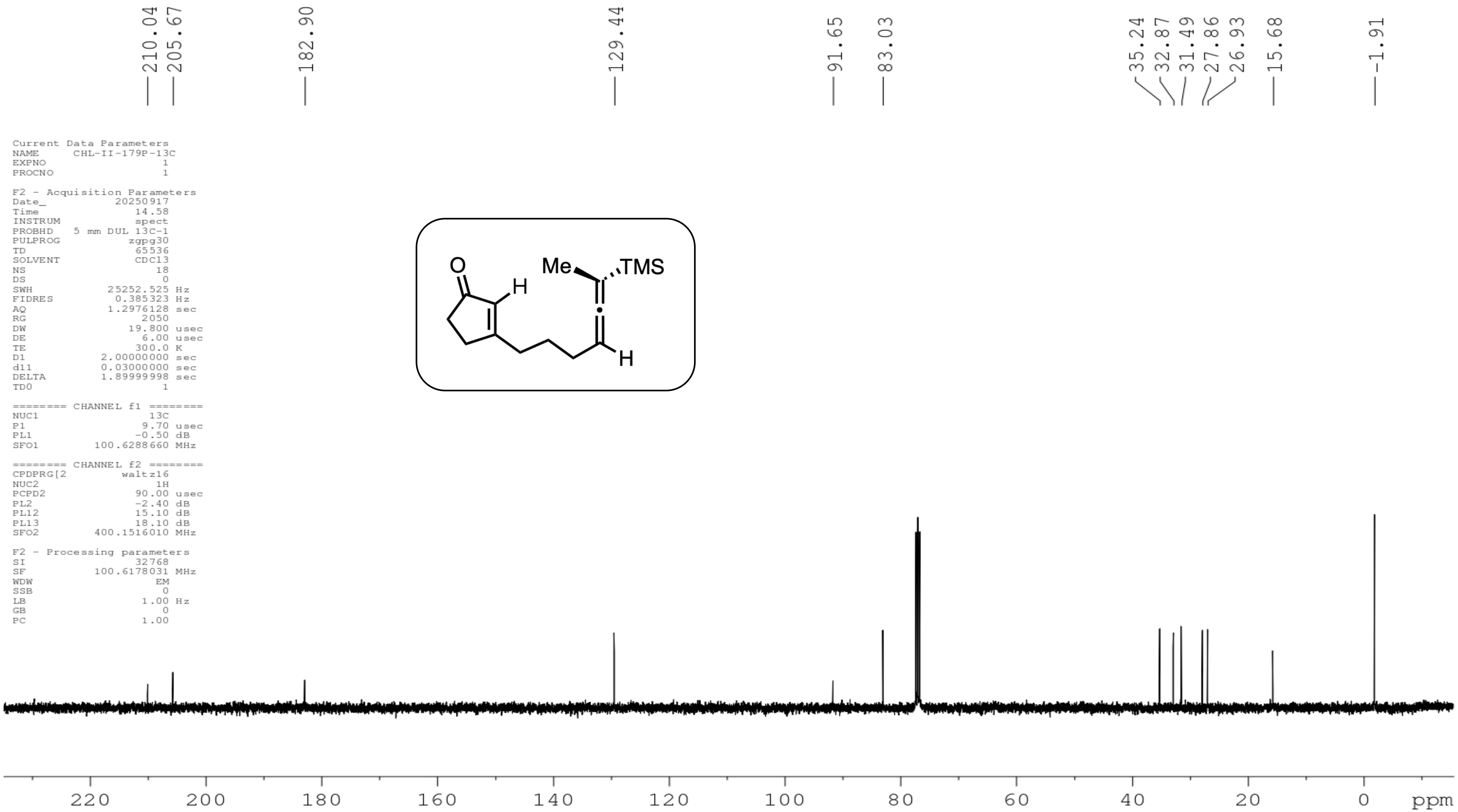

Figure 62. ^{13}C NMR (100 MHz, $CDCl_3$) (**SI-3a**)

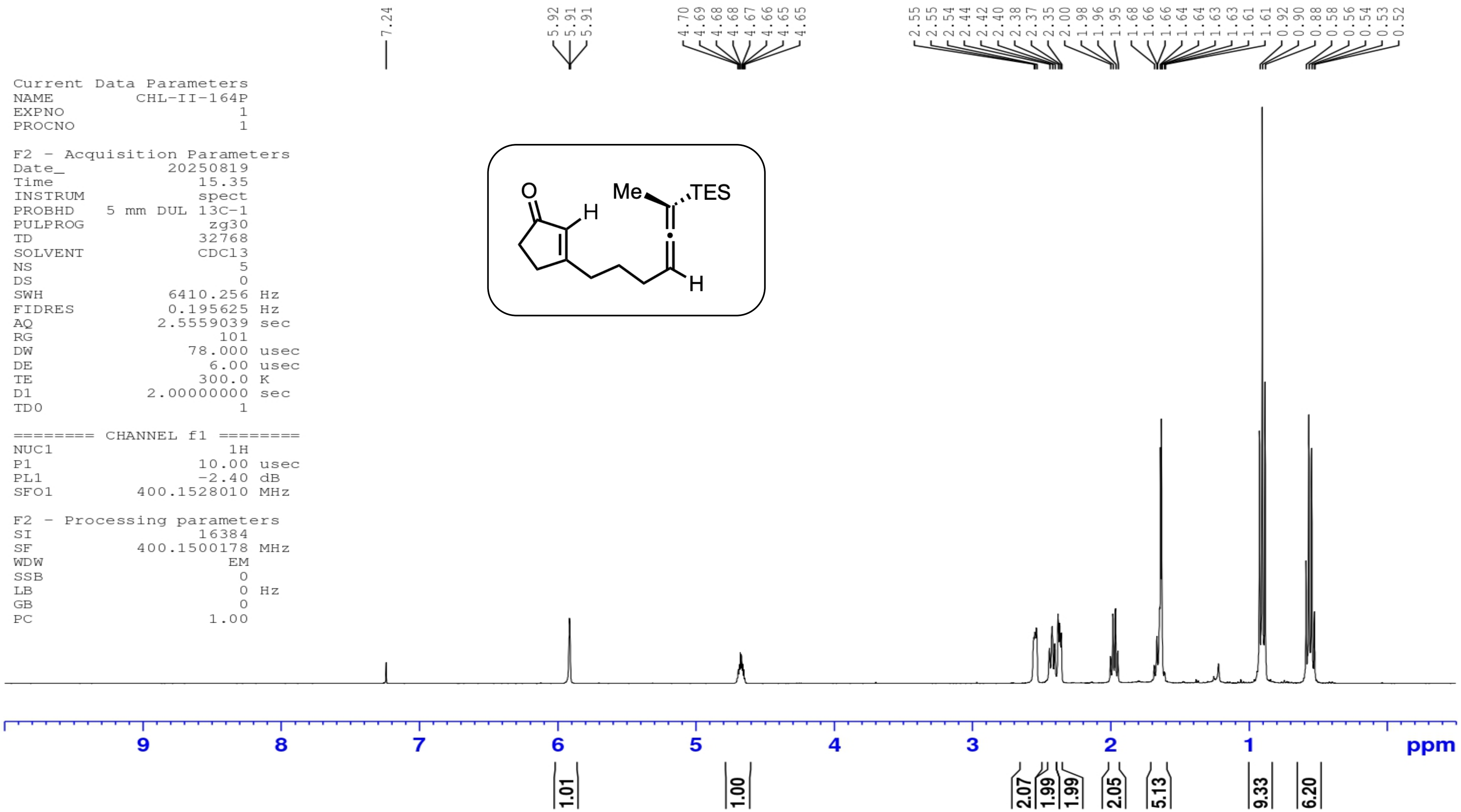

Figure 63. ^{1}H NMR (400 MHz, $CDCl_3$) (**SI-3b**)

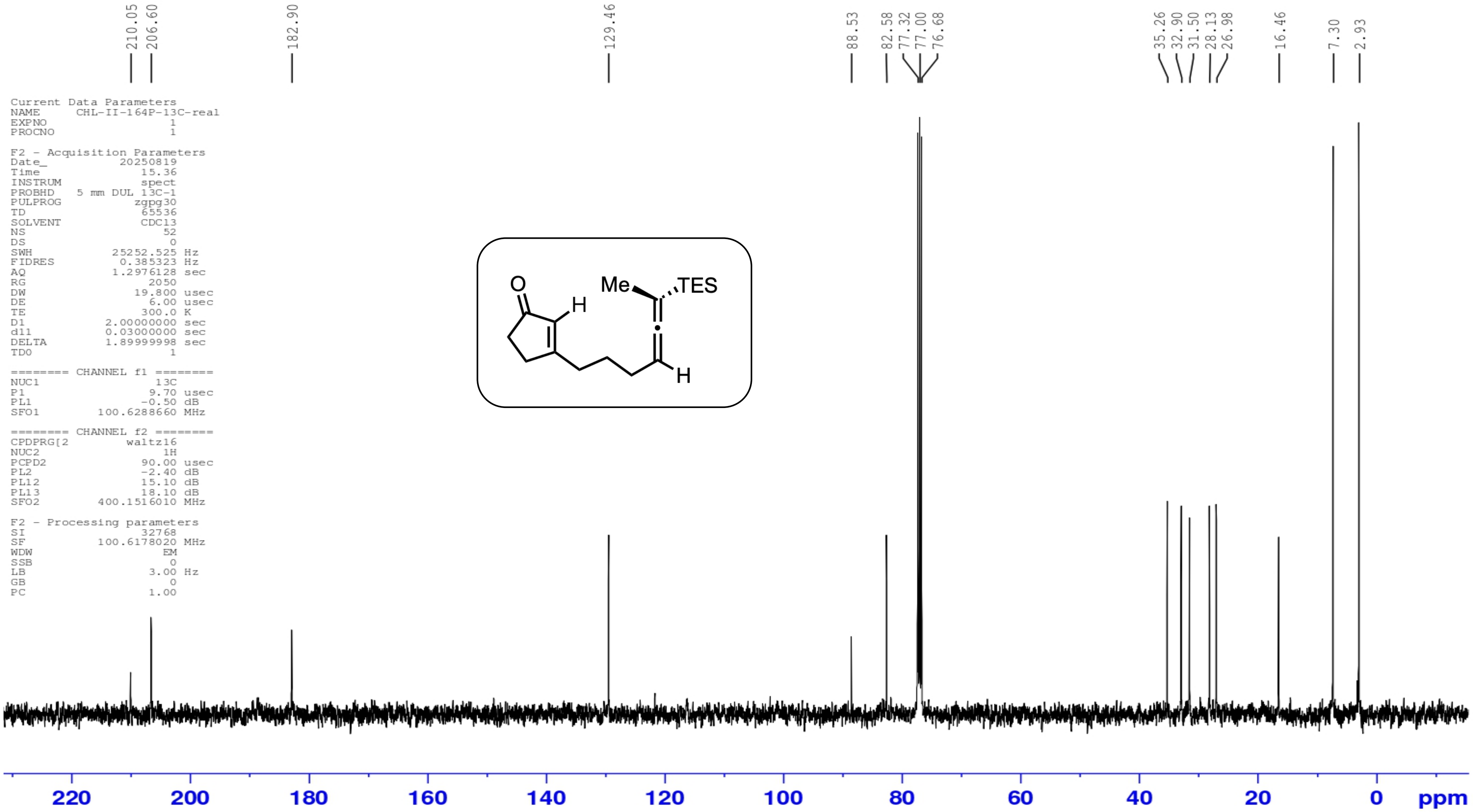

Figure 64. ^{13}C NMR (100 MHz, $CDCl_3$) (**SI-3b**)

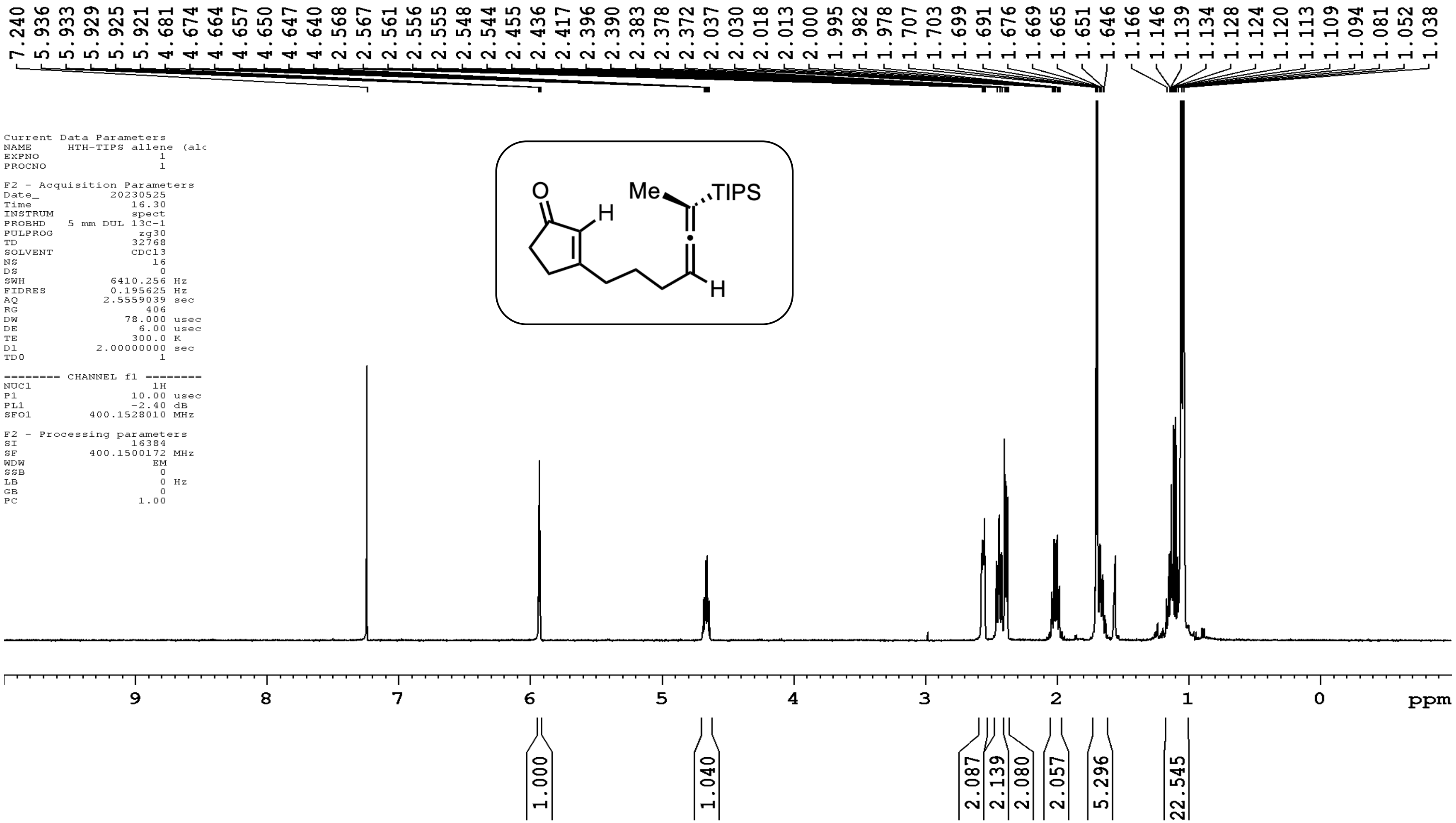

Figure 65. ^{1}H NMR (400 MHz, $CDCl_3$) (**SI-3c**)

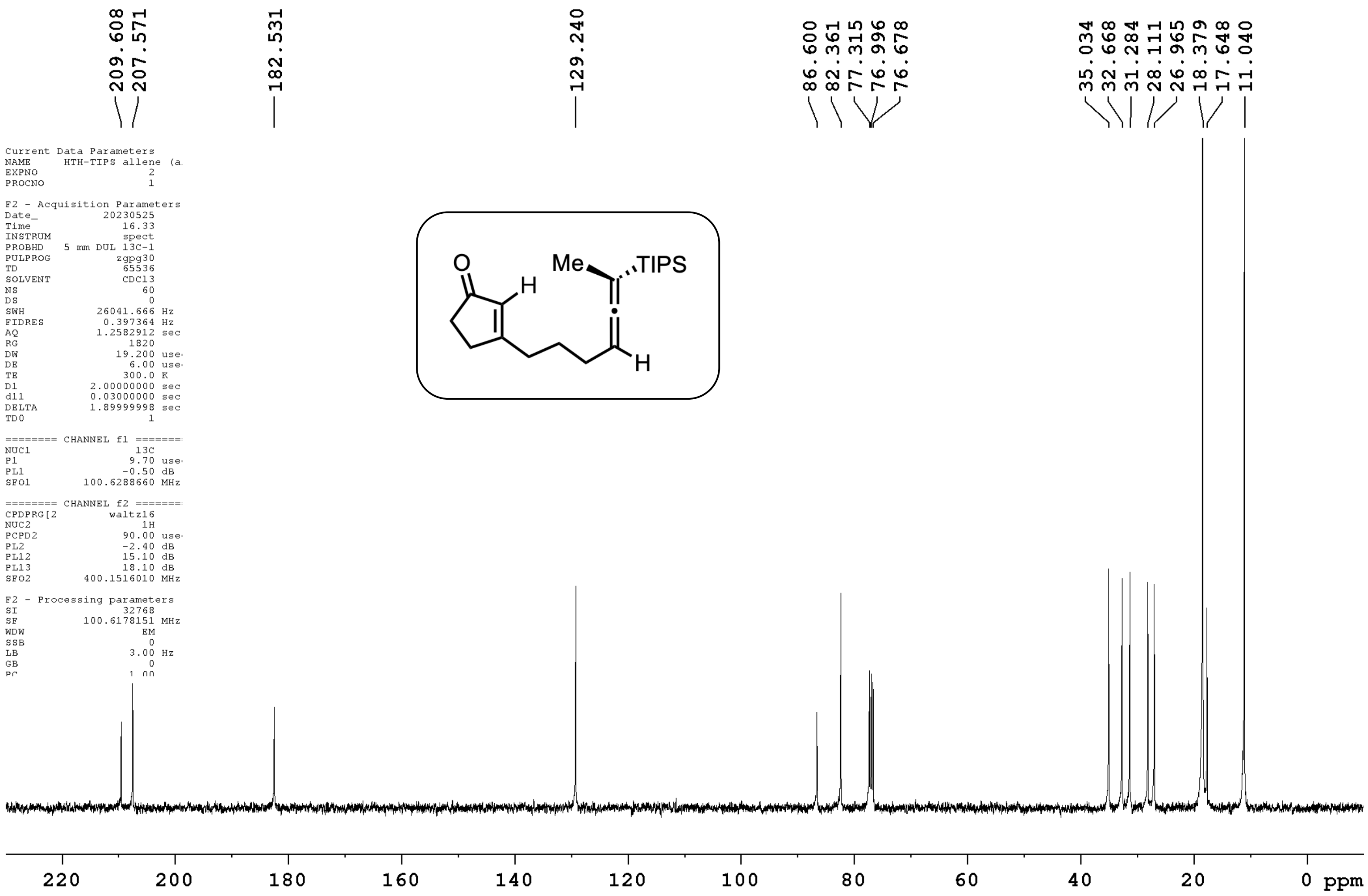

Figure 66. ^{13}C NMR (100 MHz, $CDCl_3$) (**SI-3c**)

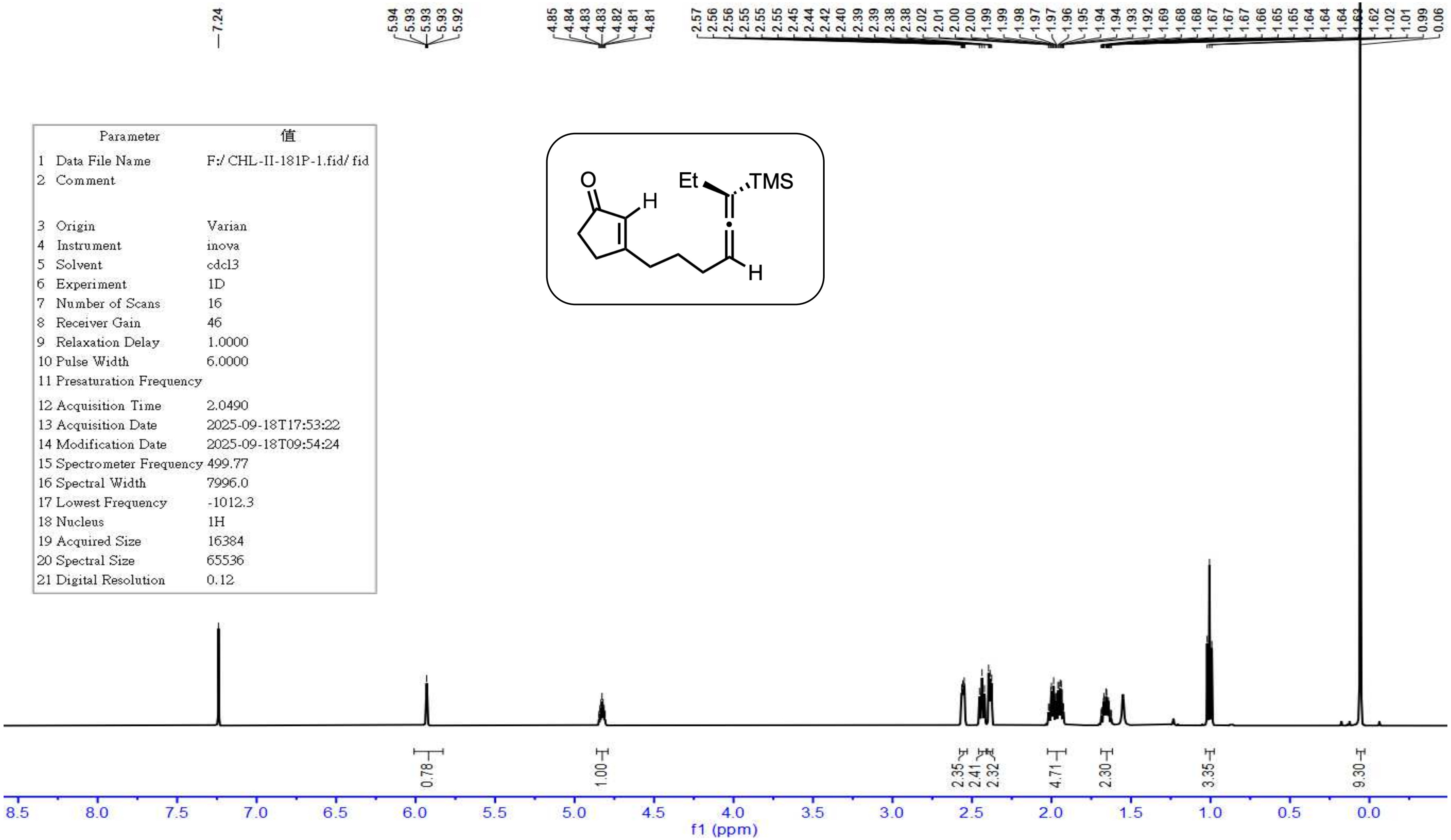

Parameter	值
1 Data File Name	F:/ CHL-II-181P-1.fid/ fid
2 Comment	
3 Origin	Varian
4 Instrument	inova
5 Solvent	cdcl3
6 Experiment	1D
7 Number of Scans	16
8 Receiver Gain	46
9 Relaxation Delay	1.0000
10 Pulse Width	6.0000
11 Presaturation Frequency	
12 Acquisition Time	2.0490
13 Acquisition Date	2025-09-18T17:53:22
14 Modification Date	2025-09-18T09:54:24
15 Spectrometer Frequency	499.77
16 Spectral Width	7996.0
17 Lowest Frequency	-1012.3
18 Nucleus	1H
19 Acquired Size	16384
20 Spectral Size	65536
21 Digital Resolution	0.12

Figure 67. ^{1}H NMR (400 MHz, $CDCl_3$) (**SI-3d**)

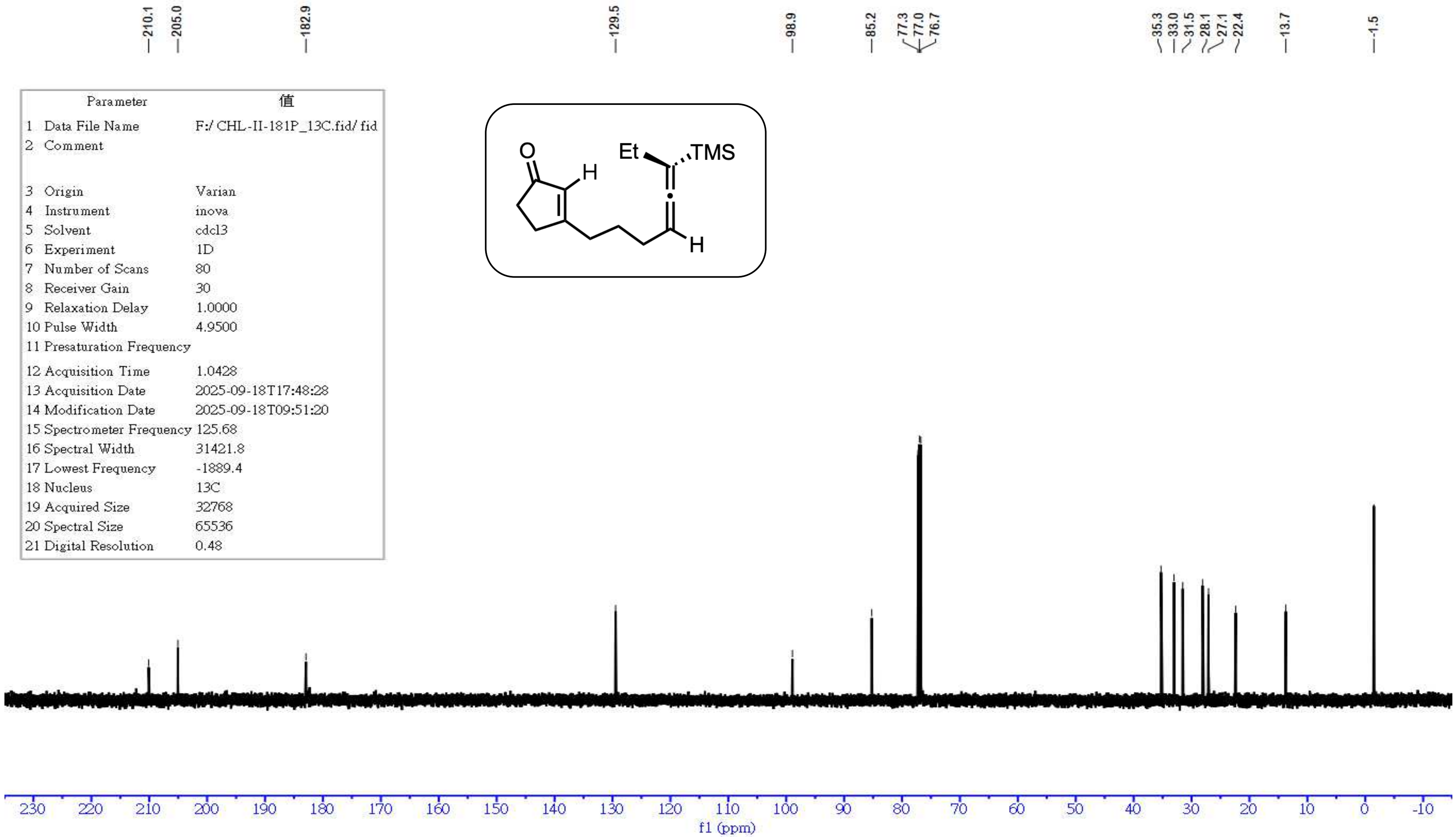

Figure 68. ^{13}C NMR (100 MHz, $CDCl_3$) (**SI-3d**)

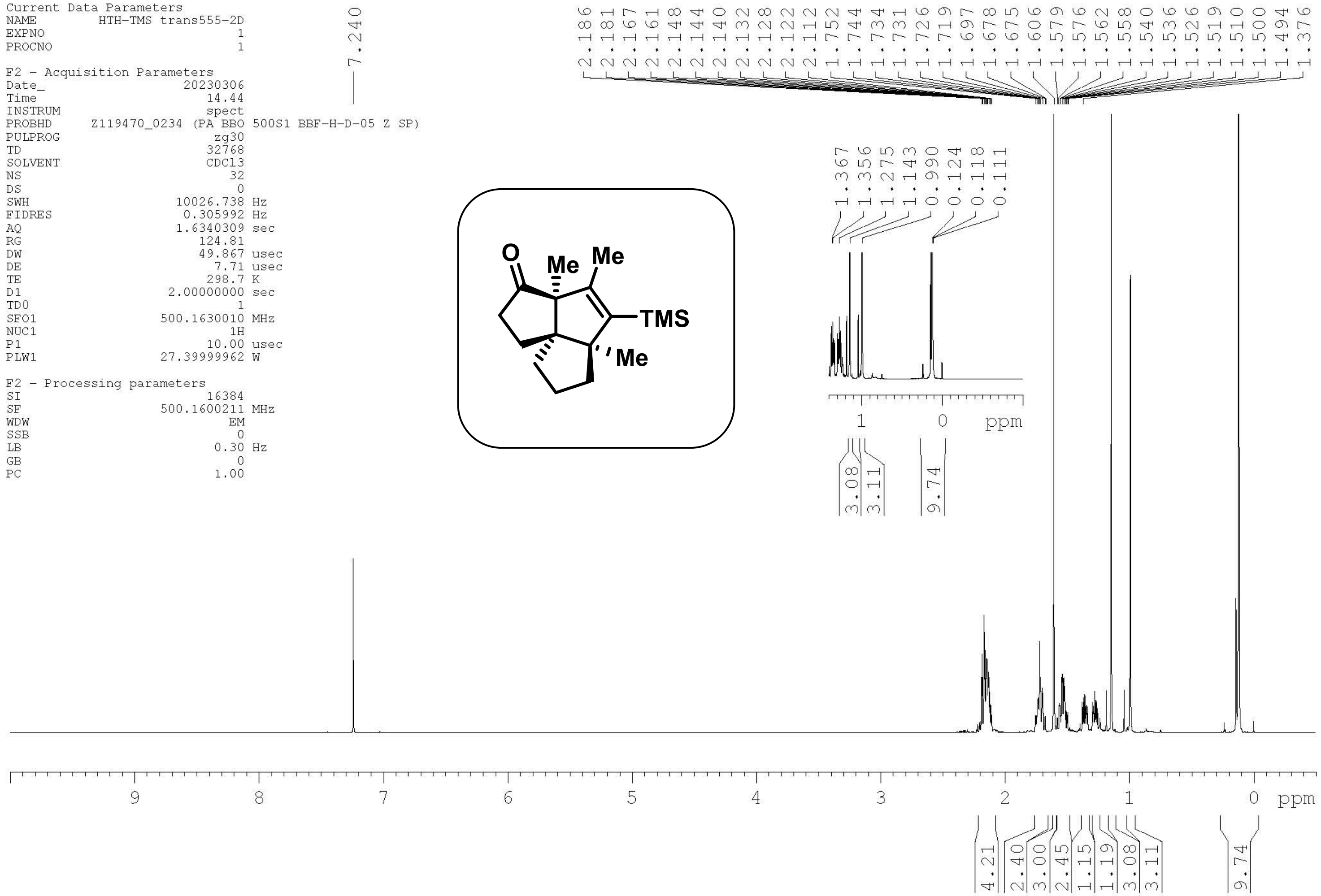

Figure 69. ^{1}H NMR (500 MHz, $CDCl_3$) (**28m-*cis*,*trans***)

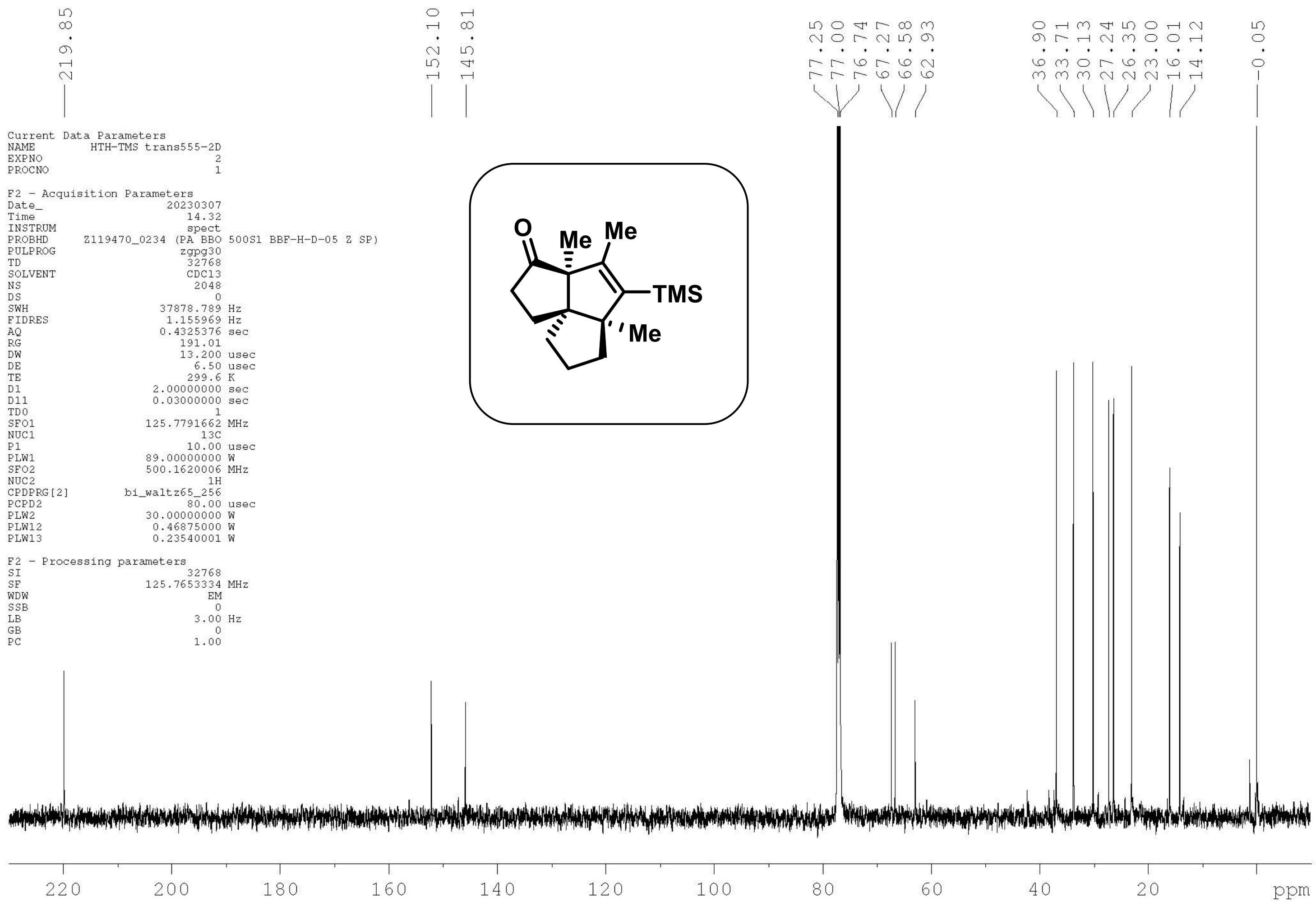

Figure 70. ^{13}C NMR (125 MHz, $CDCl_3$) (**28m-*cis,trans***)

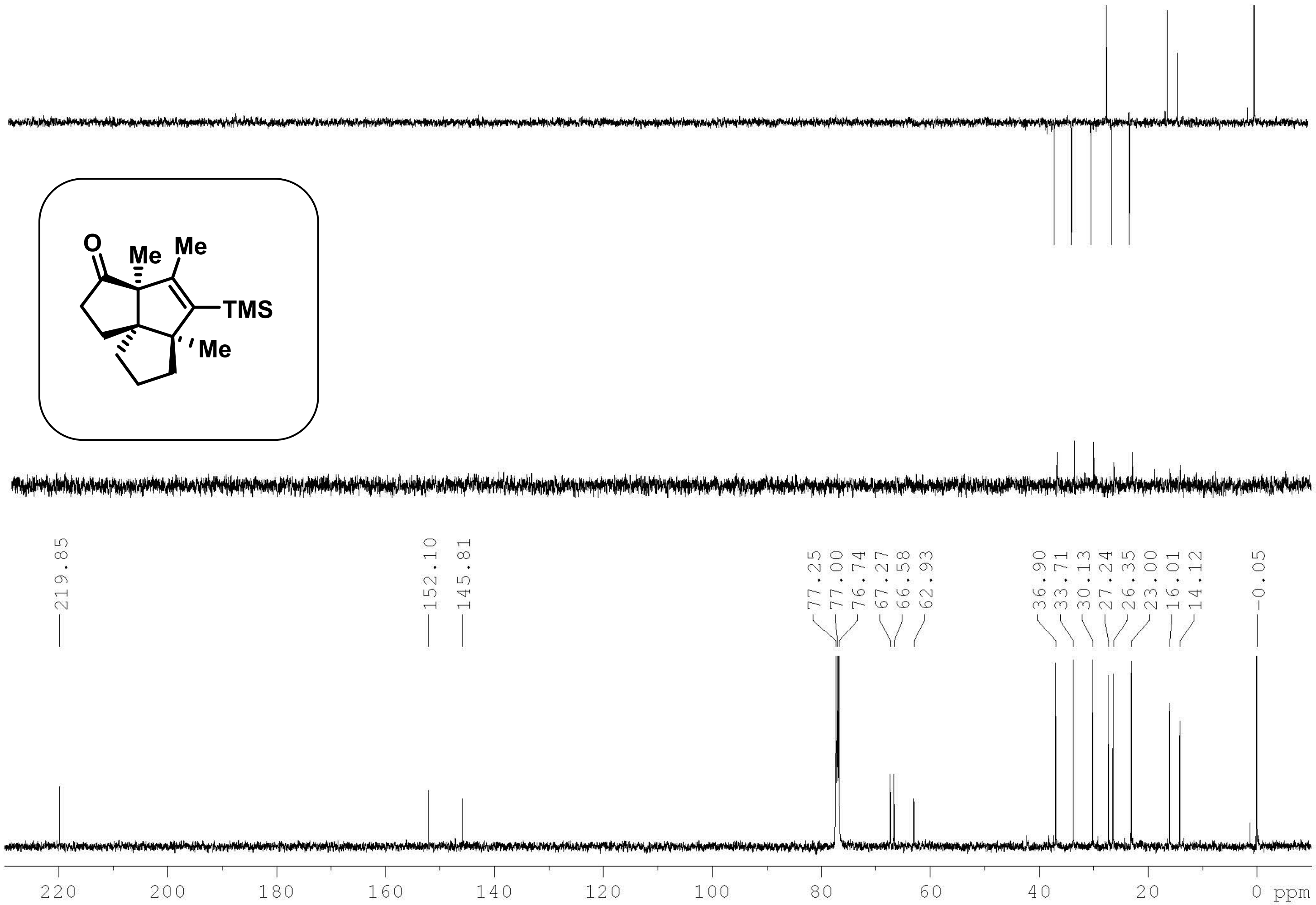

Figure 71. ^{13}C, DEPT-90, DEPT-135 NMR (125 MHz, $CDCl_3$) (**28m-*cis*,*trans***)

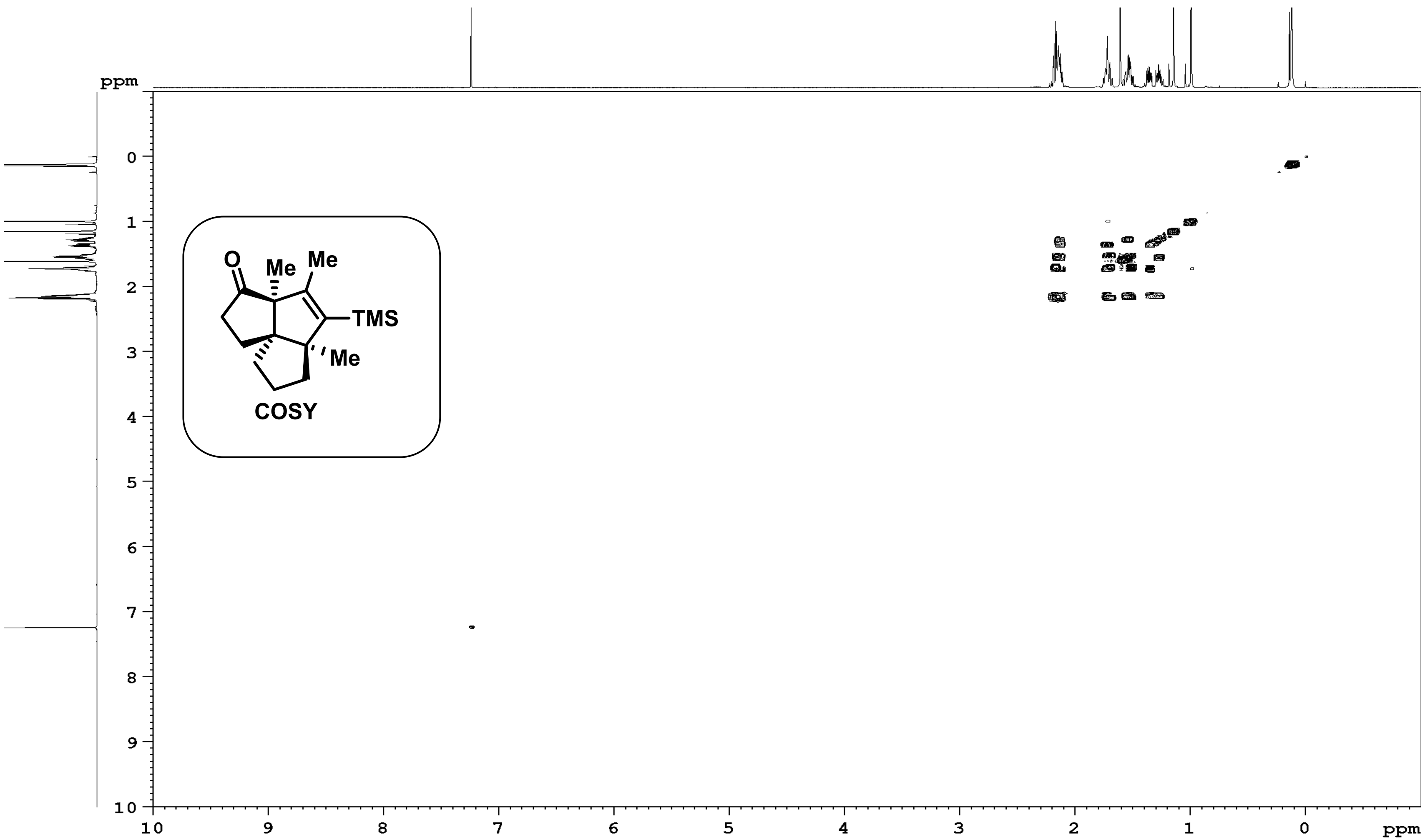

Figure 72. ^{1}H-^{1}H COSY NMR (500 MHz, $CDCl_3$) (**28m-*cis*,*trans***)

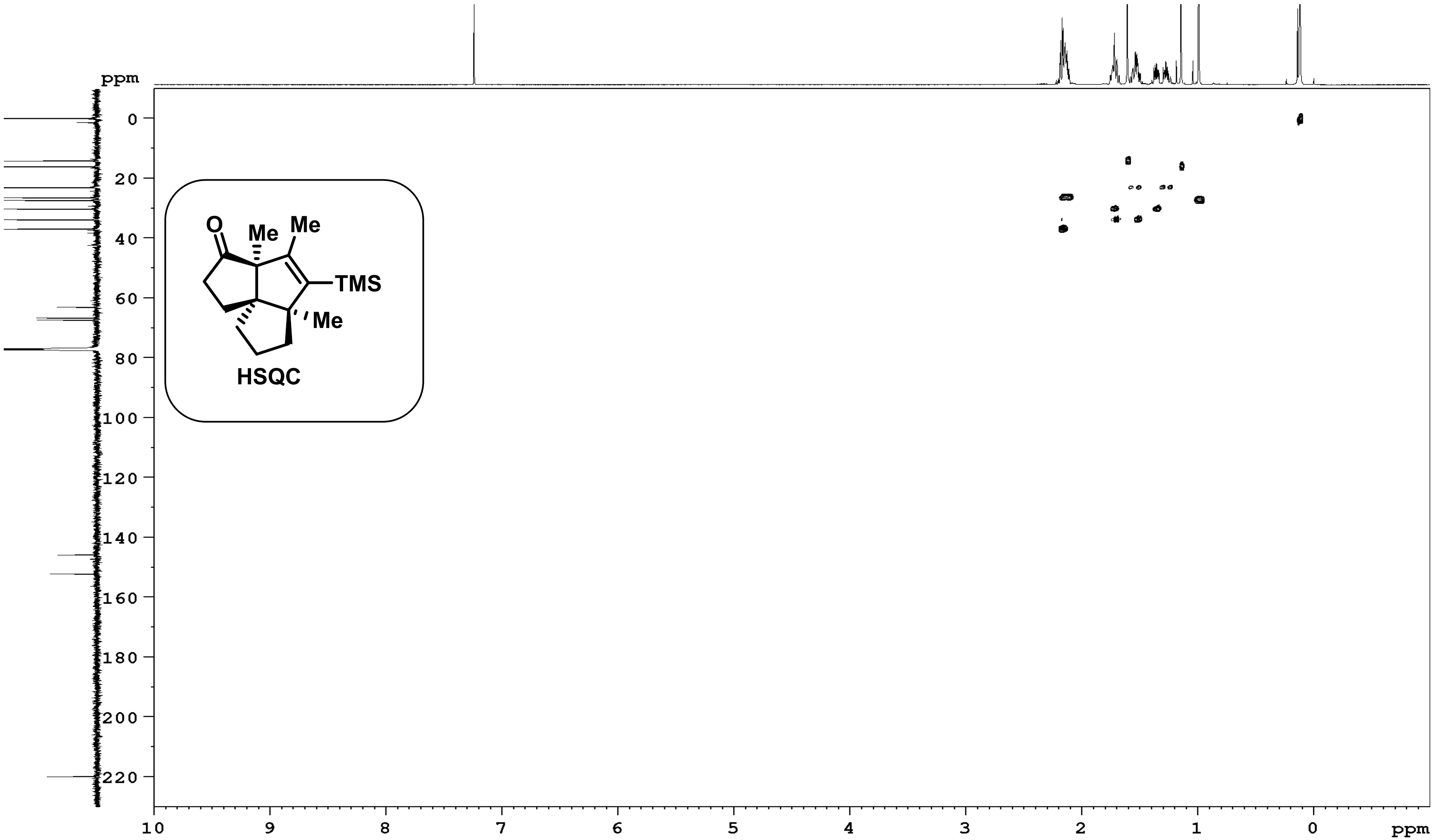

Figure 73. ^{1}H-^{13}C HSQC NMR (500 MHz, $CDCl_3$) (**28m-*cis,trans***)

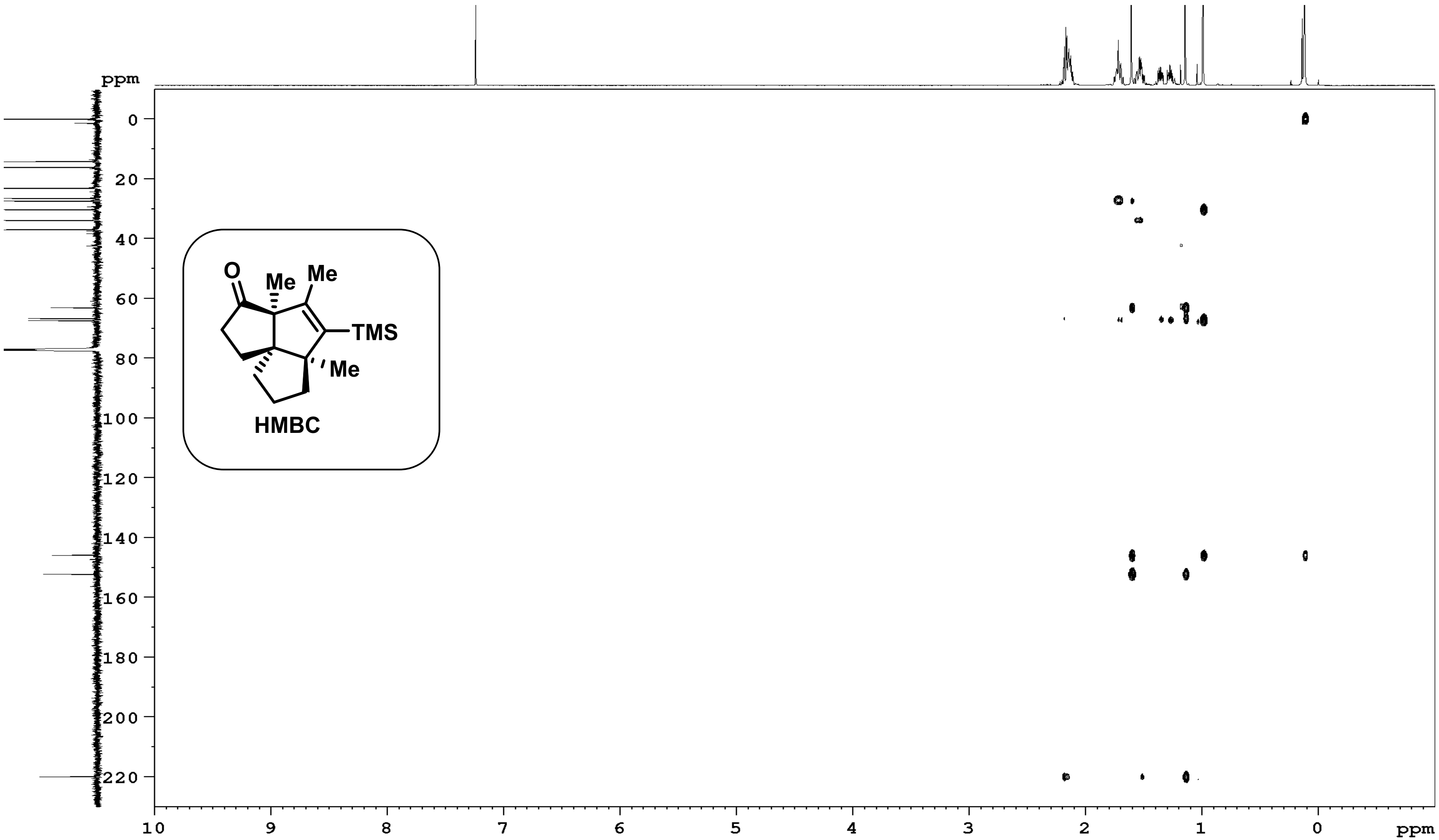

Figure 74. ^{1}H-^{13}C HMBC NMR (500 MHz, $CDCl_3$) (**28m-*cis,trans***)

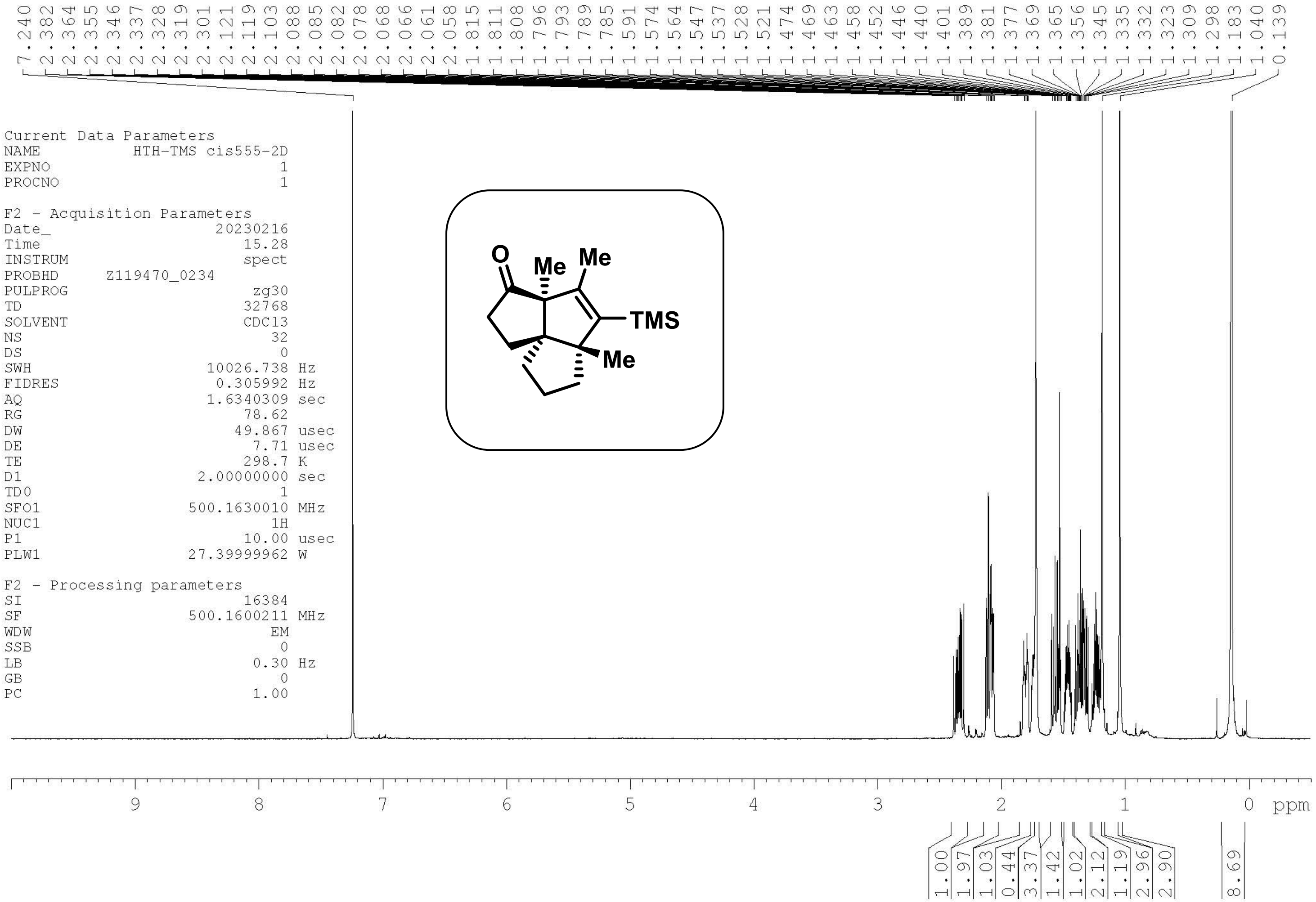

Figure 75. ^{1}H NMR (500 MHz, $CDCl_3$) (**28m-*cis,cis***)

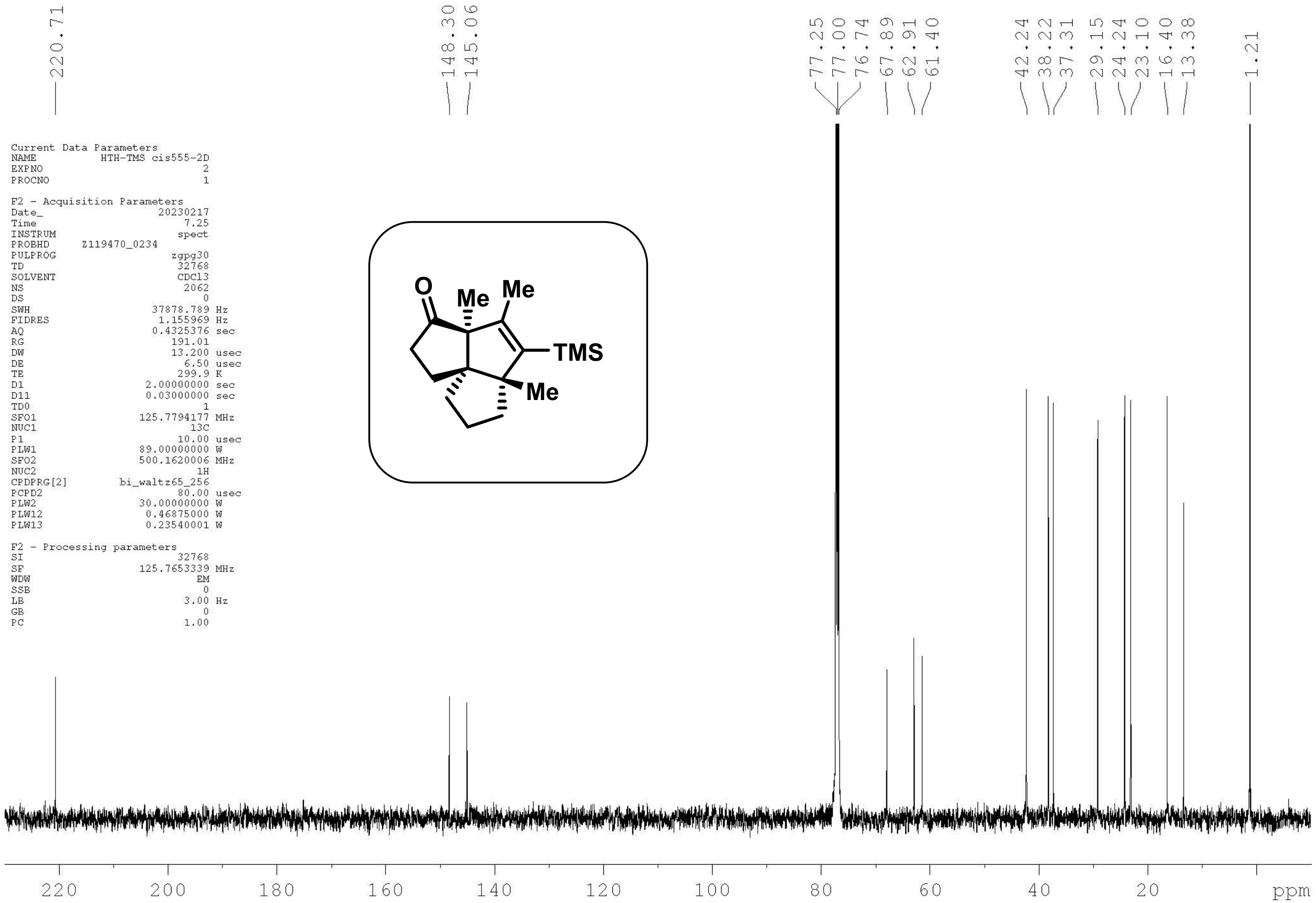

Figure 76. ^{13}C NMR (125 MHz, $CDCl_3$) (**28m-*cis,cis***)

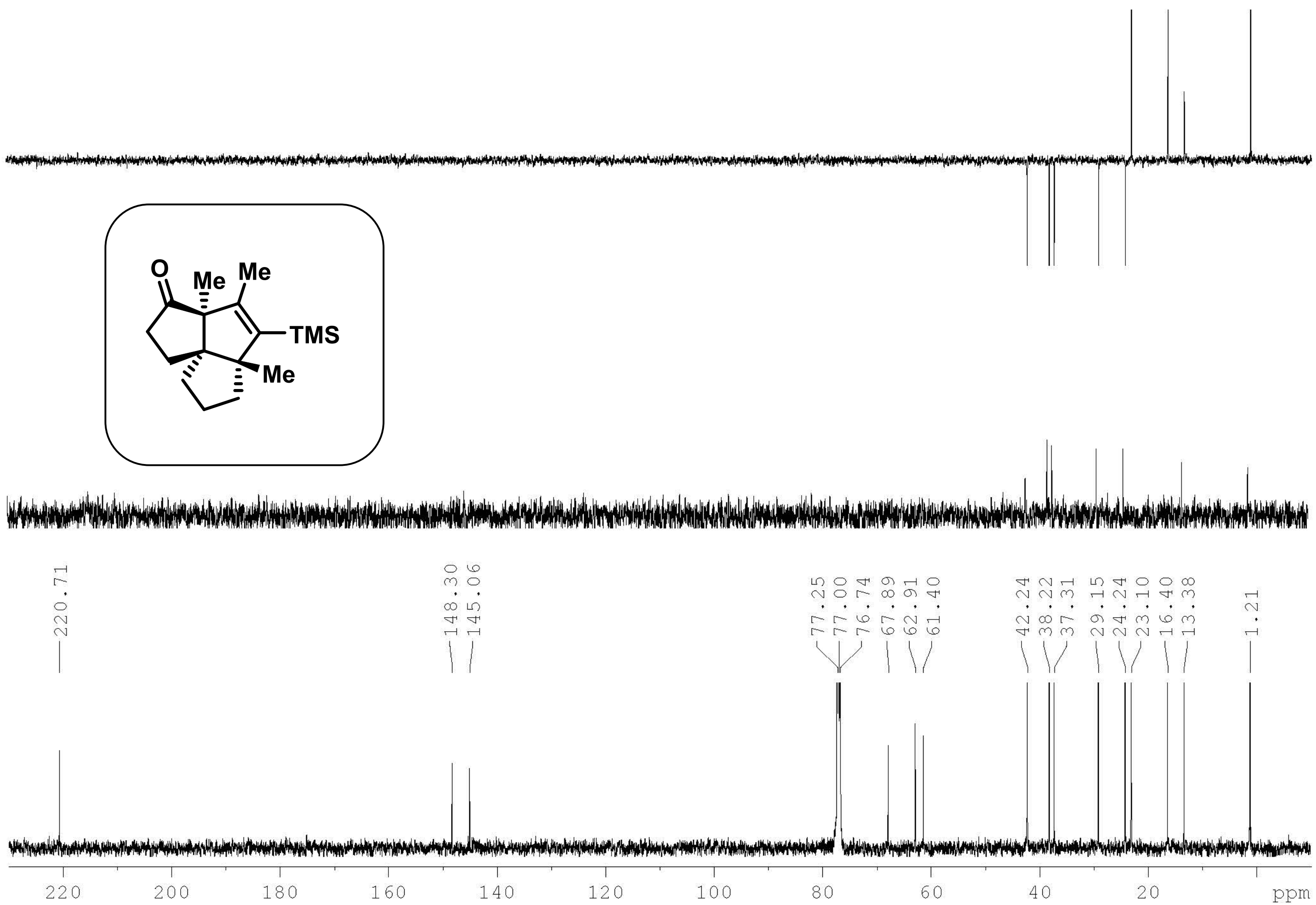

Figure 77. ^{13}C, DEPT-90, DEPT-135 NMR (125 MHz, $CDCl_3$) (**28m-*cis*,*cis***)

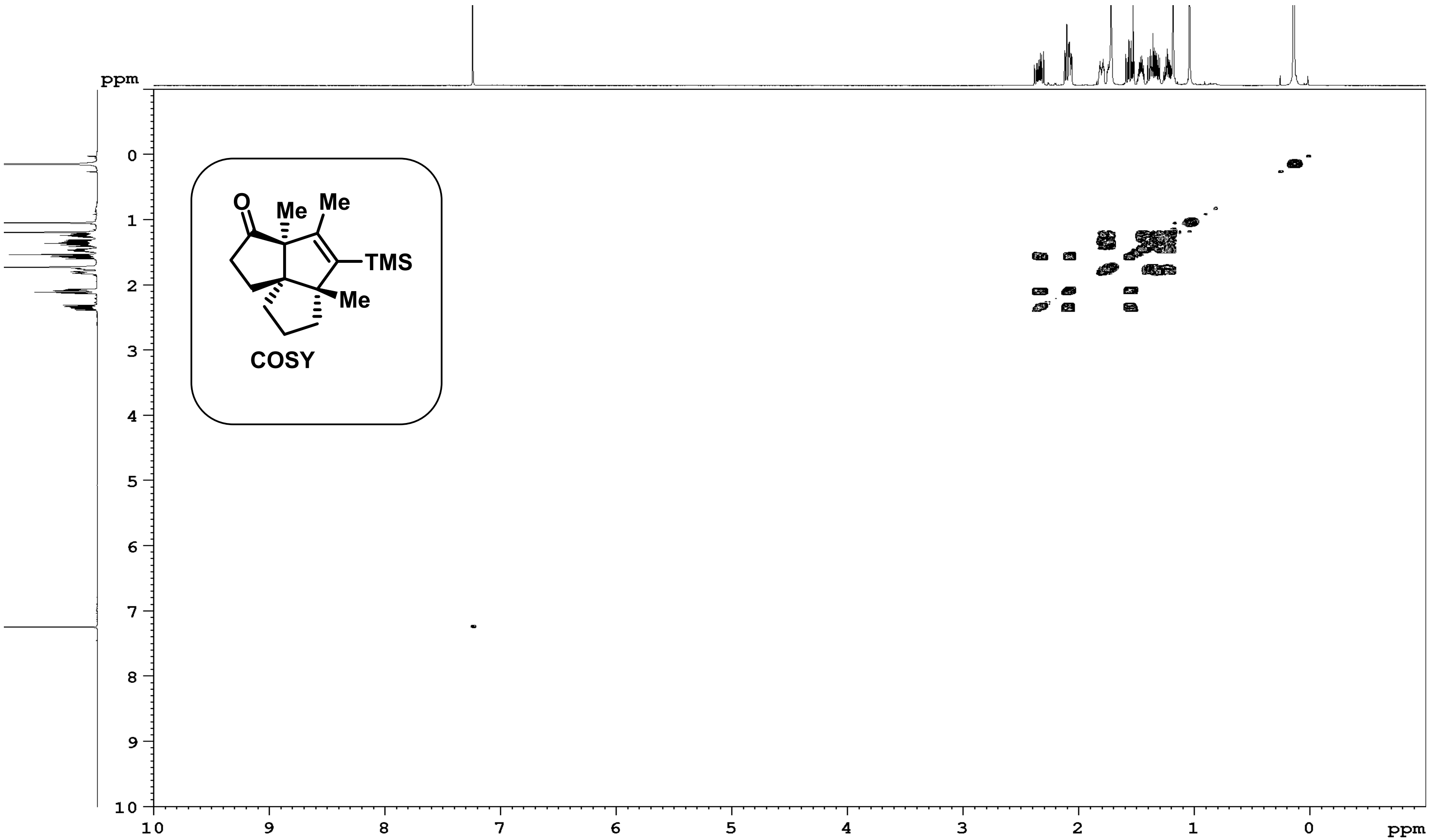

Figure 78. ^{1}H-^{1}H COSY NMR (500 MHz, $CDCl_3$) (**28m-*cis,cis***)

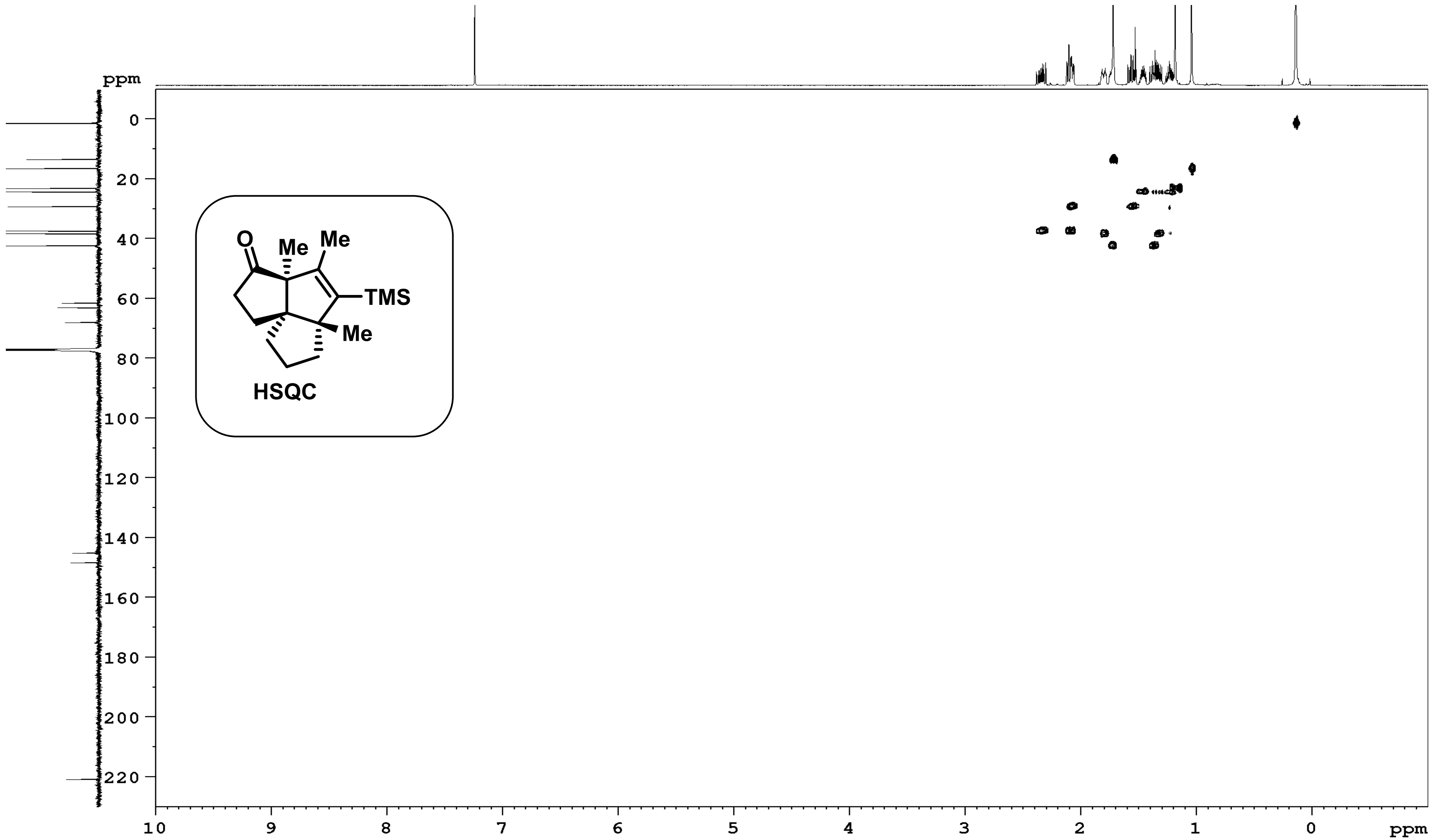

Figure 79. ^{1}H-^{13}C HSQC NMR (500 MHz, $CDCl_3$) (**28m-*cis,cis***)

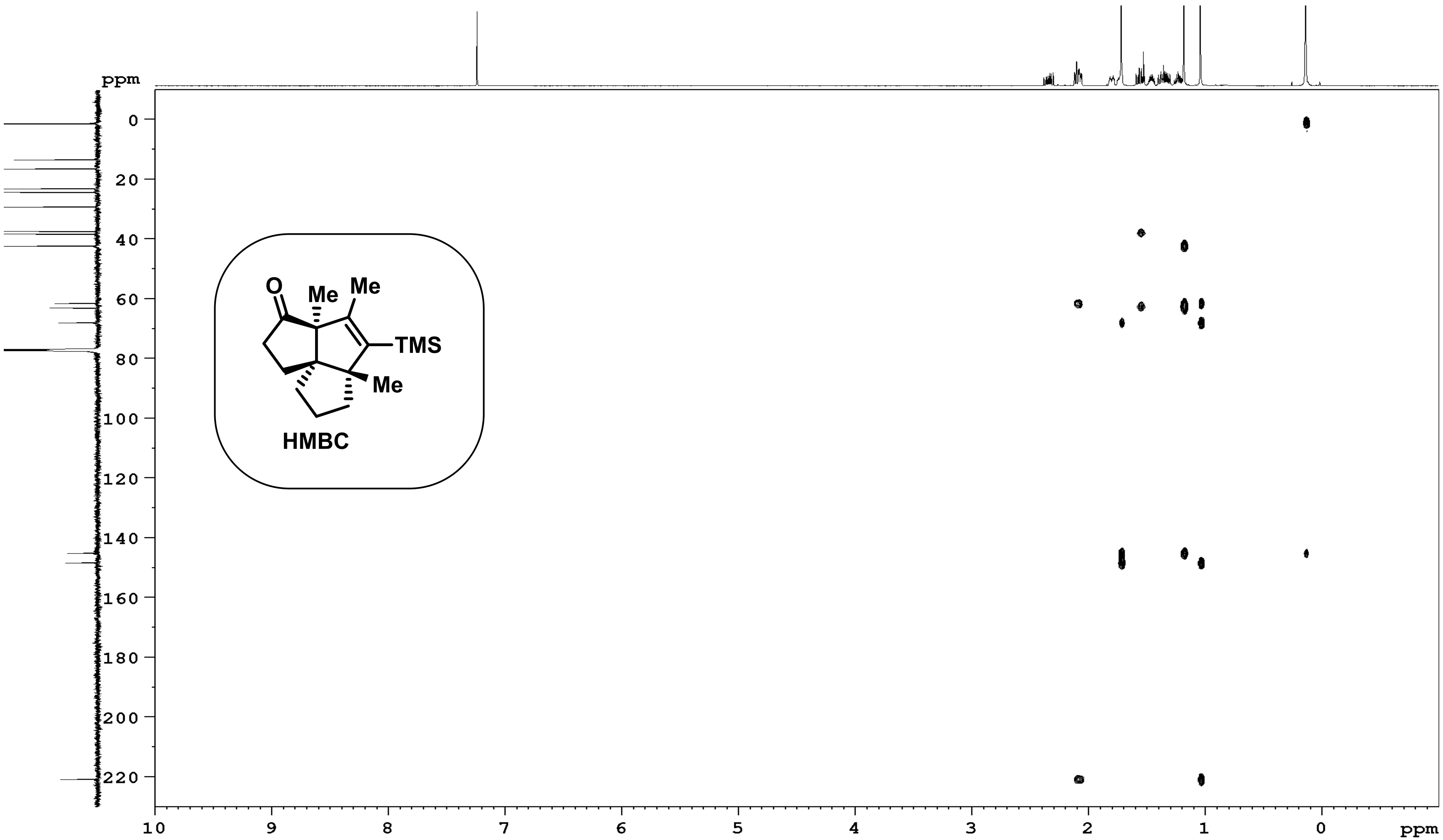

Figure 80. ^{1}H-^{13}C HMBC NMR (500 MHz, $CDCl_3$) (**28m-*cis,cis***)

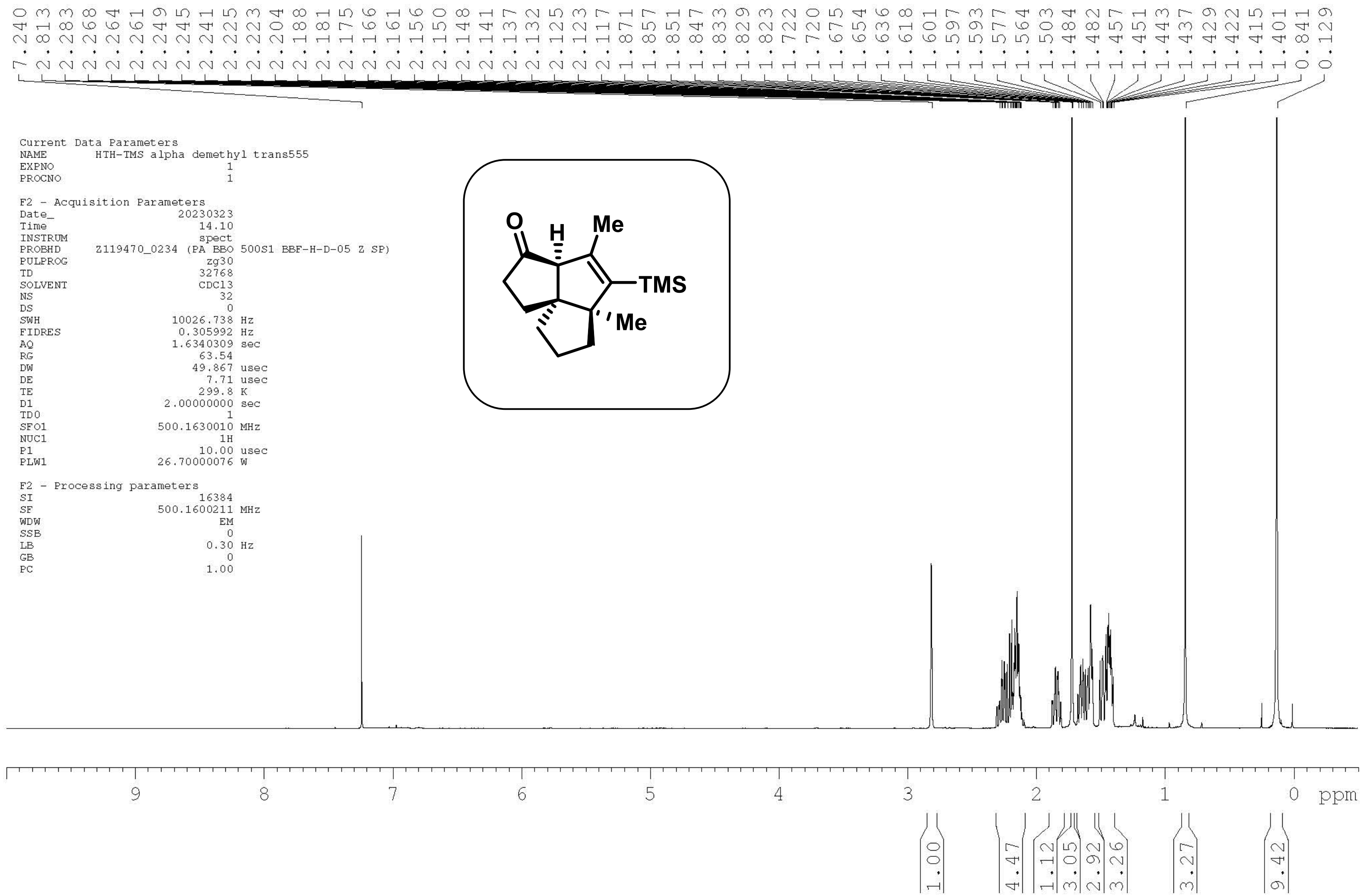

Figure 81. ^{1}H NMR (500 MHz, $CDCl_3$) (**28a-*cis,trans***)

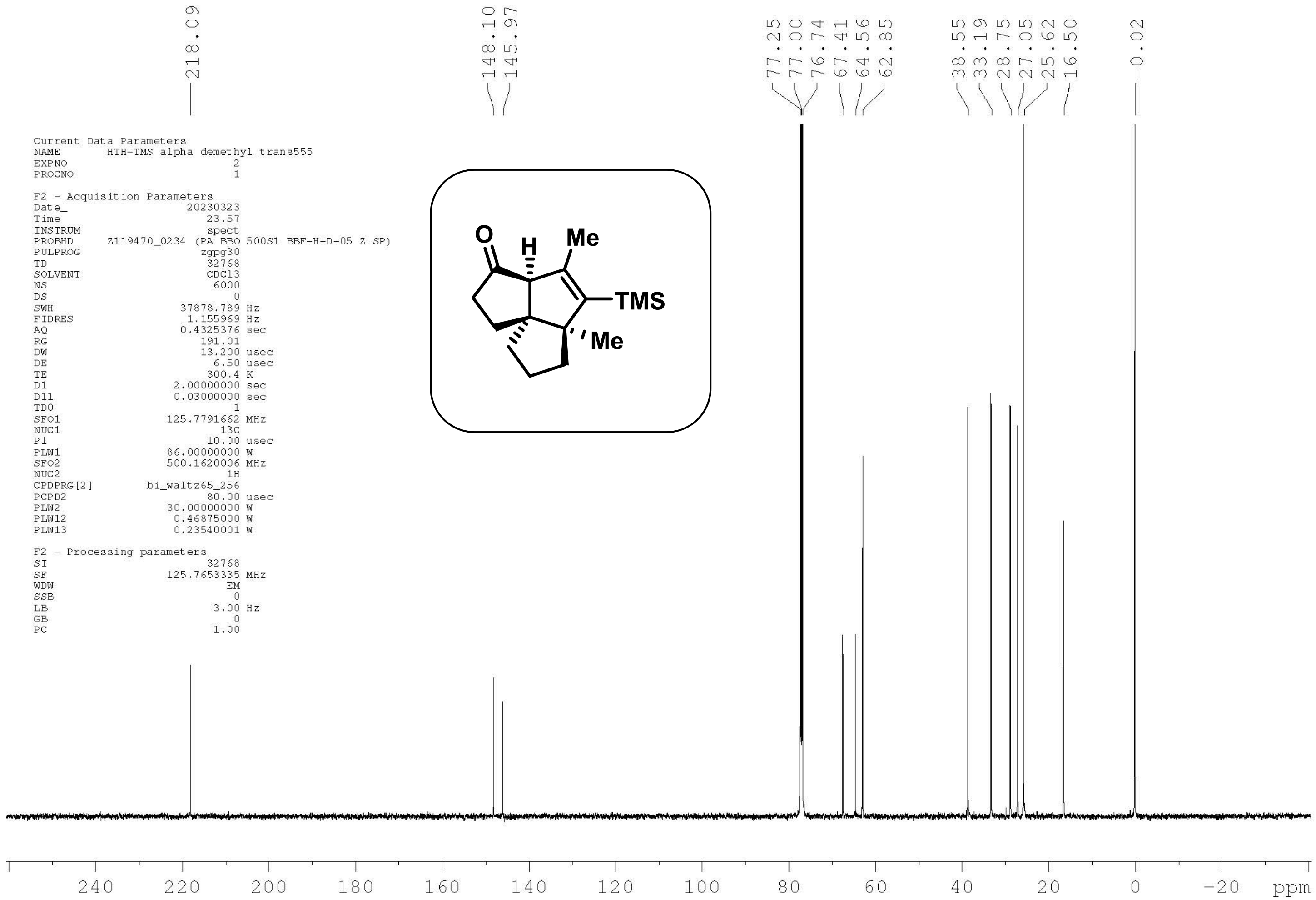

Figure 82. ^{13}C NMR (125 MHz, $CDCl_3$) (**28a-*cis,trans***)

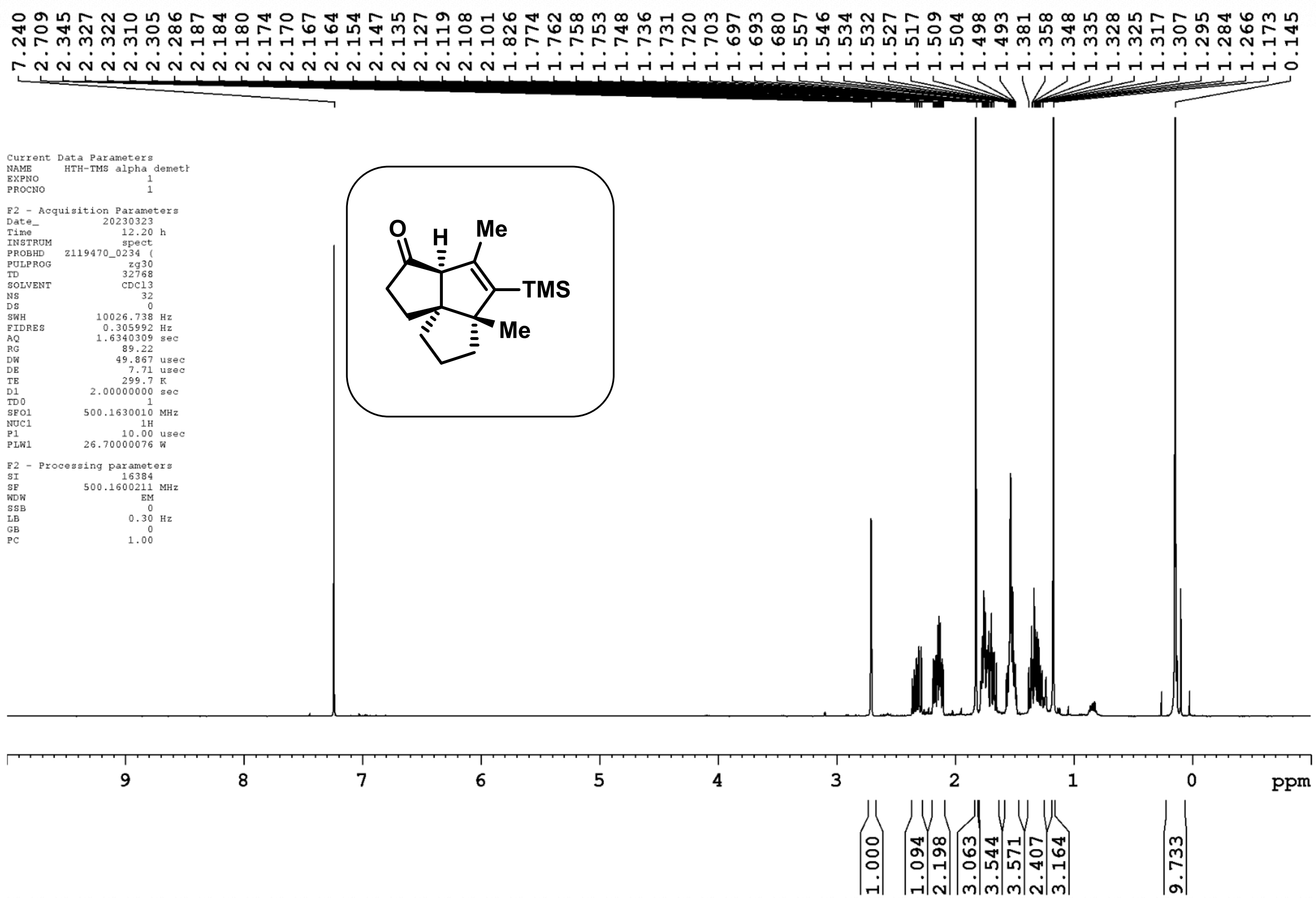

Figure 83. ^{1}H NMR (500 MHz, $CDCl_3$) (**28a-*cis,cis***)

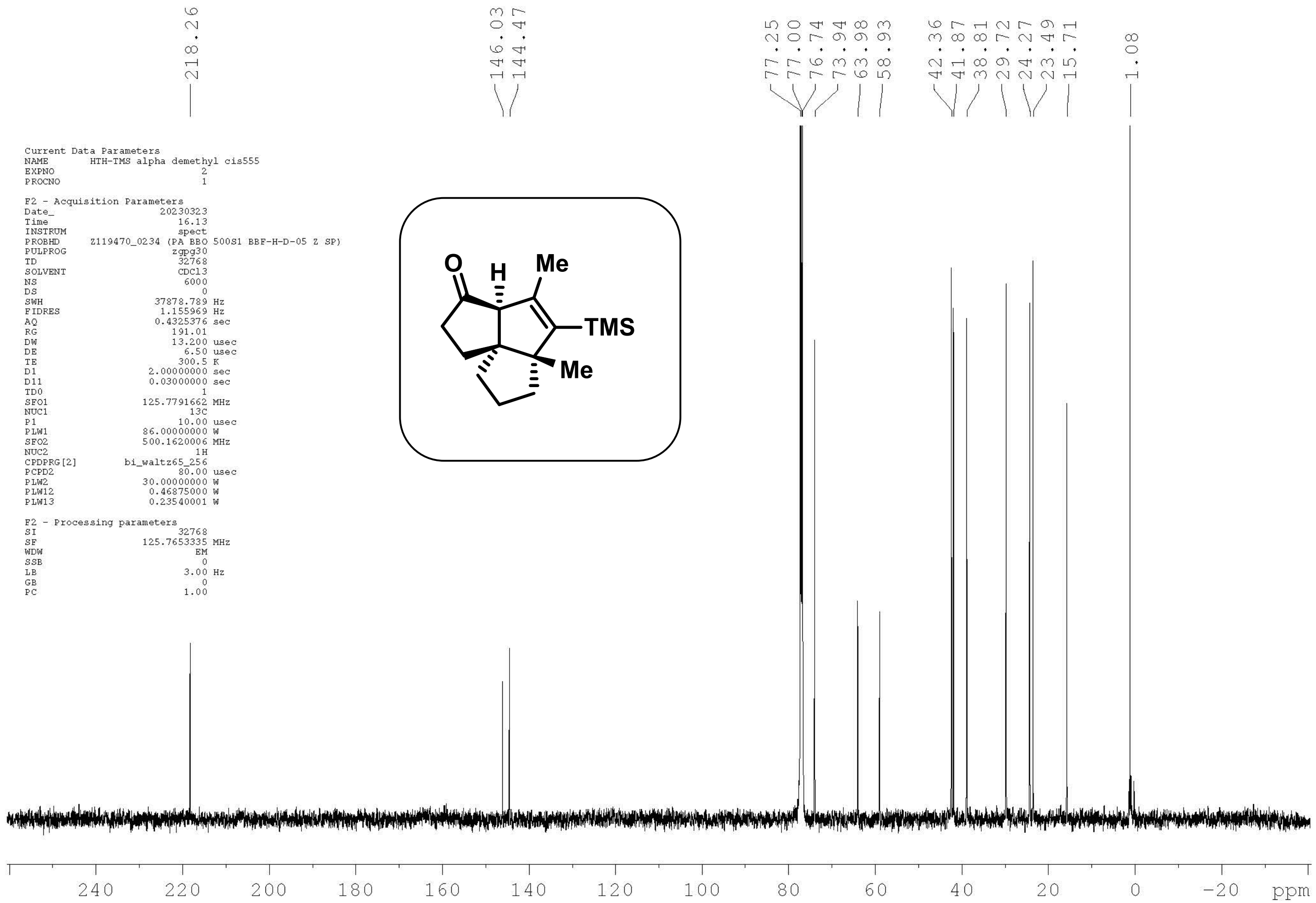

Figure 84. ^{13}C NMR (125 MHz, CDCl$_3$) (**28a-*cis,cis***)

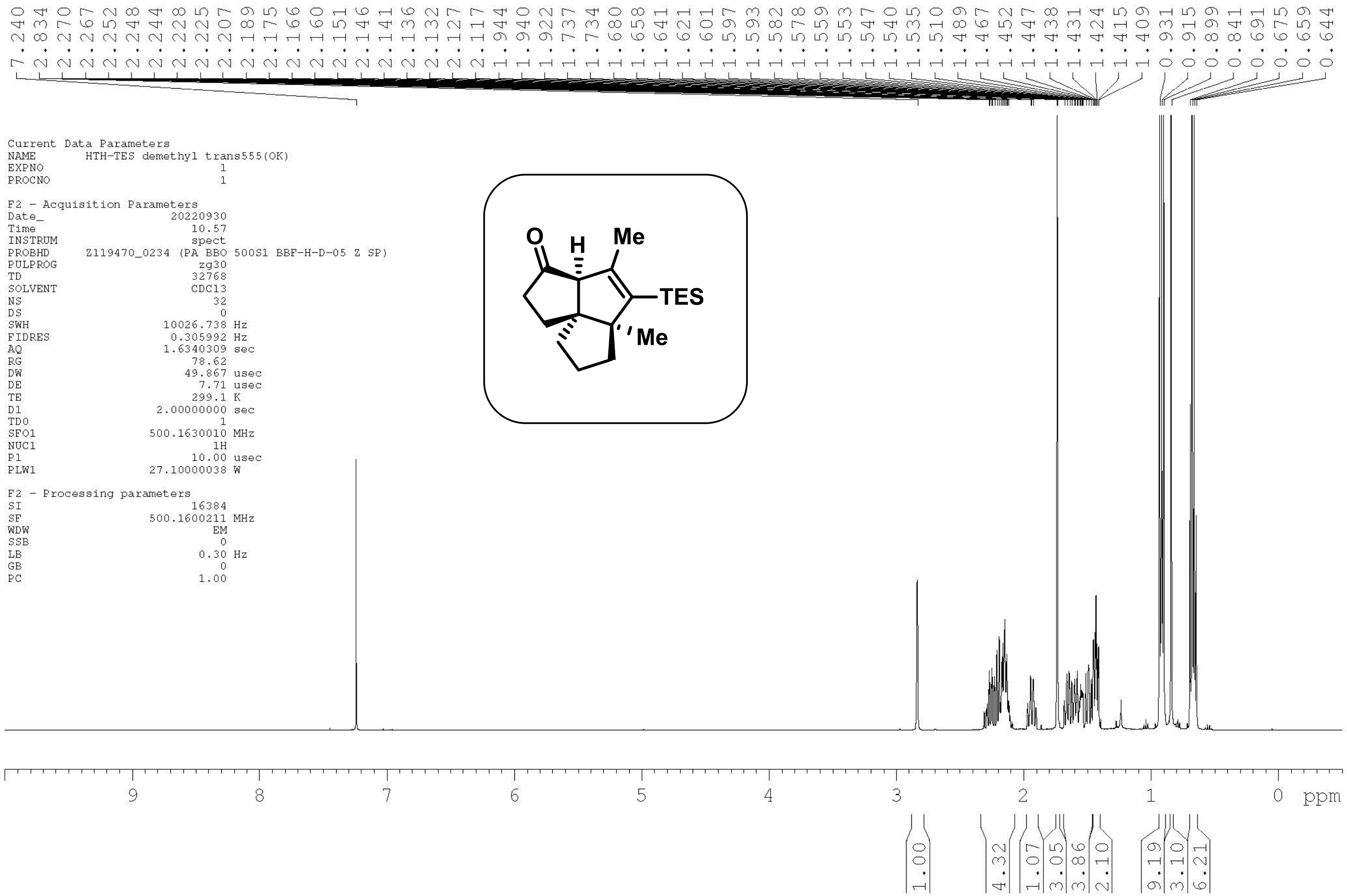

Figure 85. ^{1}H NMR (500 MHz, $CDCl_3$) (**28b-*cis,trans***)

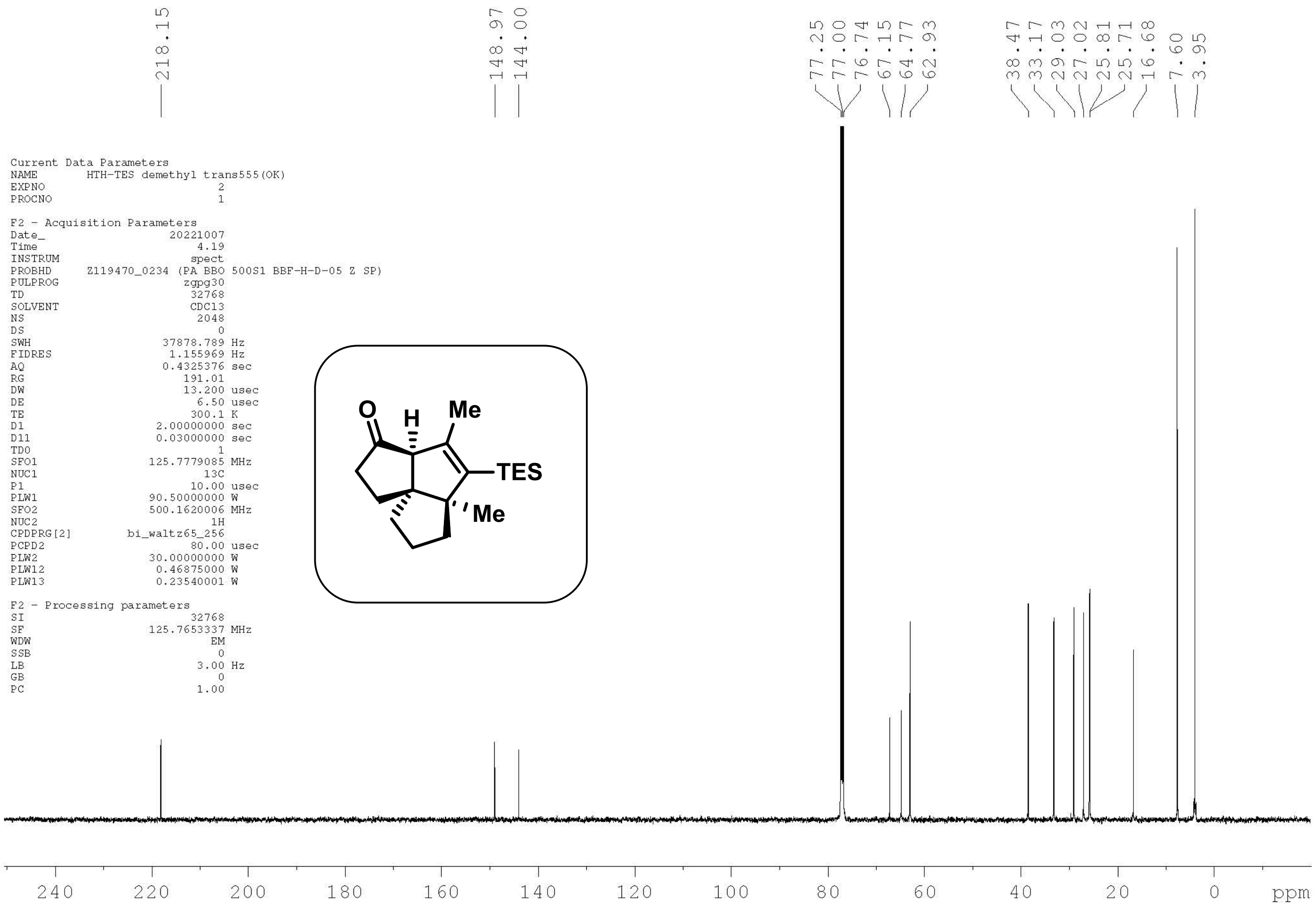

Figure 86. ^{13}C NMR (125 MHz, $CDCl_3$) (**28b-*cis,trans***)

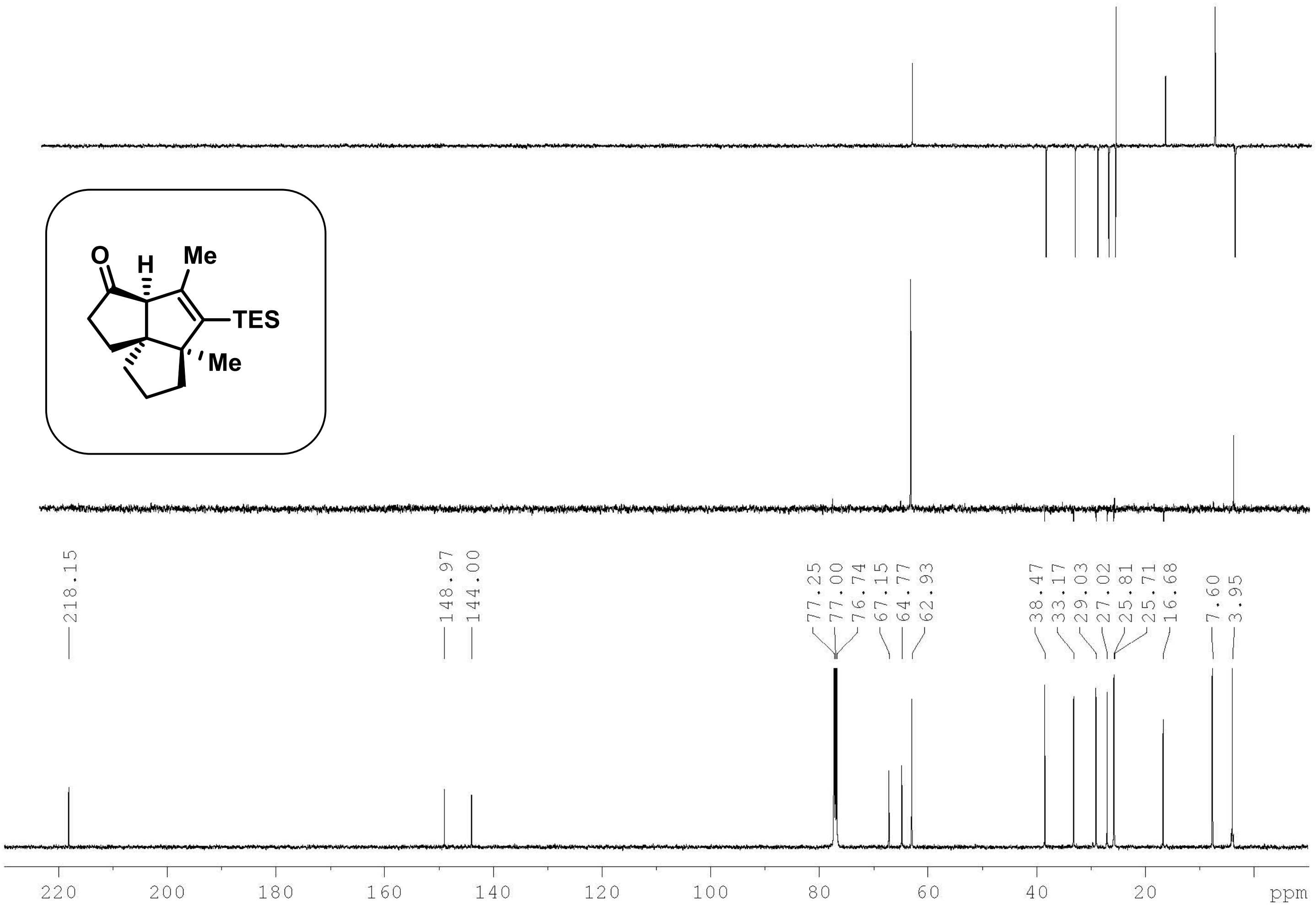

Figure 87. ^{13}C, DEPT-90, DEPT-135 NMR (125 MHz, $CDCl_3$) (**28b-*cis,trans***)

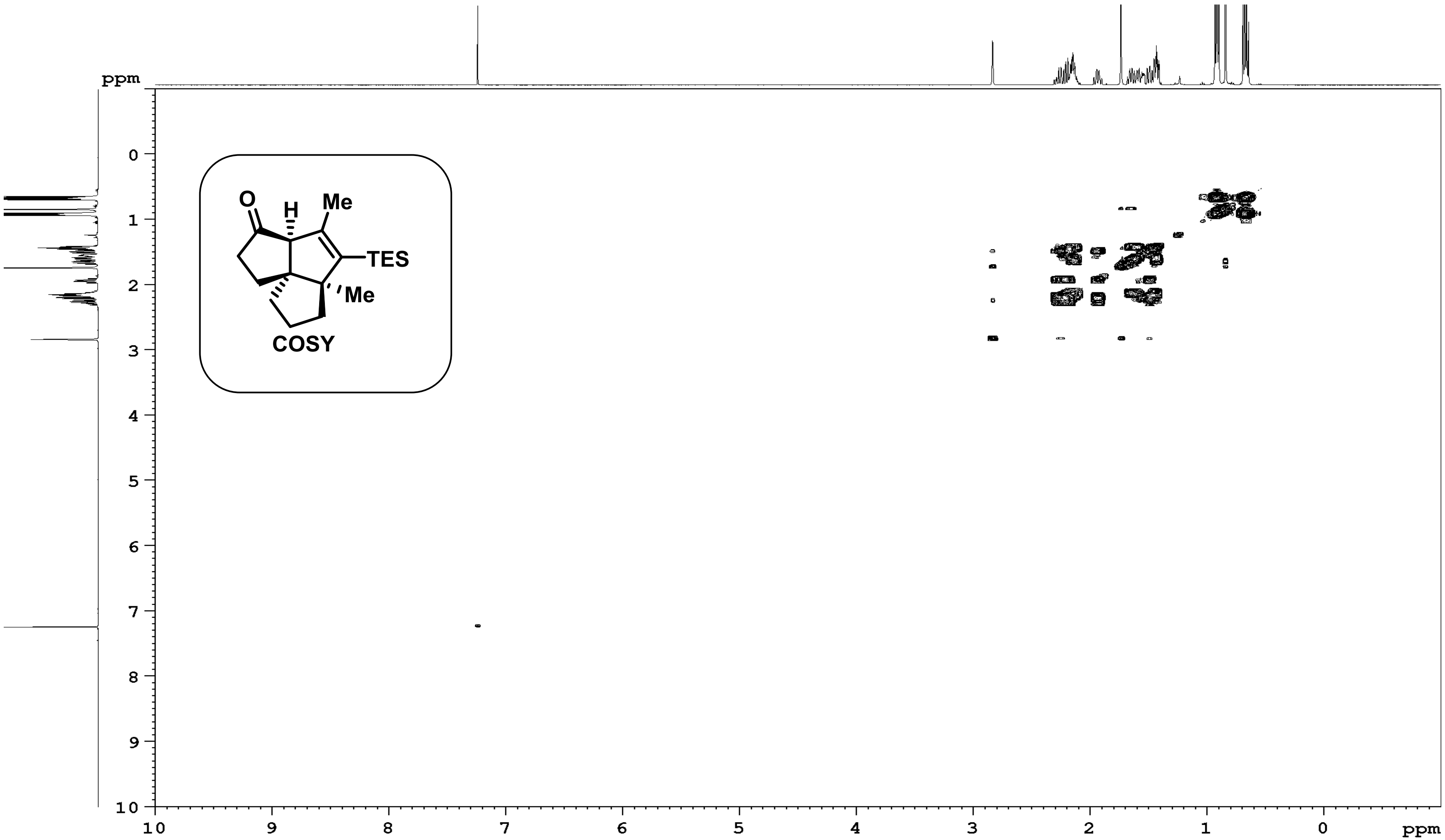

Figure 88. ^{1}H-^{1}H COSY NMR (500 MHz, $CDCl_3$) (**28b-*cis,trans***)

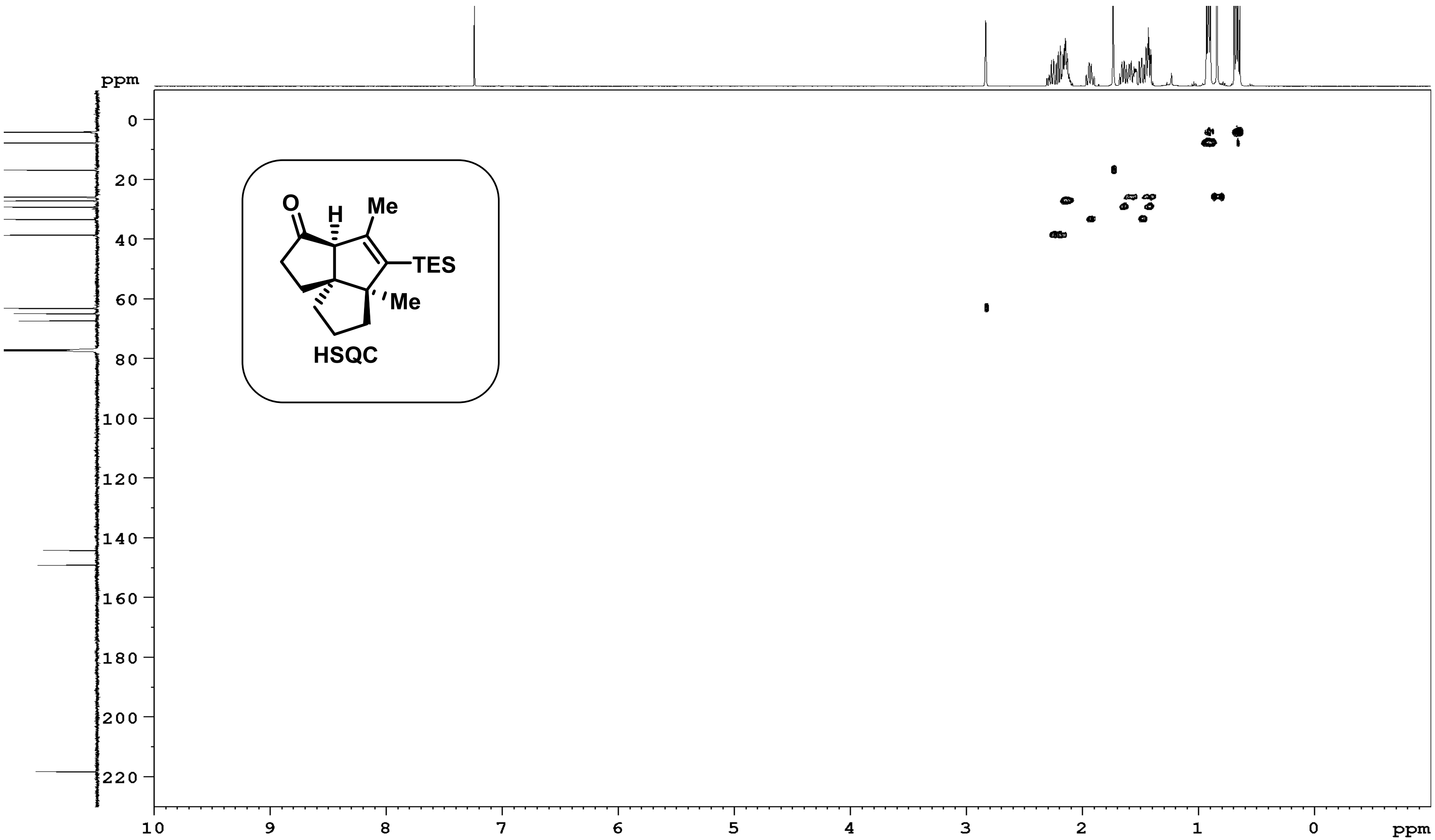

Figure 89. ^{1}H-^{13}C HSQC NMR (500 MHz, $CDCl_3$) (**28b-*cis*,*trans***)

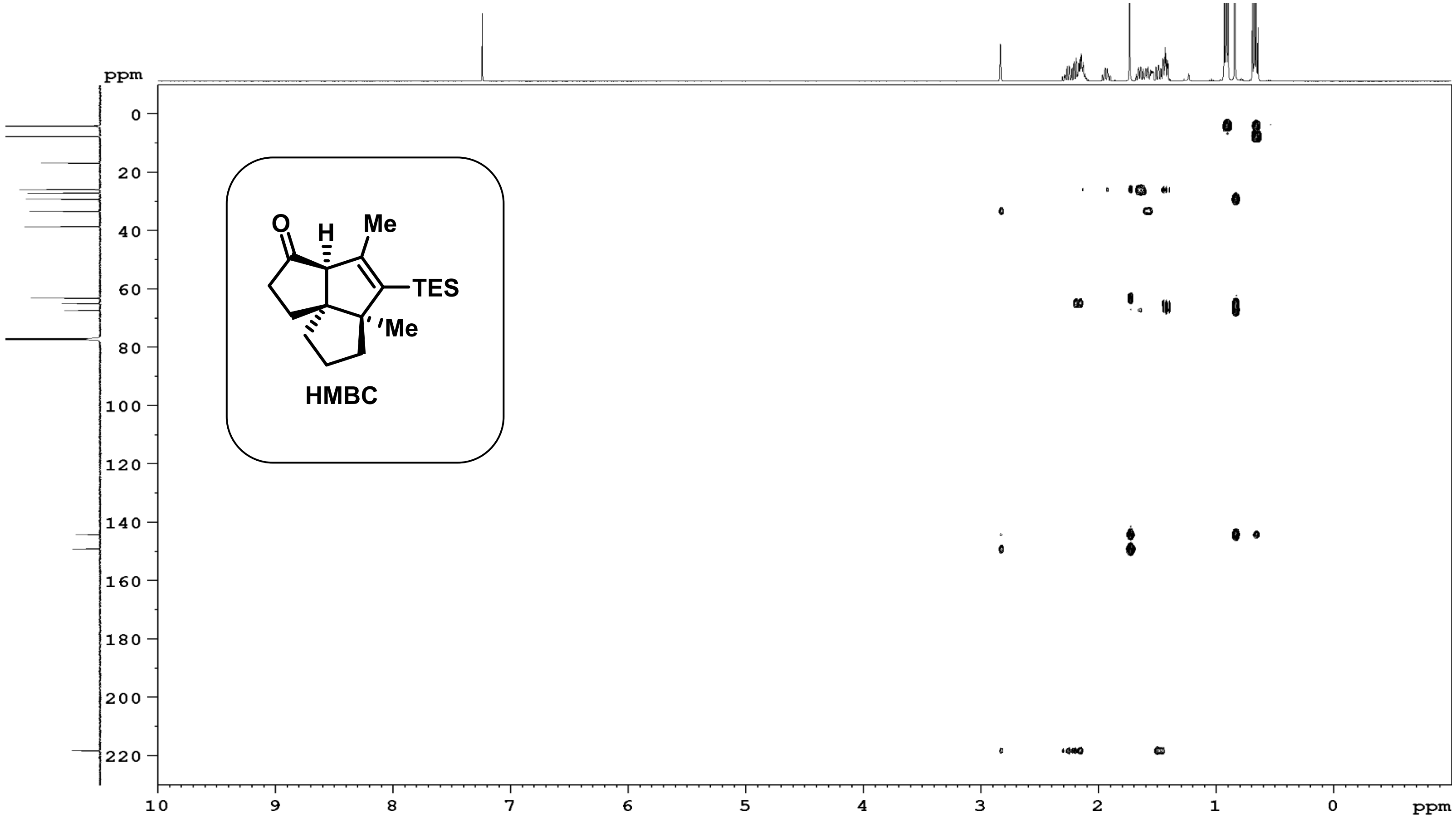

Figure 90. ^{1}H-^{13}C HMBC NMR (500 MHz, $CDCl_3$) (**28b-*cis*,*trans***)

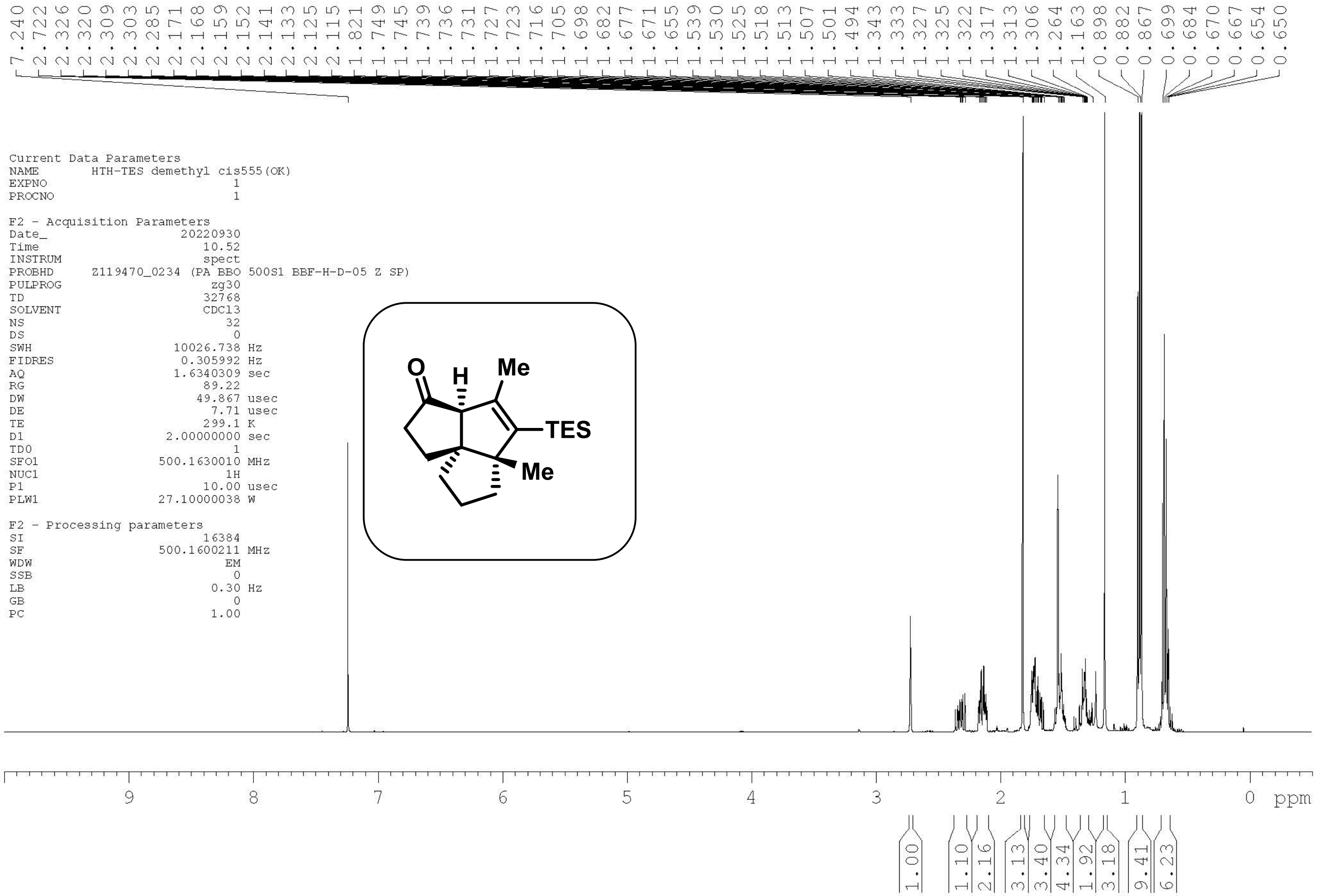

Figure 91. ^{1}H NMR (500 MHz, $CDCl_3$) (**28b-*cis,cis***)

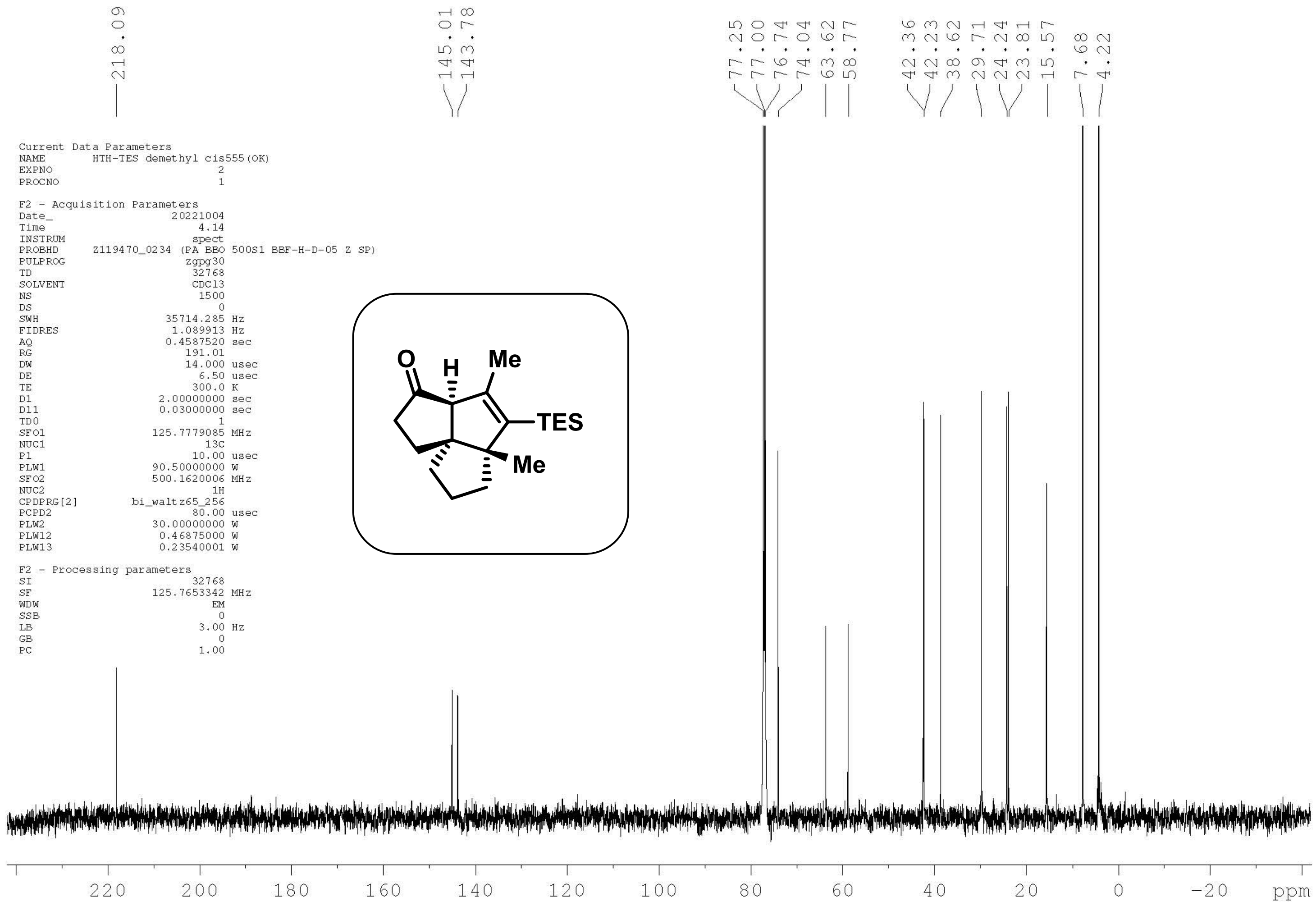

Figure 92. ^{13}C NMR (125 MHz, $CDCl_3$) (**28b-*cis,cis***)

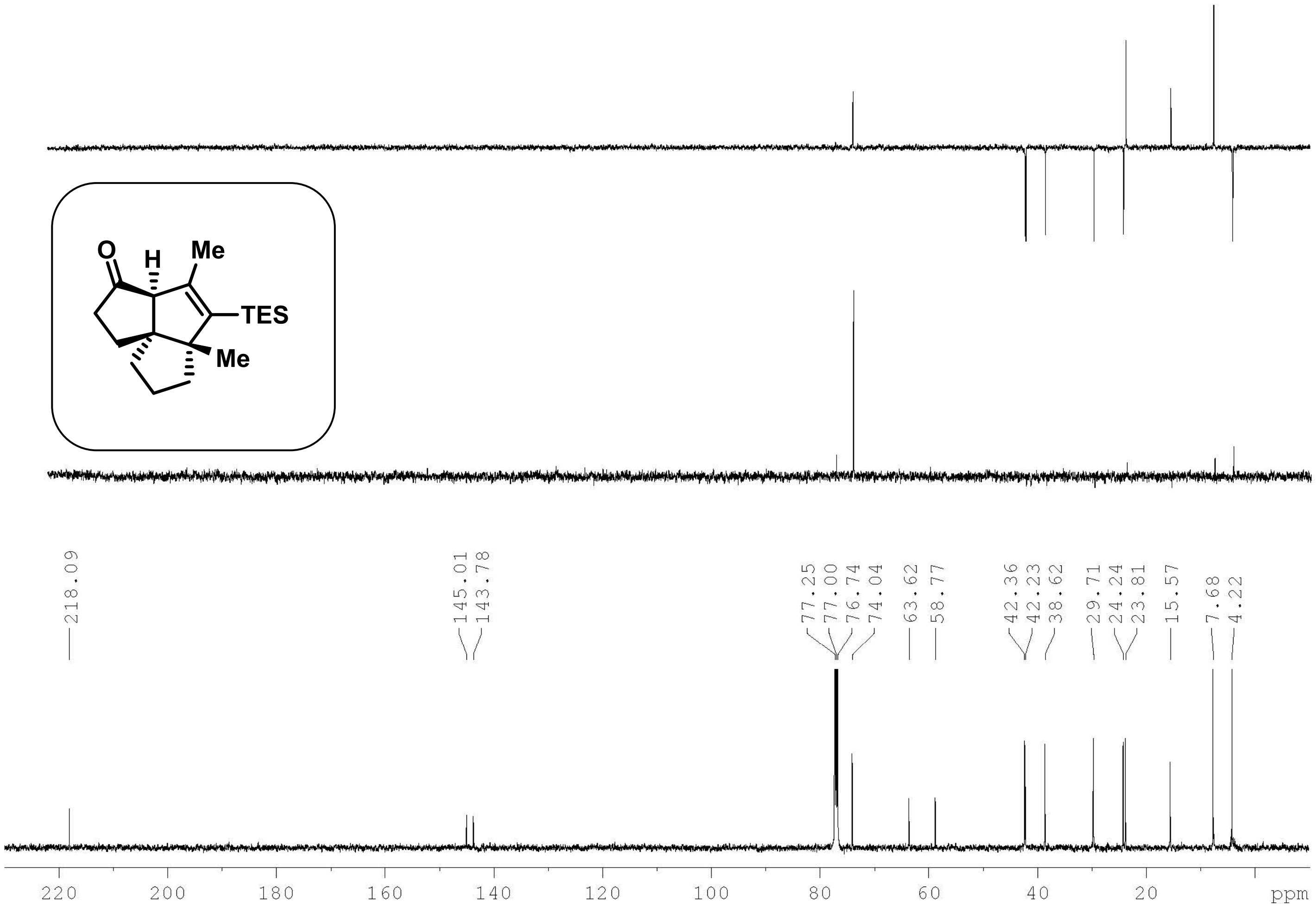

Figure 93. ^{13}C, DEPT-90, DEPT-135 NMR (125 MHz, $CDCl_3$) (**28b-*cis,cis***)

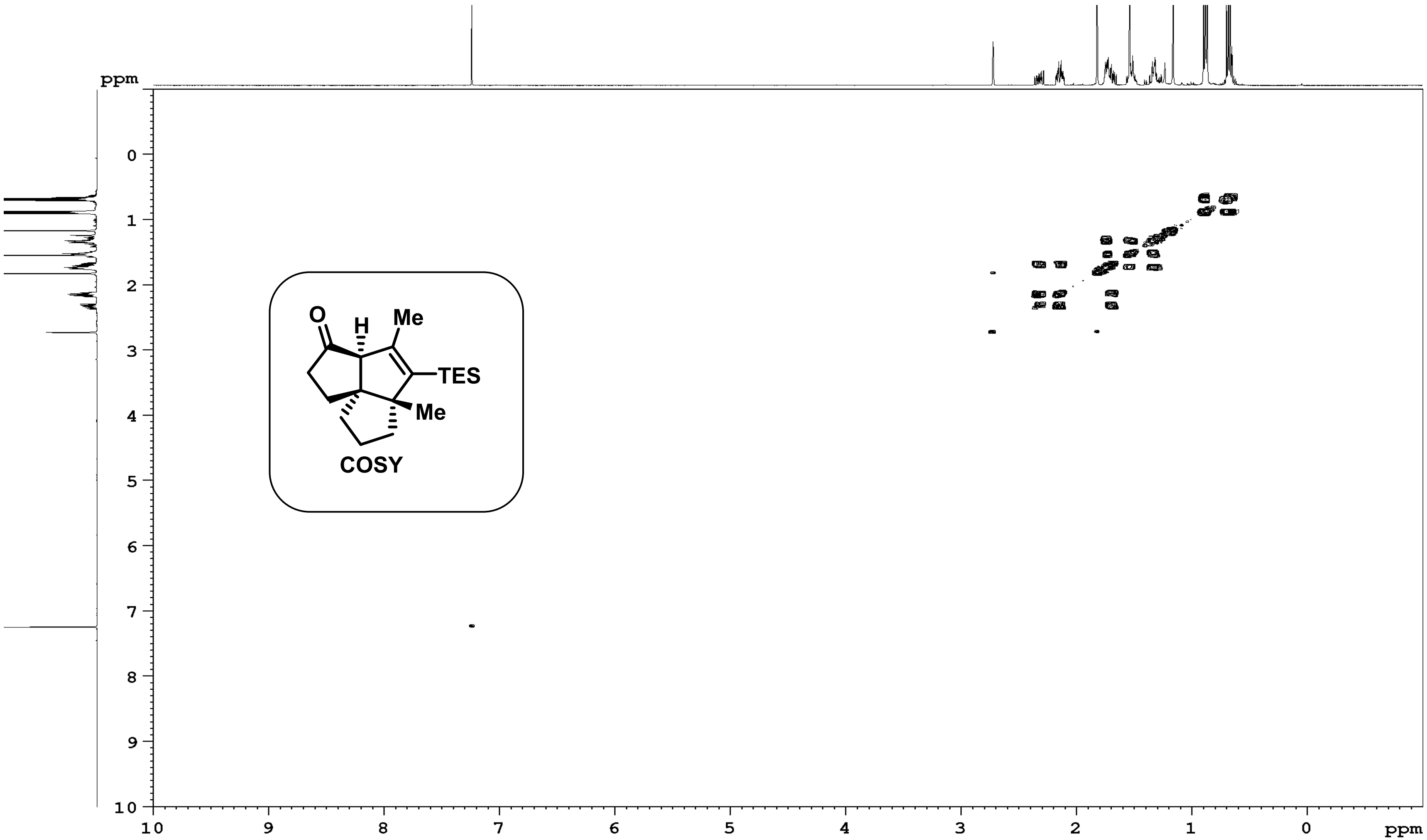

Figure 94. ^1H-^1H COSY NMR (500 MHz, $CDCl_3$) (**28b-*cis*,*cis***)

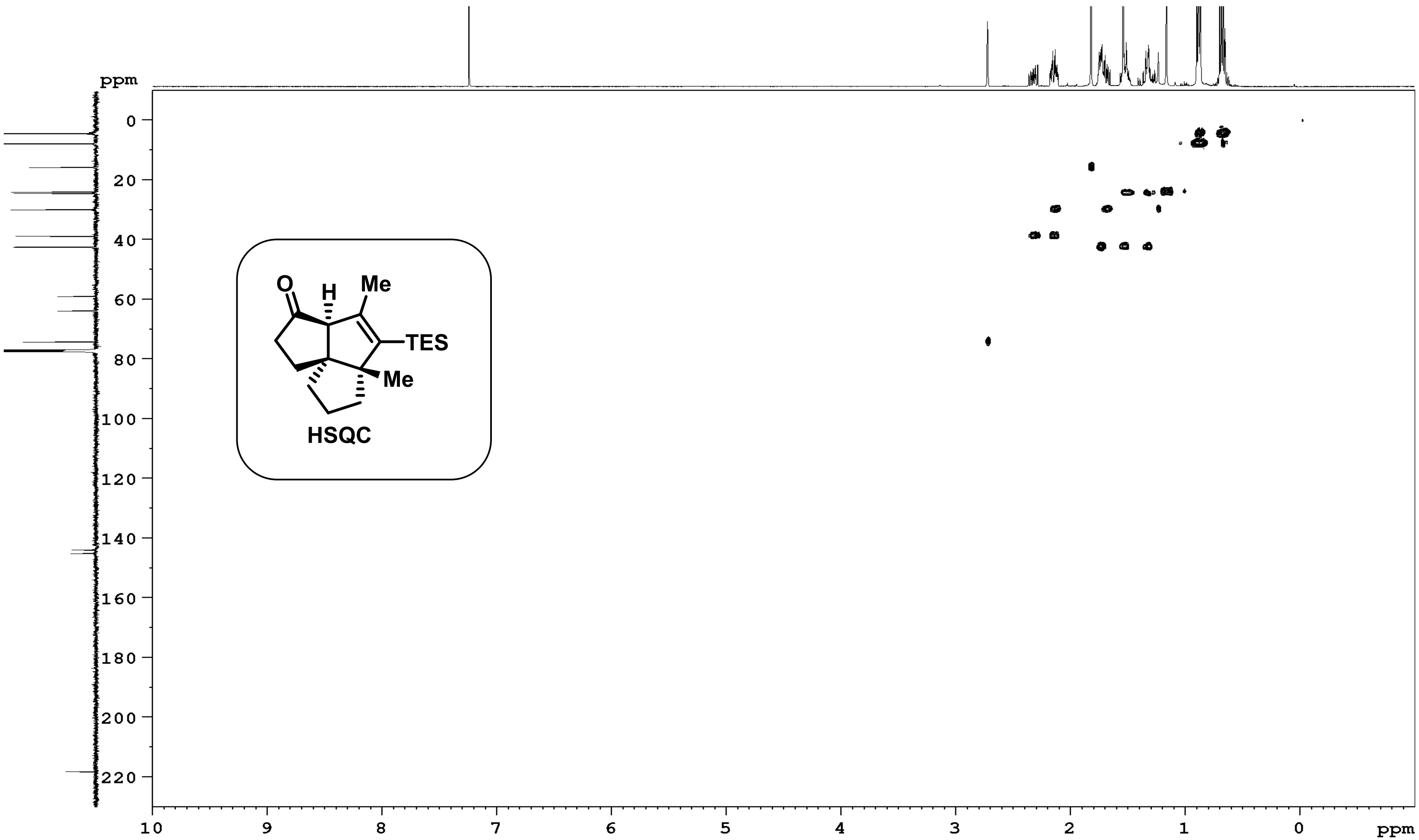

Figure 95. ^{1}H-^{13}C HSQC NMR (500 MHz, $CDCl_3$) (**28b-*cis,cis***)

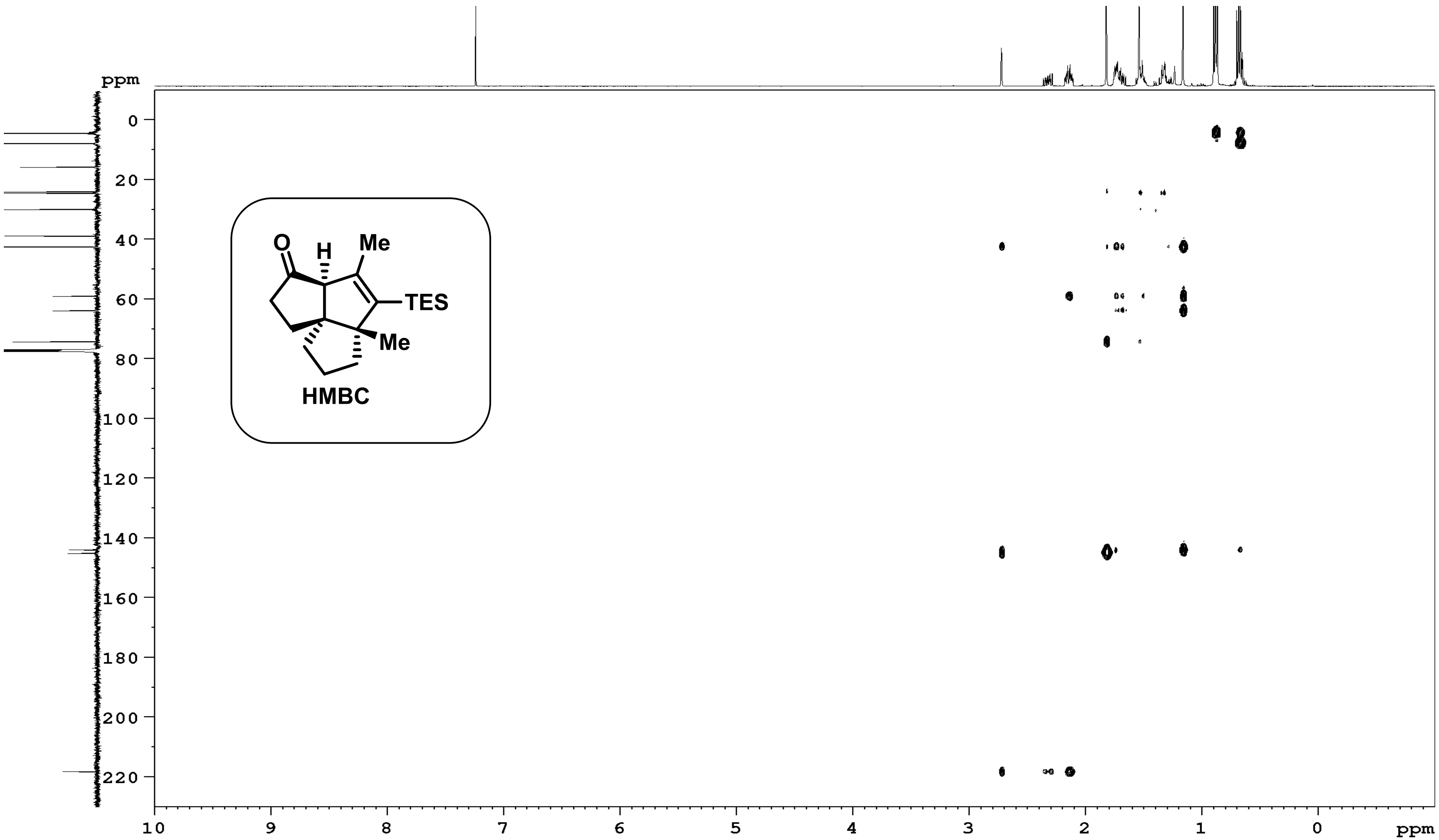

Figure 96. ^{1}H-^{13}C HMBC NMR (500 MHz, $CDCl_3$) (**28b-*cis,cis***)

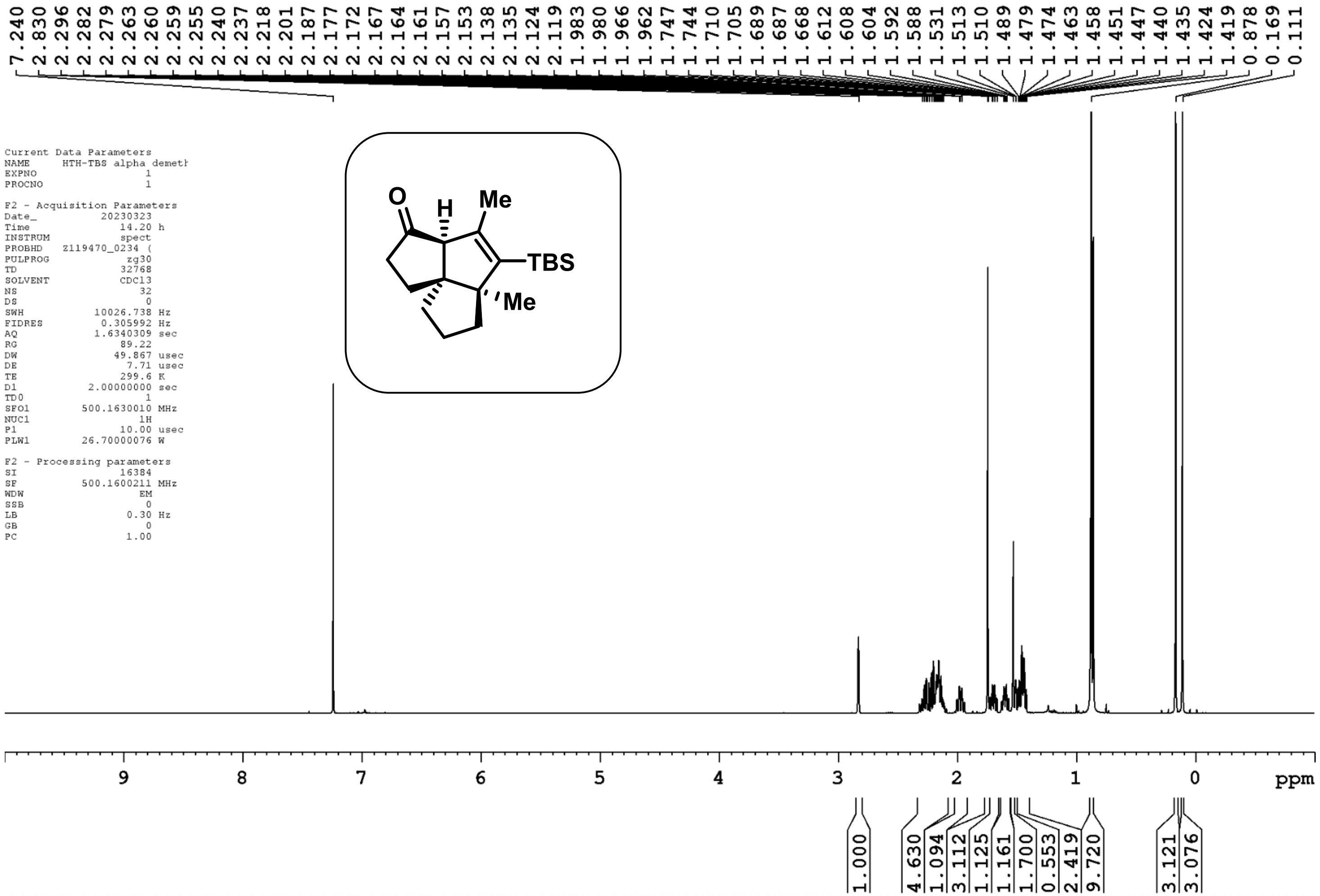

Figure 97. ^{1}H NMR (500 MHz, $CDCl_3$) (**28c-*cis*,*trans***)

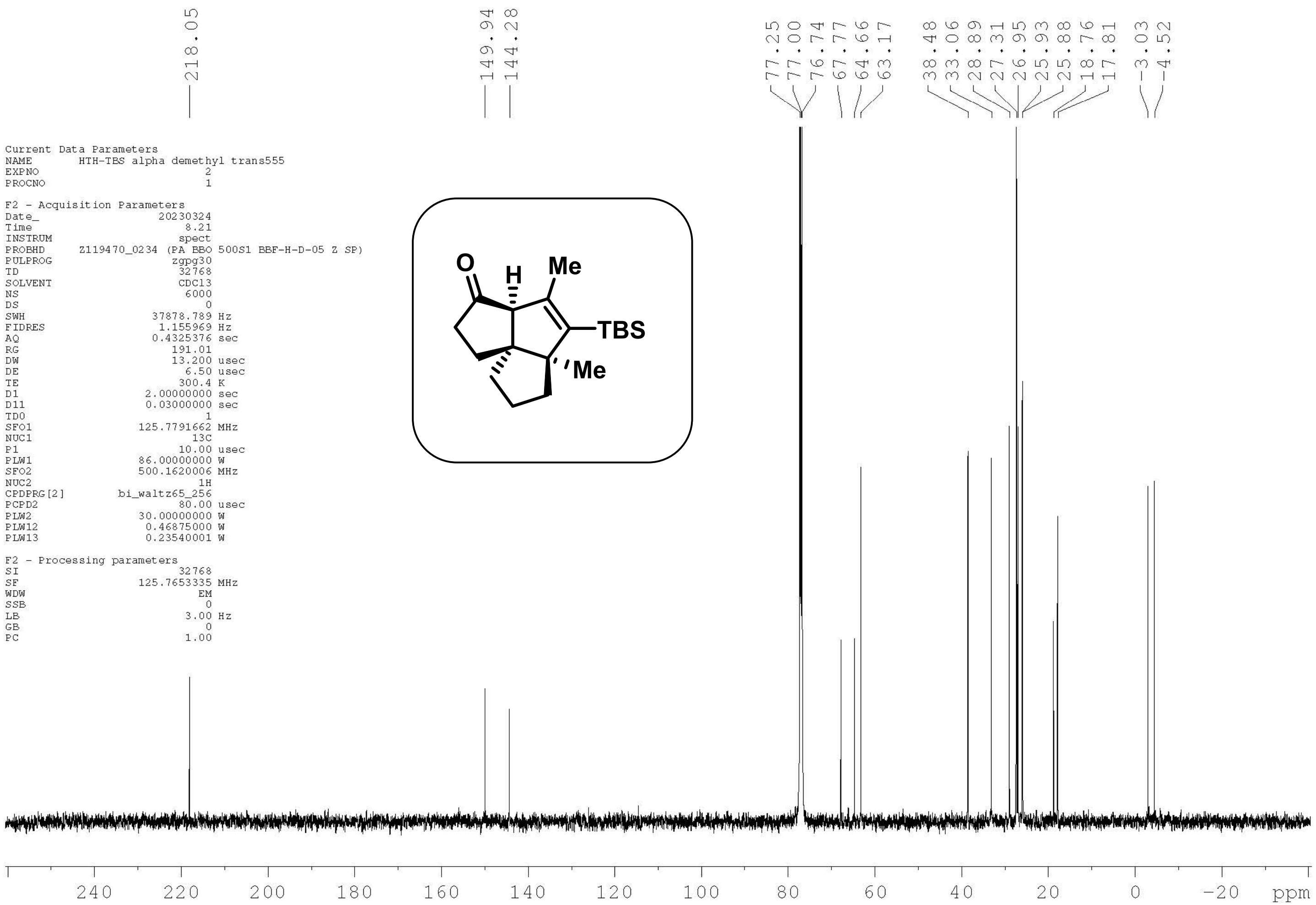

Figure 98. ^{13}C NMR (125 MHz, $CDCl_3$) (**28c-*cis,trans***)

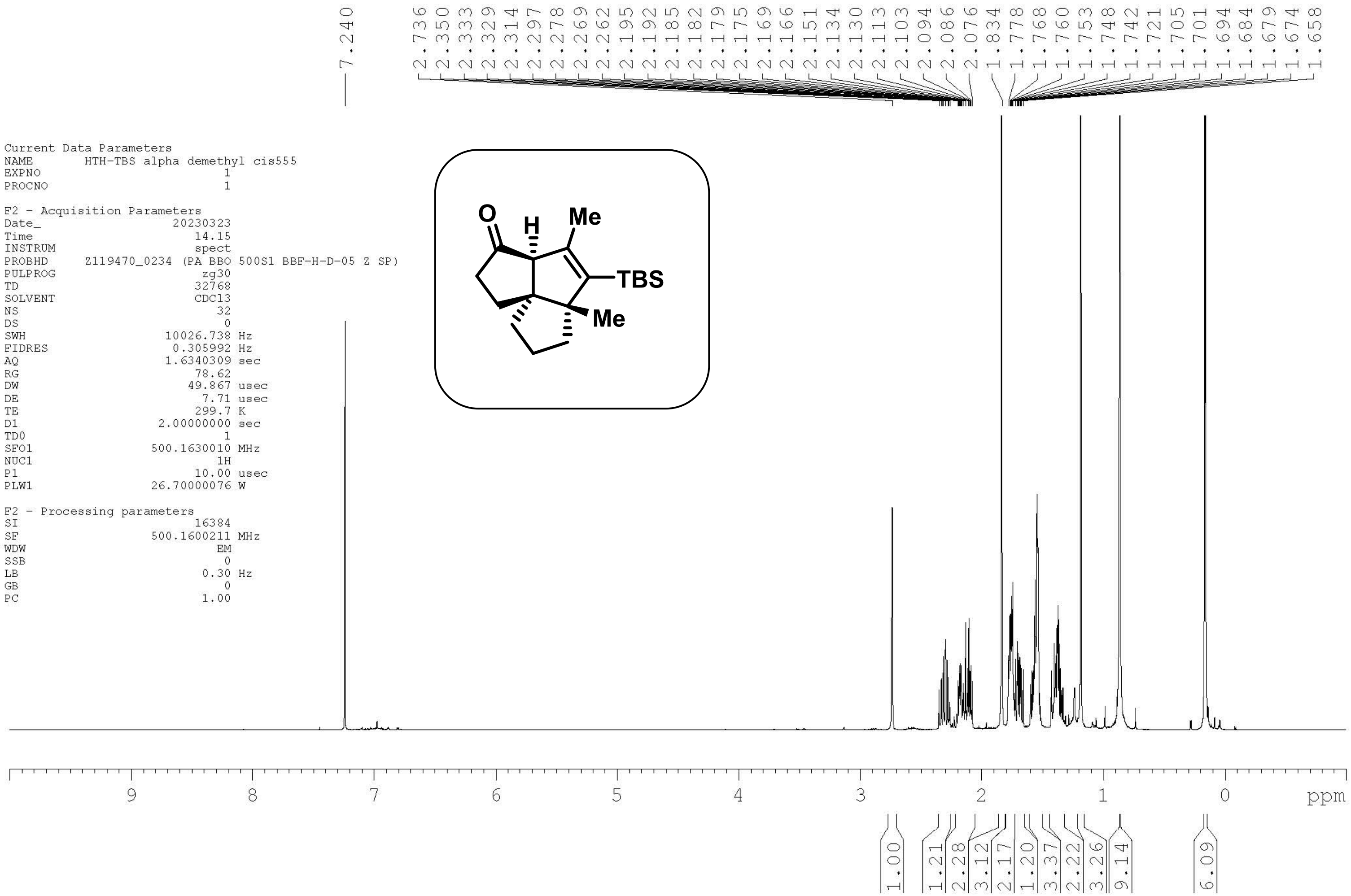

Figure 99. ^{1}H NMR (500 MHz, $CDCl_3$) (**28c-*cis,cis***)

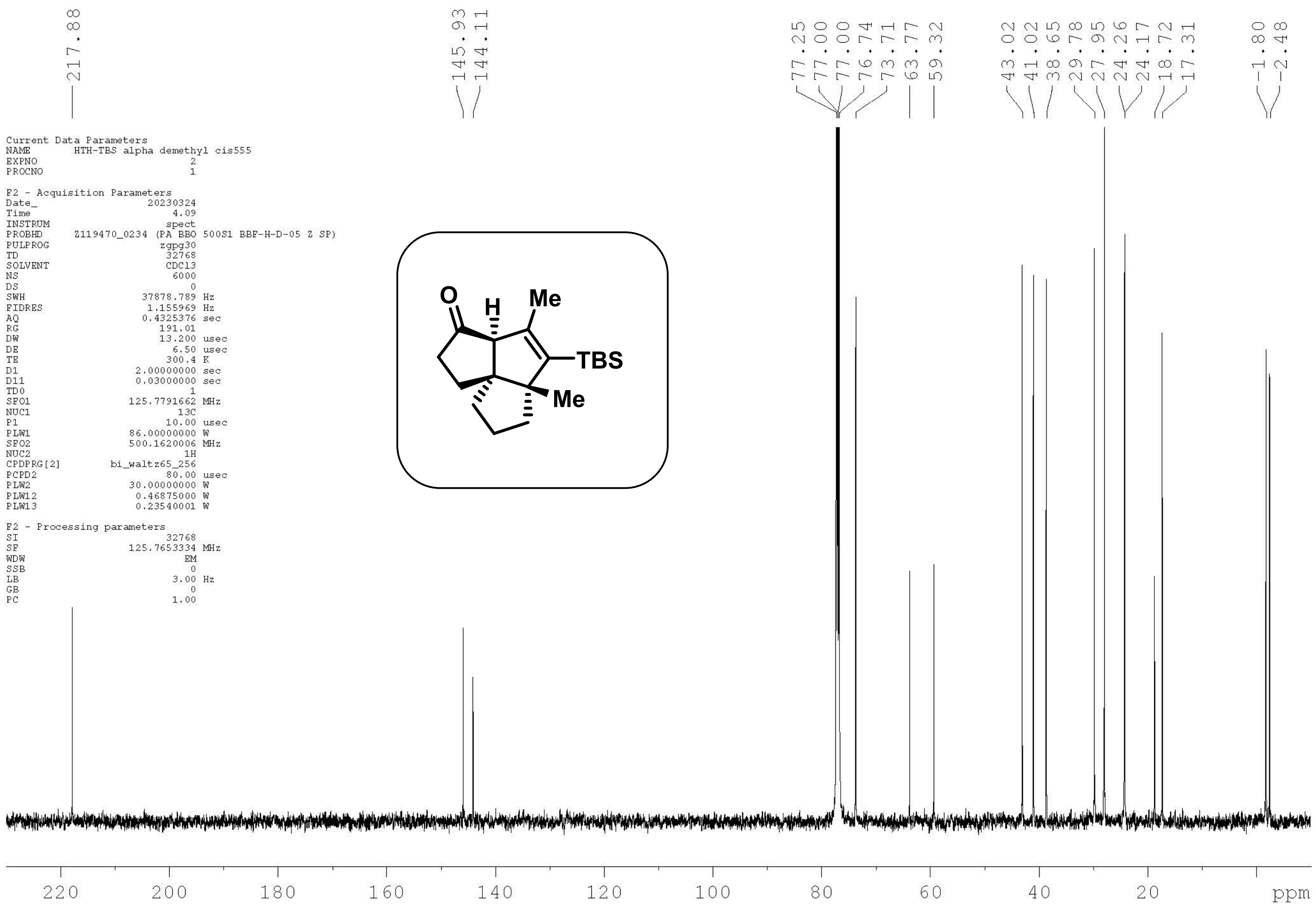

Figure 100. ^{13}C NMR (125 MHz, $CDCl_3$) (**28c-*cis,cis***)

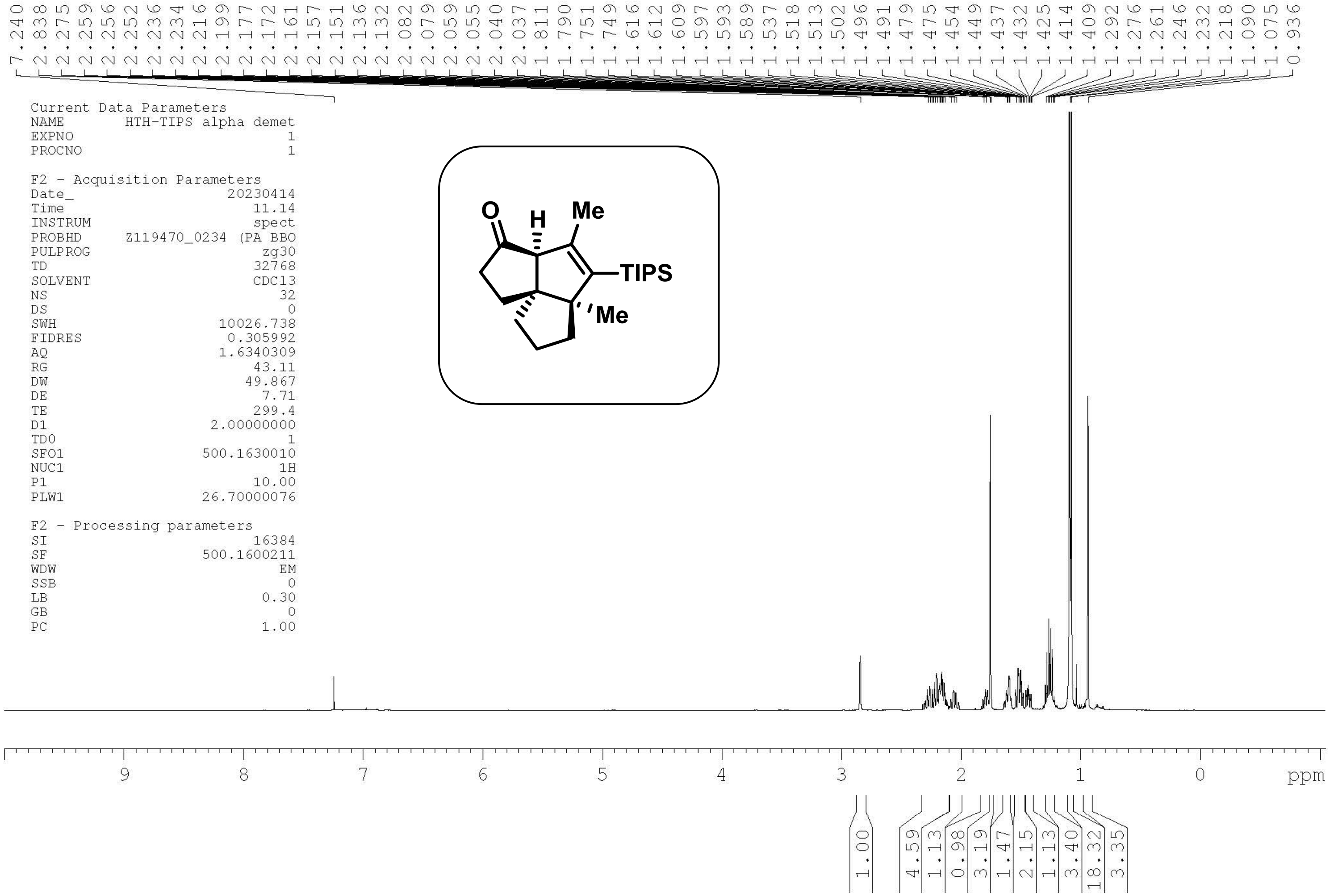

Figure 101. ^{1}H NMR (500 MHz, $CDCl_3$) (**28d-*cis,trans***)

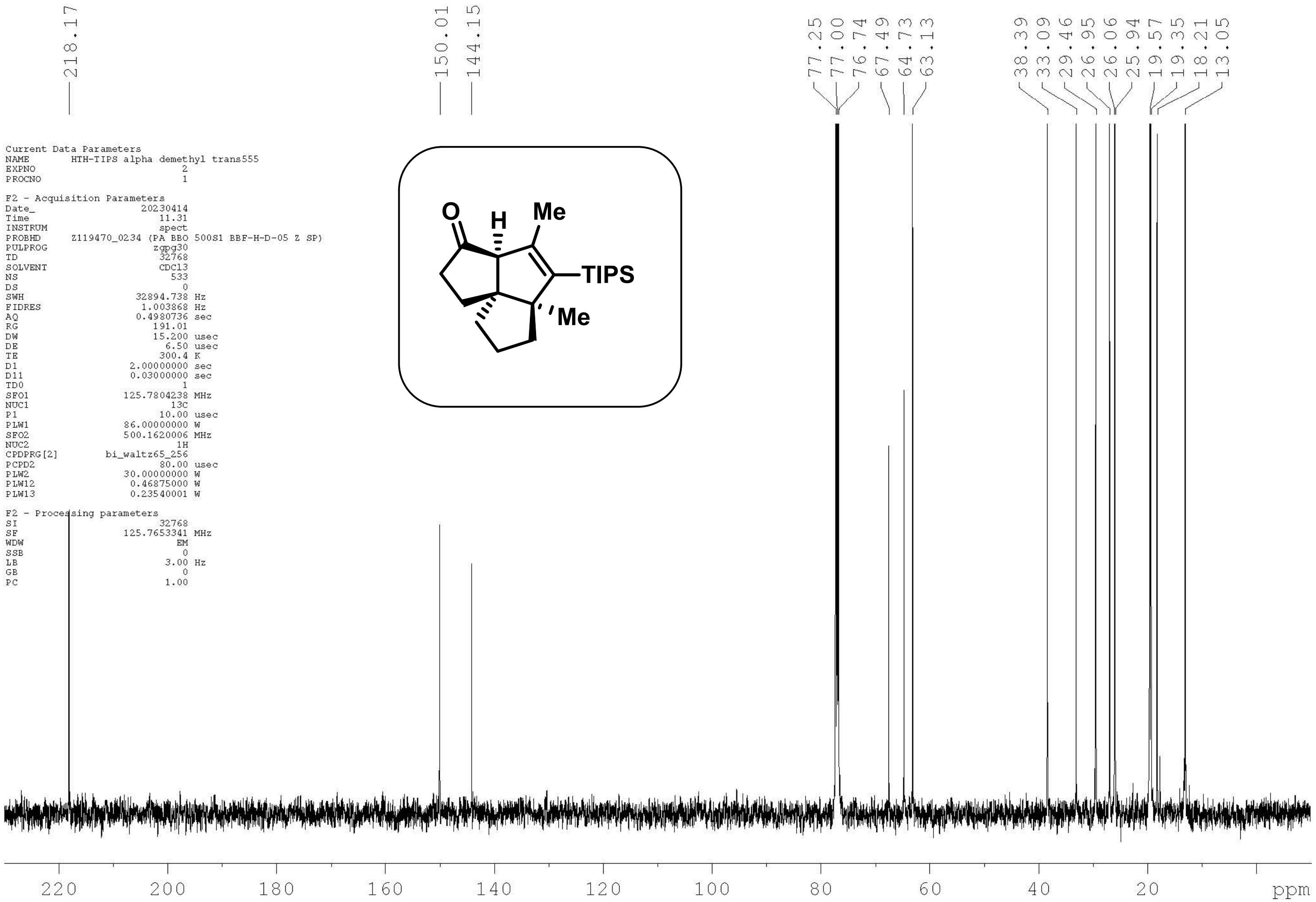

Figure 102. ^{13}C NMR (125 MHz, $CDCl_3$) (**28d-*cis,trans***)

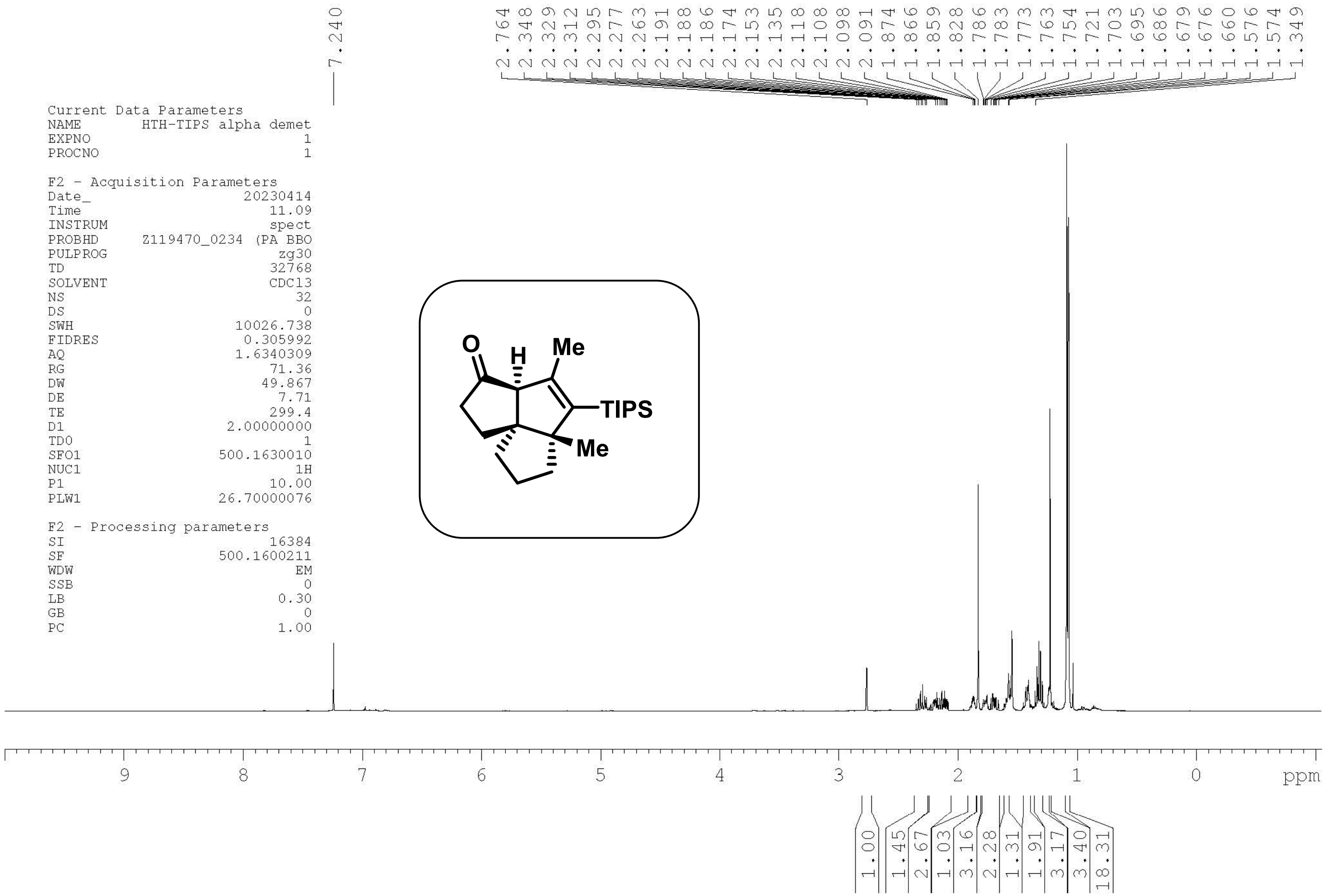

Figure 103. ^{1}H NMR (500 MHz, $CDCl_3$) (**28d-*cis,cis***)

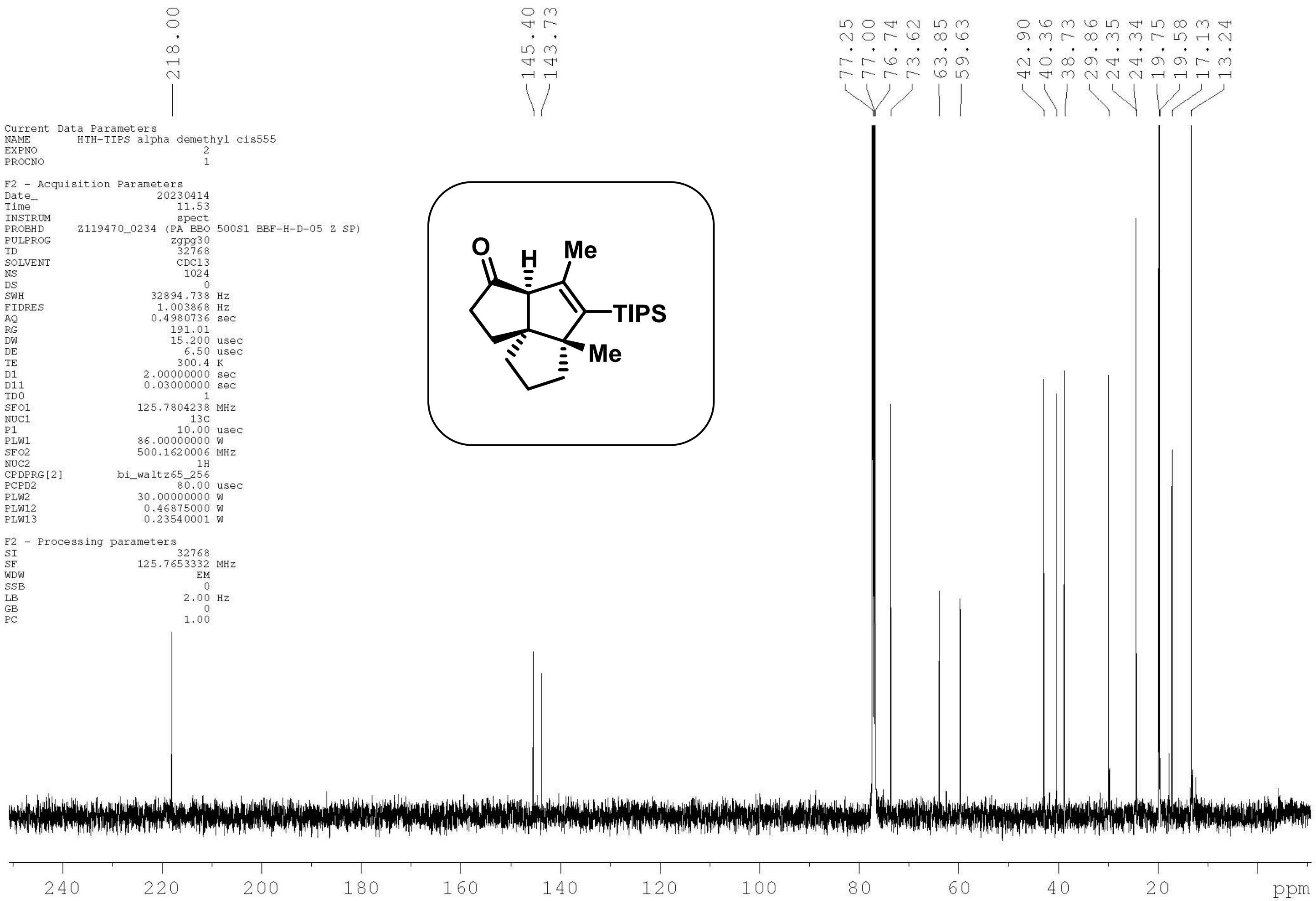

Figure 104. ^{13}C NMR (125 MHz, $CDCl_3$) (**28d-*cis,cis***)

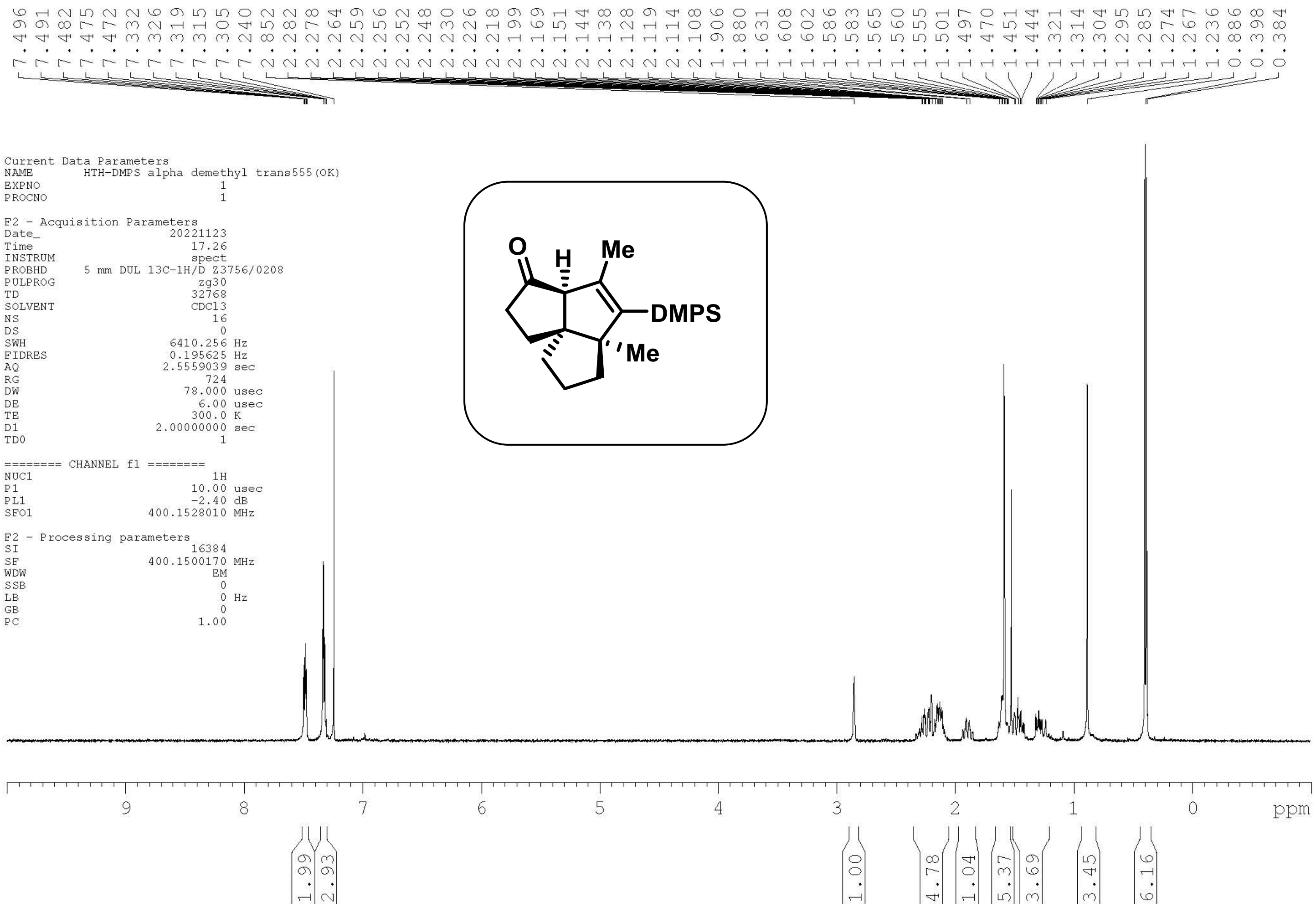

Figure 105. ^{1}H NMR (400 MHz, $CDCl_3$) (**28e-*cis,trans***)

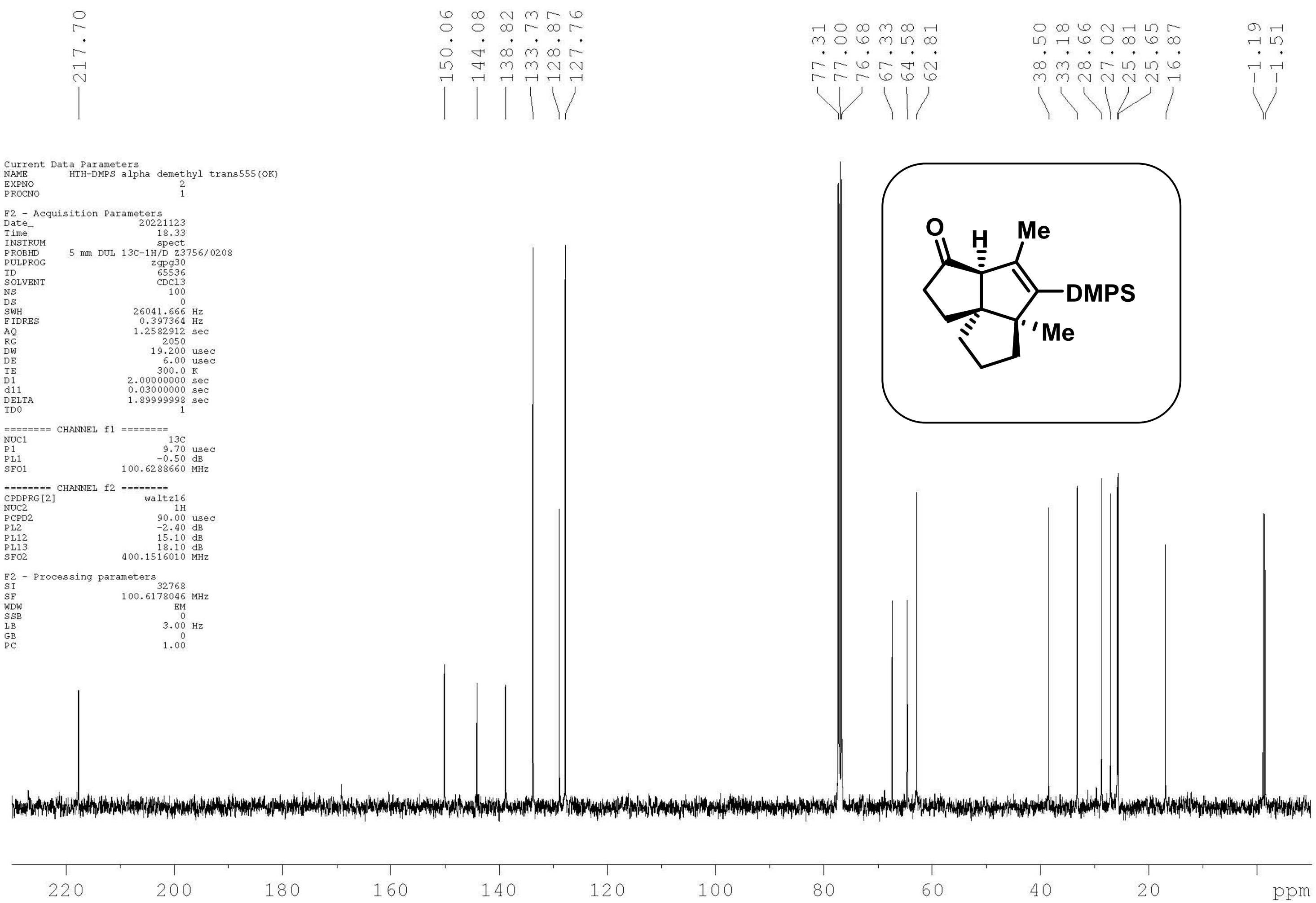

Figure 106. ^{13}C NMR (100 MHz, $CDCl_3$) (**28e-*cis,trans***)

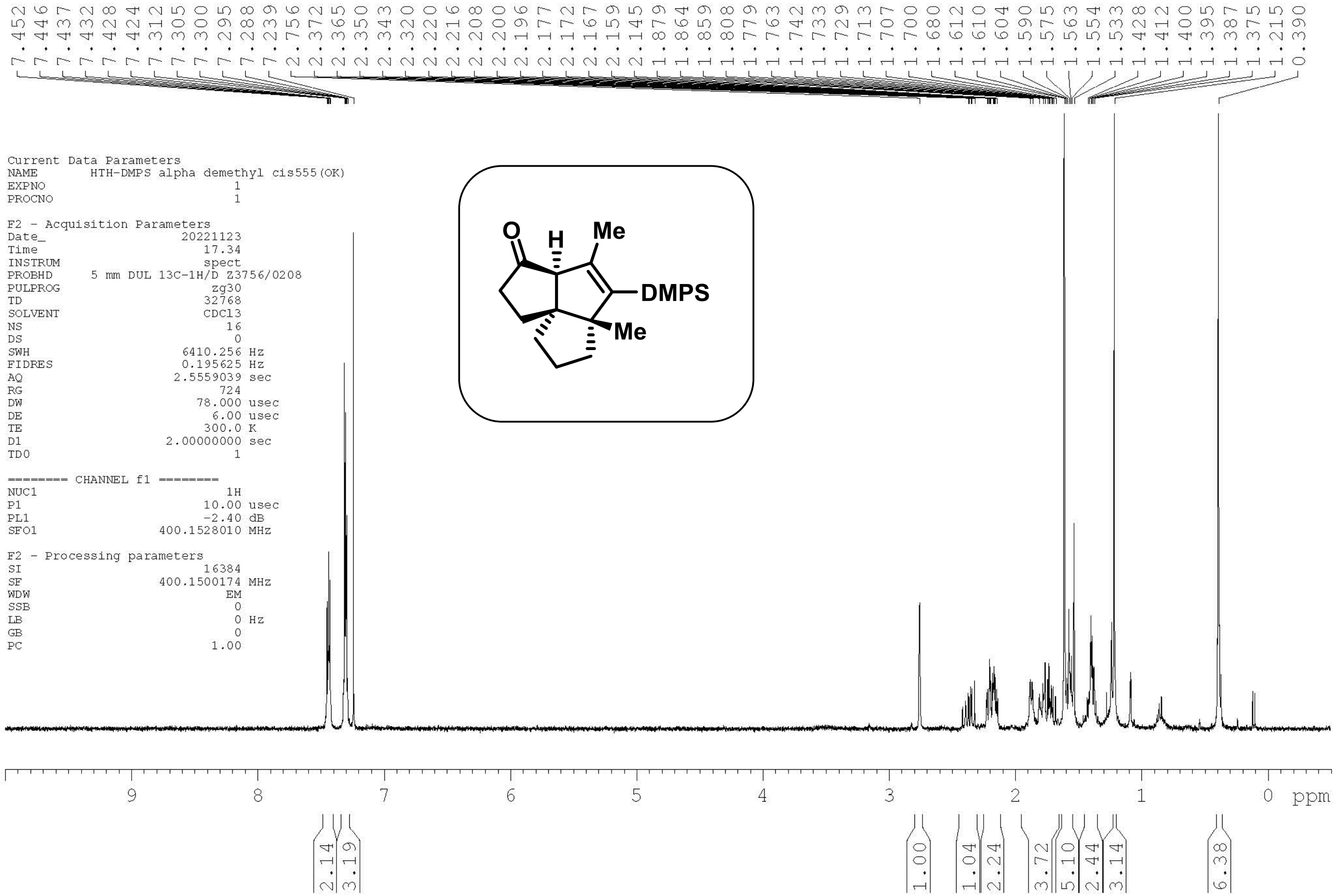

Figure 107. ^{1}H NMR (400 MHz, $CDCl_3$) (**28e-*cis,cis***)

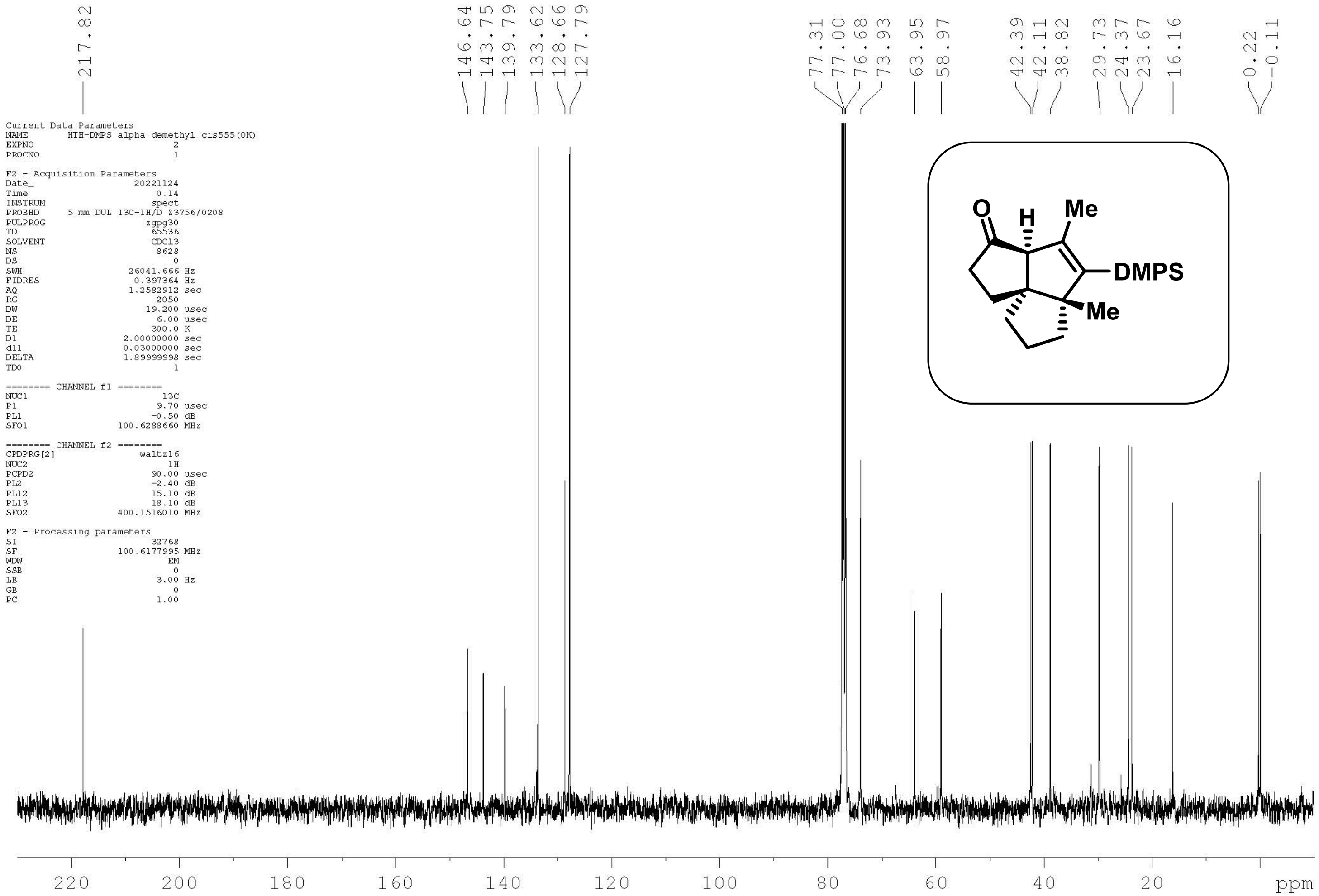

Figure 108. ^{13}C NMR (100 MHz, $CDCl_3$) (**28e-*cis,cis***)

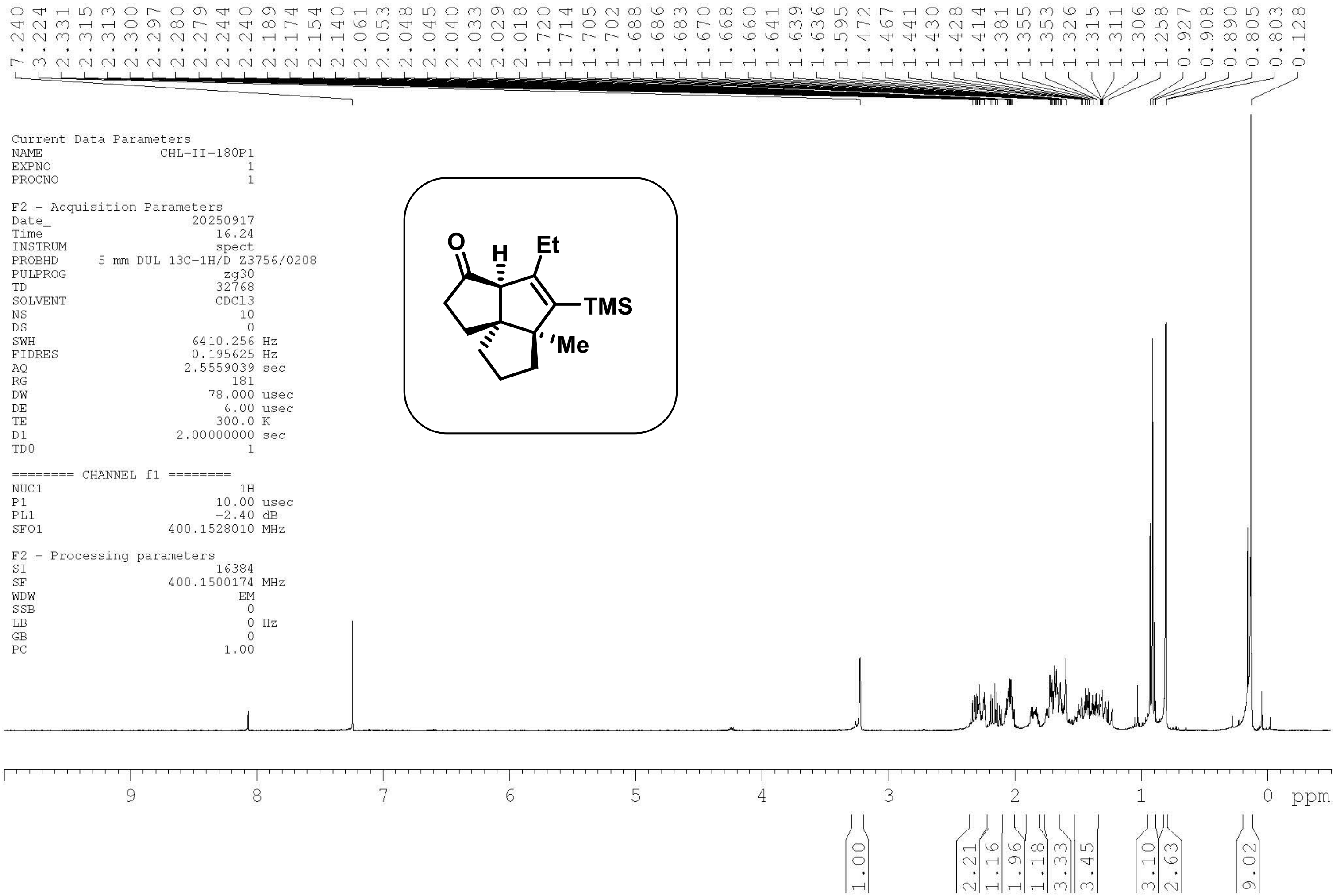

Figure 109. ^{1}H NMR (400 MHz, $CDCl_3$) (**28f-*cis,trans***)

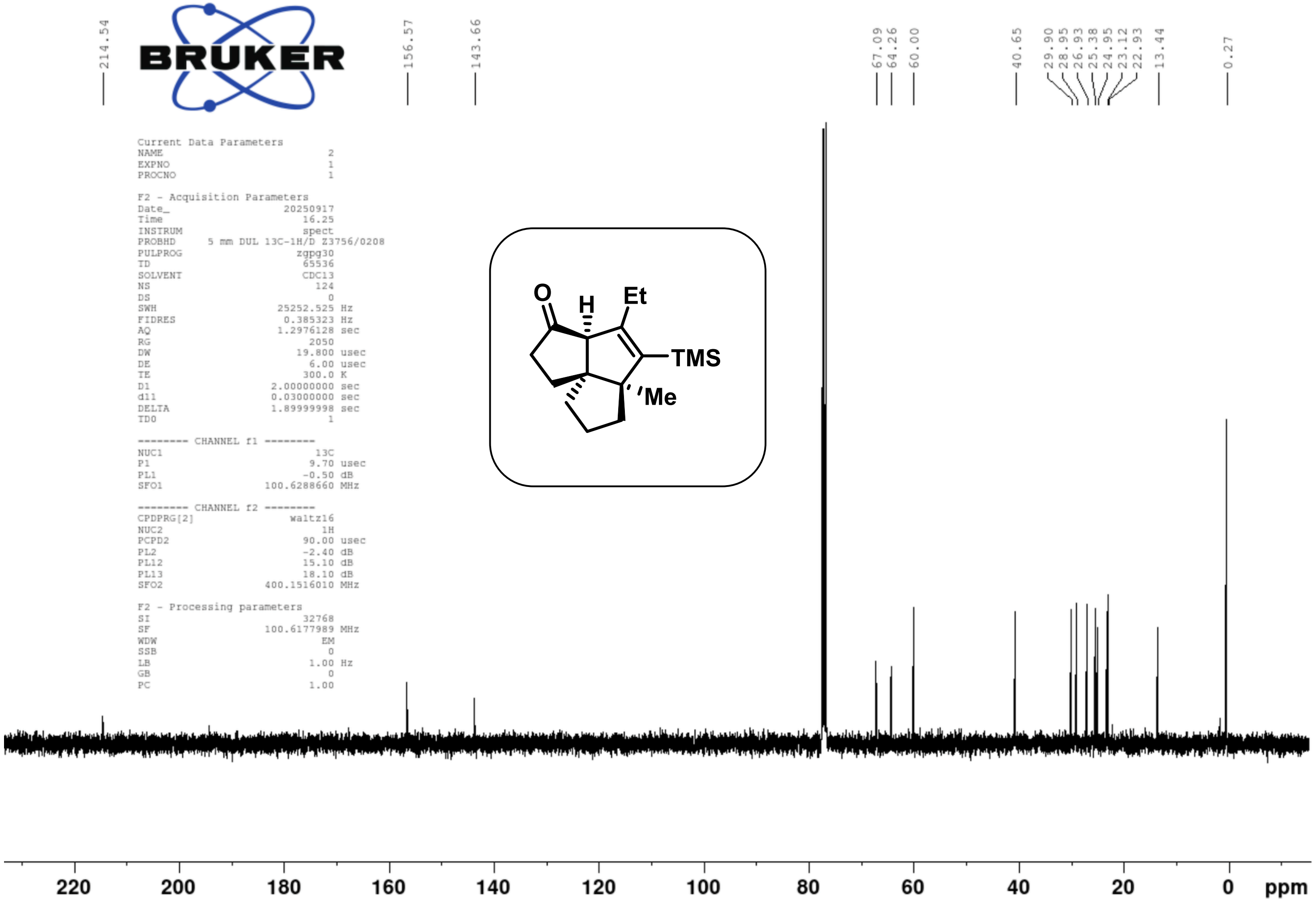

Figure 110. ^{13}C NMR (100 MHz, $CDCl_3$) (**28f-*cis,trans***)

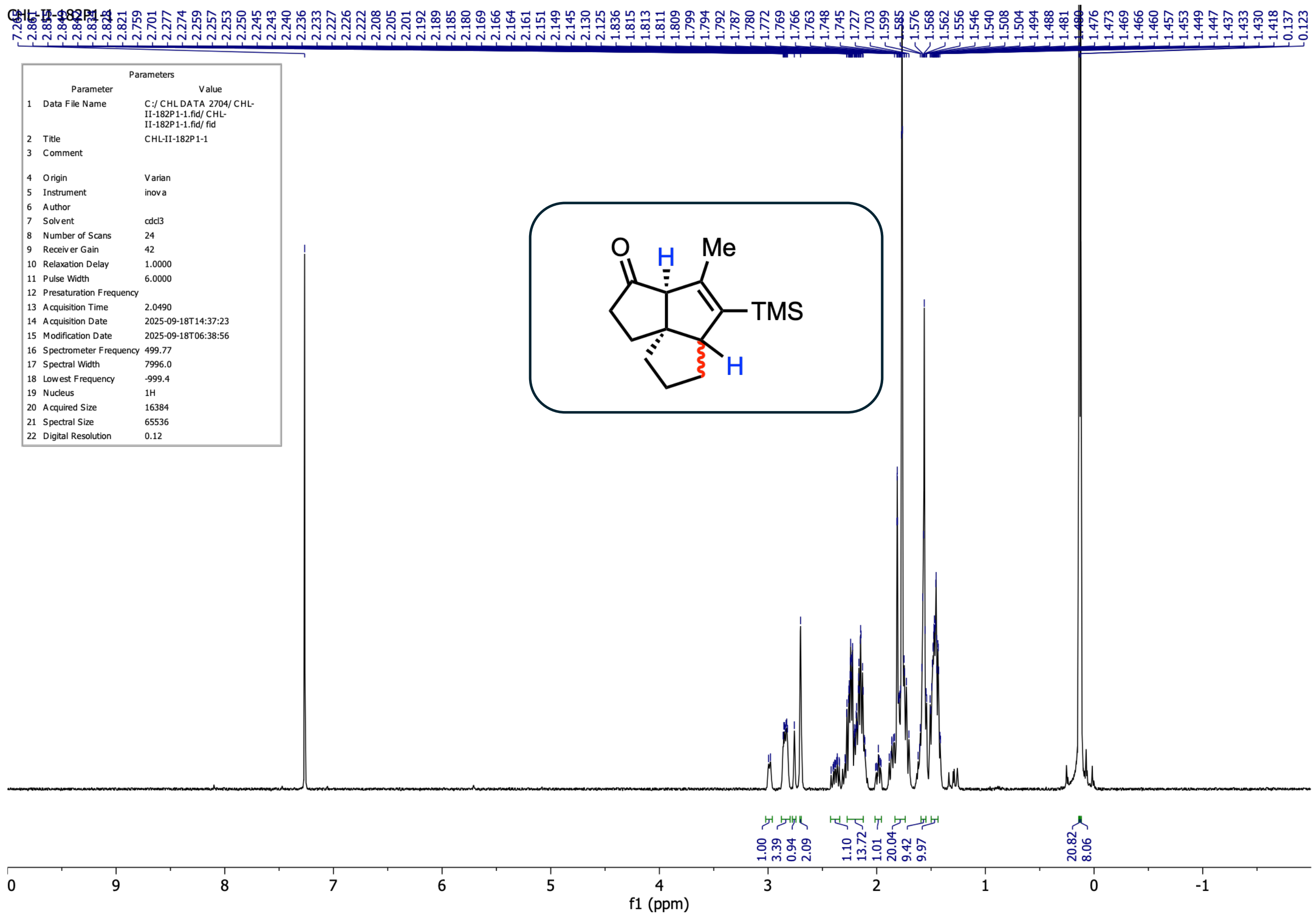

Figure 111. ^{1}H NMR (400 MHz, $CDCl_3$) (**28g-*cis*,*cis*/28g-*cis*,*trans* = 1/3**)

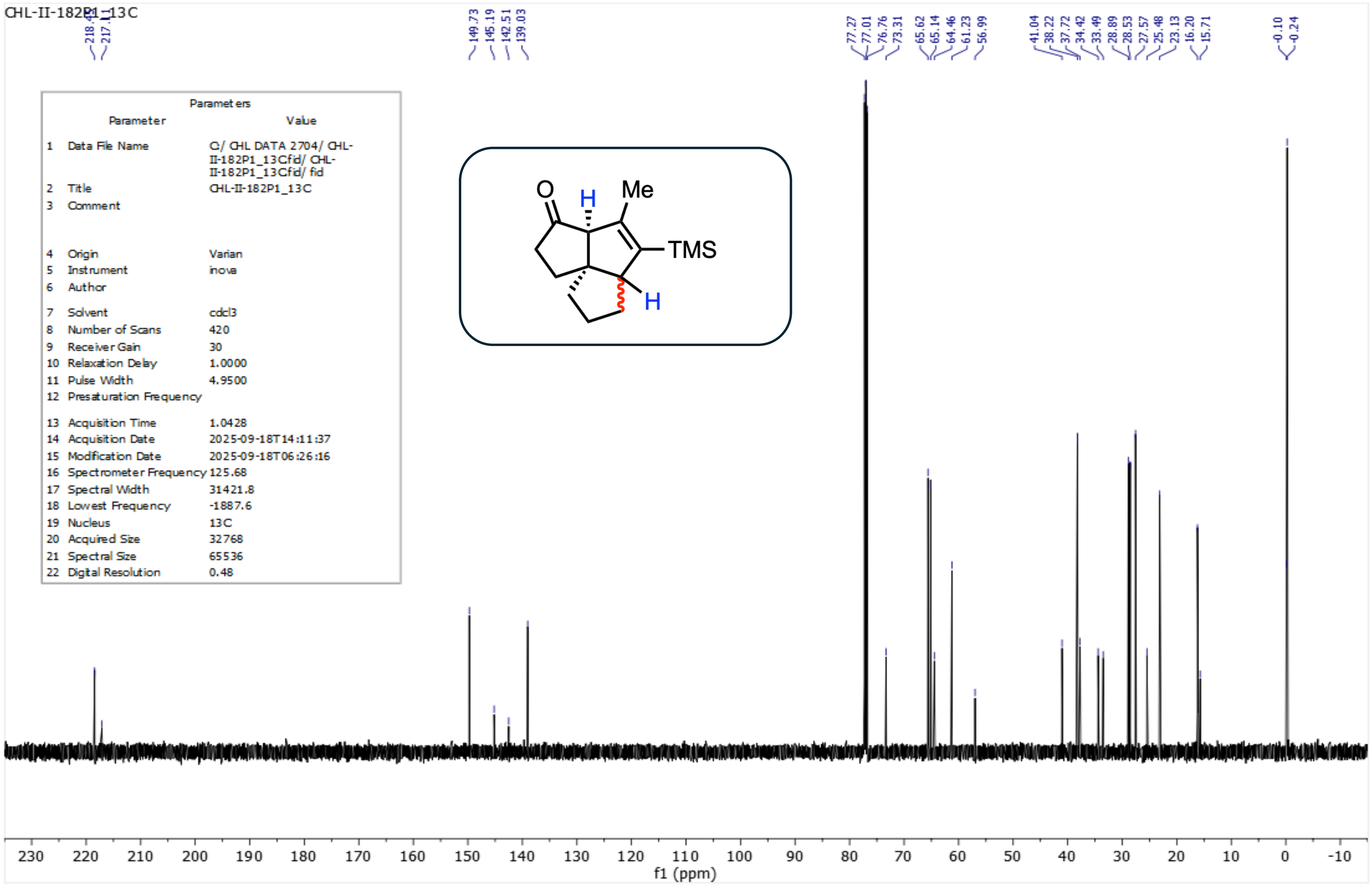

Figure 112. ^{1}H NMR (100 MHz, $CDCl_3$) (**28g-*cis,cis*/28g-*cis,trans* = 1/3**)

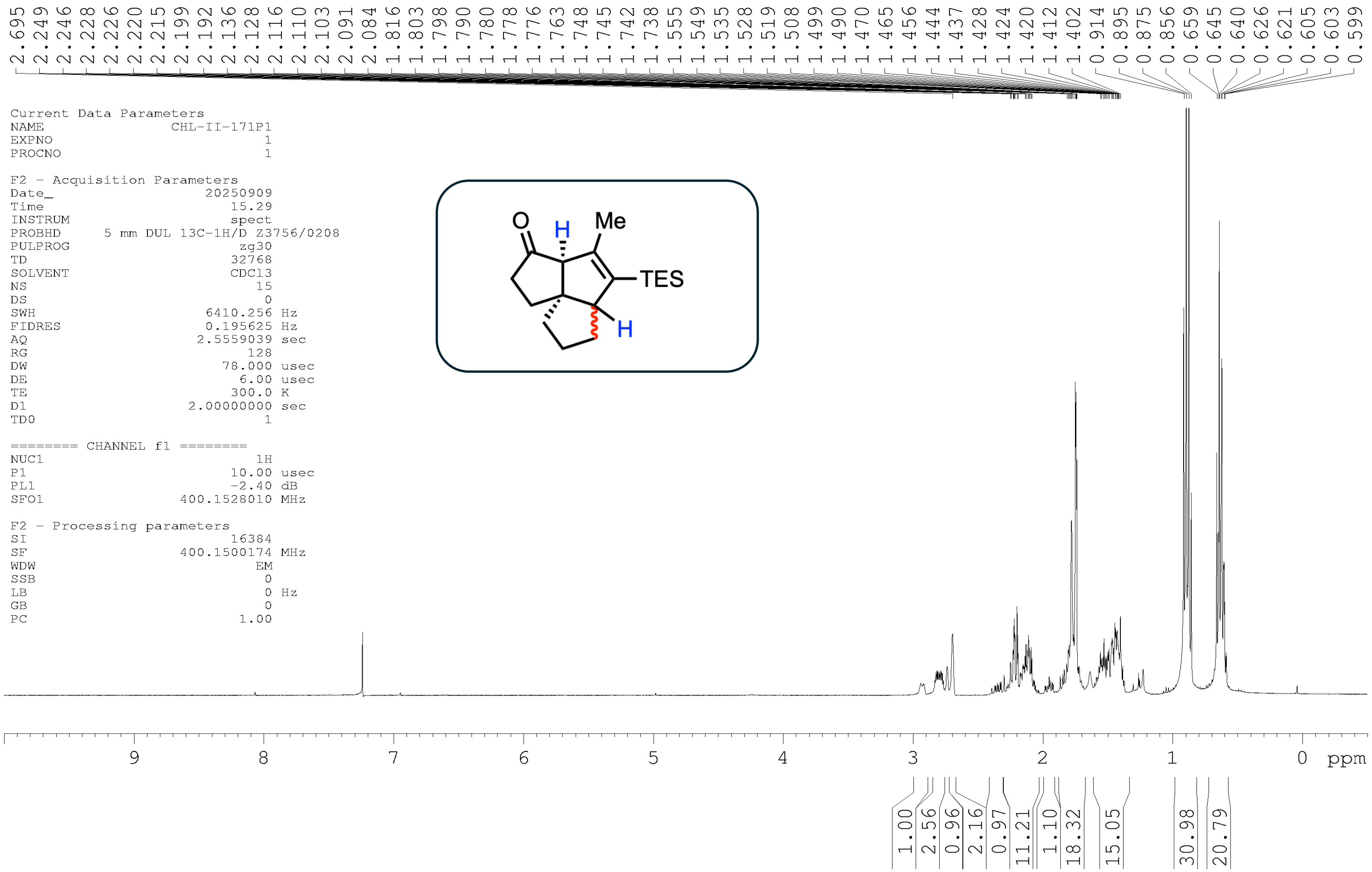

Figure 113. ^{1}H NMR (400 MHz, $CDCl_3$) (**28h-*cis*,*cis*/28h-*cis*,*trans* = 1/3**)

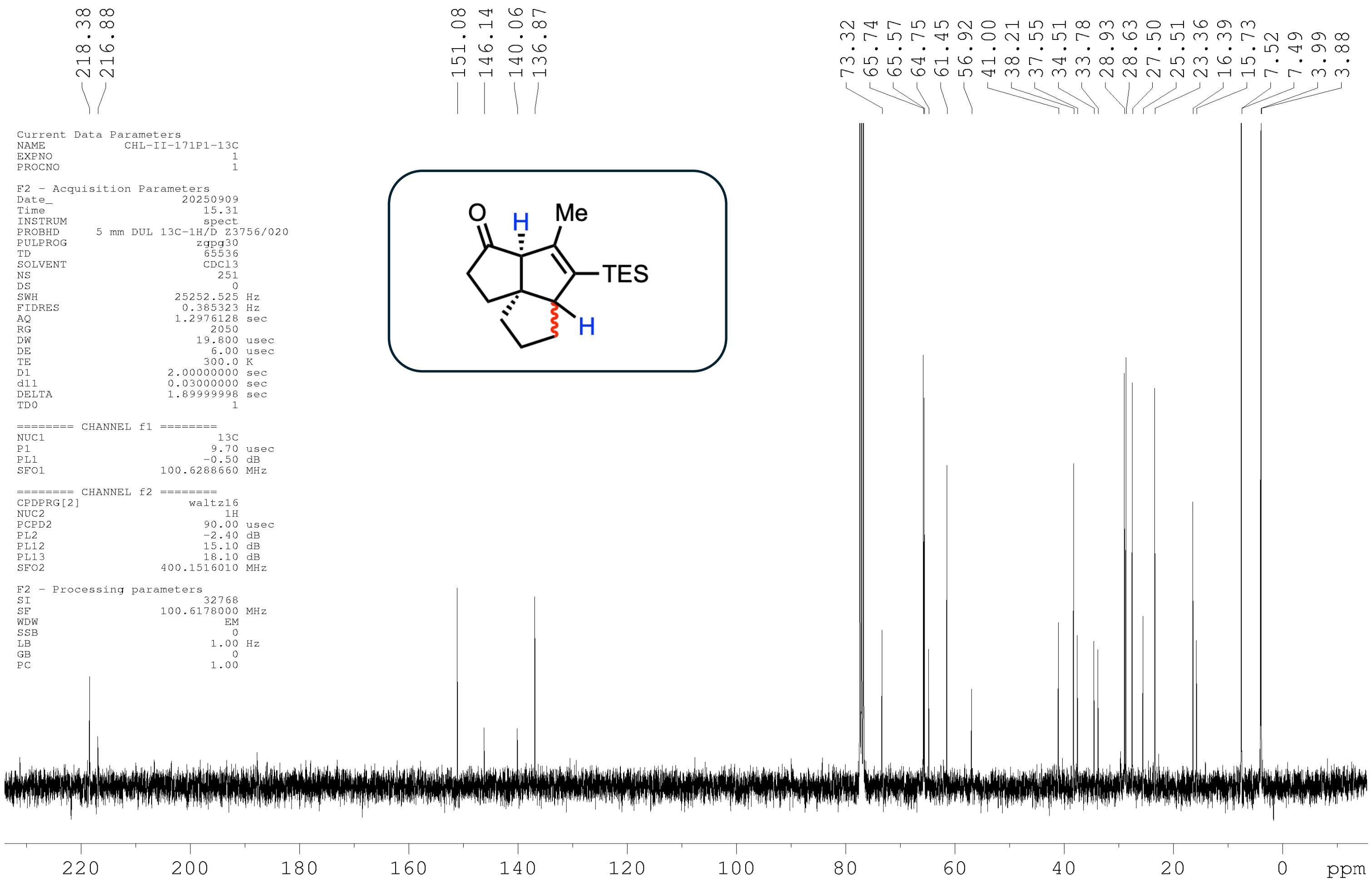

Figure 114. ^{1}H NMR (100 MHz, $CDCl_3$) (**28h-*cis*,*cis*/28h-*cis*,*trans* = 1/3**)

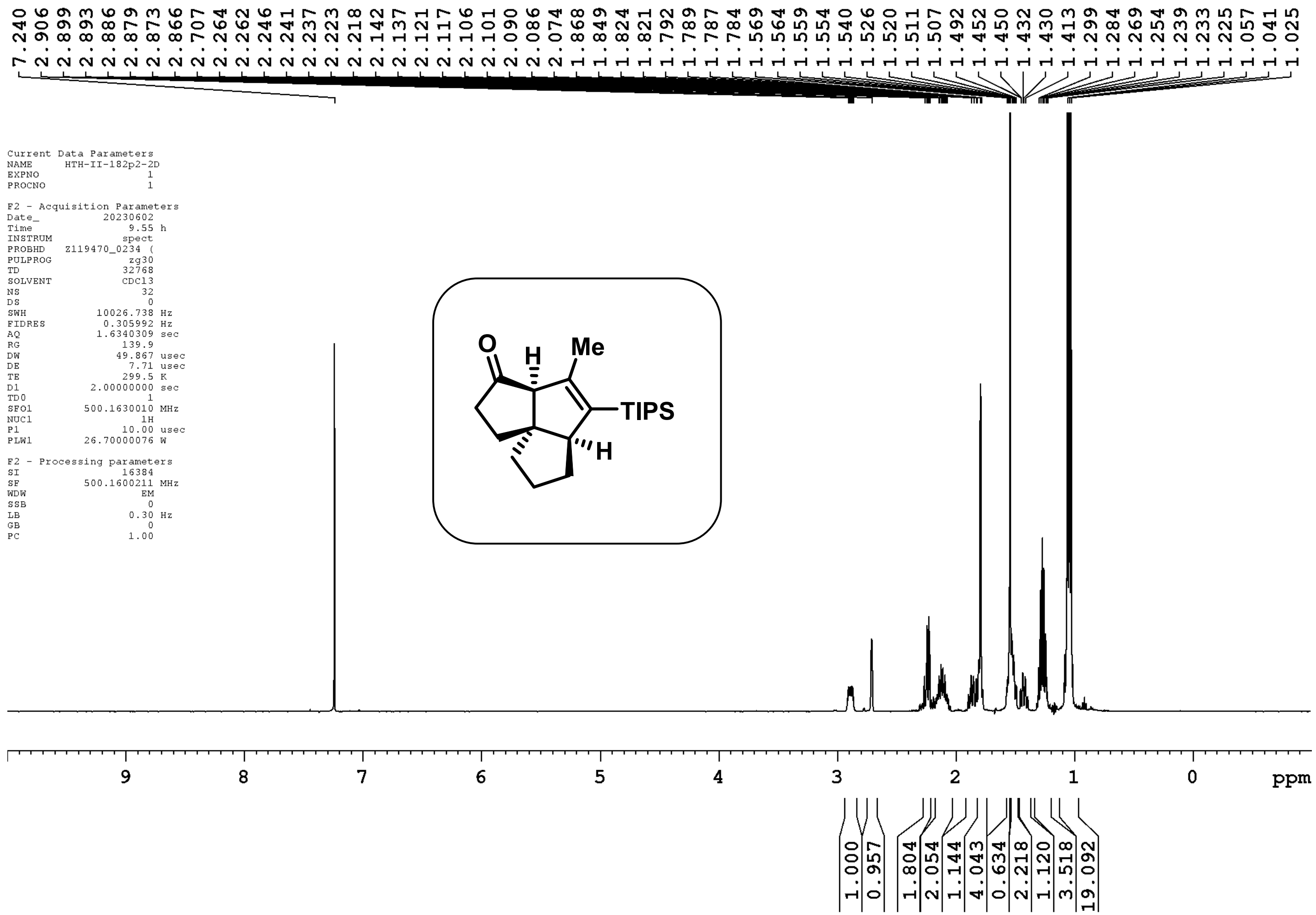

Figure 115. ¹H NMR (500 MHz, $CDCl_3$) (**28i-*cis,trans***)

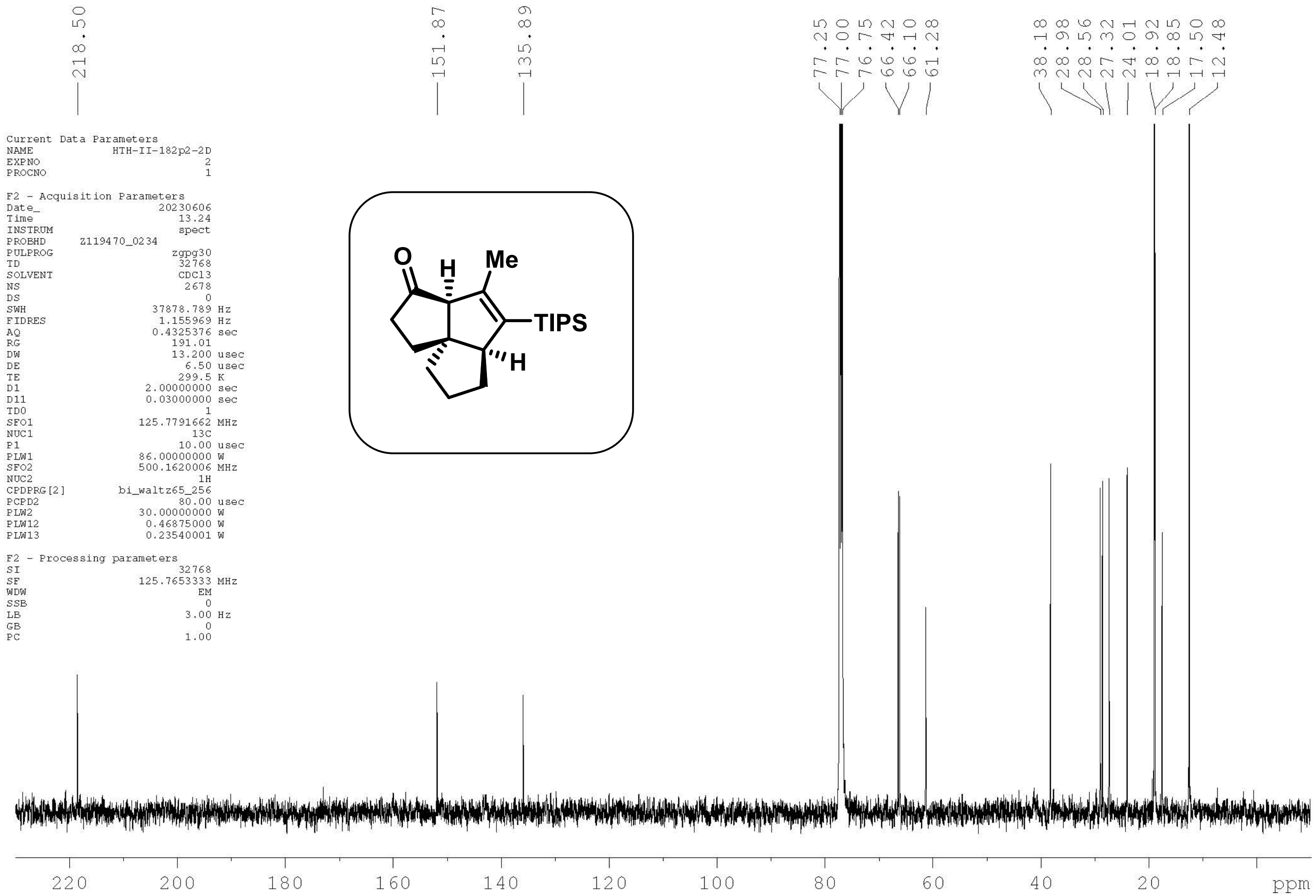

Figure 116. ^{13}C NMR (125 MHz, $CDCl_3$) (**28i-*cis,trans***)

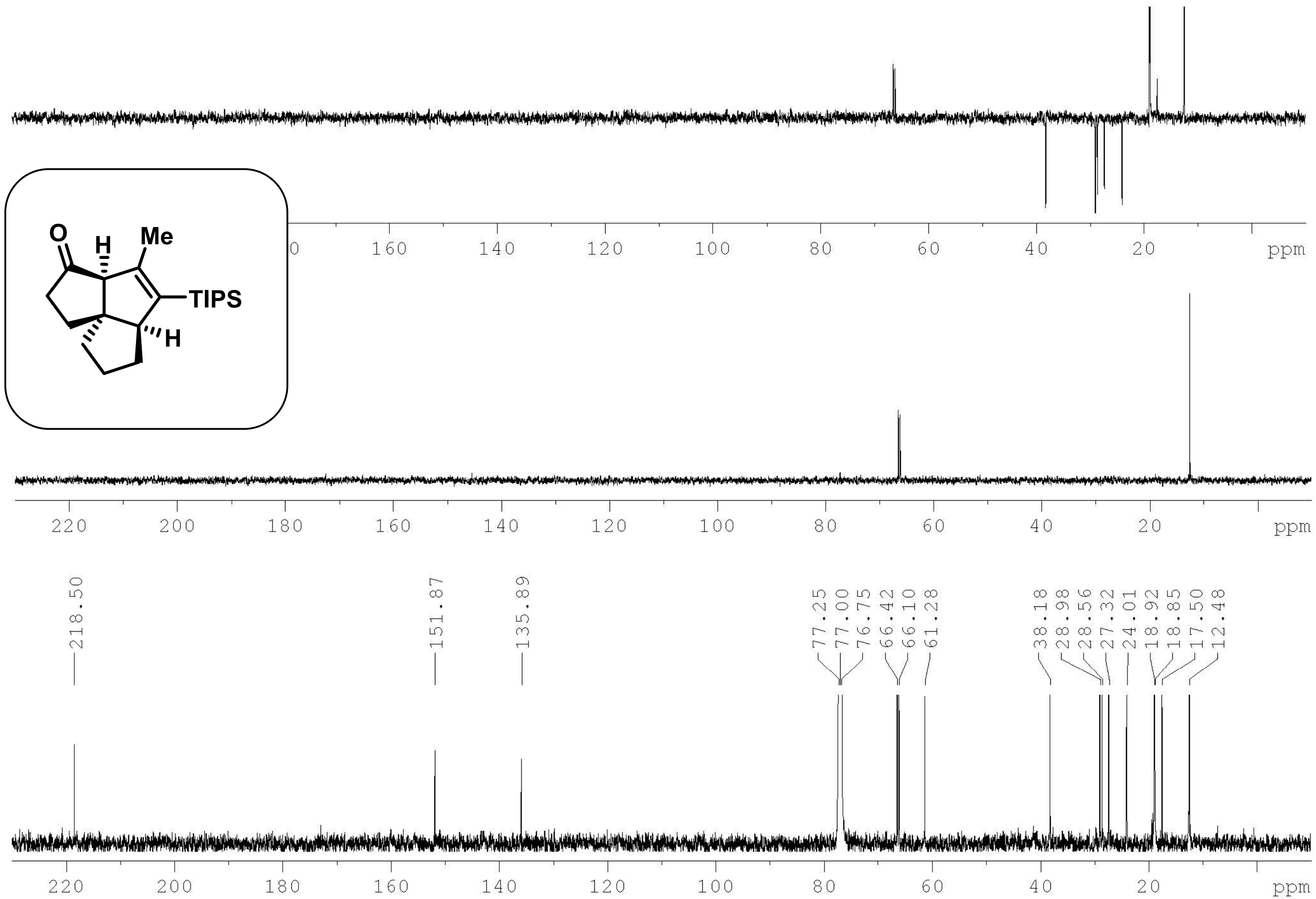

Figure 117. ^{13}C, DEPT-90, DEPT-135 NMR (125 MHz, $CDCl_3$) (**28i-*cis,trans***)

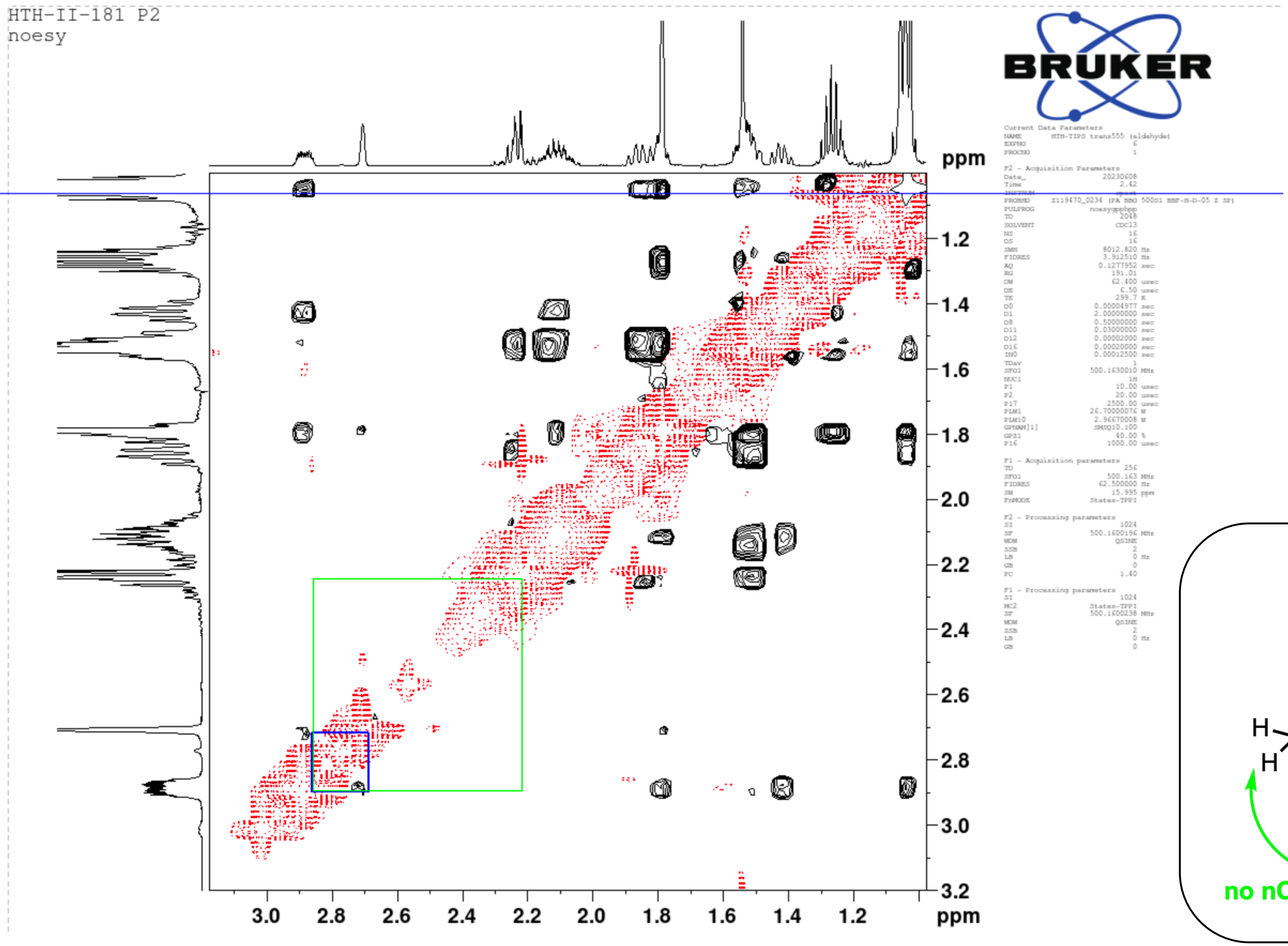

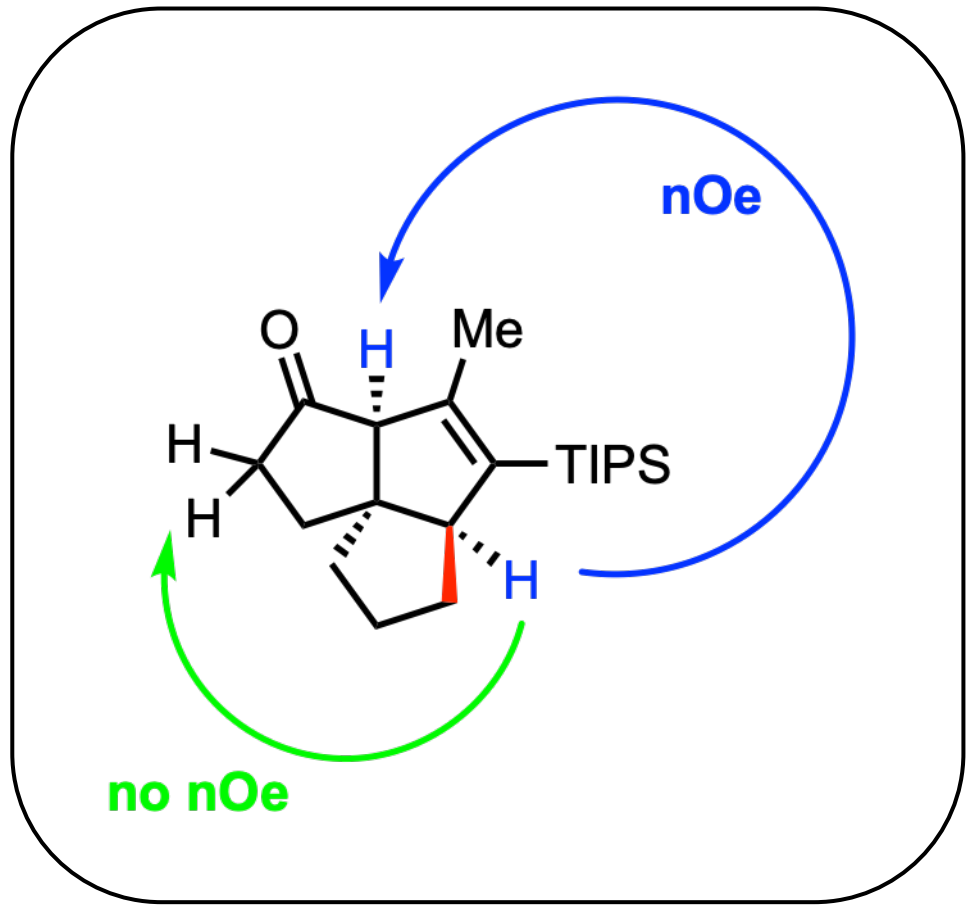

Figure 118. ^{1}H-^{1}H NOESY NMR (500 MHz, $CDCl_3$) (**28i-*cis,trans***)

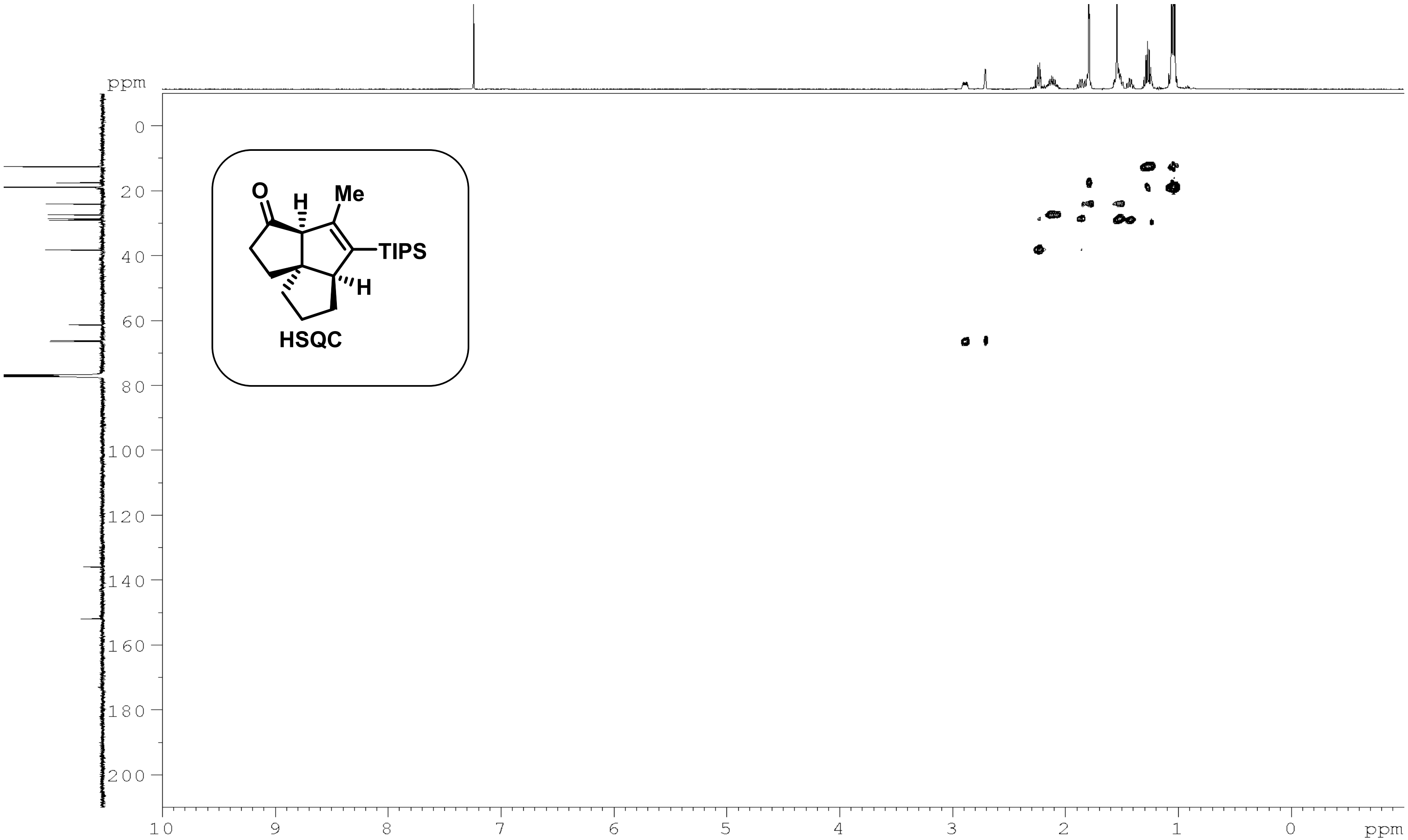

Figure 119. ^{1}H-^{13}C HSQC NMR (500 MHz, $CDCl_3$) (**28i-*cis*,*trans***)

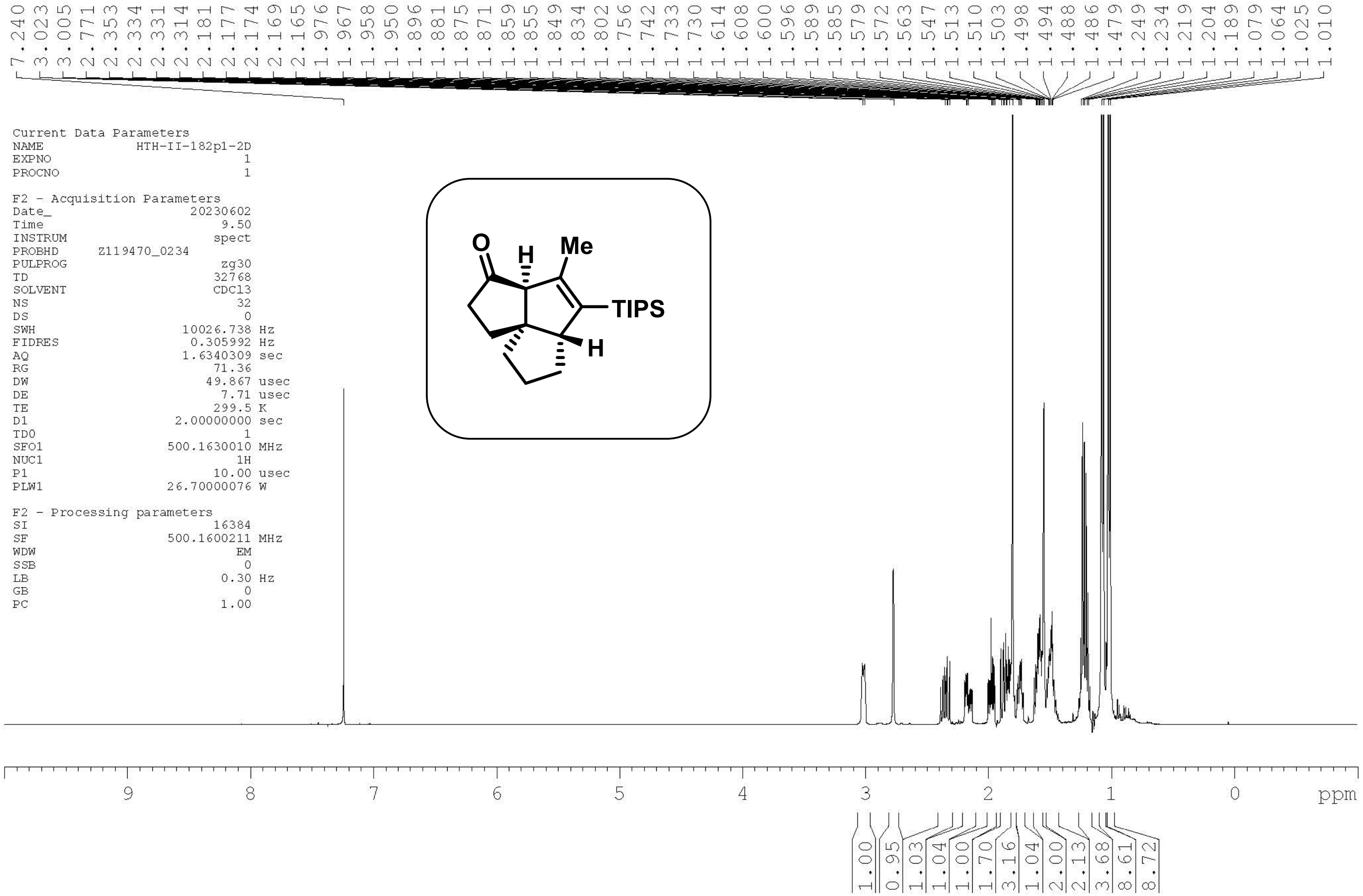

Figure 120. ^{1}H NMR (500 MHz, $CDCl_3$) (**28i-*cis,cis***)

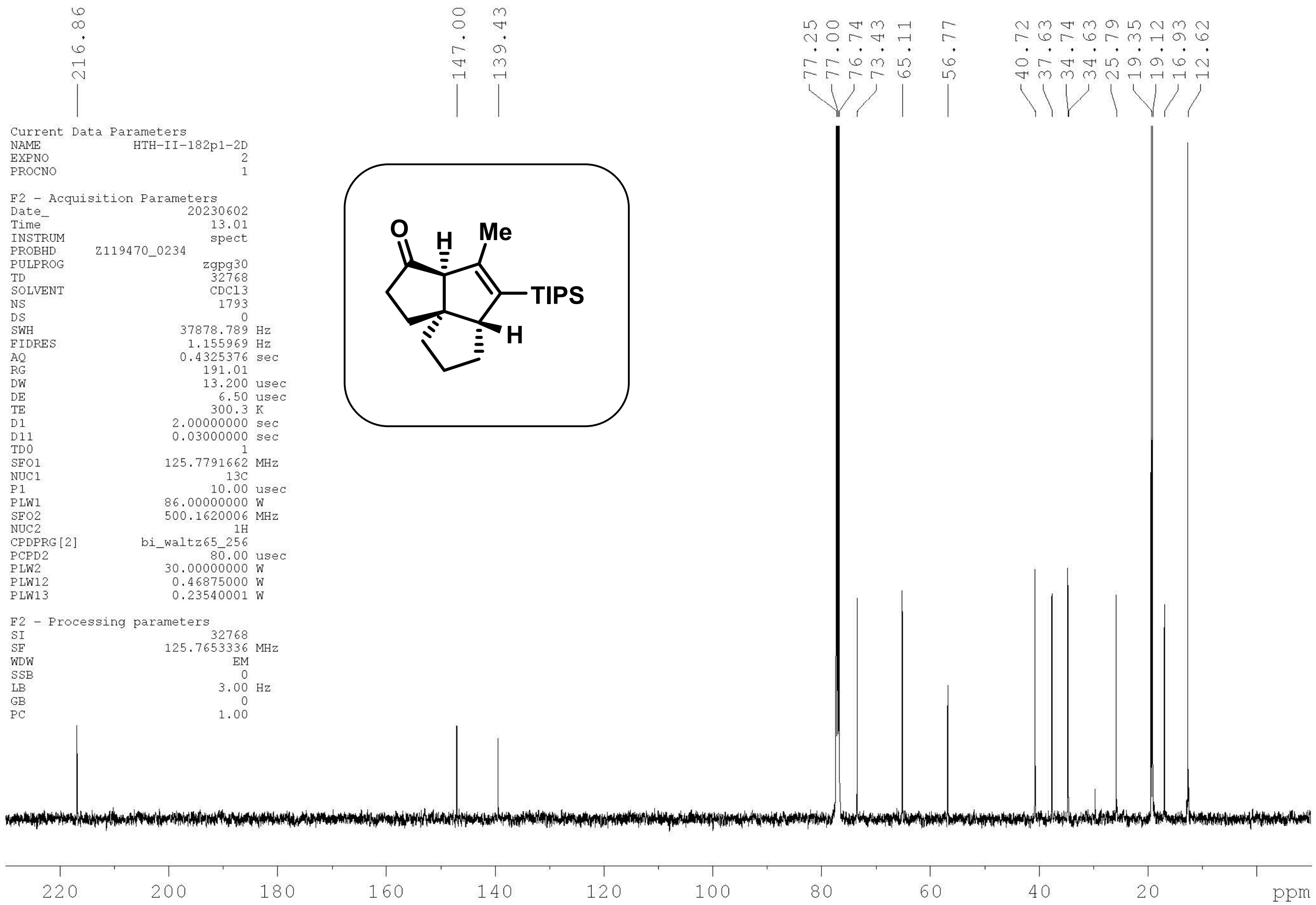

Figure 121. ^{13}C NMR (125 MHz, $CDCl_3$) (**28i-*cis*,*cis***)

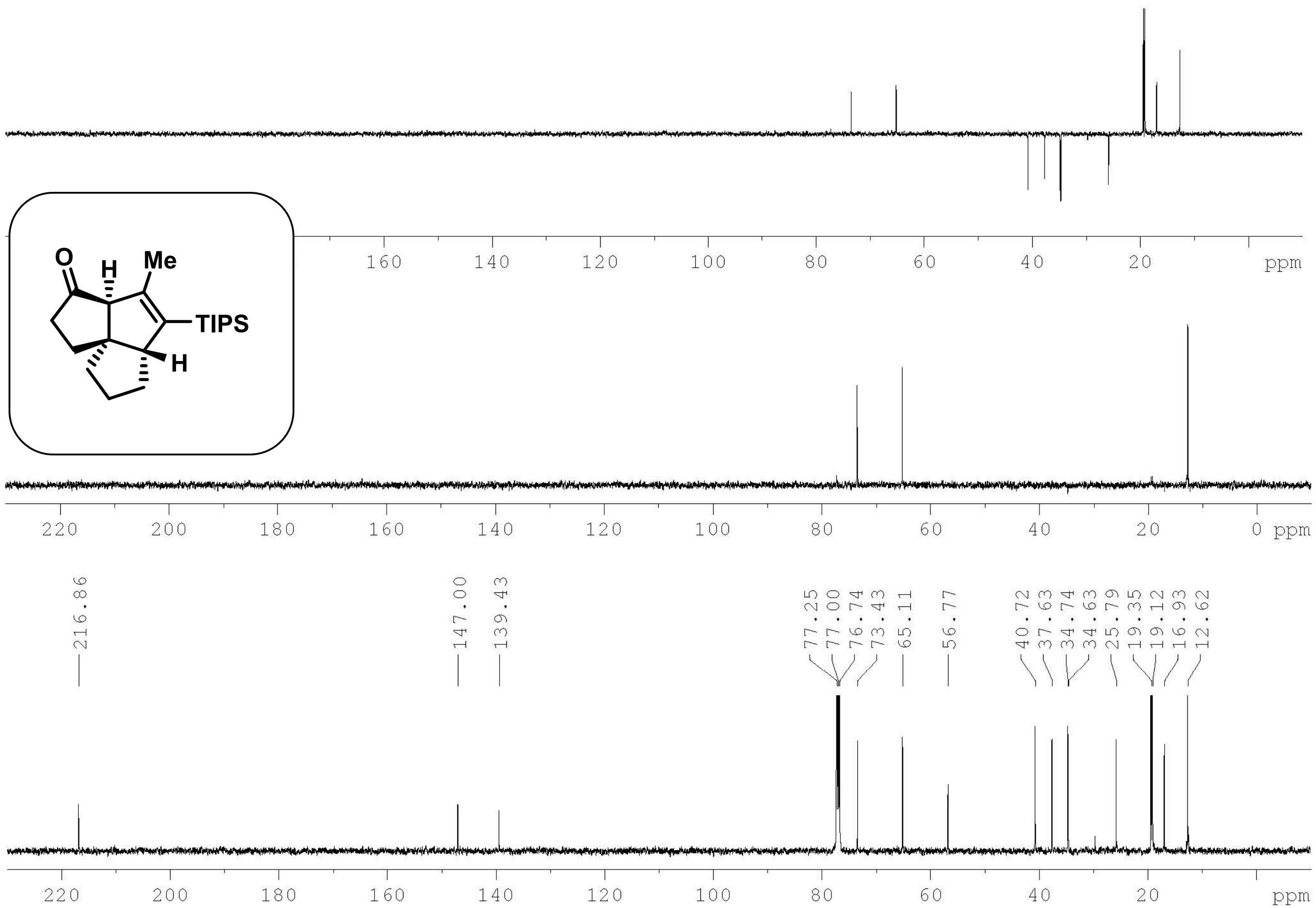

Figure 122. ^{13}C, DEPT-90, DEPT-135 NMR (125 MHz, $CDCl_3$) (**28i-*cis*,*cis***)

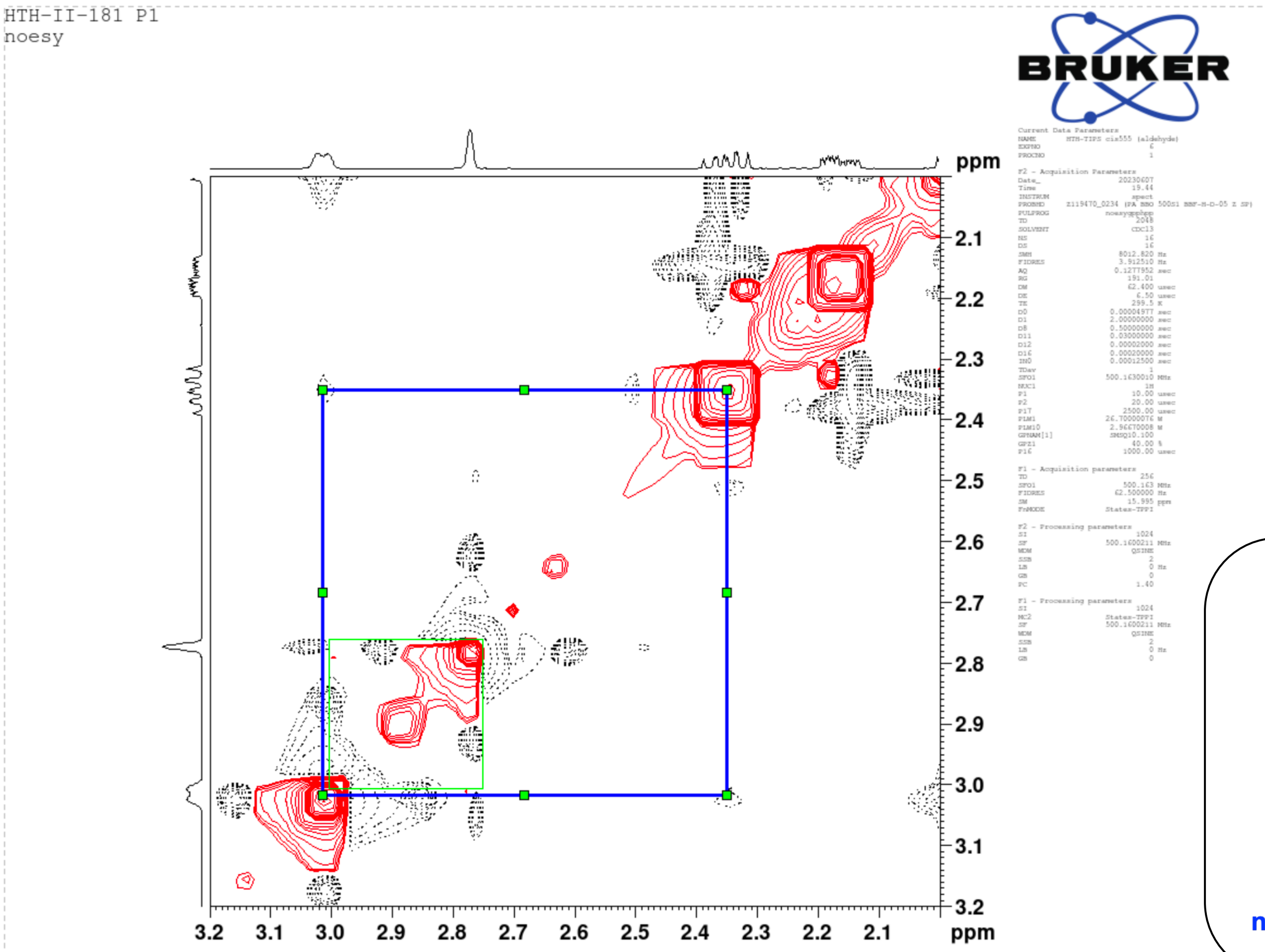

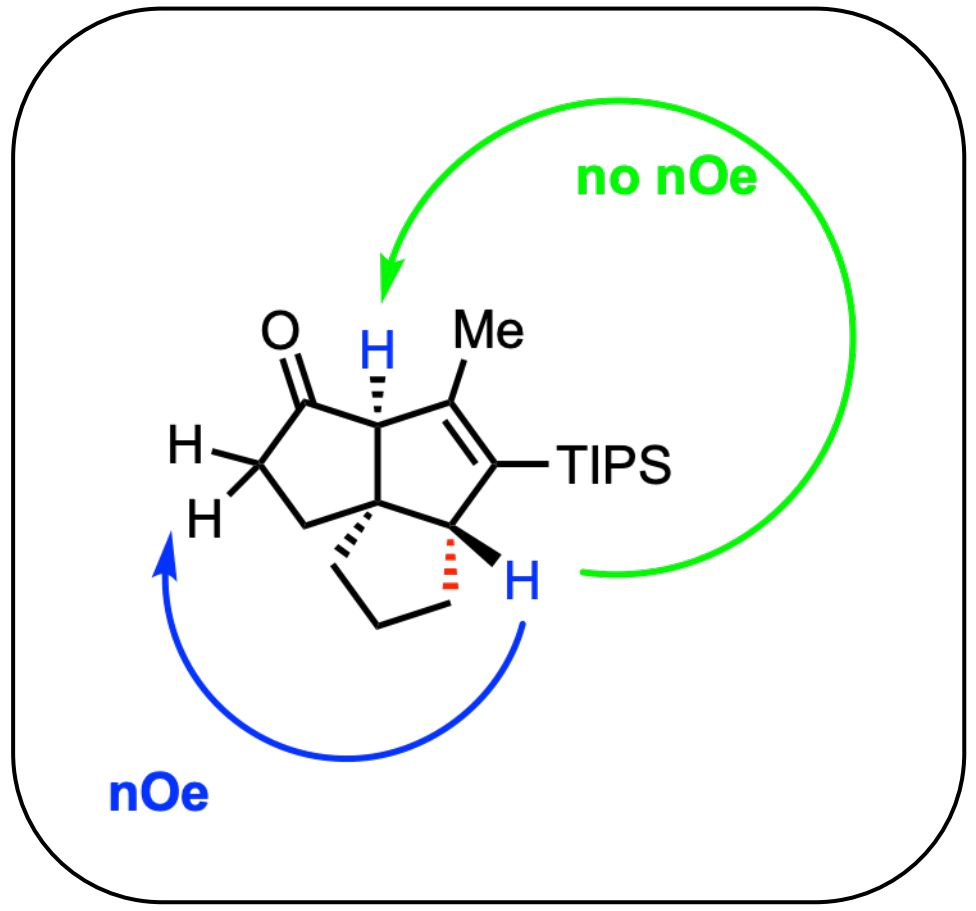

Figure 123. ^{1}H-^{1}H NOESY NMR (500 MHz, $CDCl_3$) (**28i-*cis,cis***)

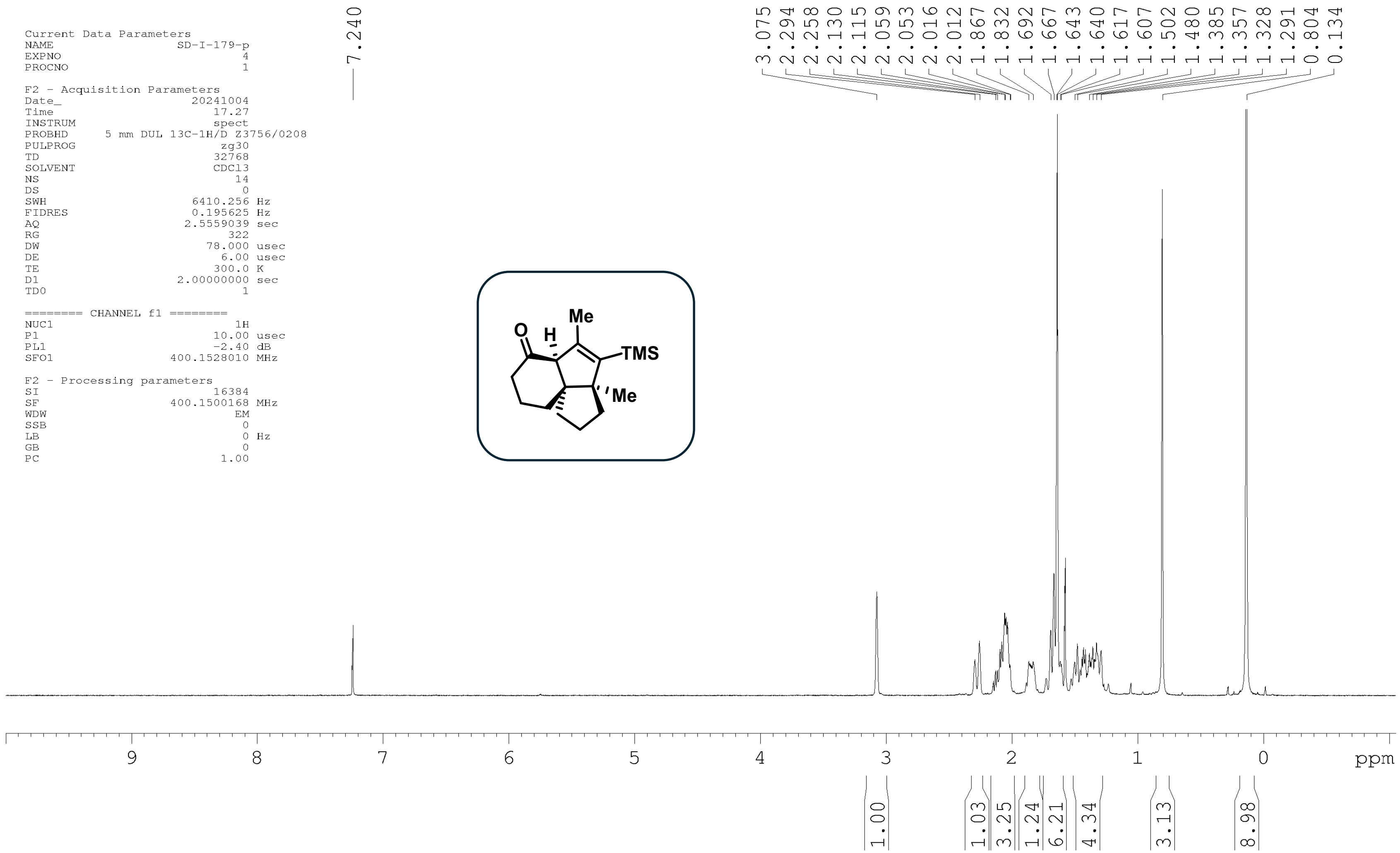

Figure 124. ^{1}H NMR (400 MHz, $CDCl_3$) (**28j-*cis,trans***)

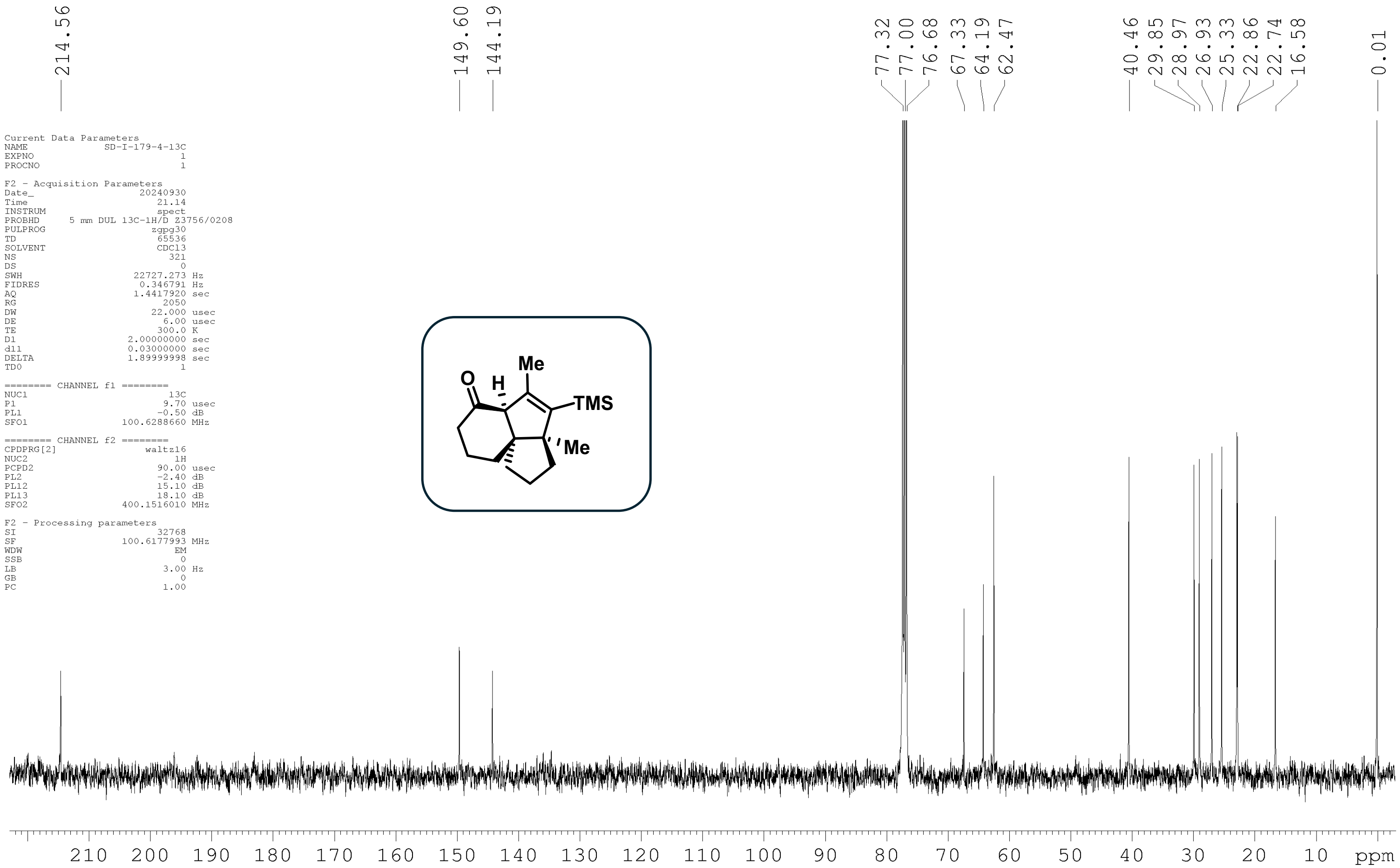

Figure 125. ^{13}C NMR (100 MHz, $CDCl_3$) (**28j-*cis*,*trans***)

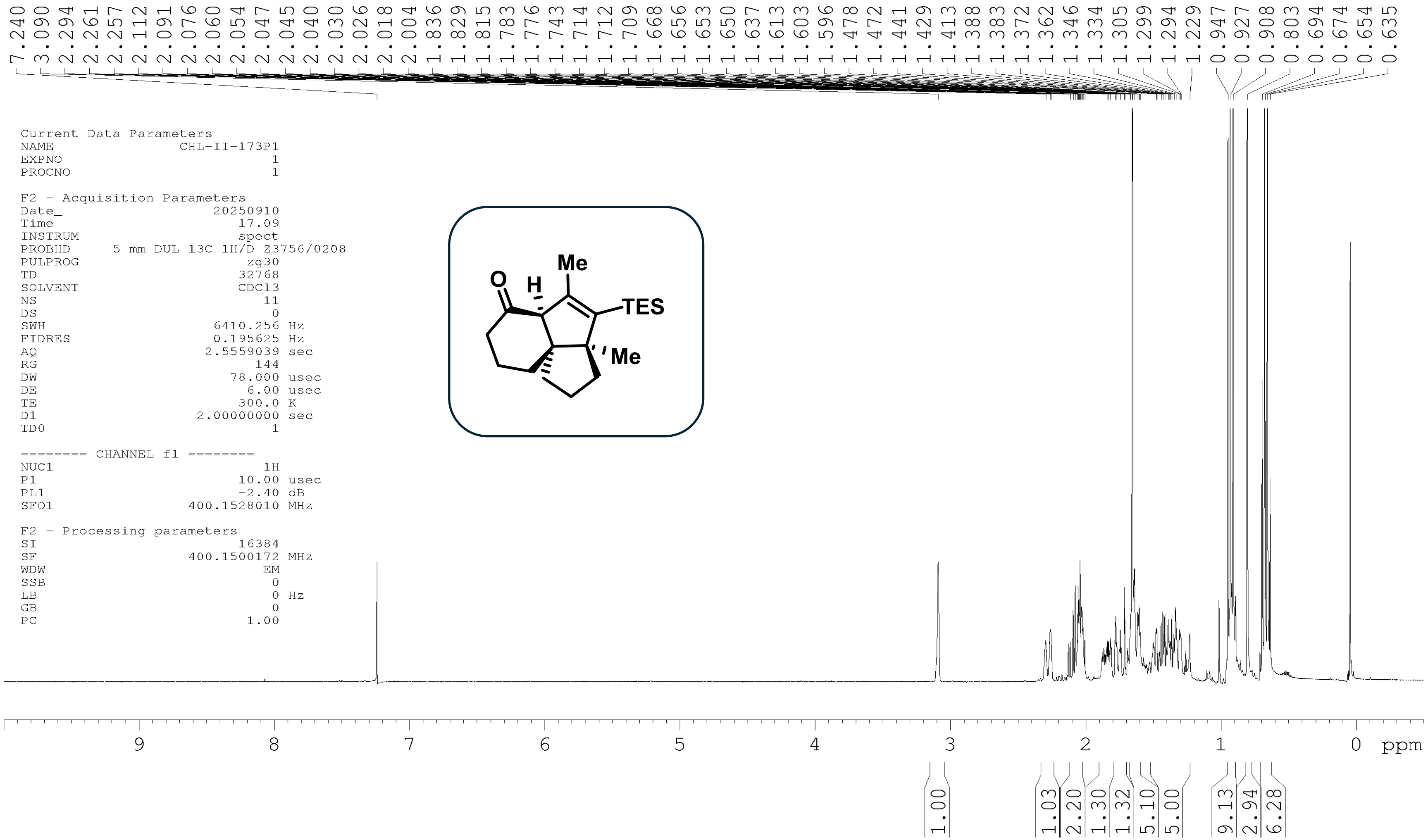

Figure 126. ^{1}H NMR (400 MHz, $CDCl_3$) (**28k-*cis,trans***)

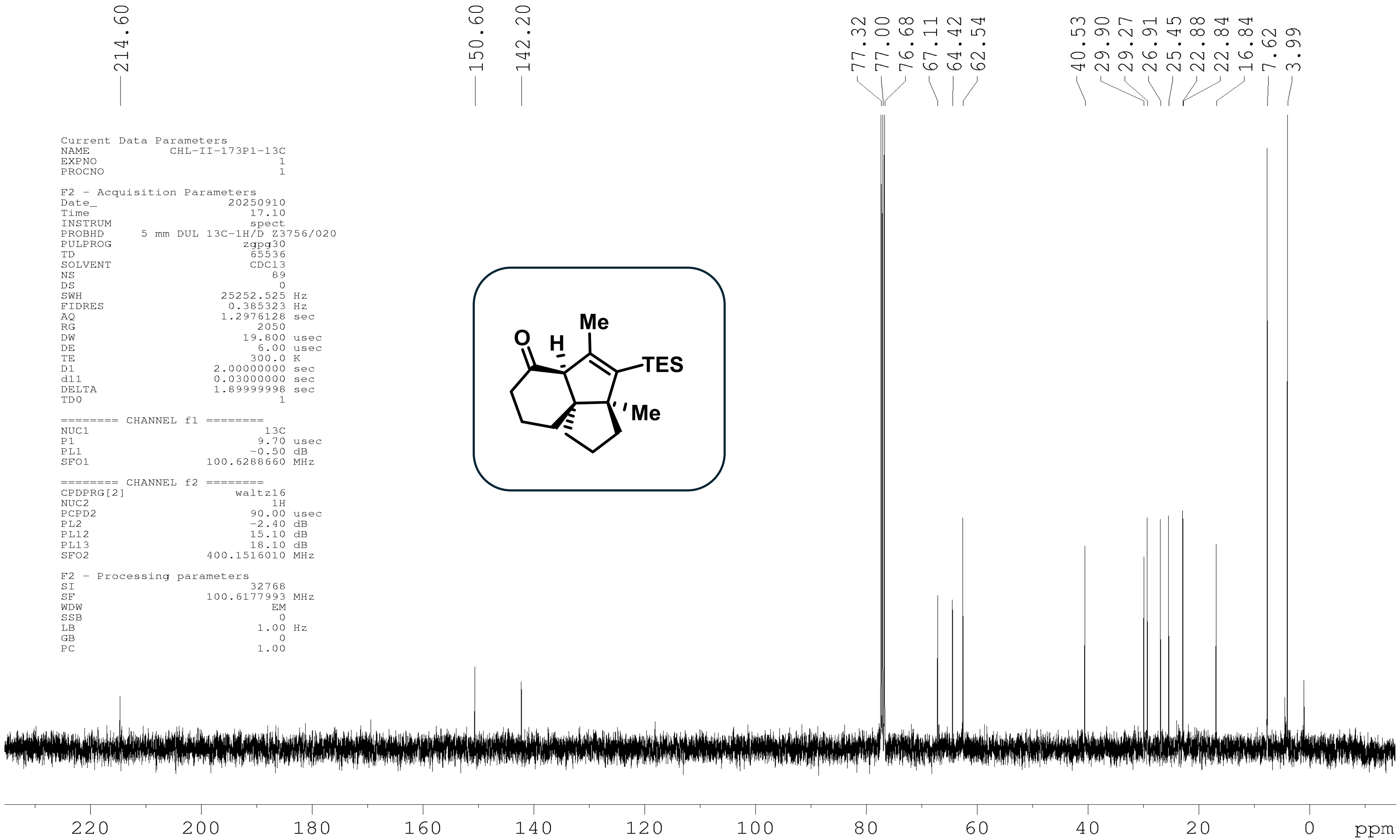

Figure 127. ^{13}C NMR (100 MHz, $CDCl_3$) (**28k-*cis,trans***)

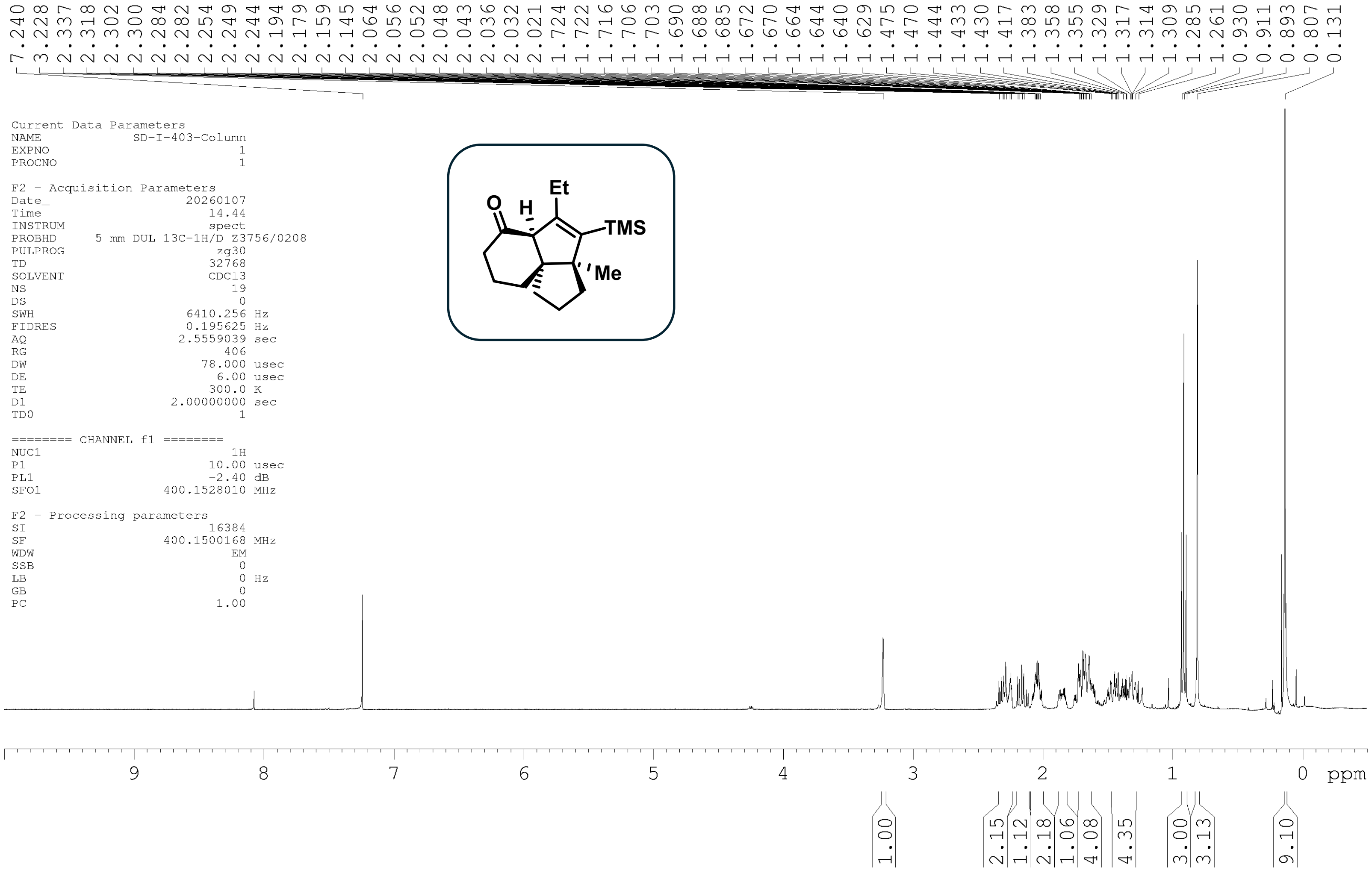

Figure 128. ^{1}H NMR (400 MHz, CDCl$_3$) (**28l-*cis*,*trans***)

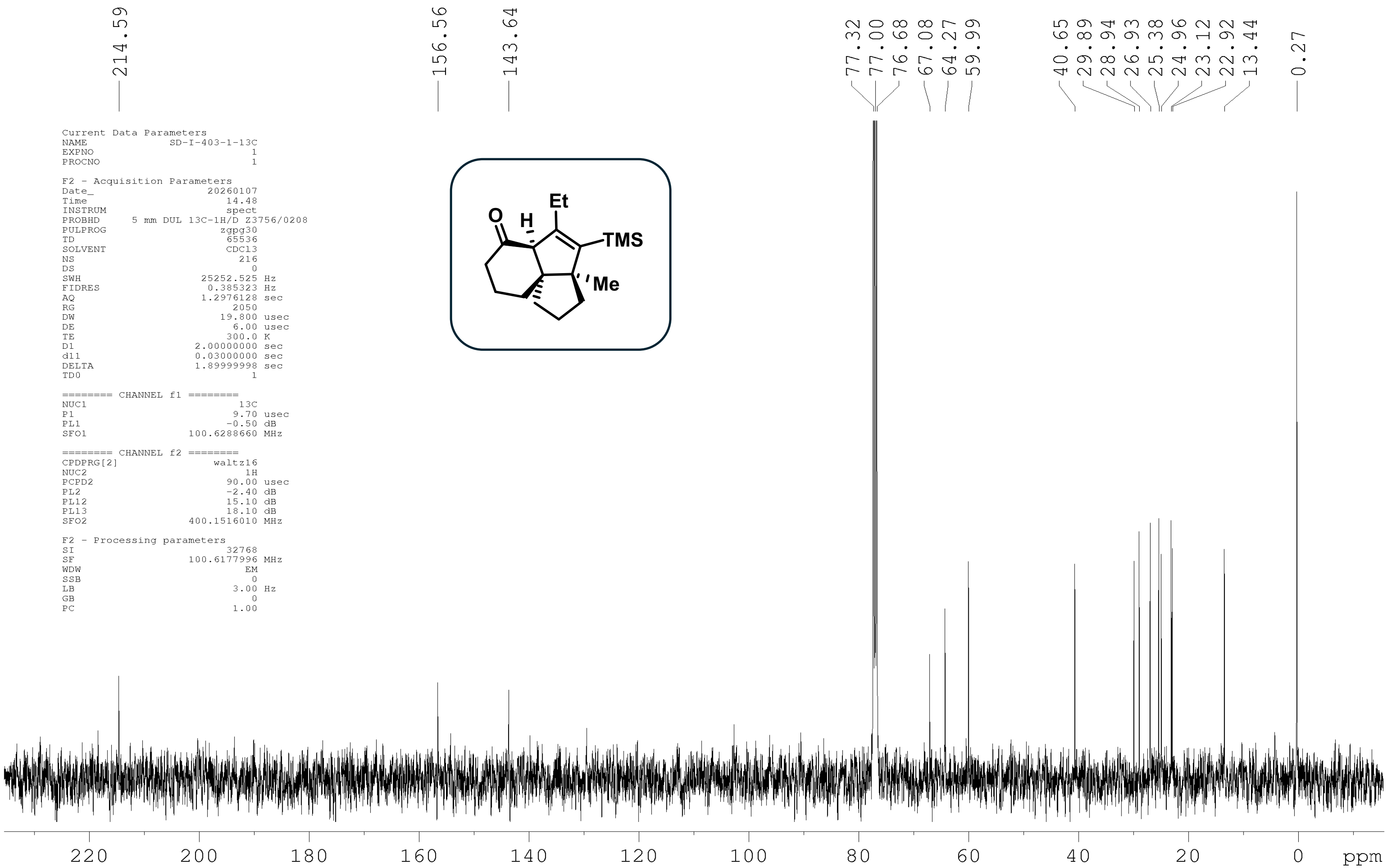

Figure 129. ^{13}C NMR (100 MHz, $CDCl_3$) (**28l-*cis*,*trans***)

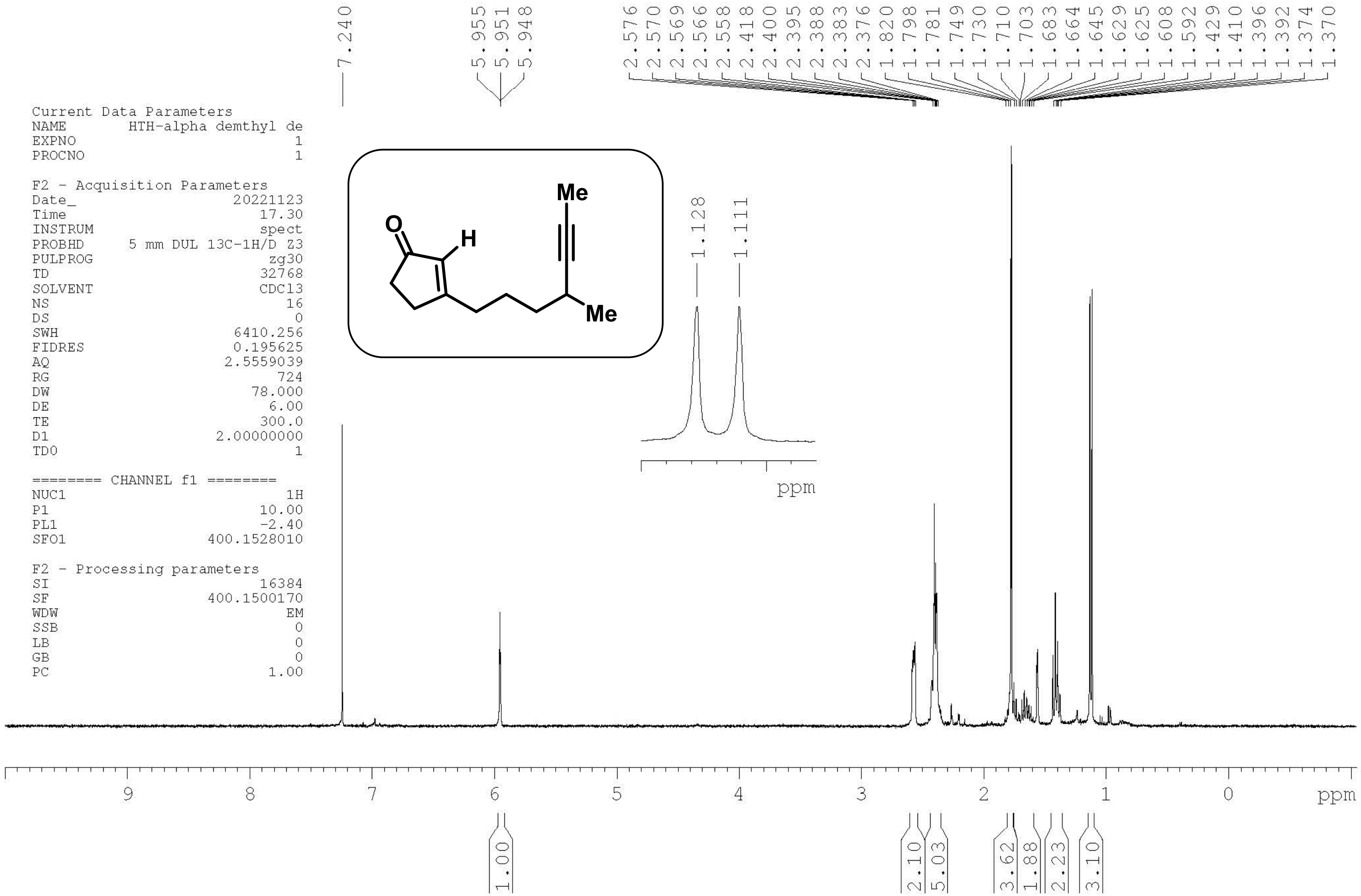

Figure 130. ^{1}H NMR (400 MHz, $CDCl_3$) (**29a**)

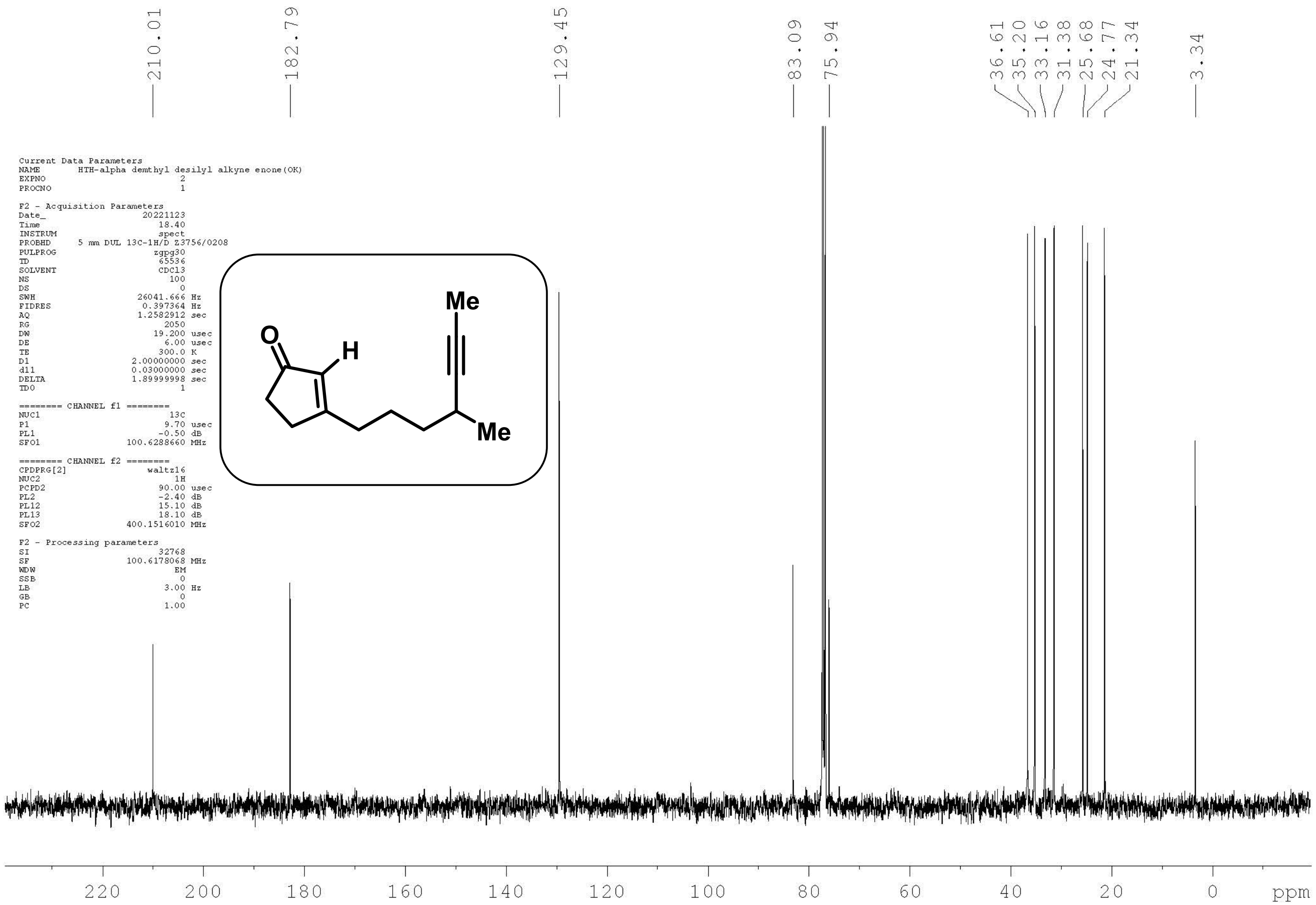

Figure 131. ^{13}C NMR (100 MHz, $CDCl_3$) (**29a**)

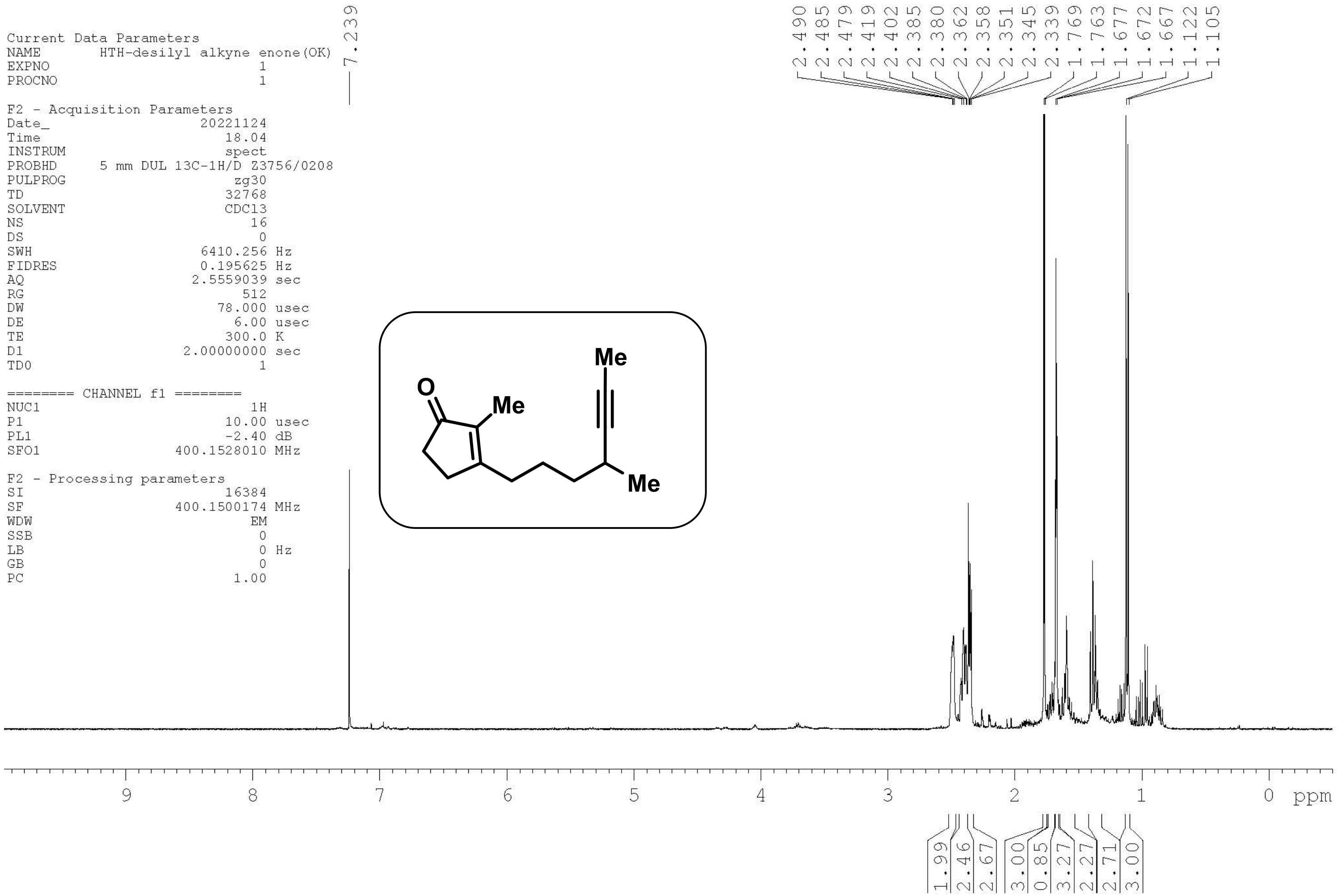

Figure 132. ^{1}H NMR (400 MHz, $CDCl_3$) (**29b**)

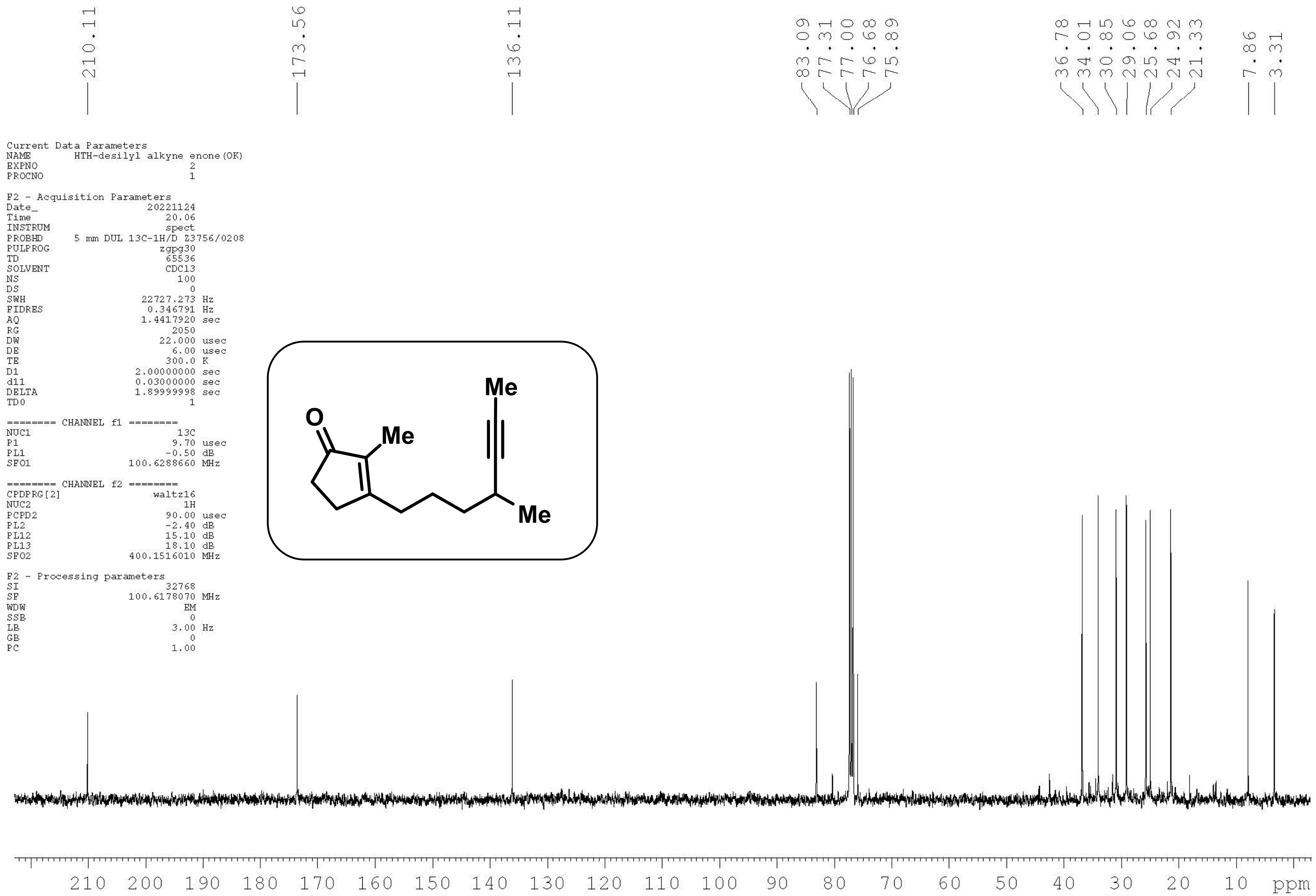

Figure 133. ^{13}C NMR (100 MHz, $CDCl_3$) (**29b**)

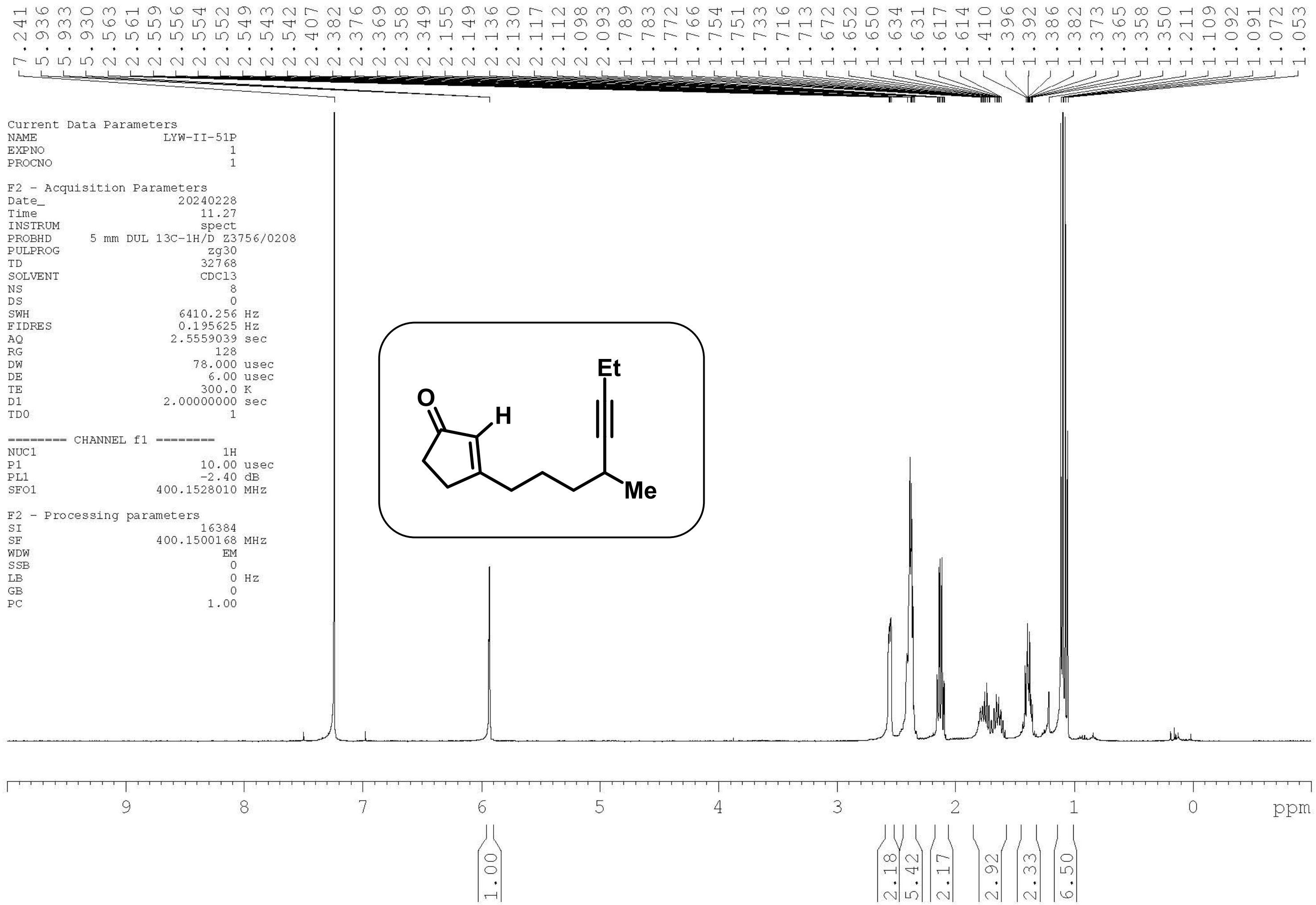

Figure 134. ^{1}H NMR (400 MHz, $CDCl_3$) (**29c**)

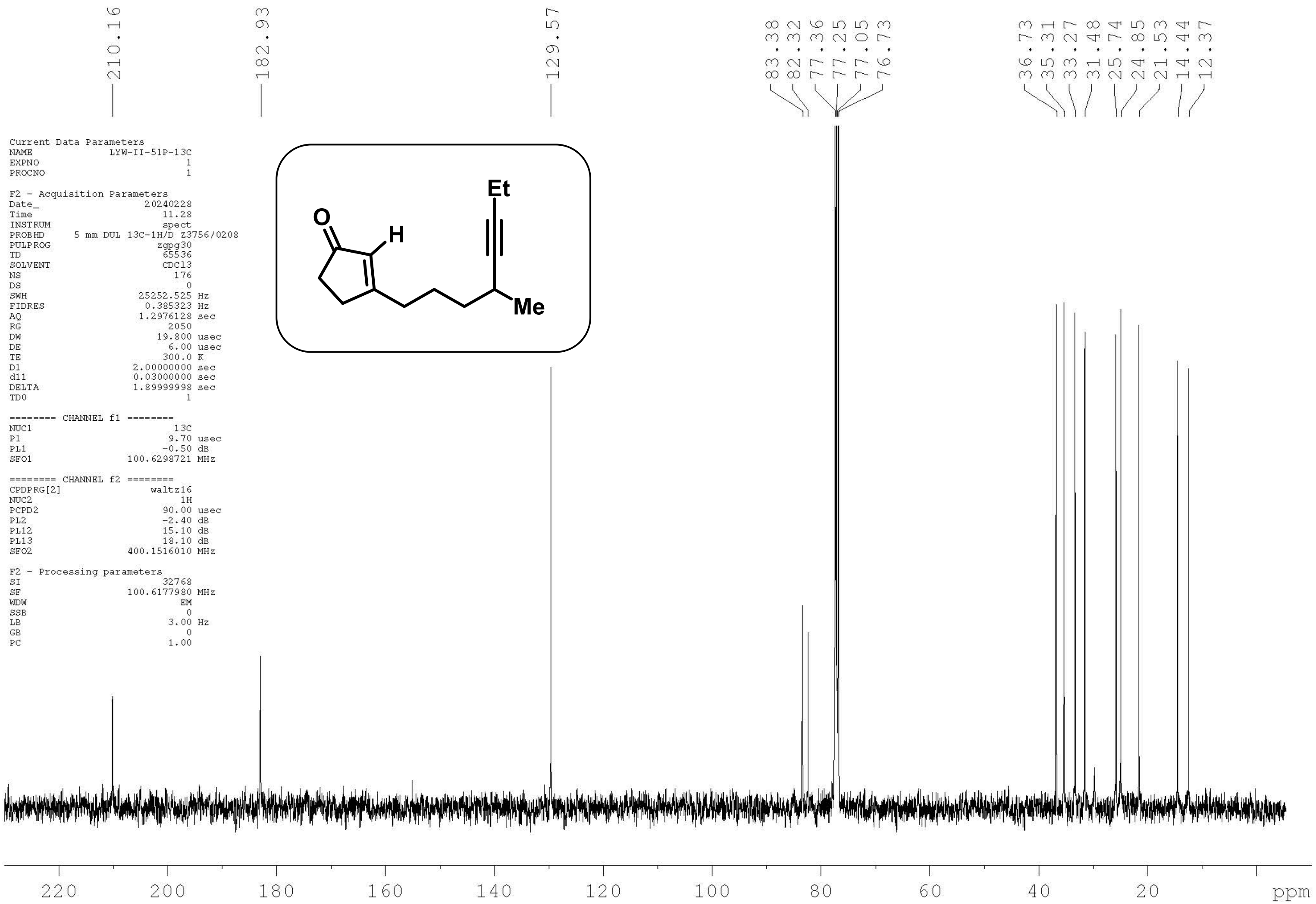

Figure 135. ^{13}C NMR (100 MHz, $CDCl_3$) (**29c**)

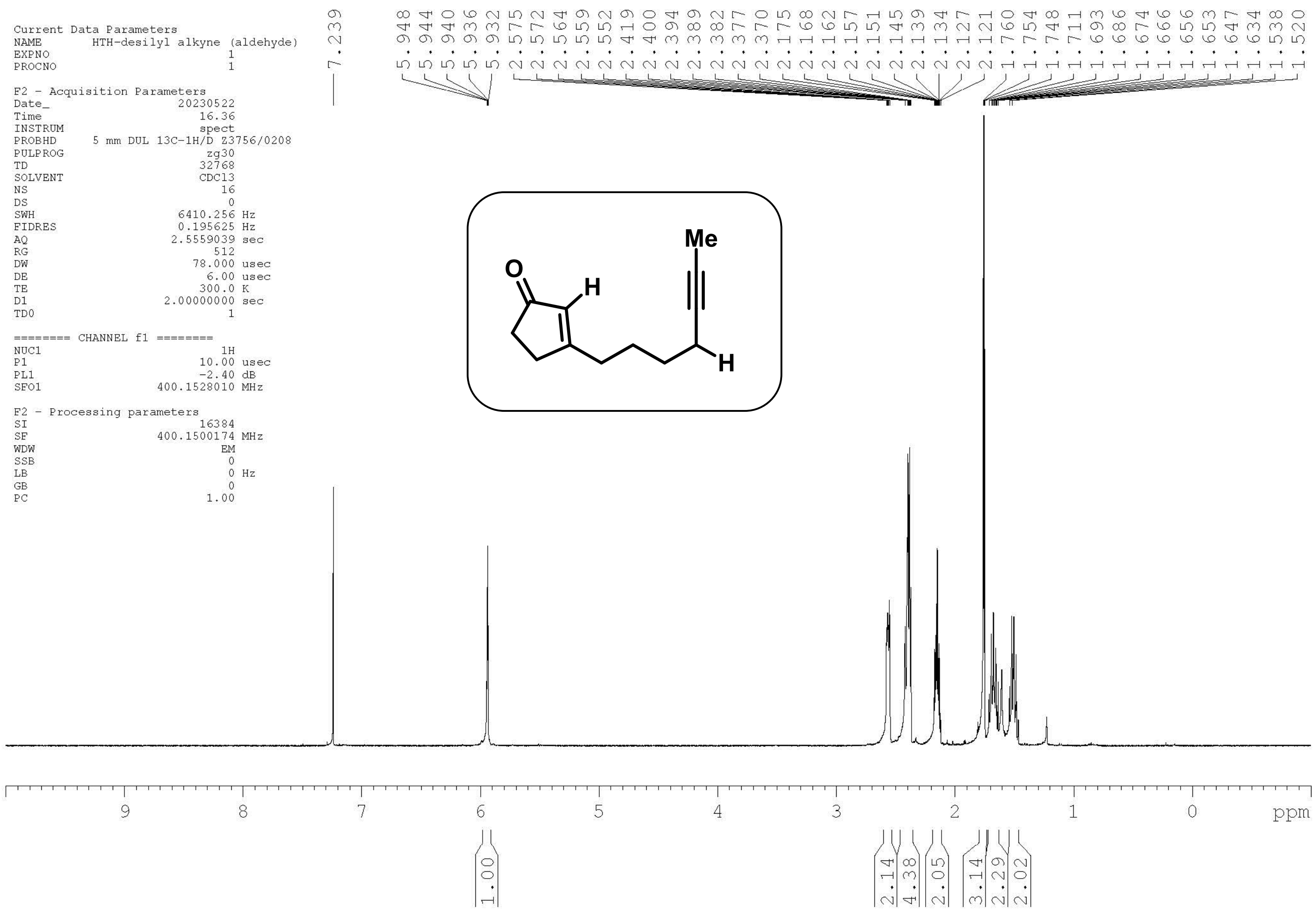

Figure 136. ^{1}H NMR (400 MHz, $CDCl_3$) (**29d**)

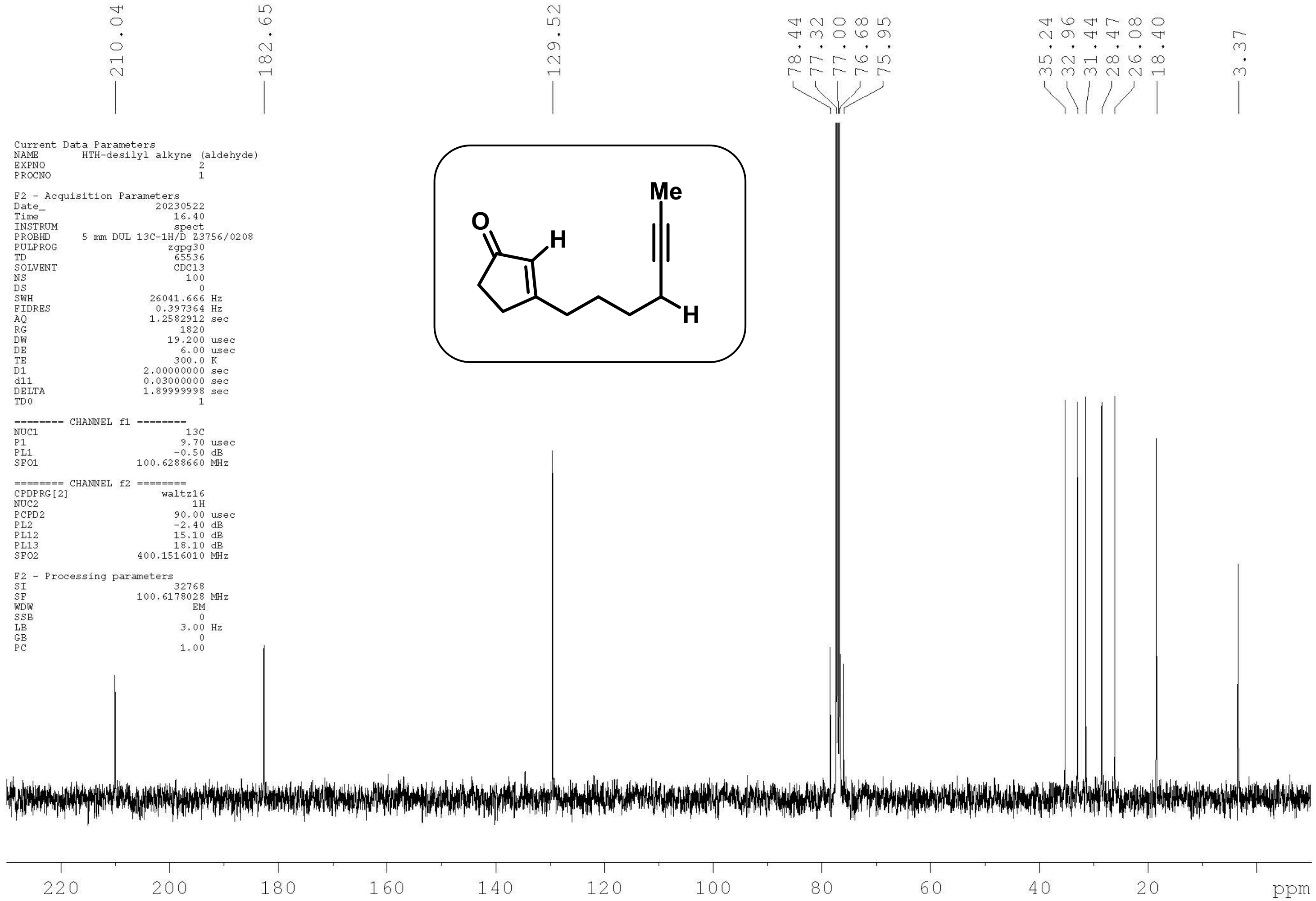

Figure 137. ^{13}C NMR (100 MHz, $CDCl_3$) (**29d**)

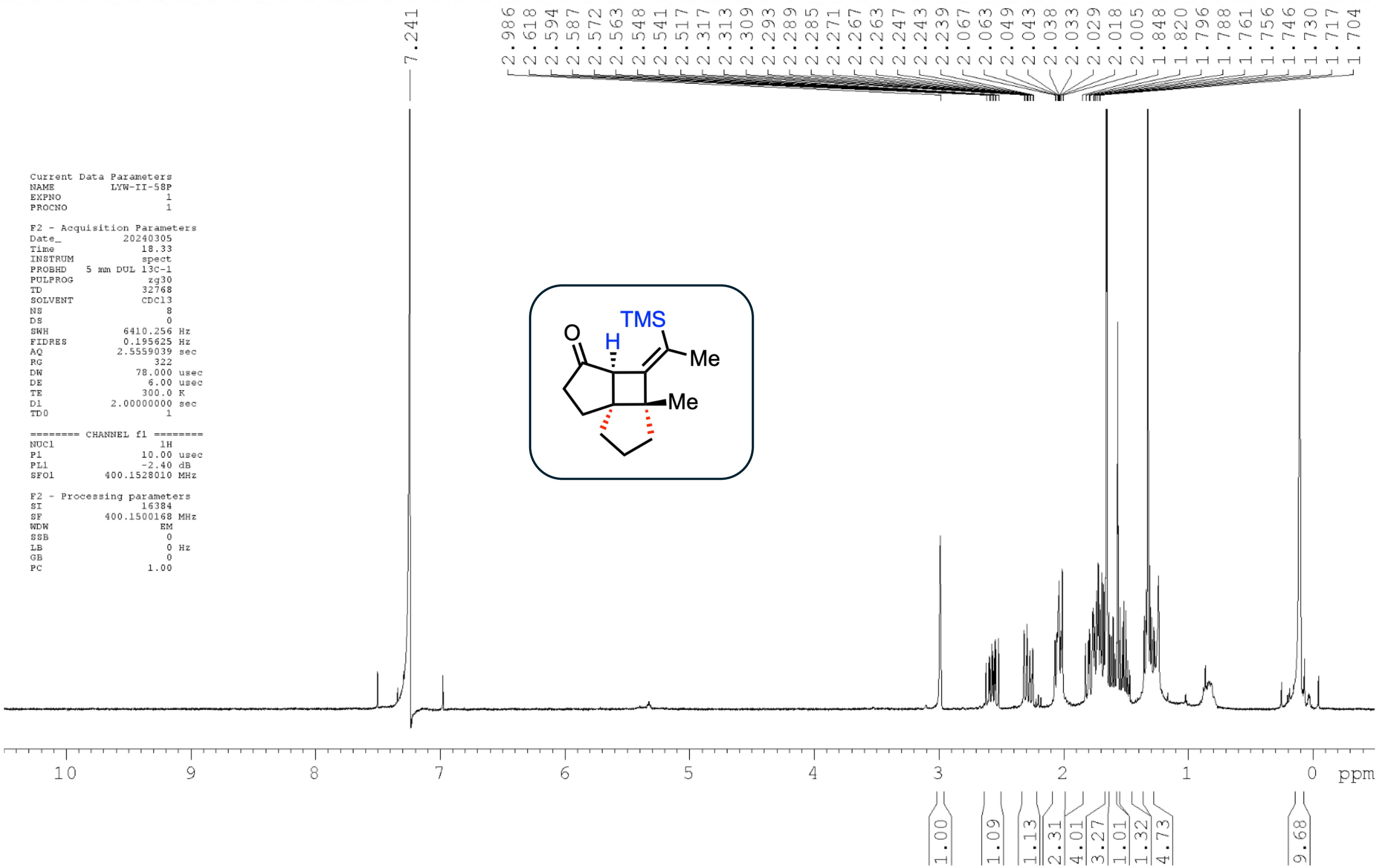

Figure 138. ^{1}H NMR (400 MHz, $CDCl_3$) (**30a**)

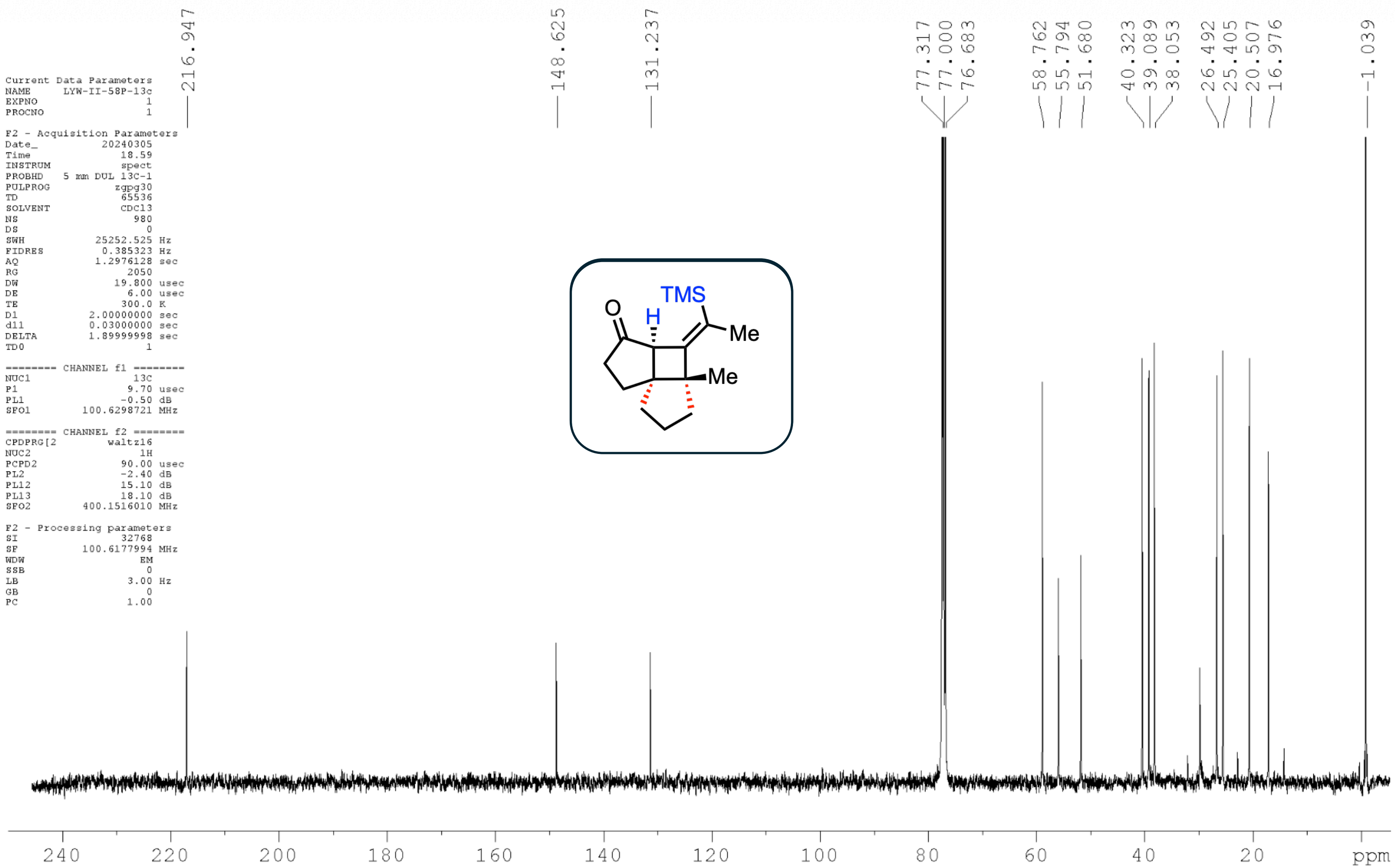

Figure 139. ^{13}C NMR (100 MHz, $CDCl_3$) (**30a**)

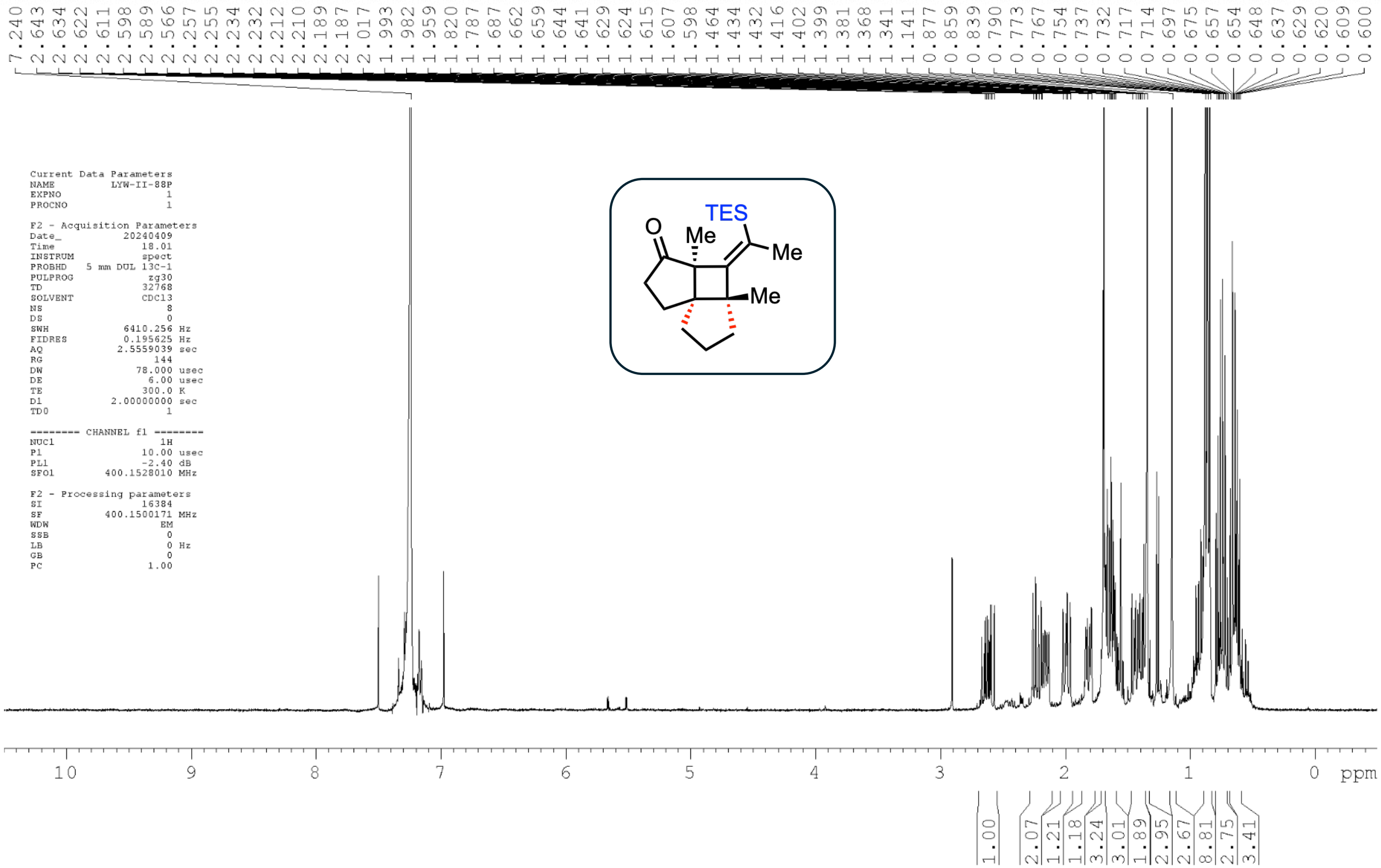

Figure 140. ^{1}H NMR (400 MHz, $CDCl_3$) (**30b**)

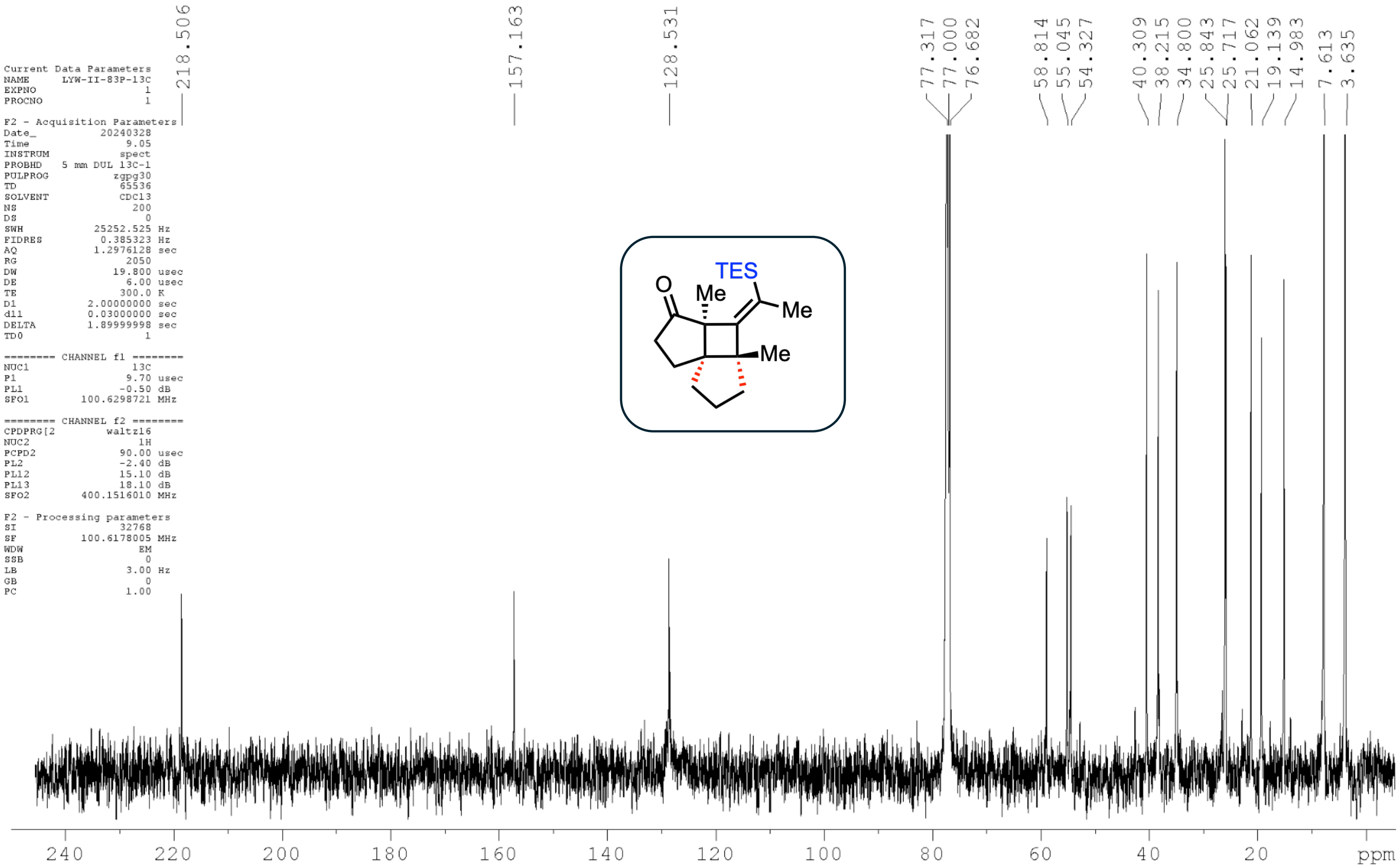

Figure 141. ^{13}C NMR (100 MHz, $CDCl_3$) (**30b**)

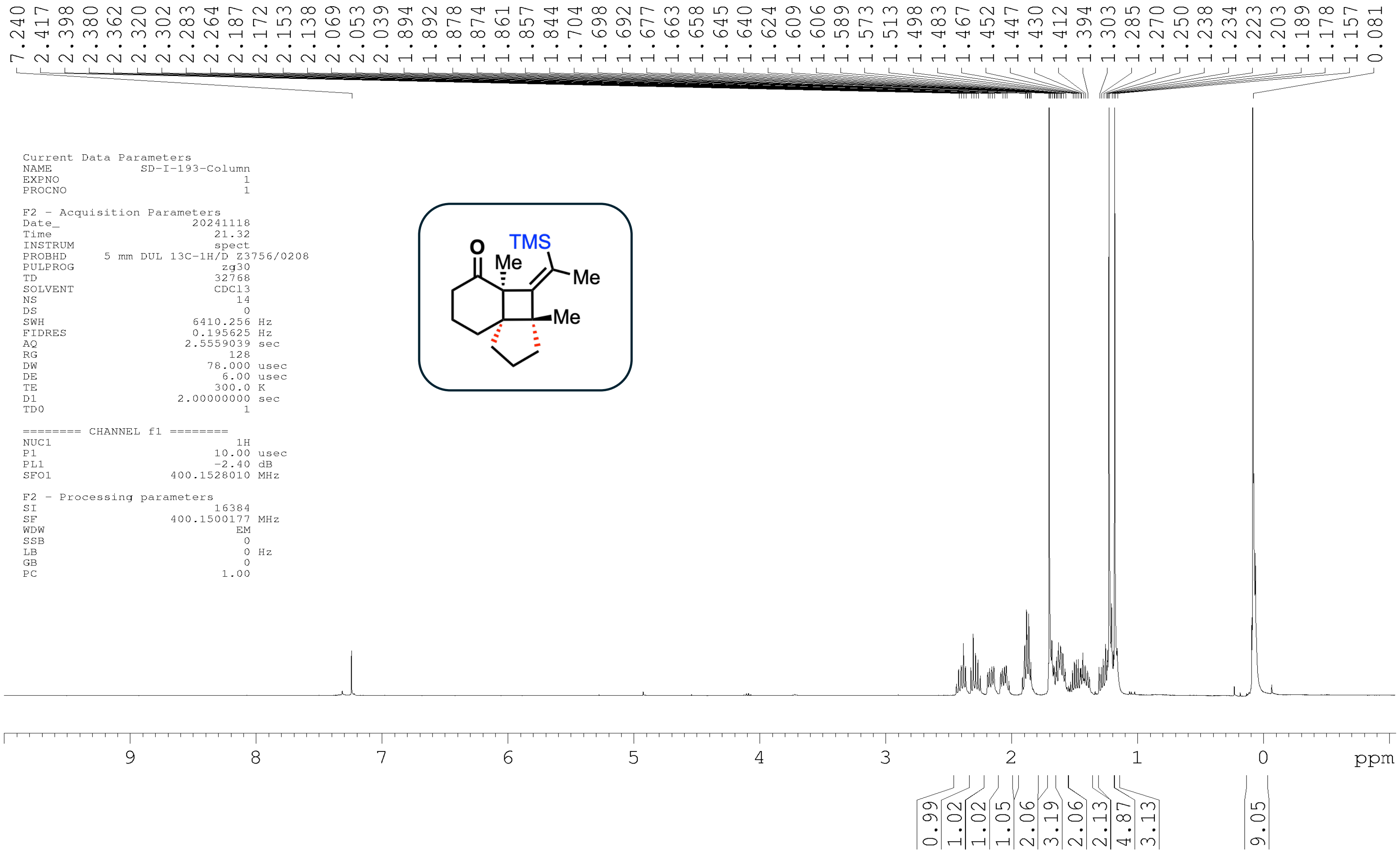

Figure 142. ^{1}H NMR (400 MHz, $CDCl_3$) (**30c**)

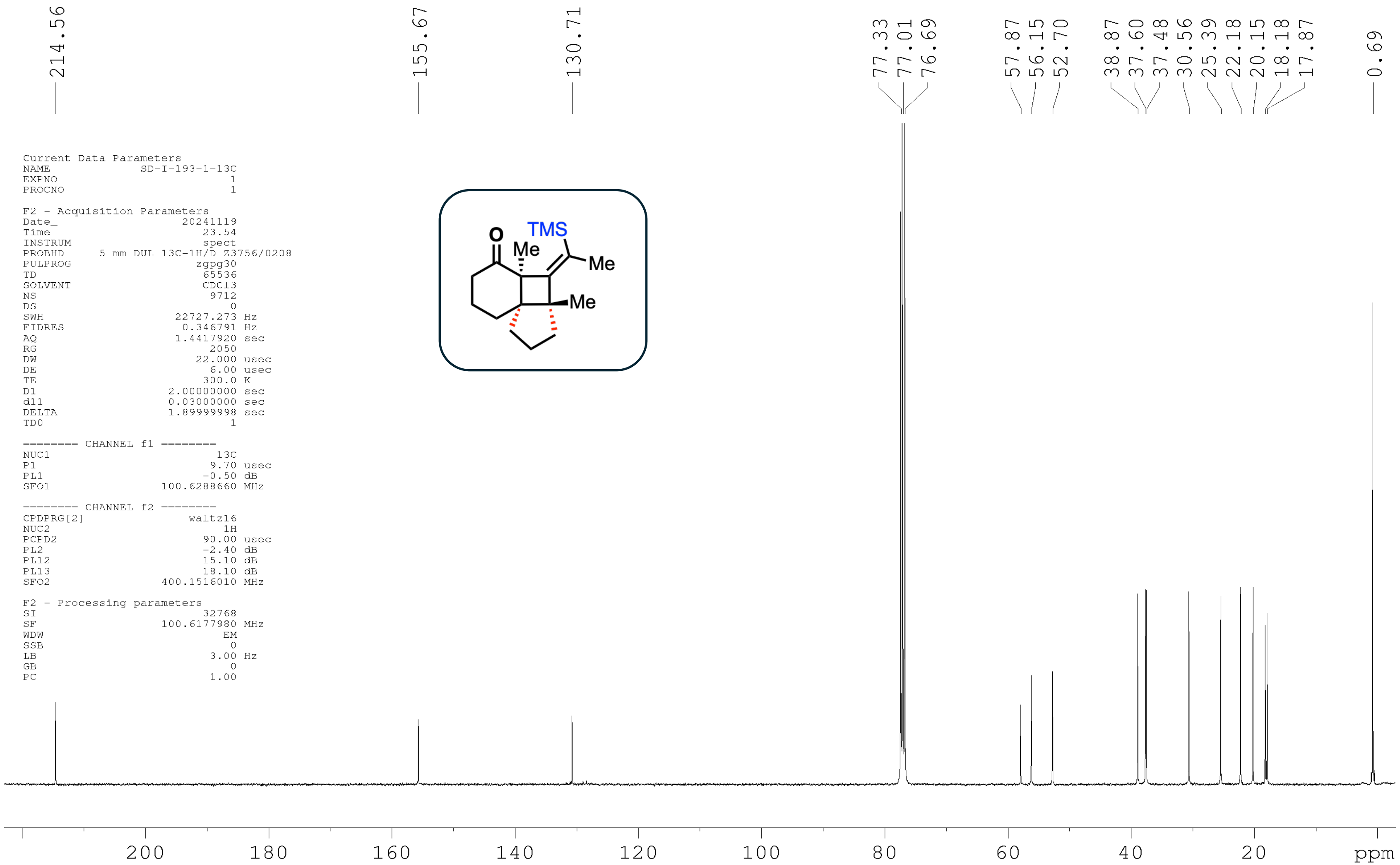

Figure 143. ^{13}C NMR (100 MHz, CDCl$_3$) (**30c**)

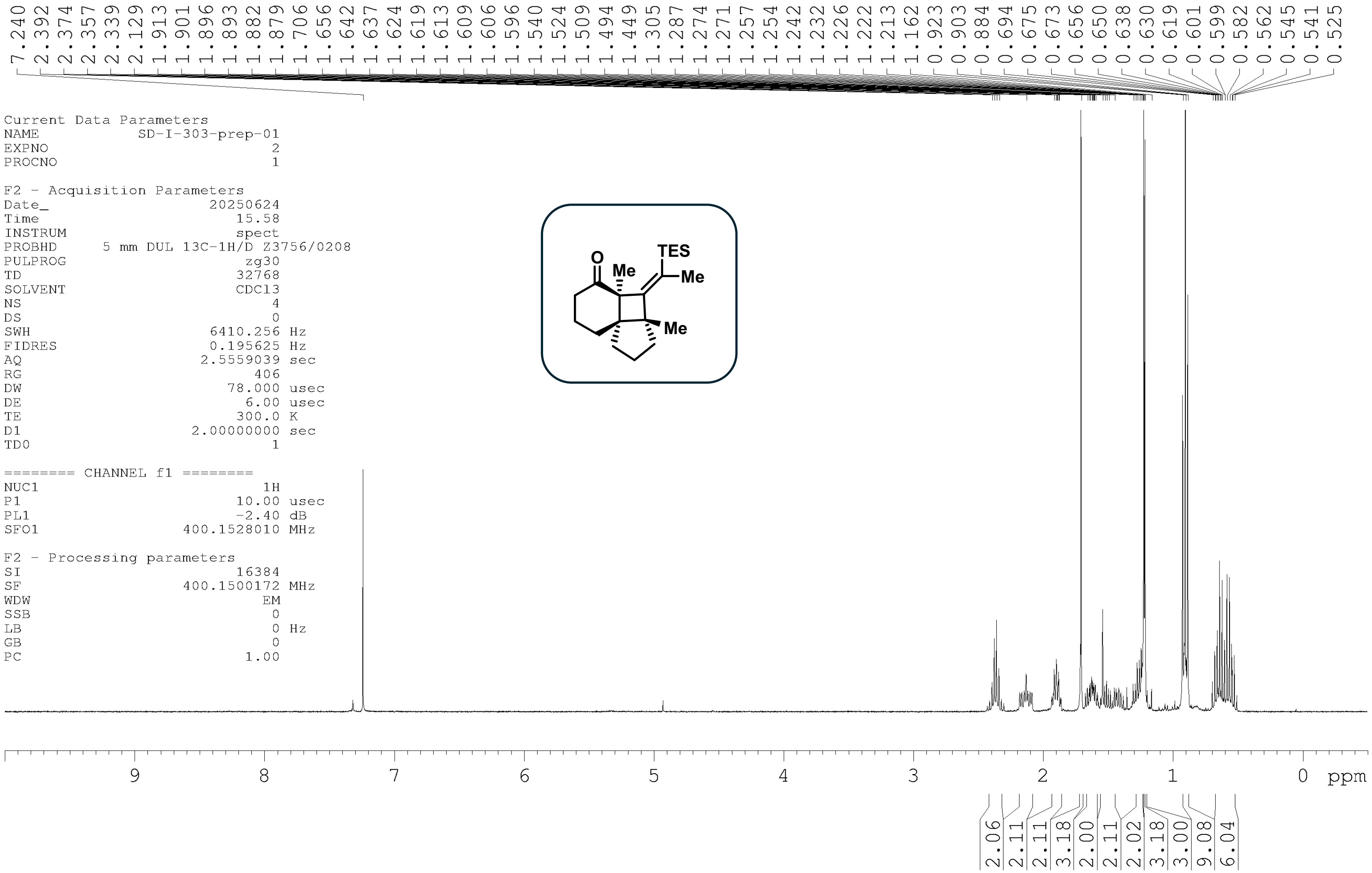

Figure 144. ^{1}H NMR (400 MHz, $CDCl_3$) (**30d**)

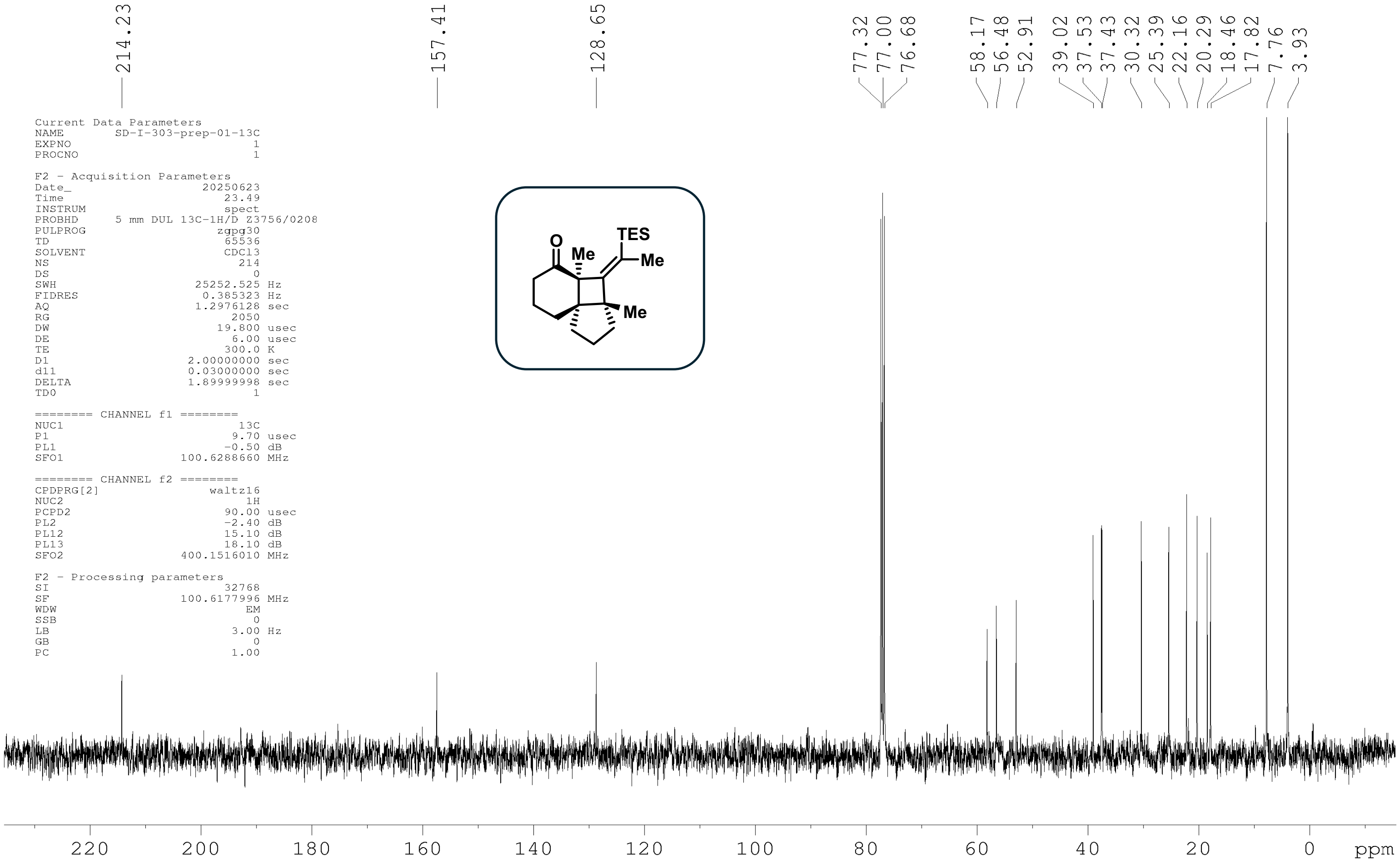

Figure 145. ^{13}C NMR (100 MHz, $CDCl_3$) (**30d**)

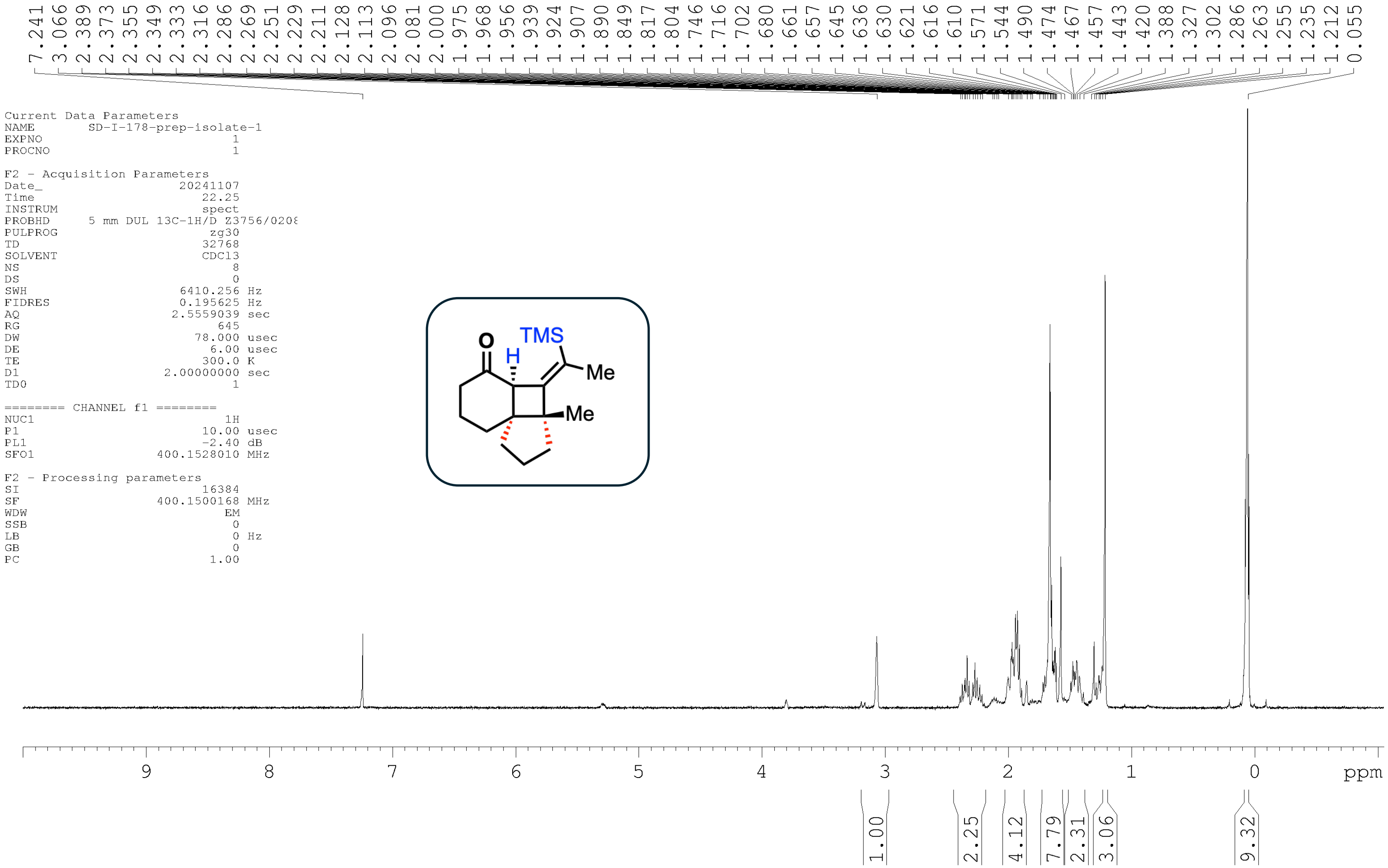

Figure 146. ^{1}H NMR (400 MHz, $CDCl_3$) (**30e**)

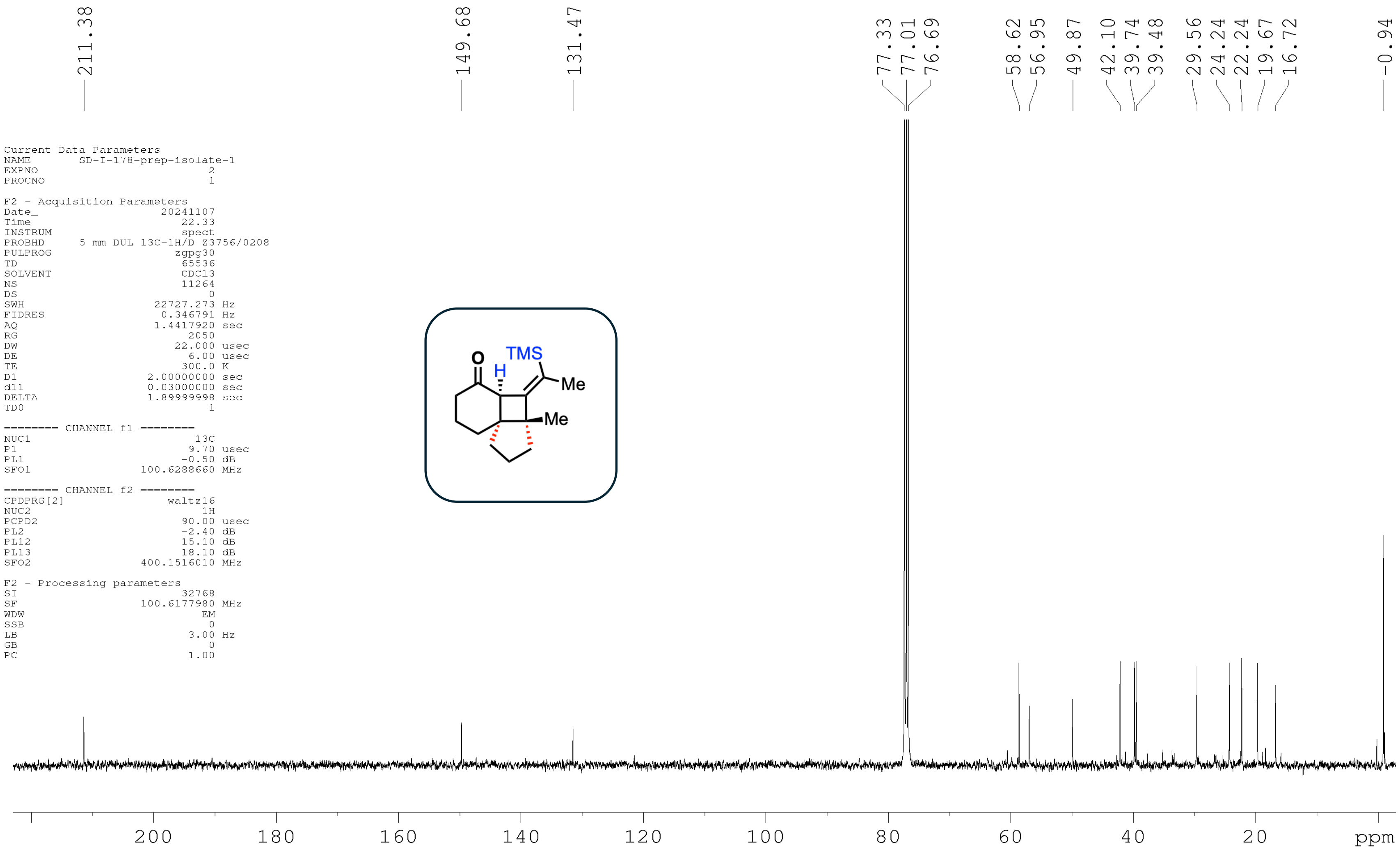

Figure 147. ^{13}C NMR (100 MHz, $CDCl_3$) (**30e**)

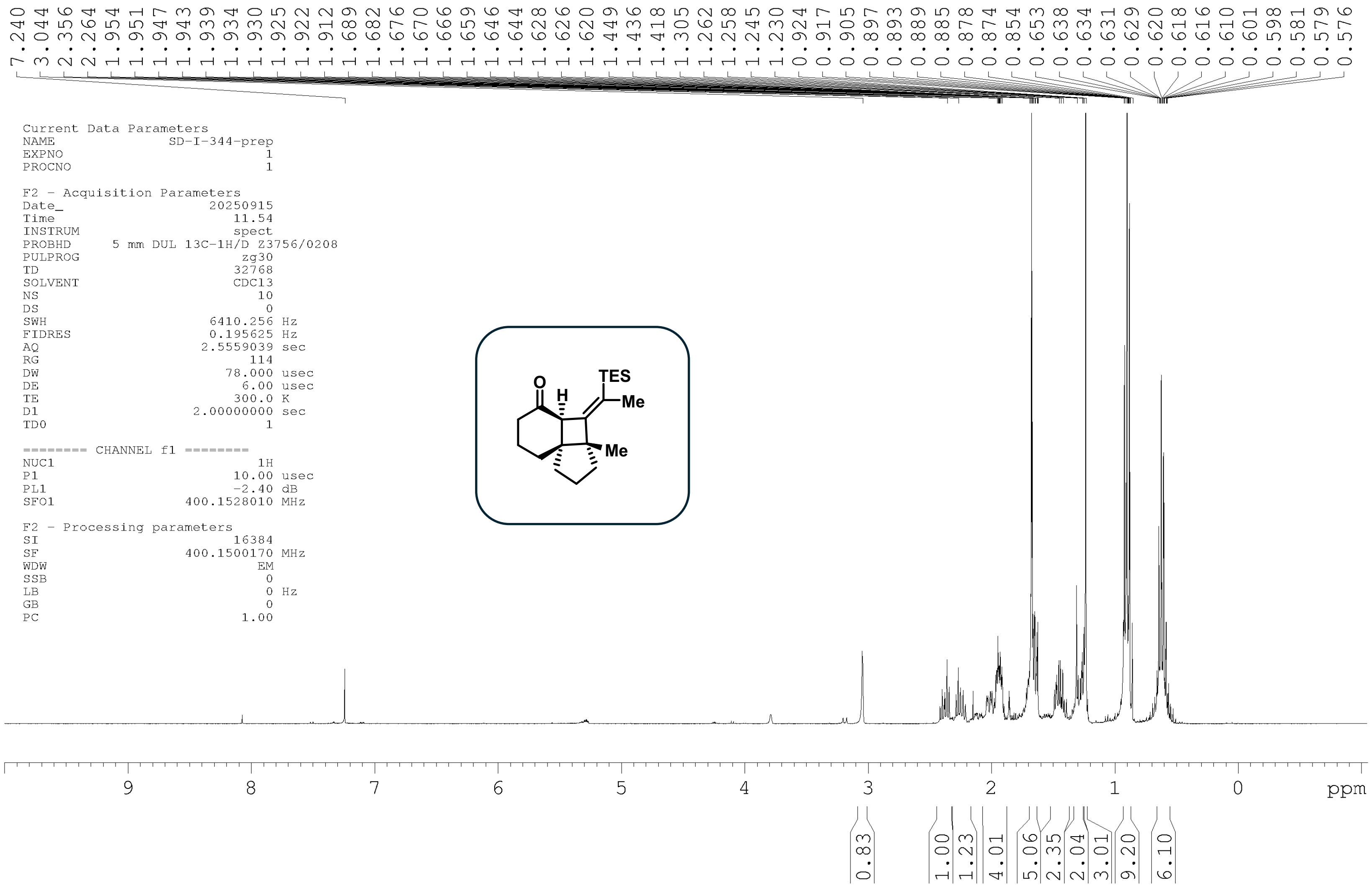

Figure 148. ^{1}H NMR (400 MHz, $CDCl_3$) (**30f**)

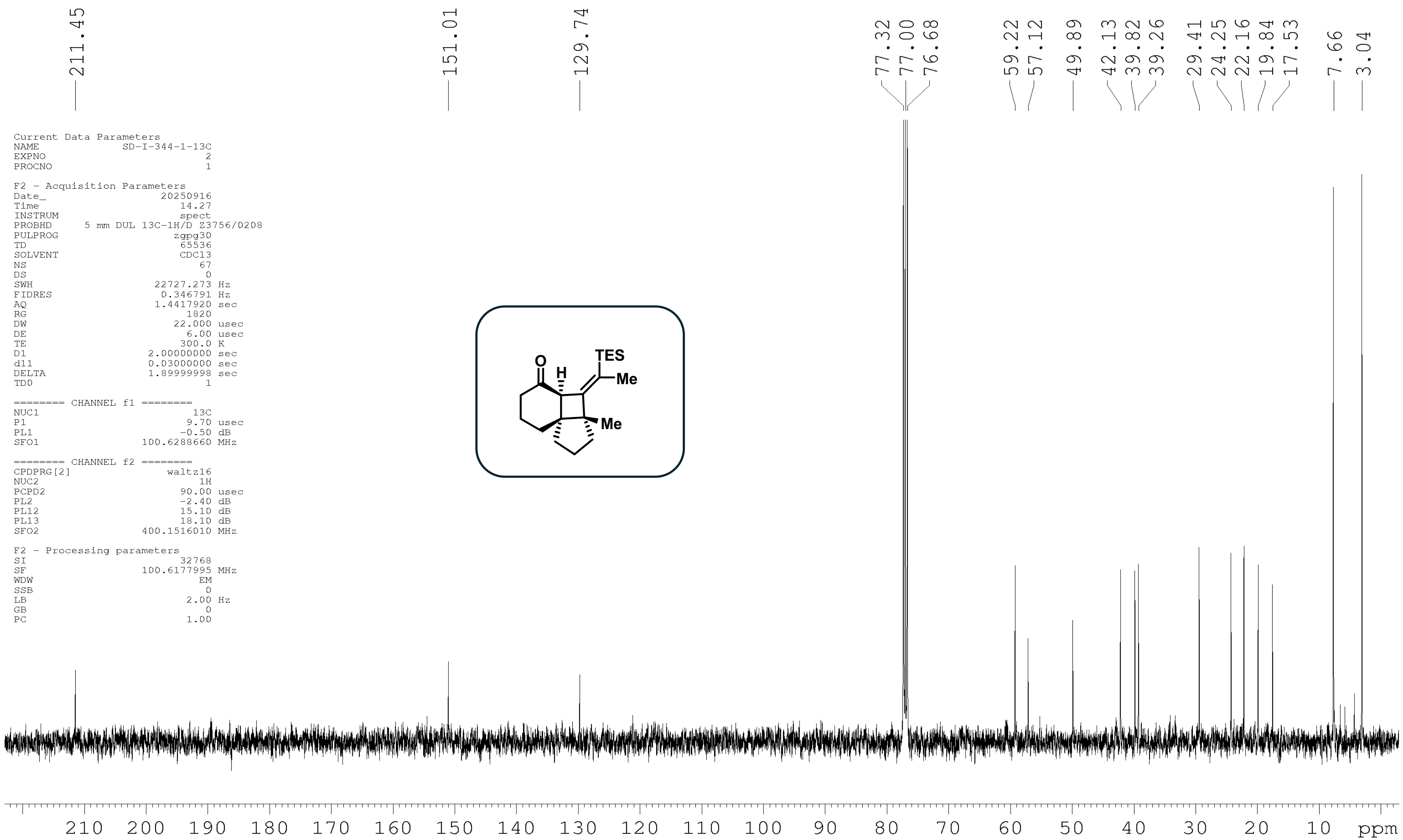

Figure 149. ^{13}C NMR (100 MHz, $CDCl_3$) (**30f**)

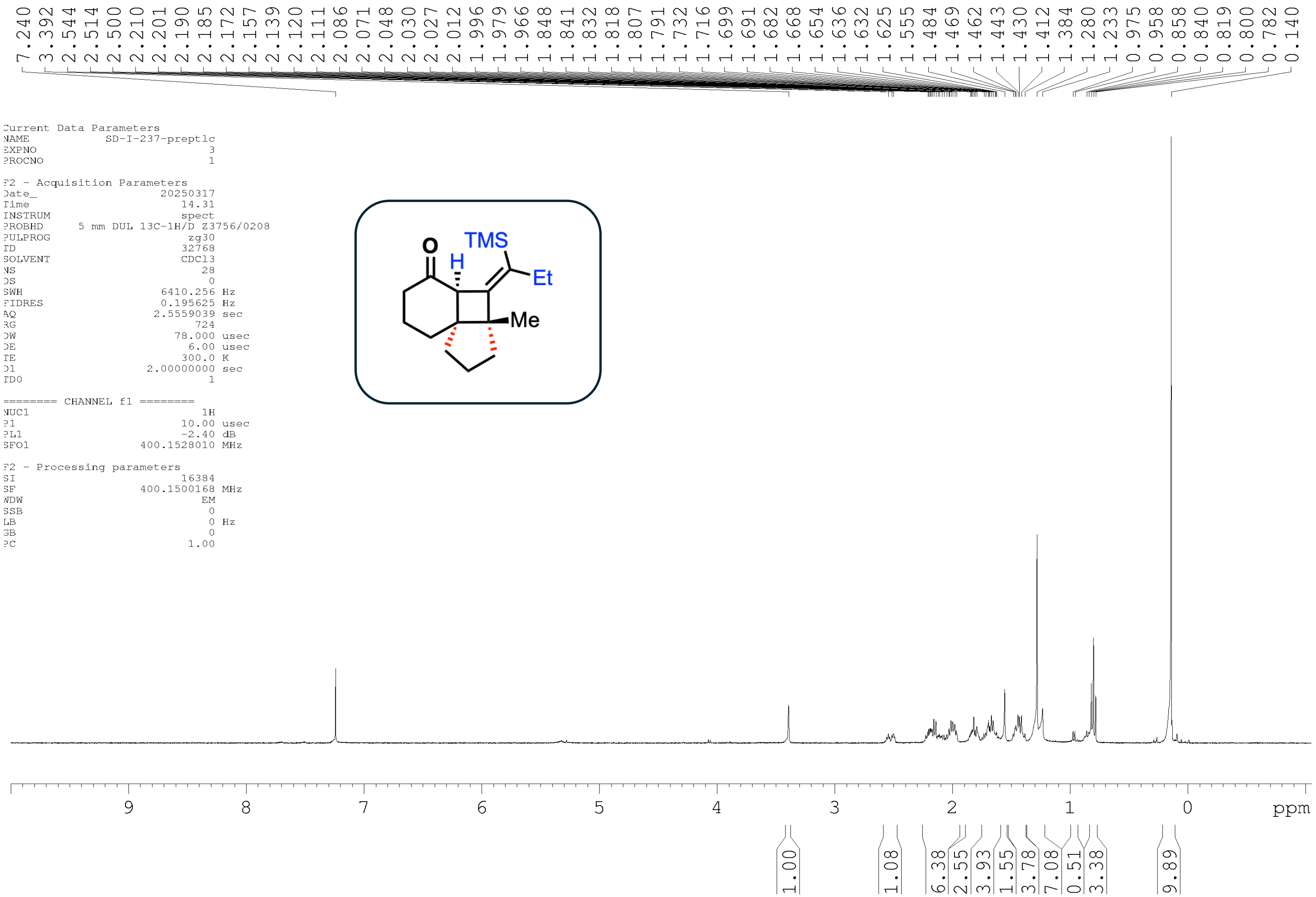

Figure 150. ^{1}H NMR (400 MHz, $CDCl_3$) (**30g**)

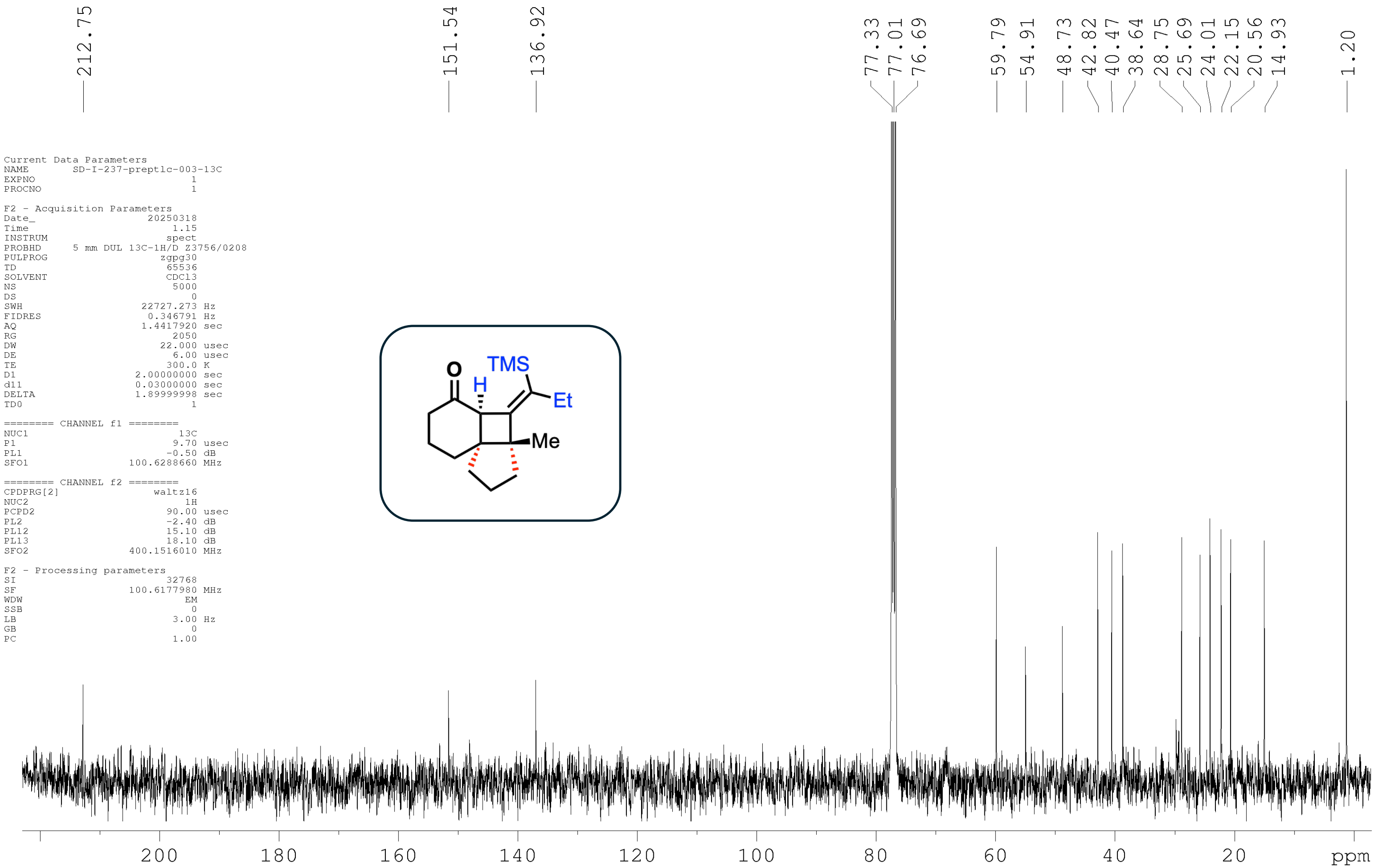

Figure 151. ^{13}C NMR (100 MHz, CDCl$_3$) (**30g**)

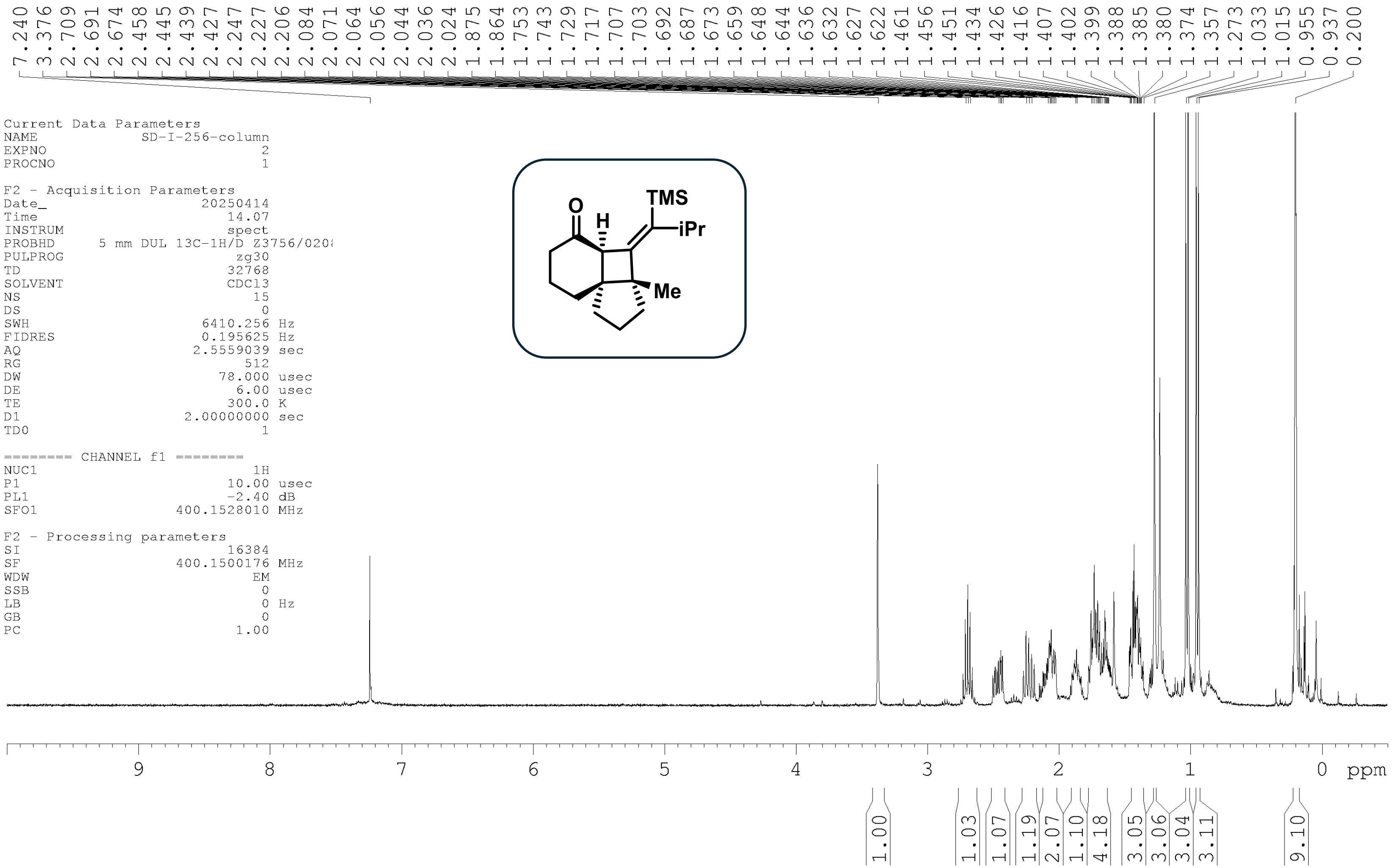

Figure 152. ^{1}H NMR (400 MHz, $CDCl_3$) (**30h**)

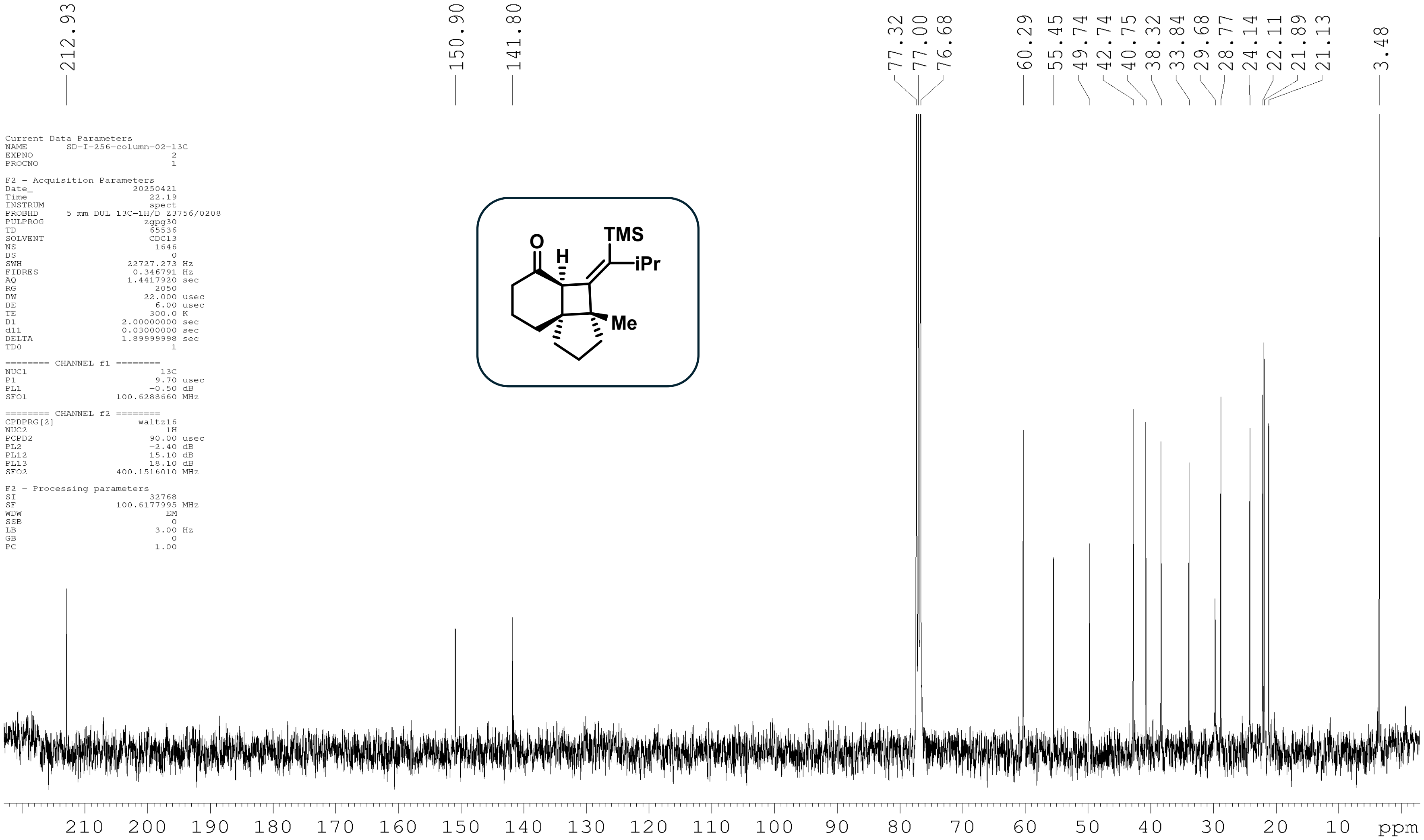

Figure 153. ^{13}C NMR (100 MHz, $CDCl_3$) (**30h**)

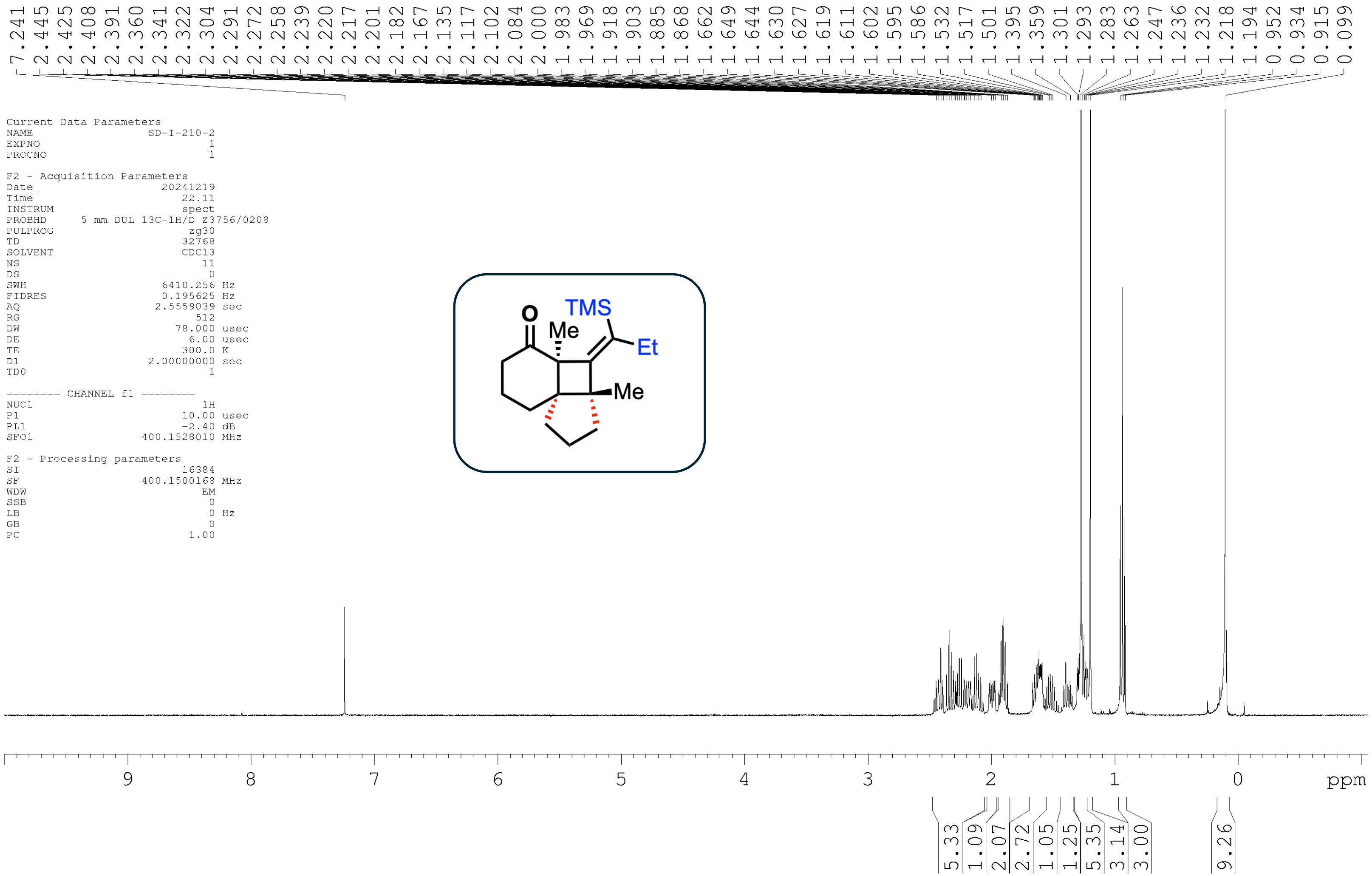

Figure 154. ^{1}H NMR (400 MHz, $CDCl_3$) (**30i**)

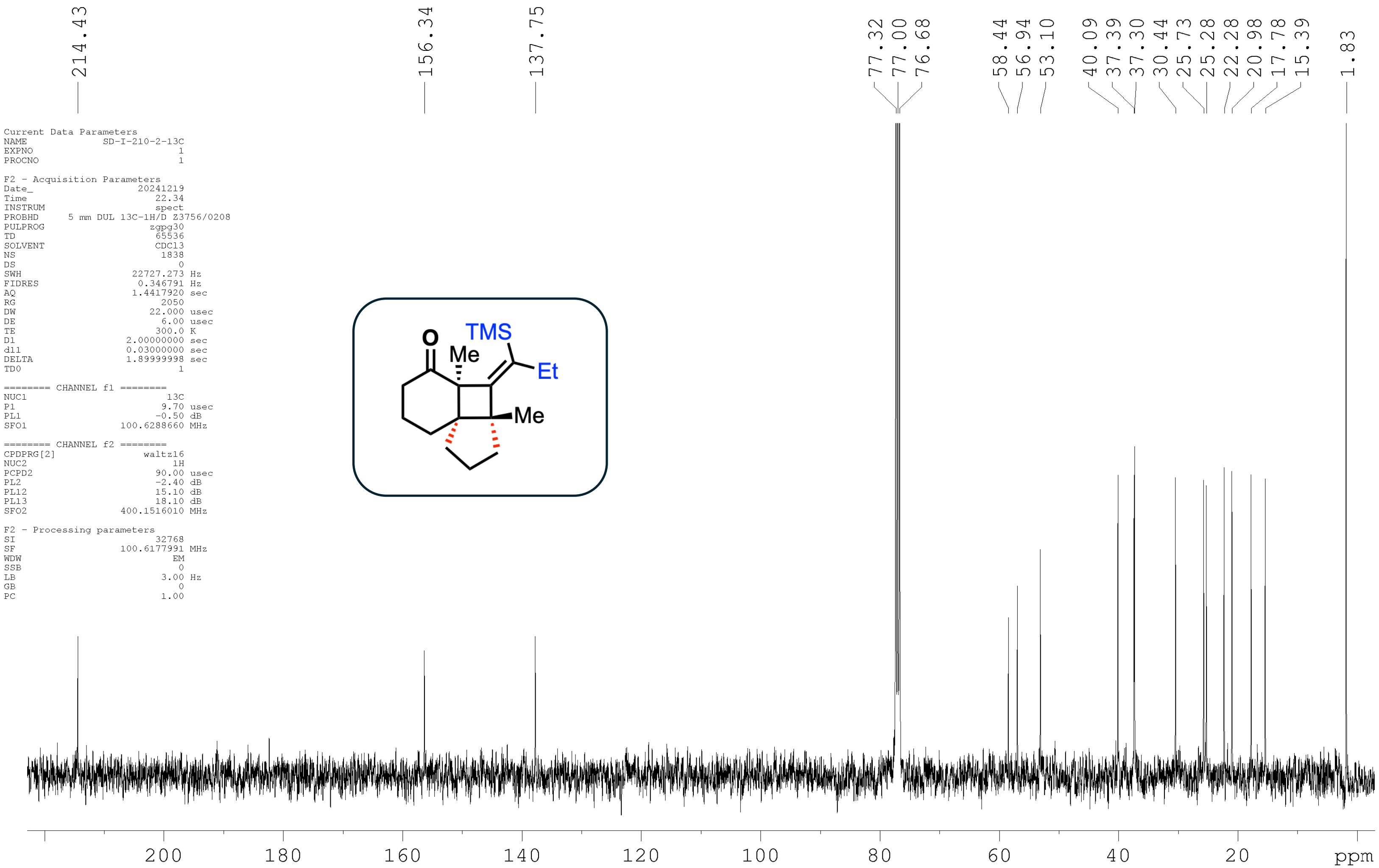

Figure 155. ^{13}C NMR (100 MHz, $CDCl_3$) (**30i**)

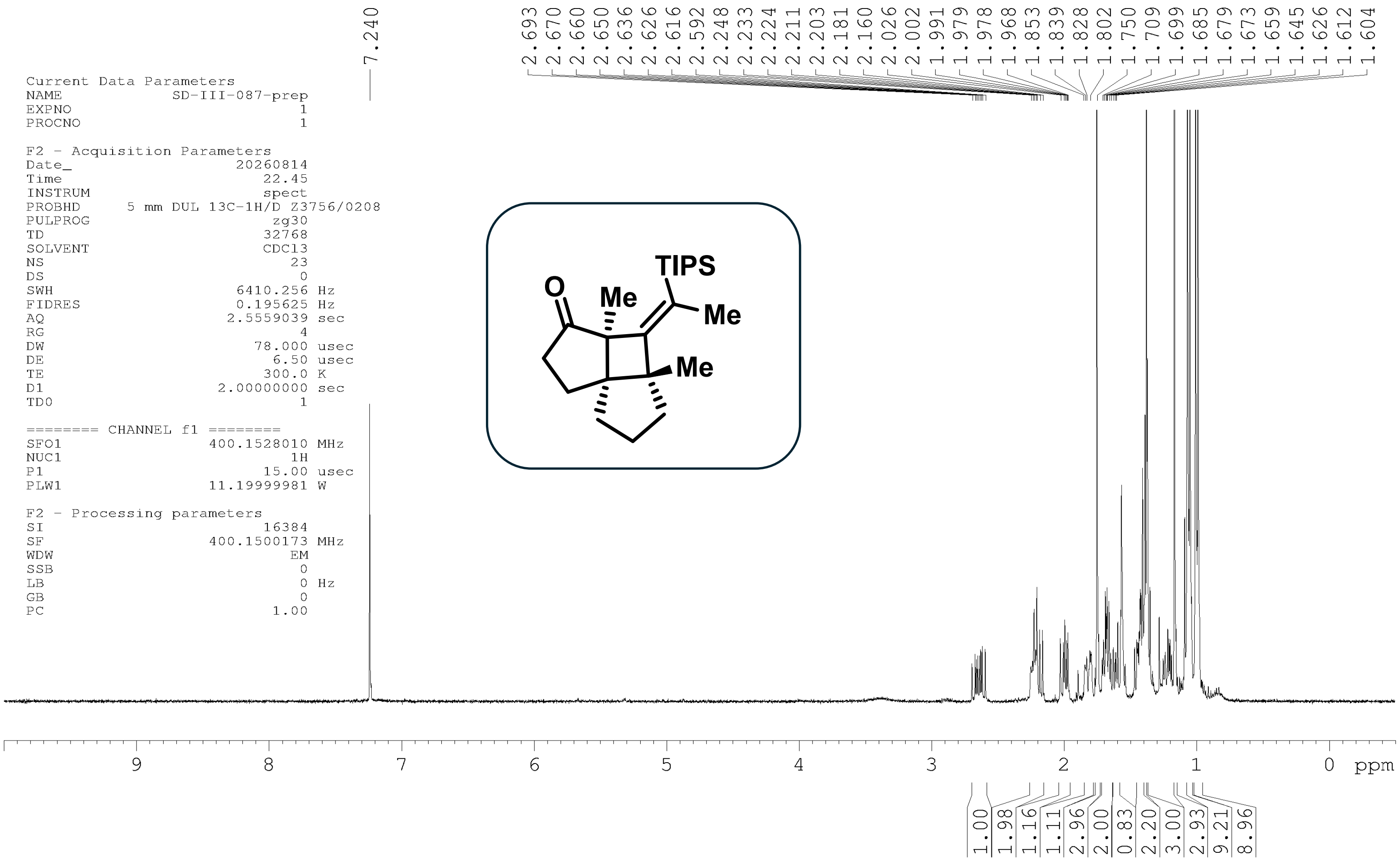

Figure 156. ^{1}H NMR (400 MHz, $CDCl_3$) (**30j**)

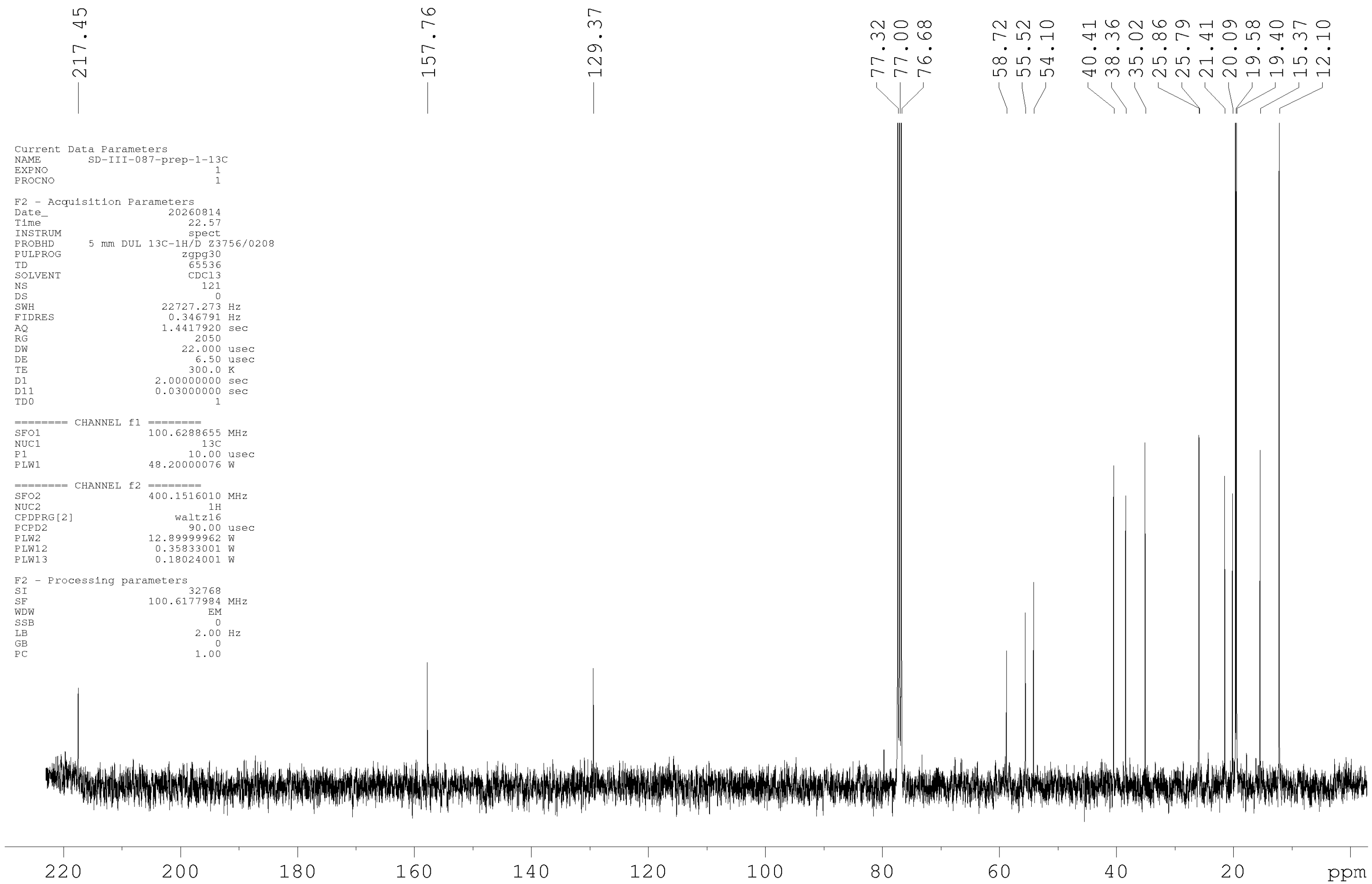

Figure 157. ^{13}C NMR (100 MHz, $CDCl_3$) (**30j**)

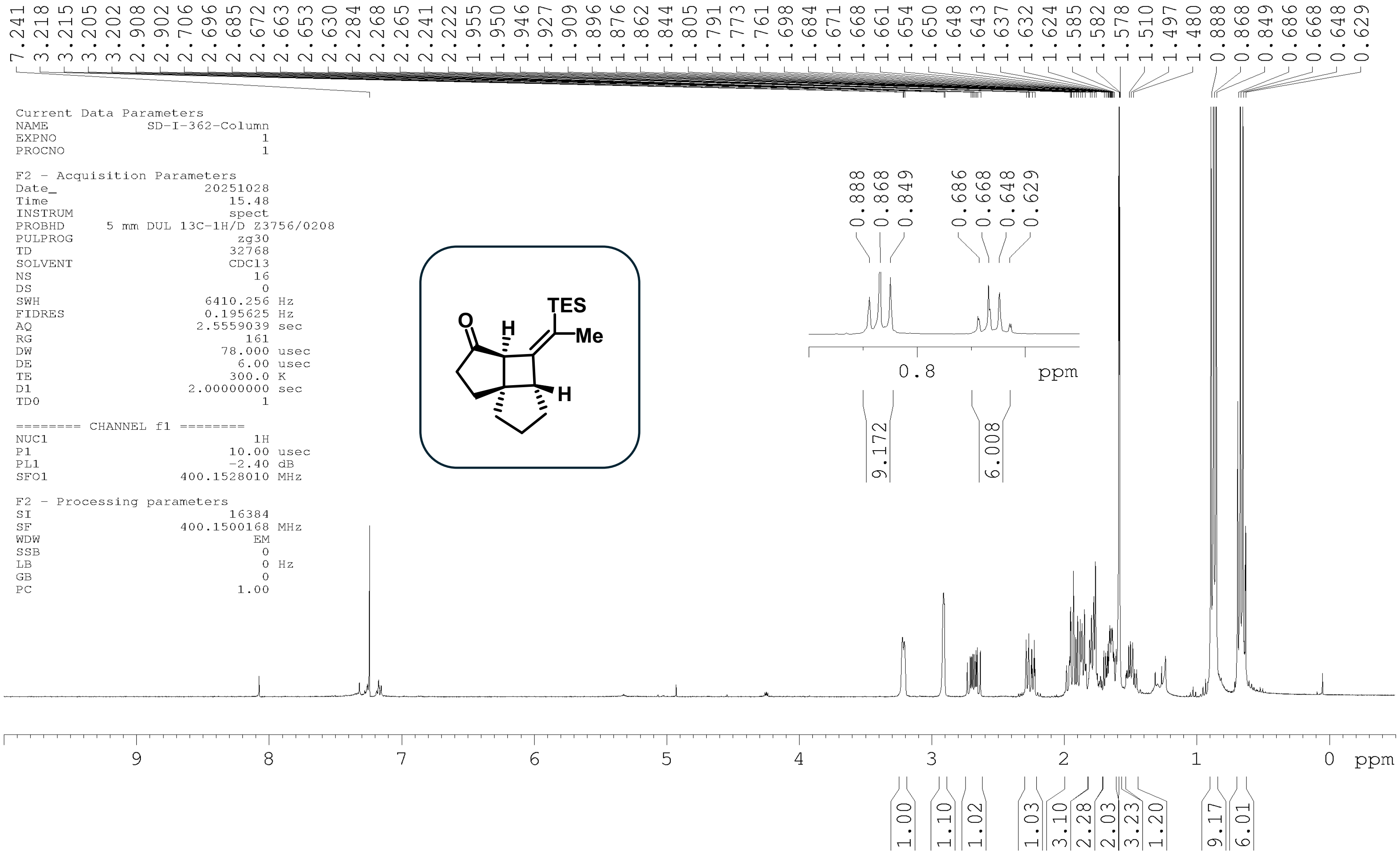

Figure 158. ^{1}H NMR (400 MHz, $CDCl_3$) (**30k**)

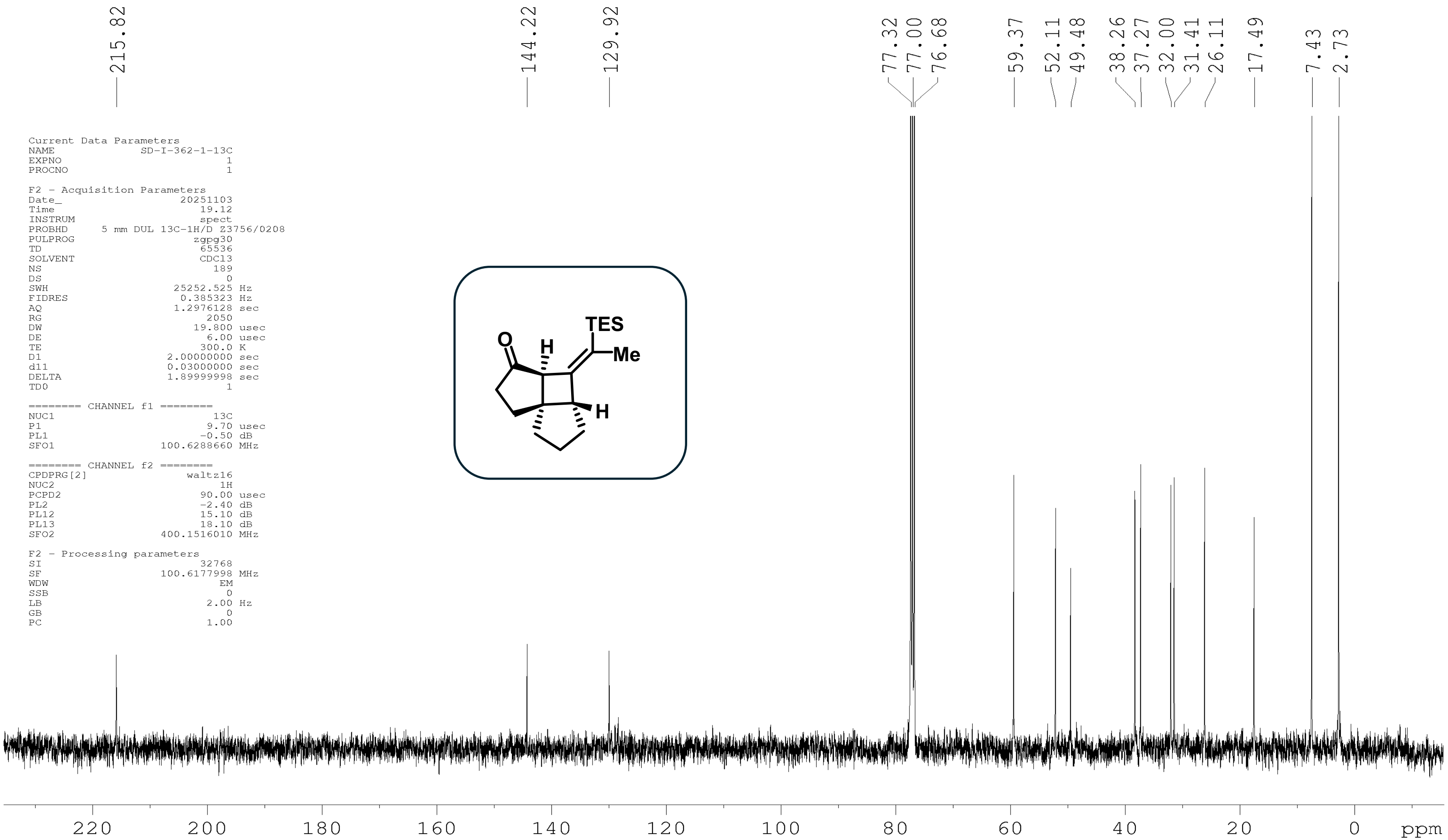

Figure 159. ^{13}C NMR (100 MHz, $CDCl_3$) (**30k**)

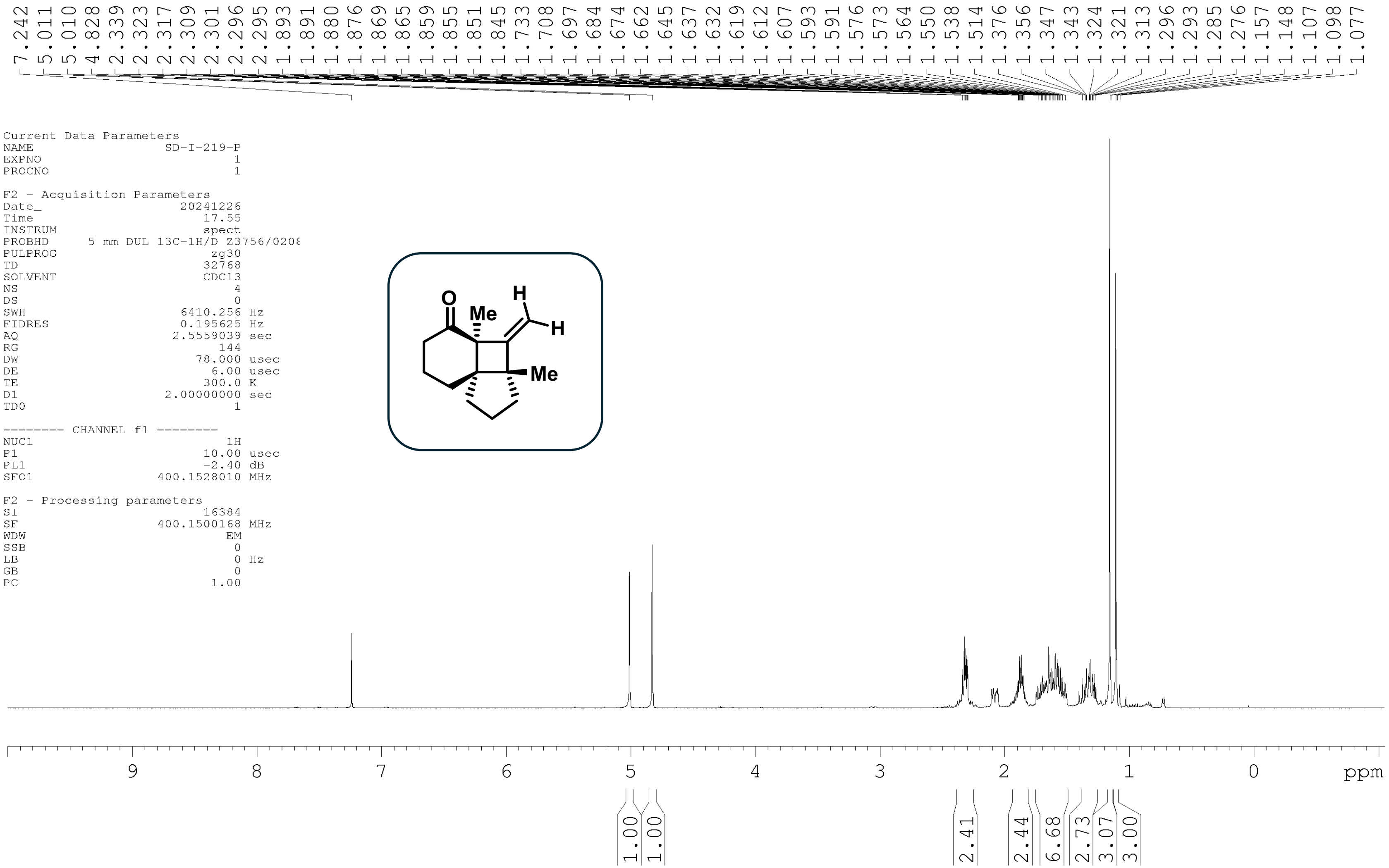

Figure 160. ^{1}H NMR (400 MHz, $CDCl_3$) (**30l**)

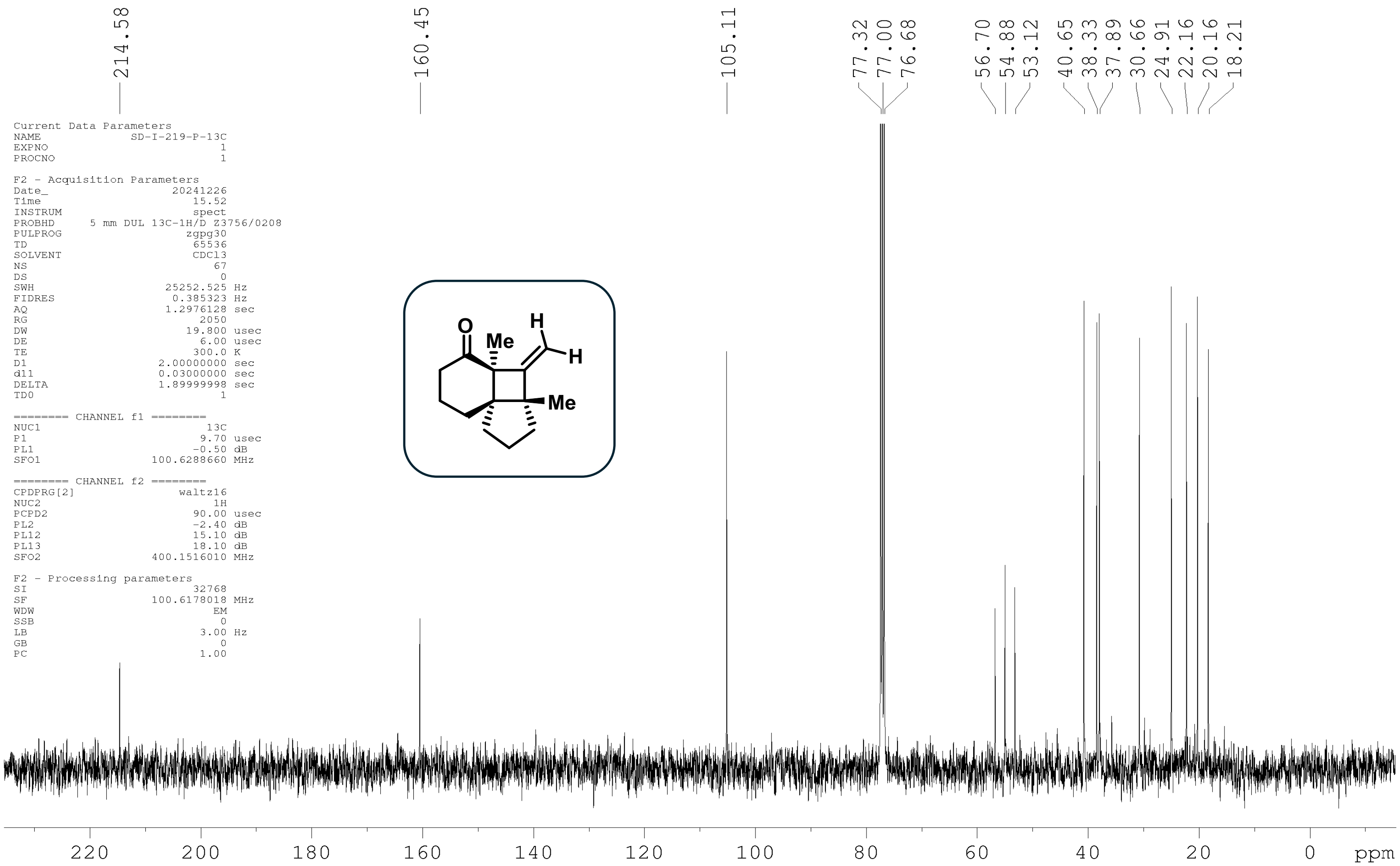

Figure 161. ^{13}C NMR (100 MHz, $CDCl_3$) (**30l**)

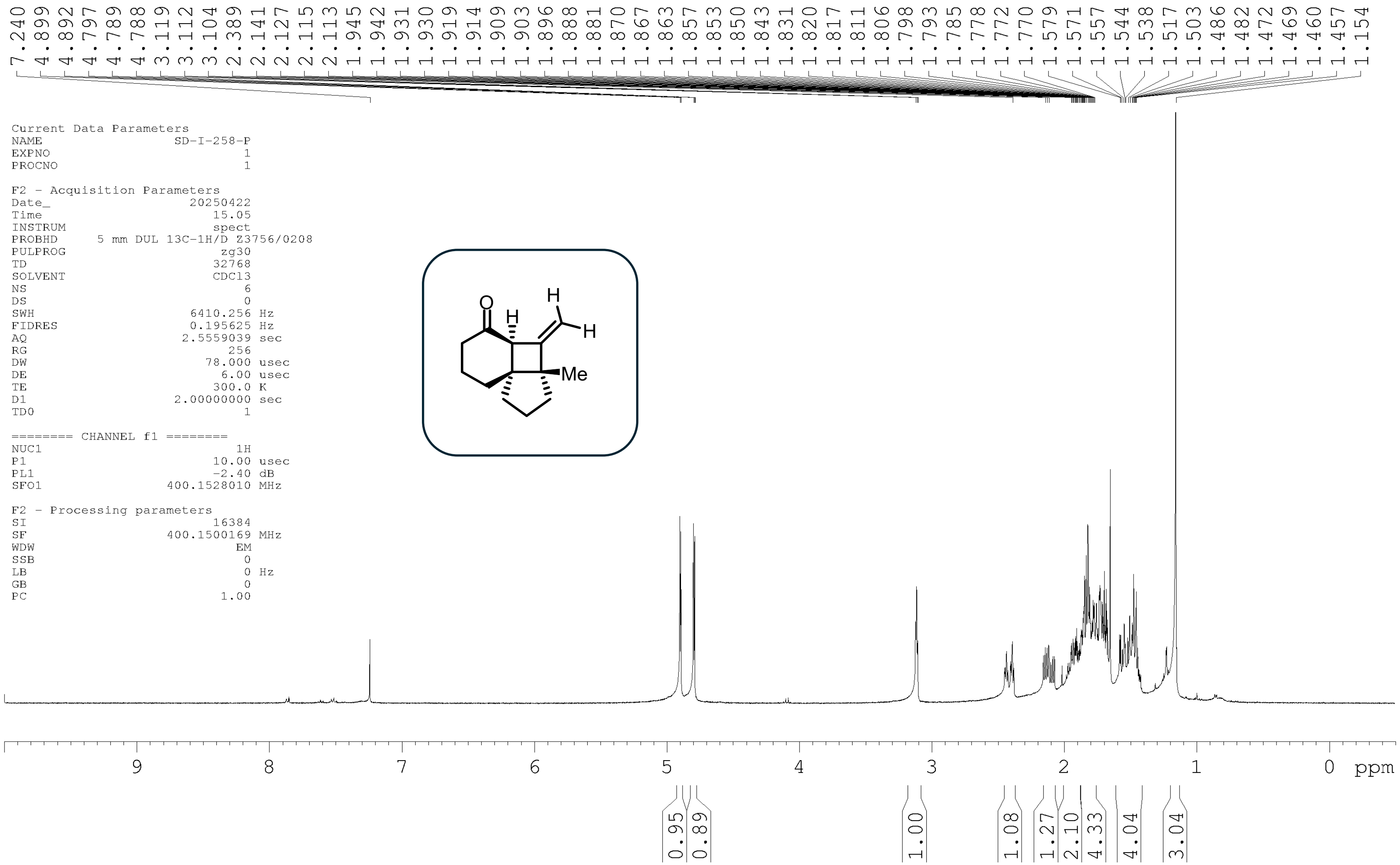

Figure 162 ^{1}H NMR (400 MHz, CDCl$_3$) (**30m**)

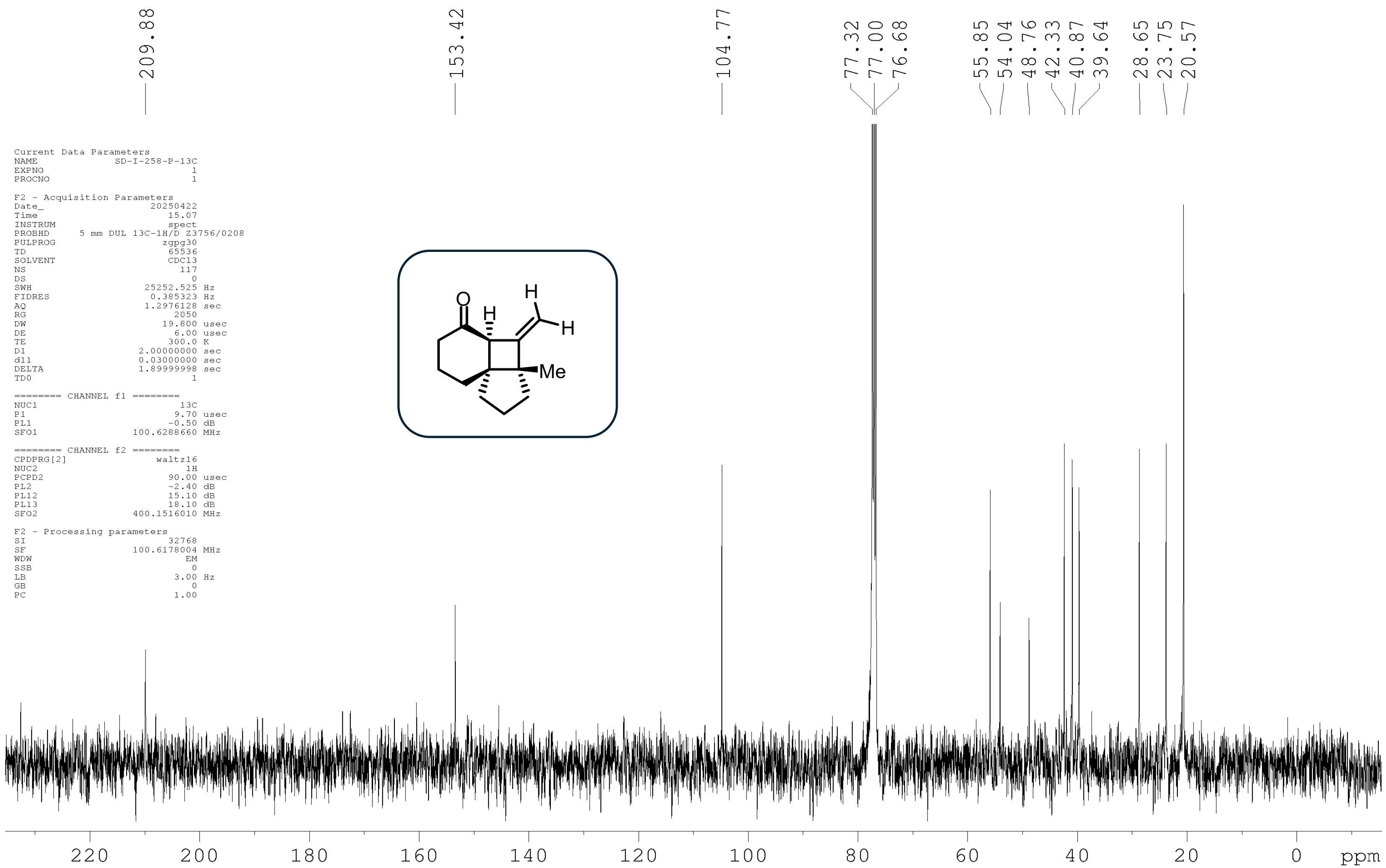

Figure 163. ^{13}C NMR (100 MHz, $CDCl_3$) (**30m**)

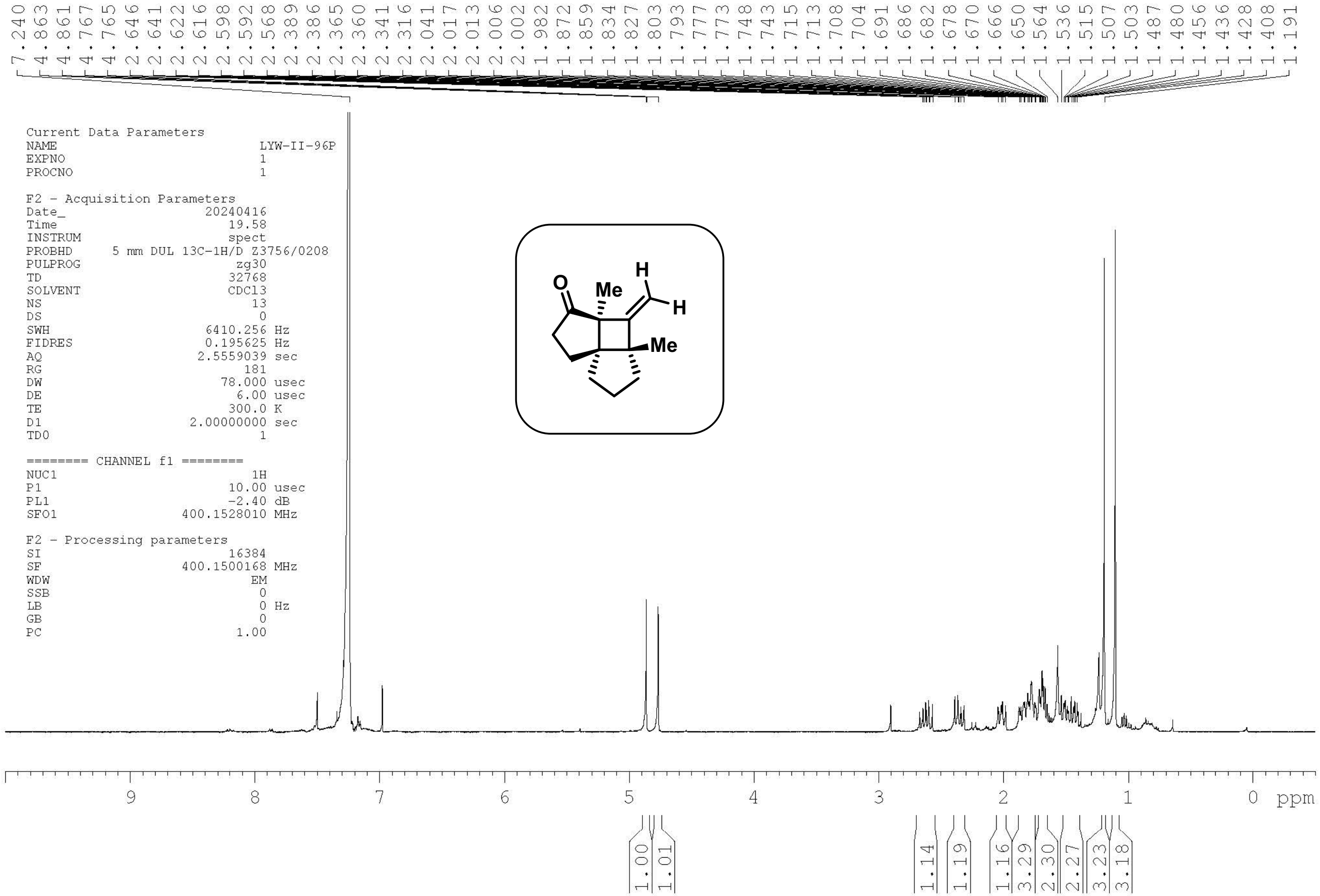

Figure 164. ^{1}H NMR (400 MHz, $CDCl_3$) (**30n**)

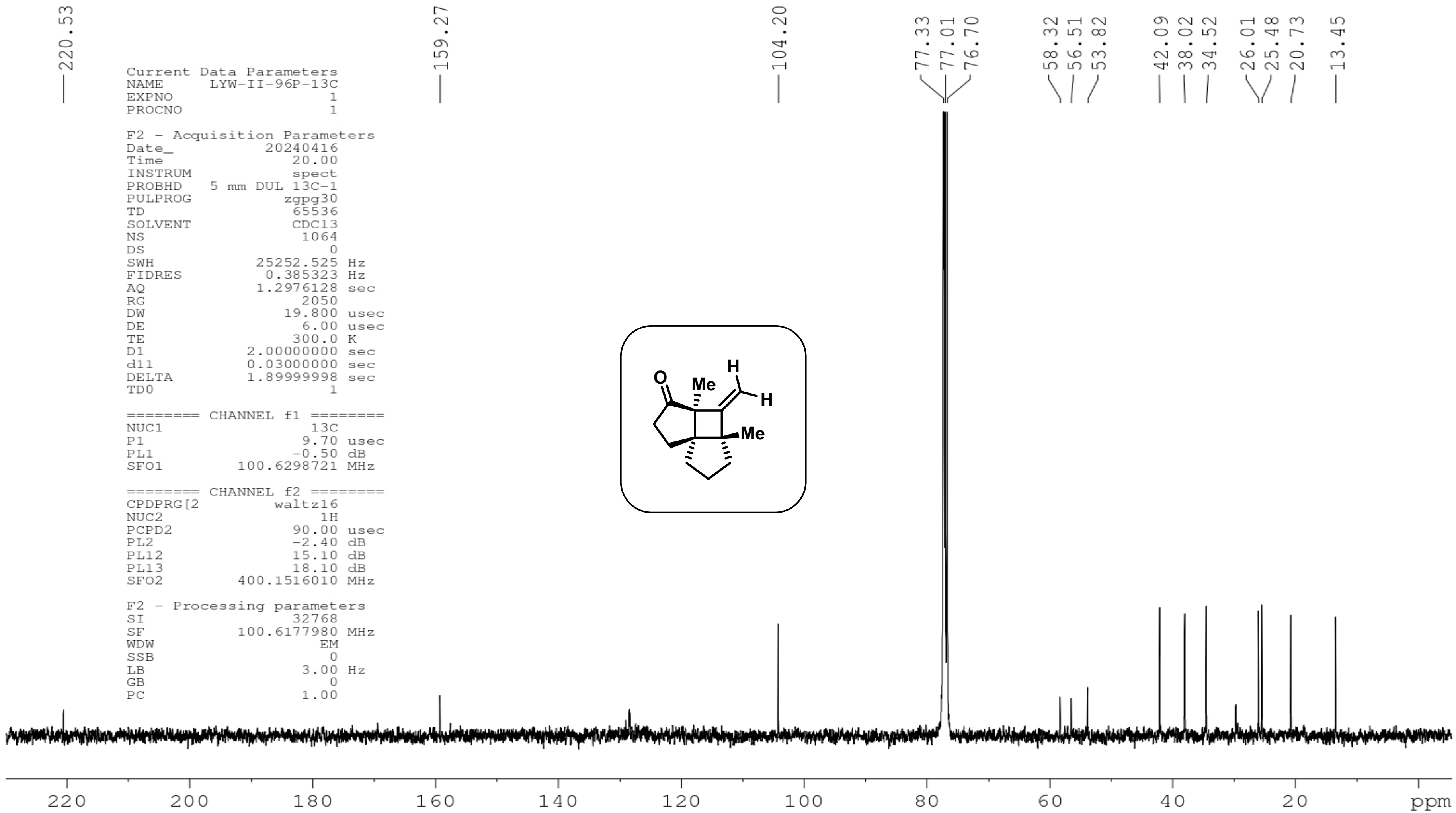

Figure 165. ^{13}C NMR (100 MHz, $CDCl_3$) (**30n**)

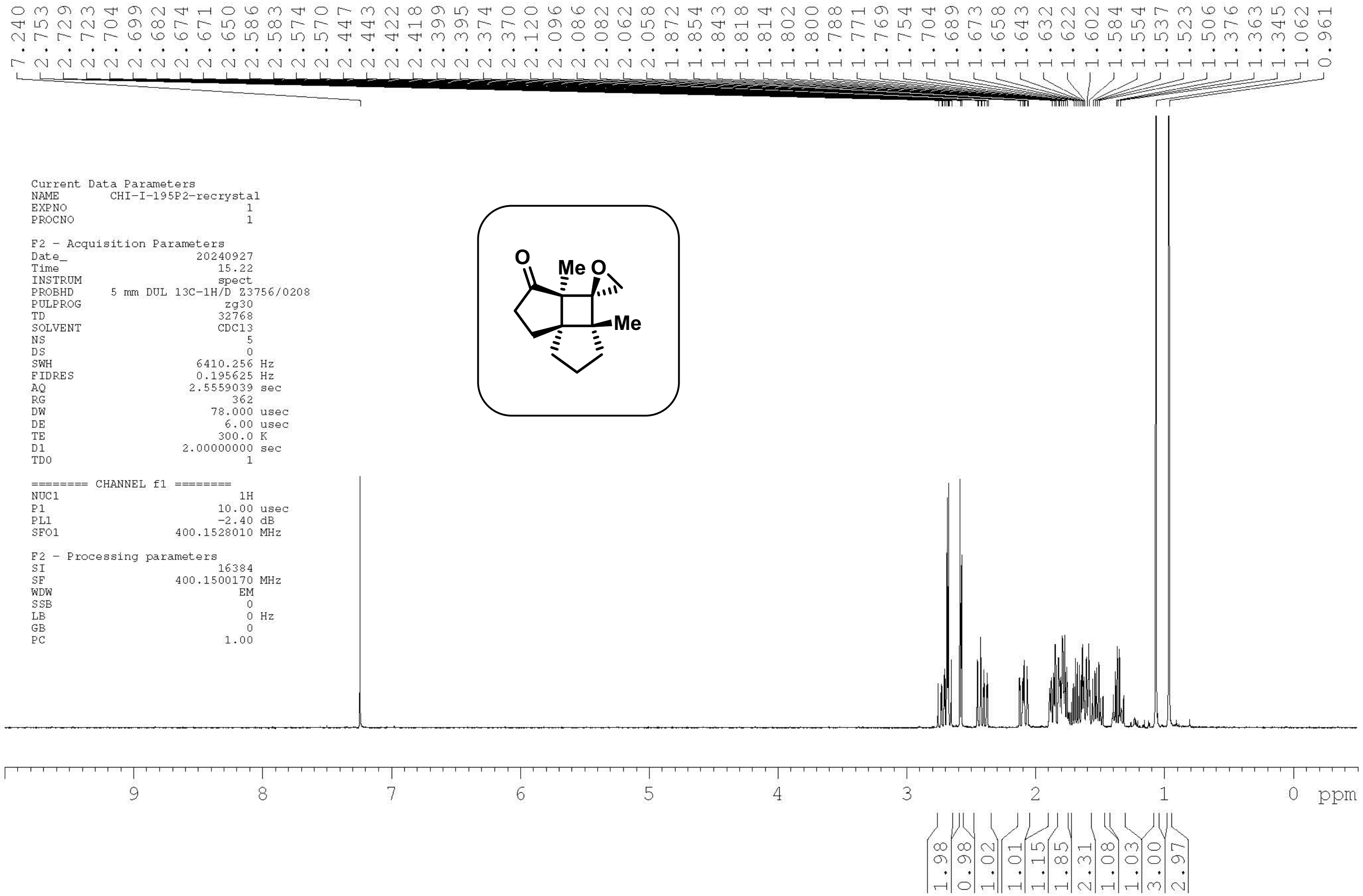

Figure 166. ^{1}H NMR (400 MHz, $CDCl_3$) (**31β**)

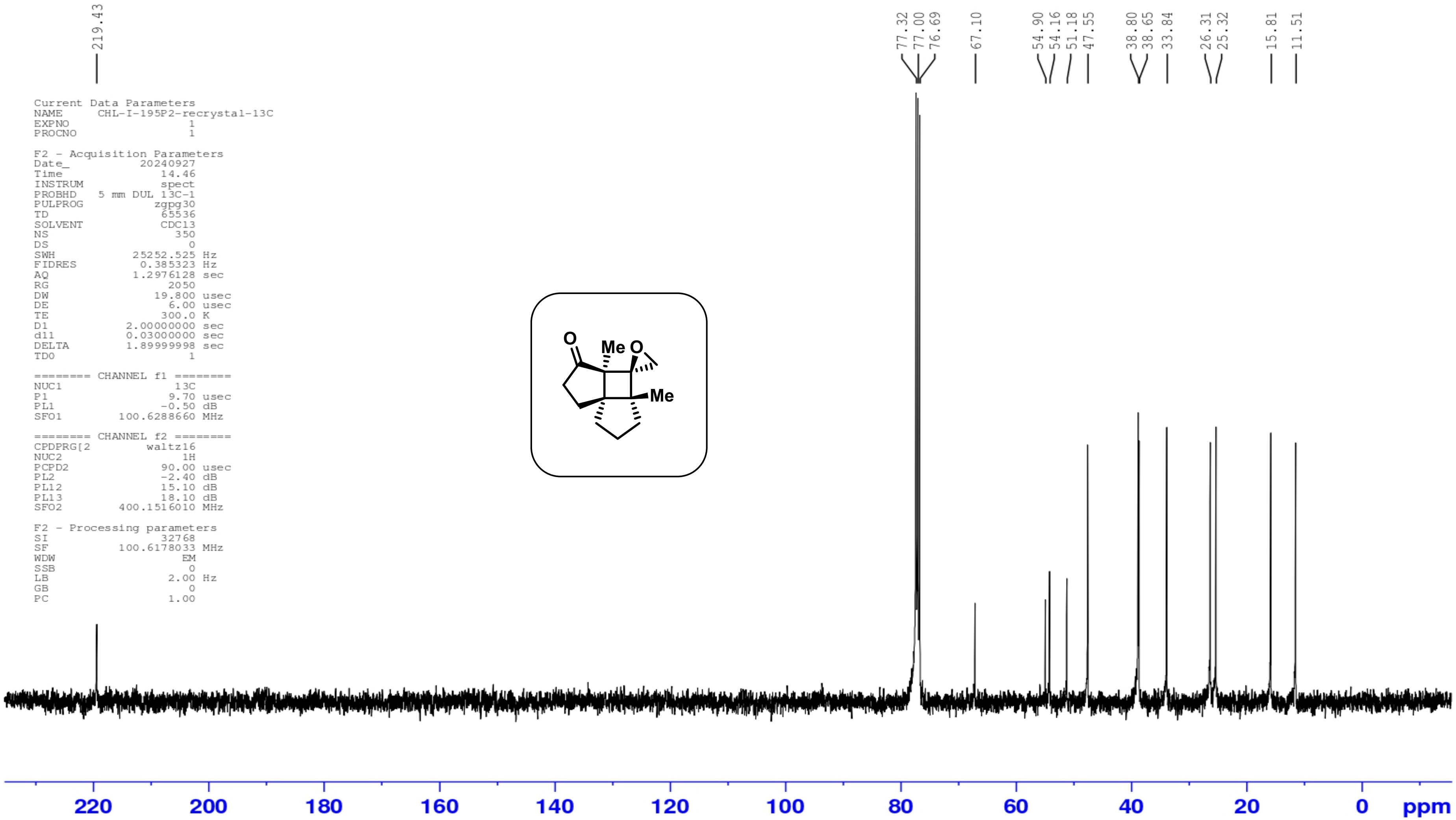

Figure 167. ^{13}C NMR (100 MHz, $CDCl_3$) (**31β**)

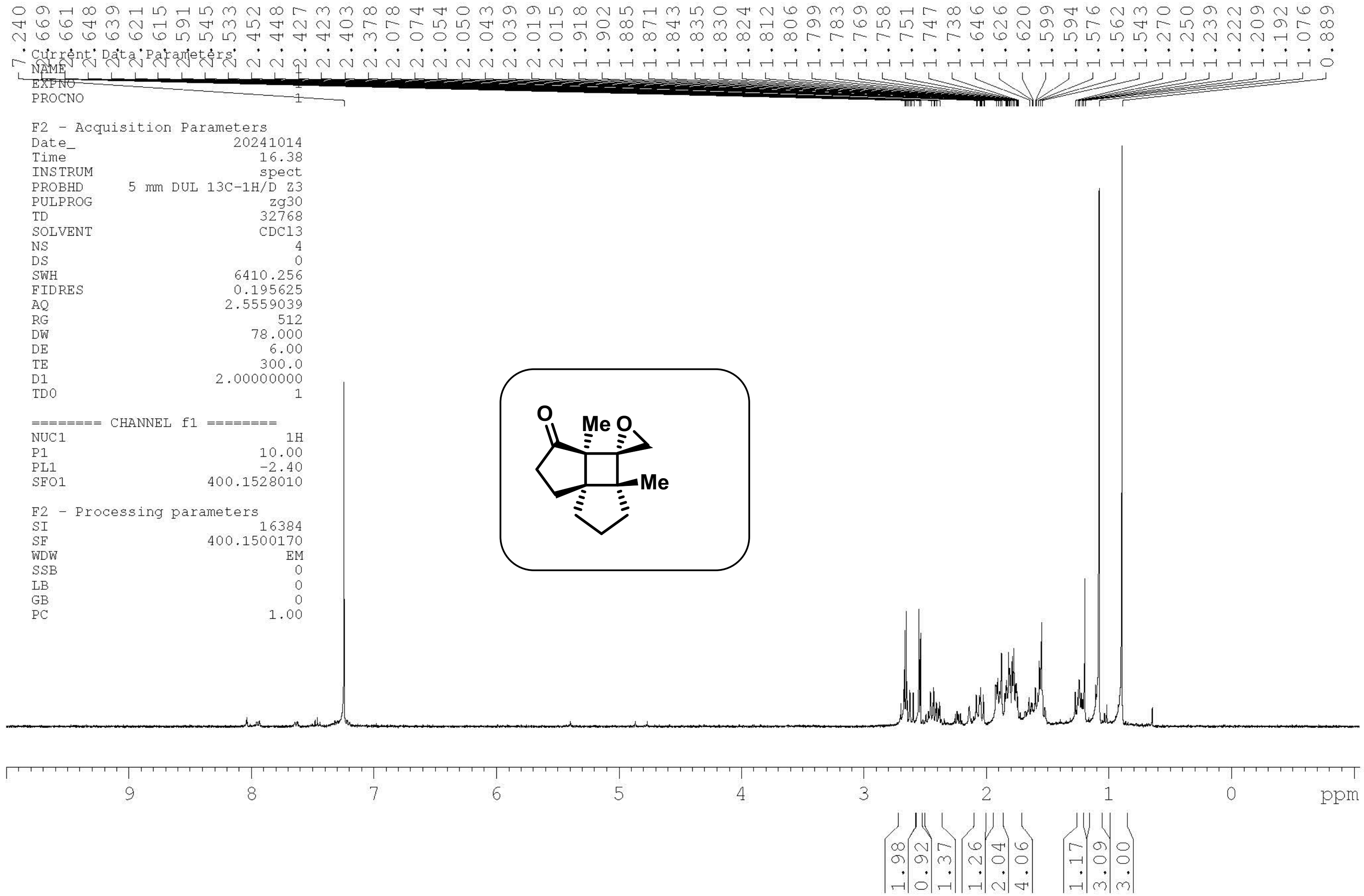

Figure 168. ^{1}H NMR (400 MHz, $CDCl_3$) (**31α**)

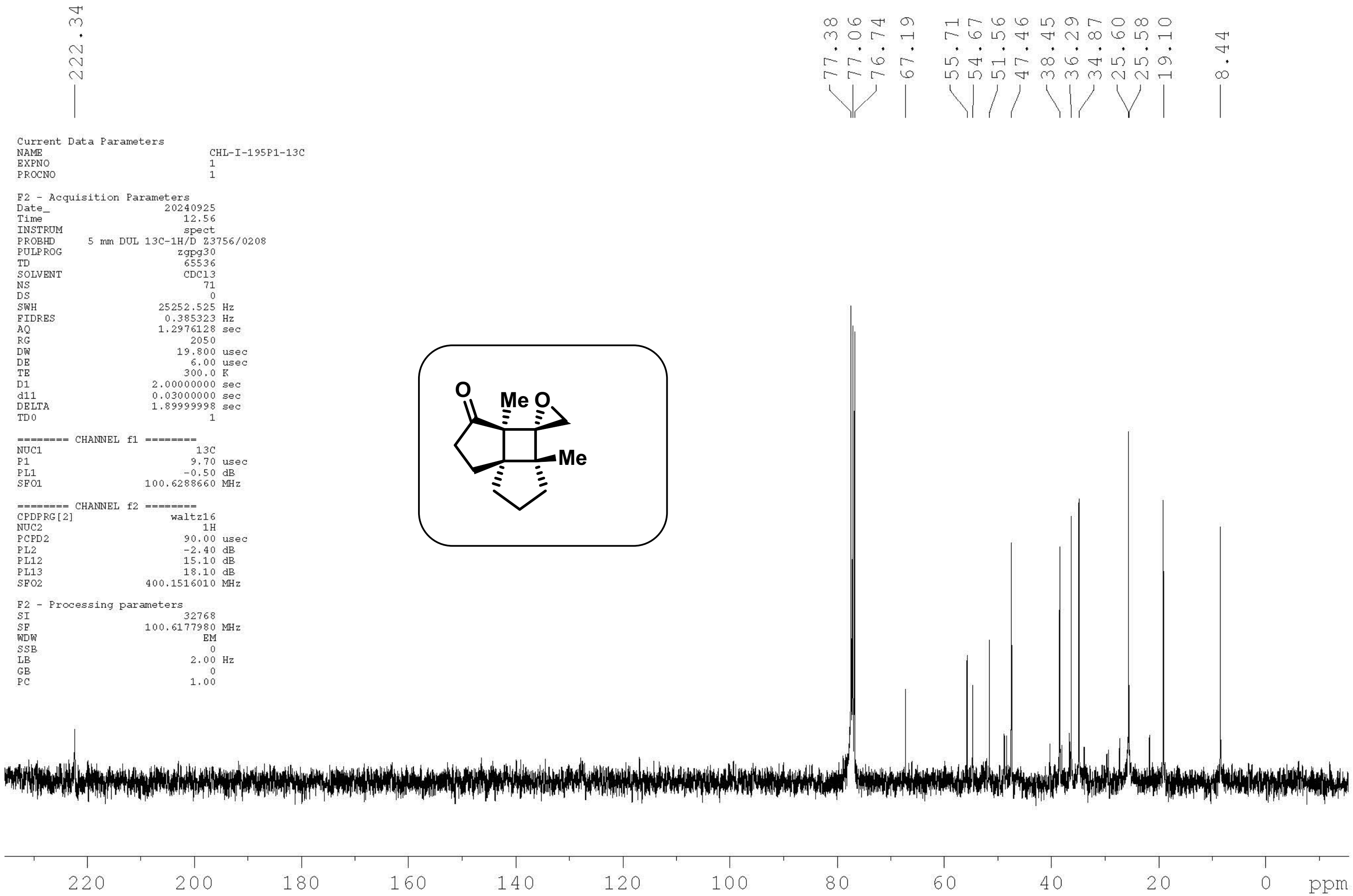

Figure 169. ^{13}C NMR (100 MHz, $CDCl_3$) (**31α**)

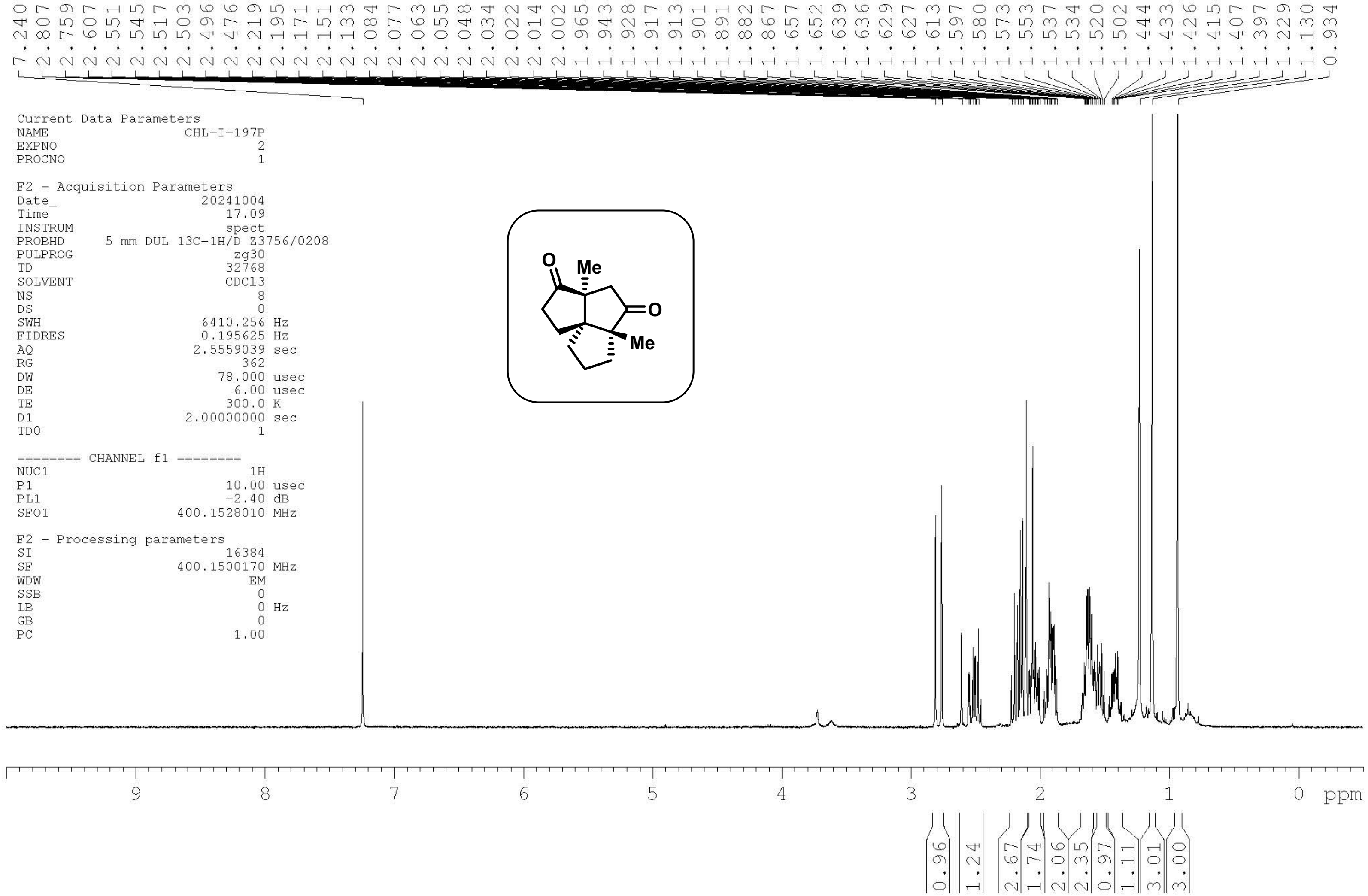

Figure 170. ^{1}H NMR (400 MHz, $CDCl_3$) (**33**)

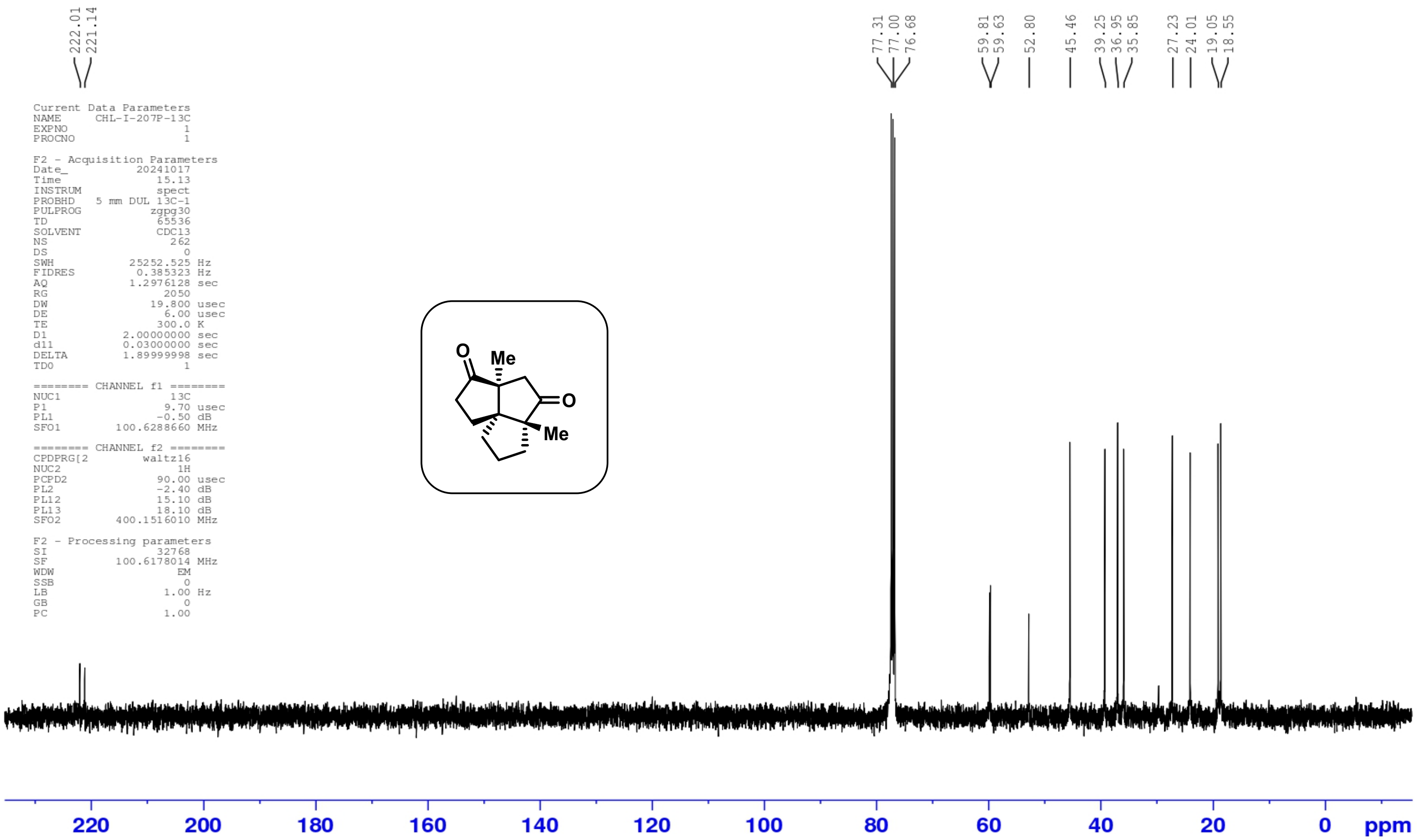

Figure 171. ^{13}C NMR (100 MHz, $CDCl_3$) (**33**)

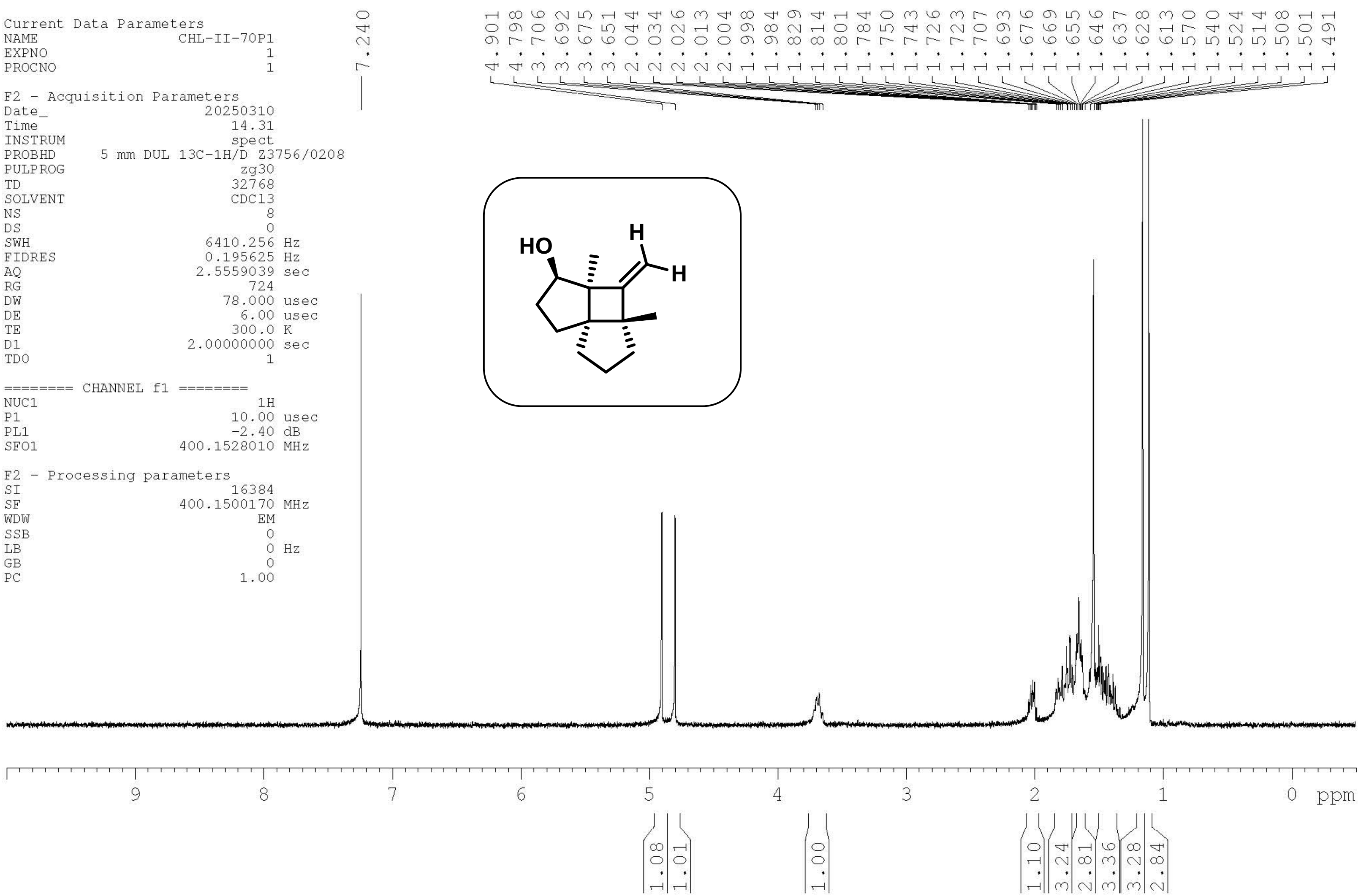

Figure 172. ^{1}H NMR (400 MHz, $CDCl_3$) (**37**)

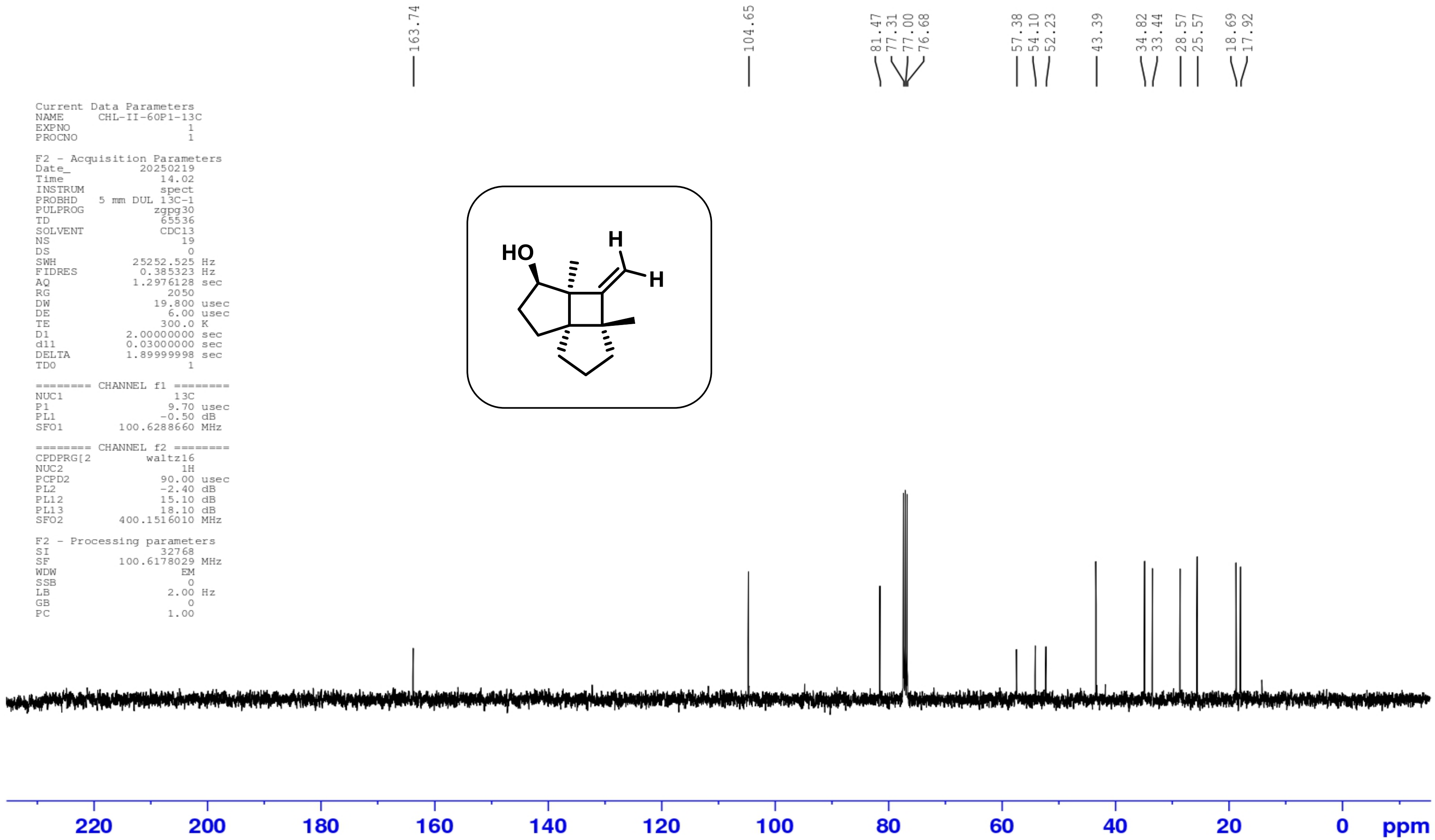

Figure 173. ^{13}C NMR (100 MHz, $CDCl_3$) (**37**)

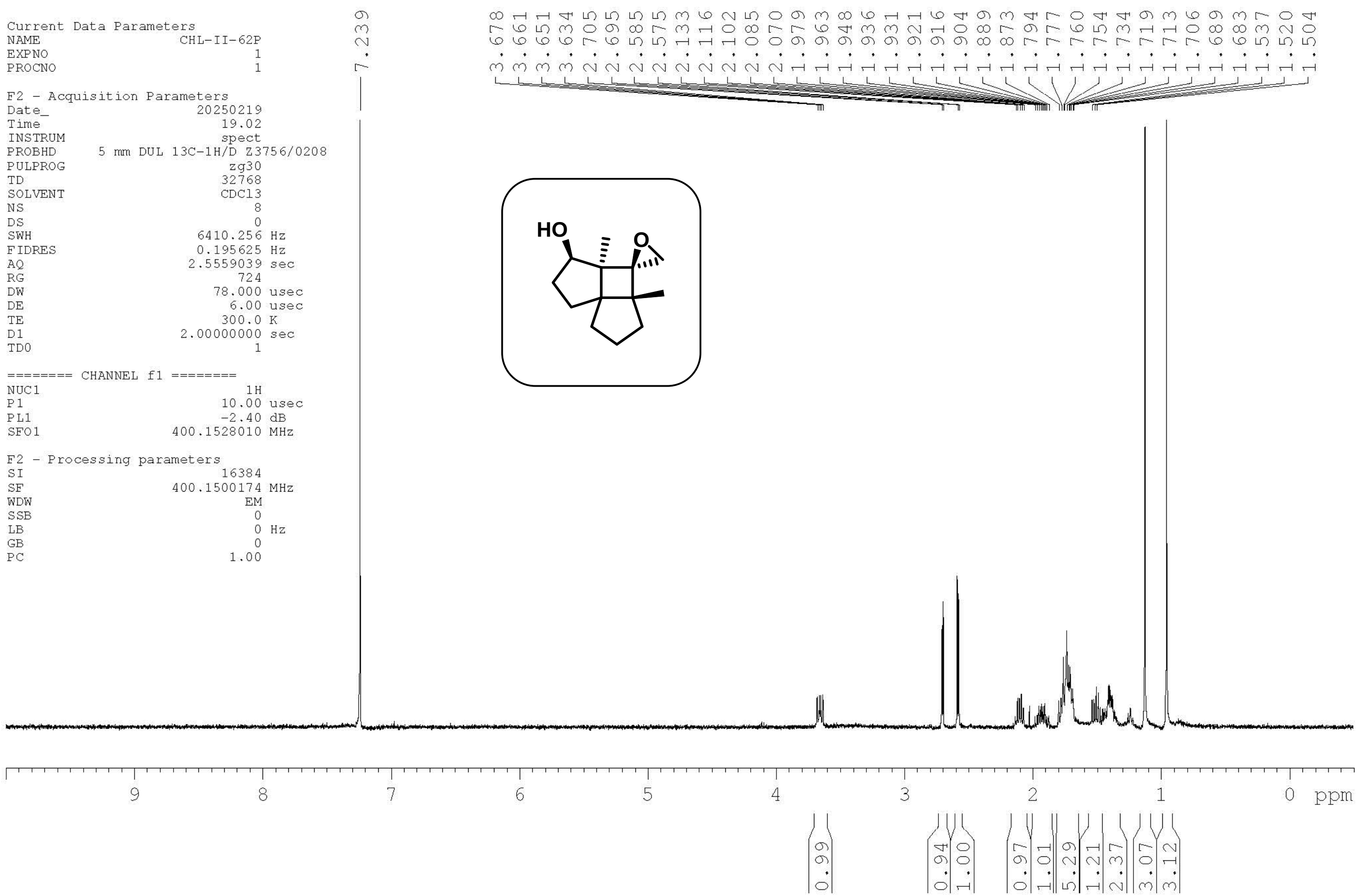

Figure 174. ^{1}H NMR (400 MHz, $CDCl_3$) (**38**)

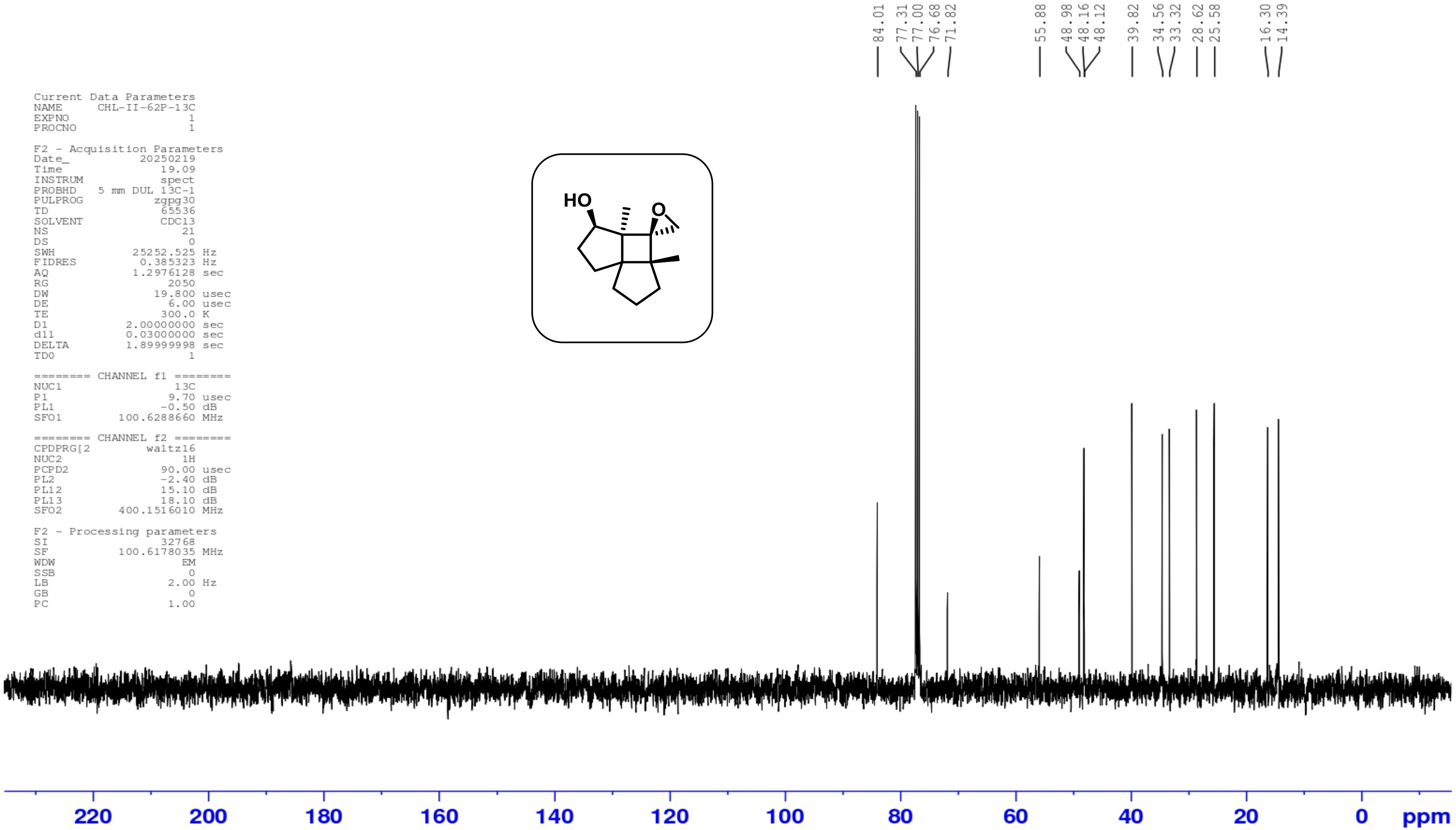

Figure 175. ^{13}C NMR (100 MHz, $CDCl_3$) (**38**)

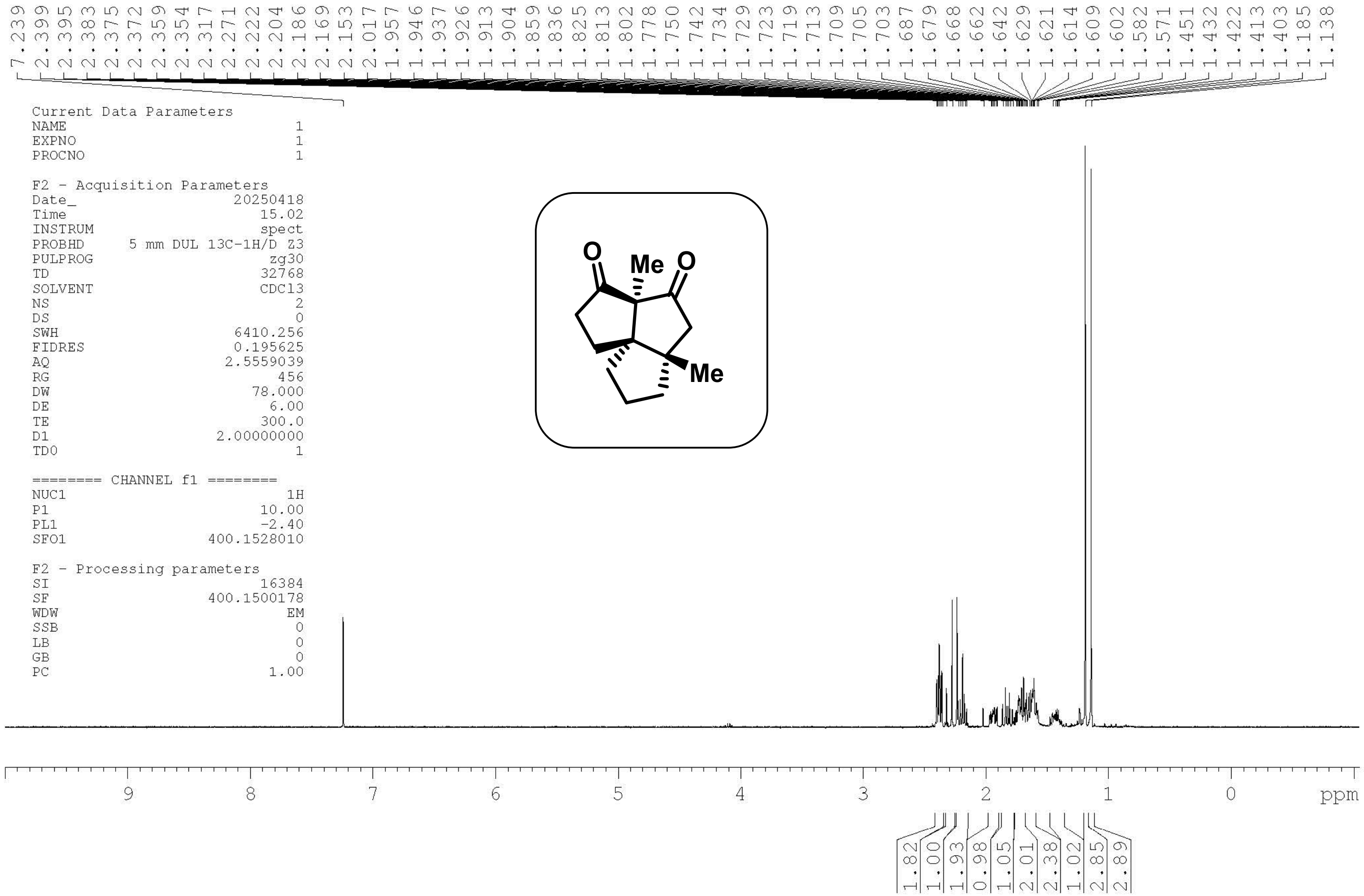

Figure 176. ^{1}H NMR (400 MHz, $CDCl_3$) (**41**)

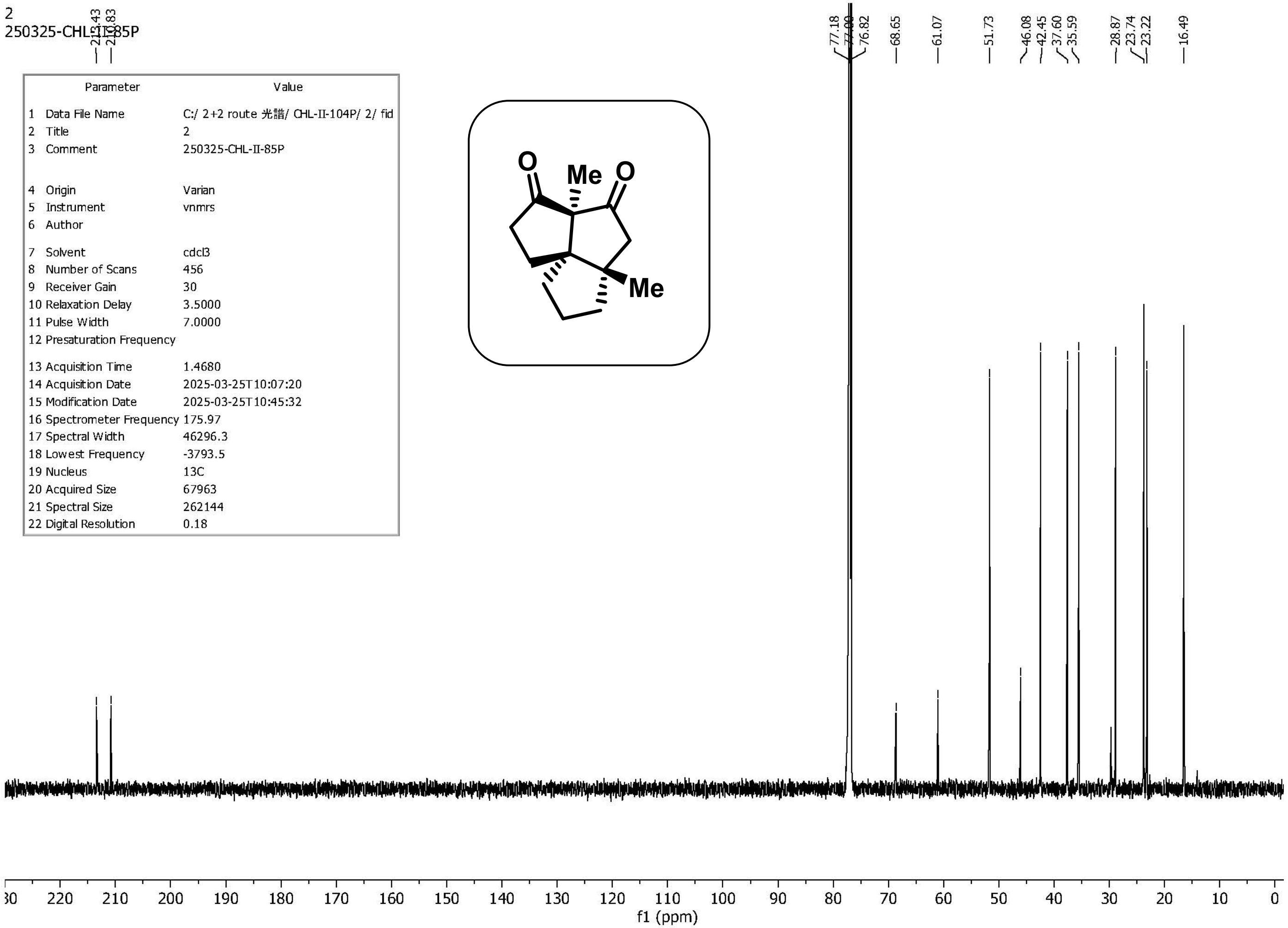

Figure 177. ^{13}C NMR (100 MHz, $CDCl_3$) (**41**)

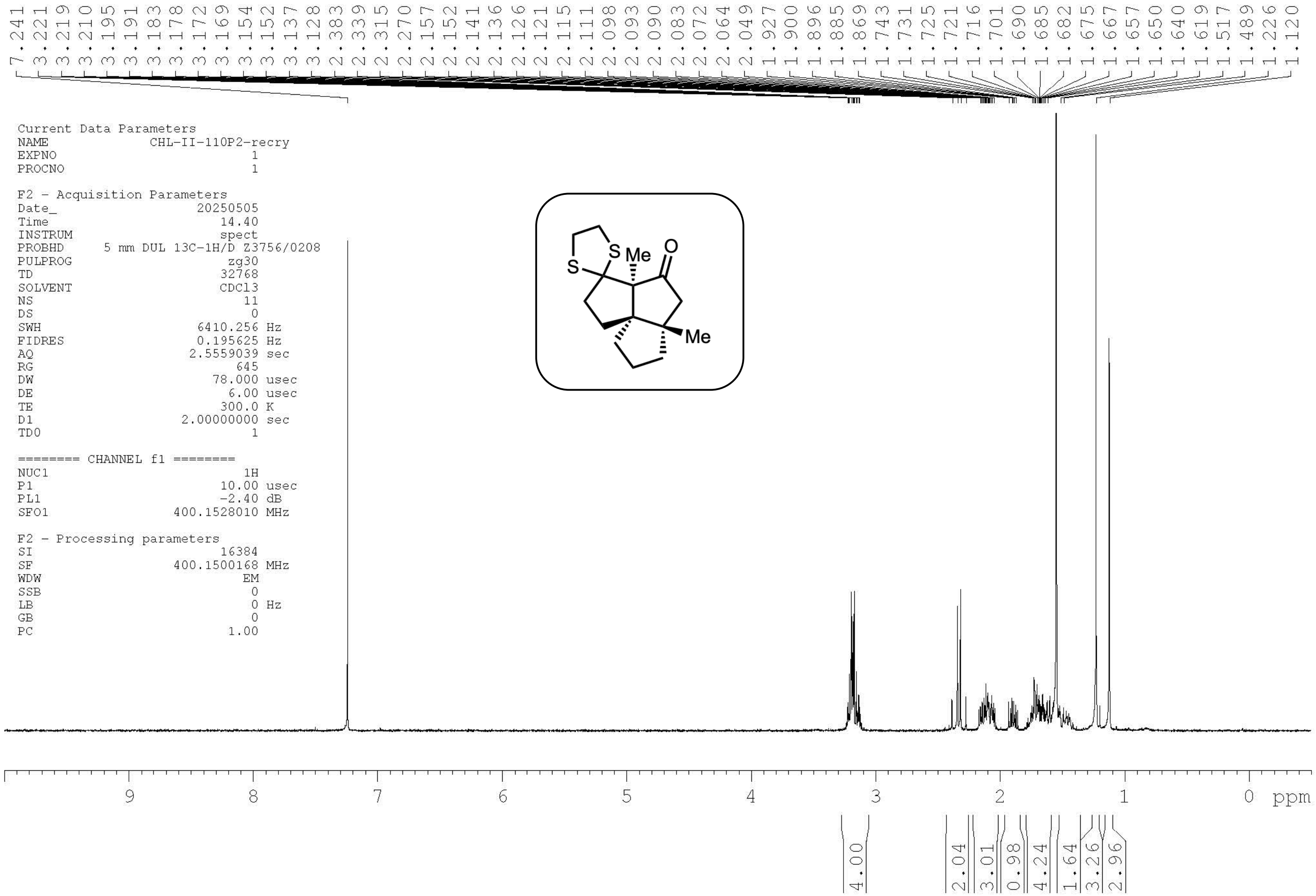

Figure 178. ^{1}H NMR (400 MHz, $CDCl_3$) (**42**)

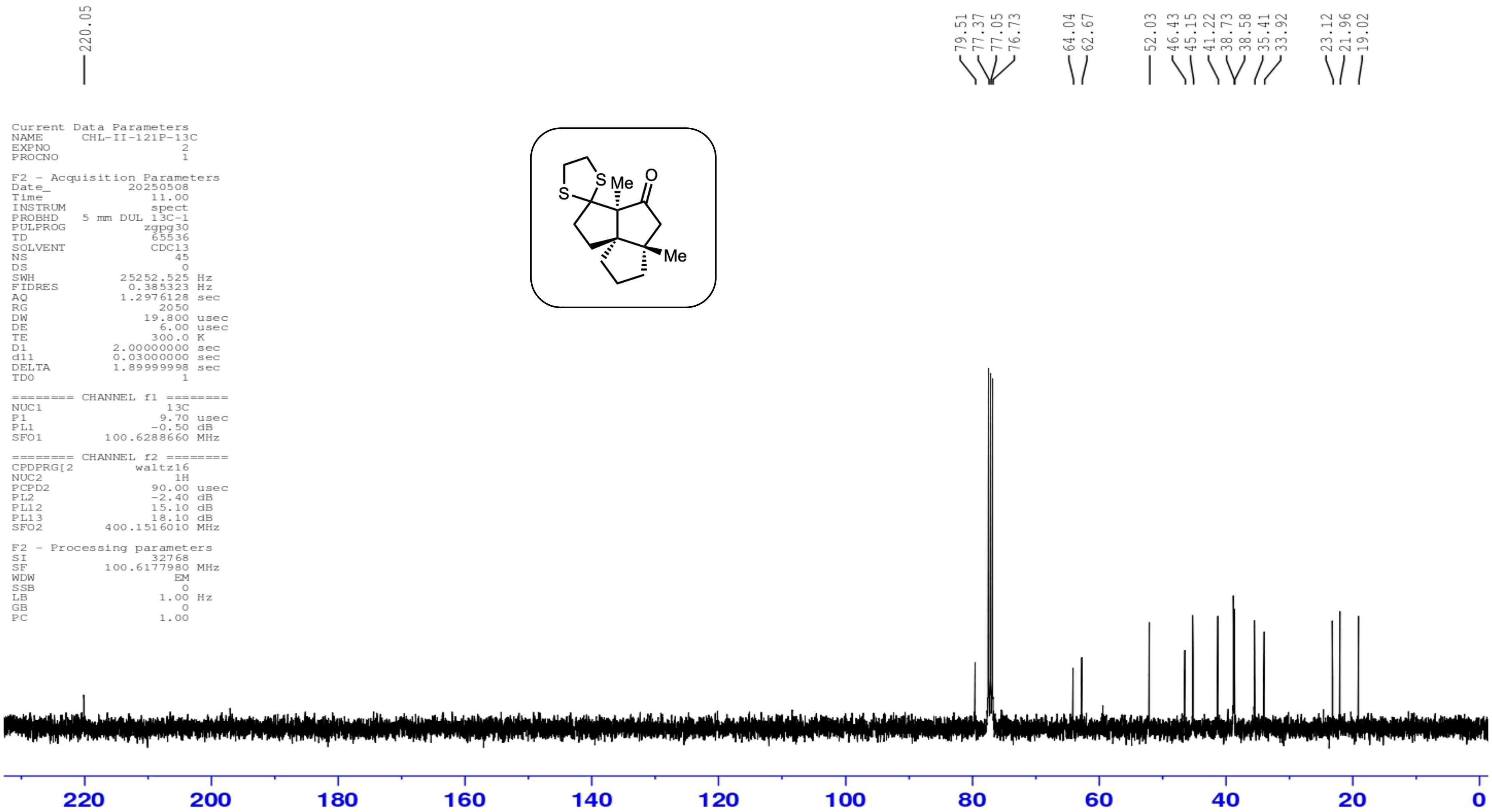

Figure 179. ^{13}C NMR (100 MHz, $CDCl_3$) (**42**)

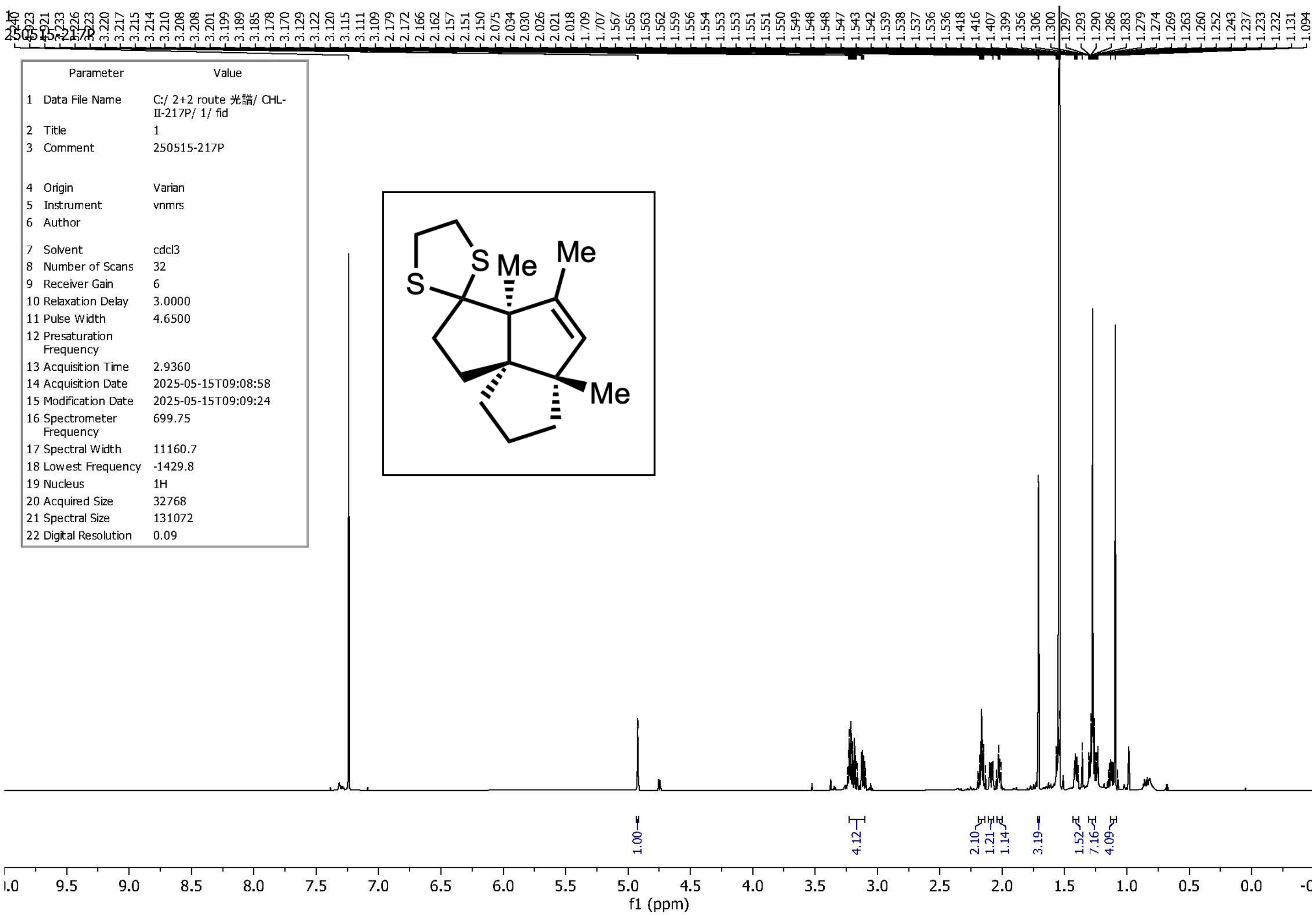

Figure 180. ^{1}H NMR (700 MHz, $CDCl_3$) (**44**)

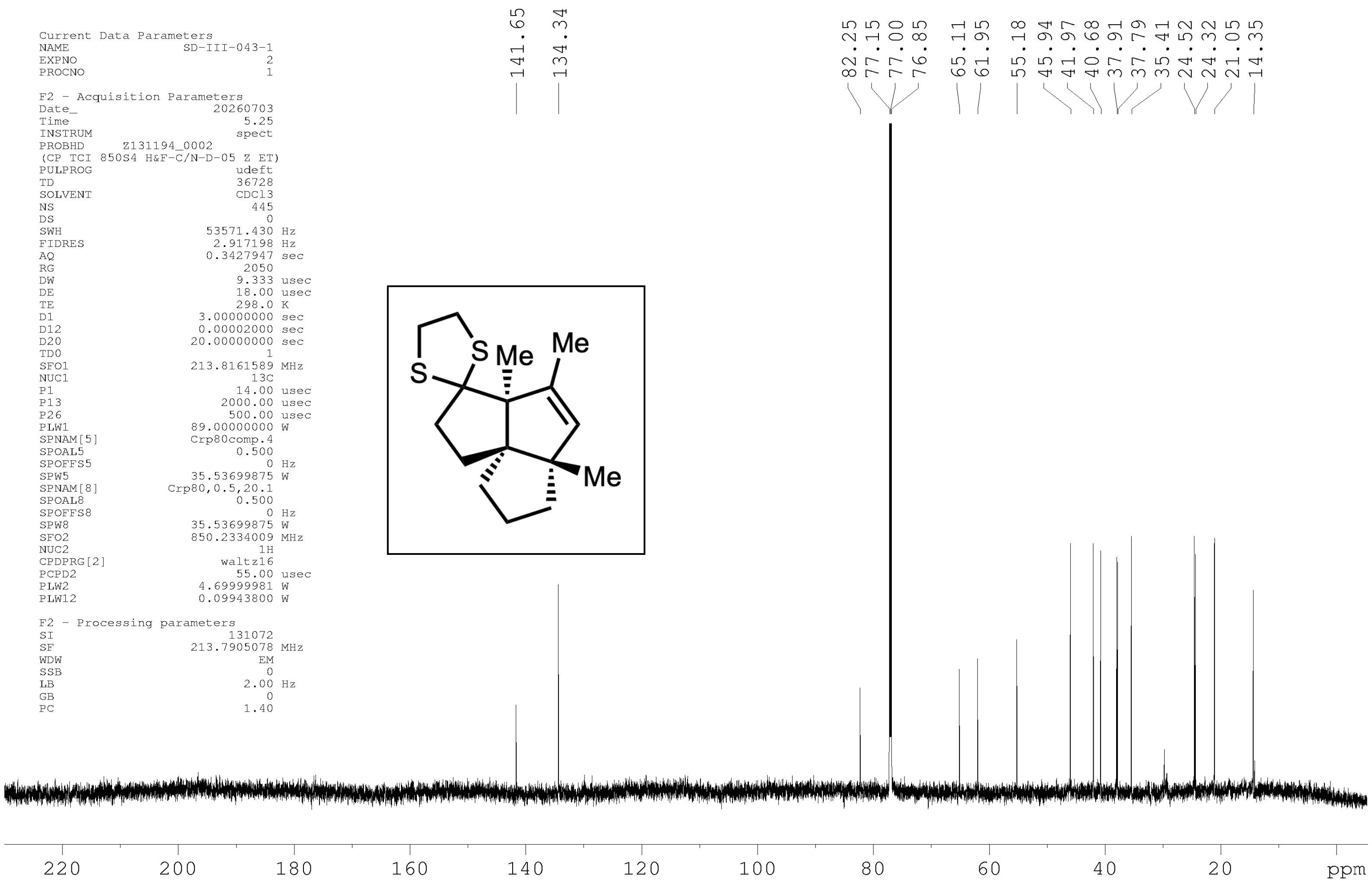

Figure 181. ^{13}C NMR (212.5 MHz, $CDCl_3$) (**44**)

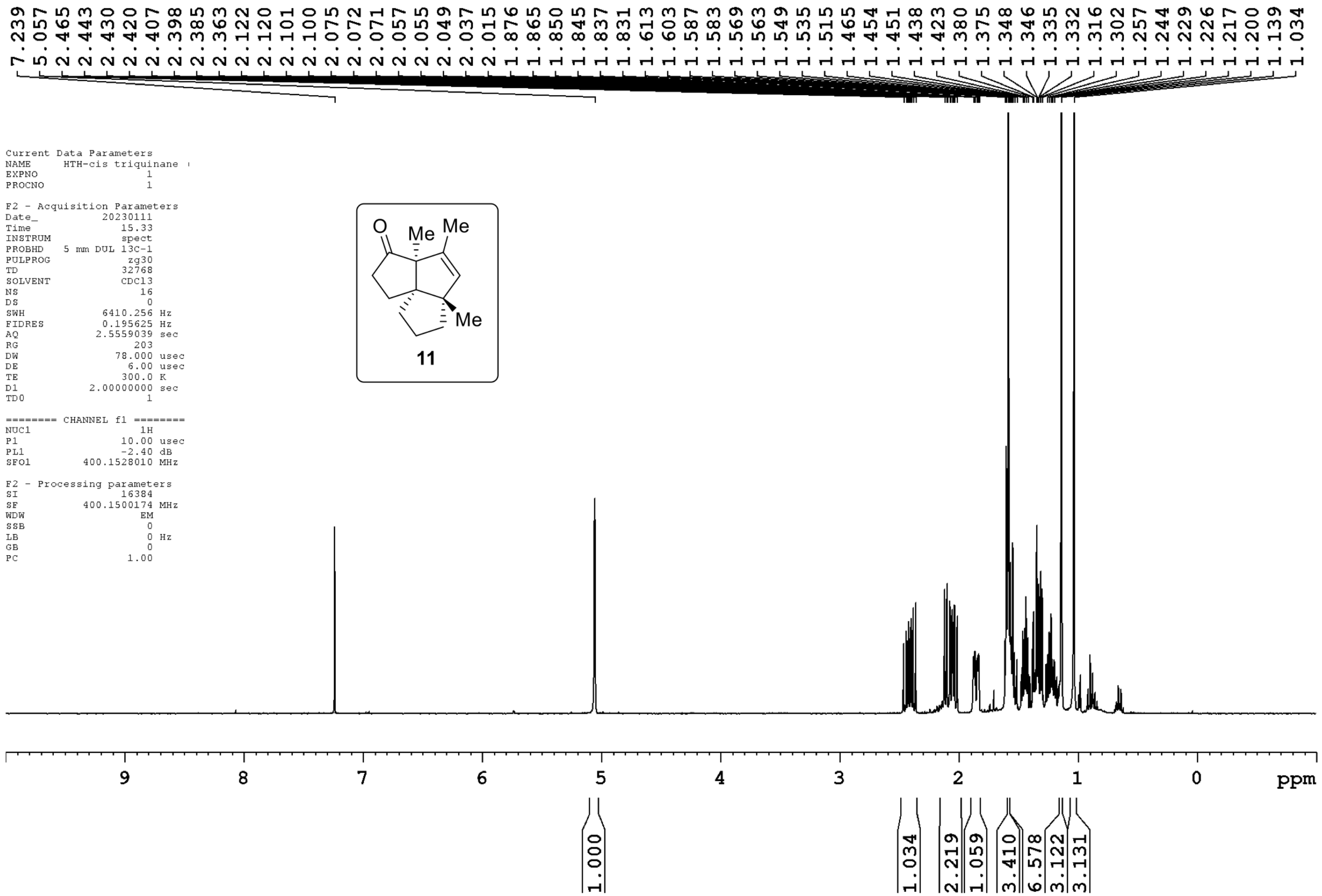

Figure 182. ^{1}H NMR (400 MHz, $CDCl_3$) (**35**)

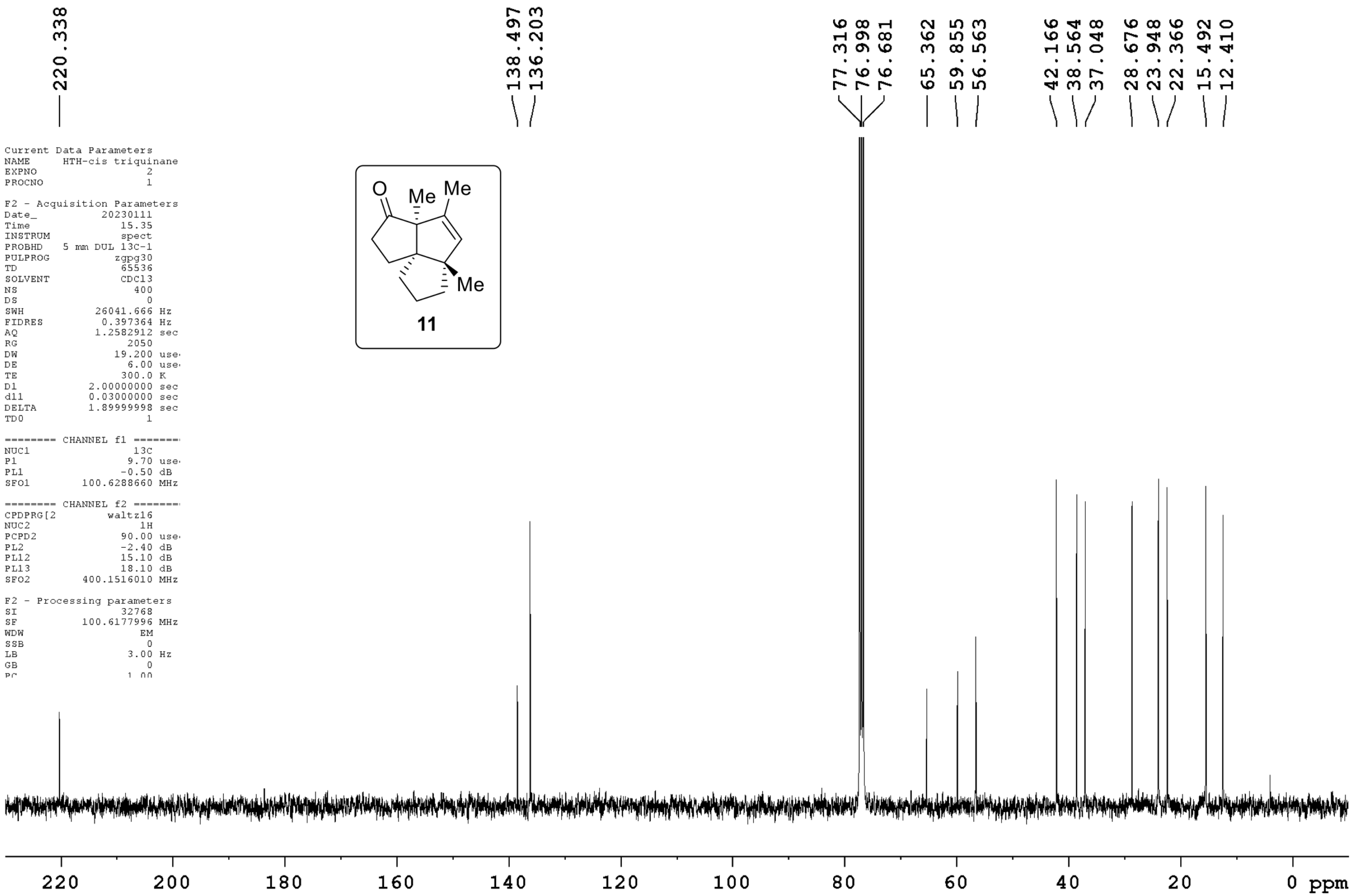

Figure 183. ^{13}C NMR (175 MHz, $CDCl_3$) (**35**)

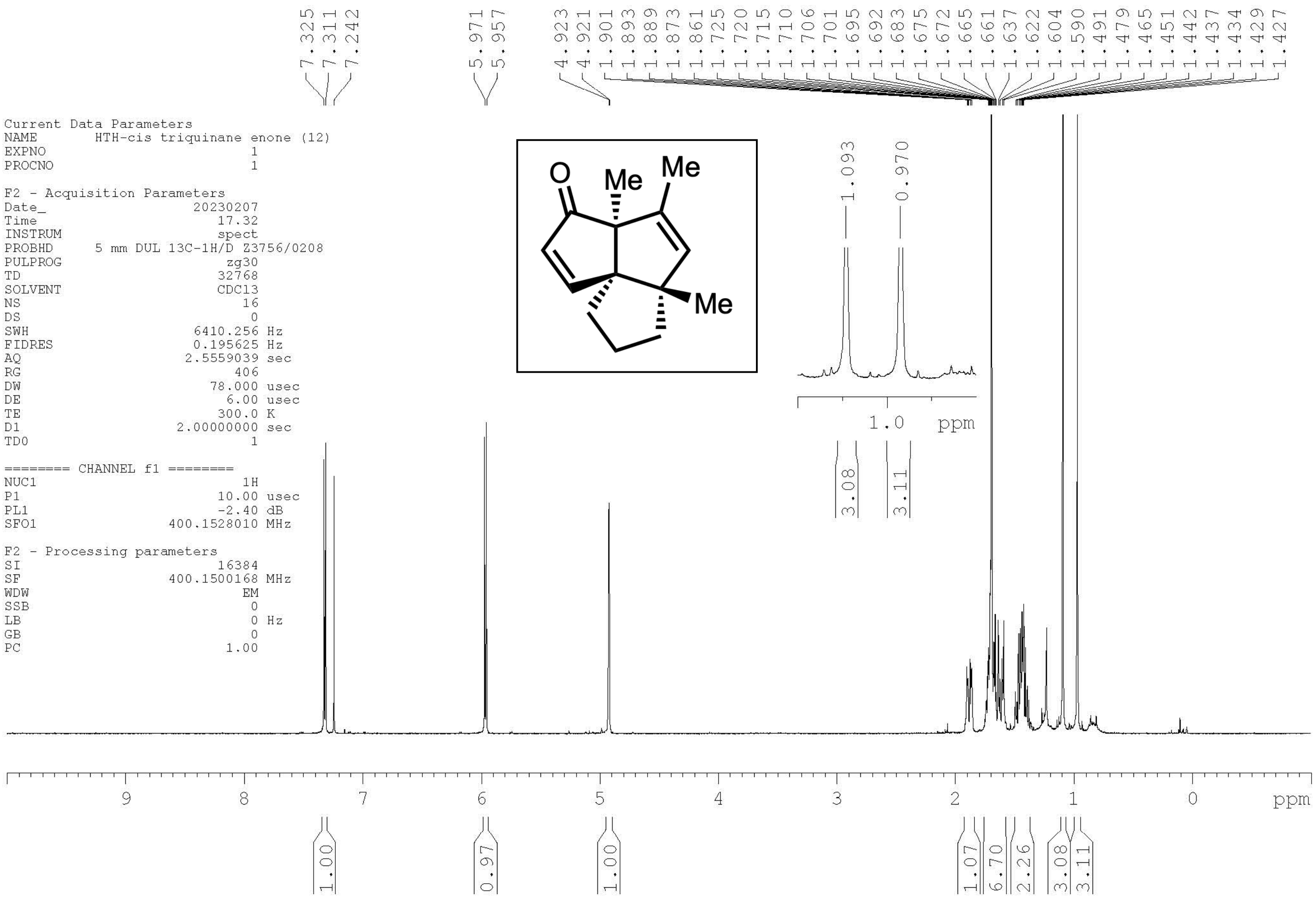

Figure 184. ^{1}H NMR (400 MHz, $CDCl_3$) (**36**)

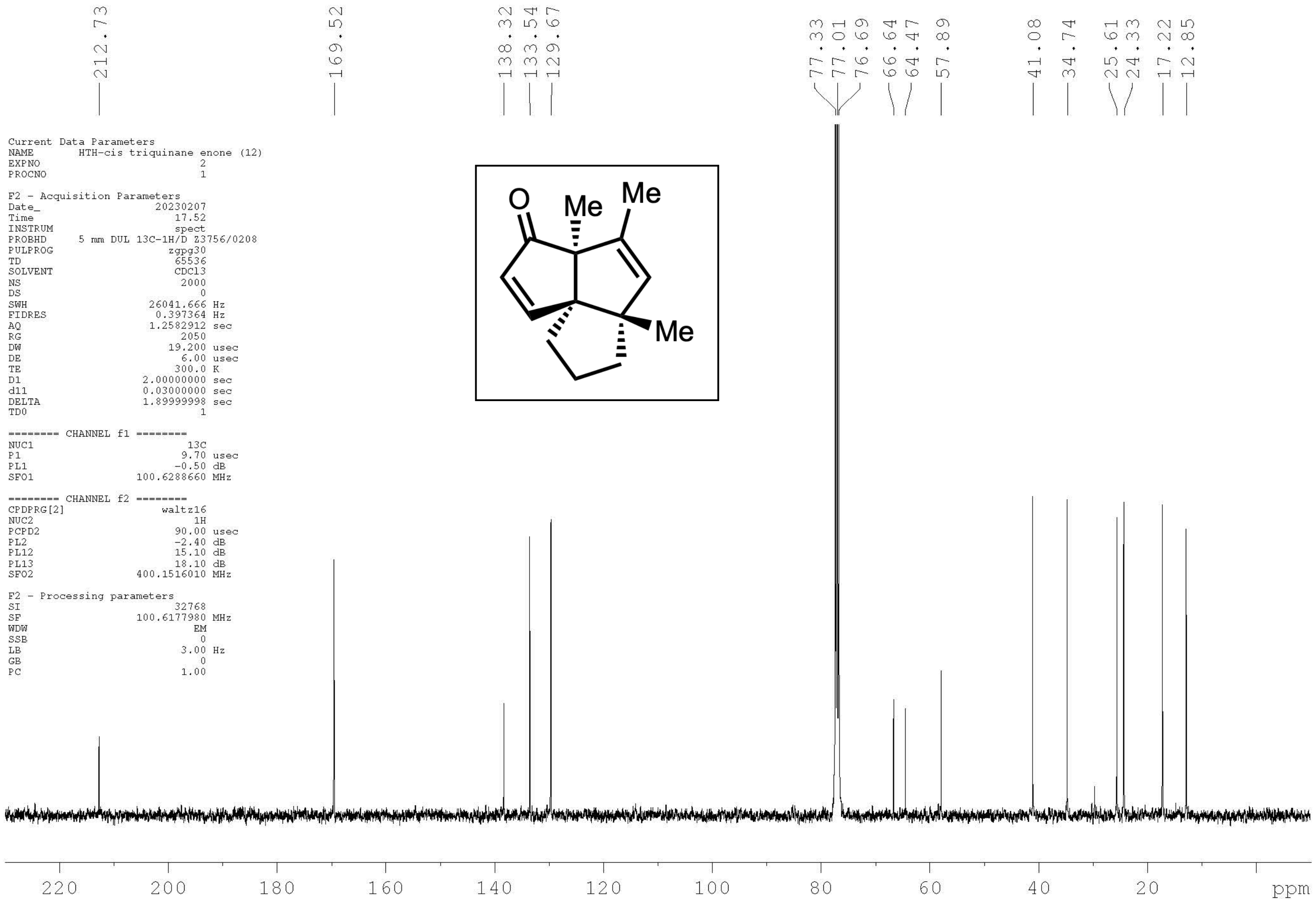

Figure 185. ^{13}C NMR (100 MHz, $CDCl_3$) (**36**)

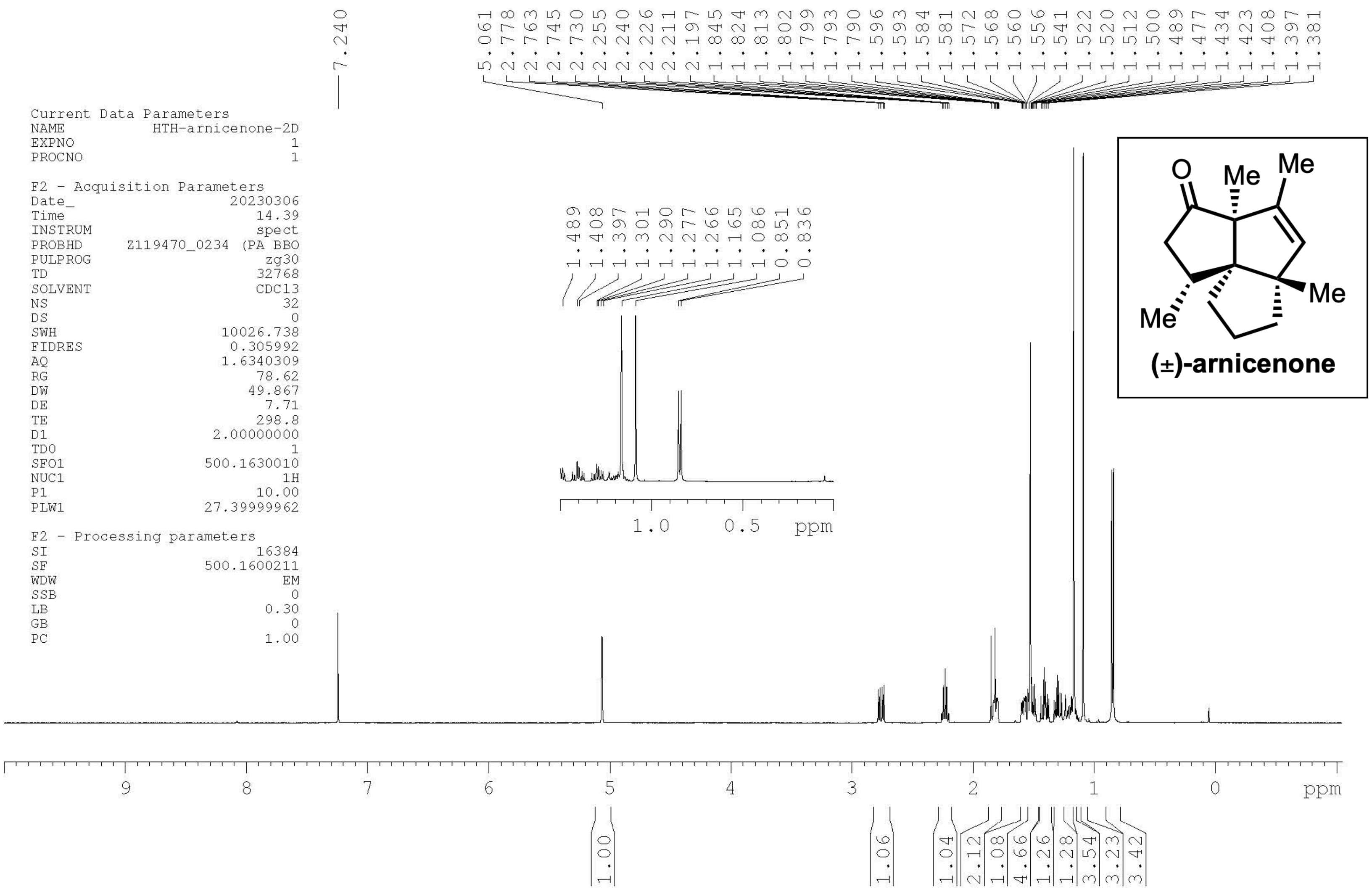

Figure 186. ^{1}H NMR (500 MHz, $CDCl_3$) (**3**)(**arnicenone**)

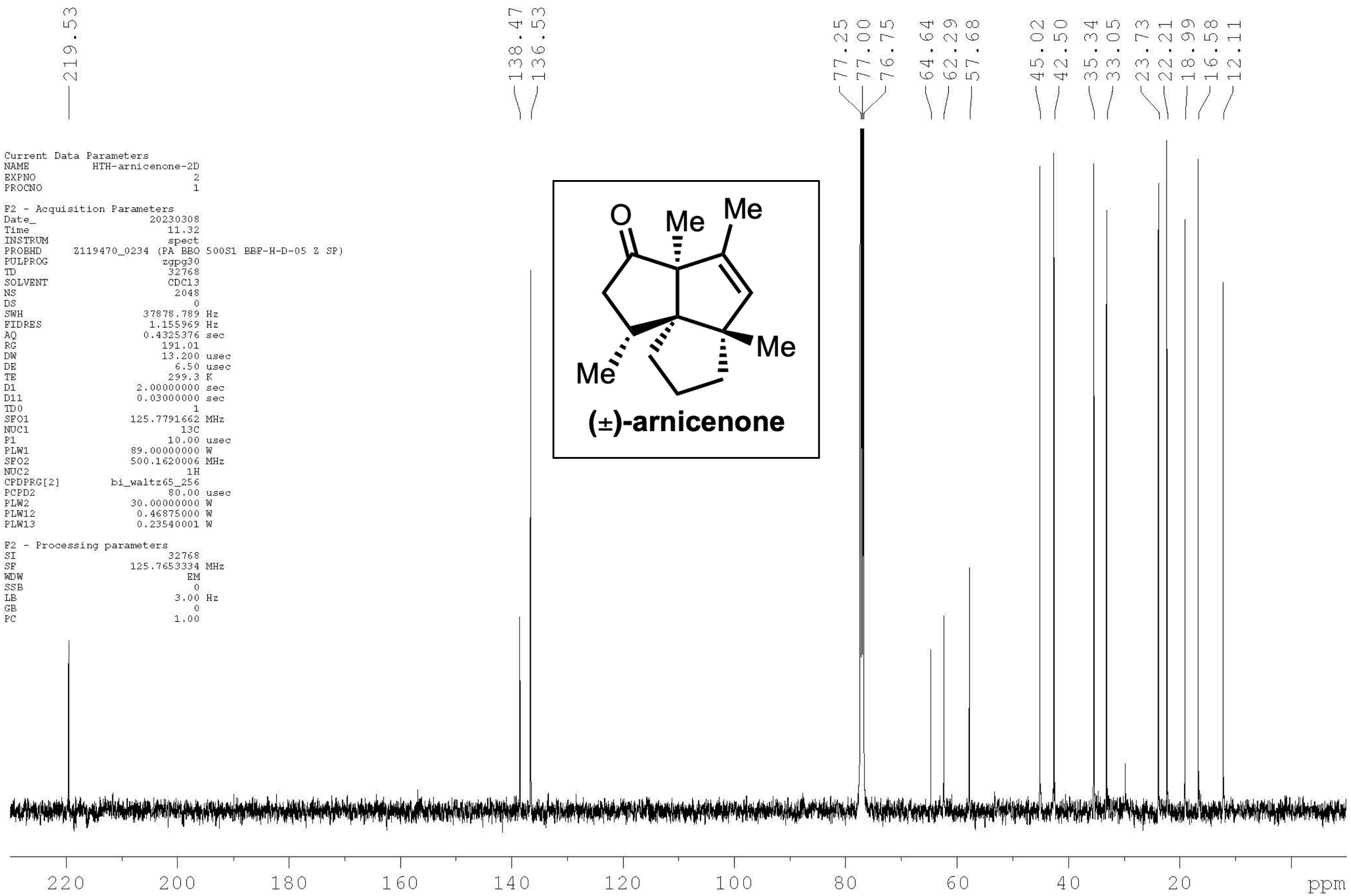

Figure 187. ^{13}C NMR (125 MHz, $CDCl_3$) (**3**)(**arnicenone**)

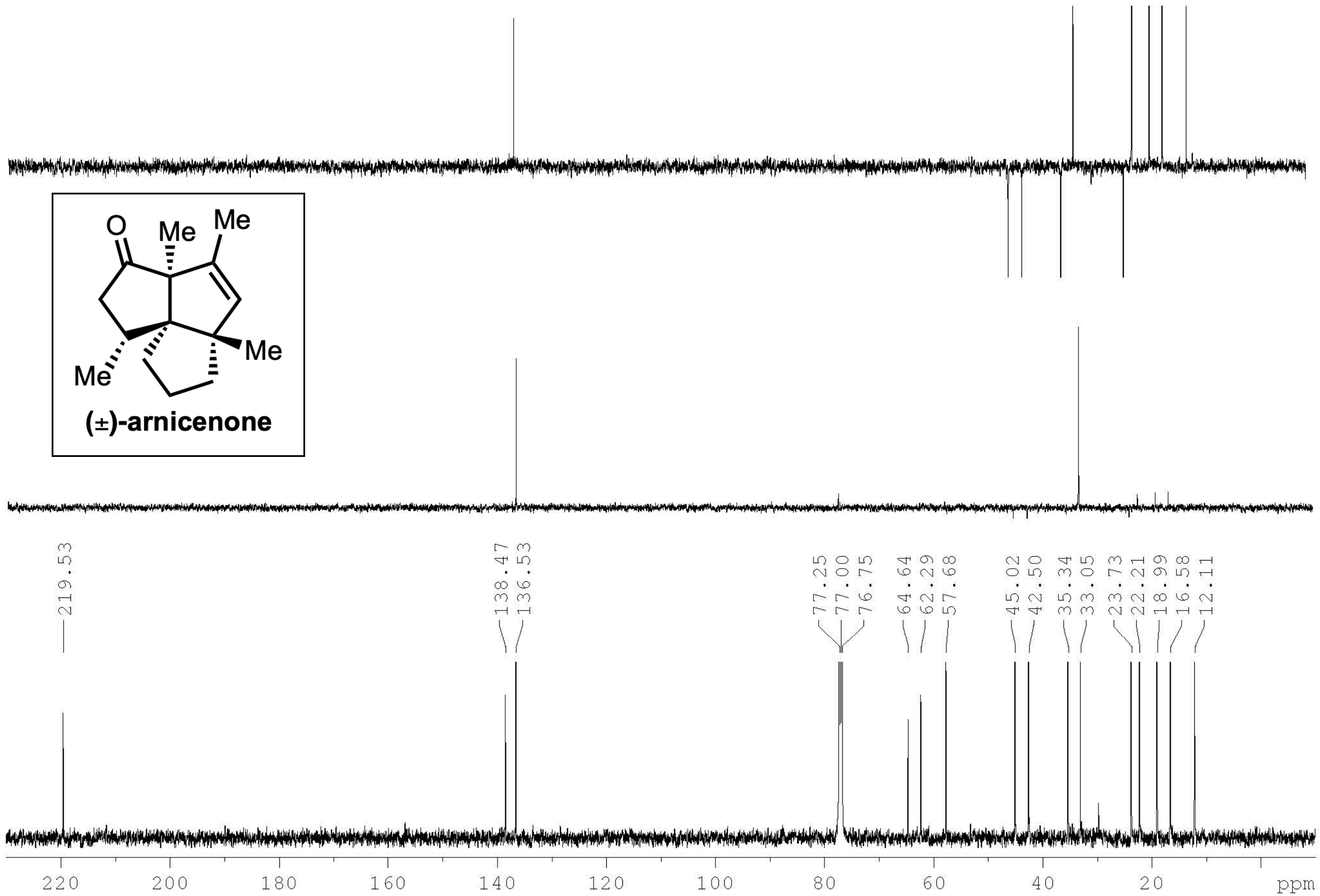

Figure 188. ^{13}C, DEPT-90, DEPT-135 NMR (125 MHz, $CDCl_3$) (**3**)(**arnicenone**)

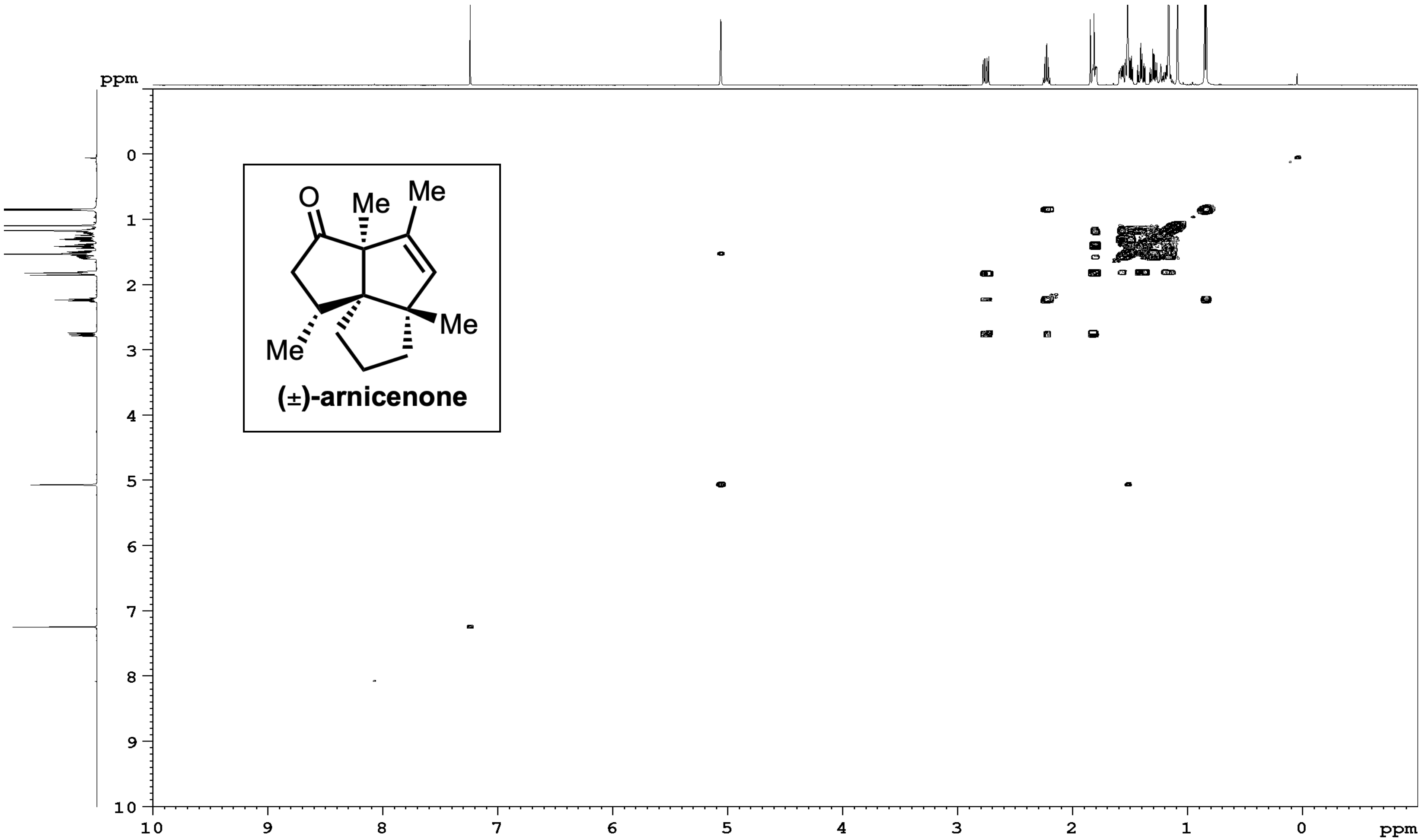

Figure 189. ^{1}H COSY NMR (500 MHz, $CDCl_3$) (**3**)(**arnicenone**)

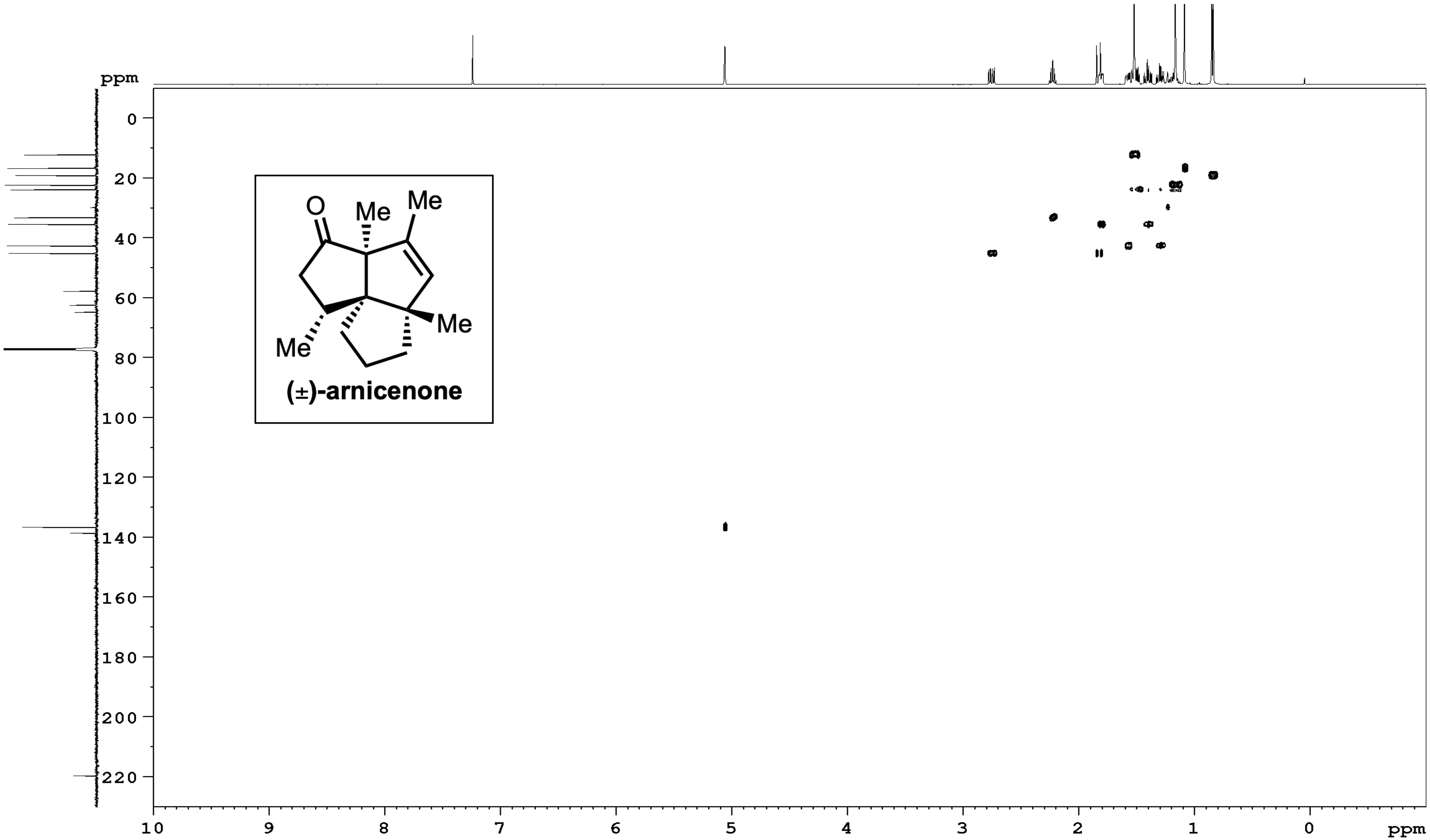

Figure 190. ^{1}H-^{13}C HSQC NMR (500 MHz, $CDCl_3$) (**3**)(**arnicenone**)

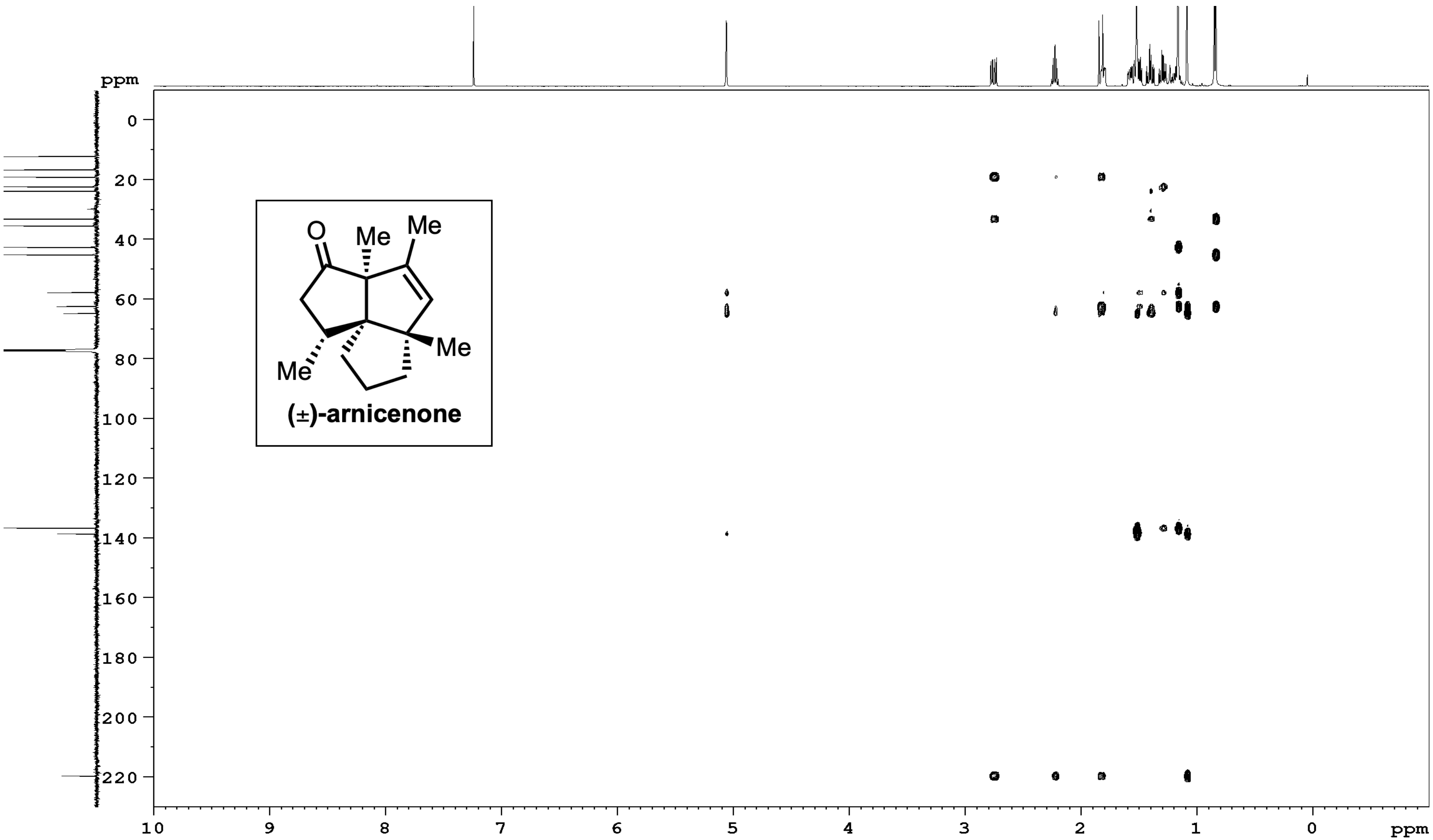

Figure 191. ^{1}H-^{13}C HMBC NMR (500 MHz, $CDCl_3$) (**3**)(**arnicenone**)

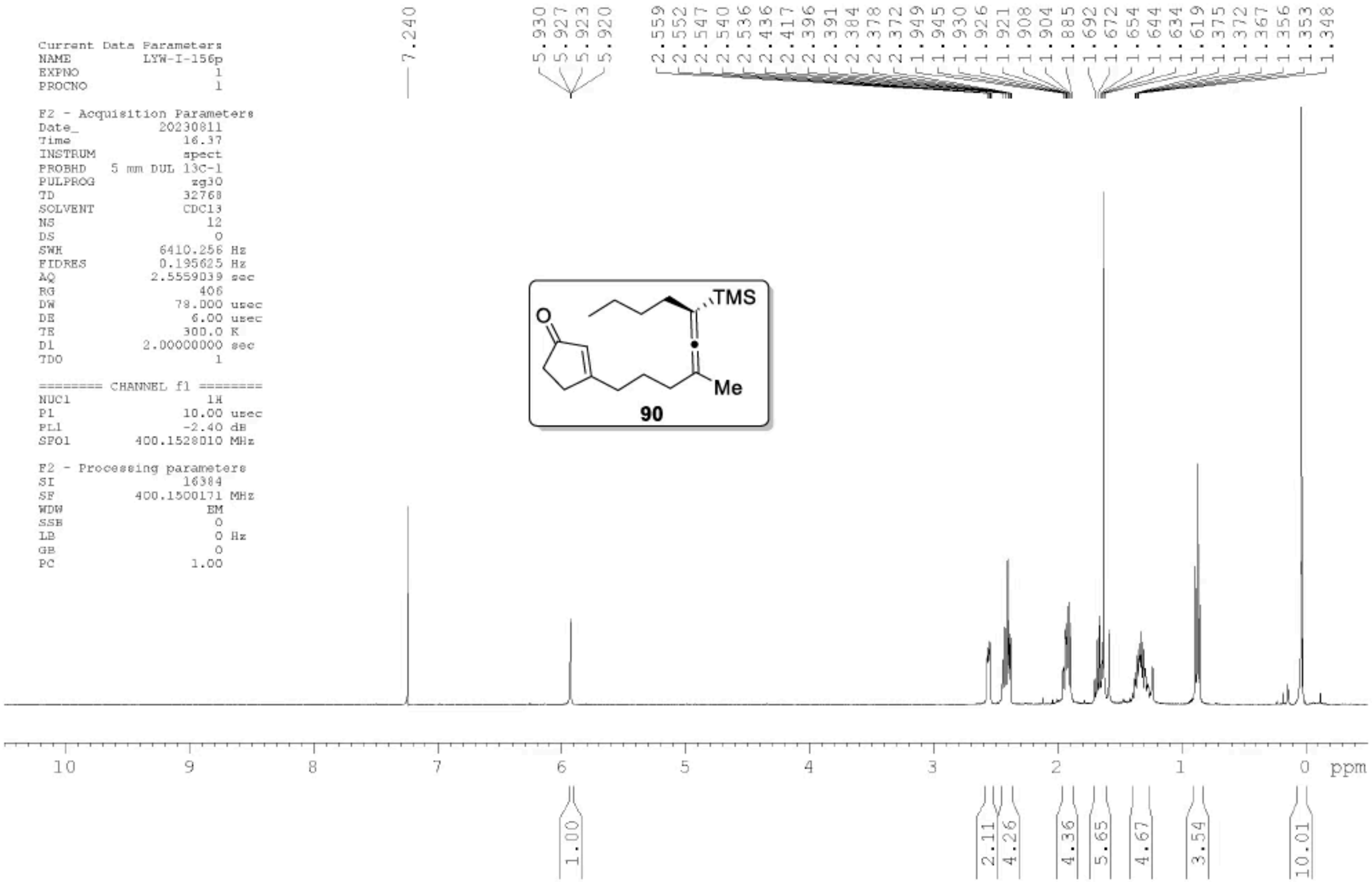

Figure 192. ^{1}H NMR (400 MHz, $CDCl_3$) (**SI-3e**)

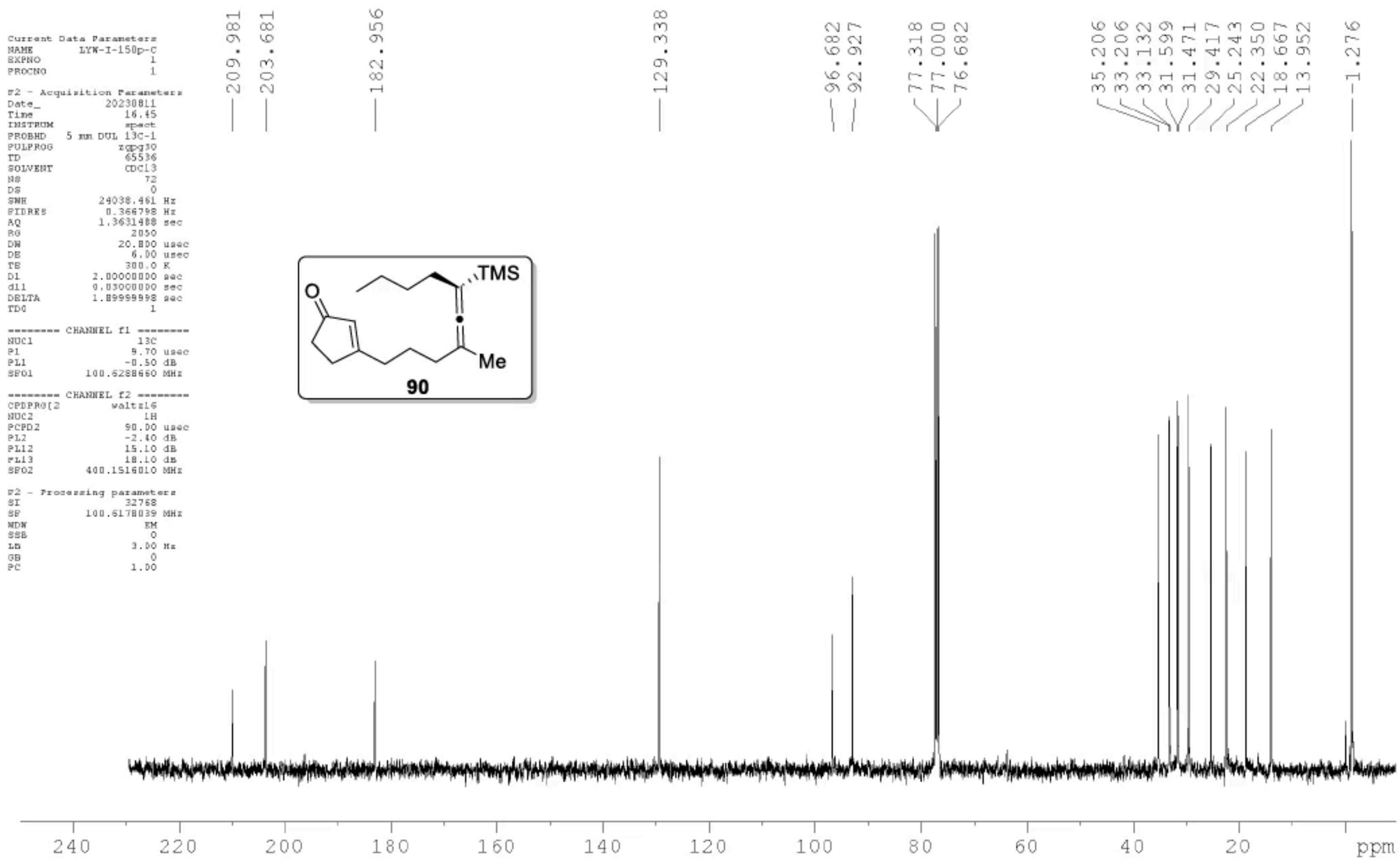

Figure 193. ^{13}C NMR (100 MHz, $CDCl_3$) (**SI-3e**)

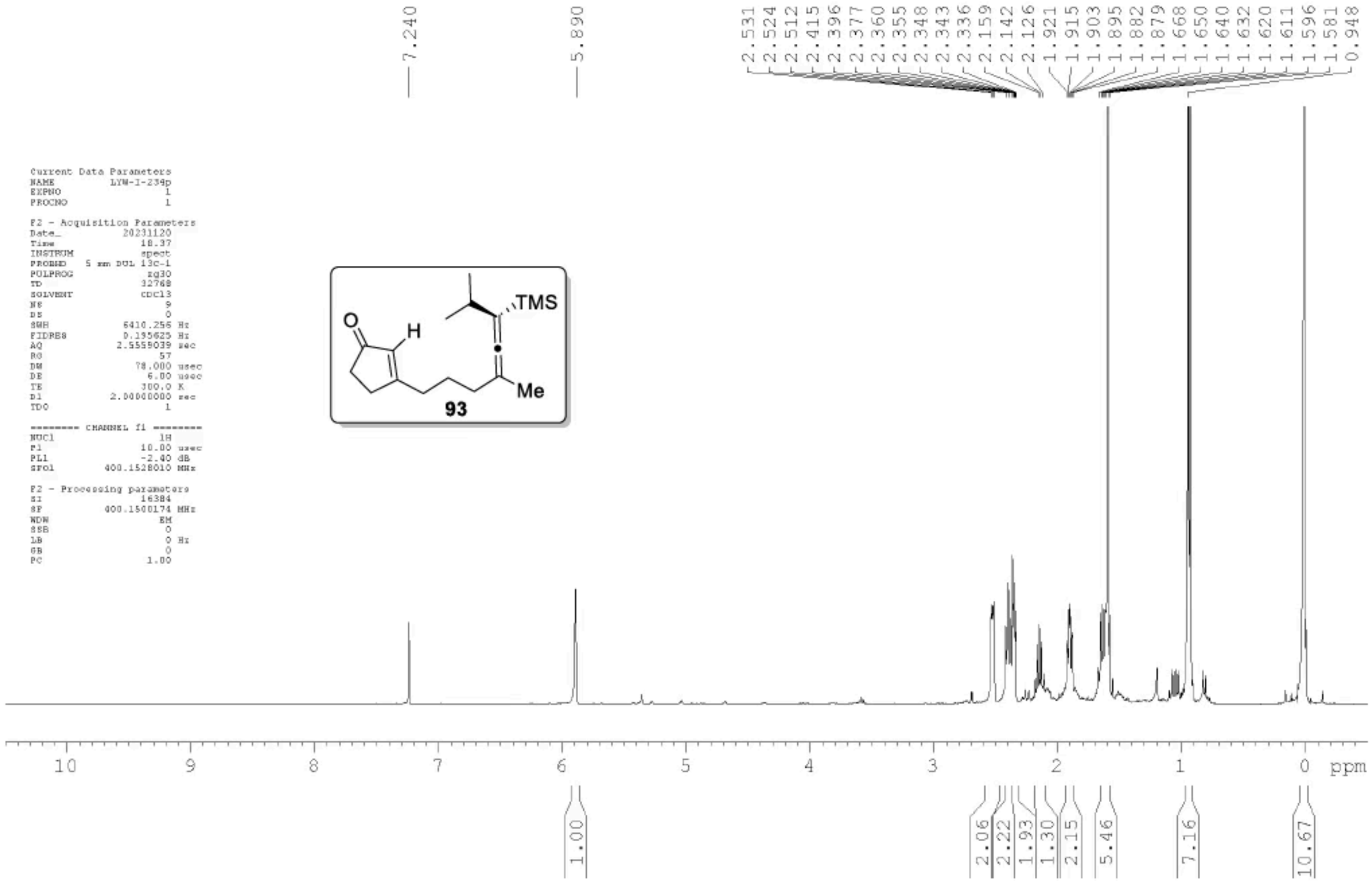

Figure 194. ^{1}H NMR (400 MHz, $CDCl_3$) (**SI-3f**)

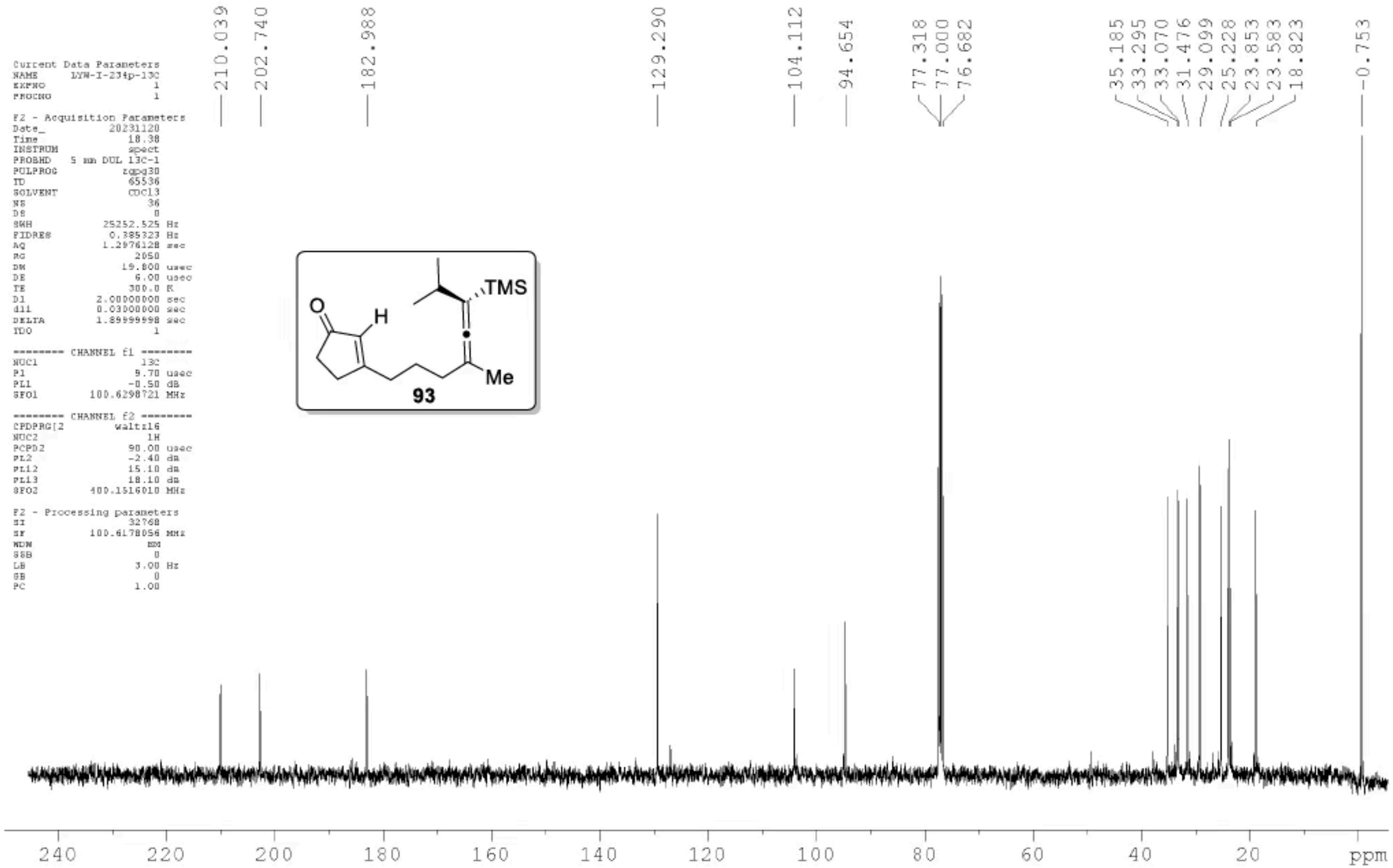

Figure 195. ^{13}C NMR (100 MHz, $CDCl_3$) (**SI-3f**)

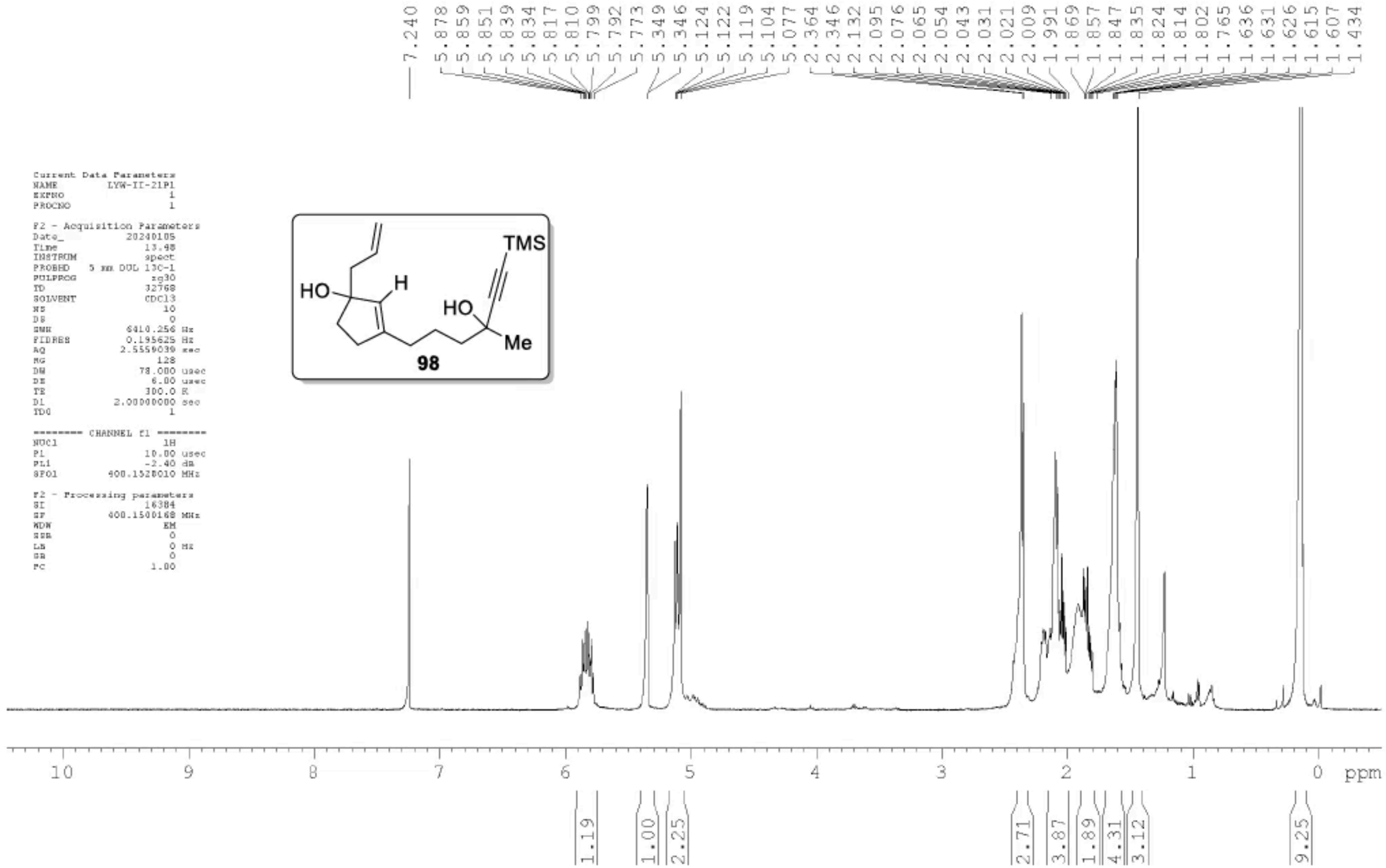

Figure 196. ^{1}H NMR (400 MHz, $CDCl_3$) (**SI-3g**)

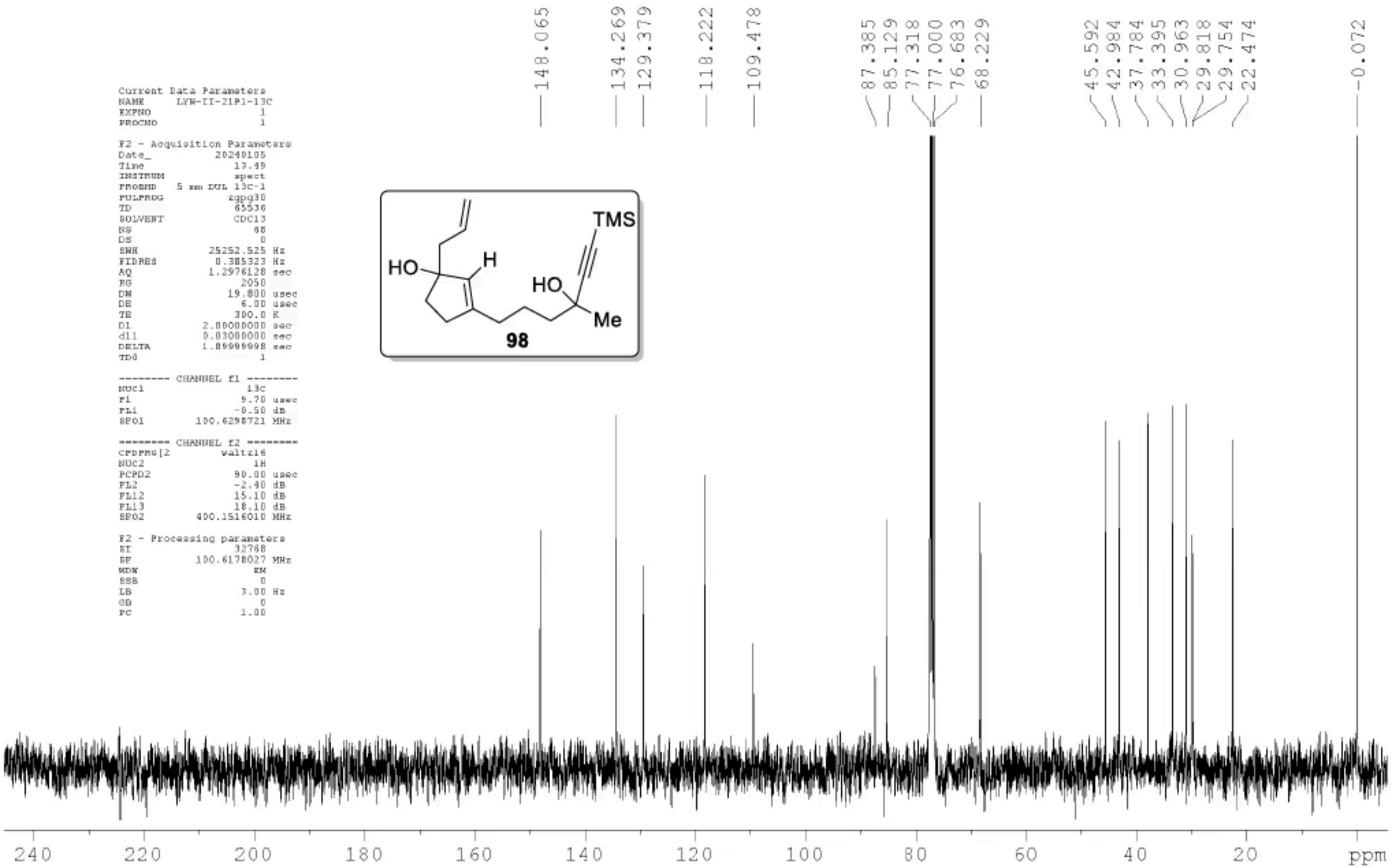

Figure 197. ^{13}C NMR (100 MHz, $CDCl_3$) (**SI-3g**)

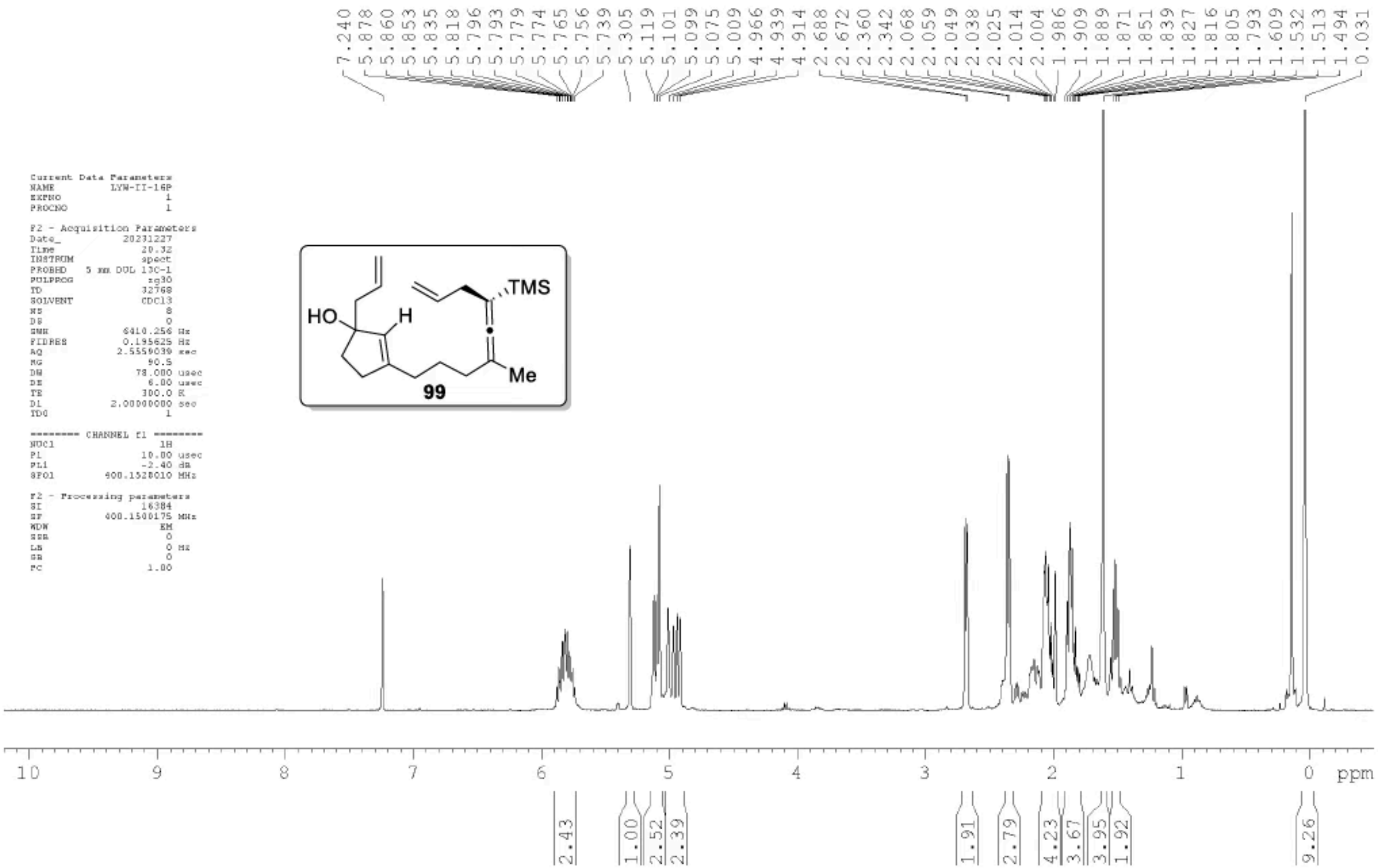

Figure 198. ^{1}H NMR (400 MHz, $CDCl_3$) (**SI-3h**)

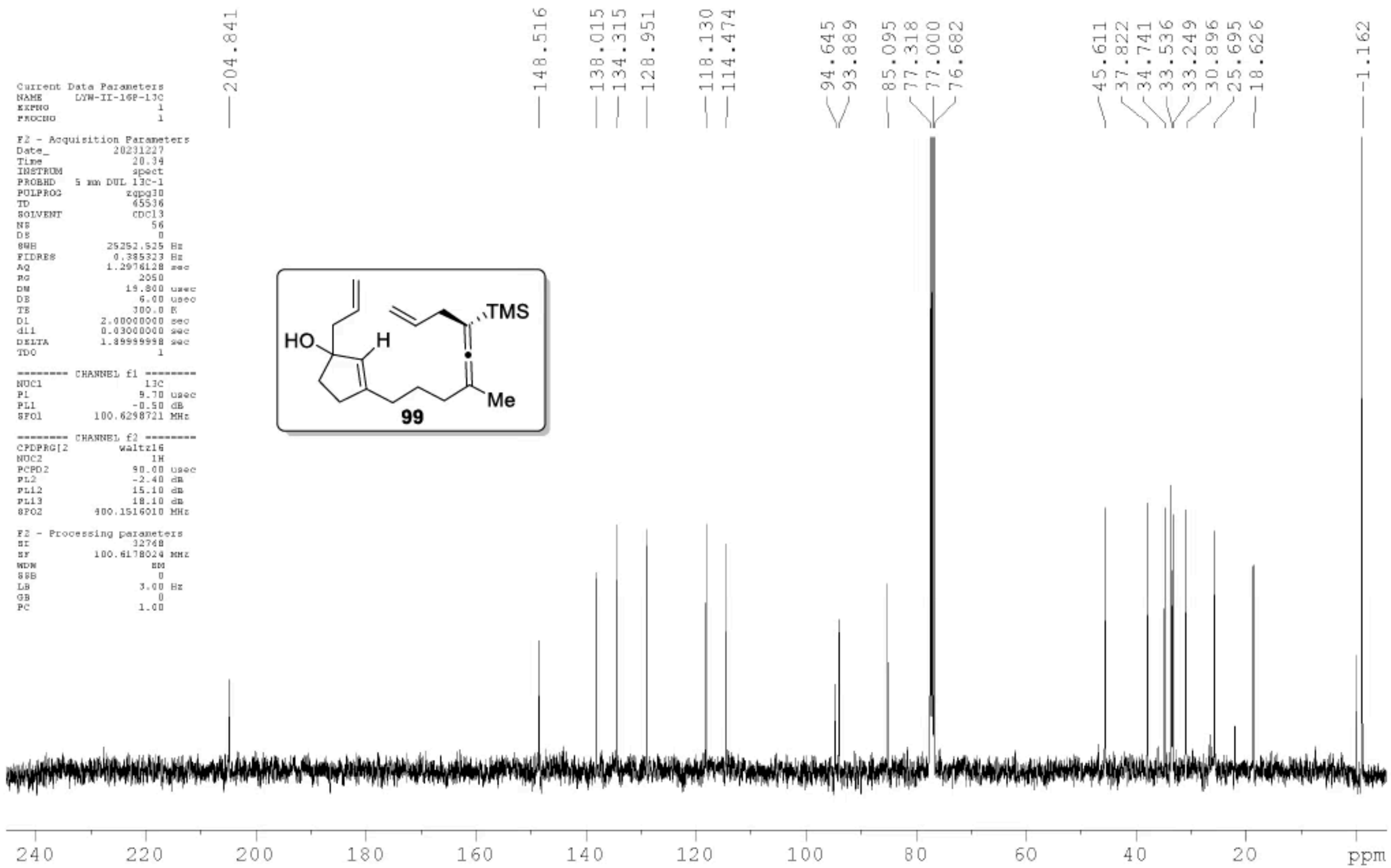

Figure 199. ^{13}C NMR (100 MHz, $CDCl_3$) (**SI-3h**)

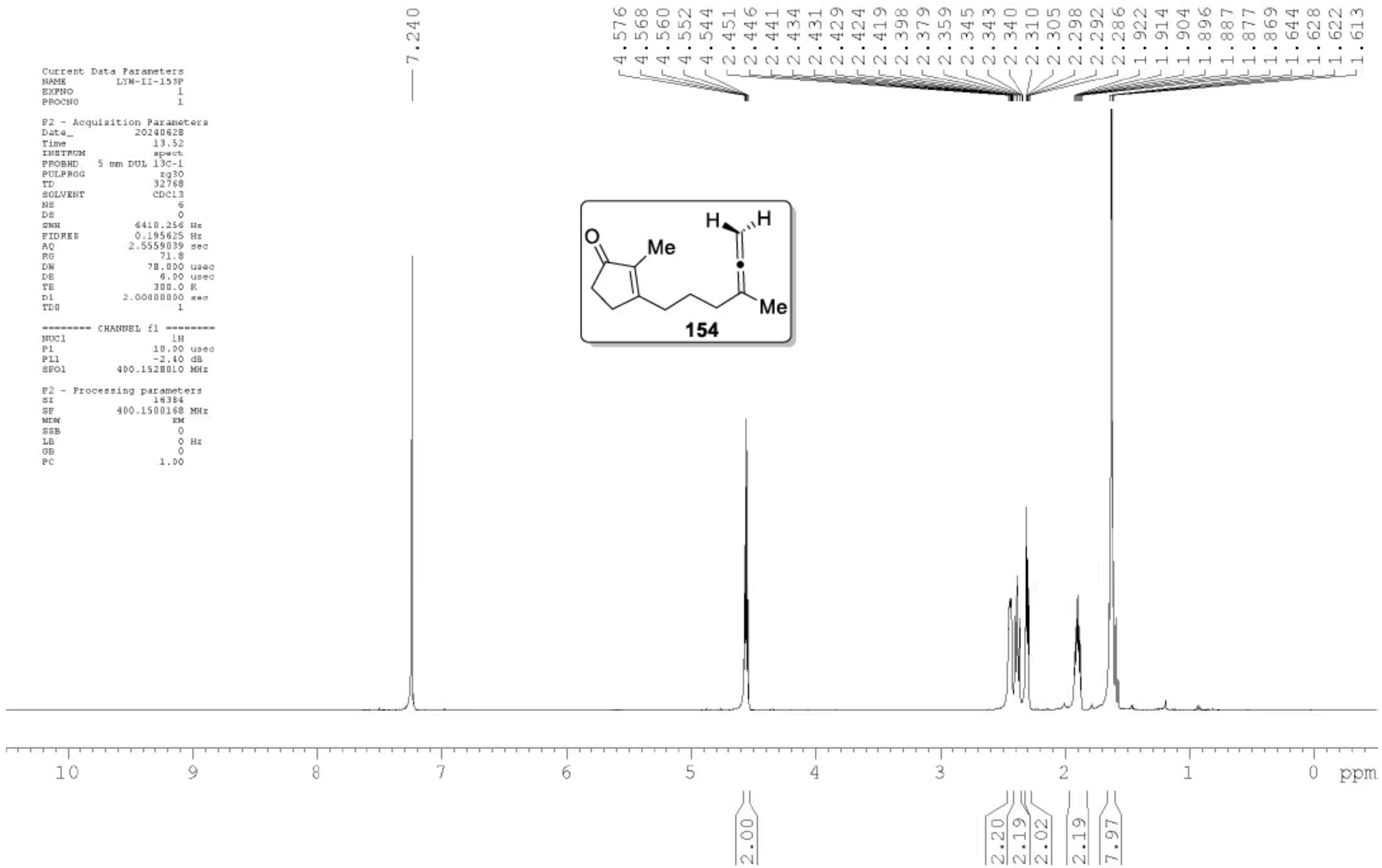

Figure 200. ^{1}H NMR (400 MHz, $CDCl_3$) (**SI-3i**)

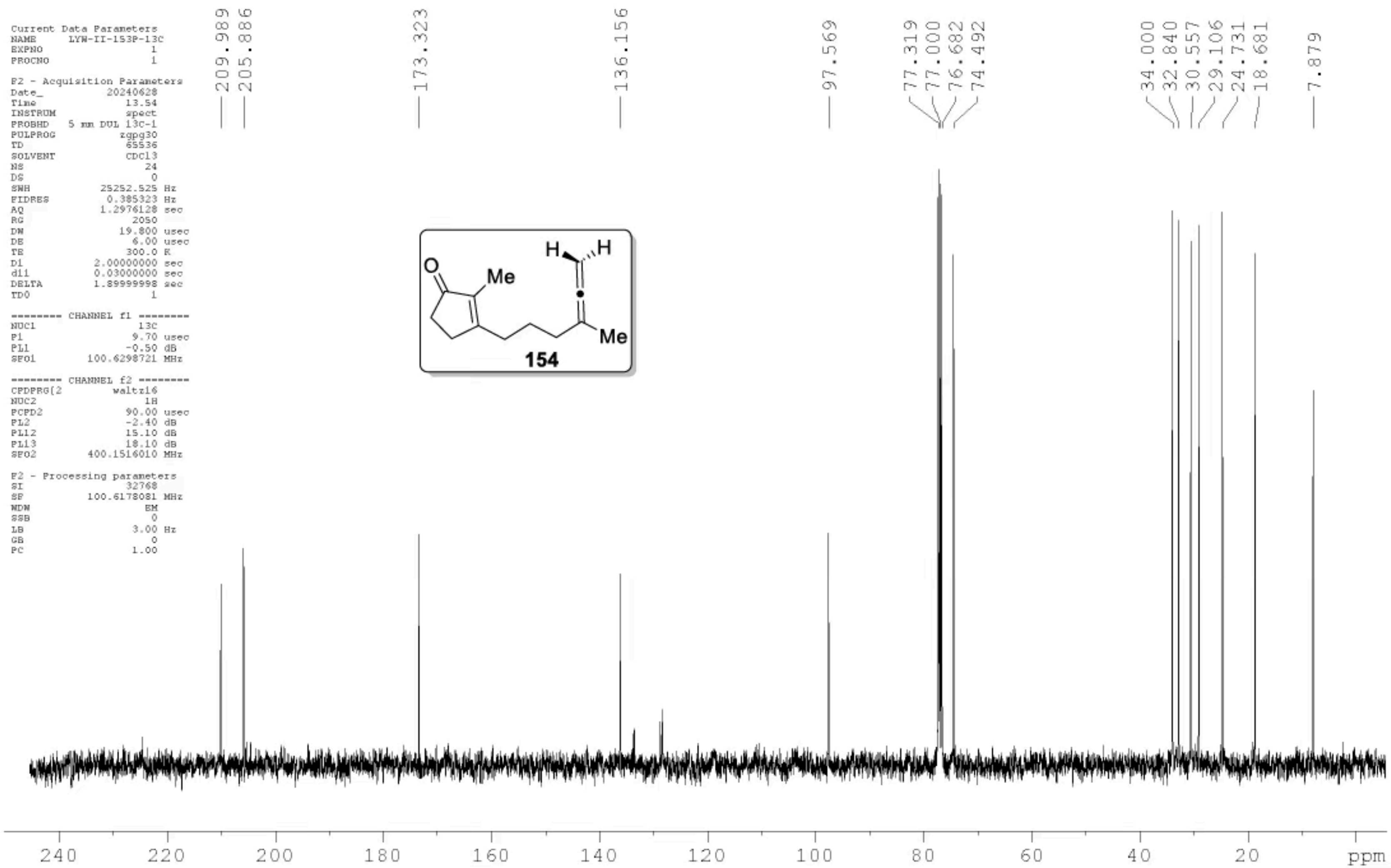

Figure 201. ^{13}C NMR (100 MHz, $CDCl_3$) (**SI-3i**)

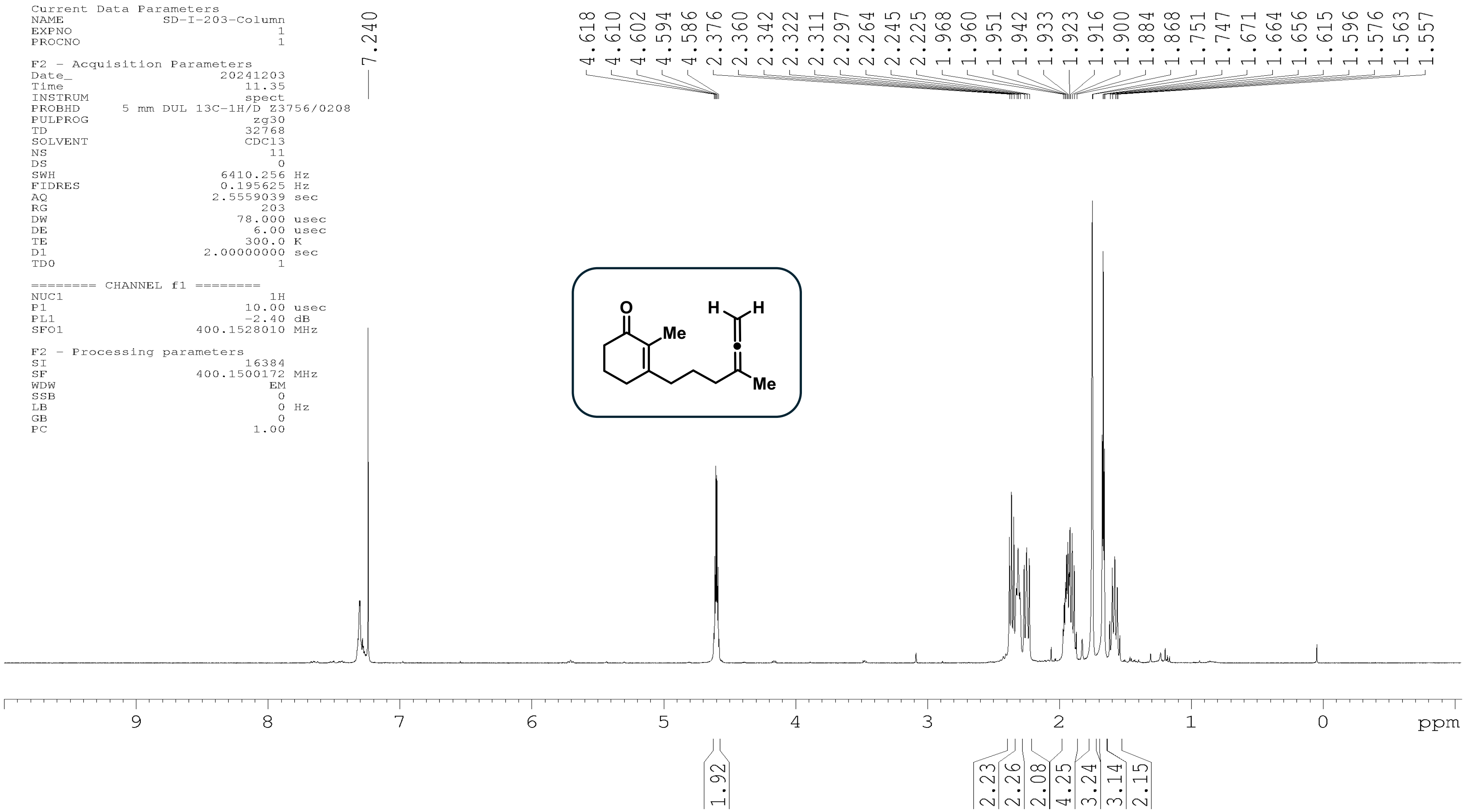

Figure 202. ^{1}H NMR (400 MHz, $CDCl_3$) (**SI-3j**)

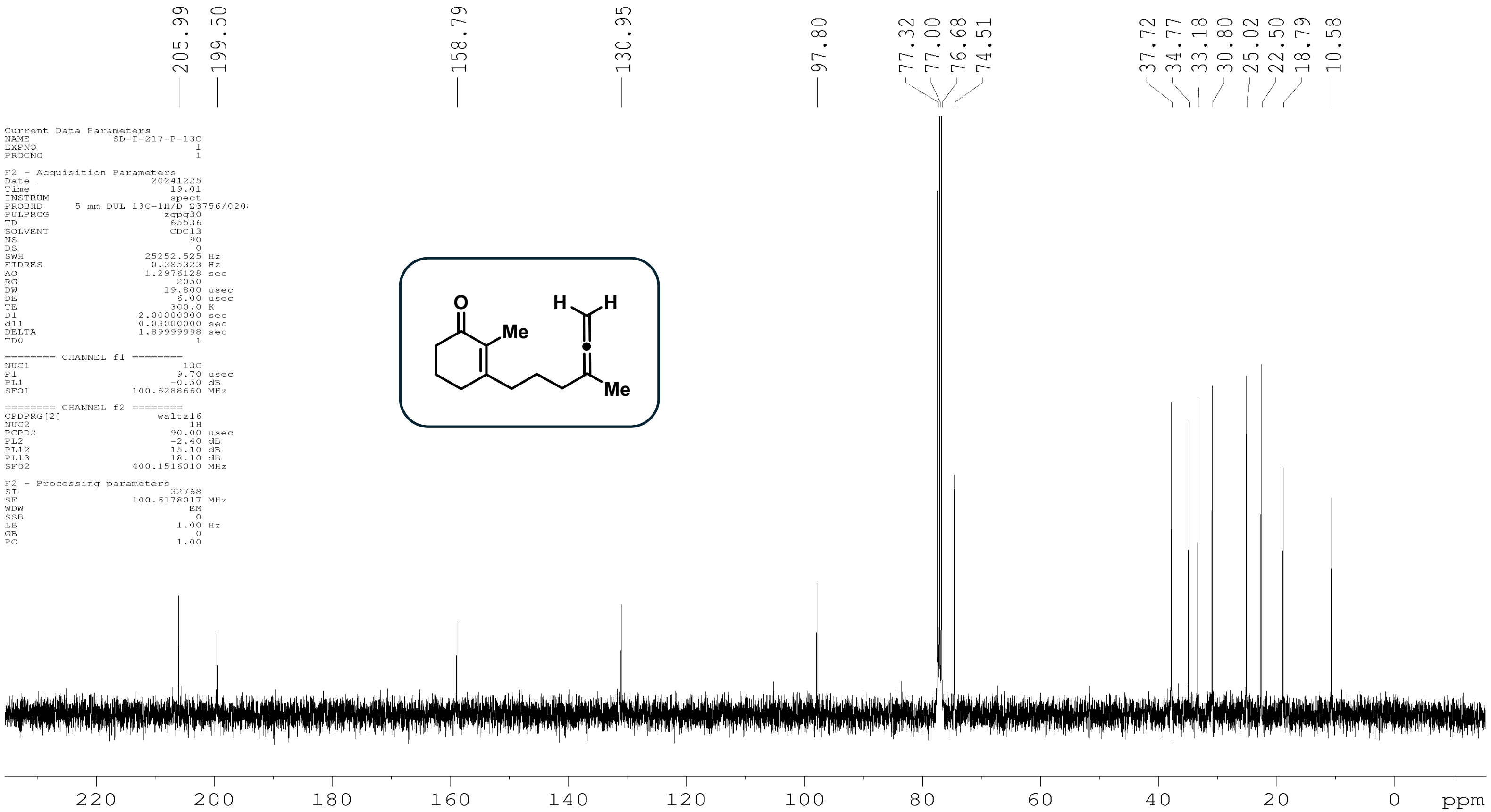

Figure 203. ^{13}C NMR (100 MHz, CDCl$_3$) (**SI-3j**)

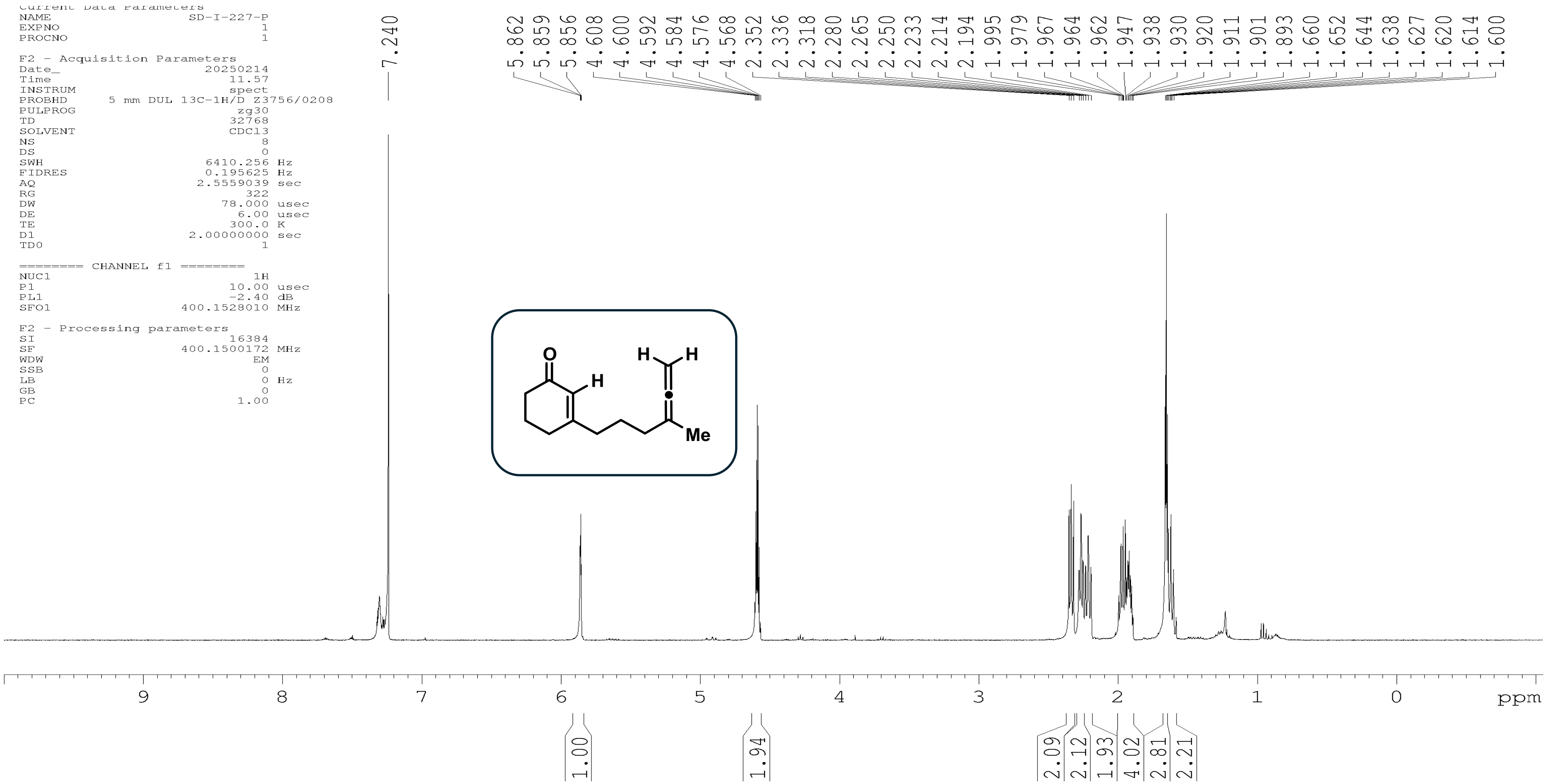

Figure 204. ^{1}H NMR (400 MHz, $CDCl_3$) (**SI-3k**)

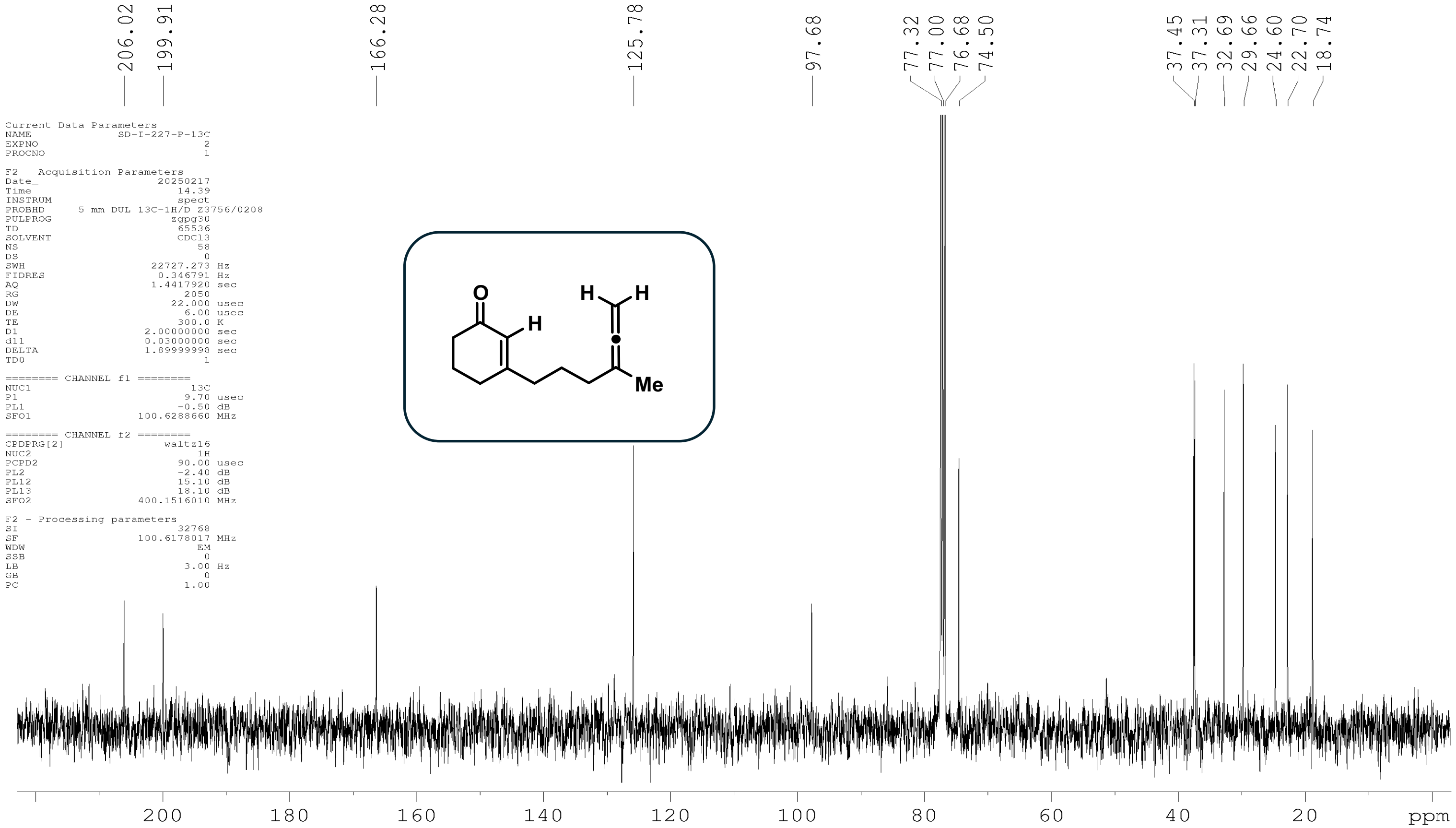

Figure 205. ^{13}C NMR (100 MHz, $CDCl_3$) (**SI-3k**)

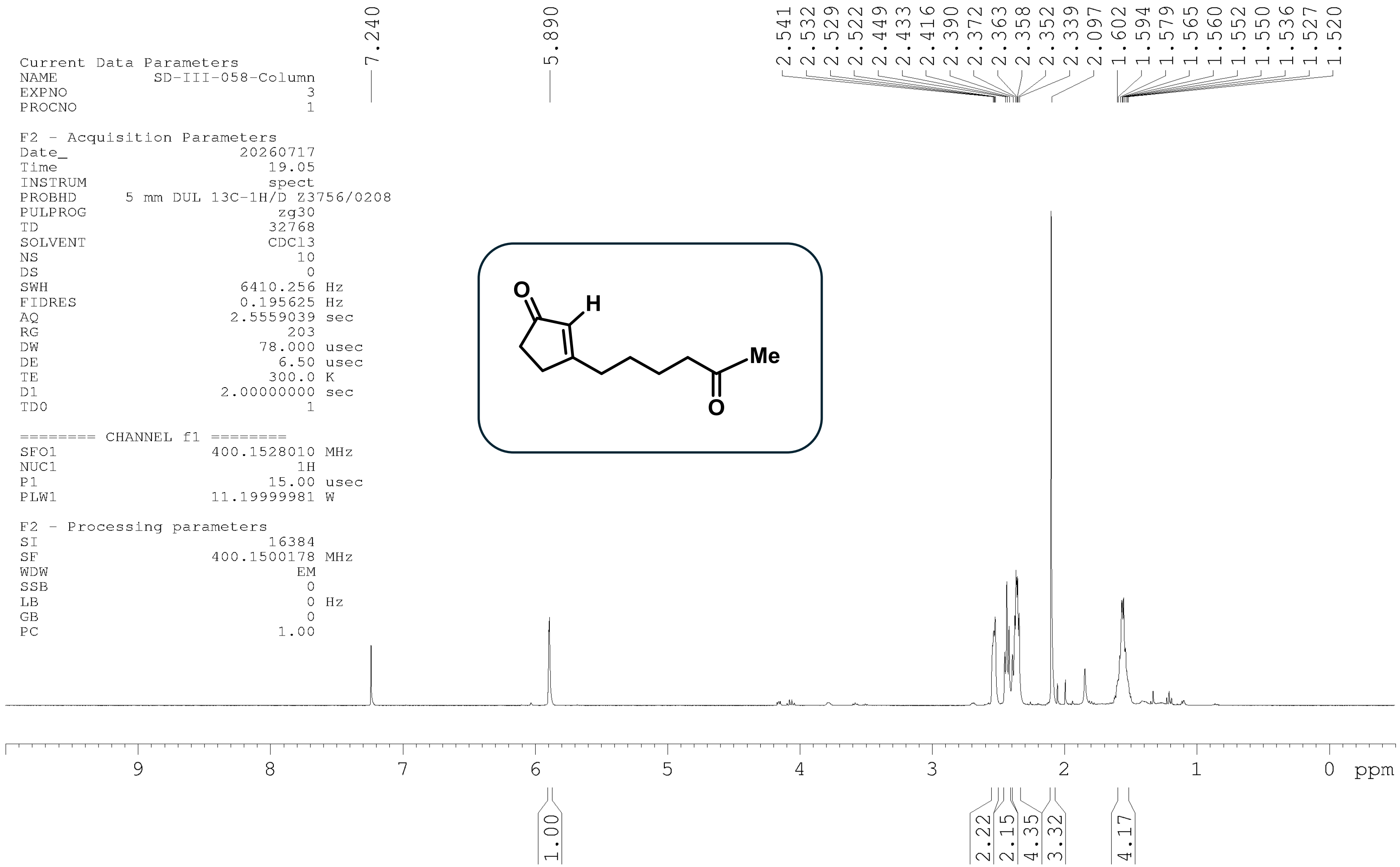

Figure 206. ^{1}H NMR (400 MHz, $CDCl_3$) (**SI-5**)

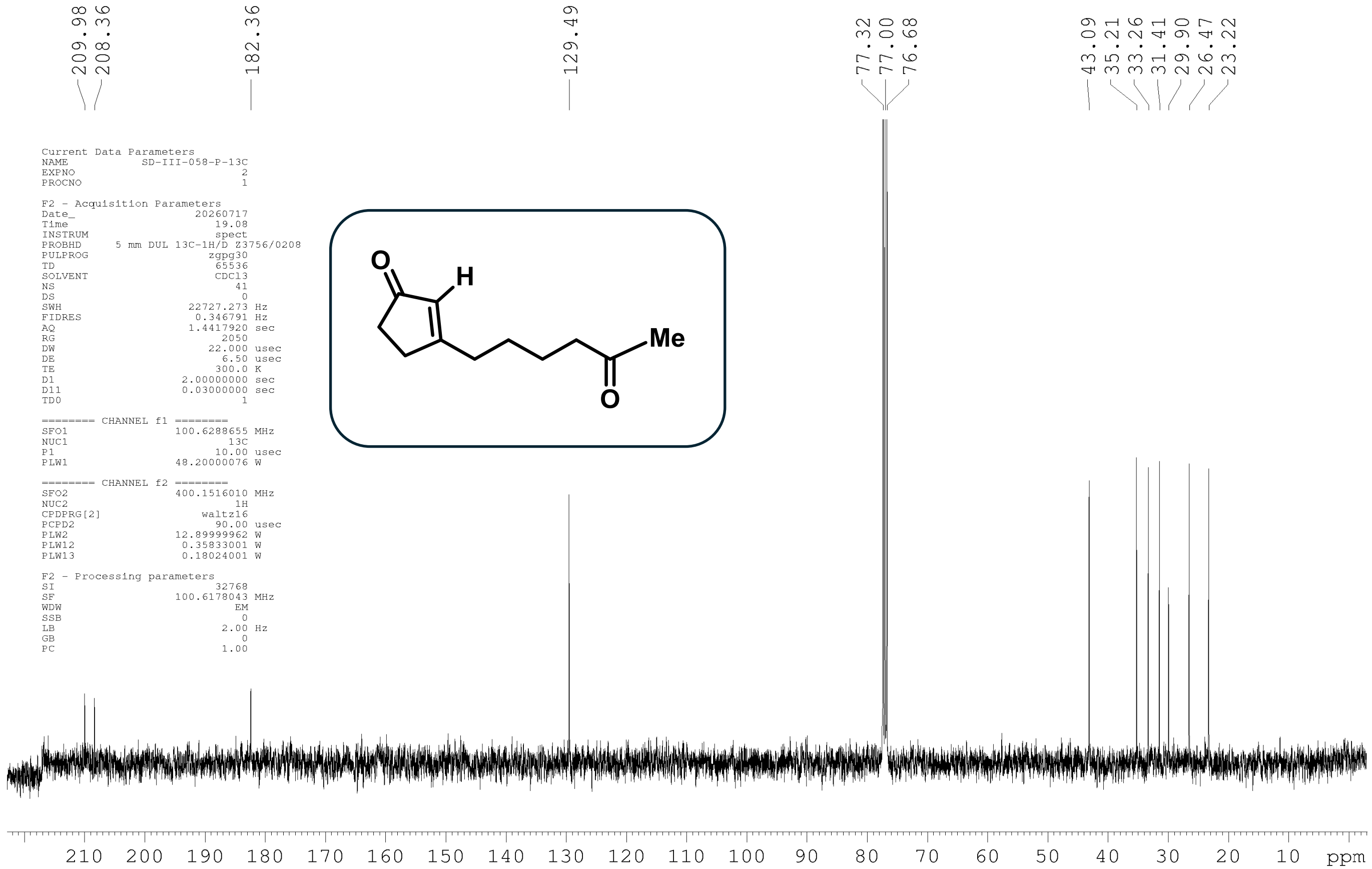

Figure 207. ^{13}C NMR (100 MHz, $CDCl_3$) (**SI-5**)

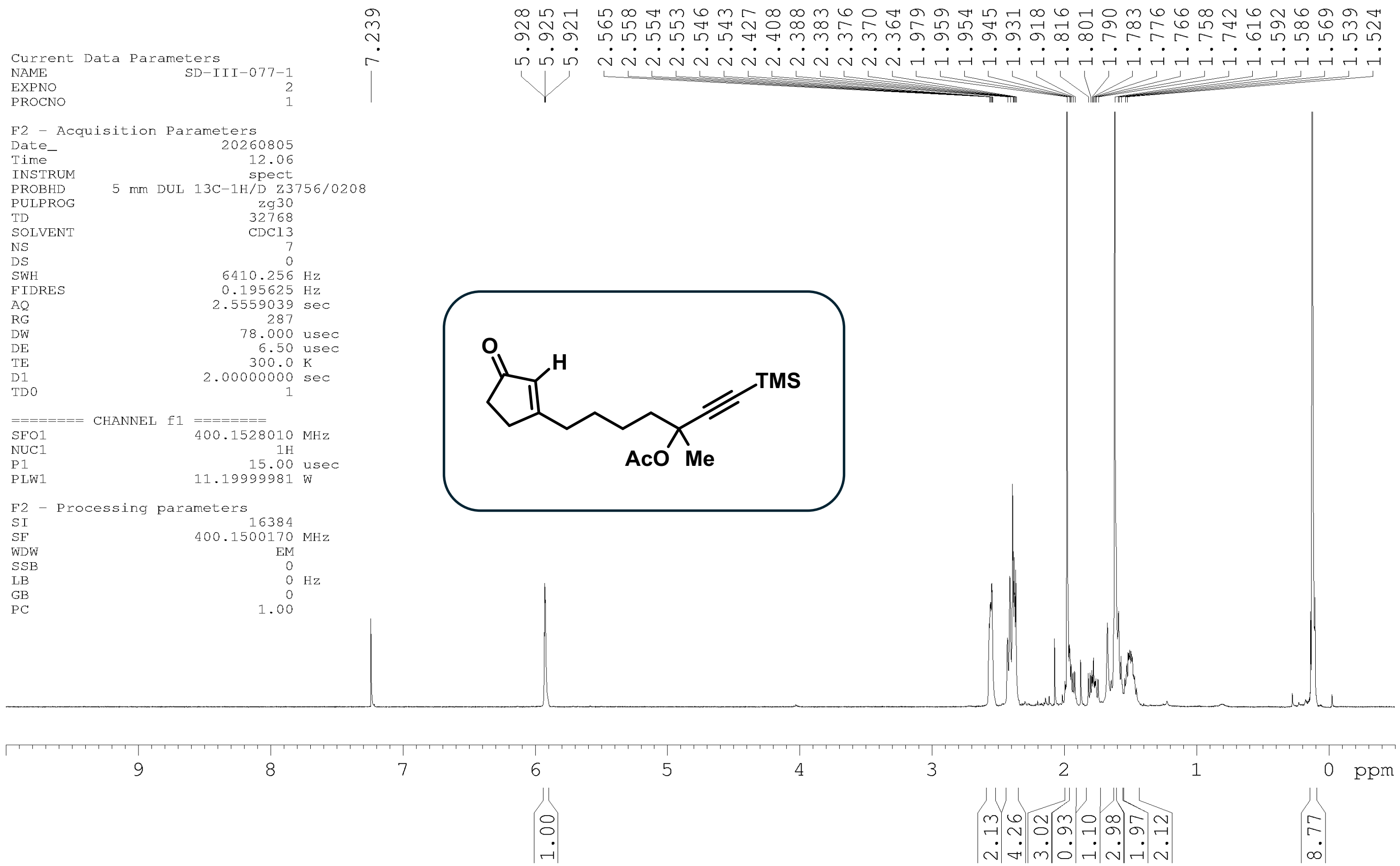

Figure 208. ^{1}H NMR (400 MHz, $CDCl_3$) (**SI-6**)

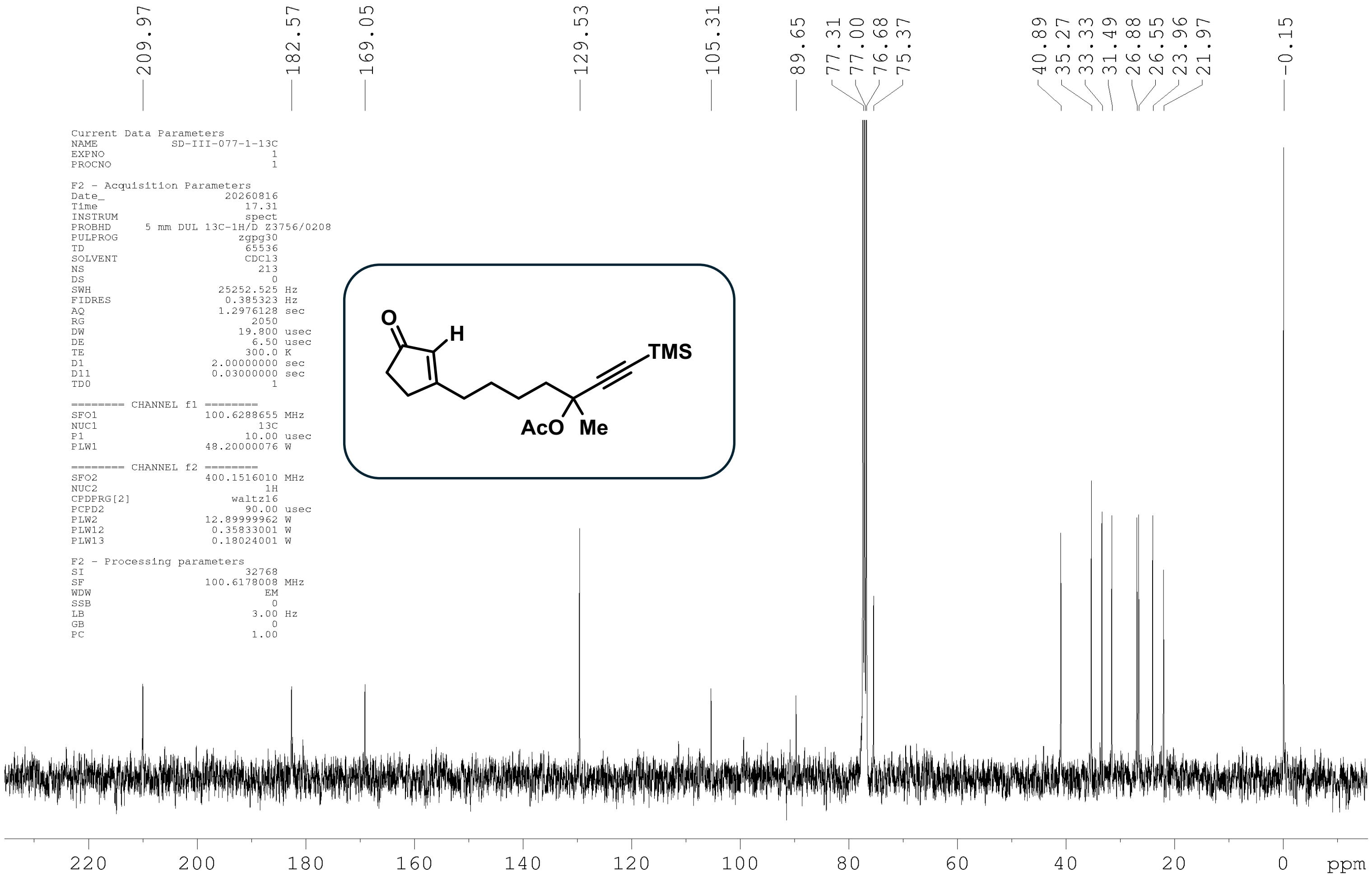

Figure 209. ^{13}C NMR (100 MHz, $CDCl_3$) (**SI-6**)

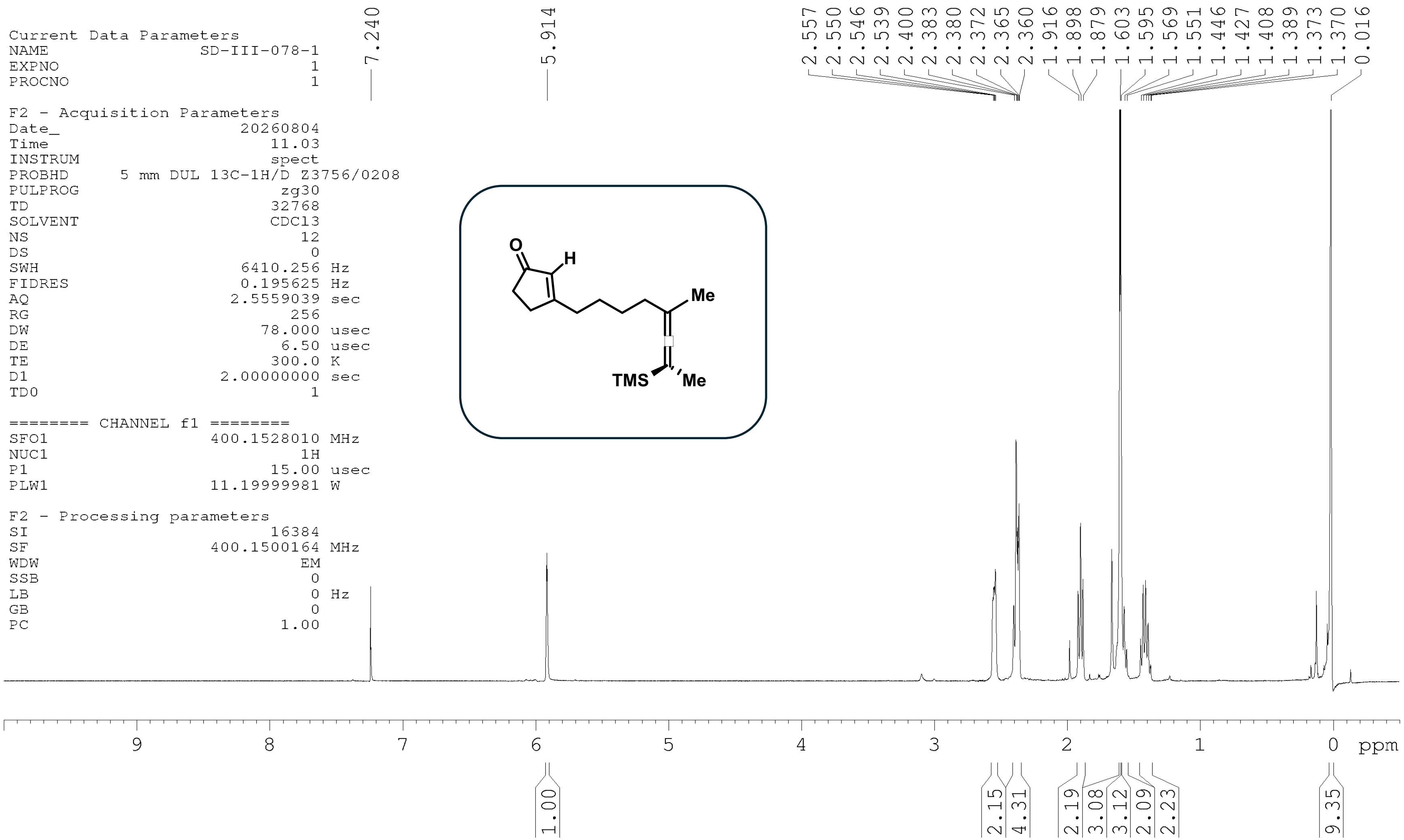

Figure 210. ^{1}H NMR (400 MHz, $CDCl_3$) (**SI-7**)

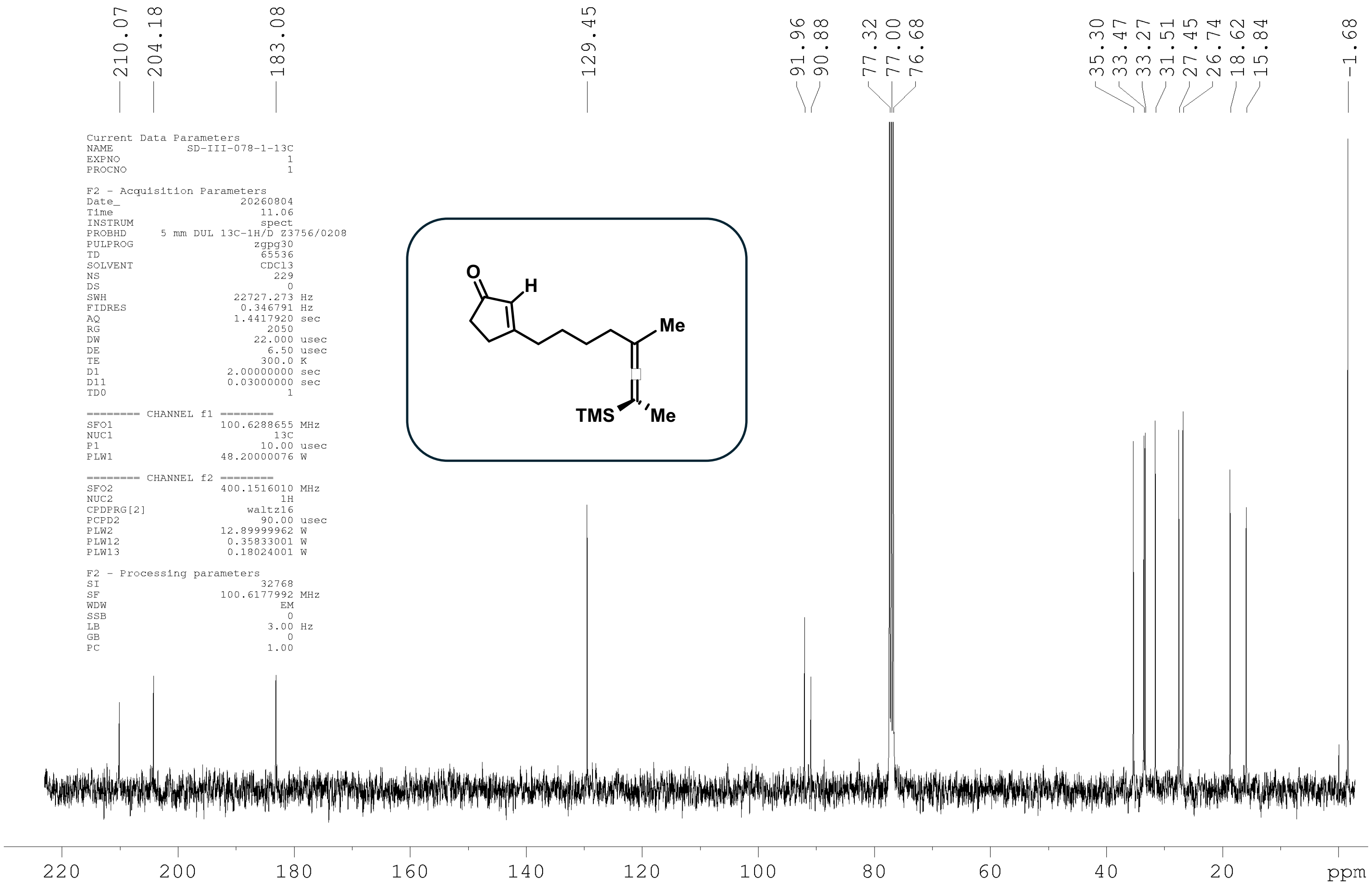

Figure 211. ^{13}C NMR (100 MHz, $CDCl_3$) (**SI-7**)

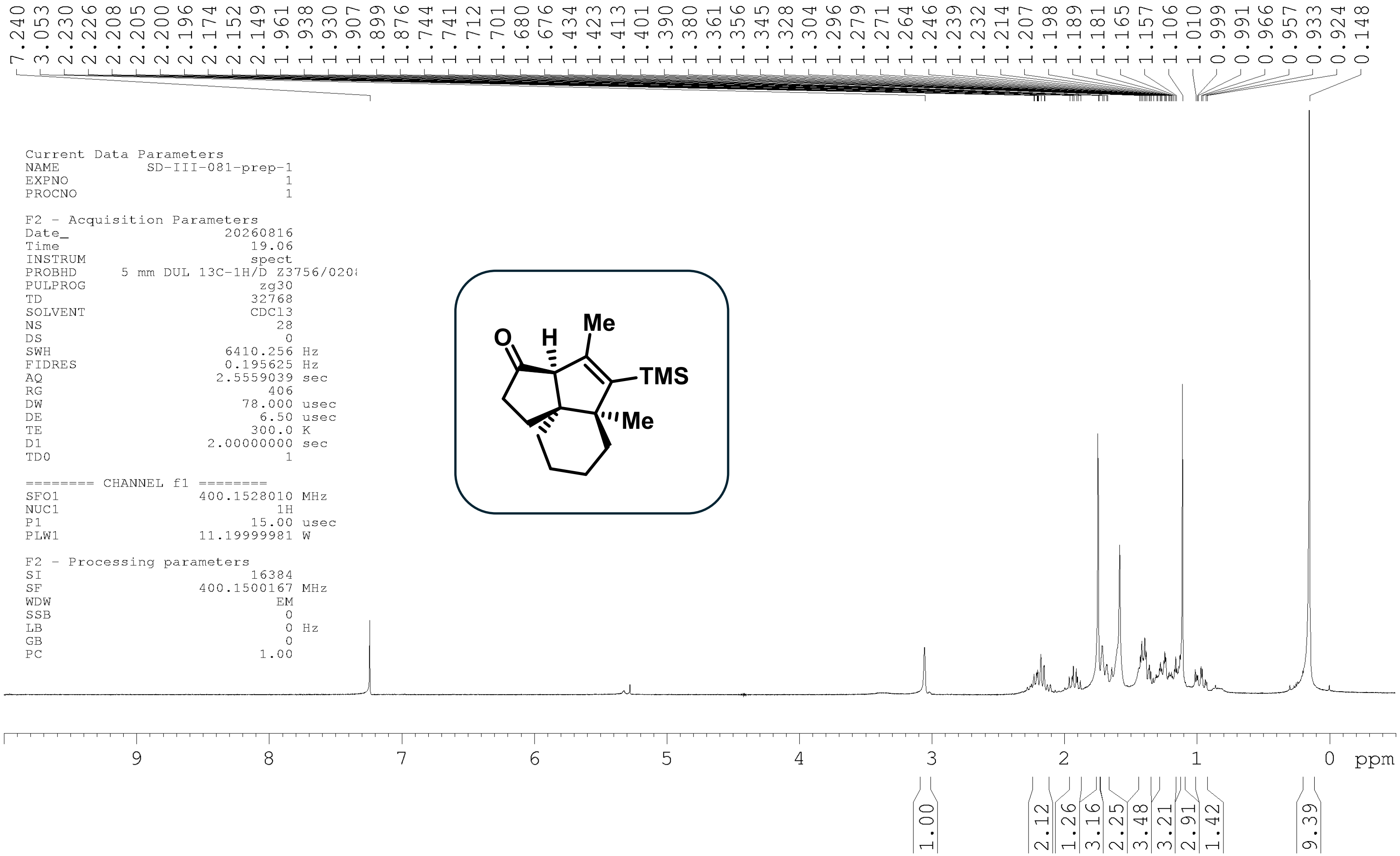

Figure 212. ^{1}H NMR (400 MHz, $CDCl_3$) (**SI-8**)

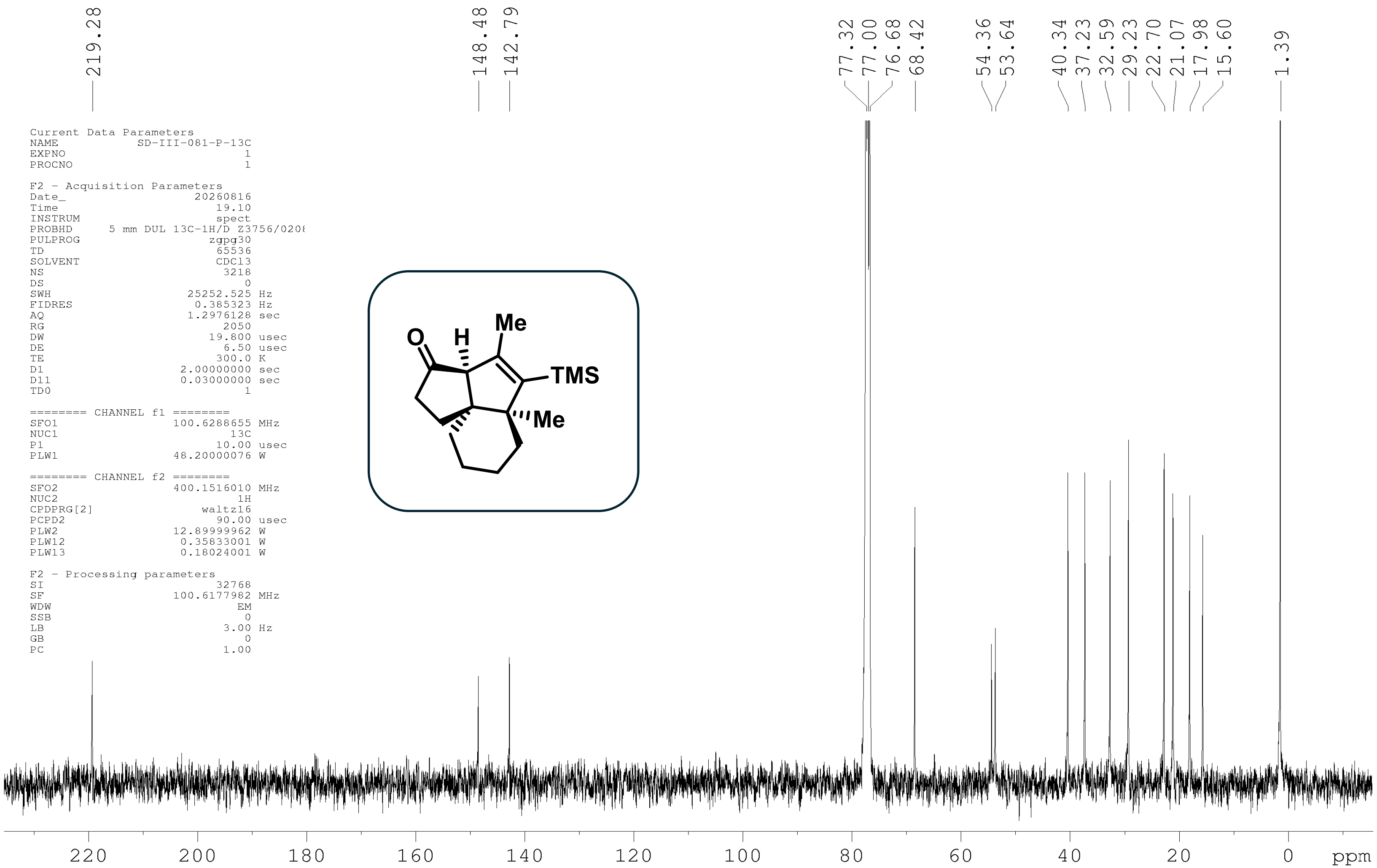

Figure 213. ^{13}C NMR (100 MHz, $CDCl_3$) (**SI-8**)

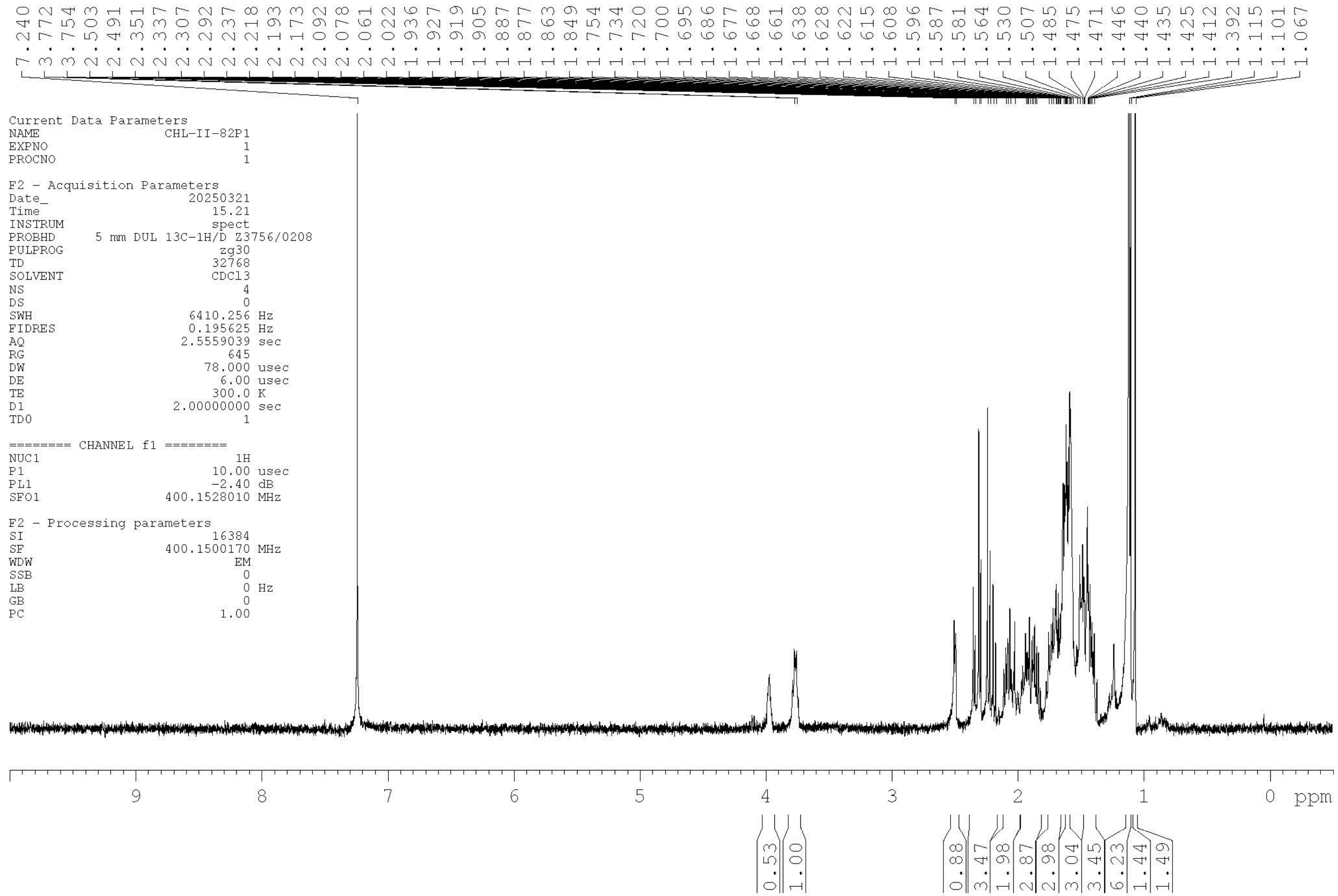

Figure 214. ^{1}H NMR (400 MHz, $CDCl_3$) (**39**)

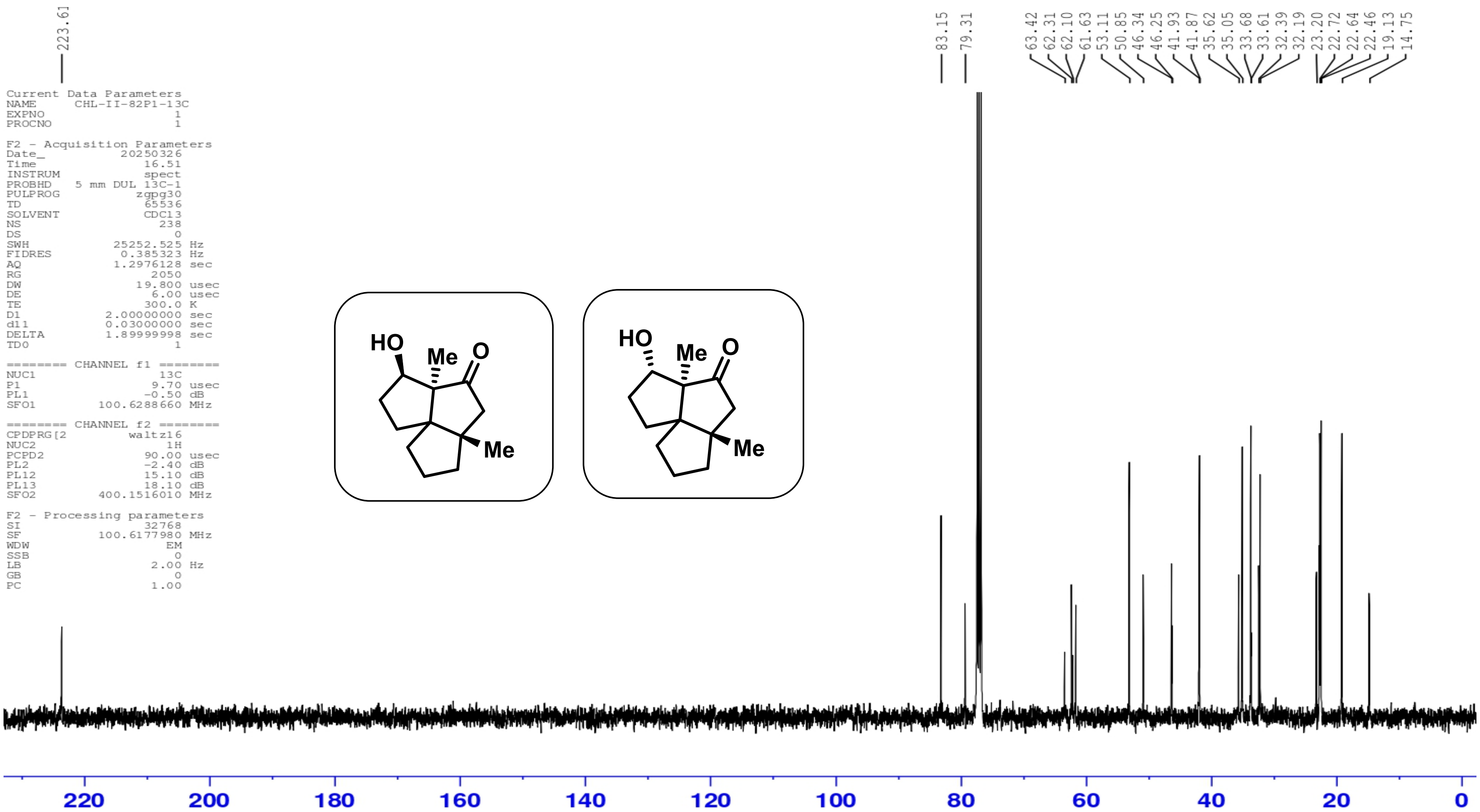

Figure 215. ^{13}C NMR (100 MHz, $CDCl_3$) (**39**)

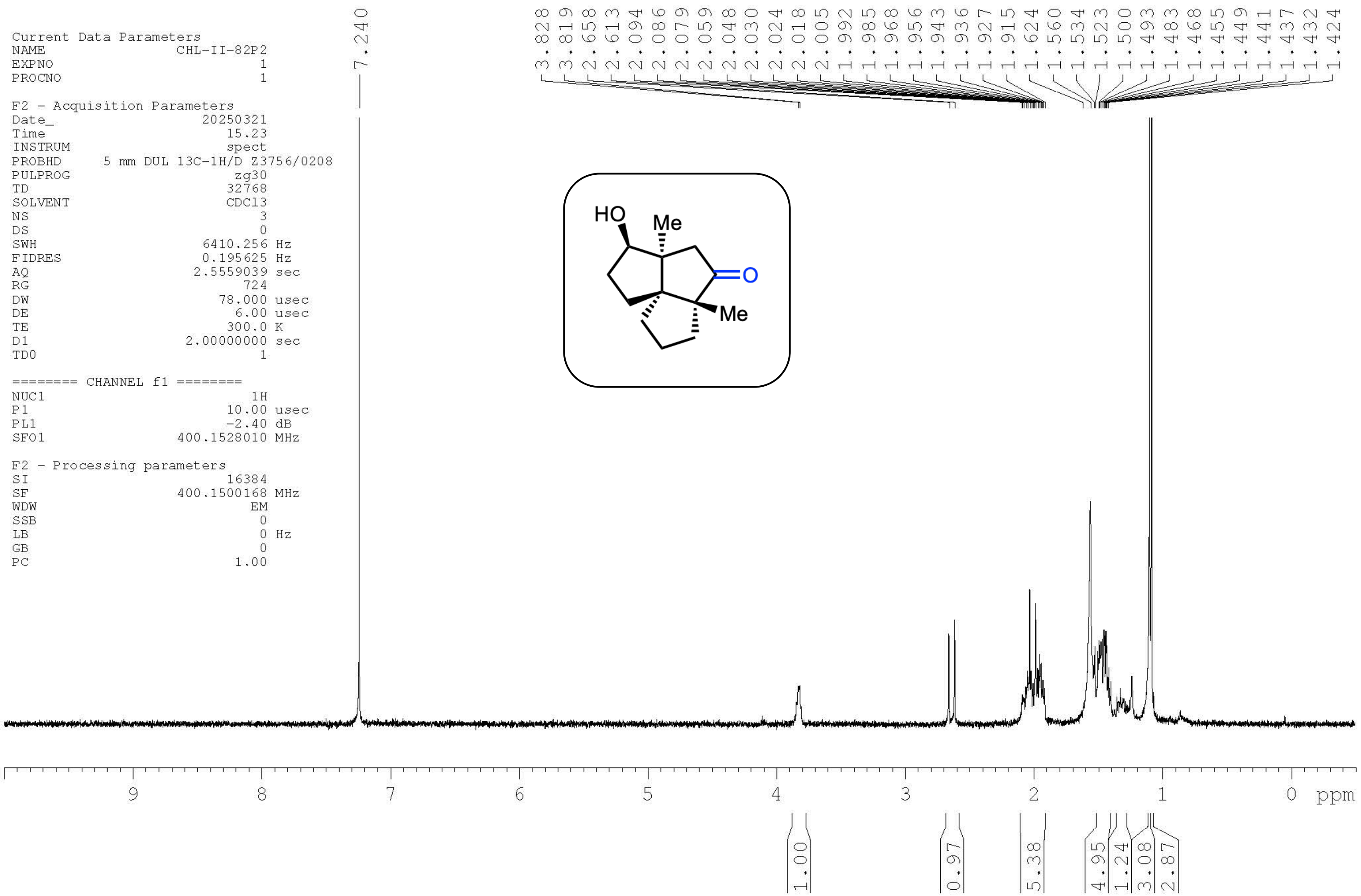

Figure 216. ^{1}H NMR (400 MHz, $CDCl_3$) (**40**)

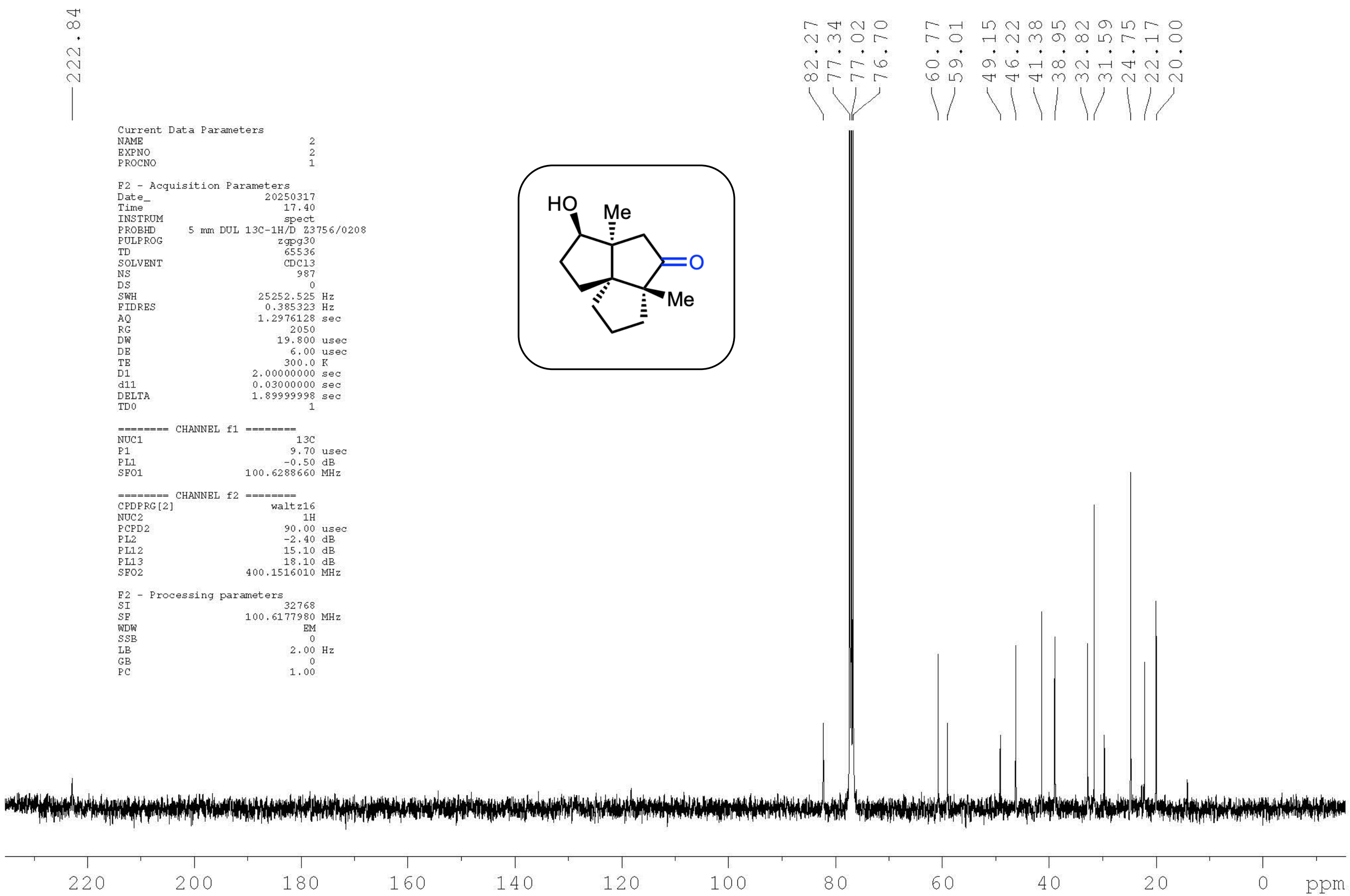

Figure 217. ^{13}C NMR (100 MHz, $CDCl_3$) (**40**)

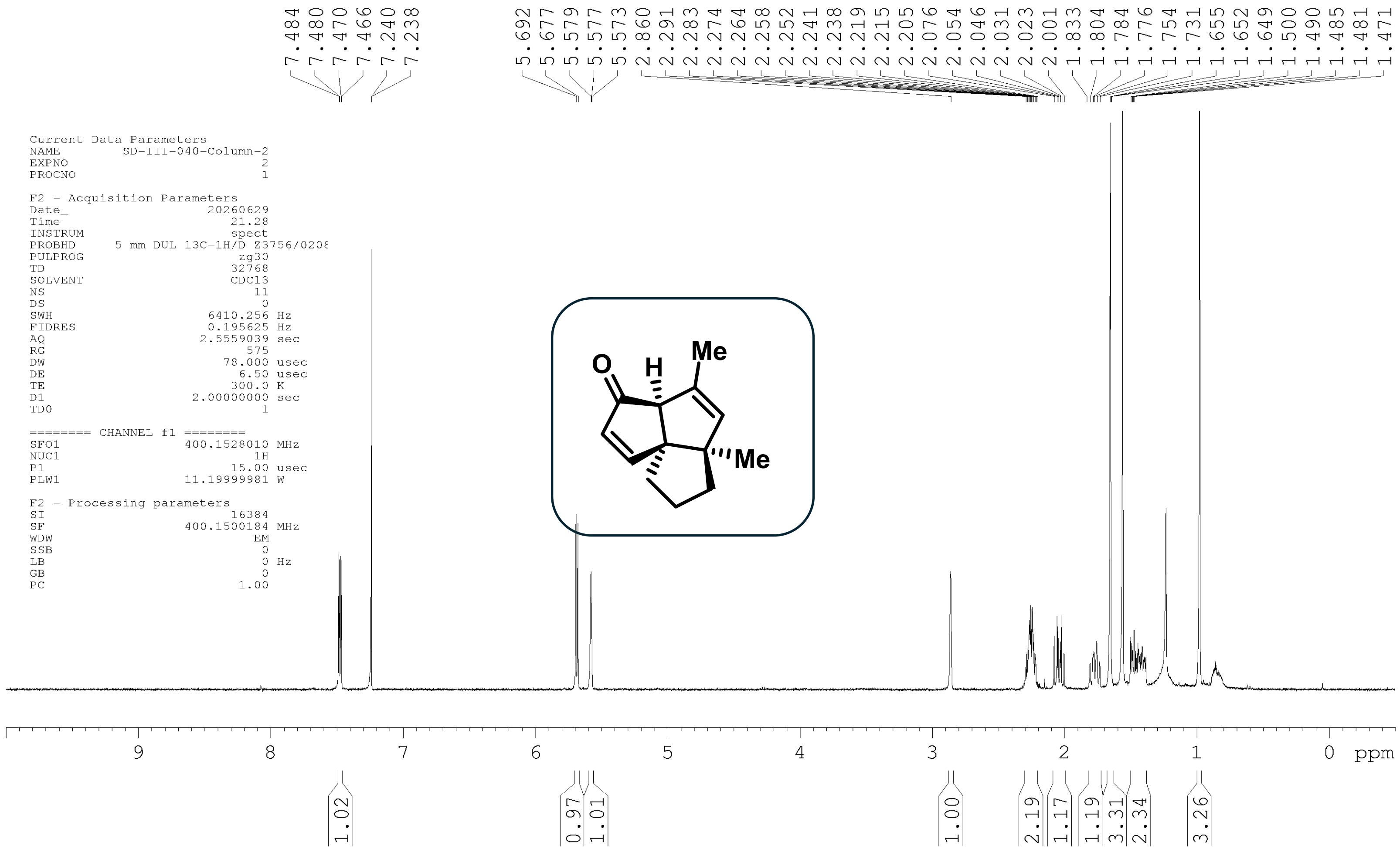

Figure 218. ^{1}H NMR (400 MHz, $CDCl_3$) (**SI-10**)

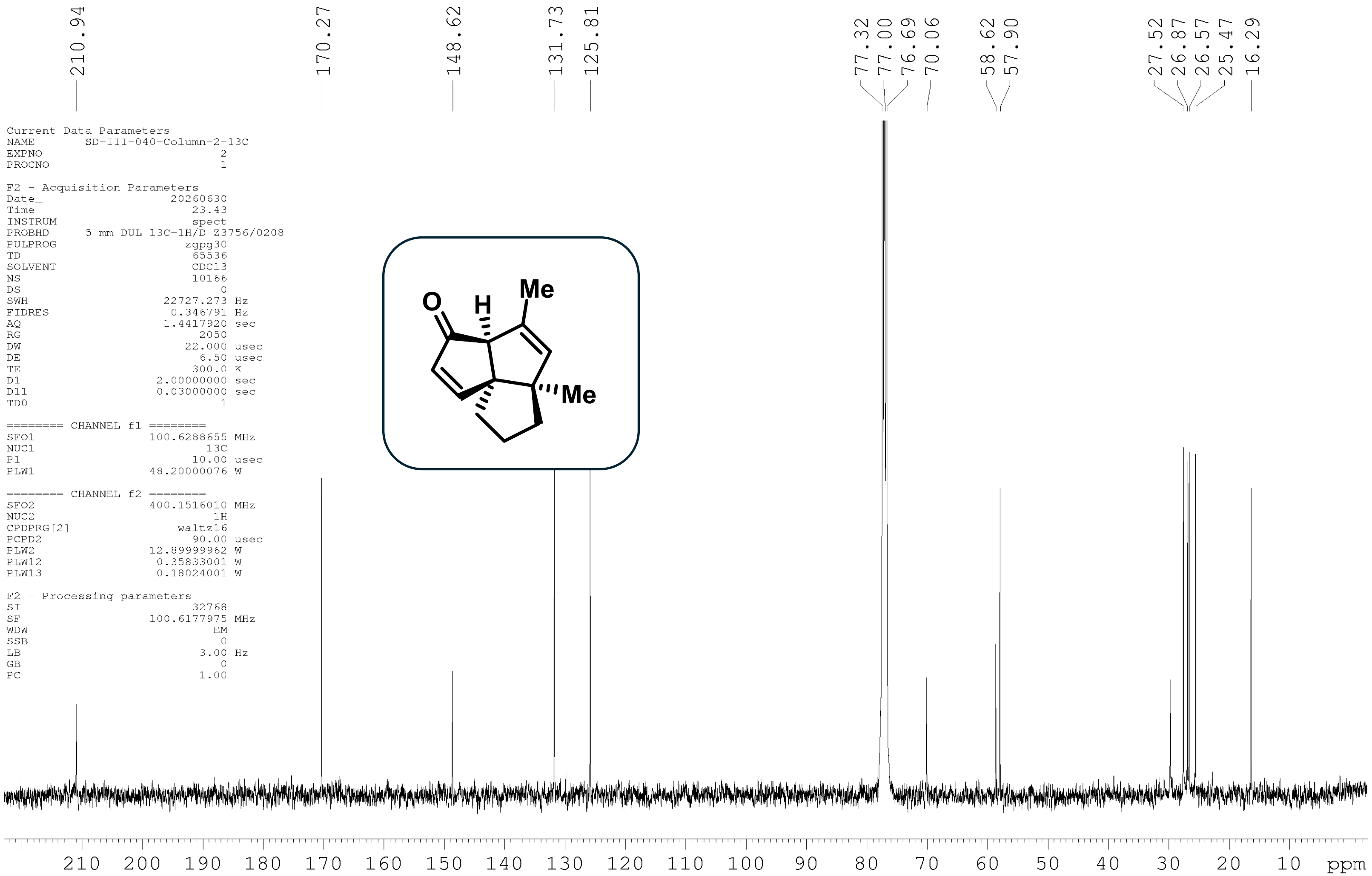

Figure 219. ^{13}C NMR (100 MHz, $CDCl_3$) (**SI-10**)

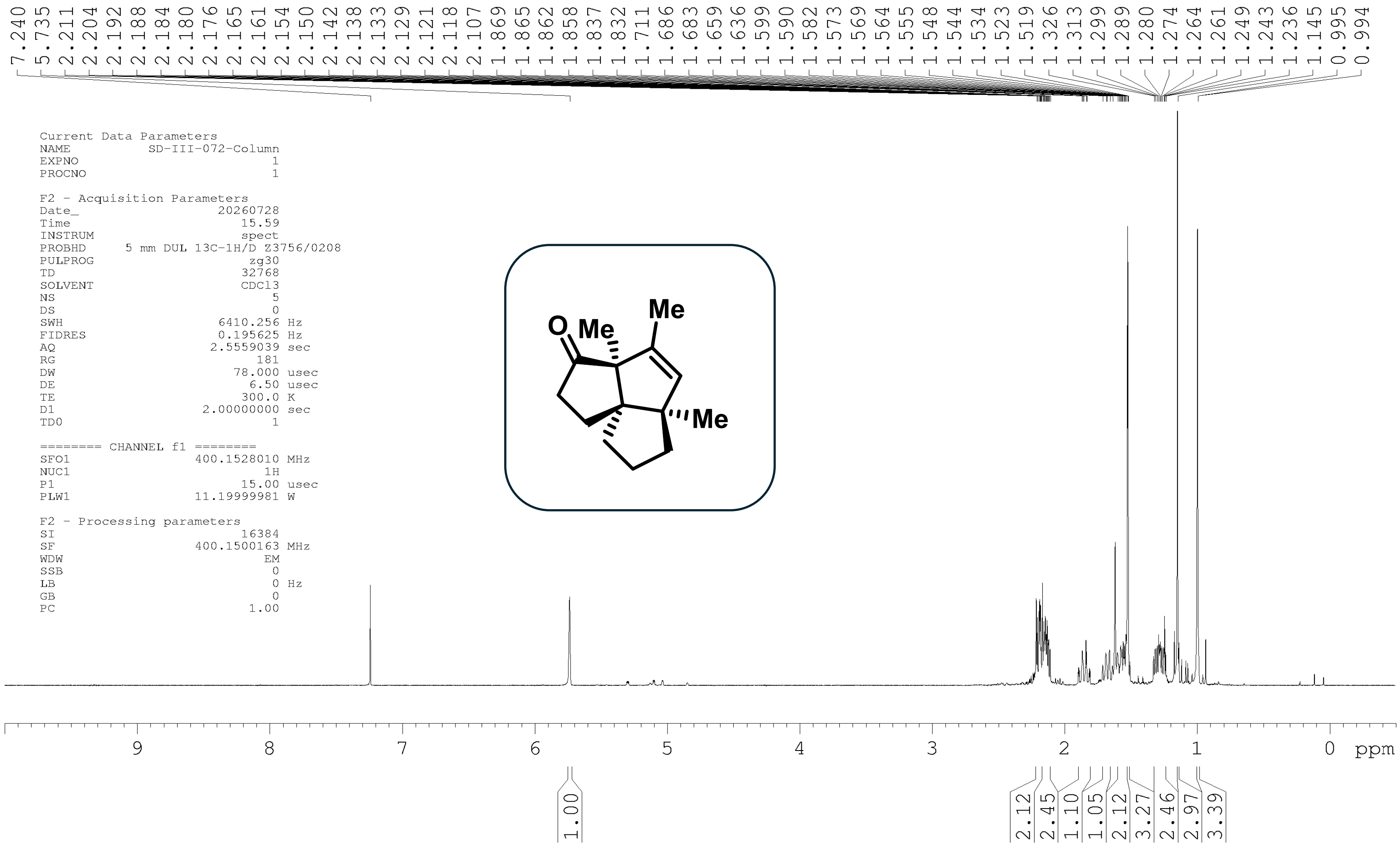

Figure 220. ^{1}H NMR (400 MHz, $CDCl_3$) (**SI-12**)

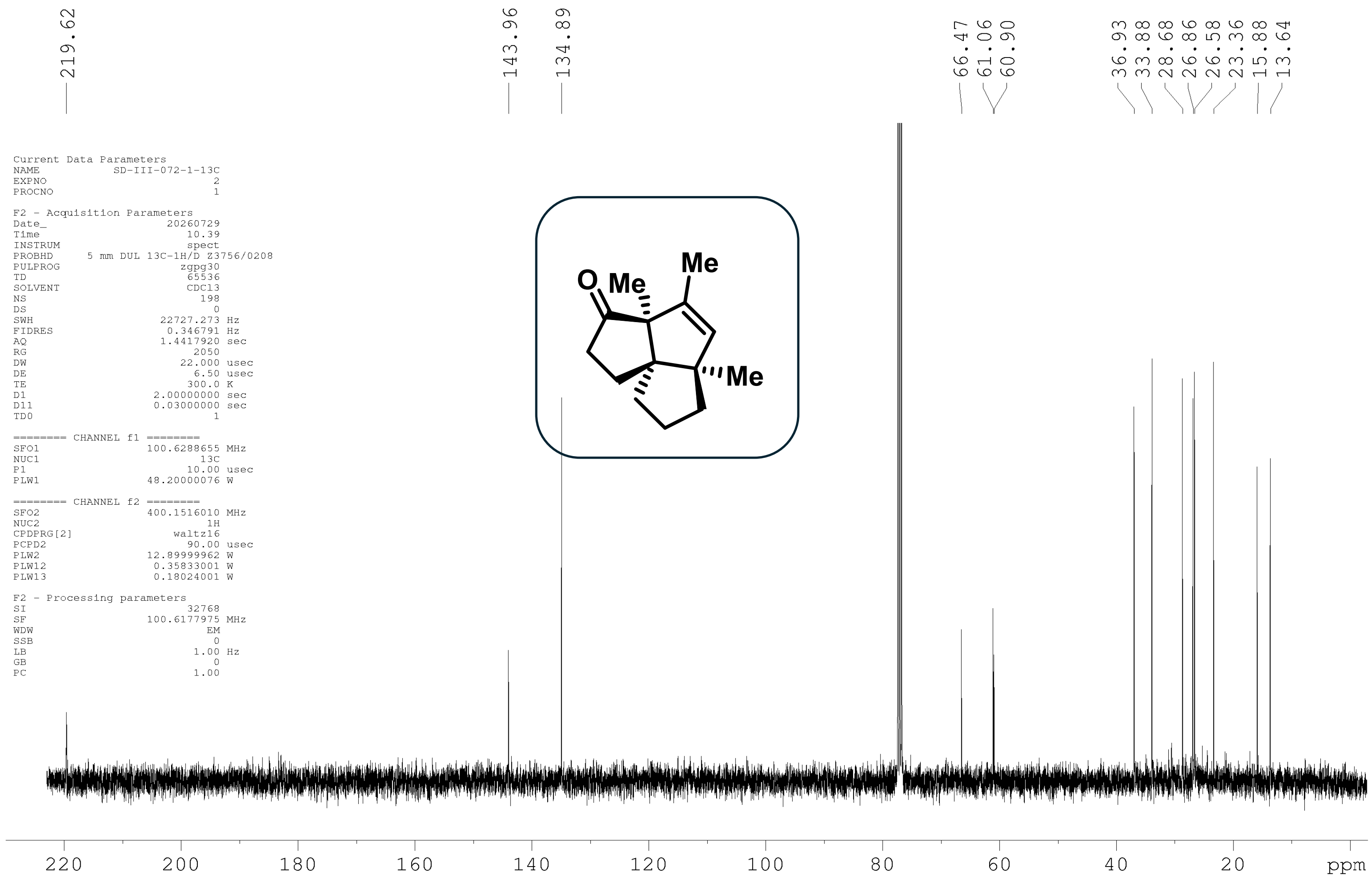

Figure 221. ^{13}C NMR (100 MHz, $CDCl_3$) (**SI-12**)

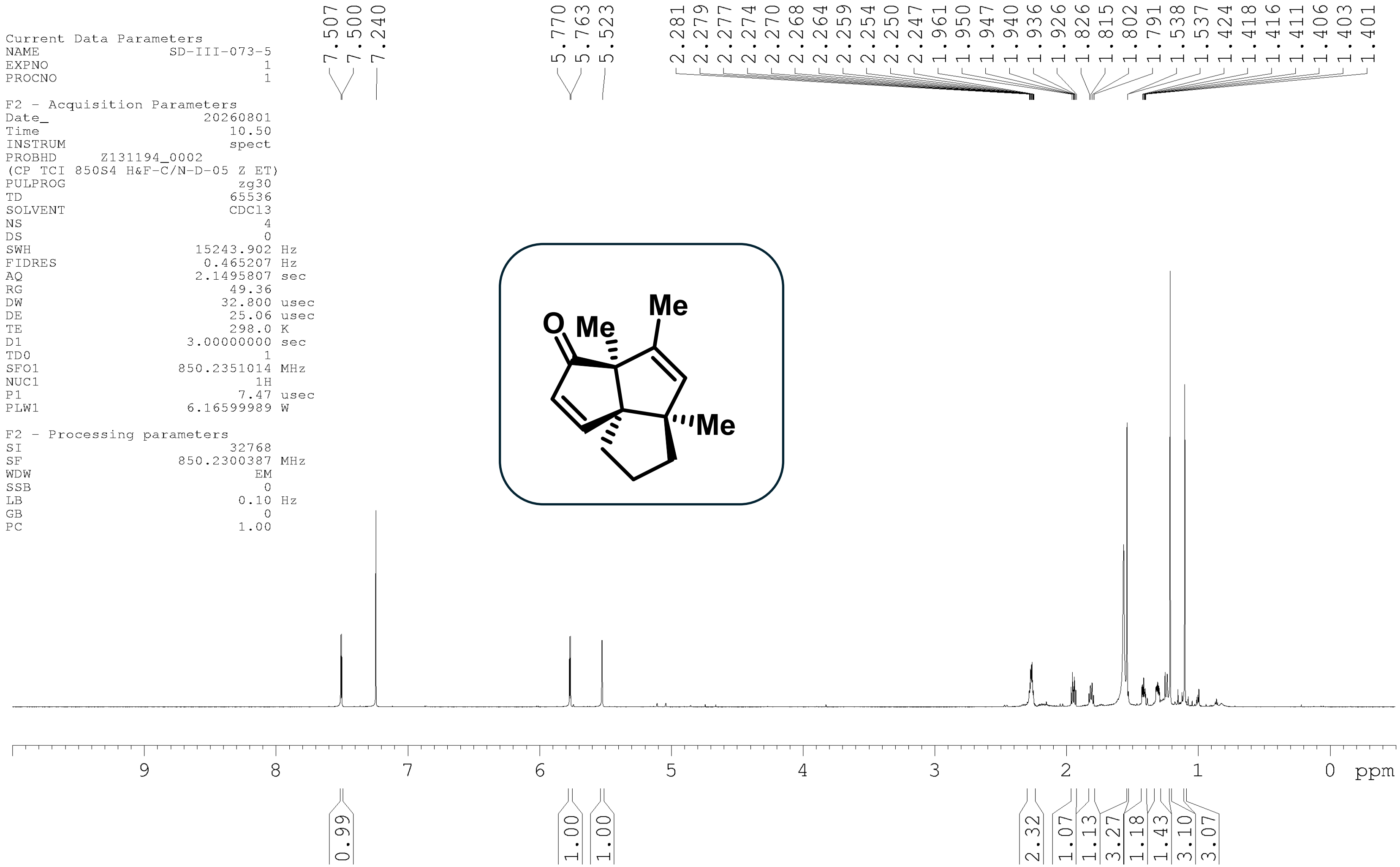

Figure 222. ^{1}H NMR (850 MHz, $CDCl_3$) (**SI-11**)

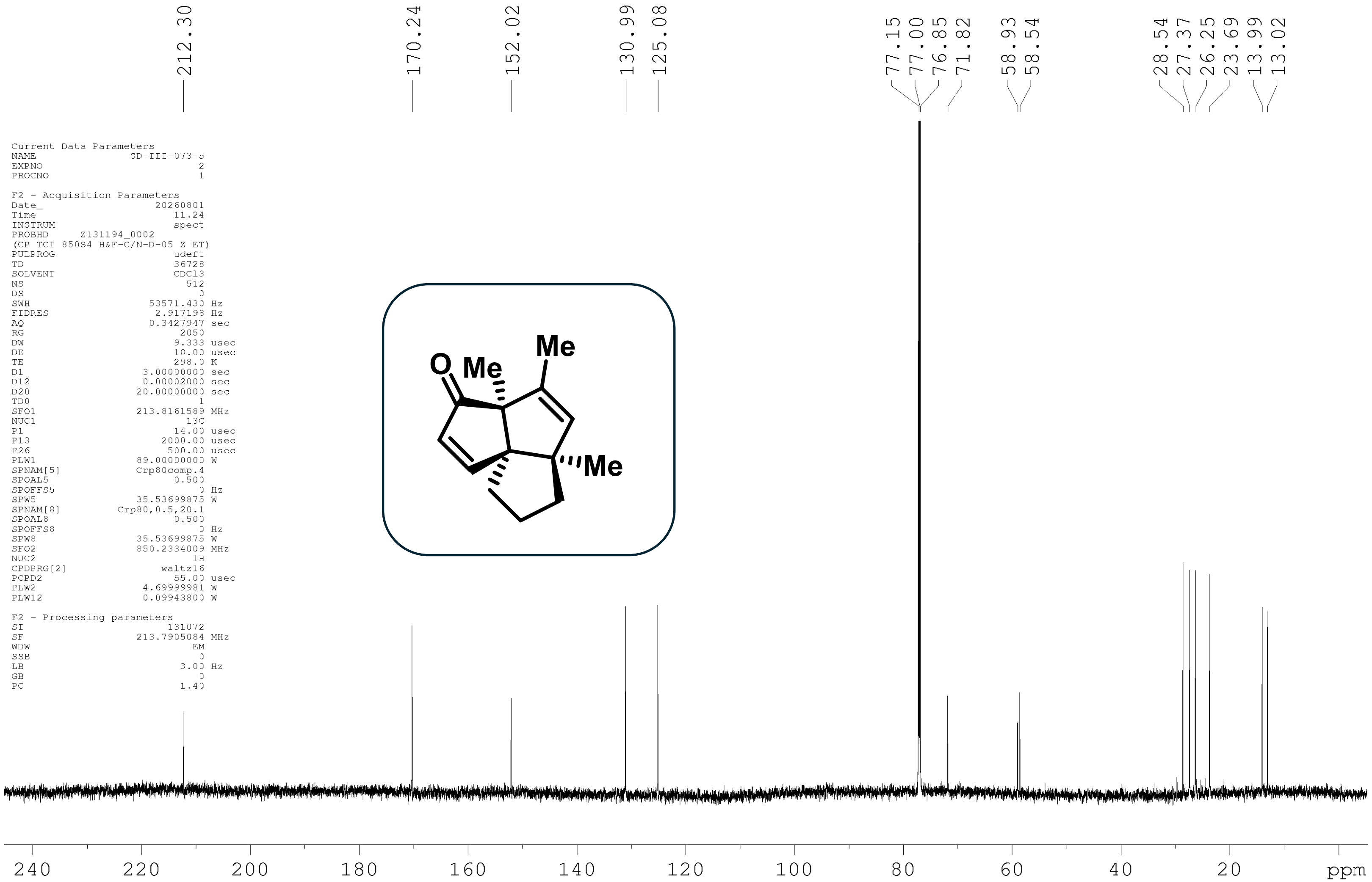

Figure 223. ^{13}C NMR (212.5 MHz, $CDCl_3$) (**SI-11**)
