# Supplementary material for: Diastereodivergent Access to cis,cis- and cis,trans-Angular Triquinanes: Total Synthesis of (±)-Arnicenone
Source: J Am Chem Soc. 2026 Sep 22;148(39):41724–32. doi: 10.1021/jacs.6c09019 (PMC13651531; doi:10.1021/jacs.6c09019)
Supplement: Supplementary file 2 [file ja6c09019_si_007.pdf]

Diastereodivergent Access to *cis*,*cis*- and *cis*,*trans*-angular Triquinanes: Total Synthesis of (±)-Arnicenone

*C.-H. Liu,[‡] H.-T. Huang,[‡] H.-J. Li,[‡†] S. A. Dighe, D.-L. Hou, L.-Y. Wu, and Y.-W. Huang**

Department of Chemistry, National Tsing Hua University, 101,
Section 2, Kuang-Fu Road, Hsinchu 300044, Taiwan R.O.C.
Email: yuwenhuang@mx.nthu.edu.tw

Content

I. General Information 6
II. Tabulated Data for Synthesized Compounds..8
III. NMR Spectra for the Synthesized Compounds...83
IV. X-ray for the Synthesized Compounds 307
28b(*cis,trans*);
28j(*cis,trans*);
28l(*cis,trans*);
28m(*cis,cis*);
28m(*cis,trans*);
30a;
30e;
31α;
42.

List of Figures

Figures 1-2 NMR (**24a**) 84

Figures 3-4 NMR (**24b**) 86

Figures 5-6 NMR (**24c**) 88

Figures 7-8 NMR (**24d**) 90

Figures 9-10 NMR (**25a**) 92

Figures 11-12 NMR (**25b**) 94

Figures 13-14 NMR (**25c**) 96

Figures 15-16 NMR (**25d**) 98

Figures 17-18 NMR (**25e**) 100

Figures 19-20 NMR (**25f**) 102

Figures 21-22 NMR (**25g**) 104

Figures 23-24 NMR (**25h**) 106

Figures 25-26 NMR (**25i**) 108

Figures 27-28 NMR (**25j**) 110

Figures 29-30 NMR (**25k**) 112

Figures 31-32 NMR (**26a**) 114

Figures 33-34 NMR (**26b**) 116

Figures 35-36 NMR (**26c**) 118

Figures 37-38 NMR (**26d**) 120

Figures 39-40 NMR (**26e**) 122

Figures 41-42 NMR (**26f**) 124

Figures 43-44 NMR (**26g**) 126

Figures 45-46 NMR (**26h**) 128

Figures 47-48 NMR (**26i**) 130

Figures 49-50 NMR (**26j**) 132

Figures 51-52 NMR (**26k**) 134
Figures 53-54 NMR (**26l**) 136
Figures 55-56 NMR (**26m**) 138
Figures 57-58 NMR (**26n**) 140
Figures 59-60 NMR (**SI-2**) 142
Figures 61-62 NMR (**SI-3a**) 144
Figures 63-64 NMR (**SI-3b**) 146
Figures 65-66 NMR (**SI-3c**) 148
Figures 67-68 NMR (**SI-3d**) 150
Figures 69-74 NMR (**28m-*cis,trans***) 152
Figures 75-80 NMR (**28m-*cis,cis***) 158
Figures 81-82 NMR (**28a-*cis,trans***) 164
Figures 83-84 NMR (**28a-*cis,cis***) 166
Figures 85-90 NMR (**28b-*cis,trans***) 168
Figures 91-96 NMR (**28b-*cis,cis***) 174
Figures 97-98 NMR (**28c-*cis,trans***) 180
Figures 99-100 NMR (**28c-*cis,cis***) 182
Figures 101-102 NMR (**28d-*cis,trans***) 184
Figures 103-104 NMR (**28d-*cis,cis***) 186
Figures 105-106 NMR (**28e-*cis,trans***) 188
Figures 107-108 NMR (**28e-*cis,cis***) 190
Figures 109-110 NMR (**28f-*cis,trans***) 192
Figures 111-112 NMR (**28g-*cis,trans* & *cis,cis***) 194
Figures 113-114 NMR (**28h-*cis,trans* & *cis,cis***) 196
Figures 115-119 NMR (**28i-*cis,trans***) 198
Figures 120-123 NMR (**28i-*cis,cis***) 203

Figures 124-125 NMR (**28j-*cis,trans***) 207

Figures 126-127 NMR (**28k-*cis,trans***) 209

Figures 128-129 NMR (**28l-*cis,trans***) 211

Figures 130-131 NMR (**29a**) 213

Figures 132-133 NMR (**29b**) 215

Figures 134-135 NMR (**29c**) 217

Figures 136-137 NMR (**29d**) 219

Figures 138-139 NMR (**30a**) 221

Figures 140-141 NMR (**30b**) 223

Figures 142-143 NMR (**30c**) 225

Figures 144-145 NMR (**30d**) 227

Figures 146-147 NMR (**30e**) 229

Figures 148-149 NMR (**30f**) 231

Figures 150-151 NMR (**30g**) 233

Figures 152-153 NMR (**30h**) 235

Figures 154-155 NMR (**30i**) 237

Figures 156-157 NMR (**30j**) 239

Figures 158-159 NMR (**30k**) 241

Figures 160-161 NMR (**30l**) 243

Figures 162-163 NMR (**30m**) 245

Figures 164-165 NMR (**30n**) 247

Figures 166-167 NMR (**31β**) 249

Figures 168-169 NMR (**31α**) 251

Figures 170-171 NMR (**33**) 253

Figures 172-173 NMR (**37**) 255

Figures 174-175 NMR (**38**) 257

Figures 176-177 NMR (**41**) …..259
Figures 178-179 NMR (**42**) …..261
Figures 180-181 NMR (**44**) …..263
Figures 182-183 NMR (**35**) …..265
Figures 184-185 NMR (**36**) …..267
Figures 186-191 NMR (**3**) (**arnicenone**) …..269
Figures 192-193 NMR (**SI-3e**) 275
Figures 194-195 NMR (**SI-3f**) 277
Figures 196-197 NMR (**SI-3g**) 279
Figures 198-199 NMR (**SI-3h**) 281
Figures 200-201 NMR (**SI-3i**) 283
Figures 202-203 NMR (**SI-3j**) 285
Figures 204-205 NMR (**SI-3k**) 287
Figures 206-207 NMR (**SI-5**) 289
Figures 208-209 NMR (**SI-6**) 291
Figures 210-212 NMR (**SI-7**) 293
Figures 213-214 NMR (**SI-8**) 295
Figures 215-216 NMR (**39**) 297
Figures 217-218 NMR (**40**) 299
Figures 219-220 NMR (**SI-10**) 301
Figures 223-224 NMR (**SI-12**) 303
Figures 225-226 NMR (**SI-11**) 305

I. General Information:

Unless otherwise stated, all reactions were carried out in oven (120 °C)-dried glassware under nitrogen (N_2) in anhydrous solvents with Schlenk techniques. Anhydrous tetrahydrofuran (THF), dichloromethane (DCM), diethyl ether (Et_2O), toluene (PhMe), and 1,4-dioxane were obtained from a solvent purification system. Triethylamine (NEt_3) was dried over CaH_2 and distilled before use. Grignard reagents were titrated with Iodine (I_2) in a saturated LiCl solution of THF. $CuBr\cdot Me_2S$ was stored in the glove box before use. All other commercially available reagents were used as received. Reactions were monitored by thin layer chromatography (TLC) carried out on silica gel 60-F254 plates from EMD. Column chromatography was performed on SiliaFlash F60 silica (particle size 40-63 μm). ^{1}H and ^{13}C NMR data were acquired on Bruker Ultrashield 400 MHz, Bruker AVANCE 500 NMR, Varian Unity Inova 500 MHz, Varian Unity Inova 700 MHz and Bruker AVANCE 850 NMR. Chemical shifts (δ) are reported in parts per million (ppm) relative to internal residual solvent peaks from the indicated deuterated solvents (^{1}H: $CHCl_3$ 7.24, ^{13}C: $CHCl_3$ 77.00). Coupling constants (*J*) are reported in Hertz (Hz) and rounded to the nearest 0.1 Hz. Multiplicities are defined as s = singlet, d = doublet, t = triplet, q = quartet, m = multiplet, dd = doublet of doublets, dt = doublet of triplets, td = triplet of doublets, qd = quartet of doublets. High-Resolution Mass Spectrometry (HRMS) was collected on Bruker, Impact HD with electro-spray ionization (ESI) and quadrupole/time-of-flight (Q-TOF) from Instrumentation Resource Center, National Yang Ming Chiao Tung University, Taiwan. Single crystal X-ray analyses were collected on Rigaku XtaLAB Synergy DW from the Instrumentation Center, National Tsing Hua University, Taiwan. The heating system was performed with an aluminum block on Heidolph.

II. Experimental Section:

General procedure for allene **26**(**a-n**) syntheses

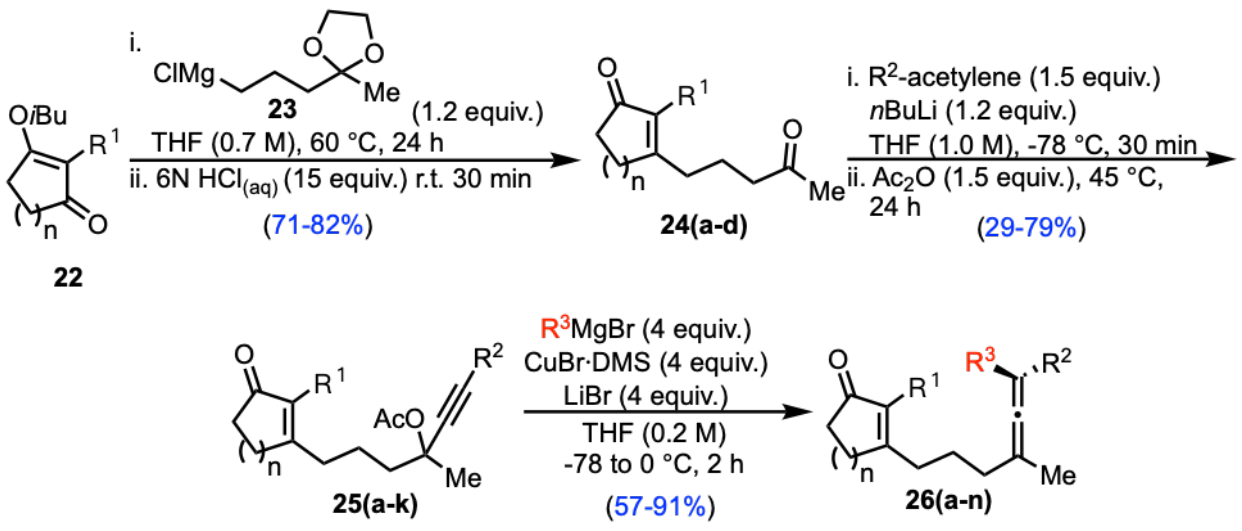

General procedure for **24**(**a-d**) syntheses

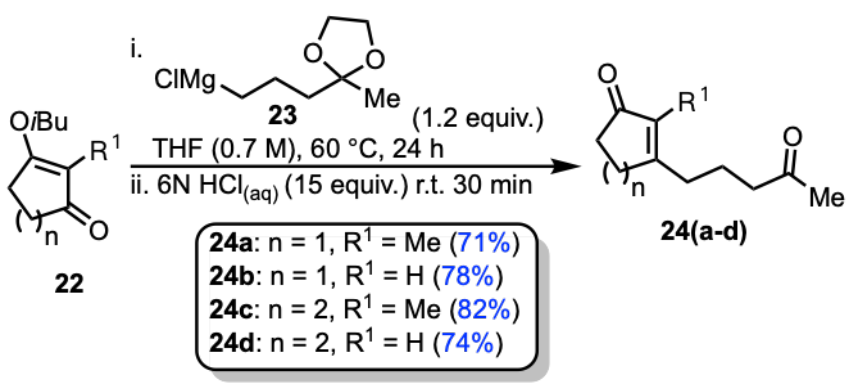

To vinylogous ester **22** (15.12 g, 0.098 mol) dissolved in THF (34 mL, 0.7 M) in a 1 L two-necked flask was dropwisely added the corresponding Grignard reagent **23** (1.2 equiv. in 73 mL THF) at 0 °C under a nitrogen atmosphere. The reaction mixture was then heated to 60 °C and stirred for 24 h. After complete consumption of vinylogous ester **22** monitored by TLC, the septum was removed, and ice-cold 6N aqueous HCl (245 mL, 1.47 mol) was added dropwise at 0 °C to remove the protecting group. After stirring for 30 min, ice-cold 6N aqueous NaOH (220 mL, 1.28 mol) was slowly added to quench the reaction. The mixture was extracted with EtOAc (500 mL) and brine (500 mL) three times. The combined organic layers were dried over $MgSO_4$, filtered, and concentrated under reduced pressure to afford the crude product. Purification by flash column chromatography (EtOAc/hexanes = 1:1) afforded diketone **24b** (12.78 g, 78%, HTH-II-98).

General procedure for **25**(**a-k**) syntheses

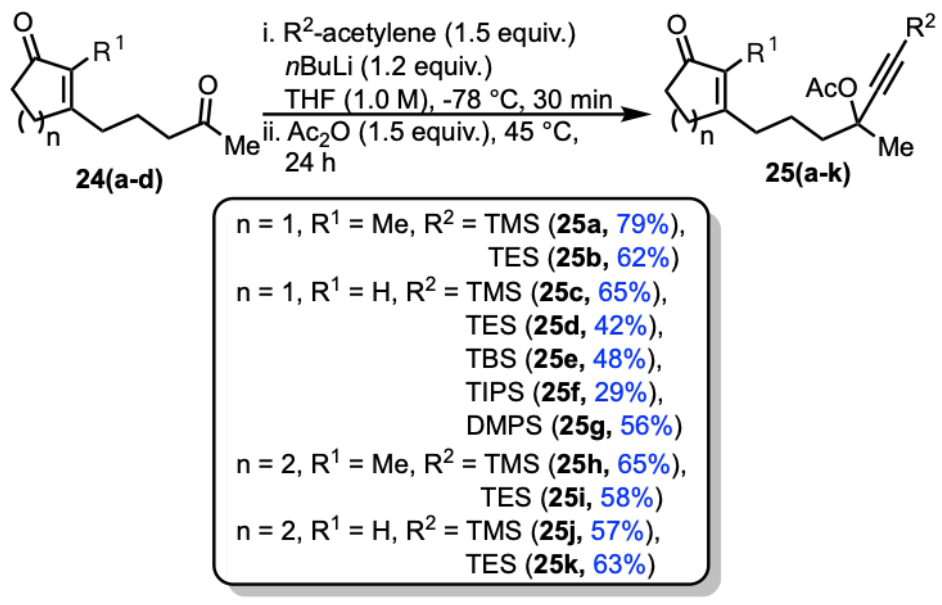

Under a nitrogen atmosphere, the corresponding silyl acetylene (1.67 mL, 11.731 mmol) was placed in a 100 mL round-bottom flask and dissolved in THF (41.2 mL, 0.2 M). *n*-Butyllithium (5.9 mL, 11.08 mmol) was added dropwise at 0 °C, and the reaction mixture was stirred for 30 min at this temperature. The mixture was then cooled to −78 °C, and a solution of **24b** (1.5 g, 9.024 mmol) in THF (5 mL) was added in one portion. After complete consumption of diketone **24b** monitored by TLC, acetic anhydride (1.28 mL, 13.536 mmol) was added, and the reaction mixture was heated to 45 °C and stirred for 24 h. The reaction was quenched with saturated aqueous $NaHCO_3$, and the mixture was extracted with EtOAc (100 mL) and brine (100 mL) three times. The combined organic layers were dried over $MgSO_4$, filtered, and concentrated under reduced pressure to afford the crude product. Purification by flash column chromatography (EtOAc/hexanes = 1:3) afforded the corresponding propargylic acetate **25c** (1.8 g, 65%, HTH-II-136).

General procedure for **26**(**a-n**) syntheses

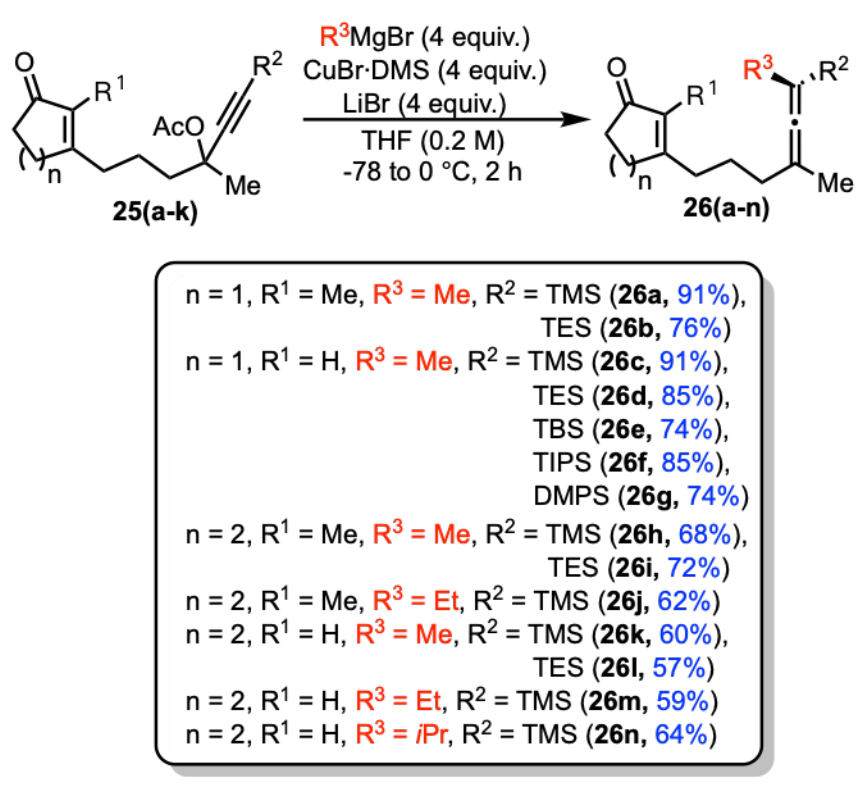

To CuBr·DMS (2.074 g, 10.089 mmol.) and LiBr (0.876 g, 10.089 mmol) were added THF (10 mL, 0.2 M) in a 250 mL round-bottom flask under a nitrogen atmosphere. The Grignard reagent (3.36 mL, 3M in Et_2O, 10.089 mmol) was added dropwise at 0 °C, and the resulting mixture was stirred for 30 min at this temperature. The reaction mixture was then cooled to −78 °C, and a solution of the corresponding propargylic acetate **25c** (0.773 g) in THF (3 mL) was added in one portion. After complete consumption of the starting material monitored by TLC, the reaction was quenched by slow addition of saturated aqueous NH_4Cl and aqueous NH_3 (20 mL). The mixture was extracted with EtOAc (50 mL) and brine (50 mL) three times, and the combined organic layers were dried over $MgSO_4$, filtered, and concentrated under reduced pressure. The crude product was purified by flash column chromatography (EtOAc/hexanes = 1:6) to afford the corresponding allene **26c** (0.575 g, 91%, HTH-II-195).

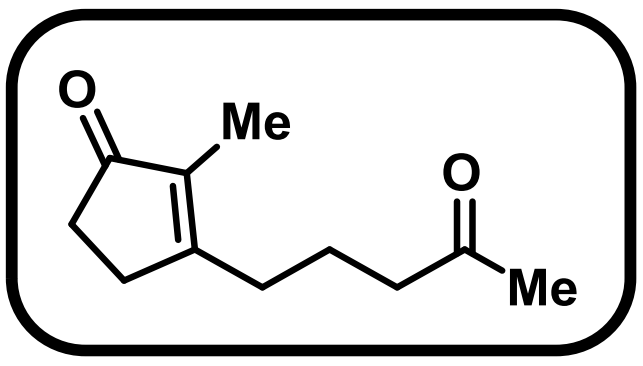

3-(4-oxopentyl)cyclopent-2-en-1-one (24a)

^{1}H NMR (400 MHz, $CDCl_3$): δ 2.48-2.34 (m, 8H), 2.13 (s, 3H), 1.83-1.75 (m, 2H), 1.66 (t, J = 2.2 Hz, 3H).

^{13}C NMR (100 MHz, $CDCl_3$): δ 209.8, 207.7, 172.3, 136.3, 42.5, 33.9, 30.1, 29.8, 28.8, 20.8, 7.8.

HRMS (ESI$^+$): calculated for $C_{11}H_{17}O_2$ $[M+H]^+$ 181.1223, found: 181.1217.

Physical Appearance: yellow oil.

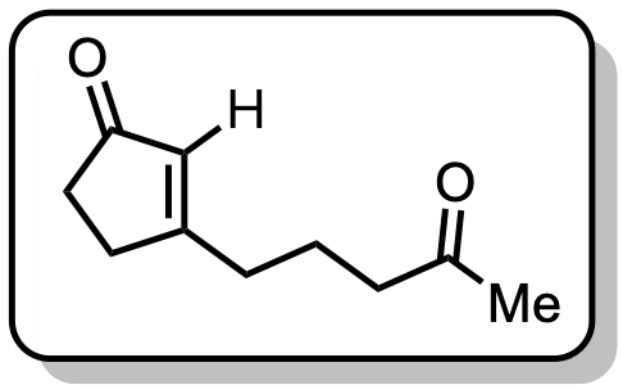

3-(4-oxopentyl)cyclopent-2-en-1-one (24b)

^{1}H NMR (400 MHz, $CDCl_3$): δ 5.94-5.92 (m, 1H), 2.57-2.54 (m, 2H), 2.48 (t, J = 7.1 Hz, 2H), 2.42-2.37 (m, 4H), 2.13 (s, 3H), 1.89-1.82 (m, 2H).

^{13}C NMR (100 MHz, $CDCl_3$): δ 209.6, 207.6, 181.8, 129.3, 42.3, 35.0, 32.3, 31.1, 29.7, 20.5.

HRMS (ESI$^+$): calculated for $C_{10}H_{15}O_2$ $[M+H]^+$ 167.1067, found: 167.1063.

Physical Appearance: brown oil.

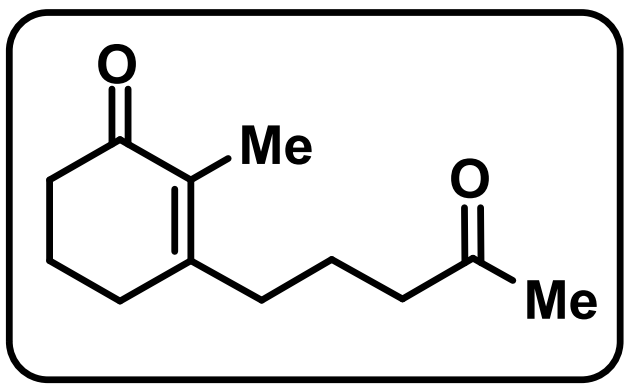

2-methyl-3-(4-oxopentyl)cyclohex-2-en-1-one (24c)

^{1}H NMR: (400 MHz, $CDCl_3$): 2.44 (t, J = 7.2 Hz, 2H), 2.35 (t, J = 6.5 Hz, 2H), 2.32-2.27 (m, 2H), 2.21 (t, J = 7.7 Hz, 2H), 2.12-2.10 (m, 3H), 1.93-1.85 (m, 2H), 1.75-1.68 (m, 5H).

^{13}C NMR (100 MHz, $CDCl_3$): δ 208.0, 199.4, 157.8, 131.2, 42.8, 37.6, 34.3, 30.5, 29.9, 22.4, 21.1, 10.6.

HRMS (ESI+): calculated for $C_{12}H_{19}O_2$ $[M+H]^+$ 195.1380, found: 195.1378.

Physical Appearance: yellow liquid.

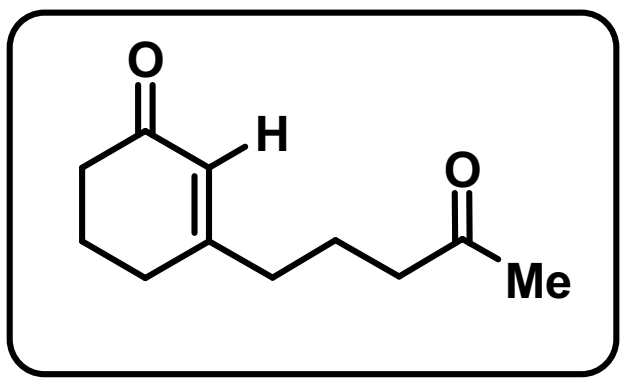

3-(4-oxopentyl)cyclohex-2-en-1-one (24d)

^{1}H NMR: (400 MHz, $CDCl_3$): δ 5.81 (s, 1H), 2.42 (t, J = 7.1 Hz, 2H), 2.31 (t, J = 6.4 Hz, 2H), 2.24 (t, J = 5.9 Hz, 2H), 2.17 (t, J = 7.4 Hz, 2H), 2.10 (s, 3H), 2.00-1.91 (m, 2H), 1.81-1.71 (m, 2H).

^{13}C NMR (100 MHz, $CDCl_3$): δ 207.7, 199.4, 165.3, 125.6, 42.3, 37.0, 36.9, 29.7, 29.2, 22.4, 20.4.

HRMS (ESI+): calculated for $C_{11}H_{17}O_2$ $[M+H]^+$ 181.1223, found:181.1227.

Physical Appearance: yellow liquid.

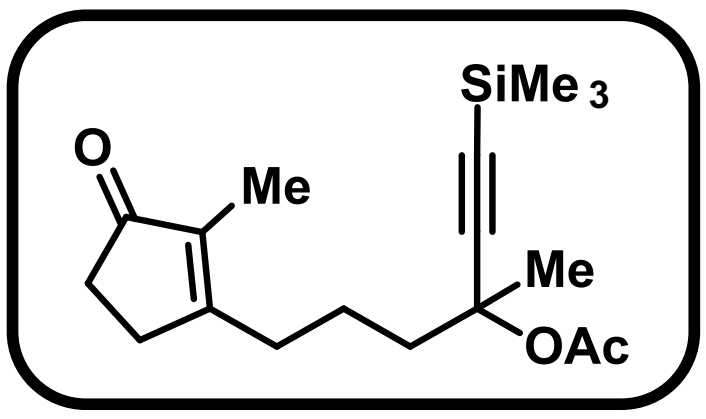

3-methyl-6-(2-methyl-3-oxocyclopent-1-en-1-yl)-1-(trimethylsilyl)hex-1-yn-3-yl acetate (25a)

^{1}H NMR (400 MHz, $CDCl_3$): δ 2.50-2.48 (m, 2H), 2.44 (t, J = 7.4 Hz, 2H), 2.38-2.36 (m, 2H), 2.00 (s, 3H), 1.98-1.92 (m, 1H), 1.76-1.70 (m, 3H), 1.68 (t, J = 1.7 Hz, 3H), 1.64 (s, 3H), 0.14 (s, 9H).

^{13}C NMR (100 MHz, $CDCl_3$): δ 209.7, 172.6, 168.7, 136.3, 104.8, 89.5, 75.0, 40.7, 33.8, 30.5, 28.8, 26.3, 21.7, 21.6, 7.8, -0.4.

HRMS (ESI^+): calculated for $C_{18}H_{28}NaO_3Si$ $[M+Na]^+$ 343.1700, found: 343.1691.

Physical Appearance: pale yellow oil.

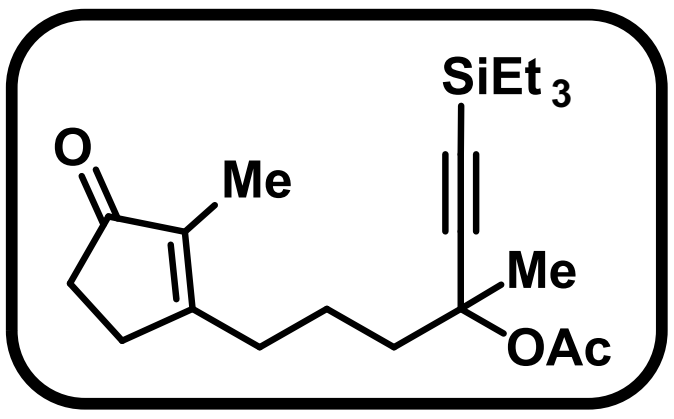

3-methyl-6-(2-methyl-3-oxocyclopent-1-en-1-yl)-1-(triethylsilyl)hex-1-yn-3-yl acetate (25b)

^{1}H NMR (400 MHz, $CDCl_3$): δ 2.49-2.47 (m, 2H), 2.43 (t, J = 7.2 Hz, 2H), 2.36-2.34 (m, 2H), 1.98 (s, 3H), 1.96-1.92 (m, 1H), 1.77-1.68 (m, 3H), 1.67 (t, J = 1.8 Hz, 3H), 1.64 (s, 3H), 0.94 (t, J = 7.8 Hz, 9H), 0.55 (q, J = 7.8 Hz, 6H).

^{13}C NMR (100 MHz, $CDCl_3$): δ 209.9, 172.7, 168.7, 136.4, 106.2, 87.0, 75.1, 41.0, 33.9, 30.7, 28.9, 26.5, 22.0, 21.7, 7.9, 7.3, 4.1.

HRMS (ESI^+): calculated for $C_{21}H_{34}NaO_3Si$ $[M+Na]^+$ 385.2169, found: 385.2155.

Physical Appearance: pale yellow oil.

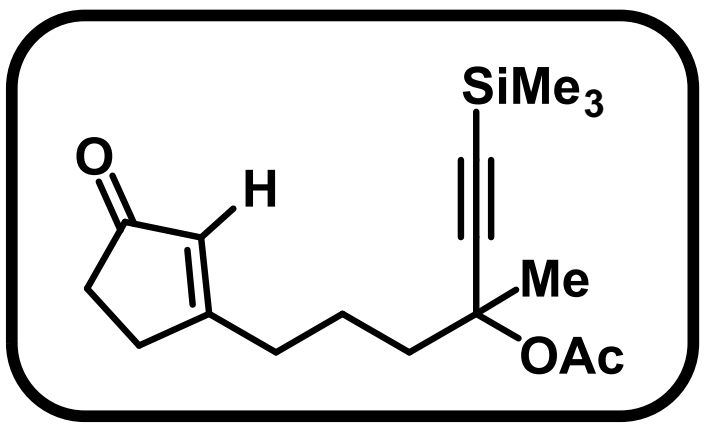

3-methyl-6-(3-oxocyclopent-1-en-1-yl)-1-(trimethylsilyl)hex-1-yn-3-yl acetate (25c)

^{1}H NMR (400 MHz, $CDCl_3$): δ 5.97-5.96 (m, 1H), 2.58-2.56 (m, 2H), 2.44 (t, J = 7.0 Hz, 2H), 2.41-2.39 (m, 2H), 2.00 (s, 3H), 1.98-1.92 (m, 1H), 1.82-1.72 (m, 3H), 1.65 (s, 3H), 0.14 (s, 9H).

^{13}C NMR (100 MHz, $CDCl_3$): δ 209.9, 182.1, 169.0, 129.7, 105.0, 90.0, 75.2, 40.8, 35.3, 33.1, 31.4, 26.6, 22.0, 21.8, -0.1.

HRMS (ESI^+): calculated for $C_{17}H_{26}NaO_3Si$ $[M+Na]^+$ 329.1543, found: 329.1553.

Physical Appearance: pale yellow oil.

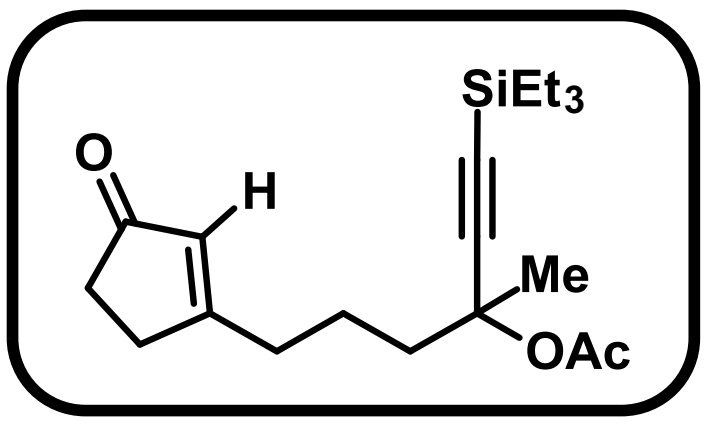

3-methyl-6-(3-oxocyclopent-1-en-1-yl)-1-(triethylsilyl)hex-1-yn-3-yl acetate (25d)

^{1}H NMR (400 MHz, $CDCl_3$): δ 5.96-5.95 (m, 1H), 2.58-2.55 (m, 2H), 2.44 (t, J = 7.2 Hz, 2H), 2.41-2.38 (m, 2H), 1.99 (s, 3H), 1.98-1.95 (m, 1H), 1.84-1.72 (m, 3H), 1.66 (s, 3H), 0.96 (t, J = 7.9 Hz, 9H), 0.57 (q, J = 7.9 Hz, 6H).

^{13}C NMR (100 MHz, $CDCl_3$): δ 209.7, 182.0, 168.7, 129.5, 106.2, 87.2, 75.0, 40.8, 35.1, 33.0, 31.2, 26.6, 21.8, 21.7, 7.3, 4.1.

HRMS (ESI^+): calculated for $C_{20}H_{32}NaO_3Si$ $[M+Na]^+$ 371.2013, found: 371.2003.

Physical Appearance: pale yellow oil.

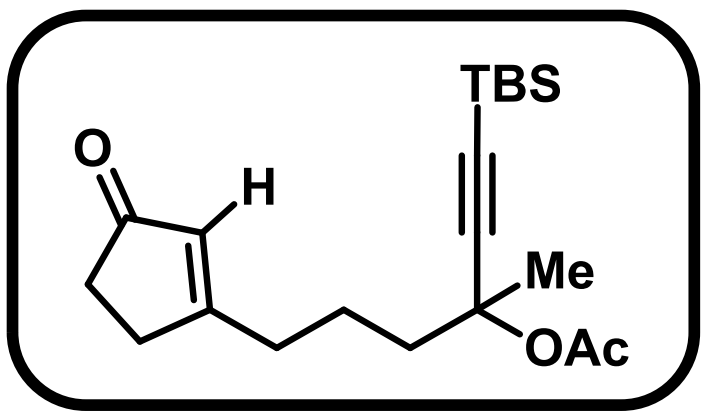

1-(tert-butyldimethylsilyl)-3-methyl-6-(3-oxocyclopent-1-en-1-yl)hex-1-yn-3-yl acetate (25e)

^{1}H NMR (400 MHz, $CDCl_3$): δ 5.96-5.95 (m,1H), 2.58-2.55 (m, 2H), 2.43 (t, J = 7.2 Hz, 2H), 2.40-2.38 (m, 2H), 1.99 (s, 3H), 1.98-1.94 (m, 1H), 1.83-1.71 (m, 3H), 1.65 (s, 3H), 0.90 (s, 9H), 0.07 (s, 6H).

^{13}C NMR (100 MHz, $CDCl_3$): δ 209.8, 182.0, 168.9, 129.7, 105.5, 88.2, 75.1, 40.8, 35.2, 33.1, 31.3, 26.6, 25.9, 21.9 (2*CH_3), 16.5, -4.8.

HRMS (ESI^+): calculated for $C_{20}H_{32}NaO_3Si$ $[M+Na]^+$ 371.2013, found: 371.2006.

Physical Appearance: pale yellow oil.

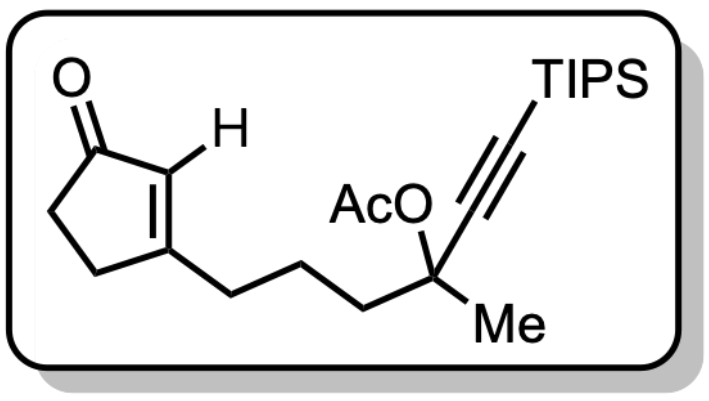

3-methyl-6-(3-oxocyclopent-1-en-1-yl)-1-(triisopropylsilyl)hex-1-yn-3-yl acetate (25f)

^{1}H NMR (400 MHz, $CDCl_3$): δ 5.95-5.94 (m, 1H), 2.58-2.55 (m, 2H), 2.44 (t, J = 7.6 Hz, 2H), 2.41-2.38 (m, 2H), 1.98 (s, 3H), 1.85-1.72 (m, 4H), 1.67 (s, 3H), 1.04 (s, 3H), 1.04 (s, 18H).

^{13}C NMR (100 MHz, $CDCl_3$): δ 209.8, 182.1, 168.8, 129.7, 106.9, 86.2, 75.1, 41.0,35.2, 33.2, 31.3, 26.8, 22.0, 21.8, 18.5, 11.0.

HRMS (ESI$^+$): calculated for $C_{23}H_{38}NaO_3Si$ $[M+Na]^+$ 413.2482, found: 413.2476.

Physical Appearance: pale yellow oil.

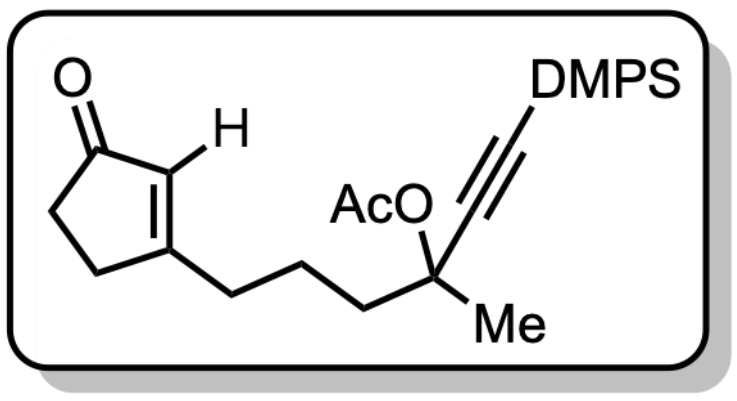

1-(dimethyl(phenyl)silyl)-3-methyl-6-(3-oxocyclopent-1-en-1-yl)hex-1-yn-3-yl acetate (25g)

^{1}H NMR (400 MHz, $CDCl_3$): δ 7.60-7.58 (m, 2H), 7.38-7.32 (m, 3H), 5.95-5.93 (m, 1H), 2.53-2.50 (m, 2H), 2.43 (t, J = 6.9 Hz, 2H), 2.38-2.36 (m, 2H), 2.01 (s, 3H), 2.00-1.97 (m, 1H), 1.81-1.74 (m, 3H), 1.68 (s, 3H), 0.39 (s, 6H).

^{13}C NMR (100 MHz, $CDCl_3$): δ 209.7, 181.9, 168.9, 136.7, 133.5, 129.6, 129.3, 127.7, 106.7, 87.9, 74.9, 40.7, 35.1, 33.0, 31.2, 26.4, 21.8, 21.7, -1.0.

HRMS (ESI+): calculated for $C_{22}H_{28}NaO_3Si$ $[M+Na]^+$ 391.1700, found: 391.1685.

Physical Appearance: pale yellow oil.

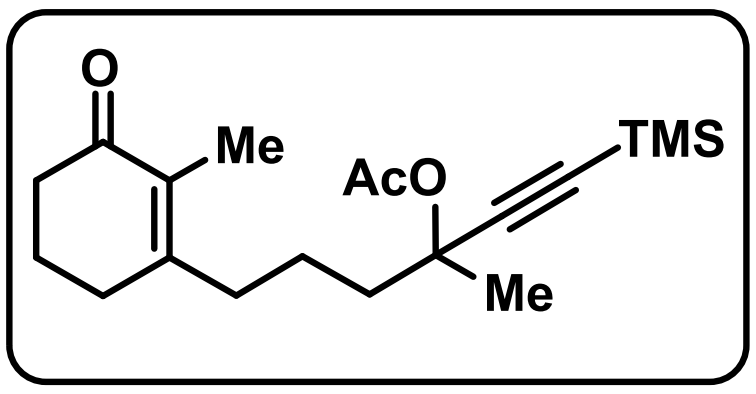

3-methyl-6-(2-methyl-3-oxocyclohex-1-en-1-yl)-1-(trimethylsilyl)hex-1-yn-3-yl acetate (25h)

^{1}H NMR: (400 MHz, $CDCl_3$): δ 2.37 (t, *J* = 6.4 Hz, 2H), 2.32-2.30 (m, 2H), 2.28-2.24 (m, 2H), 1.99-1.98 (m, 3H), 1.97-1.88 (m, 3H), 1.75-1.67 (m, 6H), 1.63 (s, 3H), 0.14-0.13 (m, 9H).

^{13}C NMR (100 MHz, $CDCl_3$): δ 199.4, 169.0, 158.2, 131.3, 105.1, 89.8, 75.4, 41.1, 37.7, 34.9, 30.7, 26.5, 22.5, 22.2, 22.0, 10.7, -0.1.

HRMS (ESI+): calculated for $C_{19}H_{30}NaO_3Si$ $[M+Na]^+$ 357.1856, found: 357.1860.

Physical Appearance: Pale yellow liquid.

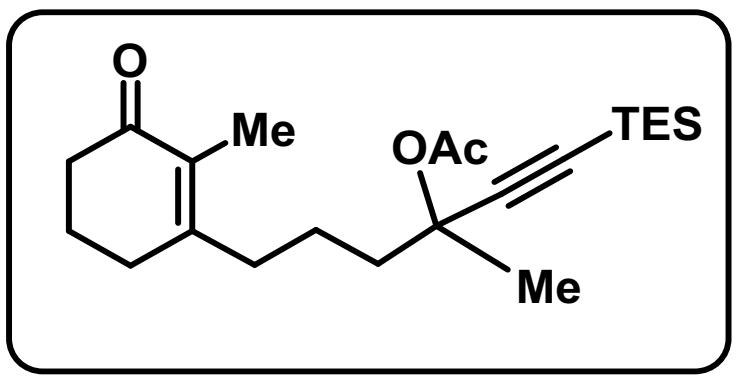

3-methyl-6-(2-methyl-3-oxocyclohex-1-en-1-yl)-1 triethylsilyl)hex-1-yn-3-yl acetate (25i)

^{1}H NMR: (400 MHz, $CDCl_3$): δ 2.37 (t, *J* = 6.2 Hz, 2H), 2.31-2.29 (m, 2H), 2.27 (t, *J* = 7.6, Hz, 2H), 1.98 (s, 3H), 1.96-1.87 (m, 3H), 1.75 (s, 3H), 1.73-1.67 (m, 3H), 1.64 (s, 3H) 0.95 (t, *J* = 7.9 Hz, 9H), 0.56 (q, *J* = 8.0 Hz, 6H).

^{13}C NMR (100 MHz, $CDCl_3$): δ 199.3, 168.8, 158.1, 131.0, 106.3, 87.0, 75.2, 41.1, 37.6, 34.8, 30.6, 26.6, 22.4, 22.2, 21.8, 10.5, 7.3, 4.2.

HRMS (ESI+): calculated for $C_{22}H_{36}NaO_3Si$ $[M+Na]^+$ 399.2320, found: 399.2328.

Physical Appearance: Pale yellow liquid.

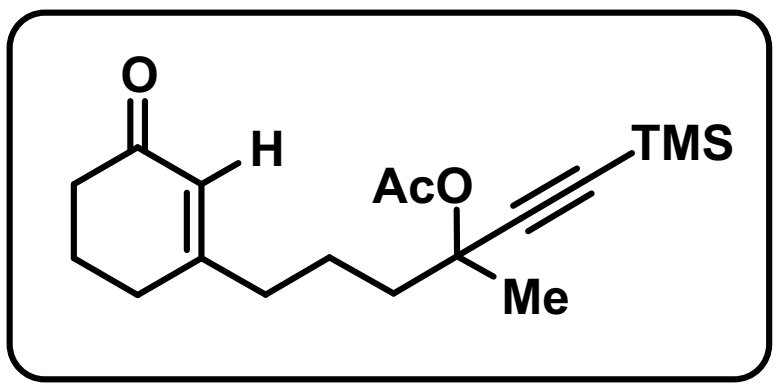

3-methyl-6-(3-oxocyclohex-1-en-1-yl)-1-(trimethylsilyl)hex-1-yn-3-yl acetate (25j)

^{1}H NMR: (400 MHz, $CDCl_3$): δ 5.86 (s, 1H), 2.34 (t, *J* = 6.2 Hz, 2H), 2.27-2.21 (m, 4H), 1.98 (s, 3H), 1.97-1.87 (m, 3H), 1.75-1.64 (m, 3H), 1.62 (s, 3H), 0.13 (s, 9H).

^{13}C NMR (100 MHz, $CDCl_3$): δ 199.7, 169.0, 165.6, 126.0, 105.1, 89.8, 75.2, 40.7, 37.6, 37.3, 29.5, 26.5, 22.7, 21.9, 21.5, -0.2.

HRMS (ESI+): calculated for $C_{18}H_{28}NaO_3Si$ $[M+Na]^+$ 343.1700, found:343.1708.

Physical Appearance: Pale yellow liquid.

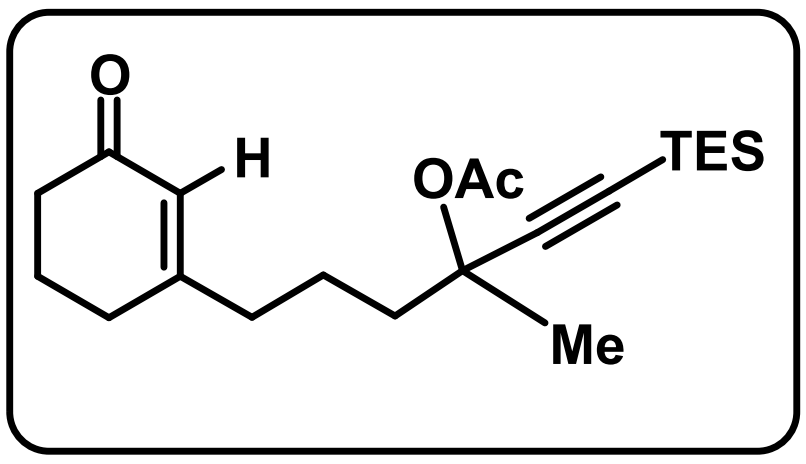

3-methyl-6-(3-oxocyclohex-1-en-1-yl)-1-(triethylsilyl)hex-1-yn-3-yl acetate (25k)

^{1}H NMR: (400 MHz, $CDCl_3$): δ 5.87 (s, 1H), 2.35 (t, *J* = 6.4 Hz, 2H), 2.28-2.22 (m, 4H), 1.99 (s, 3H), 1.97-1.93 (m, 2H), 1.77-1.67 (m, 4H), 1.64 (s, 3H), 0.96 (t, *J* = 7.9 Hz, 9H), 0.57 (q, *J* = 7.8 Hz, 6H).

^{13}C NMR (100 MHz, $CDCl_3$): δ 199.7, 168.8, 165.6, 125.9, 106.3, 87.1, 75.2, 40.8, 37.6, 37.2, 29.4, 26.6, 22.6, 21.8, 21.6, 7.3, 4.2.

HRMS (ESI+): calculated for $C_{21}H_{34}NaO_3Si$ $[M+Na]^+$ 385.2164, found: 385.2169.

Physical Appearance: Pale yellow liquid.

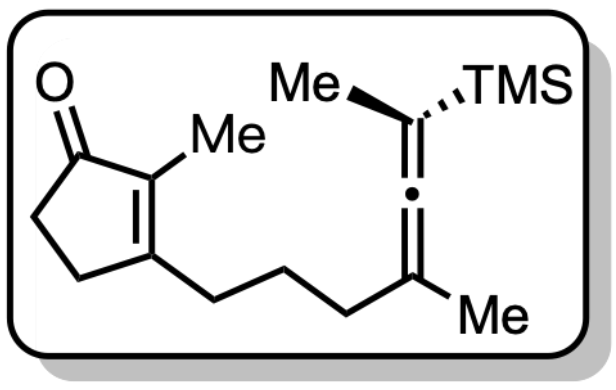

2-methyl-3-(4-methyl-6-(trimethylsilyl)hepta-4,5-dien-1-yl)cyclopent-2-en-1-one (26a)

^{1}H NMR (400 MHz, $CDCl_3$): δ 2.47-2.46 (m, 2H), 2.42 (t, *J* = 7.8 Hz, 2H), 2.36-2.34 (m, 2H), 1.90 (td, *J* = 7.4, 2.2 Hz, 2H), 1.67 (t, *J* = 2 Hz, 3H), 1.64-1.57 (m, 8H), 0.03 (s, 9H).

^{13}C NMR (100 MHz, $CDCl_3$): δ 210.1, 204.0, 173.7, 136.2, 91.7, 91.2, 34.1, 33.3, 30.9, 29.2, 25.3, 18.7, 15.8, 7.9, -1.7.

HRMS (ESI^+): calculated for $C_{17}H_{29}OSi$ $[M+H]^+$ 277.1982, found: 277.1980.

Physical Appearance: pale yellow oil.

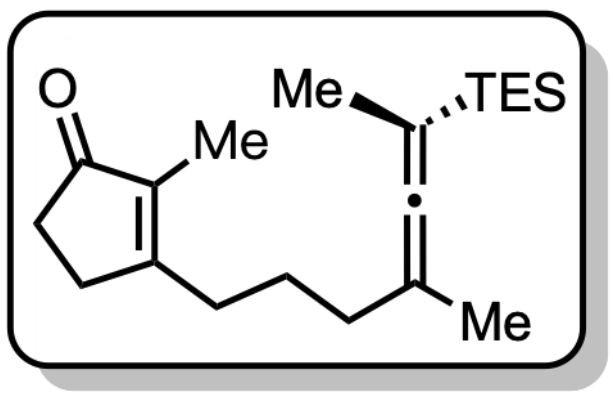

2-methyl-3-(4-methyl-6-(triethylsilyl)hepta-4,5-dien-1-yl)cyclopent-2-en-1-one (26b)

^{1}H NMR (400 MHz, $CDCl_3$): δ 2.47-2.45 (m, 2H), 2.42 (t, *J* = 7.6 Hz, 2H), 2.35-2.33 (m, 2H), 1.96-1.82 (m, 2H), 1.66 (t, *J* = 1.7 Hz, 3H), 1.64-1.56 (m, 2H), 1.61 (s, 3H), 1.61 (s, 3H), 0.90 (t, *J* = 7.8 Hz, 9H), 0.53 (q, *J* = 7.8 Hz, 6H).

^{13}C NMR (100 MHz, $CDCl_3$): δ 209.9, 204.9, 173.6, 136.0, 90.8, 87.7, 33.9, 33.2, 30.7, 29.0, 25.2, 18.6, 16.4, 7.7, 7.1, 2.9.

HRMS (ESI^+): calculated for $C_{20}H_{35}OSi$ $[M+H]^+$ 319.2452, found: 319.2455.

Physical Appearance: pale yellow oil.

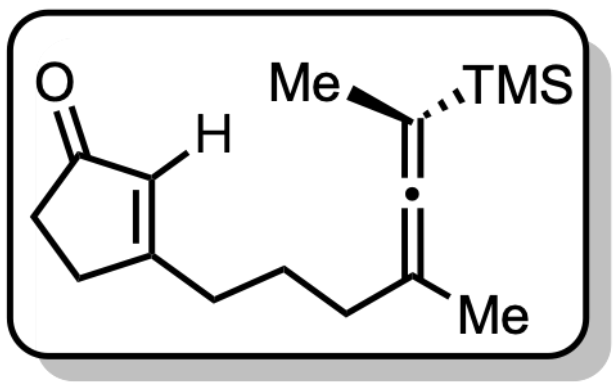

3-(4-methyl-6-(trimethylsilyl)hepta-4,5-dien-1-yl)cyclopent-2-en-1-one (26c)

^{1}H NMR (400 MHz, $CDCl_3$): δ 5.93-5.91 (m, 1H), 2.56-2.53 (m, 2H), 2.43-2.37 (m, 4H), 1.92 (t, J = 7.5 Hz, 2H), 1.66 (q, *J* = 7.5 Hz, 2H), 1.62 (s, 3H), 1.61 (s, 3H), 0.03 (s, 9H).

^{13}C NMR (100 MHz, $CDCl_3$): δ 209.9, 203.9, 182.9, 129.3, 91.6, 91.2, 35.2, 33.0, 33.0, 31.4, 25.1, 18.6, 15.8, -1.8

HRMS (ESI$^+$): calculated for $C_{16}H_{27}OSi$ $[M+H]^+$ 263.1826, found: 263.1831.

Physical Appearance: pale yellow oil.

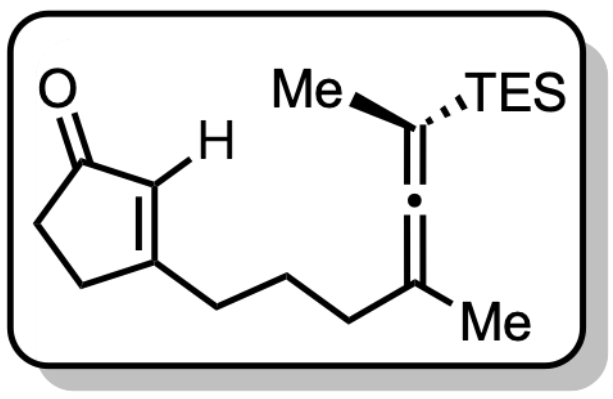

3-(4-methyl-6-(triethylsilyl)hepta-4,5-dien-1-yl)cyclopent-2-en-1-one (26d)

^{1}H NMR (400 MHz, $CDCl_3$): δ 5.93-5.92 (m, 1H), 2.56-2.54 (m, 2H), 2.44-2.37 (m, 4H), 1.99-1.85 (m, 2H), 1.68 (q, *J* = 7.4 Hz, 2H), 1.62 (s, 3H), 1.61 (s, 3H), 0.91 (t, *J* = 7.8 Hz, 9H), 0.54 (q, *J* = 7.8 Hz, 6H).

^{13}C NMR (100 MHz, $CDCl_3$): δ 209.9, 205.0, 182.9, 129.3, 90.8, 87.9, 35.1, 33.1, 33.0, 31.4, 25.1, 18.7, 16.5, 7.2, 2.9.

HRMS (ESI+): calculated for $C_{19}H_{33}OSi$ $[M+H]^+$ 305.2295, found: 305.2294.

Physical Appearance: pale yellow oil.

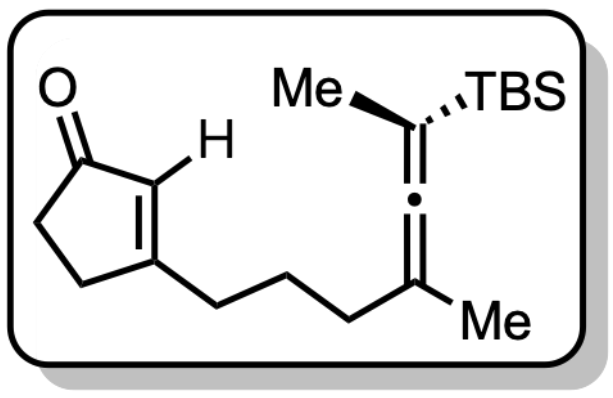

3-(6-(tert-butyldimethylsilyl)-4-methylhepta-4,5-dien-1-yl)cyclopent-2-en-1-one (26e)

^{1}H NMR (400 MHz, $CDCl_3$): δ 5.93-5.91 (m, 1H), 2.56-2.54 (m, 2H), 2.43-2.37 (m, 4H), 2.02-1.85 (m, 2H), 1.70-1.62 (m, 2H), 1.65 (s, 3H), 1.61 (s, 3H), 0.87 (s, 9H), -0.01 (s, 3H), -0.02 (s, 3H).

^{13}C NMR (100 MHz, $CDCl_3$): δ 209.9, 205.5, 182.9, 129.4, 90.9, 88.8, 35.2, 33.2, 33.0, 31.4, 26.6, 25.2, 18.5, 17.5, 17.3, -5.9, -6.2.

HRMS (ESI^+): calculated for $C_{19}H_{33}OSi$ $[M+H]^+$ 305.2295, found: 305.2301.

Physical Appearance: pale yellow oil.

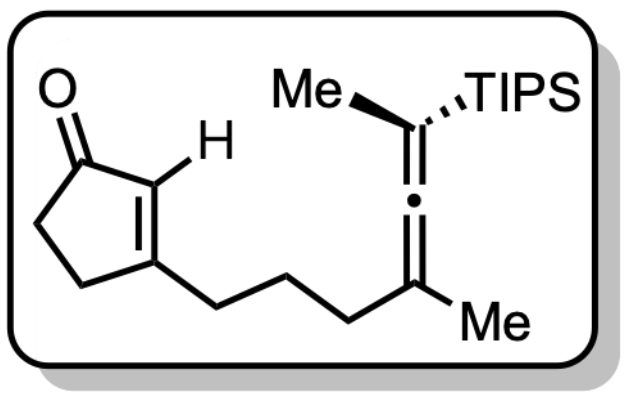

3-(6-(tert-butyldimethylsilyl)-4-methylhepta-4,5-dien-1-yl)cyclopent-2-en-1-one (26f)

^{1}H NMR (400 MHz, $CDCl_3$): δ 5.93-5.91 (m, 1H), 2.57-2.54 (m, 2H), 2.42 (t, *J* = 7.6 Hz, 2H), 2.39-2.37 (m, 2H), 2.04-1.84 (m, 2H), 1.70 (q, *J* = 7.6 Hz, 2H), 1.66 (s, 3H), 1.61 (s, 3H), 1.04 (s, 18H), 1.03 (s, 3H).

^{13}C NMR (100 MHz, $CDCl_3$): δ 209.7, 206.2, 182.7, 129.2, 90.1, 86.1, 35.1, 33.2, 32.9, 31.3, 25.1, 18.5, 18.9, 11.1.

HRMS (ESI+): calculated for $C_{22}H_{39}OSi$ $[M+H]^+$ 347.2765, found: 347.2766.

Physical Appearance: pale yellow oil.

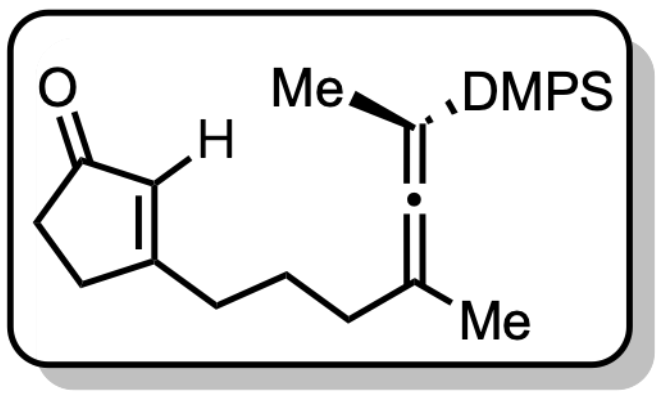

3-(6-(dimethyl(phenyl)silyl)-4-methylhepta-4,5-dien-1-yl)cyclopent-2-en-1-one (26g)

^{1}H NMR (400 MHz, $CDCl_3$): δ 7.50-7.47 (m, 2H), 7.35-7.29 (m, 3H), 5.90-5.89 (m, 1H), 2.50-2.48 (m, 2H), 2.38-2.34 (m, 4H), 1.91 (t, J = 7.9 Hz, 2H), 1.64-1.57 (m, 2H), 1.63 (s, 6H, 2*CH3), 0.31 (s, 3H), 0.30 (s, 3H).

^{13}C NMR (100 MHz, $CDCl_3$): δ 209.7, 205.3, 182.8, 138.1, 133.5, 129.2, 128.8, 127.5, 92.1, 89.8, 35.1, 32.9, 31.3, 25.0, 18.5, 16.1, -3.2, -3.2.

HRMS (ESI$^+$): calculated for $C_{21}H_{29}OSi$ $[M+H]^+$ 325.1982, found: 325.1978.

Physical Appearance: pale yellow oil.

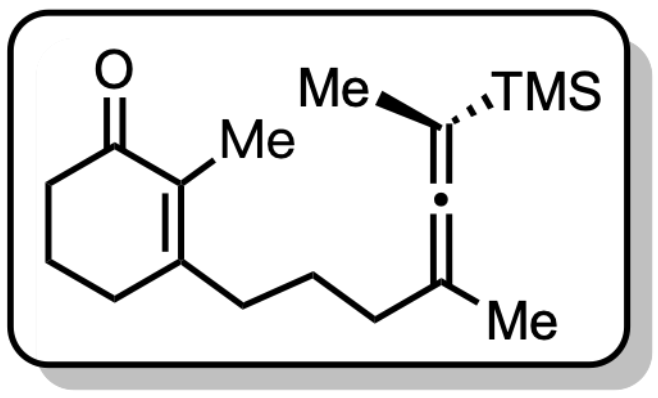

2-methyl-3-(4-methyl-6-(trimethylsilyl)hepta-4,5-dien-1-yl)cyclohex-2-en-1-one (26h)

^{1}H NMR: (400 MHz, $CDCl_3$): δ 2.36 (t, J = 6.6 Hz, 2H), 2.29 (t, 5.8 Hz, 2H), 2.24 (t, J = 7.8 Hz, 2H), 1.93-1.87 (m, 4H), 1.74 (s, 3H), 1.63-1.62 (m, 3H), 1.61 (s, 3H), 1.55-1.48 (m, 2H), 0.0 (s, 9H).

^{13}C NMR (100 MHz, $CDCl_3$): δ 204.1, 199.5, 159.1, 130.9, 91.9, 91.1, 37.8, 35.0, 33.6, 30.8, 25.5, 22.5, 18.7, 16.0, 10.6, -1.7.

HRMS (ESI+): calculated for $C_{18}H_{31}OSi$ $[M+H]^+$ 291.2139, found: 291.2144.

Physical Appearance: colorless oil.

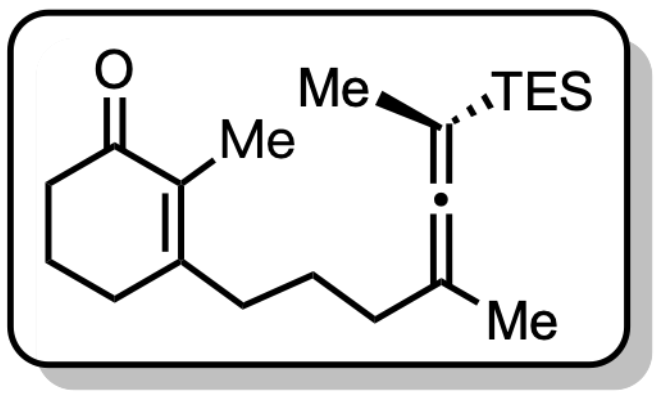

2-methyl-3-(4-methyl-6-(triethylsilyl)hepta-4,5-dien-1-yl)cyclohex-2-en-1-one (26i)

^{1}H NMR: (400 MHz, $CDCl_3$): δ 2.36 (t, *J* = 6.5 Hz, 2H), 2.30 (t, *J* = 5.2 Hz, 2H), 2.24 (t, *J* = 7.7 Hz, 2H), 1.95-1.83 (m, 4H), 1.74 (s, 3H), 1.61-1.60 (m, 6H), 1.58-1.50 (m, 2H), 0.90 (t, *J* = 7.8 Hz, 9H), 0.56-0.50 (m, 6H).

^{13}C NMR (100 MHz, $CDCl_3$): δ 205.1, 199.5, 159.1, 130.9, 91.1, 87.8, 37.7, 34.9, 33.7, 30.8, 25.6, 22.5,18.8, 16.7, 10.6, 7.3, 3.1.

HRMS (ESI+): calculated for $C_{21}H_{37}OSi$ $[M+H]^+$ 333.2608, found: 333.2609.

Physical Appearance: colorless oil.

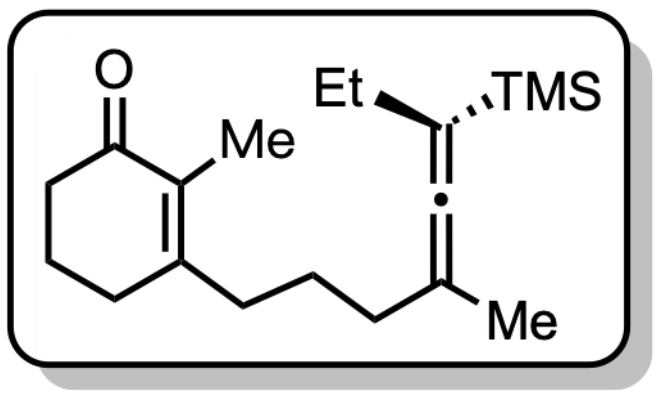

2-methyl-3-(4-methyl-6-(trimethylsilyl)octa-4,5-dien-1-yl)cyclohex-2-en-1-one (26j)

^{1}H NMR: (400 MHz, $CDCl_3$): δ 2.36 (t, *J* = 6.3 Hz, 2H), 2.32-2.28 (m, 2H), 2.24 (t, *J* = 7.8, 2H), 1.95-1.88 (m, 6H), 1.75-1.74 (m, 3H), 1.62 (s, 3H), 1.56-1.48 (m, 2H), 0.97 (t, *J* = 7.4 Hz, 3H), 0.02 (s, 9H).

^{13}C NMR (100 MHz, $CDCl_3$): δ 203.5, 199.5, 159.1, 130.9, 98.4, 94.0, 37.7, 35.1, 33.7, 30.8, 25.7, 22.8, 22.5, 18.7, 14.0, 10.6, -1.3.

HRMS (ESI+): calculated for $C_{19}H_{33}O_{Si}$ $[M+H]^+$ 305.2295, found: 305.2298.

Physical Appearance: colorless oil.

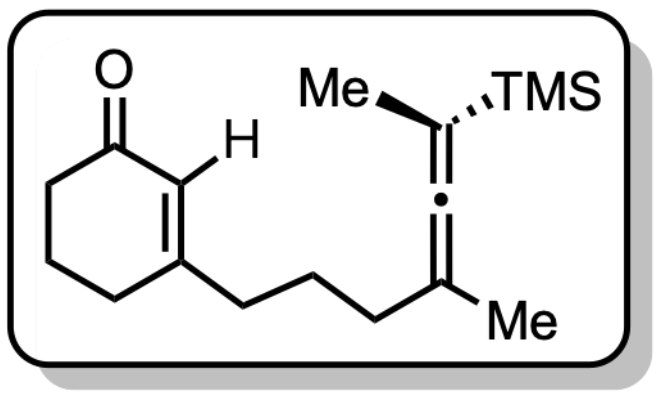

3-(4-methyl-6-(trimethylsilyl)hepta-4,5-dien-1-yl)cyclohex-2-en-1-one (26k)

^{1}H NMR: (400 MHz, $CDCl_3$): 5.84-5.84 (m, 1H), 2.34-2.31 (m, 2H), 2.26-2.19 (m, 4H), 1.99-1.92 (m, 2H), 1.87 (t, J = 7.3 Hz, 2H), 1.62 (s, 3H), 1.59 (s, 3H), 1.57-1.51 (m, 2H), 0.02 (s, 9H).

^{13}C NMR (100 MHz, $CDCl_3$): δ 203.9, 199.6, 166.4, 125.5, 91.6, 91.0, 37.5, 37.2, 32.9, 29.5, 24.9, 22.6, 18.6, 15.7,

-1.8.

HRMS (ESI+): calculated for $C_{17}H_{29}OSi$ $[M+H]^+$ 277.1982, found: 277.1984.

Physical Appearance: Pale yellow oil.

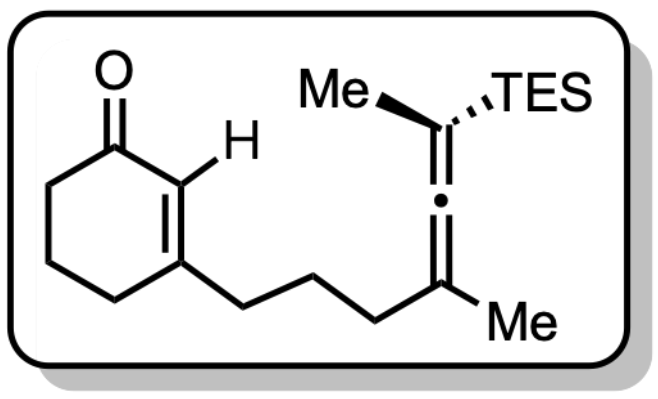

3-(4-methyl-6-(triethylsilyl)hepta-4,5-dien-1-yl)cyclohex-2-en-1-one (26l)

^{1}H NMR: (400 MHz, $CDCl_3$): δ 5.84 (s, 1H), 2.32 (t, J = 7.1 Hz, 2H), 2.26-2.19 (m, 4H), 1.97-1.84 (m, 5H), 1.60 (s, 3H), 1.59 (s, 3H), 1.57-1.55 (m, 1H), 0.92-0.87 (m, 9H), 0.56-0.49 (m, 6H).

^{13}C NMR (100 MHz, $CDCl_3$): δ 205.1, 199.9, 166.5, 125.7, 91.0, 87.9, 37.7, 37.3, 33.2, 29.7, 25.1, 22.7, 18.8, 16.7, 7.4, 3.1.

HRMS (ESI+): calculated for $C_{20}H_{35}OSi$ $[M+H]^+$ 319.2452, found: 319.2452.

Physical Appearance: colorless oil.

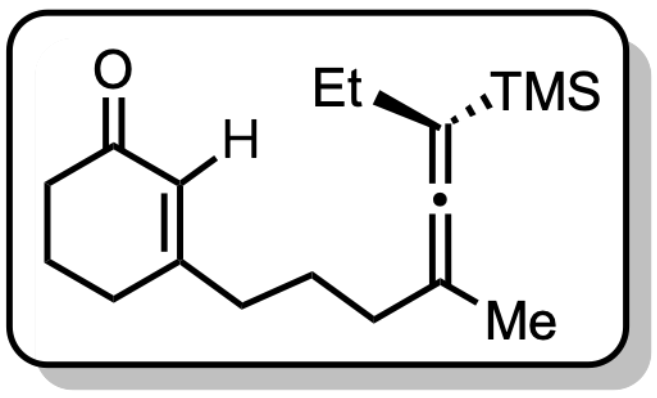

3-(4-methyl-6-(trimethylsilyl)octa-4,5-dien-1-yl)cyclohex-2-en-1-one (26m)

^{1}H NMR: (400 MHz, $CDCl_3$): δ 5.85 (s, 1H), 2.33 (t, *J* = 6.44 Hz, 2H), 2.26-2.19 (m, 4H), 1.99-1.87 (m, 6H), 1.61 (s, 3H), 1.58-1.52 (m, 2H), 0.96 (t, *J* = 7.36 Hz, 3H), 0.02 (s, 9H).

^{13}C NMR (100 MHz, $CDCl_3$): δ 203.5, 199.9, 166.5, 125.6, 98.5, 93.9, 37.8, 37.4, 33.2, 29.7, 25.2, 22.8, 22.7, 18.8, 14.0, -1.3.

HRMS (ESI+): calculated for $C_{18}H_{31}OSi$ $[M+H]^+$ 291.2139, found: 291.2145.

Physical Appearance: colorless oil.

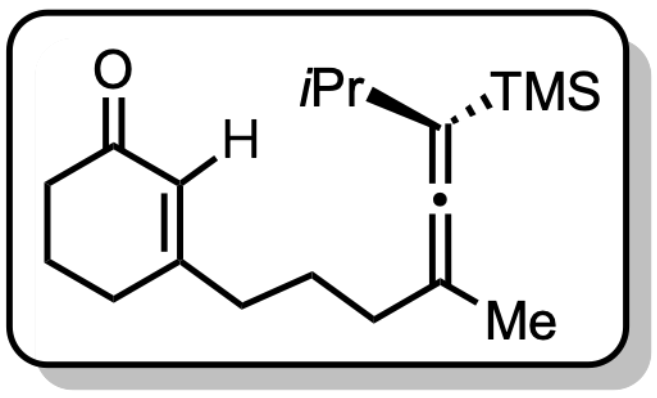

3-(4,7-dimethyl-6-(trimethylsilyl)octa-4,5-dien-1-yl)cyclohex-2-en-1-one (26n)

^{1}H NMR: (400 MHz, $CDCl_3$): δ 5.85 (s, 1H), 2.34 (t, *J* = 6.4 Hz, 2H), 2.27-2.21 (m, 4H), 2.19-2.12 (m, 1H), 1.99-1.93 (m, 2H), 1.91-1.88 (m, 2H), 1.61 (s, 3H), 1.59-1.53 (m, 2H), 0.98 (d, *J* = 6.7 Hz, 6H), 0.04 (s, 9H).

^{13}C NMR (100 MHz, $CDCl_3$): δ 202.9, 199.9, 166.4, 125.7, 104.0, 94.8, 37.9, 37.4, 33.2, 29.7, 29.2, 25.1, 23.9, 23.7, 22.7, 18.8, -0.7.

HRMS (ESI+): calculated for $C_{19}H_{33}OSi$ $[M+H]^+$ 305.2295, found: 305.2295.

Physical Appearance: Pale yellow oil.

General procedure for allene **SI-(3a-3d)** syntheses

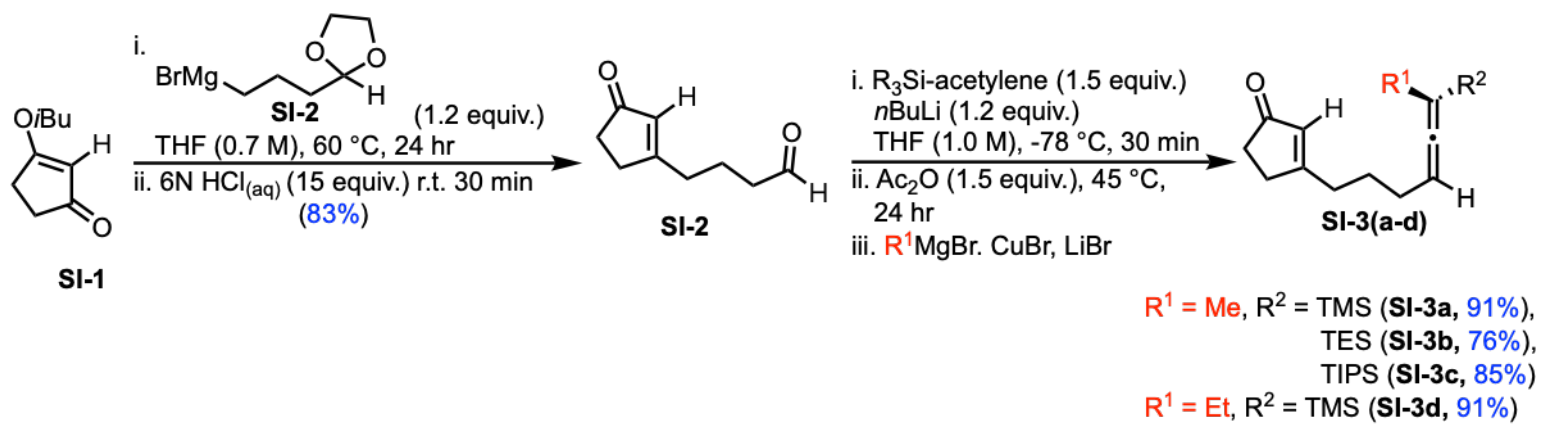

Under a nitrogen atmosphere, vinylogous ester **SI-1** (3.04 g, 0.0185 mol) in THF (49 mL) was placed in a 250 mL two-necked flask. The previously prepared Grignard reagent **SI-2** (1.1 equiv.) in THF (70.0 mL) was added dropwise at 0 °C, and the reaction mixture was heated to 60 °C and stirred for 1h. After complete consumption of the vinylogous ester **SI-1** monitored by TLC, the septum was removed, and excess glacial acetic acid (74 mL, 0.074 mmol) was added dropwise at 0 °C. The mixture was stirred for 1 h, and the reaction was then quenched by slow addition of saturated aqueous Na_2CO_3. The mixture was extracted with EtOAc (100 mL) and brine (100 mL) three times. The combined organic layers were dried over $MgSO_4$, filtered, and concentrated under reduced pressure to afford the crude product (acetal).

Under an ambient atmosphere, the crude acetal (500 mg, 2.548 mmol) was then placed in a 100 mL round-bottom flask and dissolved in a mixture of TFA and $CHCl_3$ (10.2 mL, 1:1, v/v, 0.5 M). The reaction mixture was stirred for 24 h at room temperature. The reaction was then quenched by slow addition of saturated aqueous $NaHCO_3$ at 0 °C. The mixture was extracted with $CHCl_3$ (100 mL) and brine (100 mL) three times. The combined organic layers were dried over $MgSO_4$, filtered, and concentrated under reduced pressure to afford the crude product. Purification by flash column chromatography (EtOAc/hexanes = 2:1) afforded the corresponding dicarbonyl compound (**SI-2**, 320 mg, 83%, HTH-II-176).

Under a nitrogen atmosphere, the corresponding silyl acetylene (0.51 mL, 3.558 mmol) was dissolved in THF (0.5 mL) in a 25 mL round-bottom flask. *n*-Butyllithium

(1.69 mL, 2.846 mmol) was added dropwise at 0 °C, and the reaction mixture was stirred at this temperature for 30 min. The mixture was then cooled to −78 °C, and a solution of the dicarbonyl compound **SI-2** (361 mg, 2.372 mmol) in THF (11.3 mL) was added in one portion. After complete consumption of the starting material monitored by TLC, acetic anhydride (0.34 mL, 3.558 mmol) was added, and the reaction mixture was heated to 60 °C and stirred for 24 h. The reaction was quenched with saturated aqueous $NaHCO_3$, and the mixture was extracted with EtOAc (100 mL) and brine (100 mL) three times. The combined organic layers were dried over $MgSO_4$, filtered, and concentrated under reduced pressure to afford the crude product.

Under a nitrogen atmosphere, CuBr·DMS (2.074 g, 10.089 mmol) and LiBr (876 mg, 2.522 mmol) were dissolved in THF (10.0 mL) in a 25 mL round-bottom flask. The MeMgBr Grignard reagent (3.36 mL, 10.089 mmol) was added dropwise at 0 °C, and the reaction mixture was stirred at this temperature for 30 min. The mixture was then cooled to −78 °C, and a solution of the corresponding crude propargyl acetate (773 mg, 2.522 mmol) in THF (3 mL) was added in one portion. After complete consumption of the starting material monitored by TLC, the reaction was quenched by slow addition of saturated aqueous NH_4Cl. The mixture was extracted with EtOAc (100 mL) and brine (100 mL) three times. The combined organic layers were dried over $MgSO_4$, filtered, and concentrated under reduced pressure to afford the crude product. Purification by flash column chromatography (EtOAc/hexanes = 1:6) afforded the corresponding allene **SI-3a** (542 mg, 91%, HTH-II-195).

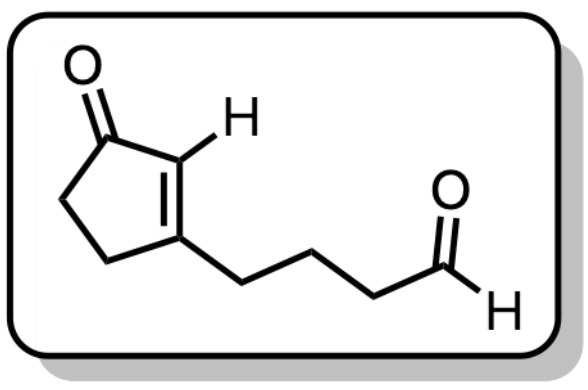

4-(3-oxocyclopent-1-en-1-yl)butanal (SI-2)

^{1}**H NMR** (400 MHz, $CDCl_3$): δ 9.78 (t, *J* = 1.1 Hz, 1H), 5.95-5.93 (m, 1H), 2.58-2.38 (m, 8H), 1.95-1.88 (m, 2H).

^{13}**C NMR** (100 MHz, $CDCl_3$): δ 209.6, 201.2, 181.3, 129.5, 42.9, 35.1, 32.4, 31.2, 19.1.

HRMS (ESI^+): calculated for $C_9H_{13}O_2$ $[M+H]^+$ 153.0910, found: 153.0909.

Physical Appearance: pale yellow liquid.

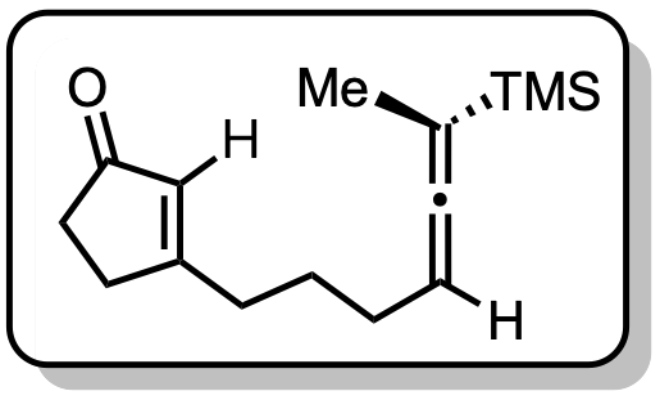

3-(6-(trimethylsilyl)hepta-4,5-dien-1-yl)cyclopent-2-en-1-one (SI-3a)

^{1}**H NMR** (400 MHz, $CDCl_3$): δ 5.97-5.86 (m, 1H), 4.84-4.62 (m, 1H), 2.62-2.51 (m, 2H), 2.43 (t, *J* = 7.6 Hz, 2H), 2.39-2.37 (m, 2H), 1.98 (q, *J* = 7.4 Hz, 2H), 1.71-1.61 (m, 5H), 0.05 (s, 9H).

^{13}**C NMR** (100 MHz, $CDCl_3$): δ 210.1, 205.7, 183.0, 129.5, 91.7, 83.1, 35.3, 32.9, 31.5, 27.9, 27.0, 15.7, -1.9.

HRMS (ESI^+): calculated for $C_{15}H_{25}OSi$ $[M+H]^+$ 249.1669, found: 249.1669.

Physical Appearance: colorless liquid.

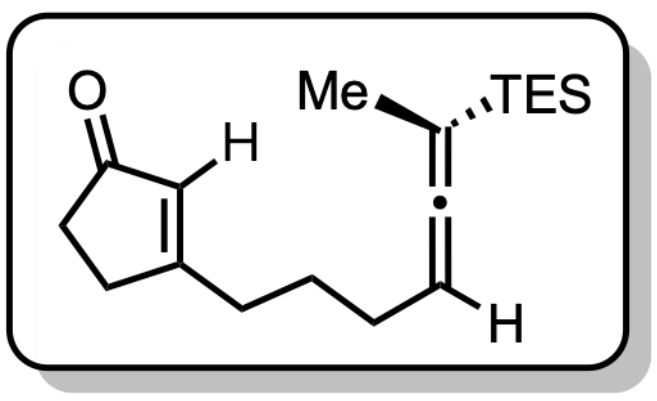

3-(6-(triethylsilyl)hepta-4,5-dien-1-yl)cyclopent-2-en-1-one (SI-3b)

^{1}**H NMR** (400 MHz, $CDCl_3$): δ 5.91 (m, 1H), 4.67 (m, 1H), 2.59-2.51 (m, 2H), 2.42 (t, *J* = 7.8 Hz, 2H), 2.39-2.34 (m, 2H), 1.97 (q, *J* = 6.9 Hz, 2H), 1.70-1.60 (m, 5H), 0.95-0.85 (m, 9H), 0.61-0.51 (m, 6H).

^{13}**C NMR** (100 MHz, $CDCl_3$): δ 210.1, 206.6, 182.9, 129.5, 88.5, 82.6, 35.3, 32.9, 31.5, 28.1, 27.0, 16.5, 7.3, 2.9.

HRMS (ESI+): calculated for $C_{18}H_{31}OSi$ $[M+H]^+$ 291.2139 , found: 291.2139.

Physical Appearance: colorless liquid.

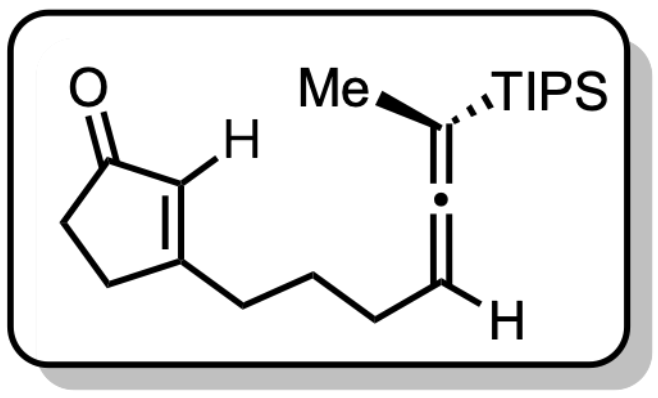

3-(6-(triisopropylsilyl)hepta-4,5-dien-1-yl)cyclopent-2-en-1-one (SI-3c)

^{1}**H NMR** (400 MHz, $CDCl_3$): δ 5.94-5.92 (m, 1H), 4.69-4.63 (m, 1H), 2.57-2.54 (m, 2H), 2.44 (t, J = 7.6 Hz, 2H), 2.40-2.37 (m, 2H), 2.04-1.98 (m, 2H), 1.71-1.63 (m, 2H), 1.69 (d, J = 2.9 Hz, 3H), 1.17-1.04 (m, 21H).

^{13}**C NMR** (100 MHz, $CDCl_3$): δ 209.6, 207.6, 182.5, 129.2, 86.6, 82.4, 35.0, 32.7, 31.3, 28.1, 27.0, 18.4, 17.7, 11.0.

HRMS (ESI^+): calculated for $C_{21}H_{37}OSi$ $[M+H]^+$ 333.2608, found: 333.2603.

Physical Appearance: colorless liquid.

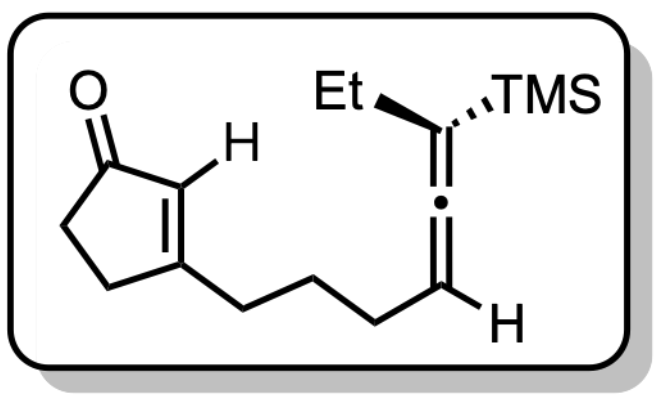

3-(4-methyl-6-(trimethylsilyl)octa-4,5-dien-1-yl)cyclopent-2-en-1-one (SI-3d)

^{1}**H NMR** (400 MHz, $CDCl_3$): δ 5.91-5.90 (m, 1H), 2.54-2.52 (m, 2H), 2.40 (t, J = 7.5 Hz, 2H), 2.37-2.35 (m, 2H), 1.93-1.88 (m, 4H), 1.67-1.64 (m, 2H), 1.61 (s, 3H), 0.94 (t, J = 7.5 Hz, 3H), 0.01 (s, 9H).

^{13}C NMR (100 MHz, $CDCl_3$): δ 210.1, 203.4, 183.1, 129.4, 98.7, 93.8, 35.3, 33.3, 33.2, 31.5, 25.3, 22.8, 18.9, 14.0, -1.3.

HRMS (ESI^+): calculated for $C_{17}H_{29}OSi$ $[M+H]^+$ 277.1982, found: 277.1982.

Physical Appearance: pale yellow oil.

Following the general procedure, the corresponding n-Bu-, i-Pr-, and allyl-substituted allenes were investigated. It is important to note that the reaction with allylMgCl resulted in unavoidable 1,2-addition to the enone, and therefore the desired allyl-substituted allene could not be obtained. Unfortunately, attempt to [3+2] annulation of either **SI-3e** or **SI-3f** failed to deliver the desired cyclization product.

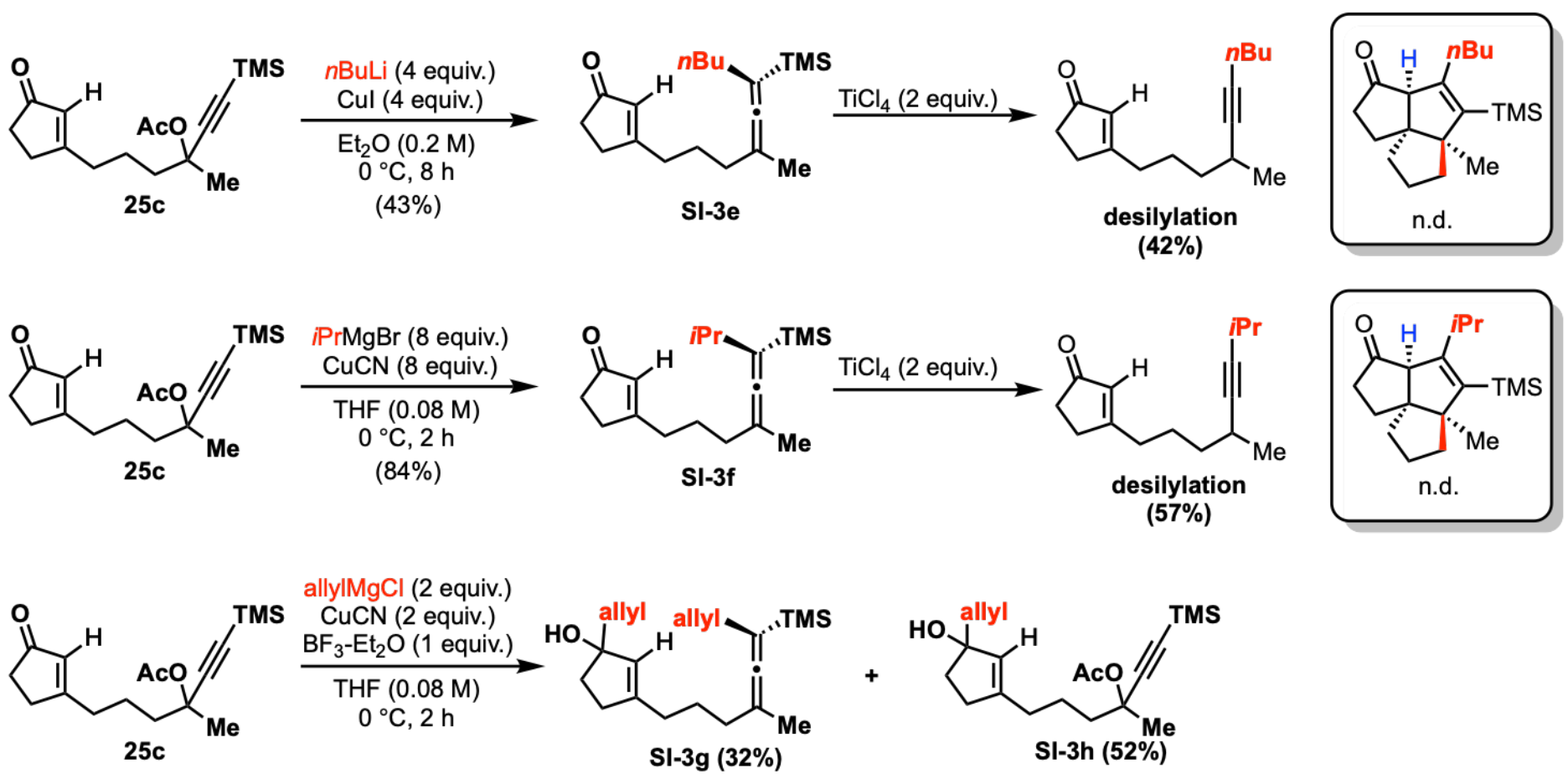

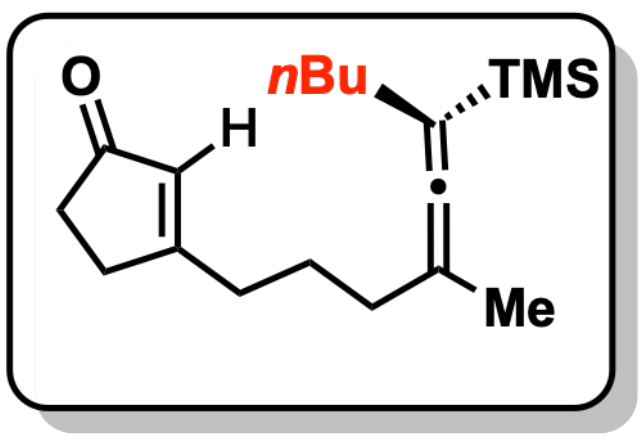

3-(4-methyl-6-(trimethylsilyl)deca-4,5-dien-1-yl)cyclopent-2-en-1-one (SI-3e)

^{1}H NMR (400 MHz, $CDCl_3$): δ 5.93-5.92 (m, 1H), 2.56-2.53 (m, 2H), 2.44-2.37 (m, 4H), 1.95-1.88 (m, 4H), 1.69-1.63 (m, 2H), 1.62 (s, 3H), 1.37-1.29 (m, 4H), 0.84 (t, *J* = 7.1 Hz, 3H), 0.03 (s, 9H).

^{13}C NMR (100 MHz, $CDCl_3$): δ 210.0, 203.7, 183.0, 129.3, 96.7, 93.0, 35.2, 33.2, 33.1, 31.6, 31.5, 29.4, 25.2, 22.4, 18.7, 14.0, -1.3.

HRMS (ESI^+): calculated for $C_{19}H_{33}OSi$ $[M+H]^+$ 305.2295, found: 305.2299.

Physical Appearance: pale yellow oil.

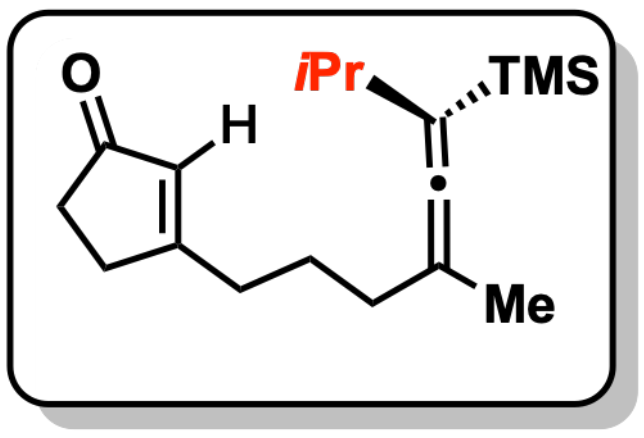

3-(4,7-dimethyl-6-(trimethylsilyl)octa-4,5-dien-1-yl)cyclopent-2-en-1-one (SI-3f)

^{1}H NMR (400 MHz, $CDCl_3$): δ 5.89 (s, 1H), 2.53-2.51 (m, 2H), 2.40 (t, *J* = 7.7Hz, 2H), 2.36-2.34 (m, 2H), 2.14 (t, *J* = 6.7 Hz, 1H), 1.92-1.88 (m, 2H), 1.67-1.61 (m, 2H), 1.60 (s, 3H), 0.94 (d, *J* = 6.6 Hz, 6H), 0.01 (s, 9H).

^{13}C NMR (100 MHz, $CDCl_3$): δ 210.0, 202.7, 183.0, 129.3, 104.1, 94.7, 35.2, 33.3, 33.1, 31.5, 29.1, 25.2, 23.9, 23.6, 18.8, -0.8.

HRMS (ESI^+): calculated for $C_{18}H_{31}OSi$ $[M+H]^+$ 291.2139, found: 291.2148.

Physical Appearance: pale yellow oil.

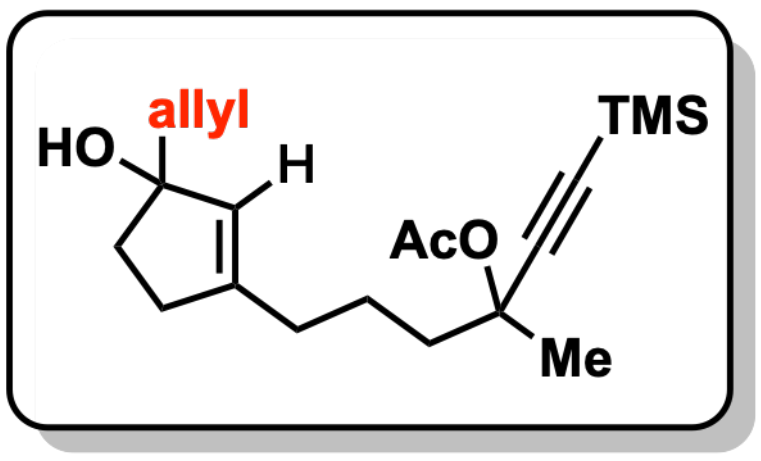

1-allyl-3-(4-hydroxy-4-methyl-6-(trimethylsilyl)hex-5-yn-1-yl)cyclopent-2-en-1-ol (SI-3g)

^{1}H NMR (400 MHz, $CDCl_3$): δ 5.88-5.77 (m, 1H), 5.35 (s, 1H), 5.12-5.08 (m, 2H), 2.35 (d, *J* = 7.5 Hz, 2H), 2.13-1.99 (m, 4H), 1.87-1.80 (m, 2H), 1.64-1.61 (m, 4H), 1.43 (s, 3H), 0.13 (s, 9H).

^{13}C NMR (100 MHz, $CDCl_3$): δ 148.1, 134.3, 129.4, 118.2, 109.5, 87.4, 85.1, 68.2, 45.6, 43.0, 37.8, 33.4, 31.0, 29.8, 29.7, 22.5, -0.1.

HRMS (ESI^+): calculated for $C_{18}H_{31}OSi$ $[M+Na]^+$ 329.1907, found: 329.1899.

Physical Appearance: pale yellow oil.

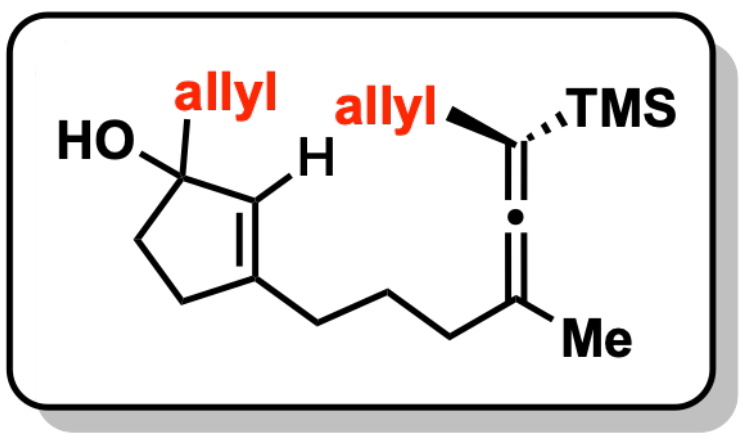

1-allyl-3-(4-methyl-6-(trimethylsilyl)nona-4,5,8-trien-1-yl)cyclopent-2-en-1-ol (SI-3h)

^{1}H NMR (400 MHz, $CDCl_3$): δ 5.88-5.84 (m, 2H), 5.30 (s, 1H), 5.12-5.08(m, 2H), 5.01-4.91 (m, 2H), 2.68 (d, J = 6.8 Hz, 2H), 2.35 (d, J = 7.3 Hz, 2H), 2.07-1.99 (m, 4H), 1.91-1.79 (m, 4H), 1.61 (s, 3H), 1.51 (t, J = 7.8 Hz, 2H), 0.03 (s, 9H).

^{13}C NMR (100 MHz, $CDCl_3$): δ 204.4, 148.5, 138.0, 134.3, 129.0, 118.1, 114.5, 94.6, 93.9, 85.0, 45.6, 37.8, 34.7, 33.5, 33.2, 30.9, 25.7, 18.6, -1.2.

HRMS (ESI^{+}): calculated for $C_{21}H_{34}NaOSi$ $[M+Na]^{+}$ 353.2271, found: 353.2272.

Physical Appearance: pale yellow oil.

General procedure for allene **SI-3**(**i-k**) syntheses:

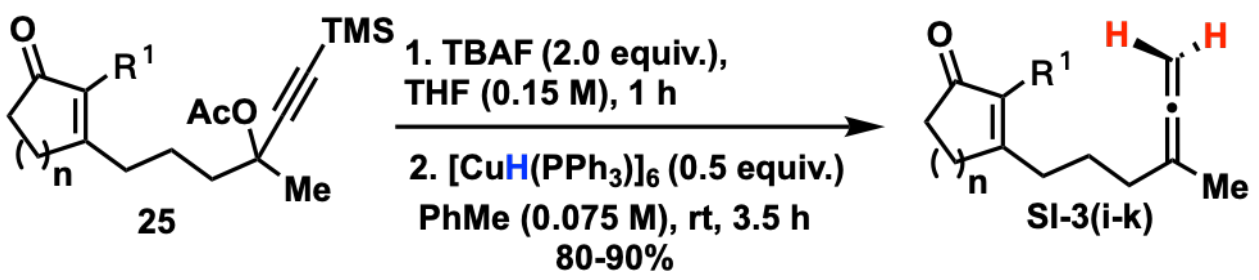

Under a nitrogen atmosphere, the starting material (1.8 g) was dissolved in THF (0.15 M), and TBAF (2.0 equiv.) was added. After complete consumption of the starting material, as monitored by TLC, the reaction was quenched with saturated aqueous NH_4Cl (20 mL). The mixture was extracted with EtOAc (3×), and the combined organic layers were washed with brine, dried over $MgSO_4$, filtered, and concentrated under reduced pressure. The crude product was purified by flash column chromatography (EtOAc/hexanes = 1:3) to afford the desired product in 92% yield.

Under a nitrogen atmosphere, Stryker's reagent (1.25 g) was suspended in toluene (0.08 M), and the starting material (791.2 mg) was added. The reaction mixture was stirred until complete consumption of the starting material, as monitored by TLC, and then filtered through a pad of Celite. The filtrate was extracted with EtOAc (3×), and the combined organic layers were washed with brine, dried over $MgSO_4$, filtered, and concentrated under reduced pressure. The crude product was purified by flash column chromatography (EtOAc/hexanes = 1:6) to afford the desired product in 98% yield.

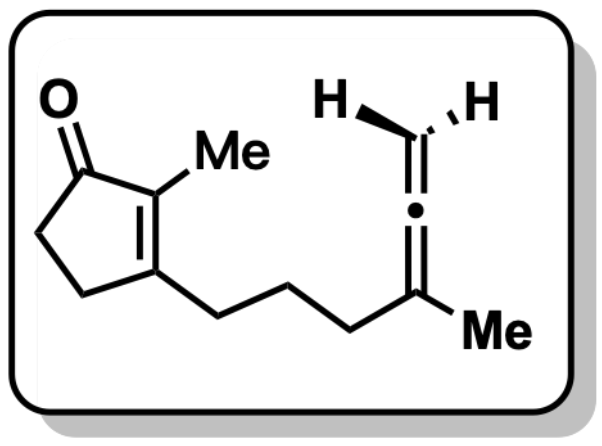

2-methyl-3-(4-methylhexa-4,5-dien-1-yl)cyclopent-2-en-1-one (SI-3I)

^{1}H NMR (400 MHz, $CDCl_3$): δ 4.58-4.54 (m, 2H), 2.45-2.42 (m, 2H), 2.38 (t, J = 7.4, 2H), 2.31-2.29 (m, 2H), 1.92-1.87 (m, 2H), 1.64-1.61 (m, 8H).

^{13}C NMR (100 MHz, $CDCl_3$): δ 210.0, 205.9, 173.3, 136.2, 97.6, 74.5, 34.0, 32.8, 30.6, 29.1, 24.7, 18.7, 7.9.

HRMS (ESI$^+$): calculated for $C_{13}H_{19}O$ $[M+H]^+$ 191.1430, found: 191.1431.

Physical Appearance: colorless oil.

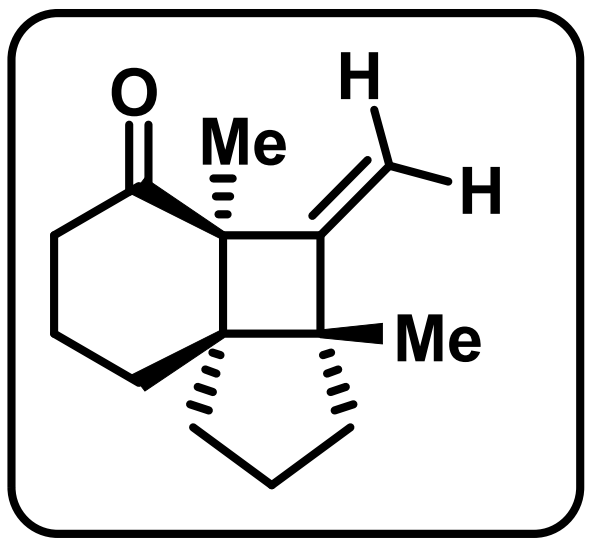

(3aS,4aS,8aR)-3a,4a-dimethyl-4-methyleneoctahydrocyclopenta[1,4]cyclobuta[1,2]benzen-5(6H)-one (SI-3j)

^{1}H NMR: (400 MHz, $CDCl_3$): δ 5.01-5.00 (m, 1H), 4.82-4.81 (m, 1H), 2.37-2.25 (m, 2H), 1.95-1.81 (m, 2H), 1.74-1.50 (m, 6H), 1.39-1.26 (m, 2H), 1.16-1.13 (m, 3H), 1.11-1.10 (m, 3H).

^{13}C NMR (100 MHz, $CDCl_3$): δ 214.6, 160.5, 105.1, 56.7, 54.9, 53.1, 40.6, 38.3, 37.9, 30.7, 24.9, 22.2, 20.2, 18.2.

HRMS (ESI+): calculated for $C_{14}H_{21}O$ $[M+H]^+$ 205.1587, found: 205.1589.

Physical Appearance: colorless liquid.

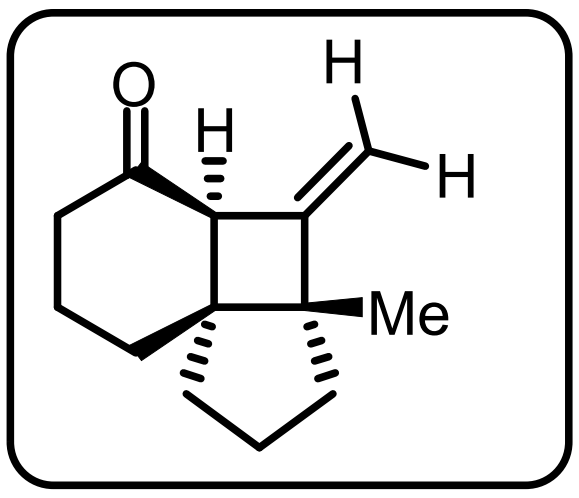

(3aS,4aS,8aS)-3a-methyl-4-methyleneoctahydrocyclopenta[1,4]cyclobuta[1,2]benzen-5(6H)-one (SI-3k)

^{1}H NMR: (400 MHz, $CDCl_3$): δ 4.90-4.89 (m, 1H), 4.80-4.79 (1H), 3.12-3.10 (m, 1H), 2.45-2.38 (m, 1H), 2.15-2.07 (m, 1H), 1.98-1.88 (m, 2H), 1.87-1.77 (m, 4H), 1.58-1.46 (m, 4H), 1.15 (s, 3H).

^{13}C NMR (100 MHz, $CDCl_3$): δ 209.9, 153.4, 104.8, 55.9, 54.0, 48.8, 42.3, 40.9, 39.6, 28.7, 23.8, 20.6.

HRMS (ESI+): calculated for $C_{13}H_{19}O$ $[M+H]^+$ 191.1430, found: 191.1435.

Physical Appearance: colorless liquid.

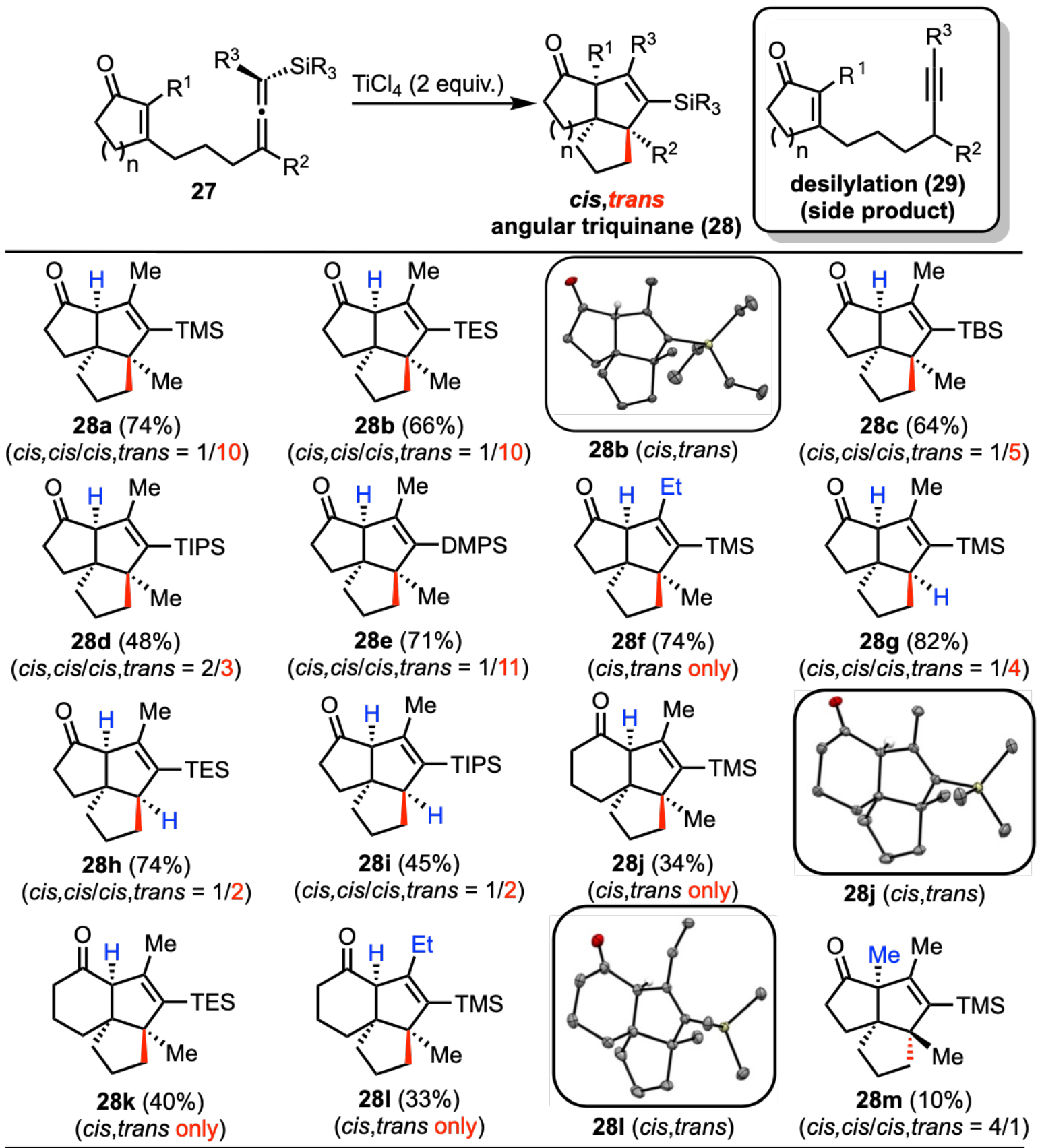

a) Reaction conditions: To SM (1 equiv., 0.2-0.4 mmol) in (0.02 M DCM) was added $TiCl_4$ (2.0 equiv.). The reaction was stirred at 40 °C until the consuption of SM monitoring with TLC. b) The reaction was carried out up to 3.8 mmol (SM) scale.

General procedure for [3+2]-Danheiser annulation

Under a nitrogen atmosphere, the corresponding allene **27** (300 mg, 1.143 mmol) was dissolved in CH_2Cl_2 (57 mL, 0.02 M) in a 100 mL round-bottom flask and preheated to 40 °C. Freshly distilled $TiCl_4$ (250 uL, 2.286 mmol) was then added in one portion. After complete consumption of the starting material monitored by TLC, the reaction was quenched with saturated aqueous $NaHCO_3$. The mixture was filtered through a pad of Celite, and the filtrate was extracted with CH_2Cl_2 (100 mL) and brine (100 mL) three times. The combined organic layers were dried over $MgSO_4$, filtered, and concentrated under reduced pressure to afford the crude product. The crude mixture was first subjected to flash column chromatography (8 cm silica gel column, EtOAc/hexanes = 1:6) to separate the alkyne byproduct from a mixture of cyclized products (*cis*,*cis* and *cis*,*trans*). The cyclized product mixture was further purified by

flash column chromatography (25 cm silica gel column, EtOAc/hexanes = 1:40) to afford the corresponding cyclized product **28a** (*cis*,*cis* 21 mg, 7%) and (*cis*,*trans* 201 mg, 67%)(HTH-II-138).

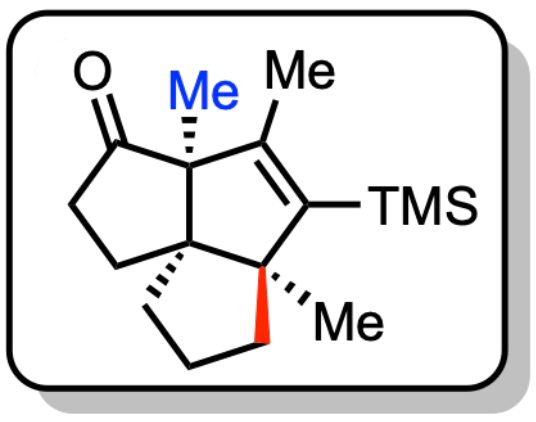

3a,4,5a-trimethyl-5-(trimethylsilyl)-1,2,5a,6,7,8-hexahydrocyclopenta[c]pentalen-3(3aH)-one (28m-*cis*,*trans*)

^{1}H NMR (500 MHz, $CDCl_3$): δ 2.23-2.06 (m, 4H), 1.76-1.67 (m, 2H), 1.61 (s, 3H), 1.59-1.49 (m, 2H), 1.38-1.25 (m, 2H), 1.14 (s, 3H), 0.99 (d, J = 1 Hz, 3H), 0.12 (s, 9H).

^{13}C NMR (125 MHz, $CDCl_3$): δ 219.9, 152.1, 145.8, 67.3, 66.6, 62.9, 36.9, 33.7, 30.1, 27.2, 26.4, 23.0, 16.0, 14.1, -0.1.

HRMS (ESI^+): calculated for $C_{17}H_{29}OSi$ $[M+H]^+$ 277.1982, found: 277.1974.

Physical Appearance: pale yellow oil.

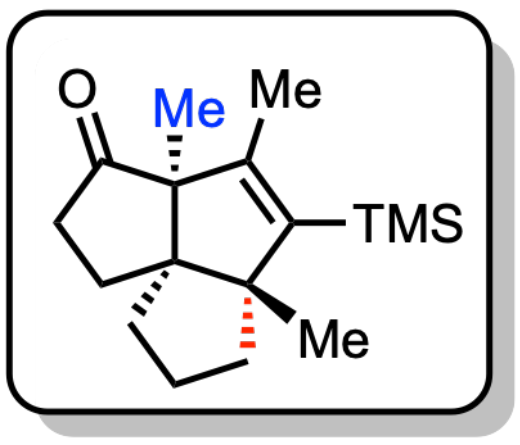

3a,4,5a-trimethyl-5-(trimethylsilyl)-1,2,5a,6,7,8-hexahydrocyclopenta[c]pentalen-3(3aH)-one (28m-*cis*,*cis*)

^{1}H NMR (500 MHz, $CDCl_3$): δ 2.39-2.29 (m, 1H), 2.13-2.05 (m, 2H), 1.83-1.71 (m, 2H), 1.72 (s, 3H), 1.60-1.20 (m, 5H), 1.18 (s, 3H), 1.04 (s, 3H), 0.14 (s, 9H).

^{13}C NMR (125 MHz, $CDCl_3$): δ 220.7, 148.3, 145.1, 67.9, 62.9, 61.4, 42.2, 38.2, 37.3, 29.2, 24.2, 23.1, 16.4, 13.4, 1.2.

HRMS (ESI^+): calculated for $C_{17}H_{29}OSi$ $[M+H]^+$ 277.1982, found: 277.1980.

Physical Appearance: White solid.

****

Distinct ^{1}H NMR Features of *cis*,*cis*- and *cis*,*trans*-Series Compounds

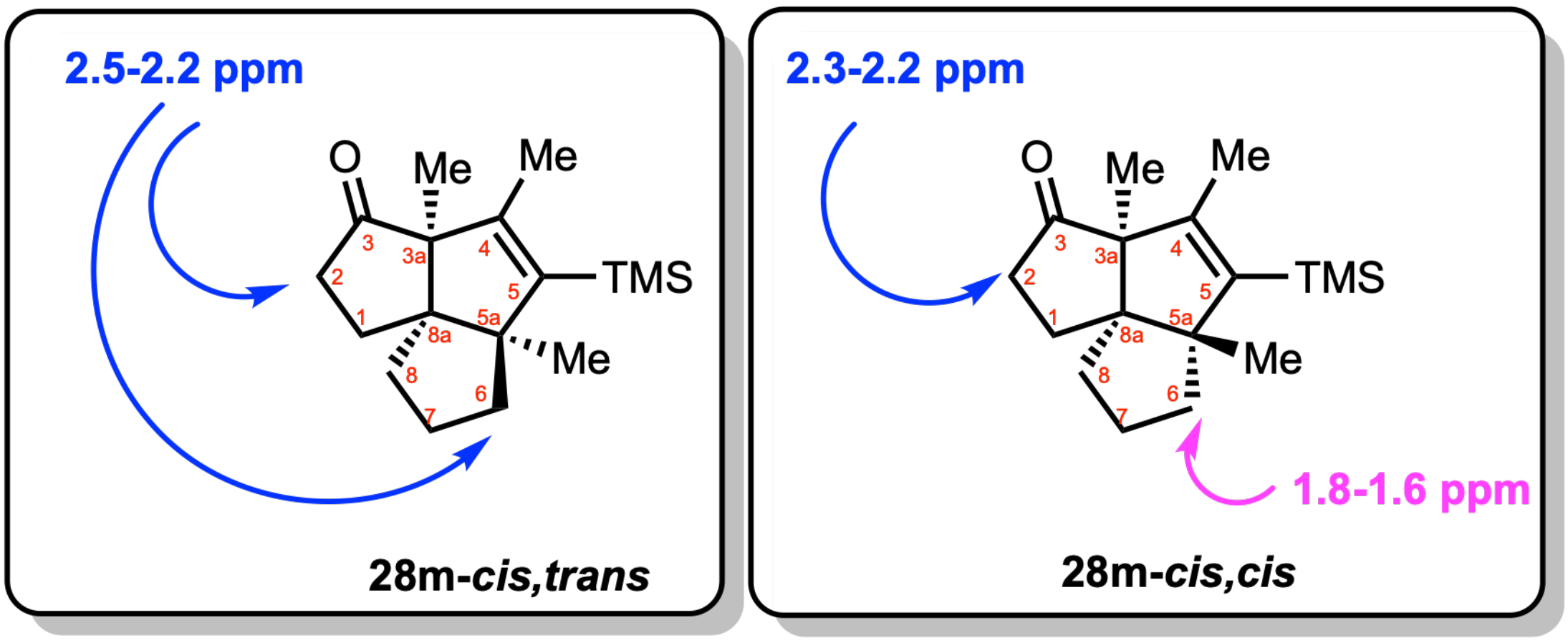

A comparison of the *cis*,*trans* and *cis*,*cis* series isomers reveals a clear difference in the chemical shift of the C(6) methylene group. In the *cis*,*trans* isomers, the C(6)-CH_2 resonance appears significantly downfield (δ 2.2–2.5 ppm), whereas in the *cis*,*cis* isomers it is observed in the typical aliphatic methylene region (δ 1.6–1.8 ppm). X-ray crystallographic analysis indicates that, in the *cis*,*trans* isomers, the C(6) methylene group is positioned in close proximity to, and adopts a near-coplanar arrangement with, the adjacent alkene π-system. This orientation places the C(6)-CH_2 protons within a region of enhanced anisotropic deshielding associated with the alkene, thereby accounting for the observed downfield shift. In contrast, the corresponding methylene group in the *cis*,*cis* isomers adopts an orientation that does not place it within this deshielding region, and thus resonates in the normal aliphatic methylene region.

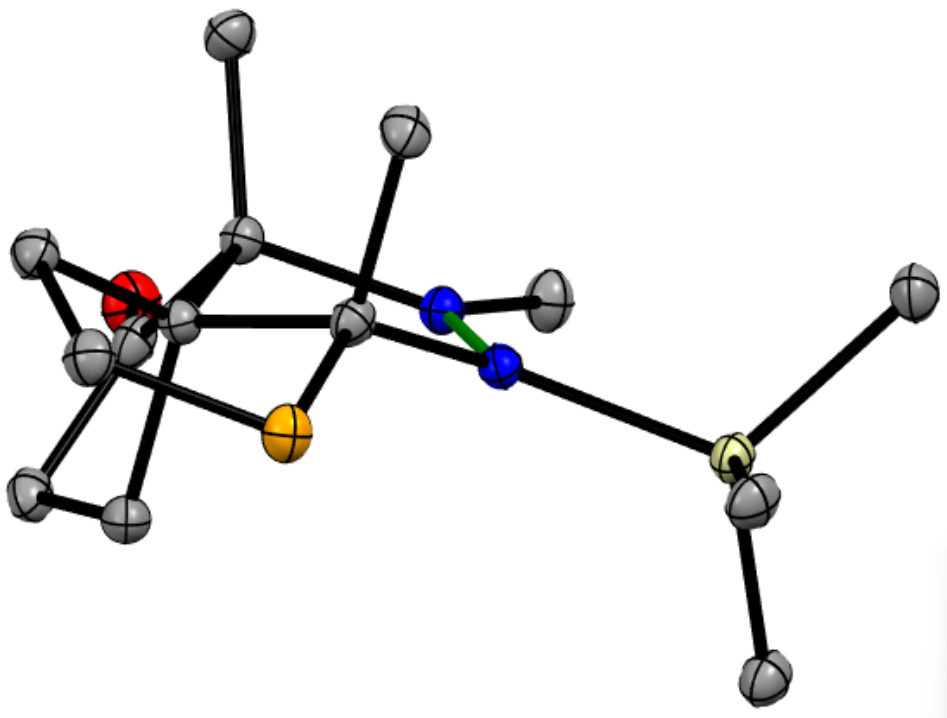

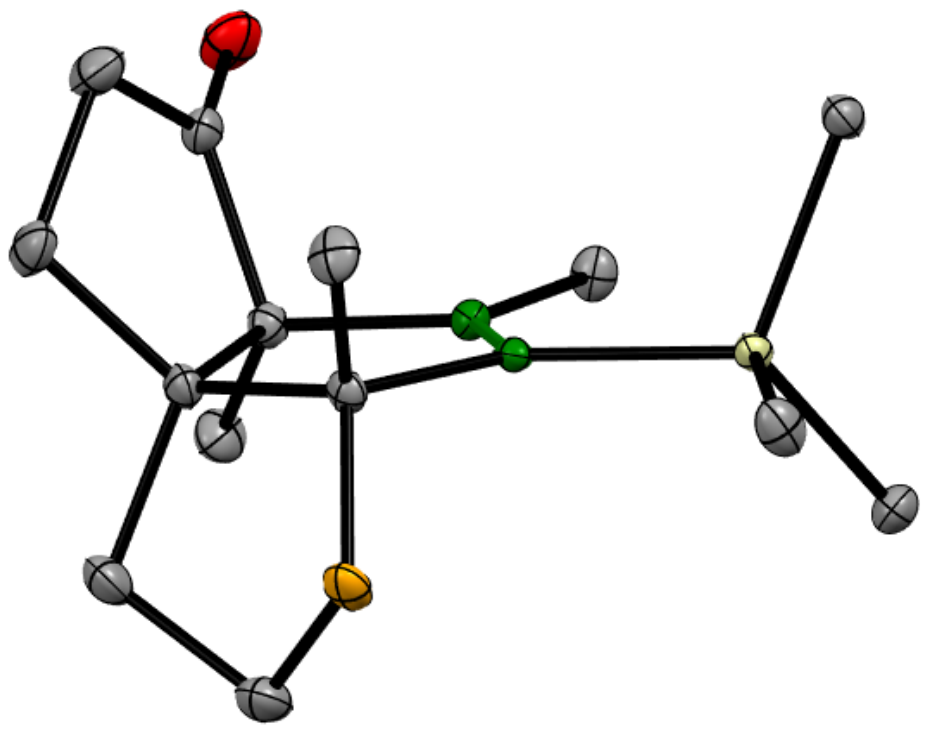

28m-*cis,trans* (left) **28m-*cis,cis*** (right)

****

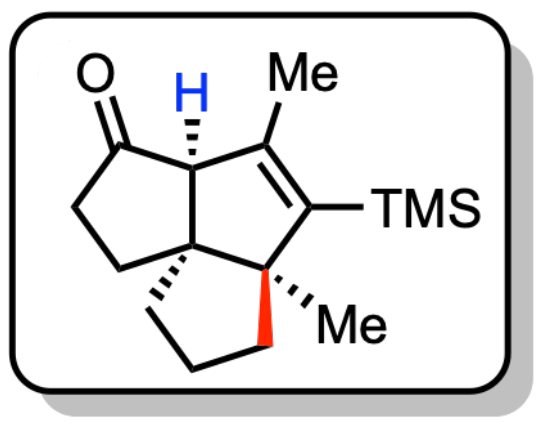

4,5a-dimethyl-5-(trimethylsilyl)-1,2,5a,6,7,8-hexahydrocyclopenta[c]pentalen-3(3aH)-one (28a-*cis,trans*)

^{1}H NMR (500 MHz, $CDCl_3$): δ 2.81 (s, 1H), 2.31-2.09 (m, 4H), 1.88-1.40 (m, 6H), 1.72 (d, J = 1.2 Hz, 3H), 0.84 (s, 3H), 0.13 (s, 9H).

^{13}C NMR (125 MHz, $CDCl_3$): δ 218.1, 148.1, 145.9, 67.4, 64.6, 62.9, 38.6, 33.2, 28.8, 27.1, 25.6, 16.5, 0.0.

HRMS (ESI$^+$): calculated for $C_{16}H_{27}OSi$ $[M+H]^+$ 263.1826, found: 263.1824.

Physical Appearance: colorless liquid.

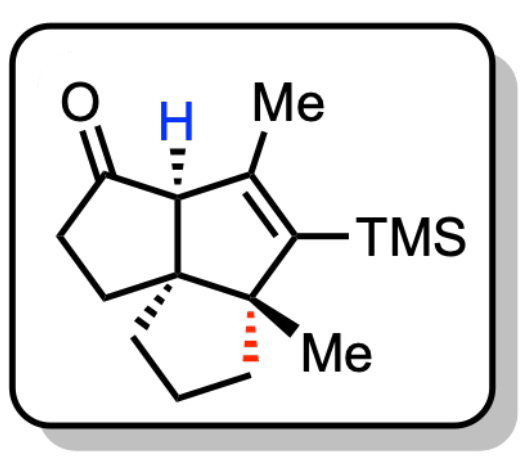

4,5a-dimethyl-5-(trimethylsilyl)-1,2,5a,6,7,8-hexahydrocyclopenta[c]pentalen-3(3aH)-one (28a-*cis*,*cis*)

^{1}H NMR (500 MHz, $CDCl_3$): δ 2.71 (s, 1H), 2.36-2.29 (m, 1H), 2.19-2.10 (m, 2H), 1.83 (s, 3H), 1.79-1.27 (m, 7H), 1.17 (s, 3H), 0.15 (s, 9H).

^{13}C NMR (125 MHz, $CDCl_3$): δ 218.3, 146.0, 144.5, 73.9, 64.0, 58.9, 42.4, 41.9, 38.8, 29.7, 24.3, 23.5, 15.7, 1.1.

HRMS (ESI^+): calculated for $C_{16}H_{27}OSi$ $[M+H]^+$ 263.1826, found: 263.1833.

Physical Appearance: colorless oil.

- **gram-scale intramolecular [3+2] Danheiser annulation**, demonstrating the practicality and scalability of the methodology.

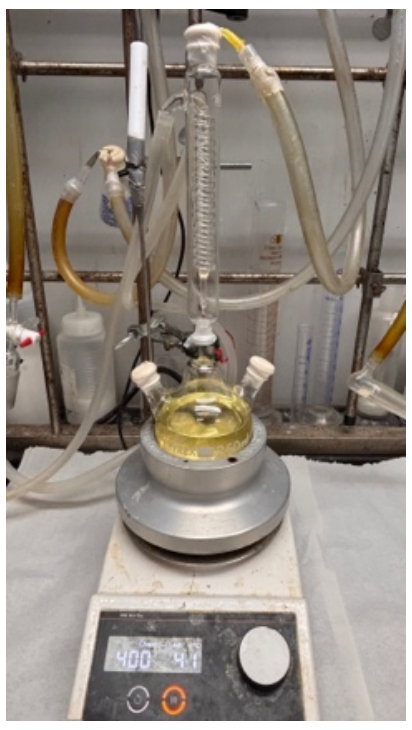

Allene in DCM

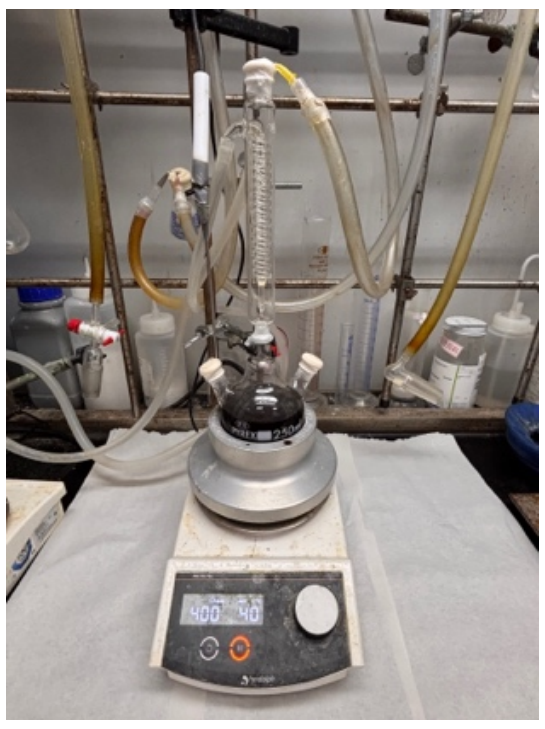

Allene w/ $TiCl_4$

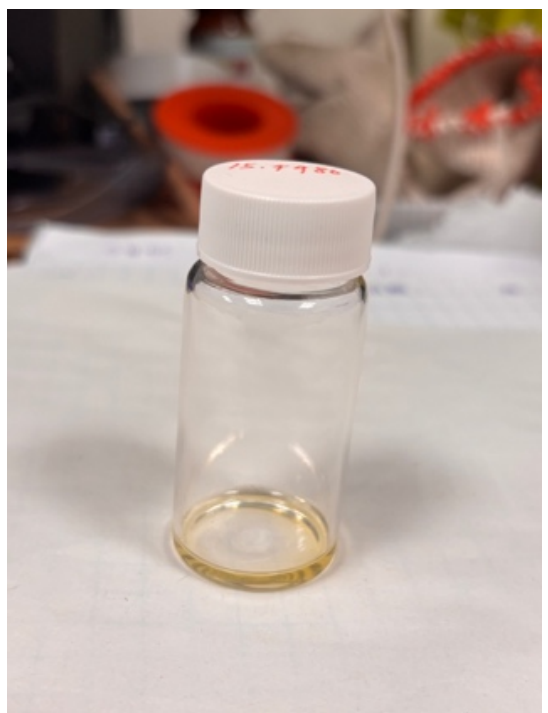

product

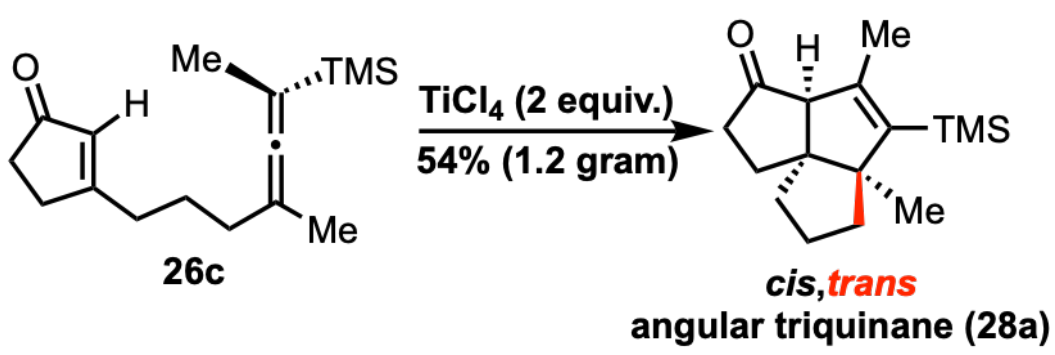

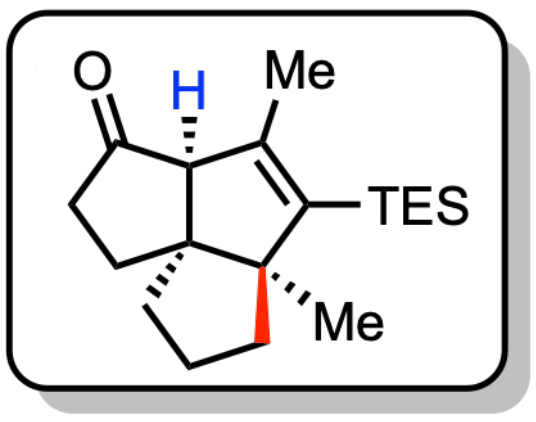

4,5a-dimethyl-5-(triethylsilyl)-1,2,5a,6,7,8-hexahydrocyclopenta[c]pentalen-3(3aH)-one (28b-*cis*,*trans*)

^{1}H NMR (500 MHz, $CDCl_3$): δ 2.83 (s, 1H), 2.31-2.08 (m, 4H), 1.97-1.90 (m, 1H), 1.73 (d, J = 1.7 Hz, 3H), 1.66-1.41 (series of m, 5H), 0.92 (t, J = 8.0 Hz, 9H), 0.84 (s, 3H), 0.67 (q, J = 8.0 Hz, 6H).

^{13}C NMR (125 MHz, $CDCl_3$): δ 218.2, 149.0, 144.0, 67.2, 64.8, 62.9, 38.5, 33.2, 29.0, 27.0, 25.8, 25.7, 16.7, 7.6, 4.0.

HRMS (ESI$^+$): calculated for $C_{19}H_{33}OSi$ $[M+H]^+$ 305.2295, found: 305.2295.

Physical Appearance: white solid.

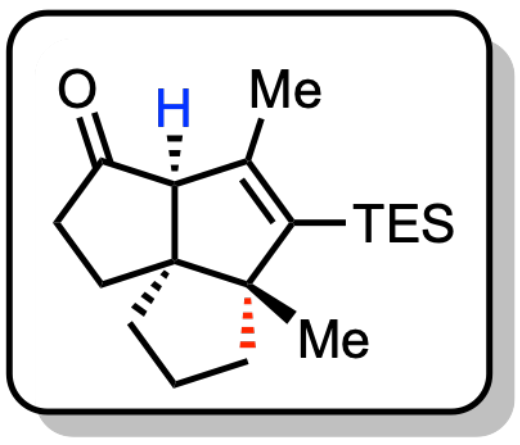

4,5a-dimethyl-5-(triethylsilyl)-1,2,5a,6,7,8-hexahydrocyclopenta[c]pentalen-3(3aH)-one (28b-*cis*,*cis*)

^{1}H NMR (500 MHz, $CDCl_3$): δ 2.27 (s, 1H), 2.36-2.28 (m, 1H), 2.17-2.11 (m, 2H), 1.82 (d, J = 1.2 Hz, 3H), 1.75-1.66 (m, 3H), 1.57-1.48 (m, 2H), 1.37-1.26 (m, 2H), 1.16 (s, 3H), 0.88 (t, J = 7.8 Hz, 9H), 0.68 (q, J = 7.8 Hz, 6H).

^{13}C NMR (125 MHz, $CDCl_3$): δ 218.1, 145.0, 143.8, 74.0, 63.6, 58.8, 42.4, 42.2, 38.6, 29.7, 24.2, 23.8, 15.6, 7.7, 4.2.

HRMS (ESI+): calculated for $C_{19}H_{33}OSi$ $[M+H]^+$ 305.2295, found: 305.2296.

Physical Appearance: colorless oil.

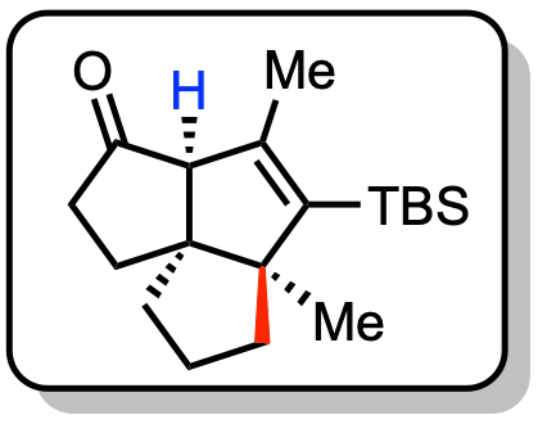

5-(tert-butyldimethylsilyl)-4,5a-dimethyl-1,2,5a,6,7,8-hexahydrocyclopenta[c]pentalen-3(3aH)-one (28c-*cis*,*trans*)

^{1}H NMR (500 MHz, $CDCl_3$): δ 2.83 (s, 1H), 2.32-2.09 (m, 4H), 2.02-1.92 (m, 1H), 1.74 (d, J = 1.2 Hz, 3H), 1.73-1.41 (m, 5H), 0.88 (s, 9H), 0.85 (s, 3H), 0.17 (s, 3H), 0.11 (s, 3H).

^{13}C NMR (125 MHz, $CDCl_3$): δ 218.1, 149.9, 144.3, 67.8, 64.7, 63.2, 38.5, 33.1, 28.9, 27.3, 27.0, 25.9, 25.9, 18.8, 17.8, -3.0, -4.5.

HRMS (ESI^+): calculated for $C_{19}H_{33}OSi$ $[M+H]^+$ 305.2295, found: 305.2294.

Physical Appearance: white solid.

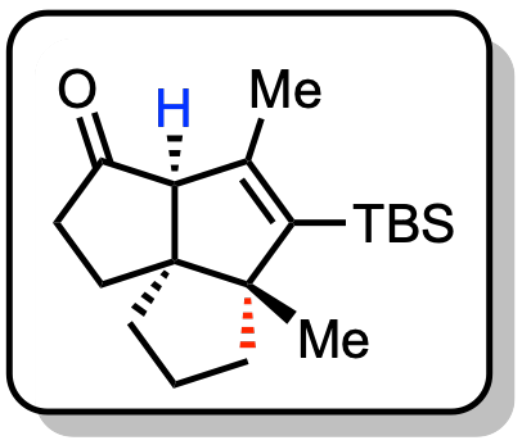

5-(tert-butyldimethylsilyl)-4,5a-dimethyl-1,2,5a,6,7,8-hexahydrocyclopenta[c]pentalen-3(3aH)-one (28c-*cis*,*cis*)

^{1}H NMR (500 MHz, $CDCl_3$): δ 2.74 (s, 1H), 2.35-2.26 (m, 1H), 2.20-2.08 (m, 2H), 1.83 (s, 3H), 1.78-1.33 (m, 7H), 1.18 (s, 3H), 0.86 (s, 9H), 0.16 (s, 3H), 0.15 (s, 3H).

^{13}C NMR (125 MHz, $CDCl_3$): δ 217.9, 145.9, 144.1, 73.7, 63.8, 59.3, 43.0, 41.0, 38.7, 29.8, 28.0, 24.3, 24.2, 18.7, 17.3, -1.8, -2.5.

HRMS (ESI^+): calculated for $C_{19}H_{33}OSi$ $[M+H]^+$ 305.2295, found: 305.2302.

Physical Appearance: colorless oil.

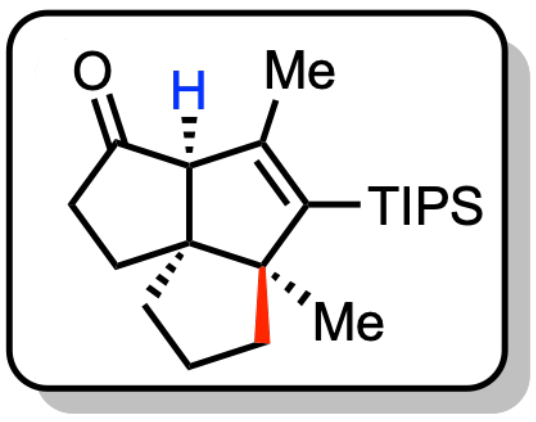

4,5a-dimethyl-5-(triisopropylsilyl)-1,2,5a,6,7,8-hexahydrocyclopenta[c]pentalen-3(3aH)-one (28d-*cis*,*trans*)

^{1}H NMR (500 MHz, $CDCl_3$): δ 2.84 (s, 1H), 2.31-2.02 (m, 5H), 1.82-1.77 (m, 1H), 1.75 (d, *J* = 1.1 Hz, 3H), 1.63-1.41 (m, 4H), 1.29-1.22 (m, 3H), 1.08 (d, *J* = 7.4 Hz, 18H), 0.94 (s, 3H).

^{13}C NMR (125 MHz, $CDCl_3$): δ 218.2, 150.0, 144.2, 67.5, 64.7, 63.1, 38.4, 33.1, 29.5, 27.0, 26.1, 25.9, 19.6, 19.4, 18.2, 13.0.

HRMS (ESI^+): calculated for $C_{22}H_{39}OSi$ $[M+H]^+$ 347.2765, found: 347.2767.

Physical Appearance: pale yellow oil.

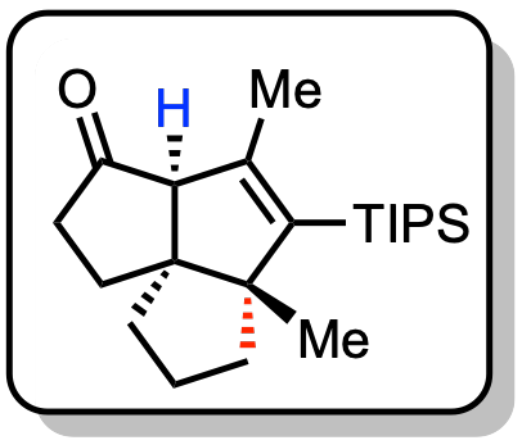

4,5a-dimethyl-5-(triisopropylsilyl)-1,2,5a,6,7,8-hexahydrocyclopenta[c]pentalen-3(3aH)-one (28d-*cis*,*cis*)

^{1}H NMR (500 MHz, $CDCl_3$): δ 2.76 (s, 1H), 2.35-2.09 (m, 4H), 1.87-1.84 (m, 1H), 1.83 (s, 3H), 1.79-1.66 (m, 2H), 1.58-1.57 (m, 1H), 1.44-1.39 (m, 2H), 1.35-1.27 (m, 3H), 1.26 (s, 3H), 1.07 (dd, J = 7.4, 1.2 Hz, 18H).

^{13}C NMR (125 MHz, $CDCl_3$): δ 218.0, 145.4, 143.7, 73.6, 63.9, 59.6, 42.9, 40.4, 38.7, 29.9, 24.4, 24.3, 19.8, 19.6, 17.1, 13.2.

HRMS (ESI^+): calculated for $C_{22}H_{39}OSi$ $[M+H]^+$ 347.2765, found: 347.2759.

Physical Appearance: colorless oil.

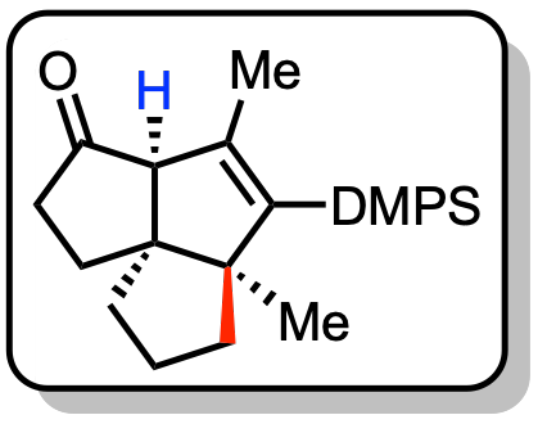

5-(dimethyl(phenyl)silyl)-4,5a-dimethyl-1,2,5a,6,7,8-hexahydrocyclopenta[c] pentalen-3(3aH)-one (28e-*cis*,*trans*)

^{1}H NMR (400 MHz, $CDCl_3$): δ 7.50-7.47 (m, 2H), 7.33-7.30 (m, 3H), 2.85 (s, 1H), 2.33-2.09 (m, 5H), 1.94-1.85 (m, 1H), 1.63-1.24 (m, 4H), 1.58 (d, J = 1.2 Hz, 3H), 0.89 (s, 3H), 0.40 (s, 3H), 0.38 (s, 3H).

^{13}C NMR (100 MHz, $CDCl_3$): δ 217.7, 150.1, 144.1, 138.8, 133.7, 128.9, 127.8, 67.3, 64.6, 62.8, 38.5, 33.2, 28.7, 27.0, 25.8, 25.7, 16.9, -1.2, -1.5.

HRMS (ESI^{+}): calculated for $C_{21}H_{29}OSi$ $[M+H]^{+}$ 325.1982, found: 325.1978.

Physical Appearance: colorless oil.

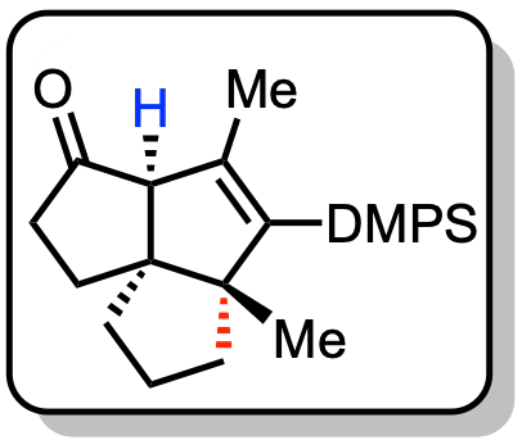

5-(dimethyl(phenyl)silyl)-4,5a-dimethyl-1,2,5a,6,7,8-hexahydrocyclopenta[c]pentalen-3(3aH)-one (28e-*cis*,*cis*)

^{1}H NMR (400 MHz, $CDCl_3$): δ 7.45-7.42 (m, 2H), 7.31-7.29 (m, 3H), 2.76 (s, 1H), 2.42-2.32 (m, 1H), 2.23-2.14 (m, 2H), 1.88-1.53 (m, 5H), 1.61 (d, J = 0.9 Hz, 3H), 1.43-1.36 (m, 2H), 1.22 (s, 3H), 0.39 (s, 6H).

^{13}C NMR (100 MHz, $CDCl_3$): δ 217.8, 146.6, 143.8, 139.8, 133.6, 128.7, 127.8, 73.9, 64.0, 59.0, 42.4, 42.1, 38.8, 29.7, 24.4, 23.7, 16.2, 0.2, -0.1.

HRMS (ESI^{+}): calculated for $C_{21}H_{29}OSi$ $[M+H]^{+}$ 325.1982, found: 325.1979.

Physical Appearance: colorless oil.

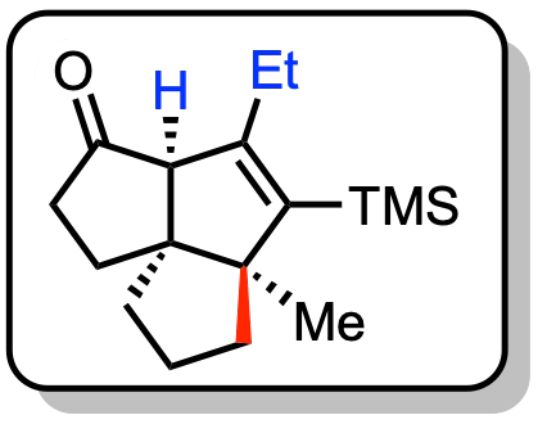

(3aR,5aS,8aS)-4-ethyl-5a-methyl-5-(trimethylsilyl)-1,2,5a,6,7,8-hexahydrocyclopenta[c]pentalen-3(3aH)-one (28f-*cis*,*trans*)

^{1}H NMR (400 MHz, $CDCl_3$): δ 3.22 (s, 1H), 2.39-2.22 (m, 2H), 2.19-2.12 (m, 1H), 2.09-2.00 (m, 2H), 1.87-1.84 (m, 1H), 1.73-1.63 (m, 3H), 1.51-1.35 (m, 3H), 0.91 (t, *J* = 7.5 Hz, 3H), 0.80 (d, *J* = 1.2 Hz, 3H), 0.13 (s, 9H).

^{13}C NMR (100 MHz, $CDCl_3$): δ 214.5, 156.6, 143.7, 67.1, 64.3, 60.0, 40.7, 29.9, 29.0, 26.9, 25.4, 25.0, 23.1, 22.9, 13.4, 0.27.

Physical Appearance: pale yellow oil.

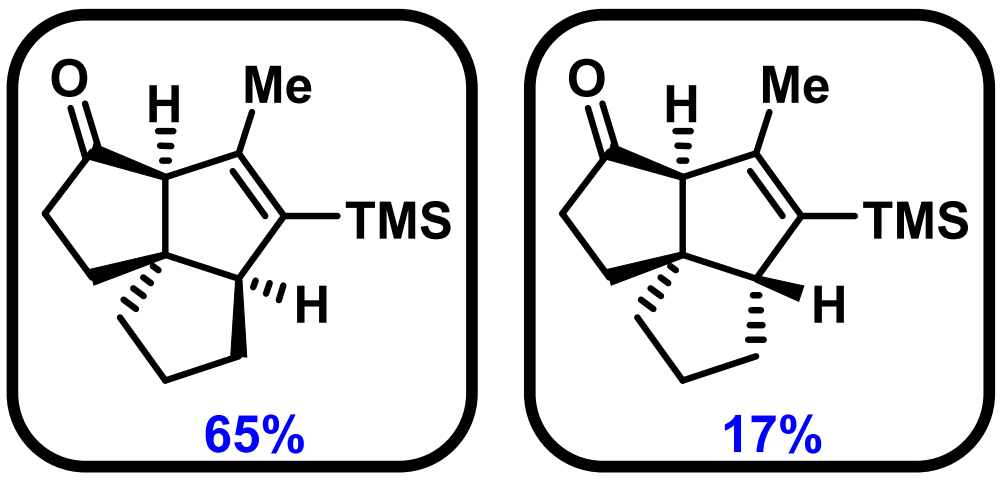

(3aR,5aS,8aS)-4-methyl-5-(trimethylsilyl)-1,2,5a,6,7,8-hexahydrocyclopenta[c] pentalen-3(3aH)-one (28g-*cis*,*trans* and **28g-*cis*,*cis*)**

^{1}H NMR (400 MHz, $CDCl_3$): δ 2.99-2.94 (m, $1H_{cis,cis}$), 2.86-2.78 (m, $1H_{cis,trans}$), 2.74 (s, $1H_{cis,cis}$), 2.68 (s, $1H_{cis,trans}$), 2.40-2.32 (m, $1H_{cis,cis}$), 2.29-2.08 (m, $1H_{cis,cis}$ and $4H_{cis,trans}$), 1.99-1.93 (m, $1H_{cis,cis}$), 1.87-1.38 (m, $7H_{cis,cis}$ and $6H_{cis,trans}$), 0.11 (s, $9H_{cis,trans}$), 0.10 (s, $9H_{cis,cis}$).

^{13}C NMR (100 MHz, $CDCl_3$): δ $218.4_{cis,trans}$, $217.1_{cis,cis}$, $149.8_{cis,trans}$, $145.2_{cis,cis}$, $142.5_{cis,cis}$, $139.2_{cis,trans}$, $73.3_{cis,cis}$, $65.6_{cis,trans}$, $65.1_{cis,trans}$, $64.4_{cis,cis}$, $61.2_{cis,trans}$, $57.0_{cis,cis}$,

41.0$_{cis,cis}$, 38.2$_{cis,trans}$, 37.7$_{cis,cis}$, 34.4$_{cis,cis}$, 33.5$_{cis,cis}$, 28.9$_{cis,trans}$, 28.5$_{cis,trans}$, 27.6$_{cis,trans}$, 25.5$_{cis,cis}$, 23.1$_{cis,trans}$, 16.2$_{cis,trans}$, 15.7$_{cis,cis}$, -0.11$_{cis,cis}$, -0.25$_{cis,trans}$.

HRMS (ESI+): calculated for $C_{15}H_{25}OSi$ $[M+H]^+$ 249.1669, found: 249.1672.

Physical Appearance: pale yellow solid.

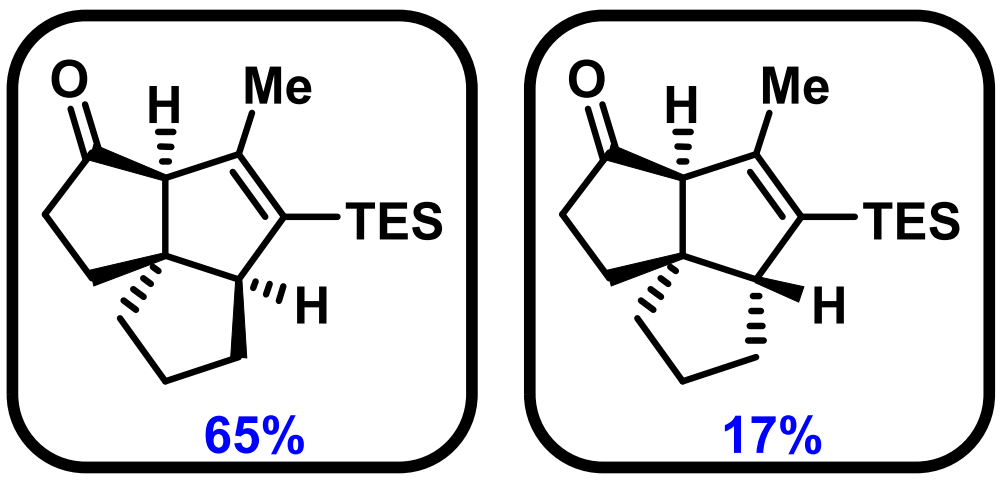

(3aR,5aS,8aS)-4-methyl-5-(trimethylsilyl)-1,2,5a,6,7,8-hexahydrocyclopenta[c] pentalen-3(3aH)-one (28h-*cis*,*trans* and **28h-*cis*,*cis*)**

^{1}H NMR (700 MHz, $CDCl_3$): δ 2.96-2.91 (m, 1H$_{cis,cis}$), 2.84-2.76 (m, 1H$_{cis,trans}$), 2.74 (s, 1H$_{cis,cis}$), 2.70 (s, 1H$_{cis,trans}$), 2.40-2.31 (m, 1H$_{cis,cis}$), 2.30-2.03 (m, 1H$_{cis,cis}$ and 4H$_{cis,trans}$), 2.00-1.91 (m, 1H$_{cis,cis}$), 1.87-1.34 (m, 7H$_{cis,cis}$ and 6H$_{cis,trans}$), 0.96-0.84 (m, 9H$_{cis,cis}$ and 9H$_{cis,trans}$), 0.68-0.59 (m, 6H$_{cis,cis}$ and 6H$_{cis,trans}$).

^{13}C NMR (175 MHz, $CDCl_3$): δ 218.4$_{cis,trans}$, 216.9$_{cis,cis}$, 151.1$_{cis,trans}$, 146.1$_{cis,cis}$, 140.1$_{cis,cis}$, 136.9$_{cis,trans}$, 73.3$_{cis,cis}$, 65.7$_{cis,trans}$, 65.6$_{cis,trans}$, 64.8$_{cis,cis}$, 61.4$_{cis,trans}$, 56.9$_{cis,cis}$, 41.0$_{cis,cis}$, 38.2$_{cis,trans}$, 37.5$_{cis,cis}$, 34.5$_{cis,cis}$, 33.8$_{cis,cis}$, 28.9$_{cis,trans}$, 28.6$_{cis,trans}$, 27.5$_{cis,trans}$, 25.5$_{cis,cis}$, 23.4$_{cis,trans}$, 16.4$_{cis,trans}$, 15.7$_{cis,cis}$, 7.5$_{cis,trans}$, 7.5$_{cis,cis}$, 4.0$_{cis,cis}$, 3.9$_{cis,trans}$.

HRMS (ESI+): calculated for $C_{18}H_{31}OSi$ $[M+H]^+$ 291.2139, found: 291.2146.

Physical Appearance: pale yellow solid.

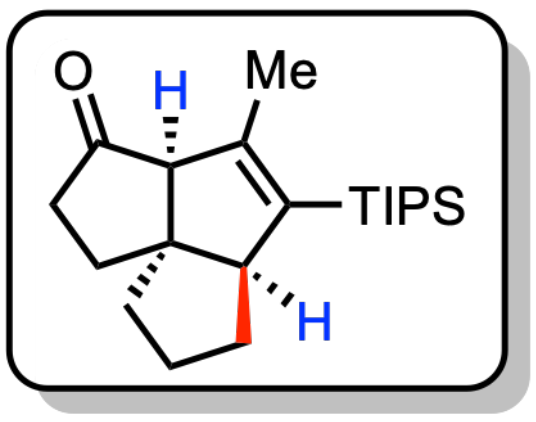

4-methyl-5-(triisopropylsilyl)-1,2,5a,6,7,8-hexahydrocyclopenta[c]pentalen-3(3aH)-one (28i-*cis,trans*)

^{1}H NMR (500 MHz, $CDCl_3$): δ 2.91-2.86 (m, 1H), 2.70 (s, 1H), 2.30-2.04 (m, 4H), 1.89-1.76 (m, 5H), 1.57-1.49 (m, 3H), 1.45-1.39 (m, 1H), 1.30-1.23 (m, 3H), 1.04 (dd, J = 7.8 Hz, 7.8 Hz, 18H).

^{13}C NMR (125 MHz, $CDCl_3$): δ 218.5, 151.9, 135.9, 66.4, 66.1, 61.3, 38.2, 29.0, 28.6, 27.3, 24.0, 18.9, 18.8, 17.5, 12.5.

HRMS (ESI$^+$): calculated for $C_{21}H_{37}OSi$ $[M+H]^+$ 333.2608, found: 333.2601

Physical Appearance: pale yellow solid.

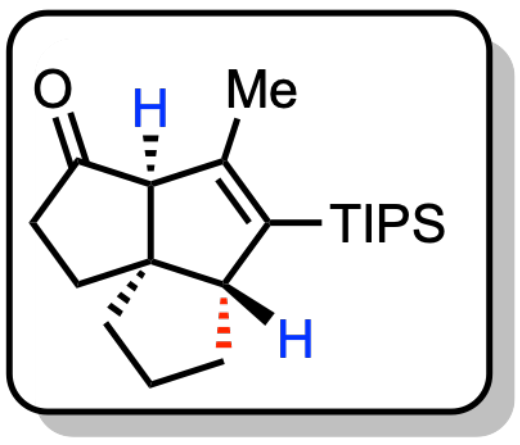

4-methyl-5-(triisopropylsilyl)-1,2,5a,6,7,8-hexahydrocyclopenta[c]pentalen-3(3aH)-one (28i-*cis,cis*)

^{1}H NMR (500 MHz, CDCl3): δ 3.01 (d, J = 9.1 Hz, 1H), 2.77 (s, 1H), 2.39-2.31 (m, 1H), 2.19-2.13 (m, 1H), 2.00-1.95 (m, 1H), 1.90-1.83 (m, 1H), 1.80 (s, 3H), 1.76-1.72 (m, 1H), 1.63-1.56 (m, 2H), 1.52-1.46 (m, 2H), 1.26-1.18 (m, 3H), 1.07 (d, J = 7.6 Hz, 9H), 1.02 (d, J = 7.6 Hz, 9H).

^{13}C NMR (125 MHz, CDCl3): δ 216.9, 147.0, 139.4, 73.4, 65.1, 56.8, 40.7, 37.6, 34.7, 34.6, 25.8, 19.4, 19.1, 16.9, 12.6.

HRMS (ESI$^+$): calculated for $C_{21}H_{37}OSi$ $[M+H]^+$ 333.2608, found: 333.2600.

Physical Appearance: colorless liquid.

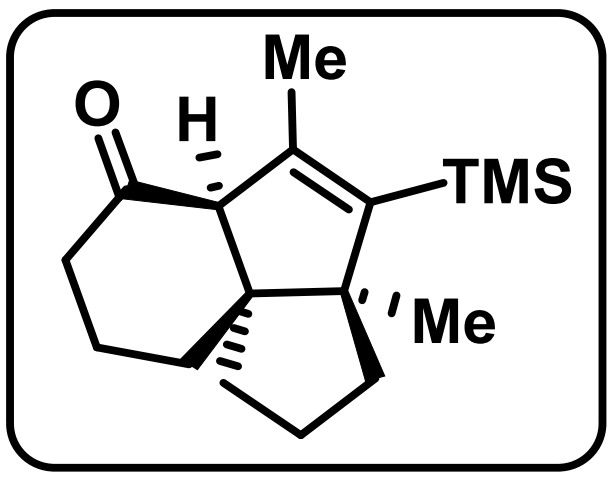

(3aS,5aR,9aS)-3a,5-dimethyl-4-(trimethylsilyl)-2,3,3a,5a,8,9-hexahydro-1H-cyclopenta[c]inden-6(7H)-one (28j-*cis*,*trans*)

^{1}H NMR: (400 MHz, $CDCl_3$): δ 3.07 (s, 1H), 2.29-2.26 (m, 1H), 2.13-2.01 (m, 3H), 1.87-1.83 (m, 1H), 1.69-1.60 (m, 6H), 1.50-1.29 (m, 4H), 0.80 (s, 3H), 0.13 (s, 9H).

^{13}C NMR (100 MHz, $CDCl_3$): δ 214.6, 149.6, 144.2, 67.3, 64.2, 62.5, 40.5, 29.9, 29.0, 26.9, 25.3, 22.9, 22.7, 16.6, 0.0.

HRMS (ESI+): calculated for $C_{17}H_{29}OSi$ $[M+H]^+$ 277.1982, found: 277.1990.

Physical Appearance: Pale yellow liquid.

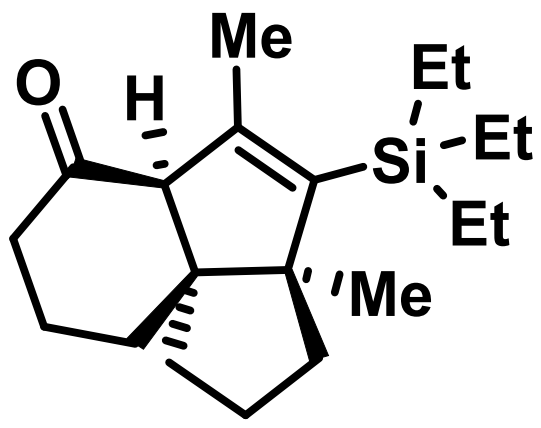

(3aS,5aR,9aS)-3a,5-dimethyl-4-(triethylsilyl)-2,3,3a,5a,8,9-hexahydro-1H-cyclopenta[c]inden-6(7H)-one (28k-*cis*,*trans*)

^{1}H NMR: (400 MHz, $CDCl_3$): δ 3.09 (s, 1H), 2.30-2.25 (m, 1H), 2.13-2.00 (m, 2H), 1.88-1.81 (m, 1H), 1.78-1.71 (m, 1H), 1.68-1.60 (m, 5H), 1.52-1.23 (m, 5H) 0.56 (t, *J* = 7.7 Hz, 9H), 0.80 (s, 3H), 0.66 (q, *J* = 7.9 Hz, 6H).

^{13}C NMR (100 MHz, $CDCl_3$): δ 214.6, 150.6, 142.2, 67.1, 64.4, 62.5, 40.5, 29.9, 29.3, 26.9, 25.5, 22.9, 22.8, 16.8, 7.6, 4.0.

HRMS (ESI+): calculated for $C_{20}H_{35}OSi$ $[M+H]^+$ 319.2452, found: 319.2457.

Physical Appearance: white solid.

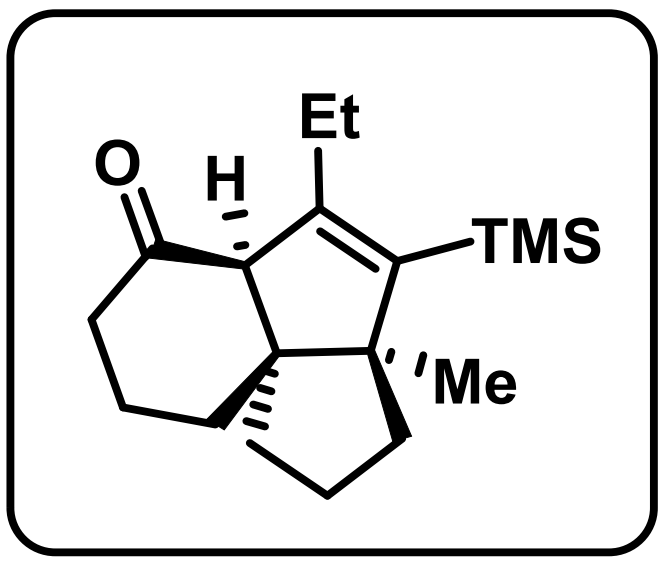

(3aS,5aR,9aS)-5-ethyl-3a-methyl-4-(trimethylsilyl)-2,3,3a,5a,8,9-hexahydro-1H-cyclopenta[c]inden-6(7H)-one (28l-*cis*,*trans*)

^{1}H NMR: (400 MHz, $CDCl_3$): δ 3.23 (s, 1H), 2.34-2.24 (m, 2H), 2.20-2.11 (m, 1H), 2.07-2.01 (m, 2H), 1.88-1.83 (m, 1H), 1.72-1.63 (m, 4H), 0.91 (t, *J* = 7.4 Hz, 3H), 0.80 (s, 3H), 0.13 (s, 9H).

^{13}C NMR (100 MHz, $CDCl_3$): δ 214.6 156.6, 143.6, 67.1, 64.3, 60.0, 40.7, 29.9, 28.9, 26.9, 25.4, 25.0, 23.1, 23.0, 13.4, 0.3.

HRMS (ESI+): calculated for $C_{18}H_{30}OSi$ $[M+H]^+$ 291.2066, found: 291.214.

Physical Appearance: white crystalline solid.

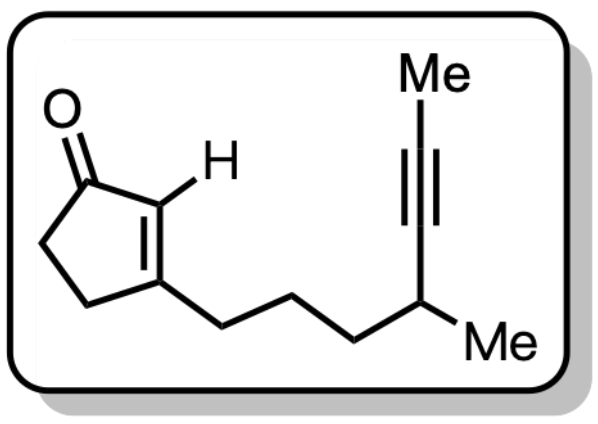

3-(4-methylhept-5-yn-1-yl)cyclopent-2-en-1-one (29a)

^{1}H NMR (400 MHz, $CDCl_3$): δ 5.96-5.94 (m, 1H), 2.58-2.56 (m, 2H), 2.42-2.38 (m, 5H), 1.82-1.59 (m, 2H), 1.77 (d, *J* = 2.3 Hz, 3H), 1.43-1.27 (m, 2H), 1.12 (d, *J* = 6.8 Hz, 3H).

^{13}C NMR (100 MHz, $CDCl_3$): δ 210.0, 182.8, 129.5, 83.1, 75.9, 36.6, 35.2, 33.2, 31.4, 25.7, 24.8, 21.3, 3.3.

HRMS (ESI^+): calculated for $C_{13}H_{19}O$ $[M+H]^+$ 191.1430, found: 191.1426.

Physical Appearance: pale yellow oil.

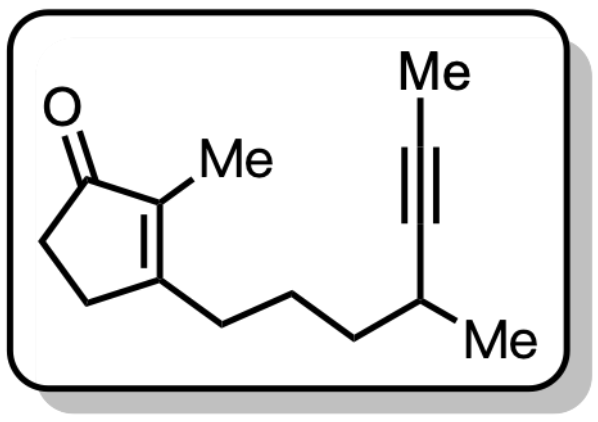

2-methyl-3-(4-methylhept-5-yn-1-yl)cyclopent-2-en-1-one (29b)

^{1}H NMR (400 MHz, $CDCl_3$): δ 2.50-2.47 (m, 2H), 2.43-2.34 (m, 5H), 1.77 (d, *J* = 2.5 Hz, 3H), 1.74-1.55 (m, 2H), 1.67 (t, *J* = 2.1 Hz, 3H), 1.40-1.34 (m, 2H), 1.11 (d, *J* = 6.7 Hz, 3H).

^{13}C NMR (100 MHz, $CDCl_3$): δ 210.1, 173.6, 136.1, 83.1, 75.9, 36.8, 34.0, 30.8, 29.1, 25.7, 24.9, 21.3, 7.9, 3.3.

HRMS (ESI^+): calculated for $C_{14}H_{21}O$ $[M+H]^+$ 205.1587, found: 205.1582.

Physical Appearance: pale yellow oil.

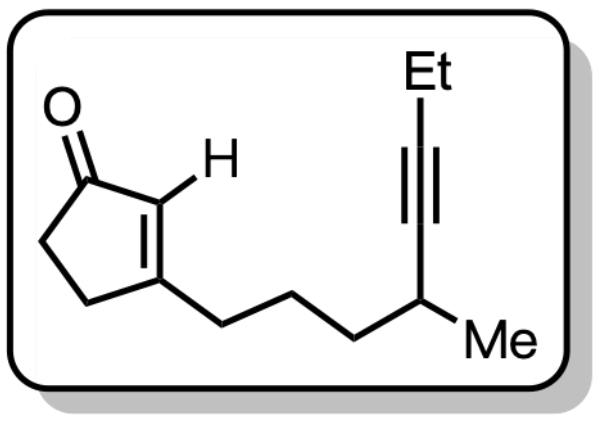

3-(4-methyloct-5-yn-1-yl)cyclopent-2-en-1-one (29c)

^{1}H NMR (400 MHz, $CDCl_3$): δ 5.93-5.92 (m, 1H), 2.56-2.54 (m, 2H), 2.41-2.36 (m, 5H), 2.12 (qd, *J* = 7.4, 2.3 Hz, 2H), 1.80-1.54 (m, 3H), 1.43-1.35 (m, 2H), 1.11-1.05 (m, 6H).

^{13}C NMR (100 MHz, $CDCl_3$): δ 210.2, 182.9, 129.6, 83.4, 82.3, 36.7, 35.3, 33.3, 31.5, 25.7, 24.9, 21.5, 14.4, 12.4.

HRMS (ESI^+): calculated for $C_{14}H_{21}O$ $[M+H]^+$ 205.1587, found: 205.1588.

Physical Appearance: yellow oil.

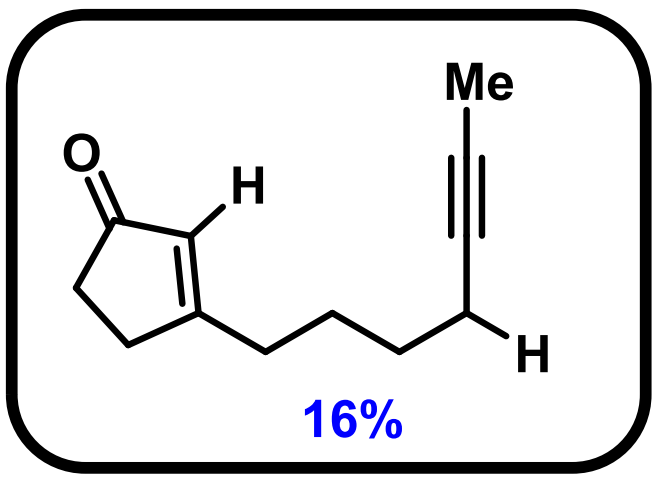

3-(hept-5-yn-1-yl)cyclopent-2-en-1-one (29d)

^{1}H NMR (400 MHz, $CDCl_3$): δ 5.95-5.93 (m, 1H), 2.57-2.55 (m, 2H), 2.42-2.37 (m, 4H), 2.18-2.12 (m, 2H), 1.75 (t, J = 2.5 Hz, 3H), 1.71-1.63 (m, 2H), 1.54-1.46 (m, 2H).

^{13}C NMR (100 MHz, $CDCl_3$): δ 210.0, 182.7, 129.5, 78.4, 76.0, 35.2, 33.0, 31.4, 28.5, 26.1, 18.4, 3.4.

HRMS (ESI^+): calculated for $C_{12}H_{17}O$ $[M+H]^+$ 177.1274, found: 177.1274.

Physical Appearance: pale yellow solid.

Table 1. Optimization of solvents and temperature.

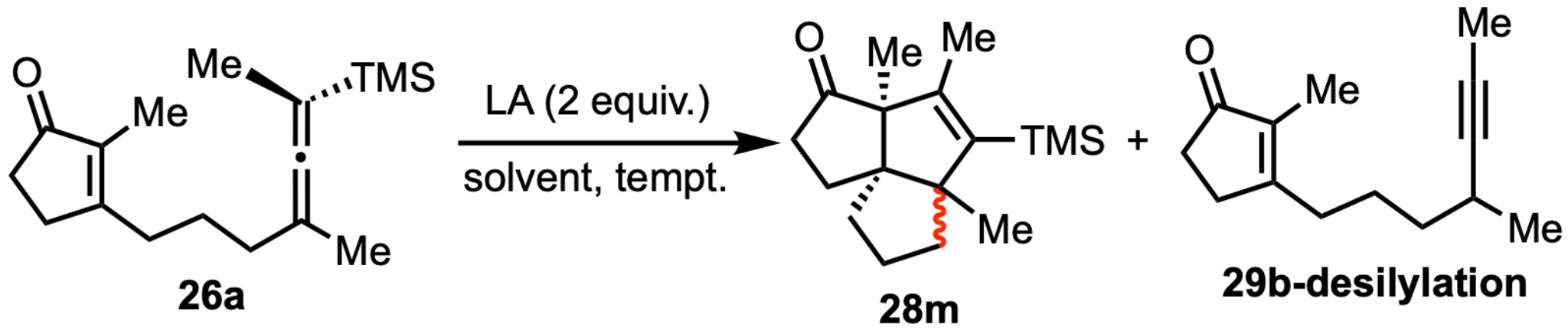

entry	Lewis acid	Solvent conc. (M)	tempt. (°C)	**28m** (%)	**29b-desilylation** (%)	Recovery (%)
1	$TiCl_4$	CH_2Cl_2 (0.02)	40	10	67	-
2	$TiCl_4$	CH_2Cl_2 (0.01)	-78	0	~100	-
3	$Ti(OiPr)_4$	CH_2Cl_2 (0.05)	-78 to RT	0	ND	~100
4	$Ti(OiPr)_4$	DCE (0.05)	90	0	ND	98
5	$Ti(OiPr)_4$	PhMe (0.1)	110	0	ND	~100
6	BF_3-Et_2O	CH_2Cl_2 (0.05)	-78 to RT	0	~100	-
7	$TiCl_2(OiPr)_2$	DCE (0.1)	RT to 90	0	18	67
8	Me_2AlCl	DCE (0.1)	RT to 90	0	5	67

Table 2. Screening of Lewis acids.

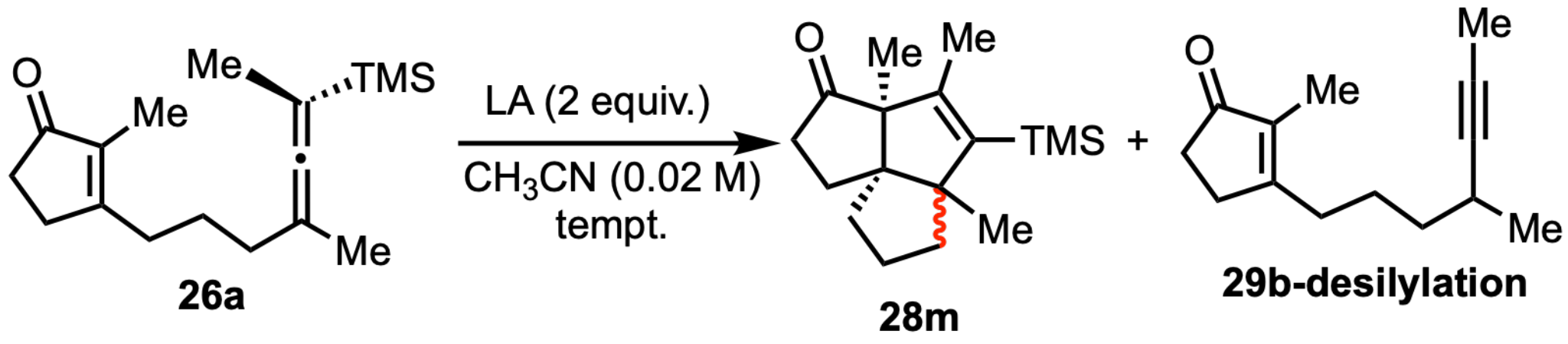

entry	Lewis acid	tempt. (°C)	**28m** (%)	**29b-desilylation** (%)	Recovery (%)
1[a]	$TiCl_4$	40	10	67	-
2	$InCl_3$	RT to 85	0	49	21
3	$InCl_3$ + 5 mol% TMSCl	RT	0	66	-
4	$Yb(OTf)_3$	RT to 85	0	32	28
5	$La(OTf)_3$	RT to 85	0	-	72
6	$EtAlCl_2$	RT	0	45	54
7	Me_2AlCl	RT to 85	0	34	42
8	TfOH	RT	0	48	-

[a] The reaction was carried out in 0.05 M CH_2Cl_2.

Table 3. Screening of solvents.

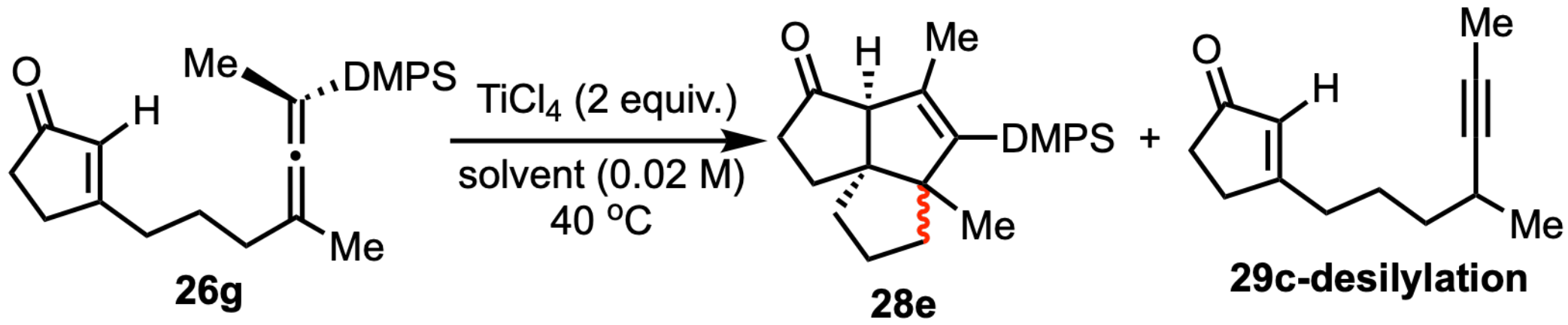

entry	solvent	**28e** (%)	**29c-desilylation** (%)	*cis,cis*/*cis,trans*
1	CH_2Cl_2	71	15	1/11
2	DCE	39	37	>1/15
3	PhF	23	-	1/7
4	PhCl	31	65	1/5
5	PhBr	34	64	1/5
6	$PhCF_3$	46	51	1/8

Optimization of the Lewis acid-mediated cyclization was initially examined by screening solvents and temperatures using $TiCl_4$ (Table 1). When the reaction was conducted in CH_2Cl_2 (0.02 M) at 40 °C, the desired product **28m** was obtained in low yield (10%), accompanied predominantly by desilylation side product **29b** (67%). Lowering the temperature to −78 °C completely suppressed the desired transformation, resulting in quantitative recovery of starting material. Alternative titanium-based Lewis acids, including $Ti(OiPr)_4$ and $TiCl_2(OiPr)_2$, as well as $BF_3{\cdot}Et_2O$ and Me_2AlCl, were also ineffective, giving either no desired product or significant recovery of starting material. These results indicated that $TiCl_4$ was uniquely capable of promoting the cyclization, although competing desilylation remained a major issue.

Subsequently, a variety of Lewis acids were evaluated under modified conditions (Table 2). Among those tested, $InCl_3$, $Yb(OTf)_3$, $La(OTf)_3$, $EtAlCl_2$, Me_2AlCl, and

TfOH failed to afford the desired cyclized product, instead leading predominantly to desilylation or recovery of starting material. The addition of catalytic TMSCl to $InCl_3$ did not improve the outcome. Overall, $TiCl_4$ remained the only Lewis acid capable of promoting productive cyclization, albeit in modest yield.

For the solvent screening studies (Table 3), substrate **26g** was selected to examine whether its aryl substituent could interact with aromatic solvents and thereby influence the stereochemical outcome of the cyclization. Accordingly, solvent effects were evaluated using **26g**, whereas the initial Lewis acid and temperature optimization studies were performed using the model substrate **26a**. Among the solvents examined, CH_2Cl_2 provided the best balance of reactivity and selectivity, affording product **28e** in 71% yield with minimal desilylation (15%) and a *cis,cis*/*cis,trans* ratio of 1/11. In contrast, DCE resulted in increased desilylation (37%) despite moderate formation of the desired product. Aromatic solvents such as PhCl and PhBr led to substantial desilylation (64–65%), while PhF and $PhCF_3$ showed diminished reactivity. Collectively, these studies established $TiCl_4$ in CH_2Cl_2 as the optimal conditions for the cyclization, providing the desired product in good yield and high diastereoselectivity.

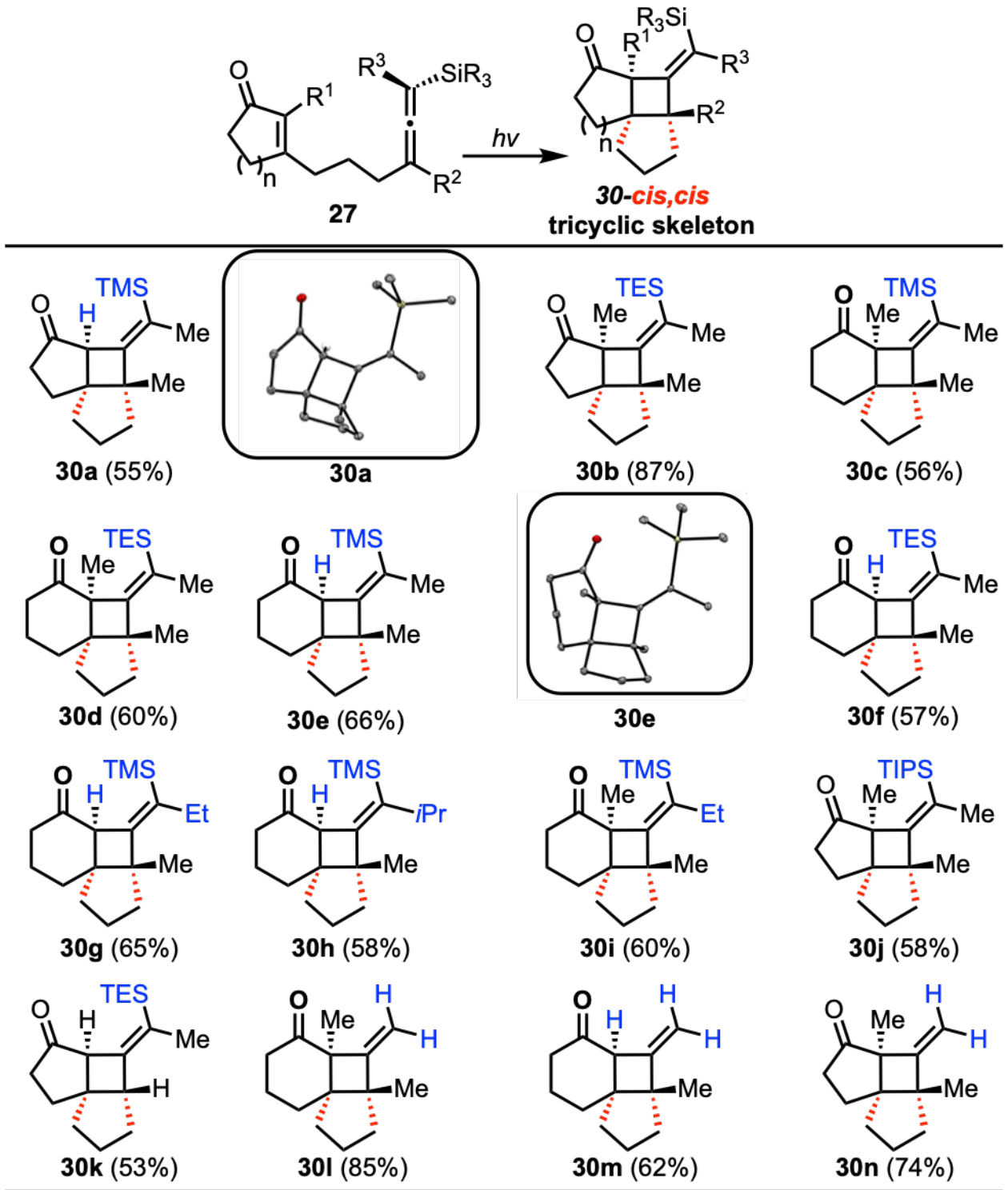

a) Reaction conditions: To SM (1 equiv., 0.5 mmol) in (0.05 M PhMe) was irradiated with UV (365 nm). The reaction was stirred at room temperature until the consuption of SM monitoring with TLC. b) The reaction was carried out up to 2.5 mmol (SM) scale.

General procedure for [2+2] cycloaddition

The starting material **27** (200 mg, 1.05 mmol) was dissolved in PhMe (21 mL) in a vial under a nitrogen atmosphere. The vial was placed in a Penn PhD M2 photoreactor and irradiated (365 nm) until complete consumption of the starting material monitored by TLC. The reaction mixture was concentrated under reduced pressure to afford the crude product. Purification by flash column chromatography (EtOAc/hexanes = 1:12) afforded the corresponding product **30n** (147.2 mg, 74%, CHL-I-180).

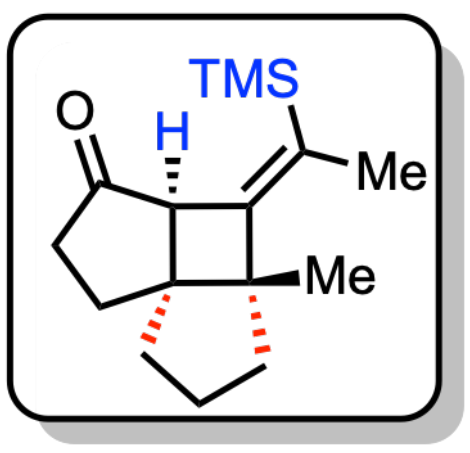

(3a*R*,4a*R*,7a*S*,*E*)-4a-methyl-4-(1-(trimethylsilyl)ethylidene)octahydro-3*H*-cyclobuta[1,2:1,4]di[5]annulen-3-one (30a)

¹H NMR (400 MHz, $CDCl_3$): δ 2.98 (s, 1H), 2.62-2.54 (m, 1H), 2.32-2.24 (m, 1H), 2.07-2.01 (m, 2H), 1.82-1.67 (m, 4H), 1.65 (s, 3H), 1.63-1.59 (s, 1H), 1.54-1.47 (m, 1H), 1.32 (s, 3H), 0.10 (s, 9H).

¹³C NMR (100 MHz, $CDCl_3$): δ 217.0, 148.6, 131.2, 58.8, 55.8, 51.7, 40.3, 39.1, 38.1, 26.5, 25.4, 20.5, 17.0, -1.03.

HRMS (ESI+): calculated for $C_{16}H_{27}OSi$ $[M+H]^+$ 263.1826, found: 263.1831.

Physical Appearance: colorless oil.

gram-scale [2+2] cycloaddition (30a, 59% yield)

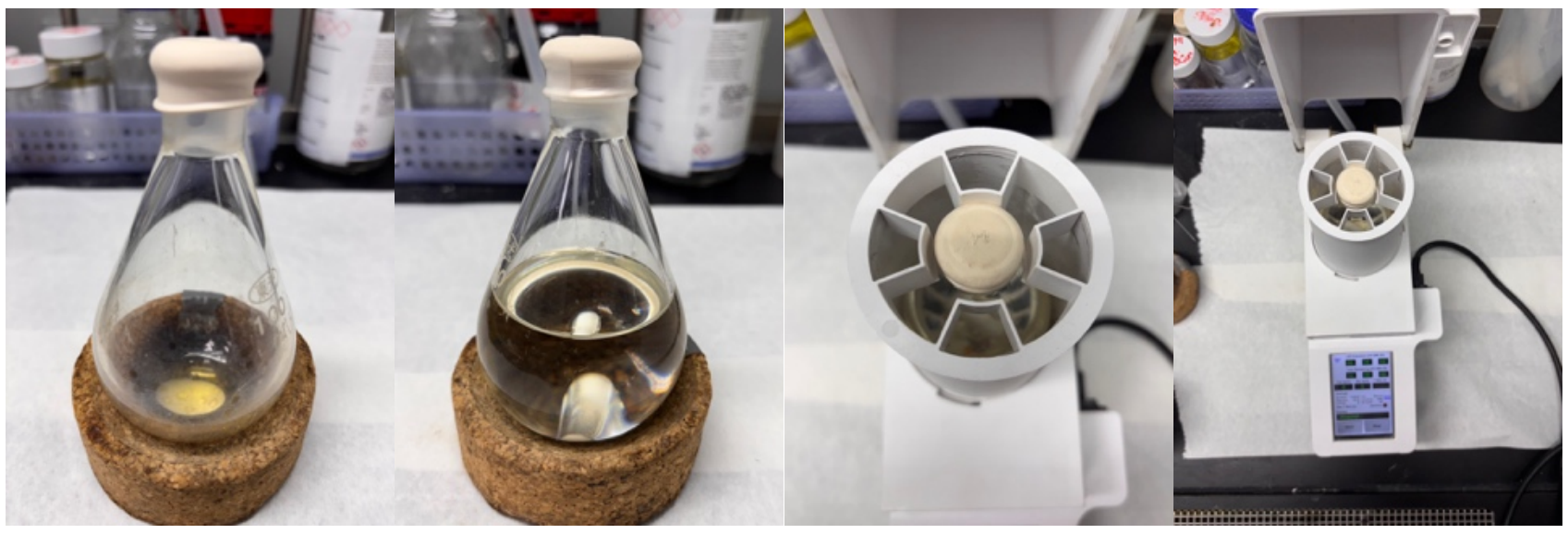

1 g of allene **27a** 1 g of **27a** in toluene (73 mL) [2+2] cycloaddition, Penn PhD Photoreactor M2 (365 nm)

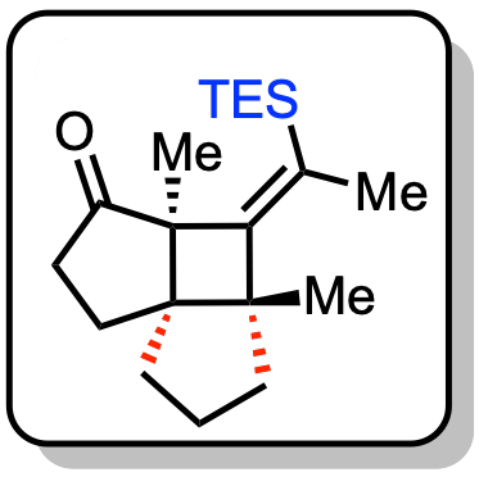

(3a*R*,4a*R*,7a*R*,*Z*)-3a,4a-dimethyl-4-(1-(triethylsilyl)ethylidene)octahydro-3*H*-cyclobuta[1,2:1,4]di[5]annulen-3-one (30b)

¹H NMR (400 MHz, $CDCl_3$): δ 2.67-2.57 (m, 1H), 2.26-2.13 (m, 2H), 2.01-1.96 (m, 1H), 1.83-1.79 (m, 1H), 1.69 (s, 3H), 1.66-1.58 (m, 3H), 1.46-1.37 (m, 2H), 1.34 (s, 3H), 1.14 (s, 3H), 0.86 (t, J = 7.5, 2H), 0.79-0.70 (m, 3H), 0.68-0.60 (m, 3H).

^{13}C NMR (100 MHz, $CDCl_3$): δ 218.5, 157.2, 128.5, 58.8, 55.0, 54.3, 40.3, 38.2, 34.8, 25.8, 25.7, 21.1, 19.1, 15.0, 7.6, 3.6.

HRMS (ESI+): calculated for $C_{20}H_{35}OSi$ $[M+H]^+$ 319.2452, found: 319.2455.

Physical Appearance: colorless oil.

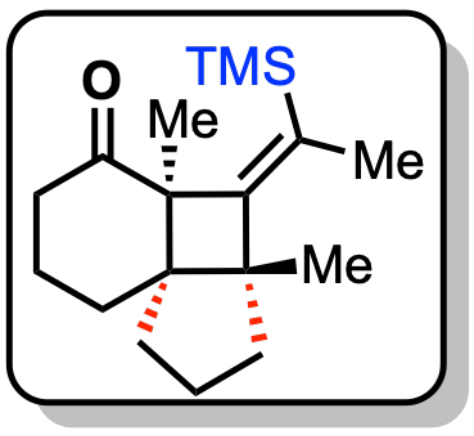

(3aR,4aR,8aR,E)-3a,4a-dimethyl-4-(1-(trimethylsilyl)ethylidene)octahydrocyclopenta[1,4]cyclobuta[1,2]benzen-5(6H)-one (30c)

^{1}H NMR: (400 MHz, $CDCl_3$): δ 2.42-2.36 (m, 1H), 2.32-2.26 (m, 1H), 2.19-2.14 (m, 1H), 2.08-2.14 (m, 1H), 1.94-1.80 (m, 2H), 1.70-1.66 (m, 3H), 1.64-1.55 (m, 2H), 1.54-1.38 (m, 2H), 1.30-1.19 (m, 5H), 1.18-1.15 (m, 3H), 0.08 (s, 9H).

^{13}C NMR (100 MHz, $CDCl_3$): δ 214.6, 155.7, 130.7, 57.9, 56.2, 52.7, 38.9, 37.6, 37.5, 30.6, 25.4, 22.2, 20.1, 18.2, 17.9, 0.7.

HRMS (ESI+): calculated for $C_{18}H_{31}OSi$ $[M+H]^+$ 291.2139, found:291.2146.

Physical Appearance: colorless liquid.

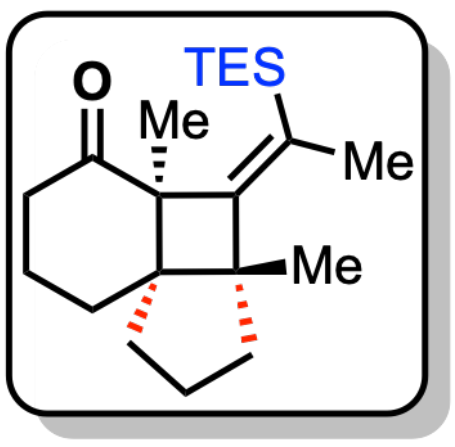

(3aR,4aR,8aR,E)-3a,4a-dimethyl-4-(1-(triethylsilyl)ethylidene)octahydrocyclopenta[1,4]cyclobuta[1,2]benzen-5(6H)-one (30d)

^{1}**H NMR:** (400 MHz, $CDCl_3$): δ 2.43-2.30 (m, 2H), 2.18-2.08(m, 2H), 1.93-1.86 (m, 2H), 1.70 (s, 3H), 1.66-1.60 (m, 2H), 1.54-1.45 (m, 2H), 1.29-1.23 (m, 2H), 1.22 (s, 3H), 1.21 (s, 3H), 0.90 (t, 9H), 0.69-0.52 (m, 6H).

^{13}**C NMR** (100 MHz, $CDCl_3$): δ 214.2, 157.4, 128.6, 58.2, 56.5, 52.9, 39.0, 37.5, 37.4, 30.3, 25.4, 22.2, 20.3, 18.5, 17.8, 7.8, 3.9.

HRMS (ESI+): calculated for $C_{21}H_{37}OSi$ $[M+H]^+$ 333.2608, found: 333.2612.

Physical Appearance: colorless liquid.

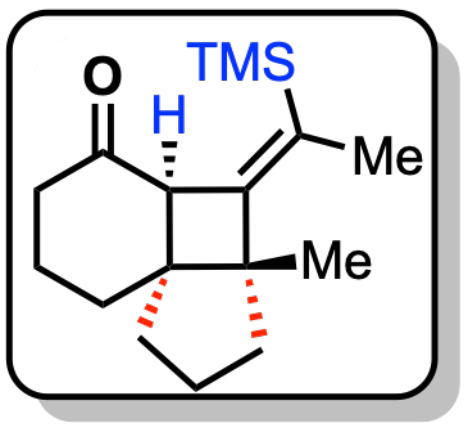

(3aR,4aR,8aS,Z)-3a-methyl-4-(1-(trimethylsilyl)ethylidene)octahydrocyclopenta[1,4] cyclobuta[1,2]benzen-5(6H)-one (30e)

^{1}**H NMR:** (400 MHz, $CDCl_3$): δ 3.0 (s, 1H), 2.38-2.19 (m, 2H), 2.00-1.89 (m, 4H), 1.74-1.57 (m, 7H), 1.49-1.38 (m, 2H), 1.20 (s, 3H), 0.05 (s, 9H).

^{13}**C NMR** (100 MHz, $CDCl_3$): δ 211.4, 149.7, 131.5, 58.6, 57.0, 49.9, 42.1, 39.7, 39.5, 29.6, 24.2, 22.2, 19.7, 16.7, -0.9.

HRMS (ESI+): calculated for $C_{17}H_{29}OSi$ $[M+H]^+$ 277.1982, found: 277.1982.

Physical Appearance: colorless liquid.

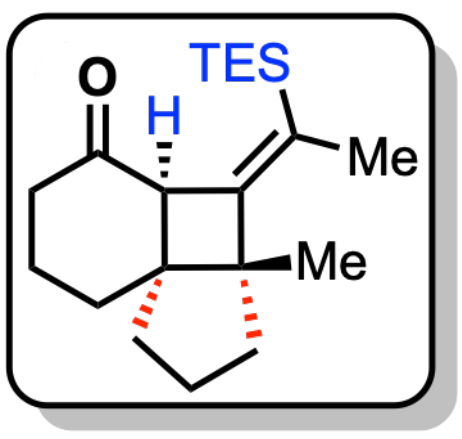

(3aR,4aR,8aS,Z)-3a-methyl-4-(1-(triethylsilyl)ethylidene)octahydrocyclopenta[1,4]cyclobuta[1,2]benzen-5(6H)-

one (30f)

^{1}H NMR: (400 MHz, $CDCl_3$): δ 3.04 (s, 1H), 2.40-2.34 (m, 1H), 2.28-2.22 (m, 1H), 2.03-1.91 (m, 4H), 1.69-1.63 (m, 5H), 1.48-1.39 (m, 2H), 1.31-1.26 (m, 2H), 1.23 (s, 3H) 0.94-0.87 (m, 9H), 0.66-0.56 (m, 6H).

^{13}C NMR (100 MHz, $CDCl_3$): δ 211.5, 151.0, 129.7, 59.2, 57.1, 49.9, 42.1, 39.8, 39.3, 29.4, 24.3, 22.2, 19.8, 17.5, 7.7, 3.0.

HRMS (ESI+): calculated for $C_{20}H_{35}OSi$ $[M+H]^+$ 319.2452, found: 319.2455.

Physical Appearance: colorless liquid.

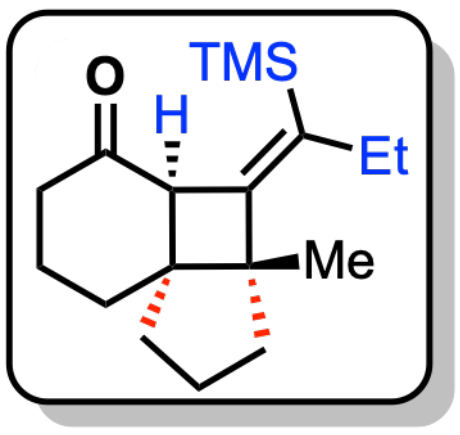

(3aR,4aR,8aS,Z)-3a-methyl-4-(1-(trimethylsilyl)propylidene)octahydrocyclopenta[1,4]cyclobuta[1,2]benzen-5(6H)-one (30g)

^{1}H NMR: (400 MHz, $CDCl_3$): δ 3.39 (s, 1H), 2.56-2.47 (m, 1H), 2.23-2.14 (m, 2H), 2.03-1.97 (m, 2H), 1.85-1.79 (m, 2H), 1.72-1.64 (m, 3H), 1.48-1.38(m, 4H), 1.28 (s, 3H), 0.80 (t, J = 7.5 Hz, 3H), 0.14 (s, 9H).

^{13}C NMR (100 MHz, $CDCl_3$): δ 212.8, 151.5, 136.9, 59.8, 54.9, 48.7, 42.8, 40.5, 38.6, 28.8, 25.7, 24.0, 22.2, 20.6, 14.9, 1.2.

HRMS (ESI+): calculated for $C_{18}H_{31}OSi$ $[M+H]^+$ 291.2139, found: 291.2141.

Physical Appearance: colorless liquid.

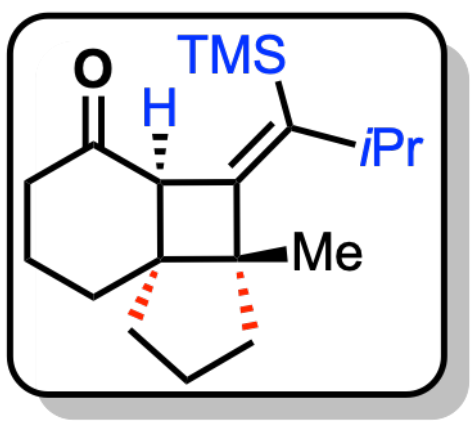

(3aR,4aR,8aS,Z)-3a-methyl-4-(2-methyl-1-

(trimethylsilyl)propylidene)octahydrocyclopenta[1,4]cyclobuta[1,2]benzen-5(6H)-one (30h)

^{1}H NMR: (400 MHz, $CDCl_3$): δ 3.38 (s, 1H), 2.73-2.66 (m, 1H), 2.50-2.43 (m, 1H), 2.27-2.18 (m, 1H), 2.12-2.02 (m, 2H), 1.90-1.83 (m, 1H), 1.77-1.63 (m, 4H), 1.46-1.36 (m, 3H), 1.27 (s, 3H), 1.02 (d, J = 7.0 Hz, 3H), 0.95 (d, J = 7.2 Hz, 3H), 0.20 (s, 9H).

^{13}C NMR (100 MHz, $CDCl_3$): δ 212.9, 150.9, 141.8, 60.3, 55.5, 49.7, 42.7, 40.8, 38.3, 33.8, 29.7, 28.8, 24.1, 22.1, 21.9, 21.1, 3.5.

HRMS (ESI+): calculated for $C_{19}H_{33}OSi$ $[M+H]^+$ 305.2295, found: 305.2298.

Physical Appearance: colorless liquid.

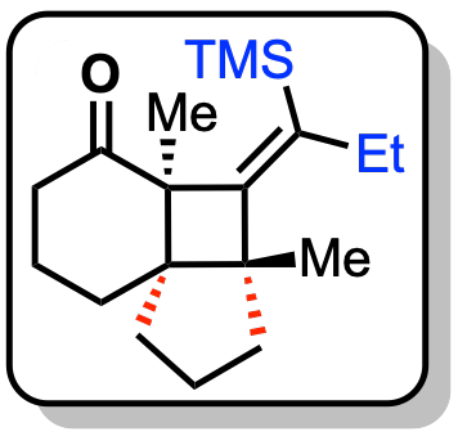

(3aR,4aR,8aR,E)-3a,4a-dimethyl-4-(1-(trimethylsilyl)propylidene)octahydrocyclopenta[1,4]cyclobuta[1,2]benzen-5(6H)-one (30i)

^{1}H NMR: (400 MHz, $CDCl_3$): δ 2.45-2.10 (m, 5H), 2.01-1.96 (m, 1H) 1.93-1.86 (m, 2H), 1.66-1.56 (m, 2H), 1.54-1.45 (m, 1H), 1.42-1.34 (m, 1H), 1.30-1.23 (m, 5H) 1.21-1.19 (m, 3H), 0.93 (t, J = 7.44 Hz, 3H), 0.09 (s, 9H).

^{13}C NMR (100 MHz, $CDCl_3$): δ 214.4, 156.3, 137.8, 58.4, 56.9, 53.1, 40.1, 37.4, 37.3, 30.4, 25.7, 25.3, 22.3, 21.0, 17.8, 15.4, 1.8.

HRMS (ESI+): calculated for $C_{19}H_{33}OSi$ $[M+H]^+$ 305.2295, found: 305.2302.

Physical Appearance: colorless liquid.

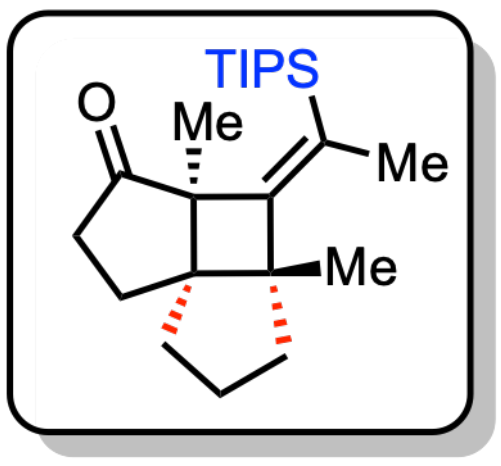

(3a*R*,4a*R*,7a*R*,*Z*)-3a,4a-dimethyl-4-(1-(triisopropylsilyl)ethylidene)octahydro-3*H*-cyclobuta[1,2:1,4]di[5]annulen-3-one (30j)

^{1}H NMR: (400 MHz, $CDCl_3$): δ 2.69-2.59 (m, 1H), 2.25-2.16 (m, 2H), 2.03-1.97 (m, 1H), 1.85-1.80 (m, 1H), 1.75 (s, 3H), 1.71-1.65 (m, 2H), 1.63-1.59 (m, 1H), 1.45-1.41 (m, 3H), 1.38 (s, 3H), 1.28-1.19 (m, 2H), 1.16 (s, 3H), 1.06 (d, J = 7.6Hz, 9H), 1.00 (d, J = 7.4Hz, 9H).

^{13}C NMR (100 MHz, $CDCl_3$): δ 217.5, 157.8, 129.4, 58.7, 55.5, 54.1, 40.4, 38.4, 35.0, 25.9, 25.8, 21.4, 20.1, 19.6, 19.4, 15.4, 12.1.

HRMS (ESI+): calculated for $C_{23}H_{41}OSi$ $[M+H]^+$ 361.2921, found: 361.2932.

Physical Appearance: colorless liquid.

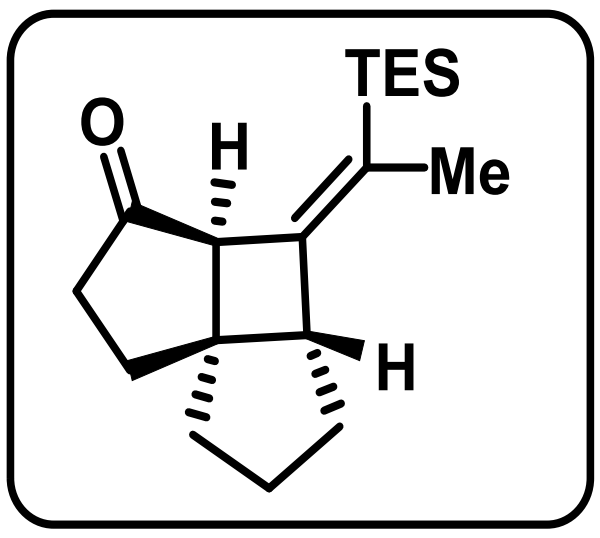

(3aR,4aR,7aS,Z)-4a-methyl-4-(1-(triethylsilyl)ethylidene)octahydro-3H-cyclobuta[1,2:1,4]di[5]annulen-3-one (30k)

^{1}H NMR: (400 MHz, $CDCl_3$): δ 3.2-3.20(m, 1H), 2.91-2.90 (m, 1H), 2.73-2.63 (m, 1H), 2.29-2.22 (m, 1H), 1.96-1.83 (m, 3H), 1.80-1.71 (m, 2H), 1.68-1.62 (m, 2H), 1.59-1.58 (m, 3H), 1.53-1.45 (m, 1H), 0.87 (t, J = 7.9Hz, 9H), 0.49 (q, J = 7.9Hz, 6H).

^{13}C NMR (100 MHz, $CDCl_3$): δ 215.8, 144.2, 129.9, 59.4, 52.1, 49.5, 38.3, 37.3, 32.0, 31.4, 26.1, 17.5, 7.4, 2.7.

HRMS (ESI+): calculated for $C_{18}H_{31}OSi$ $[M+H]^+$ 291.2144, found: 291.2143.

Physical Appearance: colorless liquid.

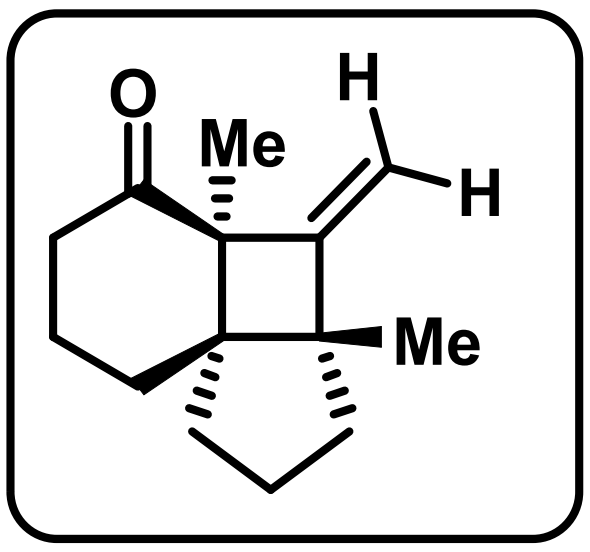

(3aS,4aS,8aR)-3a,4a-dimethyl-4-methyleneoctahydrocyclopenta[1,4]cyclobuta[1,2]benzen-5(6H)-one (30l)

^{1}H NMR: (400 MHz, $CDCl_3$): δ 5.01-5.00 (m, 1H), 4.82-4.81 (m, 1H), 2.37-2.25 (m, 2H), 1.95-1.81 (m, 2H), 1.74-1.50 (m, 6H), 1.39-1.26 (m, 2H), 1.16-1.13 (m, 3H), 1.11-1.10 (m, 3H).

^{13}C NMR (100 MHz, $CDCl_3$): δ 214.6, 160.5, 105.1, 56.7, 54.9, 53.1, 40.6, 38.3, 37.9, 30.7, 24.9, 22.2, 20.2, 18.2.

HRMS (ESI+): calculated for $C_{14}H_{21}O$ $[M+H]^+$ 205.1587, found: 205.1589.

Physical Appearance: colorless liquid.

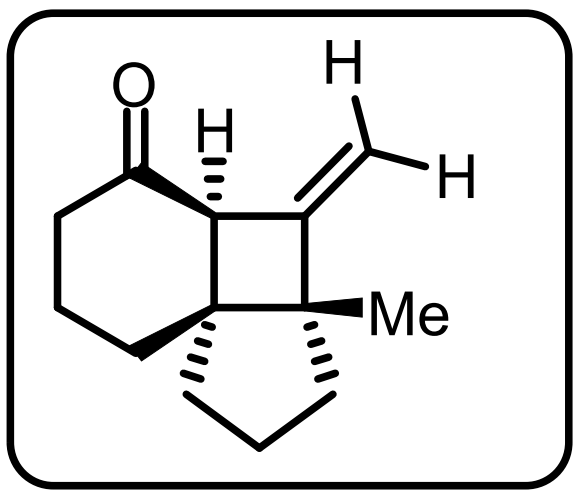

(3aS,4aS,8aS)-3a-methyl-4-methyleneoctahydrocyclopenta[1,4]cyclobuta[1,2]benzen-5(6H)-one (30m)

^{1}H NMR: (400 MHz, $CDCl_3$): δ 4.90-4.89 (m, 1H), 4.80-4.79 (1H), 3.12-3.10 (m, 1H), 2.45-2.38 (m, 1H), 2.15-2.07 (m, 1H), 1.98-1.88 (m, 2H), 1.87-1.77 (m, 4H), 1.58-1.46 (m, 4H), 1.15 (s, 3H).

^{13}C NMR (100 MHz, $CDCl_3$): δ 209.9, 153.4, 104.8, 55.9, 54.0, 48.8, 42.3, 40.9, 39.6, 28.7, 23.8, 20.6.

HRMS (ESI+): calculated for $C_{13}H_{19}O$ $[M+H]^+$ 191.1430, found: 191.1435.

Physical Appearance: colorless liquid.

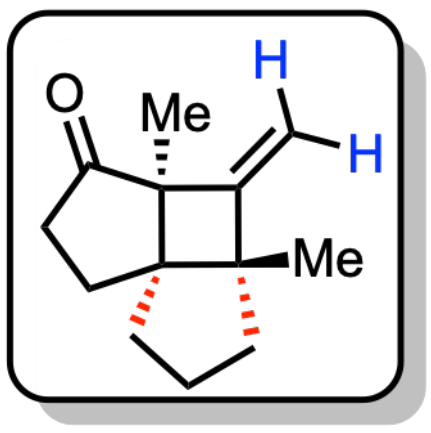

3a,4a-dimethyl-4-methyleneoctahydro-3H-cyclobuta[1,2:1,4]di[5]annulen-3-one (30n)

^{1}H NMR (400 MHz, $CDCl_3$): δ 4.86 (d, *J* = 1.0, 1H), 4.76 (d, *J* = 1.0, 1H), 2.67-2.57 (m, 1H), 2.39-2.31 (m, 1H), 2.04-1.98 (m, 1H), 1.87-1.74 (m, 3H), 1.72-1.67 (m, 2H), 1.54-1.38 (m, 2H), 1.19 (s, 3H), 1.10 (s. 3H).

^{13}C NMR (100 MHz, $CDCl_3$): δ 220.5, 159.3, 104.2, 58.3, 56.5, 53.8, 42.1, 38.0, 34.5, 26.0, 25.5, 20.7, 13.5.

HRMS (ESI+): calculated for $C_{13}H_{19}O$ $[M+H]^+$ 191.1430, found: 191.1431.

Physical Appearance: pale yellow oil.

Investigation of an allene precursor designed to generate a six-membered bottom ring.

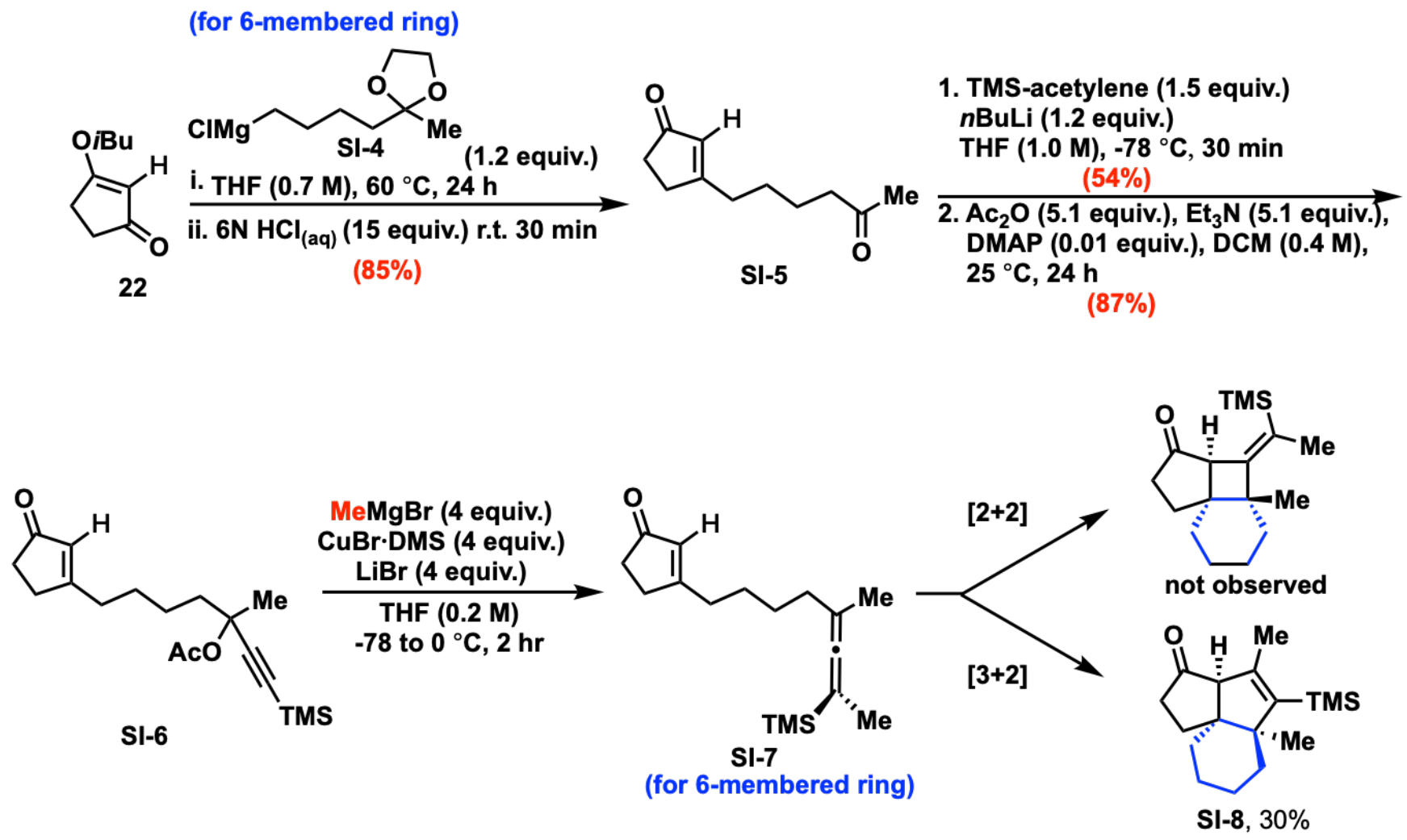

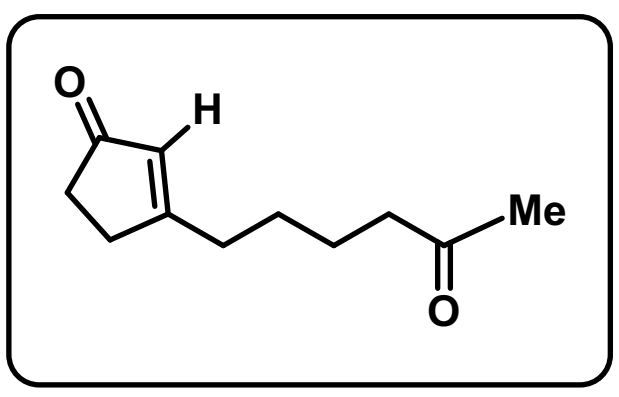

3-(5-oxohexyl)cyclopent-2-en-1-one (SI-5)

^{1}H NMR: (400 MHz, $CDCl_3$): δ 5.89 (s, 1H), 2.54-2.52 (m, 2H), 2.43 (t, J = 6.3 Hz, 2H), 2.39-2.34 (m, 4H), 2.10 (s, 3H), 1.58-1.52 (m, 4H).

^{13}C NMR (100 MHz, $CDCl_3$): δ 210.0, 208.4, 182.4, 129.5, 43.1, 35.2, 33.3, 31.4, 29.9, 26.5, 23.2.

HRMS (ESI+): calculated for $C_{11}H_{17}O_2$ $[M+H]^+$ 181.1223, found: 181.1222.

Physical Appearance: Pale yellow liquid.

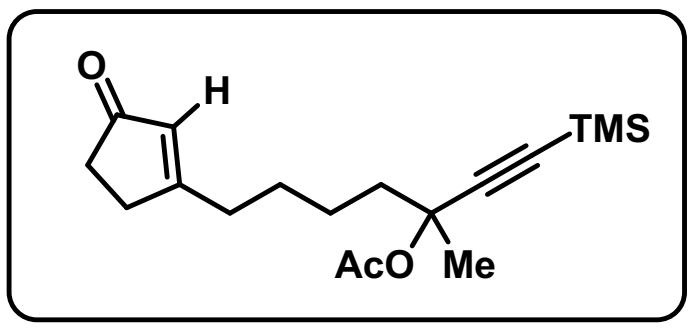

3-methyl-7-(3-oxocyclopent-1-en-1-yl)-1-(trimethylsilyl)hept-1-yn-3-yl acetate (SI-6)

^{1}H NMR: (400 MHz, $CDCl_3$): δ 5.93-5.92 (m,1H), 2.56-2.54 (m, 2H), 2.43-2.36 (m, 4H), 1.98 (s, 3H), 1.96-1.92 (m, 1H), 1.82-1.74 (m, 1H), 1.62 (s, 3H), m, 1.59-1.57(m 2H), 1.54-1.45 (m, 2H), 0.13 (s, 9H).

^{13}C NMR (100 MHz, $CDCl_3$): δ 210.0, 182.6, 169.1, 129.5, 105.3, 89.7, 75.4, 40.9, 35.3, 33.3, 31.5, 26.9, 26.6, 24.0, 22.0, -0.2.

HRMS (ESI+): calculated for $C_{18}H_{28}NaO_3Si$ $[M+Na]^+$ 343.1700, found: 343.1687.

Physical Appearance: Pale yellow liquid.

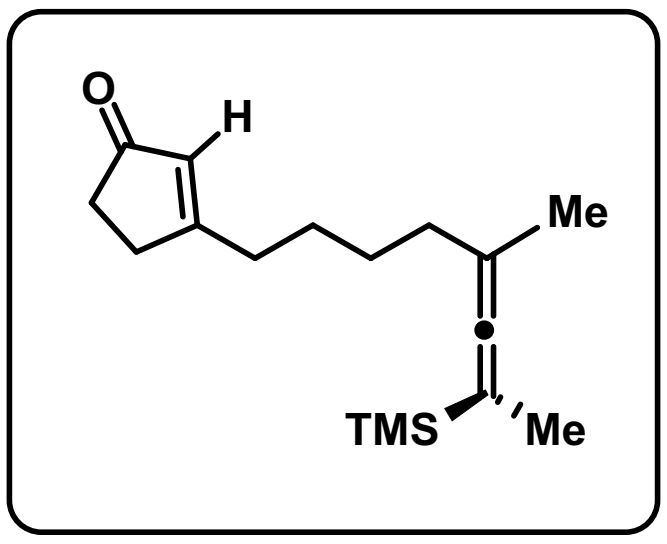

(R)-3-(5-methyl-7-(trimethylsilyl)octa-5,6-dien-1-yl)cyclopent-2-en-1-one (SI-7)

^{1}H NMR: (400 MHz, $CDCl_3$): δ 5.91 (s, 1H), 2.56-2.54 (m, 2H), 2.40-2.36 (m, 4H), 1.90 (t, J = 7.4 Hz, 2H), 1.60 (s, 3H), 1.60 (s, 3H), 1.57-1.55 (m, 2H), 1.45-1.37 (m, 2H), 0.02 (s, 9H).

^{13}C NMR (100 MHz, $CDCl_3$): δ 210.1, 204.2, 183.1, 129.5, 92.0, 90.9, 35.3, 33.5, 33.3, 31.5, 27.5, 26.7, 18.6, 15.8, -1.7.

HRMS (ESI+): calculated for $C_{17}H_{29}OSi$ $[M+H]^+$ 277.1982, found: 277.1974.

Physical Appearance: colorless liquid.

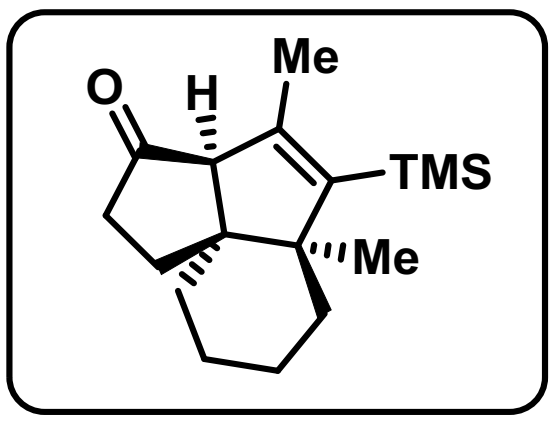

(3aR,5aS,9aS)-4,5a-dimethyl-5-(trimethylsilyl)-1,2,3a,5a,6,7,8,9-octahydro-3H-cyclopenta[c]inden-3-one (SI-8)

^{1}H NMR: (400 MHz, $CDCl_3$): δ 3.05 (s, 1H), 2.28-2.13 (m, 2H), 1.99-1.88 (m, 1H), 1.74-1.74 (m, 3H), 1.71-1.68 (m, 2H), 1.43-1.35 (m, 3H), 1.30-1.16 (m, 3H), 1.11 (s, 3H), 1.06-0.92 (m, 1H) 0.15 (s, 9H).

^{13}C NMR (100 MHz, $CDCl_3$): δ 219.3, 148.5, 142.8, 68.4, 54.4, 53.6, 40.3, 37.2, 32.6, 29.2, 22.7, 21.1, 18.0, 15.6, 1.4.

HRMS (ESI+): calculated for $C_{17}H_{29}OSi$ $[M+H]^+$ 277.1982, found: 277.1978.

Physical Appearance: colorless liquid.

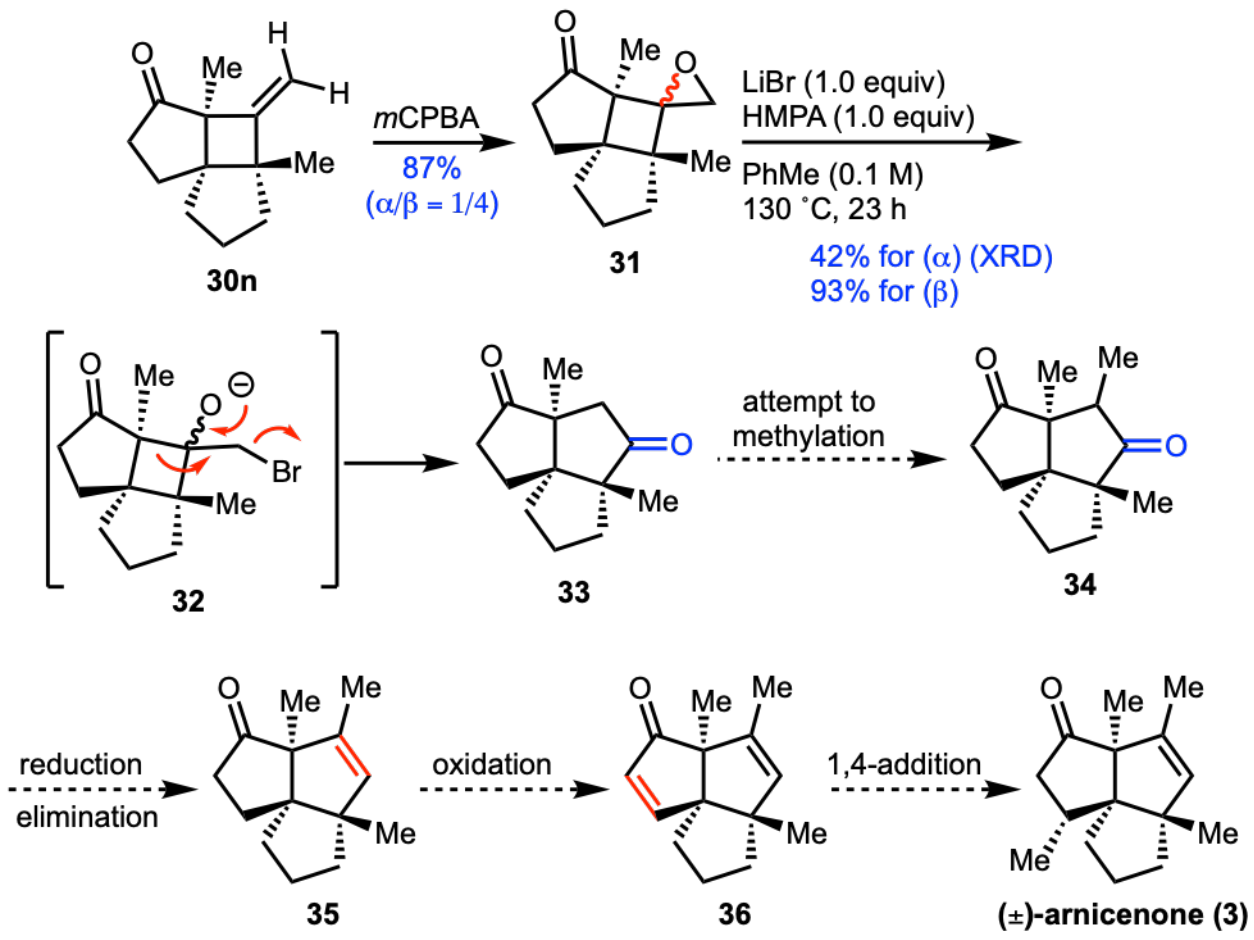

Under a nitrogen atmosphere, starting material **30n** (360 mg, 1.89 mmol) and *m*-chloroperoxybenzoic acid (*m*-CPBA, 522 mg, 2.33 mmol) were dissolved in CH_2Cl_2 (3.15 mL, 0.6 M) in a round-bottom flask. After complete consumption of the starting material monitored by TLC, the reaction was quenched by slow addition of saturated aqueous $NaHCO_3$ (10 mL). The mixture was extracted with EtOAc (50 mL) and brine (50 mL) three times. The combined organic layers were dried over $MgSO_4$, filtered, and concentrated under reduced pressure to afford the crude product. Purification by flash column chromatography (EtOAc/hexanes = 1:8) afforded the corresponding product **31**

(339 mg, 87%, α/β = 1/4, CHL-II-18).

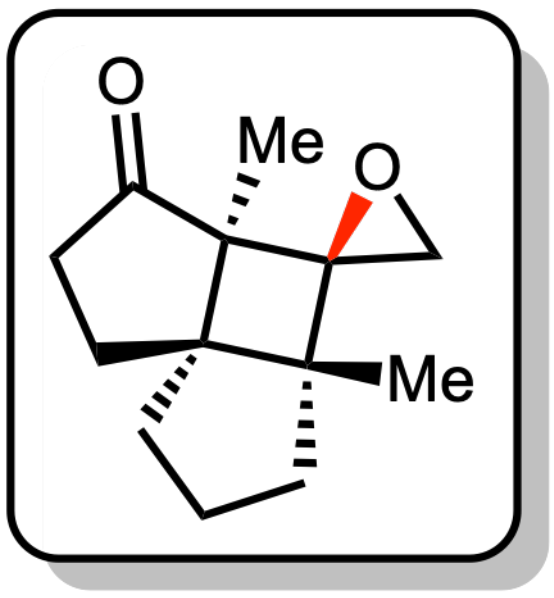

(3aR,4S,4aR,7aR)-3a,4a-dimethylhexahydrospiro[cyclobuta[1,2:1,4]di[5] annulene-4,2'-oxiran]-3(3aH)-one (31β)

^{1}H NMR (400 MHz, $CDCl_3$): δ 2.76-2.61 (m, 2H), 2.56 (d, *J* = 4.7 Hz, 1H), 2.44-2.35 (m, 1H), 2.11-2.04 (m, 1H), 1.85-1.76 (m, 2H), 1.71-1.58 (m, 4H), 1.41-1.28 (m, 1H), 1.05 (s, 3H), 0.95 (s, 3H).

^{13}C NMR (100 MHz, $CDCl_3$): δ 219.4, 67.1, 54.9, 54.2, 51.2, 47.6, 38.8, 38.7, 33.8, 26.3, 25.3, 15.8, 11.5.

HRMS (ESI+): calculated for $C_{13}H_{18}NaO_2$ $[M+Na]^+$ 229.1199, found: 229.1206.

Physical Appearance: white solid.

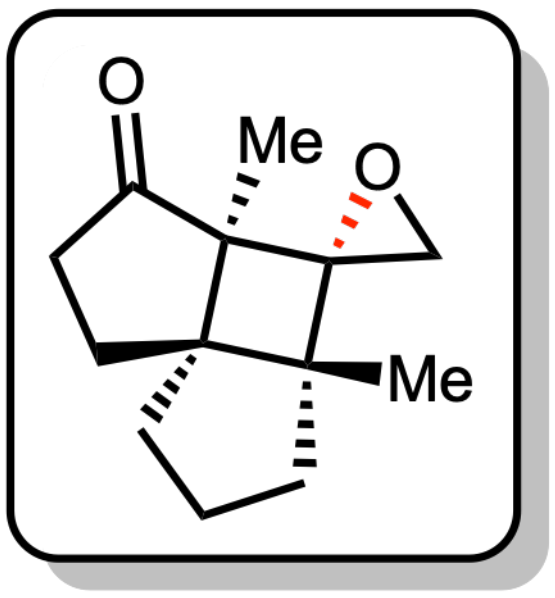

(3aR,4R,4aR,7aR)-3a,4a-dimethylhexahydrospiro[cyclobuta[1,2:1,4]di [5]annulene-4,2'-oxiran]-3(3aH)-one (31α)

^{1}H NMR (400 MHz, $CDCl_3$): δ 2.75-2.58 (m, 2H), 2.54 (d, *J* = 5.0 Hz, 1H), 2.51-2.33 (m, 1H), 2.05 (m, 1H), 1.95-1.85 (m, 2H), 1.85-1.68 (m, 4H), 1.64-1.55 (m, 2H), 1.28-1.21 (m, 1H), 1.08 (s, 3H), 0.89 (s, 3H).

^{13}C NMR (100 MHz, $CDCl_3$): δ 222.3, 67.2, 55.6, 54.7, 51.6, 47.5, 38.5, 36.3, 34.9, 25.6, 25.6, 19.1, 8.4.

HRMS (ESI$^+$): calculated for $C_{13}H_{18}NaO_2$ $[M+Na]^+$ 229.1199, found: 229.1206.

Physical Appearance: white solid.

To LiBr (7.4 mg, 0.085 mmol), and starting material **31β** (17.5 mg, 0.085 mmol) in a sealed tube were added hexamethylphosphoramide (HMPA, 15.2 mg, 0.085 mmol), and toluene (0.85 mL, 0.1 M) under a nitrogen atmosphere. The reaction mixture was stirred until complete consumption of the starting material monitored by TLC. The solvent was removed under reduced pressure to afford the crude product. Purification by flash column chromatography (EtOAc/hexanes = 1:11) afforded the corresponding product **33** (16.3 mg, 93%, CHL-I-197).

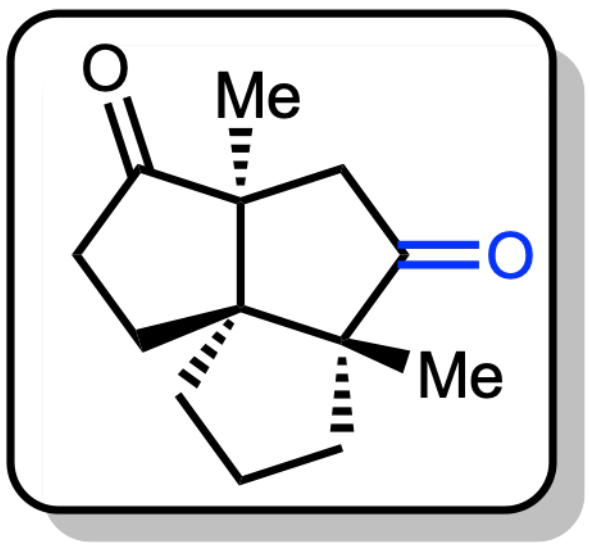

(3aS,5aR,8aR)-3a,5a-dimethyloctahydrocyclopenta[c]pentalene-3,5-dione (33)

^{1}H NMR (400 MHz, $CDCl_3$): δ 2.78 (d, J = 19.2 Hz, 1H), 2.63-2.43 (m, 1H), 2.25-2.01 (m, 4H), 1.99-1.84 (m, 2H), 1.66-1.51 (m, 3H), 1.48-1.34 (m, 1H), 1.13 (s, 3H), 0.93 (s, 3H).

^{13}C NMR (100 MHz, $CDCl_3$): δ 222.0, 221.1, 59.8, 59.6, 52.8, 45.5, 39.3, 37.0, 35.9, 27.2, 24.0, 19.1, 18.6.

HRMS (ESI$^+$): calculated for $C_{13}H_{18}NaO_2$ $[M+Na]^+$ 229.1199, found: 229.1206.

Physical Appearance: white solid.

Total synthesis of (±)-arnicenone (**3**) via [2+2] cycloaddition/ring expansion route:

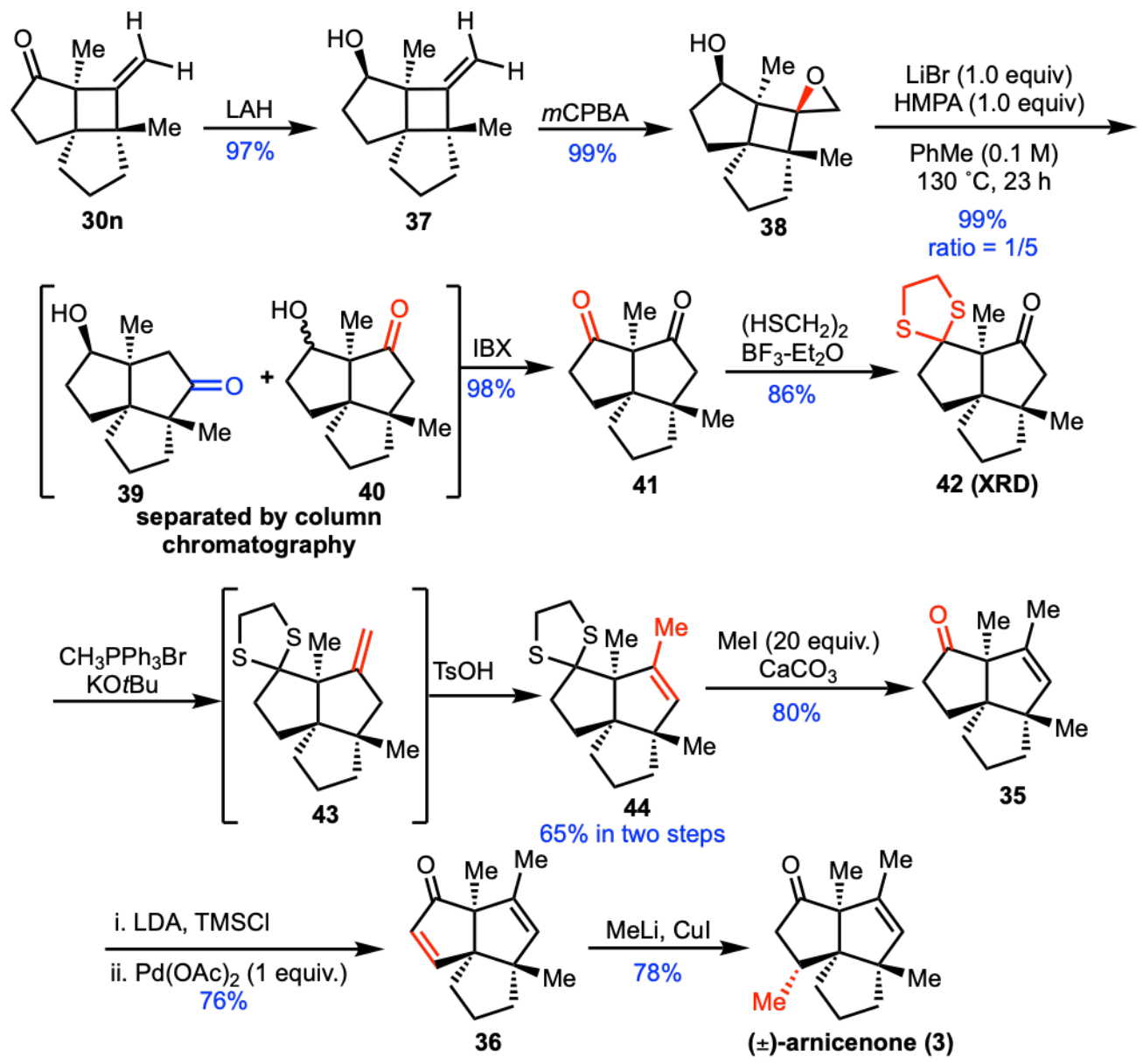

Under a nitrogen atmosphere, the starting material **30n** (200 mg, 1.05 mmol) was dissolved in THF (2.6 mL, 0.4 M) in a round-bottom flask and cooled to 0 °C for 10 min. A solution of lithium aluminum hydride ($LiAlH_4$, 1.05 mL, 1.0 M in THF, 1.05 mmol) was then added dropwise. After complete consumption of the starting material monitored by TLC, saturated aqueous potassium sodium tartrate (Rochelle salt) was added to quench the reaction, and the mixture was stirred for 1 h. The mixture was extracted with EtOAc (50 mL) and brine (50 mL) three times. The combined organic layers were dried over $MgSO_4$, filtered, and concentrated under reduced pressure to afford the crude product. Purification by flash column chromatography (EtOAc/hexanes = 1:8) afforded the desired product **37** (196 mg, 97%, CHL-II-87).

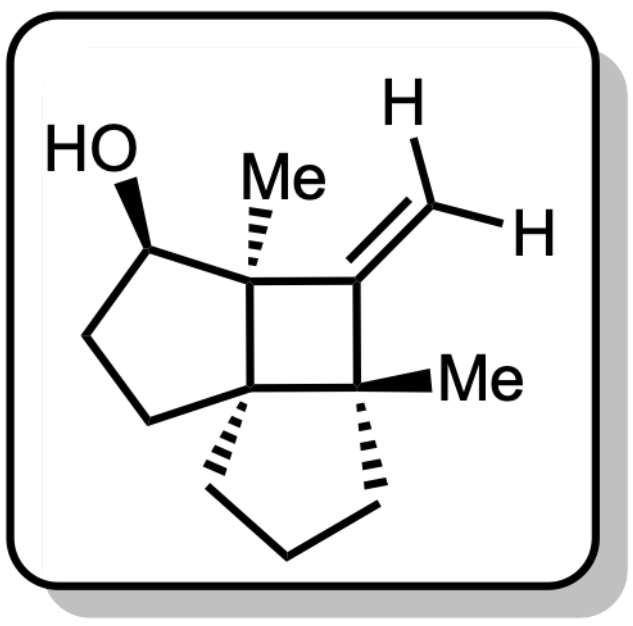

(3R,3aS,4aS,7aR)-3a,4a-dimethyl-4-methyleneoctahydro-1H-cyclobuta[1,2:1,4]di[5]annulen-3-ol (37)

^{1}H NMR (400 MHz, $CDCl_3$): δ 4.90 (s, 1H), 4.80 (s, 1H), 3.77-3.61 (m, 1H), 2.10-1.92 (m, 1H), 1.84-1.69 (m, 3H), 1.69-1.59 (m, 3H), 1.52-1.38 (m, 3H), 1.16 (s, 3H), 1.11 (s, 3H).

^{13}C NMR (100 MHz, $CDCl_3$): δ 163.7, 104.7, 81.5, 57.4, 54.1, 52.2, 43.4, 34.8, 33.4, 28.6, 25.6, 18.7, 17.9.

HRMS (ESI+): calculated for $C_{13}H_{21}O$ $[M+H]^+$ 193.1587, found: 193.1584.

Physical Appearance: White crystal.

To a solution of starting material **37** (97 mg, 0.5 mmol) in CH_2Cl_2 (0.8 mL, 0.6 M) at 0 °C under a nitrogen atmosphere was added *m*-chloroperoxybenzoic acid (*m*-CPBA, 140 mg, 0.624 mmol). After complete consumption of starting material **37** (monitored by TLC), the reaction was quenched by slow addition of saturated aqueous $NaHCO_3$ (10 mL). The mixture was extracted with EtOAc (50 mL) and brine (50 mL) three times. The combined organic layers were dried over $MgSO_4$, filtered, and concentrated under reduced pressure to afford the crude product. Purification by flash column chromatography (EtOAc/hexanes = 1:6) afforded the corresponding product **38** (103 mg, 99%, CHL-II-111).

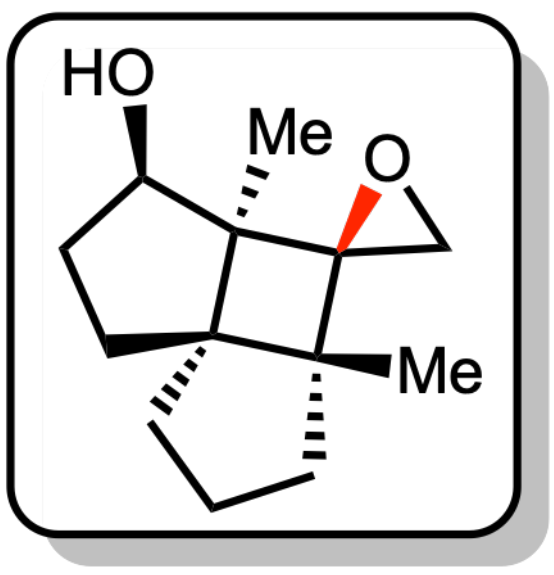

(3R,3aS,4S,4aR)-3a,4a-dimethyloctahydrospiro[cyclobuta[1,2:1,4]di[5]annulene-4,2'-oxiran]-3-ol (38)

^{1}H NMR (400 MHz, $CDCl_3$): δ 3.66 (dd, J = 10.8, 6.8 Hz, 1H), 2.70 (d, J = 4.2 Hz, 1H), 2.58 (d, J = 4.2 Hz, 1H), 2.16-2.04 (m, 1H), 2.01-1.85 (m, 1H), 1.82-1.64 (m, 5H), 1.55-1.45 (m, 1H), 1.44-1.33 (m, 2H), 1.12 (s, 3H), 0.96 (s, 3H).

^{13}C NMR (100 MHz, $CDCl_3$): δ 84.0, 71.8, 55.9, 49.0, 48.2, 48.1, 39.8, 34.6, 33.3, 28.6, 25.6, 16.3, 14.4.

HRMS (ESI+): calculated for $C_{13}H_{20}NaO_2$ $[M+Na]^+$ 231.1356, found: 231.1366.

Physical Appearance: White crystal.

To LiBr (12 mg. 0.14 mmol), and starting material **38** (28.6 mg, 0.14 mmol) in a sealed tube were added hexamethylphosphoramide (HMPA, 24.1 uL, 0.14 mmol), and toluene (1.4 mL, 0.1 M) under a nitrogen atmosphere. The reaction mixture was stirred until complete consumption of the starting material monitored by TLC. The solvent was removed under reduced pressure to afford the crude product. Purification by quick flash column chromatography (EtOAc/hexanes = 1:11) afforded the relatively clean corresponding products **39** (5 mg, 17%) and **40** (23.5 mg, 81%) (CHL-II-91).

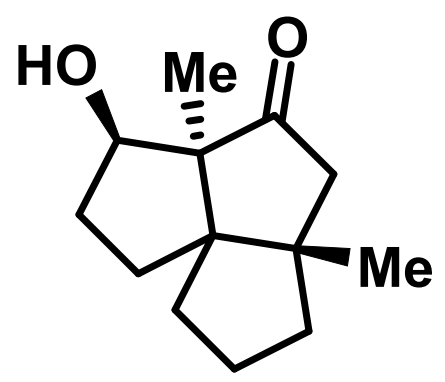

a

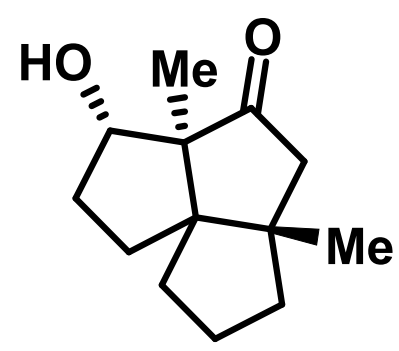

b

(3a*S*,5a*S*,8a*S*)-3-hydroxy-3a,5a-dimethyl-4-oxodecahydrocyclopenta[*c*]pentalen-2-ylium (40)

Note: The sample was obtained as a mixture of two epimers, and extensive overlap of their NMR resonances precluded unambiguous assignment of the individual signals. Subsequent IBX oxidation converted both epimers into the same diketone **41**, which could be readily purified and unambiguously characterized.

^{1}H NMR (400 MHz, $CDCl_3$): δ 3.77 (m, 0.5 Ha), 3.75 (m, 1Hb) 2.50 (m, 1Hb), 2.49-2.30 (m, 3H), 2.29-2.19 (m, 2H), 2.17-1.94 (m, 3H), 1.93-1.91 (m, 3H), 1.88-1.62 (m, 3H), 1.44-1.39 (m, 3H), 1.12 (m, 6H) 1.10 (m, 1H), 1.07 (m, 1H).

^{13}C NMR (100 MHz, $CDCl_3$): δ 223.6 x 2, 83.2, 79.3, 63.4, 62.3, 62.1, 53.1, 50.9, 46.3, 46.3, 41.9, 41.9, 35.6, 35.1, 33.7, 33.6, 32.4, 32.2, 23.2, 22.7, 22.6, 22.5, 19.1, 14.8.

HRMS (ESI+): calculated for $C_{13}H_{21}O$ $[M+H]^+$ 209.1536, found: 209.1535.

Physical Appearance: White crystal.

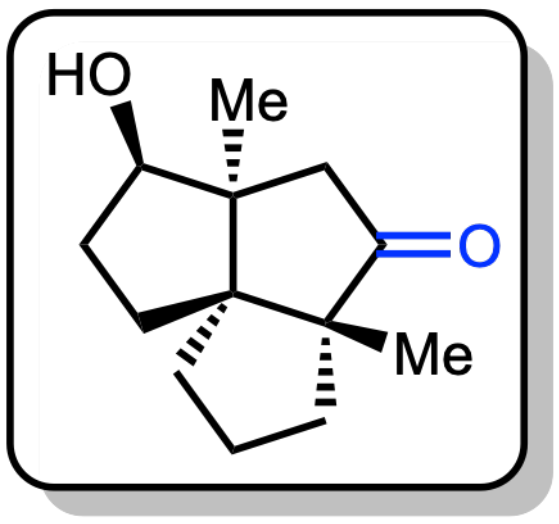

(3aR,5aS)-6-hydroxy-3a,5a-dimethyloctahydrocyclopenta[c]pentalen-4(5H)-one (39)

^{1}H NMR (400 MHz, $CDCl_3$): δ 3.83 (m, 1H), 2.64 (d, *J* = 17.9 Hz, 1H), 2.11-1.87 (m, 6H), 1.50-1.38 (m, 5H), 1.33 (m, 1H), 1.10 (s, 3H), 1.08 (s, 3H).

^{13}C NMR (100 MHz, $CDCl_3$): δ 222.8, 82.3, 60.8, 59.0, 49.2, 46.2, 41.4, 39.0, 32.8, 31.6, 24.8, 22.2, 20.0.

HRMS (ESI+): calculated for $C_{13}H_{21}O$ $[M+H]^+$ 209.1536, found: 209.1535.

Physical Appearance: White crystal.

To a solution of starting material **40** (19 mg, 0.091 mmol) in DMSO (0.2 mL, 0.5 M) under a nitrogen atmosphere was added 2-iodoxybenzoic acid (IBX, 64 mg, 0.228 mmol). After complete consumption of the starting material (monitored by TLC), saturated aqueous $Na_2S_2O_3$ (20 mL) was added to quench the reaction. The mixture was extracted with EtOAc (100 mL) and brine (100 mL) three times. The combined organic layers were dried over $MgSO_4$, filtered, and concentrated under reduced pressure to afford the crude product. Purification by flash column chromatography (EtOAc/hexanes = 1:6) afforded the desired product **41** (19 mg, 98%, CHL-II-105).

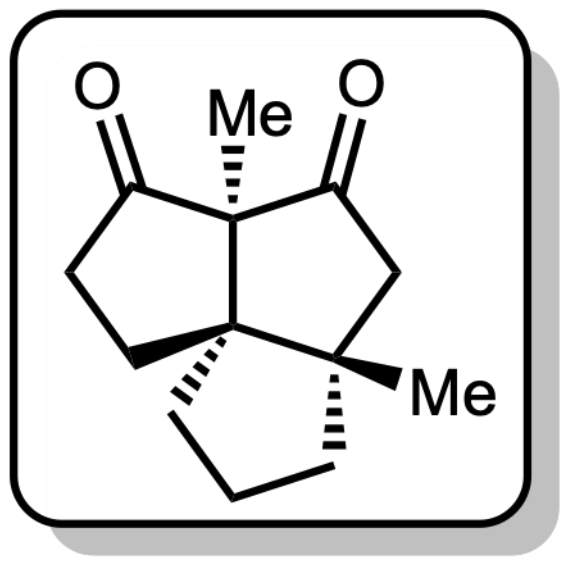

(3aS,5aS)-3a,5a-dimethylhexahydrocyclopenta[c]pentalene-3,4(3aH,5H)-dione (41)

^{1}H NMR (400 MHz, $CDCl_3$): δ 2.41-2.34 (m, 2H), 2.33-2.25 (m, 1H), 2.25-2.14 (m, 2H), 1.99-1.89 (m, 1H), 1.87-1.78 (m, 1H), 1.77-1.67 (m, 2H), 1.67-1.56 (m, 2H), 1.49-1.36 (m, 1H), 1.19 (s, 3H), 1.14 (s, 3H).

^{13}C NMR (100 MHz, $CDCl_3$): δ 213.4, 210.8, 68.7, 61.1, 51.7, 46.1, 42.5, 37.6, 35.6, 28.9, 23.7, 23.2, 16.5.

HRMS (ESI+): calculated for $C_{13}H_{19}O$ $[M+H]^+$ 207.1380 , found: 207.1382.

Physical Appearance: White crystal.

To a solution of starting material **41** (12 mg, 0.06 mmol) and 1,2-ethanedithiol (5.6 uL, 0.07 mmol) in CH_2Cl_2 (0.1 mL, 0.5 M) under a nitrogen atmosphere was added boron trifluoride diethyl etherate ($BF_3 \cdot Et_2O$, 8.2 uL, 0.07 mmol) dropwise at 0 °C after cooling for 10 min. The reaction mixture was stirred until no further consumption of the starting material (monitored by TLC). The reaction was quenched with saturated aqueous $NaHCO_3$, and the mixture was extracted with EtOAc (100 mL) and brine (100 mL) three times. The combined organic layers were dried over $MgSO_4$, filtered, and concentrated under reduced pressure to afford the crude product. Purification by flash column chromatography (EtOAc/hexanes = 1:20) afforded the desired product **42** (15 mg, 86%).

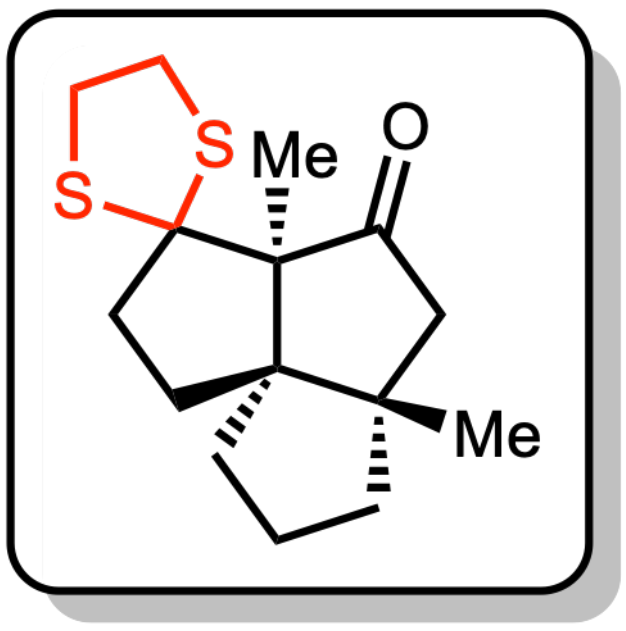

(3aR,5aS,8aR)-3a,5a-dimethylhexahydro-5H-spiro[cyclopenta[c]pentalene-3,2'-[1,3]dithiolan]-4(3aH)-one (42)

^{1}H NMR (400 MHz, $CDCl_3$): δ 3.27-3.07 (m, 4H), 2.36 (d, *J* = 17.7, 1H), 2.29 (d, *J* = 17.9, 1H), 2.20-2.01 (m, 3H), 1.95-1.82 (m, 1H), 1.80-1.59 (m, 4H), 1.53-1.37 (m, 2H), 1.23 (s, 3H), 1.12 (s, 3H).

^{13}C NMR (100 MHz, $CDCl_3$): δ 220.1, 79.5, 64.0, 62.7, 52.0, 46.4, 45.2, 41.2, 38.7, 38.6, 35.4, 33.9, 23.1, 22.0, 18.9.

HRMS (ESI+): calculated for $C_{15}H_{23}OS_2$ $[M+H]^+$ 283.1185, found: 283.1185.

Physical Appearance: White crystal.

Under a nitrogen atmosphere, to a suspension of methyltriphenylphosphonium bromide (27 mg, 0.07 mmol) and potassium *tert*-butoxide (0.073 mL, 1M in THF, 0.07 mmol) in toluene (0.071 mL, 0.5 M) in viak was heated to 110 °C for 1h to generate the corresponding ylide. To a solution of starting material **42** (10 mg, 0.04 mmol) in toluene (0.071 mL, 0.5 M) under a nitrogen atmosphere in a sealed tube was added the freshly prepared ylide solution dropwise. The reaction mixture was stirred until no further consumption of the starting material (monitored by TLC). The reaction was quenched with water, and the mixture was extracted with Et_2O (50 mL) and brine (50 mL) three times. The combined organic layers were dried over $MgSO_4$, filtered, and concentrated under reduced pressure to afford the crude product **43**.

Under a nitrogen atmosphere, to a solution of starting material **43** (crude product from the Wittig reaction) in CH_2Cl_2 (0.6 mL, 0.5 M) was added *p*-toluenesulfonic acid

(p-TsOH, 5.0 mg, 0.03 mmol). The reaction mixture was transferred to a sealed tube and heated at reflux until no further consumption of the starting material (monitored by TLC). The reaction was quenched with water, and the mixture was extracted with Et_2O (50 mL) and brine (50 mL) three times. The combined organic layers were dried over $MgSO_4$, filtered, and concentrated under reduced pressure to afford the crude product. Purification by flash column chromatography (EtOAc/hexanes = 1:60) afforded the desired product **44** (6 mg, 65% in two-step, CHL-II-143).

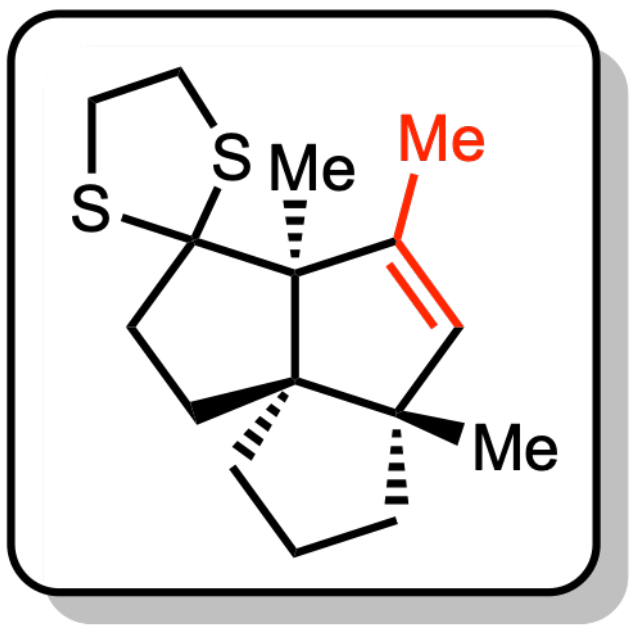

(3aS,5aS,8aR)-3a,4,5a-trimethyl-1,2,3a,5a,7,8-hexahydro-6H-spiro[cyclopenta [c]pentalene-3,2'-[1,3]dithiolane] (44)

^{1}H NMR (700 MHz, $CDCl_3$): δ 4.92 (d, J = 1.5 Hz, 1H), 3.24-3.10 (m, 4H), 2.21-2.13 (m, 2H), 2.11-2.06 (m, 1H), 2.05-1.98 (m, 1H), 1.71 (d, J = 1.4 Hz, 3H), 1.42-1.38 (m, 1H), 1.31-1.24 (m, 7H), 1.14-1.08 (m, 4H).

^{13}C NMR (212.5 MHz, $CDCl_3$): δ 141.7, 134.3, 82.3, 65.1, 62.0, 55.2, 45.9, 42.0, 40.7, 37.9, 37.8, 35.4, 24.5, 24.3, 21.1, 14.4.

HRMS (ESI^+): calculated for $C_{16}H_{25}S_2$ $[M+H]^+$ 281.1392, found: 281.1391.

Physical Appearance: White crystal.

Under a nitrogen atmosphere, to starting material **44** (35 mg, 0.14 mmol) and $CaCO_3$ (30 mg, 0.3 mmol) in $MeCN/H_2O$ (4.3 mL, v/v = 5.1/1.0) was added methyl iodide (MeI, 0.2 mL, 2.9 mmol). The reaction mixture was transferred to a sealed tube and heated at reflux until complete consumption of the starting material (monitored by TLC). The reaction was quenched with water, and the mixture was extracted with Et_2O

(100 mL) and brine (100 mL) three times. The combined organic layers were dried over $MgSO_4$, filtered, and concentrated under reduced pressure to afford the crude product. Purification by flash column chromatography (hexanes) afforded the desired product **35** (20.5 mg, 80%, CHL-II-145).

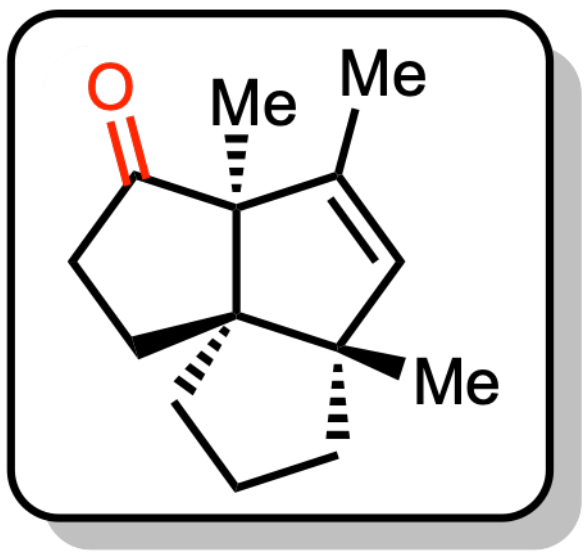

(3aS,5aS,8aR)-3a,4,5a-trimethyl-1,2,5a,6,7,8-hexahydrocyclopenta[c]pentalen-3(3aH)-one (35)

^{1}H NMR (700 MHz, $CDCl_3$): δ 5.06 (m, 1H), 2.47-2.36 (m, 1H), 2.12-2.02 (m, 2H), 1.88-1.83 (m, 1H), 1.61-1.20 (m, 6H), 1.58 (d, J = 1.5 Hz, 3H), 1.14 (s, 3H), 1.03 (s, 3H).

^{13}C NMR (175 MHz, $CDCl_3$): δ 220.4, 138.5, 136.2, 65.4, 59.8, 56.6, 42.2, 38.6, 37.1, 28.7, 23.9, 22.4, 15.5, 12.4.

HRMS (ESI$^+$): calculated for $C_{14}H_{21}O$ $[M+H]^+$ 205.1587, found: 205.1584.

Physical Appearance: pale yellow solid.

To a solution of diisopropylamine (30.1 µL, 0.21 mmol) in THF (1 mL, 0.02 M) at 0 °C was added *n*-butyllithium (0.16 mL, 1.16 M in THF, 0.19 mmol) dropwise under a nitrogen atmosphere. The reaction mixture was stirred for 30 min and then cooled to −78 °C. A solution of triquinane **35** (4 mg, 0.02 mmol) in THF (0.5 mL) was added in one portion, and the reaction mixture was stirred at −78 °C for 1 h. A solution of trimethylsilyl chloride (TMSCl, 24.7 µL, 0.19 mmol) and triethylamine (8.2 µL, 0.06 mmol) in THF (0.5 mL) was then added, and the reaction mixture was allowed to warm gradually to room temperature. After complete consumption of the starting material (monitored by TLC), the reaction was quenched with saturated aqueous $NaHCO_3$. The

mixture was extracted with Et_2O (50 mL) and brine (50 mL) three times. The combined organic layers were dried over Na_2SO_4, filtered, and concentrated under reduced pressure to afford the crude intermediate.

Without further purification, the crude product was dissolved in MeCN (0.65 mL, 0.03 M), and $Pd(OAc)_2$ (5 mg, 0.024 mmol) was added. After complete consumption of the silyl enol ether intermediate (monitored by TLC), the reaction mixture was filtered through a short silica gel column (5 cm, eluted with EtOAc) to remove insoluble materials. The filtrate was concentrated under reduced pressure to afford the crude product. Purification by flash column chromatography (EtOAc/hexanes = 1:30) afforded triquinane enone **36** (3.0 mg, 76%, CHL-II-148).

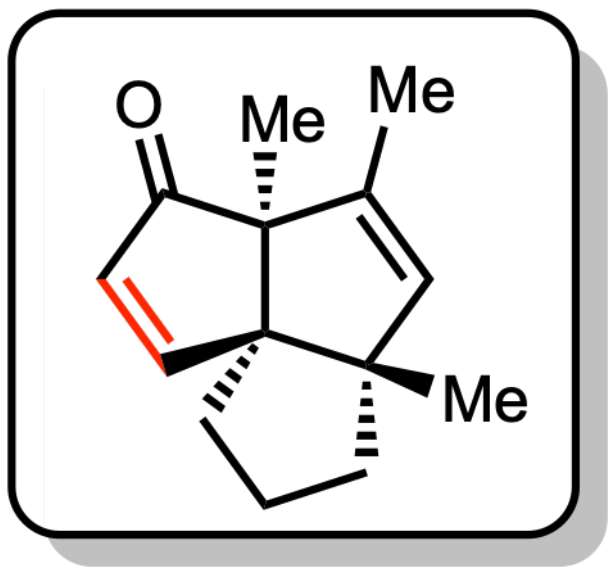

(3aS,5aS,8aR)-3a,4,5a-trimethyl-1,2,5a,6,7,8-hexahydrocyclopenta[c]pentalen-3(3aH)-one (36)

^{1}H NMR (400 MHz, $CDCl_3$): δ 7.31 (d, J = 5.6 Hz, 1H), 5.96 (d, J = 5.6 Hz, 1H), 4.92 (m, 1H), 1.91-1.84 (m, 1H), 1.73-1.59 (m, 3H), 1.69 (d, J = 1.4 Hz, 3H), 1.49-1.38 (m, 2H), 1.09 (s, 3H), 0.97 (s, 3H).

^{13}C NMR (175 MHz, $CDCl_3$): δ 212.7, 169.5, 138.3, 133.5, 129.7, 66.6, 64.5, 57.9, 41.1, 34.7, 25.6, 24.3, 17.2, 12.9.

HRMS (ESI+): calculated for $C_{14}H_{19}O$ $[M+H]^+$ 203.1430, found: 203.1429.

Physical Appearance: pale yellow solid.

Under a nitrogen atmosphere, to a suspension of CuI (167 mg, 0.876 mmol) in Et_2O (2.9 mL, 0.05 M) at −40 °C was added methyllithium (MeLi, 0.97 mL, 1.5 M in

Et_2O, 1.46 mmol) dropwise. The reaction mixture was stirred for 30 min at −40 °C, and a solution of triquinane enone **36** (30 mg, 0.146 mmol) in Et_2O (0.5 mL) was added in one portion. After complete consumption of starting material **36** (monitored by TLC), the reaction was quenched with saturated aqueous $NaHCO_3$. The mixture was extracted with Et_2O (100 mL) and (100 mL) three times. The combined organic layers were dried over Na_2SO_4, filtered, and concentrated under reduced pressure to afford the crude product. Purification by flash column chromatography (EtOAc/hexanes = 1:30) afforded the target natural product (±)-arnicenone (**3**) in 78% yield (25 mg) (HTH-II-130), whose spectroscopic data were consistent with those reported in the literature.[5,6]

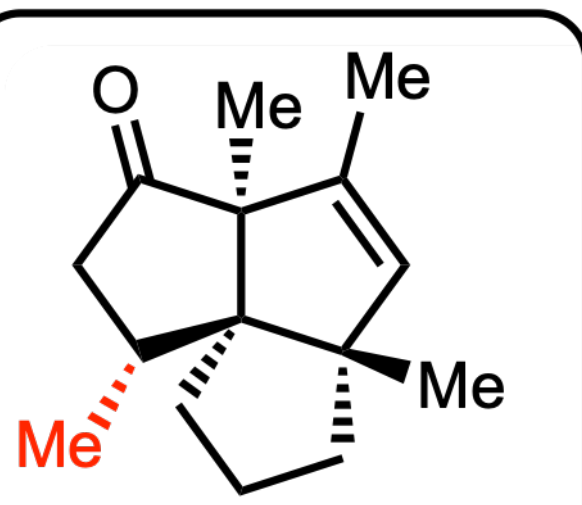

(±)-arnicenone (3)

(3aS,5aS,8aR)-3a,4,5a-trimethyl-1,2,5a,6,7,8-hexahydrocyclopenta[c]pentalen-3(3aH)-one (3)(arnicenone)

^{1}H NMR (500 MHz, $CDCl_3$): δ 5.06 (q, *J* = 1.5 Hz, 1H), 2.75 (dd, *J* = 16.8, 7.3 Hz, 1H), 2.22 (m, 1H), 1.83 (d, *J* = 16.8 Hz, 1H), 1.83-1.78 (m, 1H), 1.60-1.10 (m, 5H), 1.52 (d, *J* = 1.5 Hz, 3H), 1.16 (s, 3H), 1.08 (s, 3H), 0.84 (d, *J* = 7.5 Hz, 3H).

^{13}C NMR (125 MHz, $CDCl_3$): δ 219.5, 138.5, 136.5, 64.6, 62.3, 57.7, 45.0, 42.5, 35.3, 33.1, 23.7, 22.2, 19.0, 16.6, 12.1.

HRMS (ESI^+): calculated for $C_{15}H_{23}O$ $[M+H]^+$ 219.1743, found: 219.1745.

Physical Appearance: colorless solid.

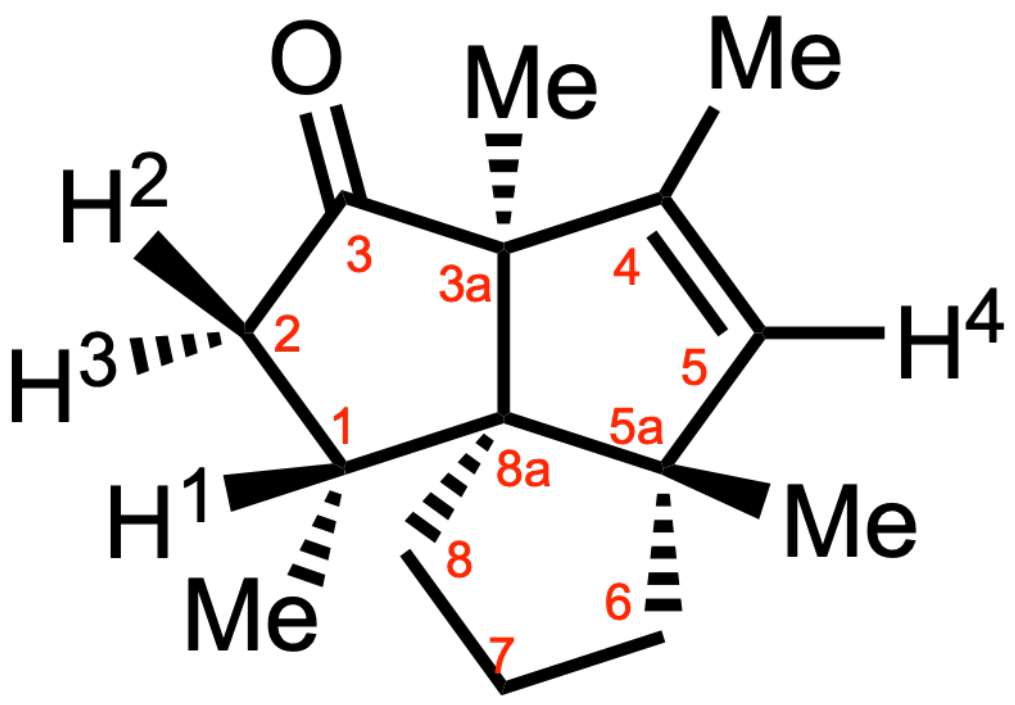

(±)-arnicenone

assignment	Natural (ppm) 200 MHz[4]	Paquette (ppm) 90 MHz[6]	Delta (ppm) Natural&Huang	Huang (ppm) 500 MHz
1-Me	0.86	0.87	0.02	0.84
3a-Me	1.11	1.10	0.03	1.08
5a-Me	1.18	1.18	0.02	1.16
4-Me	1.54	1.55	0.02	1.52
H^2	1.84	-	0.01	1.83
H^2	2.25	2.27	0.03	2.22
H^2	2.79	2.75	0.04	2.75
H^2	5.08	5.03	0.02	5.06
3*CH_2	1.02-1.76	1.40(br) H*7	-	1.10-1.60

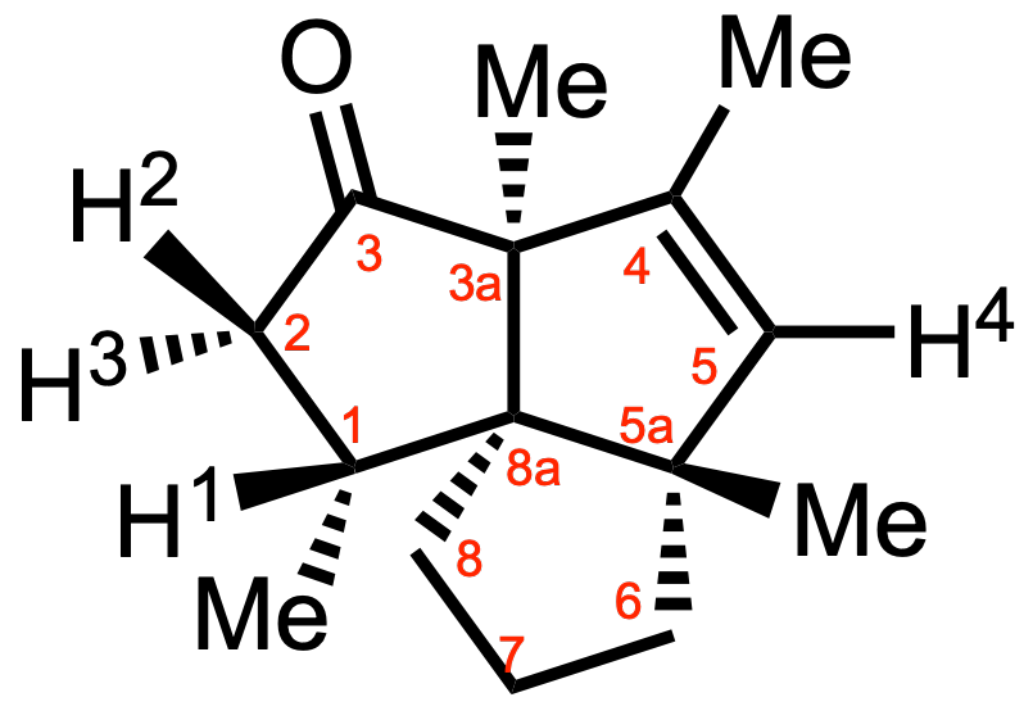

(±)-arnicenone

assignment	Natural (ppm) 22.6 MHz[4]	Delta (ppm) Natural&Huang	Huang (ppm) 500 MHz
1-Me	19.0	-	19.0
3a-Me	16.6	-	16.6
5a-Me	22.2	-	22.2
4-Me	12.1	-	12.1
7	23.8	0.1	23.7
1	33.1	-	33.1
8	35.4	0.1	35.3
6	42.6	0.1	42.5
2	45.0	-	45.0
5a	57.7	-	57.7
3a	62.3	-	62.3
8a	64.7	0.1	64.6
4	136.6	0.1	136.5
5	138.6	0.1	138.5
3	219.3	0.2	219.5

Total synthesis of (±)-arnicenone (**3**) via [3+2] route:

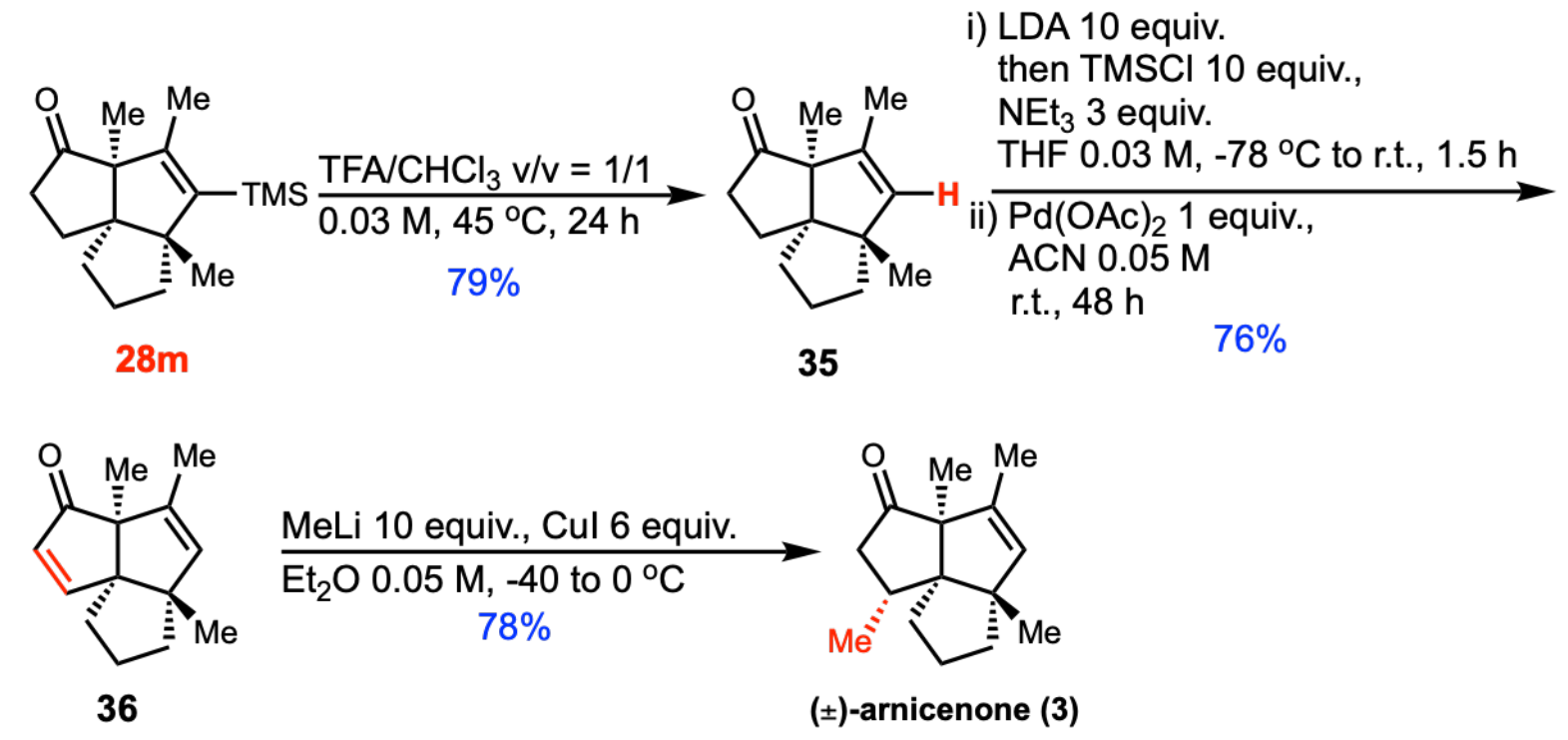

Note: **36** and **3** are identical to the [2+2] cycloaddition/ring expansion route.

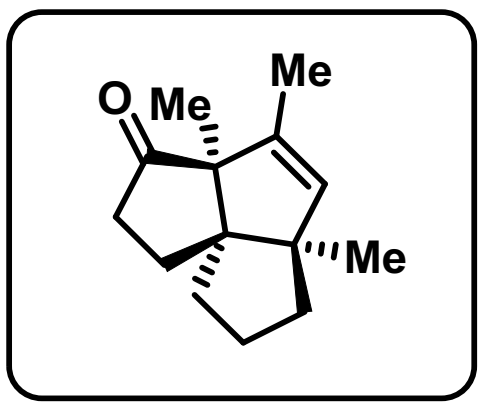

Under a nitrogen atmosphere, the starting material **28m** was dissolved in the chloroform and trifluoroacetic acid. The reaction mixture was stirred until complete consumption of the starting material **28m**, as monitored by TLC. The reaction was then quenched with saturated aqueous NH_4Cl (20 mL). The mixture was extracted with EtOAc (3×), and the combined organic layers were washed with brine, dried over $MgSO_4$, filtered, and concentrated under reduced pressure. The crude product was purified by flash column chromatography (EtOAc/hexanes = 1:4) to afford the desired product in 79% yield.

(3aS,5aR,8aR)-3a,4,5a-trimethyl-1,2,5a,6,7,8-hexahydrocyclopenta[c]pentalen-3(3aH)-one (35)

^{1}H NMR: (400 MHz, $CDCl_3$): δ 5.74 (s, 1H), 2.22-2.18 (m, 2H), 2.16-2.11 (m, 2H), 1.89-1.81 (m, 1H), 1.71-1.64 (m, 1H), 1.60-1.53 (m, 2H), 1.52-1.52 (m, 3H), 1.33-1.24 (m, 2H), 1.15 (s, 3H), 1.00-0.99 (m, 3H).

^{13}C NMR (100 MHz, $CDCl_3$): δ 219.6, 144.0, 134.9, 66.5, 61.1, 60.9, 36.9, 33.9, 28.7, 26.9, 26.6, 23.4, 15.9, 13.7.

HRMS (ESI+): calculated for $C_{14}H_{21}O$ $[M+H]^+$ 205.1587, found: 205.1584.

Physical Appearance: colorless liquid.

Attempt to total synthesis of (±)-*epi*-arnicenone:

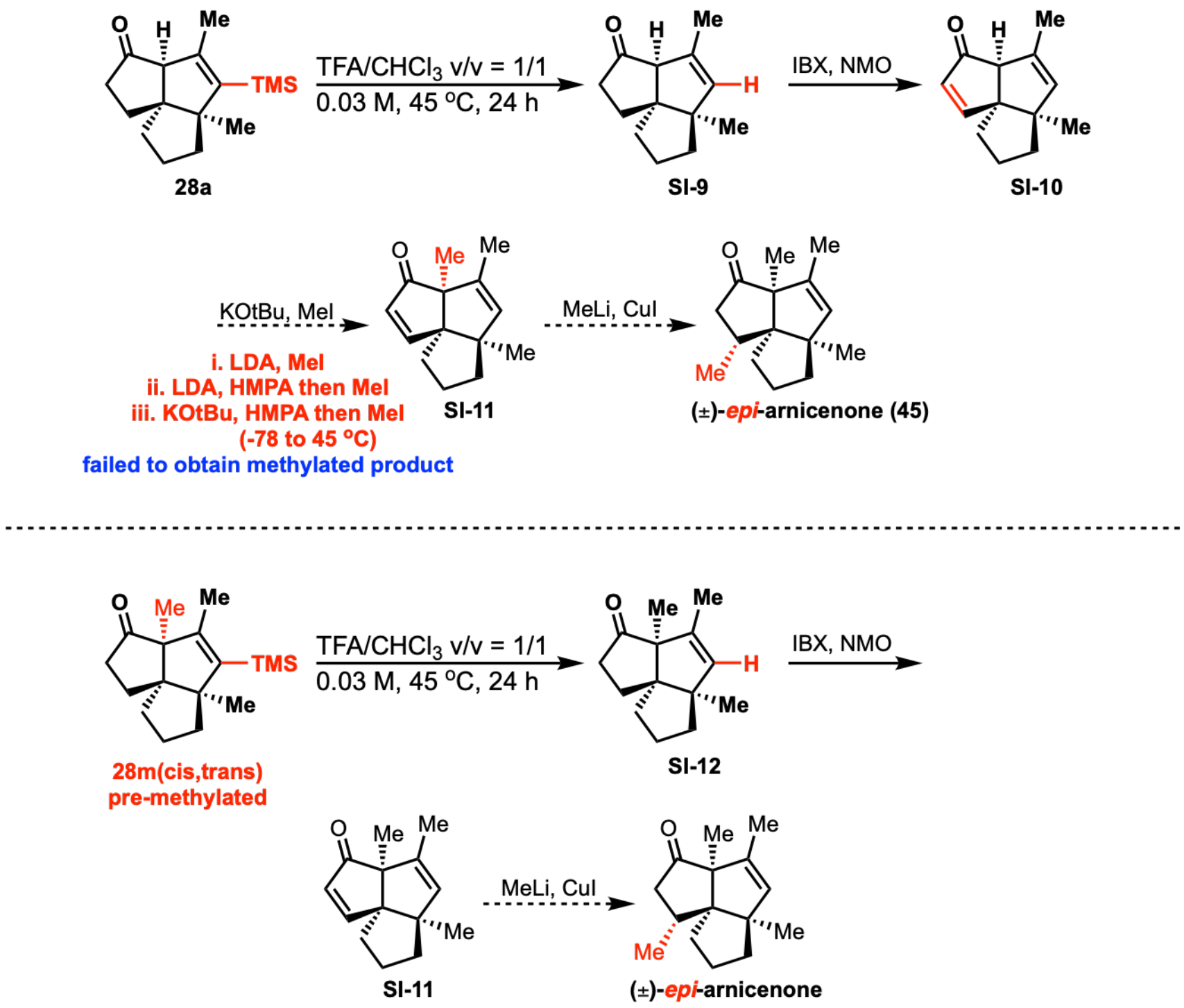

We initially investigated the synthesis of (±)-*epi*-arnicenone from **28a**. Desilylation of **28a** followed by IBX oxidation afforded enone **SI-10**. However, extensive attempts to introduce the required α-methyl group using a variety of enolate alkylation conditions were unsuccessful. We therefore revised our strategy and employed the pre-methylated *cis,trans*-[3+2] annulation product **28m** as the starting material. Desilylation of **28m** followed by IBX oxidation provided the corresponding α-methylated enone **SI-11**, thereby circumventing the problematic α-methylation step. Subsequent attempts to introduce the remaining methyl group through conjugate addition, however, did not afford the desired (±)-*epi*-arnicenone under the conditions examined.

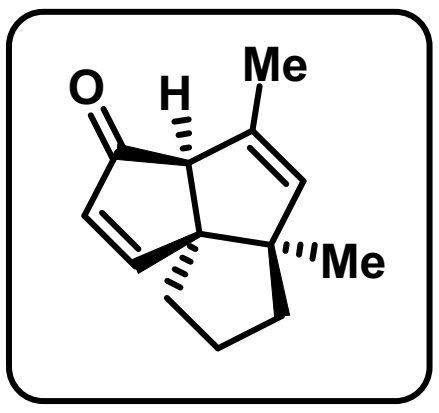

(3aS,5aR,8aS)-4,5a-dimethyl-5a,6,7,8-tetrahydrocyclopenta[c]pentalen-3(3aH)-one (SI-10)

^{1}H NMR: (400 MHz, $CDCl_3$): δ 7.48-7.47 (m, 1H), 5.68 (d, J = 5.8Hz, 1H), 5.58-5.57 (m, 1H), 2.86 (s, 1H), 2.29-2.20 (m, 2H), 2.08-2.00 (m, 1H), 1.83-1.73 (m, 1H), 1.65-1.65 (m, 3H), 1.50-1.42 (m, 2H), 0.98 (s, 3H).

^{13}C NMR (100 MHz, $CDCl_3$): δ 210.9, 170.3, 148.6, 131.7, 125.8, 70.1, 58.6, 57.9, 27.5, 26.9, 26.6, 25.5, 16.3.

HRMS (ESI+): calculated for $C_{13}H_{17}O$ $[M+H]^+$ 189.1274, found: 189.1279.

Physical Appearance: colorless liquid.

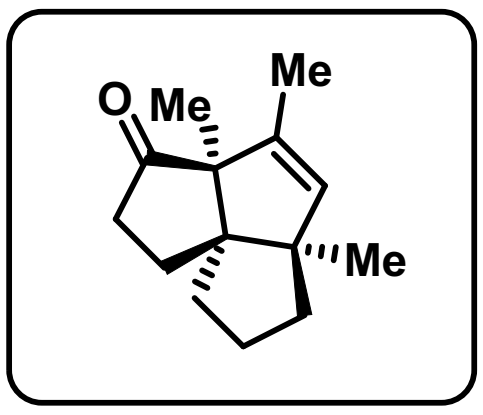

(3aS,5aR,8aR)-3a,4,5a-trimethyl-1,2,5a,6,7,8-hexahydrocyclopenta[c]pentalen-3(3aH)-one (SI-12)

^{1}H NMR: (400 MHz, $CDCl_3$): δ 5.74 (s, 1H), 2.22-2.18 (m, 2H), 2.16-2.11 (m, 2H), 1.89-1.81 (m, 1H), 1.71-1.64 (m, 1H), 1.60-1.53 (m, 2H), 1.52-1.52 (m, 3H), 1.33-1.24 (m, 2H), 1.15 (s, 3H), 1.00-0.99 (m, 3H).

^{13}C NMR (100 MHz, $CDCl_3$): δ 219.6, 144.0, 134.9, 66.5, 61.1, 60.9, 36.9, 33.9, 28.7, 26.9, 26.6, 23.4, 15.9, 13.7.

HRMS (ESI+): calculated for $C_{14}H_{21}O$ $[M+H]^+$ 205.1587, found: 205.1584.

Physical Appearance: colorless liquid.

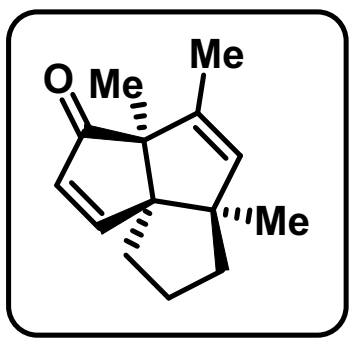

(3aS,5aR,8aR)-3a,4,5a-trimethyl-5a,6,7,8-tetrahydrocyclopenta[c]pentalen-3(3aH)-one (SI-11)

^{1}H NMR: (850 MHz, $CDCl_3$): δ 7.50 (d, J = 5.9Hz, 1H), 5.77 (d, J = 6.1Hz, 1H), 5.52 (s, 1H), 2.28-2.25 (m, 2H), 1.96-1.93 (m, 1H), 1.83-1.79 (m, 1H), 1.53-1.53 (m, 3H), 1.42-1.40 (m, 1H), 1.32-1.29 (m, 1H), 1.21 (s, 3H), 1.10 (s, 3H).

^{13}C NMR (212.5 MHz, $CDCl_3$): δ 212.3, 170.2, 152.0, 131.0, 125.1, 71.8, 58.9, 58.5, 28.5, 27.4, 26.3, 23.7, 14.0, 13.0.
HRMS (ESI+): calculated for $C_{14}H_{19}O$ $[M+H]^+$ 203.1430, found: 203.1430.
Physical Appearance: colorless liquid.

Density functional theory (DFT) calculations were performed using the Gaussian 16 suite of programs.[1] Geometry optimizations were carried out at the BP86 level of theory,[2, 3] employing the LANL2DZ basis set[4-6] for Ti and the 6-31G(d,p) basis set[7, 8] for all other atoms. Grimme's D3 dispersion correction with Becke–Johnson damping (GD3BJ) was included,[9, 10] and solvent effects (dichloromethane) were modeled using the SMD implicit solvation model.[11] Single-point free energy calculations were subsequently performed at the PBE0[12]/def2-TZVP[13] level with GD3BJ dispersion correction and SMD solvation (dichloromethane) based on the optimized geometries. Transition states were confirmed by vibrational frequency analysis (one imaginary frequency), and their connections to the corresponding reactants and products were verified by intrinsic reaction coordinate (IRC) calculations.

(1) **Gaussian 16**, Revision C.01; Gaussian, Inc.: Wallingford, CT, 2016.

(2) Becke, A. D. Density-Functional Exchange-Energy Approximation with Correct Asymptotic Behavior. *Phys. Rev. A* **1988**, *38*, 3098–3100.

(3) Perdew, J. P. Density-Functional Approximation for the Correlation Energy of the Inhomogeneous Electron Gas. *Phys. Rev. B* **1986**, *33*, 8822–8824.

(4) Hay, P. J.; Wadt, W. R. Ab Initio Effective Core Potentials for Molecular Calculations. Potentials for K to Au Including the Outermost Core Orbitals. *J. Chem. Phys.* **1985**, *82*, 299–310.

(5) Hay, P. J.; Wadt, W. R. Ab Initio Effective Core Potentials for Molecular Calculations. Potentials for the Transition Metal Atoms Sc to Hg. *J. Chem. Phys. 1985*, *82*, 270–283.

(6) Wadt, W. R.; Hay, P. J. Ab Initio Effective Core Potentials for Molecular Calculations. Potentials for Main Group Elements Na to Bi. *J. Chem. Phys. 1985*, *82*, 284–298.

(7) Feller, D. The Role of Databases in Support of Computational Chemistry Calculations. *J. Comput. Chem.* **1996**, *17*, 1571–1586.

(8) Schuchardt, K. L.; Didier, B. T.; Elsethagen, T.; Sun, L.; Gurumoorthi, V.; Chase, J.; Li, J.; Windus, T. L. Basis Set Exchange: A Community Database for Computational Sciences. *J. Chem. Inf. Model.* **2007**, *47*, 1045–1052.

(9) Grimme, S.; Antony, J.; Ehrlich, S.; Krieg, H. A Consistent and Accurate *Ab Initio* Parametrization of Density Functional Dispersion Correction (DFT-D) for the 94 Elements H–Pu. *J. Chem. Phys.* **2010**, *132*, 154104.

(10) Grimme, S.; Ehrlich, S.; Goerigk, L. Effect of the Damping Function in Dispersion Corrected Density Functional Theory. *J. Comput. Chem.* **2011**, *32*, 1456–1465.

(11) Marenich, A. V.; Cramer, C. J.; Truhlar, D. G. Universal Solvation Model Based on Solute Electron Density and on a Continuum Model of the Solvent Defined by the Bulk Dielectric Constant and Atomic Surface Tensions. *J. Phys. Chem. B* **2009**, *113*, 6378–6396.

(12) Adamo, C.; Barone, V. Toward Reliable Density Functional Methods without Adjustable Parameters: The PBE0 Model. *J. Chem. Phys.* **1999**, *110*, 6158–6170.

(13) Weigend, F.; Ahlrichs, R. Balanced Basis Sets of Split Valence, Triple Zeta Valence and Quadruple Zeta Valence Quality for H to Rn: Design and Assessment of Accuracy. *Phys. Chem. Chem. Phys.* **2005**, *7*, 3297–3305.
