# Supplementary material for: Diastereodivergent Access to cis,cis- and cis,trans-Angular Triquinanes: Total Synthesis of (±)-Arnicenone
Source: J Am Chem Soc. 2026 Sep 22;148(39):41724–32. doi: 10.1021/jacs.6c09019 (PMC13651531; doi:10.1021/jacs.6c09019)
Supplement: Supplementary file 3 [file ja6c09019_si_001.zip › FID 24-26/26a/HTH-TMS allene MS.pdf]

Display Report

Analysis Info

Analysis Name	D:\Data\nctu service\data\2022\20221122\1_GB6_01_33156.d	Acquisition Date	11/22/2022 10:04:49 PM
Method	Small molecule.m	Operator	NCTU
Sample Name	1	Instrument	impact HD 1819696.00164
Comment			

Acquisition Parameter

Source Type	ESI	Ion Polarity	Positive	Set Nebulizer	1.0 Bar
Focus	Active	Set Capillary	4500 V	Set Dry Heater	200 °C
Scan Begin	50 m/z	Set End Plate Offset	-500 V	Set Dry Gas	6.0 l/min
Scan End	1500 m/z	Set Charging Voltage	2000 V	Set Divert Valve	Waste
		Set Corona	0 nA	Set APCI Heater	0 °C

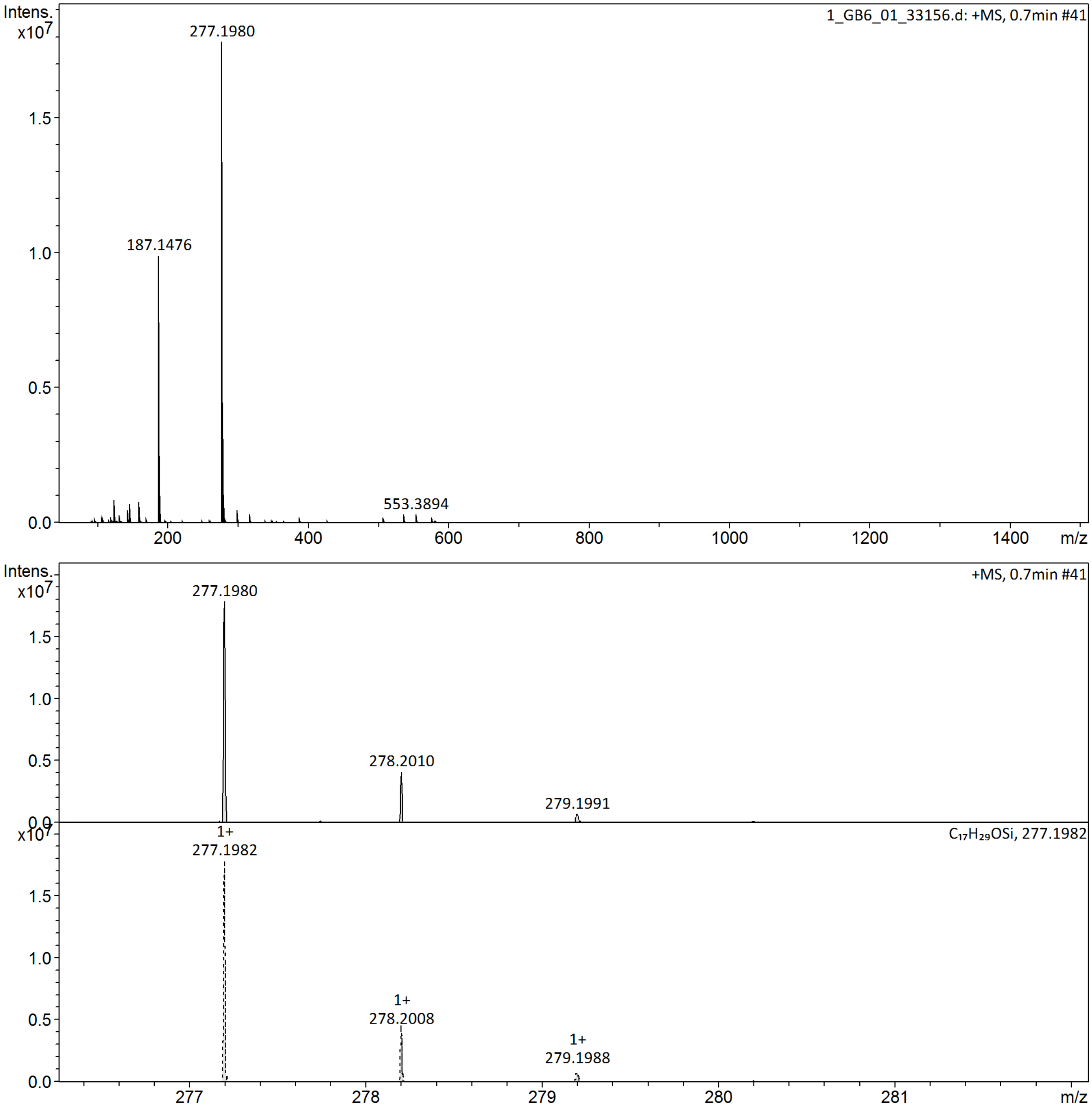

Display Report

Meas. m/z	#	Ion Formula	m/z	err [ppm]	mSigma	# Sigma	Score	rdb	e⁻ Conf	N-Rule	Adduct
277.1980	1	C17H29OSi	277.1982	-0.7	11.2	1	100.00	4.5	even	ok	M+H

+MS, 0.7min #41

#	m/z	Res.	S/N	I	I %	FWHM
1	91.0545	15276	50.5	30506	0.2	0.0060
2	93.0698	16165	124.9	75550	0.4	0.0058
3	95.0855	17278	228.8	138744	0.8	0.0055
4	97.0646	16198	48.2	29278	0.2	0.0060
5	105.0697	18001	271.8	165548	0.9	0.0058
6	107.0852	17537	222.0	135678	0.8	0.0061
7	109.1008	15927	31.1	19006	0.1	0.0069
8	116.1066	17779	148.5	91242	0.5	0.0065
9	117.0704	14165	45.1	27746	0.2	0.0083
10	118.0859	16985	43.5	26780	0.2	0.0070
11	119.0851	18142	205.2	126378	0.7	0.0066
12	122.0962	17103	47.0	29050	0.2	0.0071
13	123.0802	20427	1374.6	849276	4.8	0.0060
14	124.0837	17299	127.9	79006	0.4	0.0072
15	127.0723	17857	74.1	45852	0.3	0.0071
16	129.0707	15873	32.0	19814	0.1	0.0081
17	131.0851	19227	456.7	282702	1.6	0.0068
18	132.0885	15984	54.5	33736	0.2	0.0083
19	133.1005	17785	82.4	51006	0.3	0.0075
20	143.0881	20822	731.5	454990	2.6	0.0069
21	144.0904	15360	89.4	55682	0.3	0.0094
22	145.1009	20693	1106.3	689196	3.9	0.0070
23	146.1041	18332	129.8	80798	0.5	0.0080
24	147.0912	16750	46.0	28614	0.2	0.0088
25	147.1161	17601	110.7	68820	0.4	0.0084
26	149.0227	18198	32.6	20308	0.1	0.0082
27	159.1165	22139	1225.0	762906	4.3	0.0072
28	160.1195	18897	148.2	92298	0.5	0.0085
29	161.1315	17307	59.4	37022	0.2	0.0093
30	163.1112	18083	35.1	21856	0.1	0.0090
31	169.1036	19223	200.2	124218	0.7	0.0088
32	181.1400	18827	39.4	24174	0.1	0.0096
33	187.1126	9528	69.7	42288	0.2	0.0196
34	187.1476	29244	16314.6	9892782	55.6	0.0064
35	187.1730	15854	59.9	36332	0.2	0.0118
36	188.1513	24310	2231.5	1351184	7.6	0.0077
37	189.1545	18304	151.5	91542	0.5	0.0103
38	195.1193	20529	155.0	93110	0.5	0.0095
39	197.1345	19752	75.1	45018	0.3	0.0100
40	203.1416	17097	34.1	20270	0.1	0.0119
41	205.1579	19431	48.9	29002	0.2	0.0106
42	219.1731	19731	30.9	18092	0.1	0.0111
43	221.1350	19584	122.1	71294	0.4	0.0113
44	249.1662	20515	183.5	105134	0.6	0.0121
45	250.1690	17242	37.8	21614	0.1	0.0145
46	257.1892	18077	34.6	19394	0.1	0.0142
47	259.1876	18686	153.4	85674	0.5	0.0139
48	260.1899	17875	36.5	20358	0.1	0.0146
49	261.1666	18991	171.6	95600	0.5	0.0138
50	262.1694	17846	41.3	22960	0.1	0.0147
51	277.1661	7577	105.4	56200	0.3	0.0366
52	277.1980	29236	33379.2	17800370	100.0	0.0095
53	277.2368	10928	58.3	31098	0.2	0.0254
54	277.7404	20993	189.6	101008	0.6	0.0132
55	278.2010	32435	7753.9	4126478	23.2	0.0086
56	279.1991	18571	1352.8	718810	4.0	0.0150
57	280.1996	15788	194.6	103158	0.6	0.0177
58	282.2786	21728	198.7	104856	0.6	0.0130
59	283.2819	18839	39.7	20864	0.1	0.0150

#	m/z	Res.	S/N	I	I %	FWHM
1	277.1982	29236		17800370	100.0	0.0095
2	278.2008	29341		4236551	23.8	0.0095
3	279.1988	14723		1096770	6.2	0.0190
4	280.1984	29552		136314	0.8	0.0095

#	m/z	Res.	S/N	I	I %	FWHM
60	291.2135	18454	48.4	24862	0.1	0.0158
61	299.1802	23553	934.8	467702	2.6	0.0127
62	300.1824	20126	202.9	101282	0.6	0.0149
63	301.1803	14741	48.1	23934	0.1	0.0204
64	317.1905	23767	661.5	315432	1.8	0.0133
65	318.1929	20446	147.1	69954	0.4	0.0156
66	338.3414	20833	212.6	94508	0.5	0.0162
67	339.3452	21325	55.0	24368	0.1	0.0159
68	347.2398	20564	184.4	79960	0.4	0.0169
69	348.2421	20687	52.1	22546	0.1	0.0168
70	349.2412	15755	164.3	70924	0.4	0.0222
71	350.2425	16428	56.8	24444	0.1	0.0213
72	354.1188	20737	89.9	37878	0.2	0.0171
73	365.2498	19015	81.6	32558	0.2	0.0192
74	387.2322	22164	593.0	212754	1.2	0.0175
75	388.2349	19158	152.8	54546	0.3	0.0203
76	413.1941	17309	56.2	18318	0.1	0.0239
77	427.3018	21520	262.8	83872	0.5	0.0199
78	428.3046	20127	87.0	27748	0.2	0.0213
79	459.3283	19577	87.1	27380	0.2	0.0235
80	495.3643	21068	78.3	24506	0.1	0.0235
81	499.3209	20006	73.4	22986	0.1	0.0250
82	506.5295	22119	609.5	190736	1.1	0.0229
83	507.5326	21015	221.0	69102	0.4	0.0242
84	508.5413	18016	81.4	25474	0.1	0.0282
85	519.3468	17872	72.4	22706	0.1	0.0291
86	535.3786	23675	970.6	304912	1.7	0.0226
87	536.3810	22143	454.1	142690	0.8	0.0242
88	537.3768	13537	182.1	57238	0.3	0.0397
89	553.3894	23955	980.0	308156	1.7	0.0231
90	554.3917	21909	466.7	146768	0.8	0.0253
91	555.3917	19405	166.6	52386	0.3	0.0286
92	572.3621	19979	76.6	24062	0.1	0.0286
93	572.8633	20714	71.6	22480	0.1	0.0277
94	573.3626	21866	63.3	19892	0.1	0.0262
95	575.3710	22326	669.3	210366	1.2	0.0258
96	576.3740	20796	323.2	101632	0.6	0.0277
97	577.3729	16547	109.2	34348	0.2	0.0349
98	580.3481	21292	178.0	55880	0.3	0.0273
99	580.8494	22486	164.8	51754	0.3	0.0258
100	581.3497	21582	177.3	55682	0.3	0.0269
101	581.8497	20637	101.3	31790	0.2	0.0282
102	582.3491	21794	86.8	27240	0.2	0.0267
103	623.3922	17912	68.5	21026	0.1	0.0348
104	753.5466	18378	80.3	21702	0.1	0.0410
