# Supplementary material for: Diastereodivergent Access to cis,cis- and cis,trans-Angular Triquinanes: Total Synthesis of (±)-Arnicenone
Source: J Am Chem Soc. 2026 Sep 22;148(39):41724–32. doi: 10.1021/jacs.6c09019 (PMC13651531; doi:10.1021/jacs.6c09019)
Supplement: Supplementary file 4 [file ja6c09019_si_002.zip › FID 28/e cis trans/HTH-DMPS alpha demethyl trans555-MS.pdf]

Display Report

Analysis Info

Analysis Name	D:\Data\nctu service\data\2022\20221216\11HTH-alpha dimethyl DMPS tran_GB4_01_33719.d	Acquisition Date	12/19/2022 2:56:05 PM
Method	Small molecule.m	Operator	NCTU
Sample Name	11HTH-alpha dimethyl DMPS tran	Instrument	impact HD 1819696.00164
Comment			

Acquisition Parameter

Source Type	ESI	Ion Polarity	Positive	Set Nebulizer	1.0 Bar
Focus	Active	Set Capillary	4500 V	Set Dry Heater	200 °C
Scan Begin	50 m/z	Set End Plate Offset	-500 V	Set Dry Gas	6.0 l/min
Scan End	1500 m/z	Set Charging Voltage	2000 V	Set Divert Valve	Waste
		Set Corona	0 nA	Set APCI Heater	0 °C

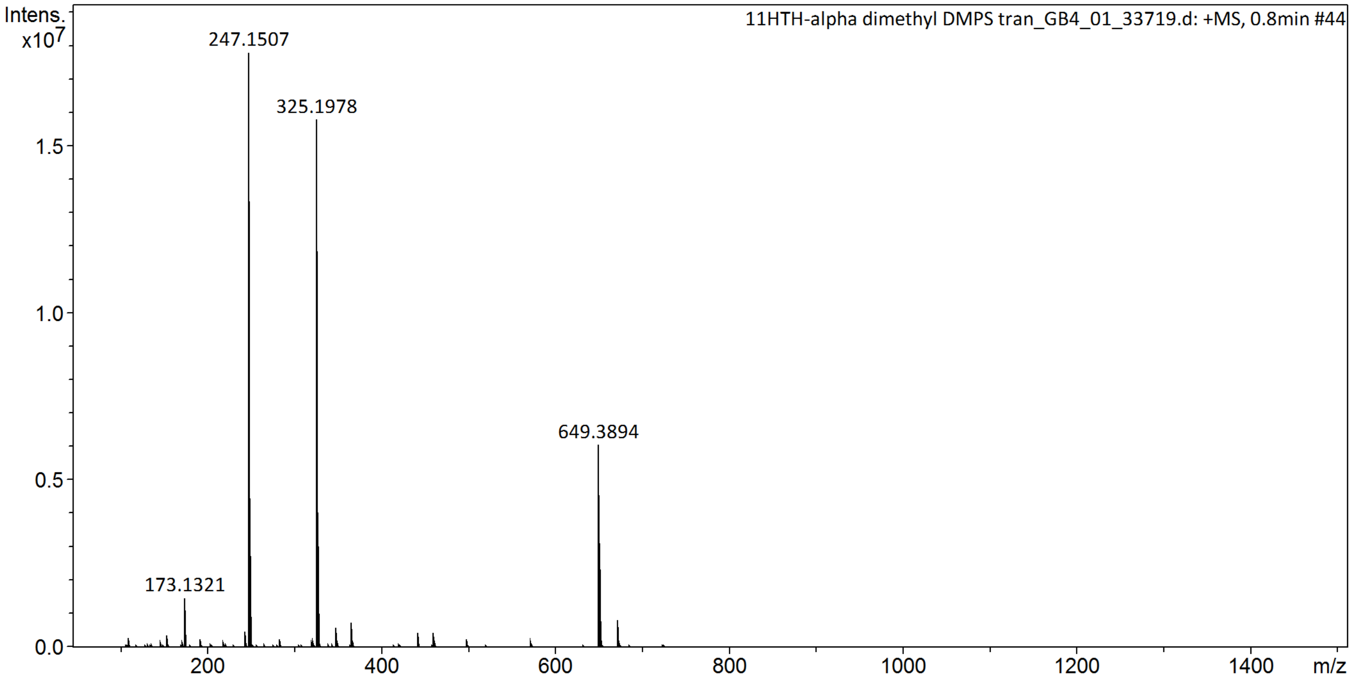

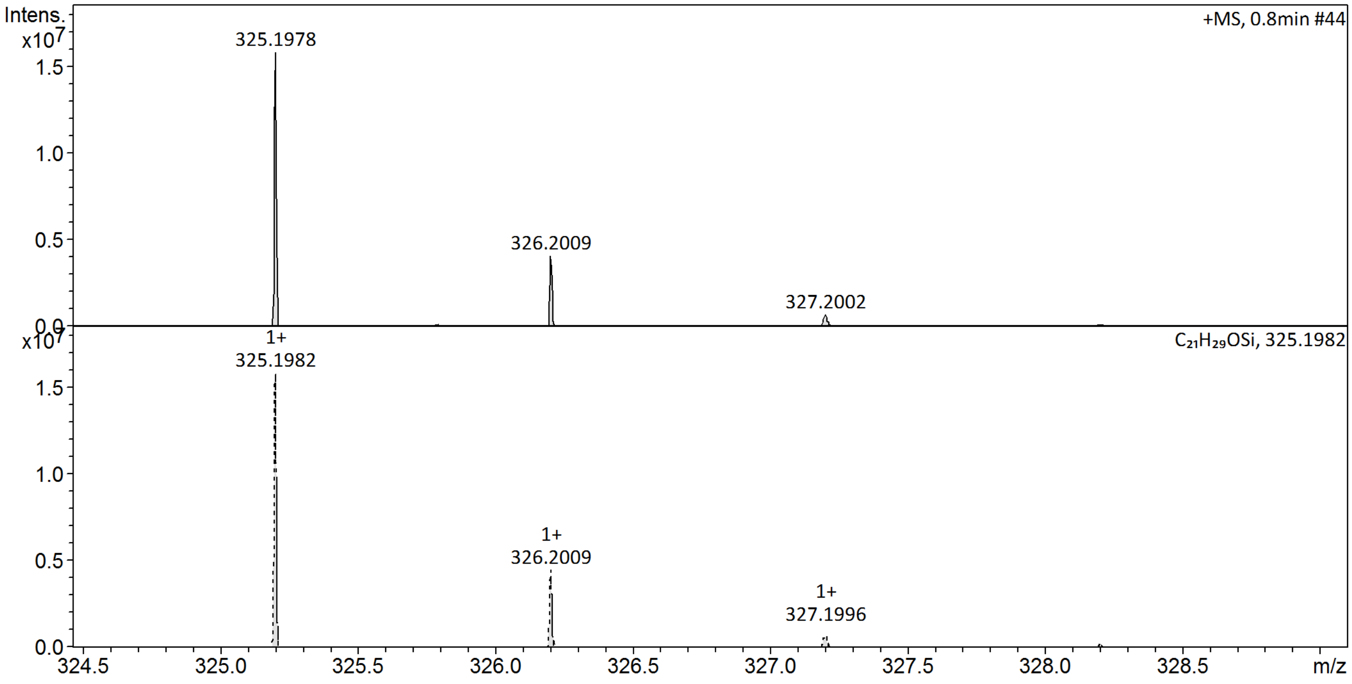

Display Report

Meas. m/z	#	Ion Formula	m/z	err [ppm]	mSigma	# Sigma	Score	rdb	e¯ Conf	N-Rule	Adduct
325.1978	1	C21H29OSi	325.1982	-1.1	19.9	1	100.00	8.5	even	ok	M+H

+MS, 0.8min #44

#	m/z	Res.	S/N	I	I %	FWHM
1	93.0364	16441	31.3	18080	0.1	0.0057
2	93.0699	15390	33.6	19368	0.1	0.0060
3	105.0695	16479	50.6	29644	0.2	0.0064
4	107.0851	16670	79.9	46988	0.3	0.0064
5	109.0645	17439	475.4	279636	1.6	0.0063
6	110.0678	16898	43.1	25368	0.1	0.0065
7	117.0696	17212	59.2	35174	0.2	0.0068
8	122.0958	16327	31.7	18942	0.1	0.0075
9	127.0721	17567	46.7	28064	0.2	0.0072
10	127.1476	16971	86.0	51644	0.3	0.0075
11	131.0849	17988	155.2	93672	0.5	0.0073
12	133.1005	18090	78.6	47640	0.3	0.0074
13	135.0619	17447	117.3	71256	0.4	0.0077
14	143.0849	17132	36.1	22118	0.1	0.0084
15	145.1005	18963	192.4	118362	0.7	0.0077
16	147.1160	17093	30.4	18728	0.1	0.0086
17	149.0227	17393	60.2	37212	0.2	0.0086
18	153.0725	20668	593.6	367868	2.1	0.0074
19	154.0745	15731	69.6	43200	0.2	0.0098
20	165.0723	17497	36.4	22706	0.1	0.0094
21	169.1027	17131	56.7	35450	0.2	0.0099
22	171.0829	18565	135.5	84766	0.5	0.0092
23	171.1161	19538	181.0	113250	0.6	0.0088
24	172.1191	19406	31.2	19536	0.1	0.0089
25	173.1321	24350	2344.4	1468060	8.3	0.0071
26	174.1352	19425	309.7	193866	1.1	0.0090
27	177.0725	18981	29.9	18772	0.1	0.0093
28	179.0879	19462	88.8	55706	0.3	0.0092
29	191.0880	21094	375.1	238064	1.3	0.0091
30	192.0904	17715	63.3	40258	0.2	0.0108
31	203.1243	20326	106.2	68404	0.4	0.0100
32	205.1032	17570	44.9	28962	0.2	0.0117
33	207.1185	18319	31.6	20316	0.1	0.0113
34	217.1039	19097	39.1	25408	0.1	0.0114
35	217.1401	19073	174.0	113178	0.6	0.0114
36	218.1426	18265	36.5	23706	0.1	0.0119
37	219.1191	20056	70.7	46000	0.3	0.0109
38	221.1347	20151	114.1	74242	0.4	0.0110
39	223.1141	17826	41.4	26970	0.2	0.0125
40	229.1402	18864	61.1	39608	0.2	0.0121
41	237.1300	17764	29.8	19066	0.1	0.0133
42	242.2834	19911	143.1	90738	0.5	0.0122
43	243.1195	23649	712.3	451354	2.5	0.0103
44	243.1551	17123	64.5	40860	0.2	0.0142
45	244.1219	19049	140.5	88856	0.5	0.0128
46	247.1208	12816	75.8	47716	0.3	0.0193
47	247.1507	28437	28261.9	17786024	100.0	0.0087
48	247.1860	12148	45.6	28696	0.2	0.0203
49	247.6631	20661	150.0	94334	0.5	0.0120
50	248.1535	31550	5830.1	3664026	20.6	0.0079
51	249.1520	16041	934.7	586338	3.3	0.0155
52	250.1529	15074	137.6	86112	0.5	0.0166
53	251.1780	19479	48.0	29966	0.2	0.0129
54	256.2627	18855	61.8	38258	0.2	0.0136
55	265.1612	20547	138.5	85452	0.5	0.0129
56	275.1631	20994	76.8	47370	0.3	0.0131
57	280.2628	21117	54.7	33506	0.2	0.0133
58	282.2785	21709	404.6	247180	1.4	0.0130
59	283.2818	18876	79.5	48514	0.3	0.0150

#	m/z	Res.	S/N	I	I %	FWHM
1	325.1982	36938		15773298	100.0	0.0088
2	326.2009	37052		4436500	28.1	0.0088
3	327.1996	18583		1145356	7.3	0.0176
4	328.2007	18640		179122	1.1	0.0176

Display Report

#	m/z	Res.	S/N	I	I %	FWHM
60	288.1773	20036	44.6	27114	0.2	0.0144
61	293.1743	18934	43.8	26462	0.1	0.0155
62	304.2607	19812	64.2	38082	0.2	0.0154
63	307.1872	18851	54.5	32176	0.2	0.0163
64	319.2447	20363	190.7	108824	0.6	0.0157
65	320.2473	17802	51.4	29158	0.2	0.0180
66	321.1696	21911	290.3	163802	0.9	0.0147
67	322.1715	19510	76.4	42856	0.2	0.0165
68	325.1620	9017	80.4	44668	0.3	0.0361
69	325.1978	36938	28376.1	15773298	88.7	0.0088
70	325.2392	11667	69.2	38490	0.2	0.0279
71	325.7854	22946	128.3	71270	0.4	0.0142
72	326.2009	35676	7285.4	4043790	22.7	0.0091
73	327.2002	20882	1207.8	668820	3.8	0.0157
74	328.2001	17009	193.7	106928	0.6	0.0193
75	338.3413	20824	160.9	85748	0.5	0.0162
76	339.3443	19629	41.3	21916	0.1	0.0173
77	343.2084	19914	154.0	80298	0.5	0.0172
78	344.2110	18276	45.7	23732	0.1	0.0188
79	347.1801	25933	1100.5	564900	3.2	0.0134
80	348.1824	21231	291.8	149242	0.8	0.0164
81	349.1810	15703	61.0	31082	0.2	0.0222
82	363.1534	18145	61.6	29870	0.2	0.0200
83	365.1908	26988	1557.8	753566	4.2	0.0135
84	366.1933	21542	412.9	199252	1.1	0.0170
85	367.1919	15989	93.6	45070	0.3	0.0230
86	399.2164	19811	46.0	20596	0.1	0.0202
87	413.2654	21284	76.2	32208	0.2	0.0194
88	419.3150	21386	222.9	92672	0.5	0.0196
89	420.3184	20312	63.7	26420	0.1	0.0207
90	421.1985	19875	84.4	34960	0.2	0.0212
91	441.2975	25551	1151.0	447846	2.5	0.0173
92	442.3006	22199	329.8	128274	0.7	0.0199
93	443.3039	19856	54.2	21032	0.1	0.0223
94	457.2709	19618	92.7	34202	0.2	0.0233
95	459.2534	24701	1131.0	415512	2.3	0.0186
96	460.2558	21489	455.2	166960	0.9	0.0214
97	461.2541	18709	152.4	55856	0.3	0.0247
98	495.3077	20447	80.0	27896	0.2	0.0242
99	497.3233	22947	715.2	249156	1.4	0.0217
100	498.3261	21919	307.9	107276	0.6	0.0227
101	499.3268	19011	87.9	30600	0.2	0.0263
102	508.2760	21688	55.6	19324	0.1	0.0234
103	519.3045	21833	98.7	34236	0.2	0.0238
104	537.3162	19748	55.3	19060	0.1	0.0272
105	571.3419	21499	422.1	144274	0.8	0.0266
106	572.3445	21017	213.7	73080	0.4	0.0272
107	573.3450	18283	79.6	27192	0.2	0.0314
108	631.3787	18733	89.6	29348	0.2	0.0337
109	643.4357	20633	85.9	27652	0.2	0.0312
110	649.3397	15055	95.6	30484	0.2	0.0431
111	649.3894	47449	18983.0	6055482	34.0	0.0137
112	649.4390	14292	286.7	91458	0.5	0.0454
113	650.3924	41282	9762.8	3111486	17.5	0.0158
114	651.3929	30129	3215.4	1023244	5.8	0.0216
115	652.3933	23158	888.3	282330	1.6	0.0282
116	653.3936	19189	183.0	58102	0.3	0.0340
117	670.3723	21857	67.6	20914	0.1	0.0307
118	670.8729	20456	66.8	20638	0.1	0.0328
119	671.3718	29797	2635.8	814304	4.6	0.0225
120	672.3740	25607	1406.1	433894	2.4	0.0263
121	673.3742	21855	530.9	163648	0.9	0.0308
122	674.3746	18921	146.2	45010	0.3	0.0356
123	676.3491	22143	80.4	24664	0.1	0.0305
124	676.8496	21698	86.8	26608	0.1	0.0312
125	677.3500	20298	71.3	21844	0.1	0.0334

#	m/z	Res.	S/N	I	I %	FWHM
126	677.8497	26194	60.0	18374	0.1	0.0259
127	684.3692	22910	109.7	33258	0.2	0.0299
128	684.8702	20210	124.8	37798	0.2	0.0339
129	685.3701	19539	92.4	27948	0.2	0.0351
130	723.4083	20367	183.4	50836	0.3	0.0355
131	724.4110	19421	121.7	33670	0.2	0.0373
132	830.4585	24983	95.6	20788	0.1	0.0332
133	830.9598	22294	113.1	24562	0.1	0.0373
134	831.4590	24480	103.9	22556	0.1	0.0340
135	859.6058	20621	89.9	18236	0.1	0.0417
