# Supplementary material for: Diastereodivergent Access to cis,cis- and cis,trans-Angular Triquinanes: Total Synthesis of (±)-Arnicenone
Source: J Am Chem Soc. 2026 Sep 22;148(39):41724–32. doi: 10.1021/jacs.6c09019 (PMC13651531; doi:10.1021/jacs.6c09019)
Supplement: Supplementary file 4 [file ja6c09019_si_002.zip › FID 28/e cis trans/HTH-DMPS alpha demethyl trans555-13C.pdf]

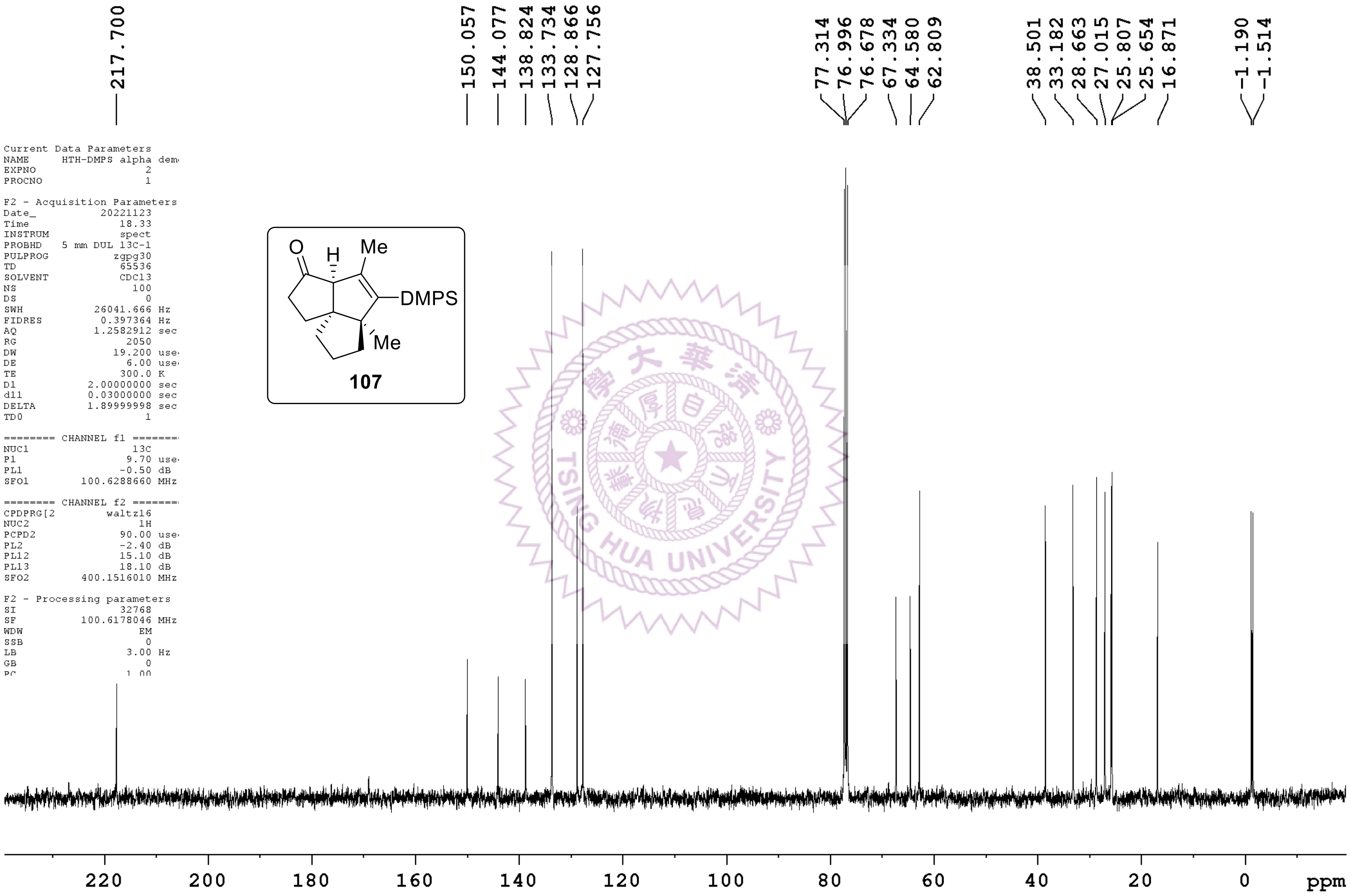

TSING HUA UNIVERSITY
