# Supplementary material for: Diastereodivergent Access to cis,cis- and cis,trans-Angular Triquinanes: Total Synthesis of (±)-Arnicenone
Source: J Am Chem Soc. 2026 Sep 22;148(39):41724–32. doi: 10.1021/jacs.6c09019 (PMC13651531; doi:10.1021/jacs.6c09019)
Supplement: Supplementary file 4 [file ja6c09019_si_002.zip › FID 28/e cis cis/HTH-DMPS alpha demethyl cis555-13C.pdf]

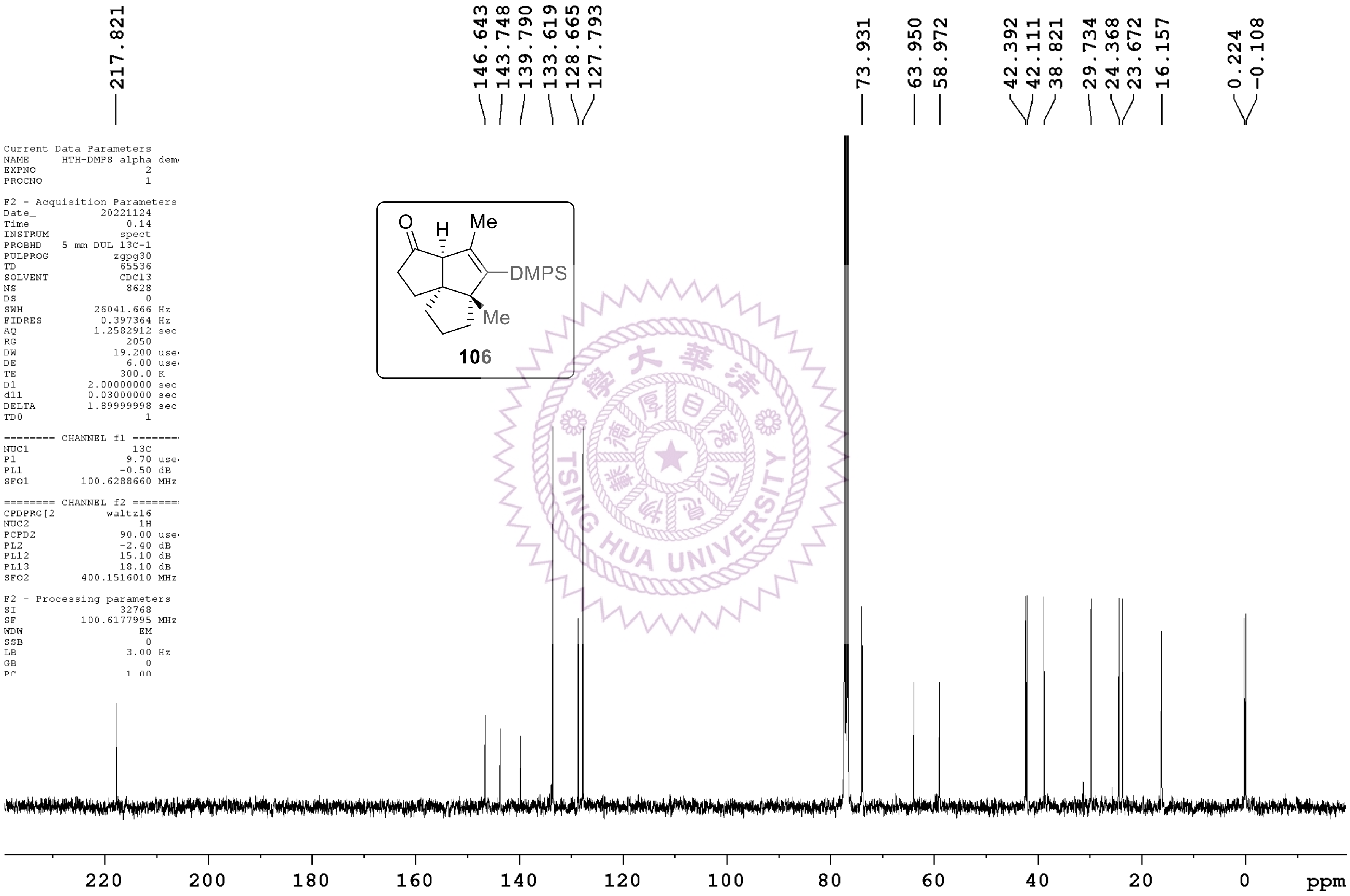
217.821
146.643
143.748
139.790
133.619
128.665
127.793
73.931
63.950
58.972
42.392
42.111
38.821
29.734
24.368
23.672
16.157
0.224
-0.108
Current Data Parameters
NAME HTH-DMPS alpha dem
EXPNO 2
PROCNO 1
F2 - Acquisition Parameters
Date_ 20221124
Time 0.14
INSTRUM spect
PROBHD 5 mm DUL 13C-1
PULPROG zgpg30
TD 65536
SOLVENT CDCl3
NS 8628
DS 0
SWH 26041.666 Hz
FIDRES 0.397364 Hz
AQ 1.2582912 sec
RG 2050
DW 19.200 use
DE 6.00 use
TE 300.0 K
D1 2.00000000 sec
d11 0.03000000 sec
DELTA 1.89999998 sec
TD0 1
======== CHANNEL f1 ========
NUC1 13C
P1 9.70 use
PL1 -0.50 dB
SFO1 100.6288660 MHz
======== CHANNEL f2 ========
CPDPRG[2 waltz16
NUC2 1H
PCPD2 90.00 use
PL2 -2.40 dB
PL12 15.10 dB
PL13 18.10 dB
SFO2 400.1516010 MHz
F2 - Processing parameters
SI 32768
SF 100.6177995 MHz
WDW EM
SSB 0
LB 3.00 Hz
GB 0
O
H
Me
DMPS
Me
106
TSING HUA UNIVERSITY
220
200
180
160
140
120
100
80
60
40
20
0
ppm
