# Supplementary material for: Diastereodivergent Access to cis,cis- and cis,trans-Angular Triquinanes: Total Synthesis of (±)-Arnicenone
Source: J Am Chem Soc. 2026 Sep 22;148(39):41724–32. doi: 10.1021/jacs.6c09019 (PMC13651531; doi:10.1021/jacs.6c09019)

```
Current Data Parameters
NAME     HTH-DMPS alpha demet
EXPNO                    1
PROCNO                   1

F2 - Acquisition Parameters
Date_             20221123
Time                 17.34
INSTRUM              spect
PROBHD   5 mm DUL 13C-1
PULPROG               zg30
TD                   32768
SOLVENT              CDCl3
NS                      16
DS                       0
SWH               6410.256 Hz
FIDRES            0.195625 Hz
AQ               2.5559039 sec
RG                     724
DW                  78.000 usec
DE                    6.00 usec
TE                   300.0 K
D1              2.00000000 sec
TD0                      1

======== CHANNEL f1 ========
NUC1                    1H
P1                   10.00 usec
PL1                  -2.40 dB
SFO1           400.1528010 MHz

F2 - Processing parameters
SI                   16384
SF             400.1500174 MHz
WDW                     EM
SSB                      0
LB                       0 Hz
GB                       0
PC                    1.00
```

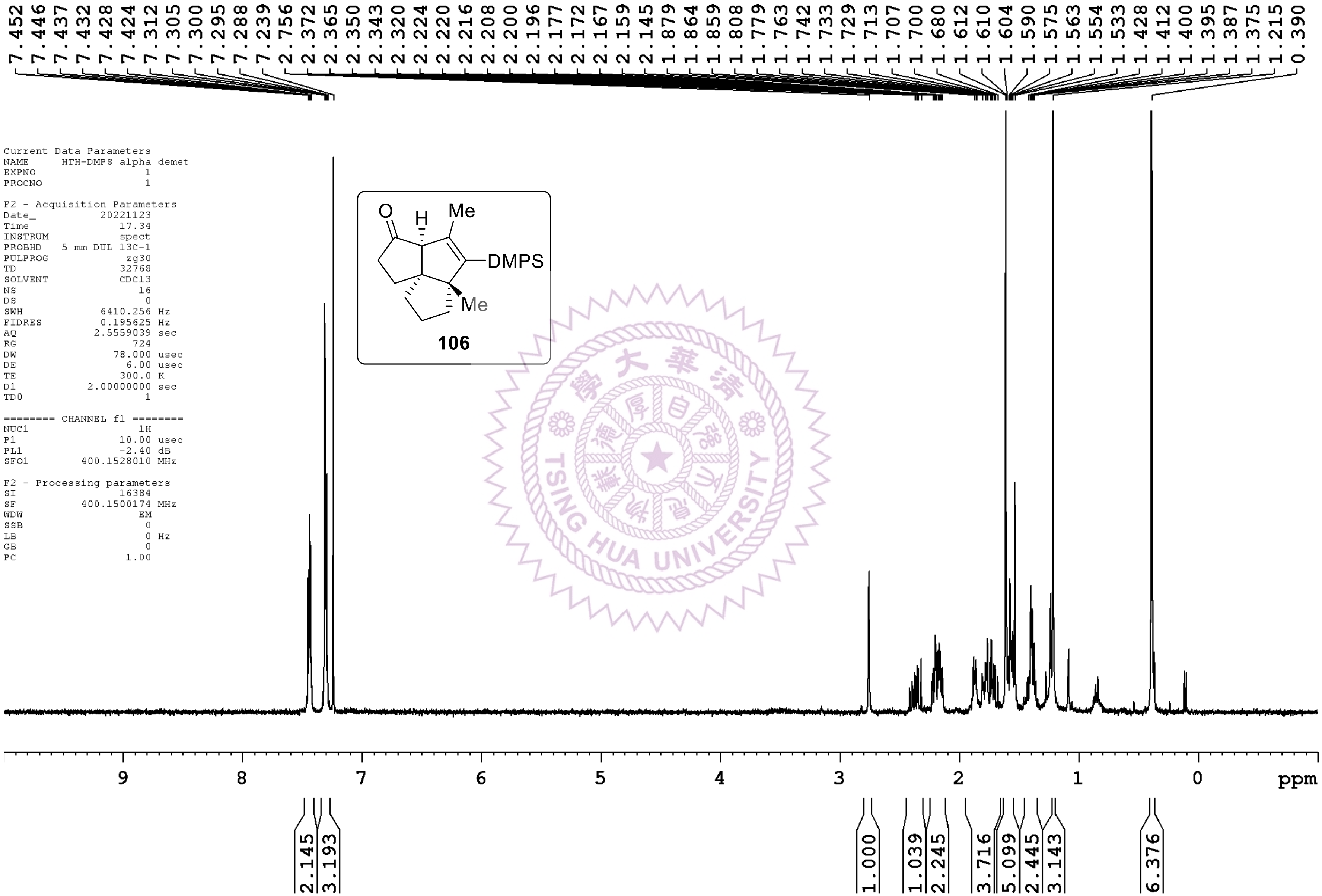

Supplement: Supplementary file 4 [file ja6c09019_si_002.zip › FID 28/e cis cis/HTH-DMPS alpha demethyl cis555-1H.pdf]
