# Supplementary material for: Diastereodivergent Access to cis,cis- and cis,trans-Angular Triquinanes: Total Synthesis of (±)-Arnicenone
Source: J Am Chem Soc. 2026 Sep 22;148(39):41724–32. doi: 10.1021/jacs.6c09019 (PMC13651531; doi:10.1021/jacs.6c09019)
Supplement: Supplementary file 4 [file ja6c09019_si_002.zip › FID 28/e cis cis/HTH-DMPS alpha demethyl cis555-MS.pdf]

Display Report

Analysis Info

Analysis Name	D:\Data\nctu service\data\2022\20221216\10HTH-alpha dimethyl DMPS cis _GB3_01_33718.d	Acquisition Date	12/19/2022 2:51:26 PM
Method	Small molecule.m	Operator	NCTU
Sample Name	10HTH-alpha dimethyl DMPS cis	Instrument	impact HD 1819696.00164
Comment			

Acquisition Parameter

Source Type	ESI	Ion Polarity	Positive	Set Nebulizer	1.0 Bar
Focus	Active	Set Capillary	4500 V	Set Dry Heater	200 °C
Scan Begin	50 m/z	Set End Plate Offset	-500 V	Set Dry Gas	6.0 l/min
Scan End	1500 m/z	Set Charging Voltage	2000 V	Set Divert Valve	Waste
		Set Corona	0 nA	Set APCI Heater	0 °C

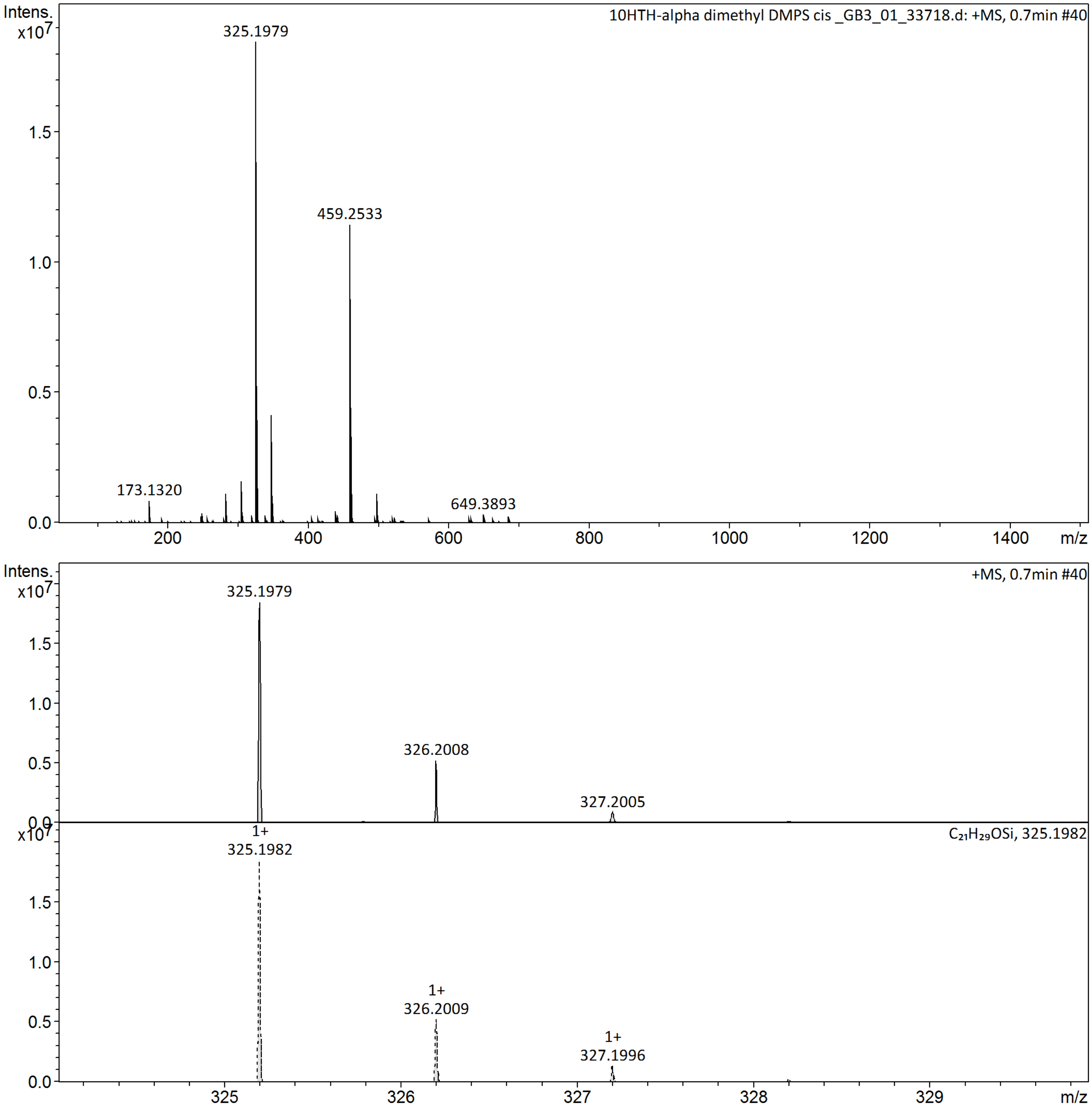

Display Report

Meas. m/z	#	Ion Formula	m/z	err [ppm]	mSigma	# Sigma	Score	rdb	e⁻ Conf	N-Rule	Adduct
325.1979	1	C21H29OSi	325.1982	0.9	13.2	1	100.00	8.5	even	ok	M+H

+MS, 0.7min #40

#	m/z	Res.	S/N	I	I %	FWHM
1	109.1008	17359	39.4	18628	0.1	0.0063
2	122.0963	15614	37.4	18594	0.1	0.0078
3	127.1477	17349	59.2	29978	0.2	0.0073
4	131.0848	17744	55.4	28540	0.2	0.0074
5	133.1032	16349	96.6	50104	0.3	0.0081
6	145.1005	17789	109.8	59466	0.3	0.0082
7	147.1161	17776	51.7	28196	0.2	0.0083
8	149.0225	19099	156.2	85630	0.5	0.0078
9	153.0723	18379	158.7	87858	0.5	0.0083
10	159.0643	19813	64.3	36222	0.2	0.0080
11	167.0332	17777	53.0	30570	0.2	0.0094
12	171.1184	15786	33.6	19504	0.1	0.0108
13	173.1320	23094	1463.5	855412	4.6	0.0075
14	174.1352	18649	189.4	110902	0.6	0.0093
15	175.1463	16808	62.1	36440	0.2	0.0104
16	187.1472	17750	47.3	28506	0.2	0.0105
17	191.0878	20226	126.1	76554	0.4	0.0094
18	191.1423	19318	188.0	114074	0.6	0.0099
19	200.2001	19281	81.0	49796	0.3	0.0104
20	205.1579	17452	33.9	20998	0.1	0.0118
21	219.1735	19944	78.0	49316	0.3	0.0110
22	223.1502	19025	58.2	36968	0.2	0.0117
23	228.2311	20762	42.2	26978	0.1	0.0110
24	233.1892	20358	52.0	33384	0.2	0.0115
25	243.1198	15808	29.3	18958	0.1	0.0154
26	247.1507	22174	367.3	239098	1.3	0.0111
27	247.2411	18980	46.9	30570	0.2	0.0130
28	248.1533	18875	75.8	49380	0.3	0.0131
29	249.1660	21787	568.6	370734	2.0	0.0114
30	250.1690	18719	126.6	82662	0.4	0.0134
31	256.2629	20133	262.5	172842	0.9	0.0127
32	257.2659	19382	49.8	32798	0.2	0.0133
33	263.2361	20497	58.7	39044	0.2	0.0128
34	265.1614	19457	56.0	37532	0.2	0.0136
35	265.2517	20648	111.9	74996	0.4	0.0128
36	274.2735	18910	27.8	19292	0.1	0.0145
37	275.1636	18133	27.1	18830	0.1	0.0152
38	277.1967	16648	29.4	20442	0.1	0.0166
39	280.2629	20703	170.0	118610	0.6	0.0135
40	281.2667	17609	32.2	22484	0.1	0.0160
41	282.2788	27832	1614.4	1127910	6.1	0.0101
42	283.2818	21522	303.4	212326	1.2	0.0132
43	284.2921	17541	98.8	69420	0.4	0.0162
44	287.1823	18972	37.2	26564	0.1	0.0151
45	290.2680	19425	46.8	33782	0.2	0.0149
46	293.1741	17699	33.5	24206	0.1	0.0166
47	301.1403	18696	72.0	52834	0.3	0.0161
48	302.2445	20692	59.3	43708	0.2	0.0146
49	304.2606	20965	242.5	180202	1.0	0.0145
50	305.2294	29934	2160.1	1607744	8.7	0.0102
51	306.2319	22227	484.6	361258	2.0	0.0138
52	307.1872	19976	90.1	67238	0.4	0.0154
53	307.2301	15632	104.3	77892	0.4	0.0197
54	308.1900	18206	26.6	19860	0.1	0.0169
55	319.2448	22796	388.1	295284	1.6	0.0140
56	320.2475	20141	107.7	81956	0.4	0.0159
57	321.2460	13175	26.7	20384	0.1	0.0244
58	325.1620	11850	78.3	59782	0.3	0.0274
59	325.1979	28317	24159.7	18446184	100.0	0.0115

#	m/z	Res.	S/N	I	I %	FWHM
1	325.1982	28317		18446184	100.0	0.0115
2	326.2009	28404		5188293	28.1	0.0115
3	327.1996	28491		1339444	7.3	0.0115
4	328.2007	28578		209476	1.1	0.0115

Display Report

#	m/z	Res.	S/N	I	I %	FWHM
60	325.2414	13188	55.1	42080	0.2	0.0247
61	325.5180	18654	32.5	24812	0.1	0.0175
62	325.7857	22238	163.7	124984	0.7	0.0147
63	326.2008	36838	6852.2	5230052	28.4	0.0089
64	327.2005	21904	1130.1	862102	4.7	0.0149
65	328.2006	16647	176.0	134320	0.7	0.0197
66	338.3413	24084	398.4	302514	1.6	0.0140
67	339.1787	14981	27.2	20662	0.1	0.0226
68	339.2130	17214	29.7	22562	0.1	0.0197
69	339.3446	20170	92.5	70214	0.4	0.0168
70	342.2243	21712	122.7	93030	0.5	0.0158
71	343.2258	18067	37.5	28394	0.2	0.0190
72	347.1801	36788	5484.3	4144946	22.5	0.0094
73	348.1829	28911	1320.8	996984	5.4	0.0120
74	349.1816	17693	264.5	199456	1.1	0.0197
75	350.1823	17347	47.3	35678	0.2	0.0202
76	360.3232	22850	69.6	52084	0.3	0.0158
77	363.1541	20261	126.8	95246	0.5	0.0179
78	364.1574	18355	37.4	28070	0.2	0.0198
79	365.2287	21340	58.7	44156	0.2	0.0171
80	381.3007	17503	33.0	24834	0.1	0.0218
81	393.2973	19472	36.2	27528	0.1	0.0202
82	399.2142	19505	89.8	68212	0.4	0.0205
83	400.2172	19952	28.6	21714	0.1	0.0201
84	405.2603	21769	204.3	154380	0.8	0.0186
85	406.2633	20959	71.4	53920	0.3	0.0194
86	413.2659	23676	246.2	182396	1.0	0.0175
87	414.2691	21428	65.7	48682	0.3	0.0193
88	416.3340	20419	41.4	30602	0.2	0.0204
89	419.2763	19721	50.1	36844	0.2	0.0213
90	419.3142	19539	75.3	55368	0.3	0.0215
91	421.1986	21274	48.0	35372	0.2	0.0198
92	421.3283	19490	26.3	19340	0.1	0.0216
93	439.2847	26042	631.2	449124	2.4	0.0169
94	440.2869	21178	234.9	166988	0.9	0.0208
95	441.2964	22111	430.8	306388	1.7	0.0200
96	442.2999	19577	115.2	81916	0.4	0.0226
97	445.1198	20650	40.5	28768	0.2	0.0216
98	447.3440	18544	26.4	18652	0.1	0.0241
99	453.2995	18970	32.9	22768	0.1	0.0239
100	457.2708	18204	33.6	22986	0.1	0.0251
101	459.2095	15033	61.6	42018	0.2	0.0305
102	459.2533	44455	16782.6	11444848	62.0	0.0103
103	459.3000	13367	79.7	54332	0.3	0.0344
104	459.9521	22603	45.1	30758	0.2	0.0203
105	460.2193	11324	30.6	20838	0.1	0.0406
106	460.2560	40899	6508.6	4433314	24.0	0.0113
107	461.2554	29701	1879.9	1279328	6.9	0.0155
108	462.2556	21442	379.9	257954	1.4	0.0216
109	463.2558	17322	75.2	51006	0.3	0.0267
110	467.1019	21182	27.6	18614	0.1	0.0221
111	495.3076	22589	285.9	185460	1.0	0.0219
112	496.3105	21531	123.0	79758	0.4	0.0231
113	497.3236	31621	1699.4	1102132	6.0	0.0157
114	498.3265	26031	679.7	440350	2.4	0.0191
115	499.3271	19236	177.8	115124	0.6	0.0260
116	500.3285	18004	35.1	22736	0.1	0.0278
117	503.2531	19993	30.7	19806	0.1	0.0252
118	506.5285	21133	77.3	49790	0.3	0.0240
119	507.5317	20332	33.3	21406	0.1	0.0250
120	517.2897	20627	53.1	33868	0.2	0.0251
121	519.1392	20086	38.9	24796	0.1	0.0258
122	519.3049	22047	238.7	151994	0.8	0.0236
123	520.3075	21581	101.1	64348	0.3	0.0241
124	521.3082	18002	29.4	18692	0.1	0.0290
125	523.3242	23617	297.6	189084	1.0	0.0222

Display Report

#	m/z	Res.	S/N	I	I %	FWHM
126	524.3278	22394	88.0	55716	0.3	0.0234
127	531.2830	20670	81.2	50570	0.3	0.0257
128	532.2862	20001	36.5	22710	0.1	0.0266
129	533.2781	12981	106.2	65986	0.4	0.0411
130	534.2774	12921	49.9	30958	0.2	0.0413
131	535.2849	11952	50.7	31396	0.2	0.0448
132	536.1648	20381	54.3	33588	0.2	0.0263
133	537.1658	17855	30.7	18946	0.1	0.0301
134	537.3144	16992	41.8	25814	0.1	0.0316
135	539.2312	19105	31.5	19358	0.1	0.0282
136	539.3000	17946	31.7	19480	0.1	0.0301
137	563.5496	24625	32.3	18456	0.1	0.0229
138	571.3420	22690	251.8	140544	0.8	0.0252
139	572.3449	20181	117.0	65146	0.4	0.0284
140	573.3450	18993	47.7	26516	0.1	0.0302
141	606.4692	20455	46.6	22800	0.1	0.0296
142	629.4206	22890	395.0	182750	1.0	0.0275
143	630.4233	20949	213.5	98670	0.5	0.0301
144	631.3784	22401	386.3	178306	1.0	0.0282
145	632.3809	22168	213.7	98430	0.5	0.0285
146	633.3807	18368	83.6	38444	0.2	0.0345
147	643.4366	21212	52.8	23504	0.1	0.0303
148	649.3893	25104	711.8	310968	1.7	0.0259
149	650.3917	22819	387.0	168696	0.9	0.0285
150	651.3924	19887	146.8	63824	0.3	0.0328
151	652.3933	18547	43.3	18768	0.1	0.0352
152	663.4536	22048	336.1	139854	0.8	0.0301
153	664.4574	20254	153.6	63814	0.3	0.0328
154	665.3397	19209	45.1	18698	0.1	0.0346
155	671.3714	20968	162.7	66526	0.4	0.0320
156	672.3738	21040	87.2	35572	0.2	0.0320
157	685.4356	24152	629.1	246066	1.3	0.0284
158	686.4384	21453	287.9	112320	0.6	0.0320
159	687.4425	21277	72.0	28032	0.2	0.0323
