# Supplementary material for: Diastereodivergent Access to cis,cis- and cis,trans-Angular Triquinanes: Total Synthesis of (±)-Arnicenone
Source: J Am Chem Soc. 2026 Sep 22;148(39):41724–32. doi: 10.1021/jacs.6c09019 (PMC13651531; doi:10.1021/jacs.6c09019)
Supplement: Supplementary file 4 [file ja6c09019_si_002.zip › FID 28/f cis trans/2/180P-13C.pdf]

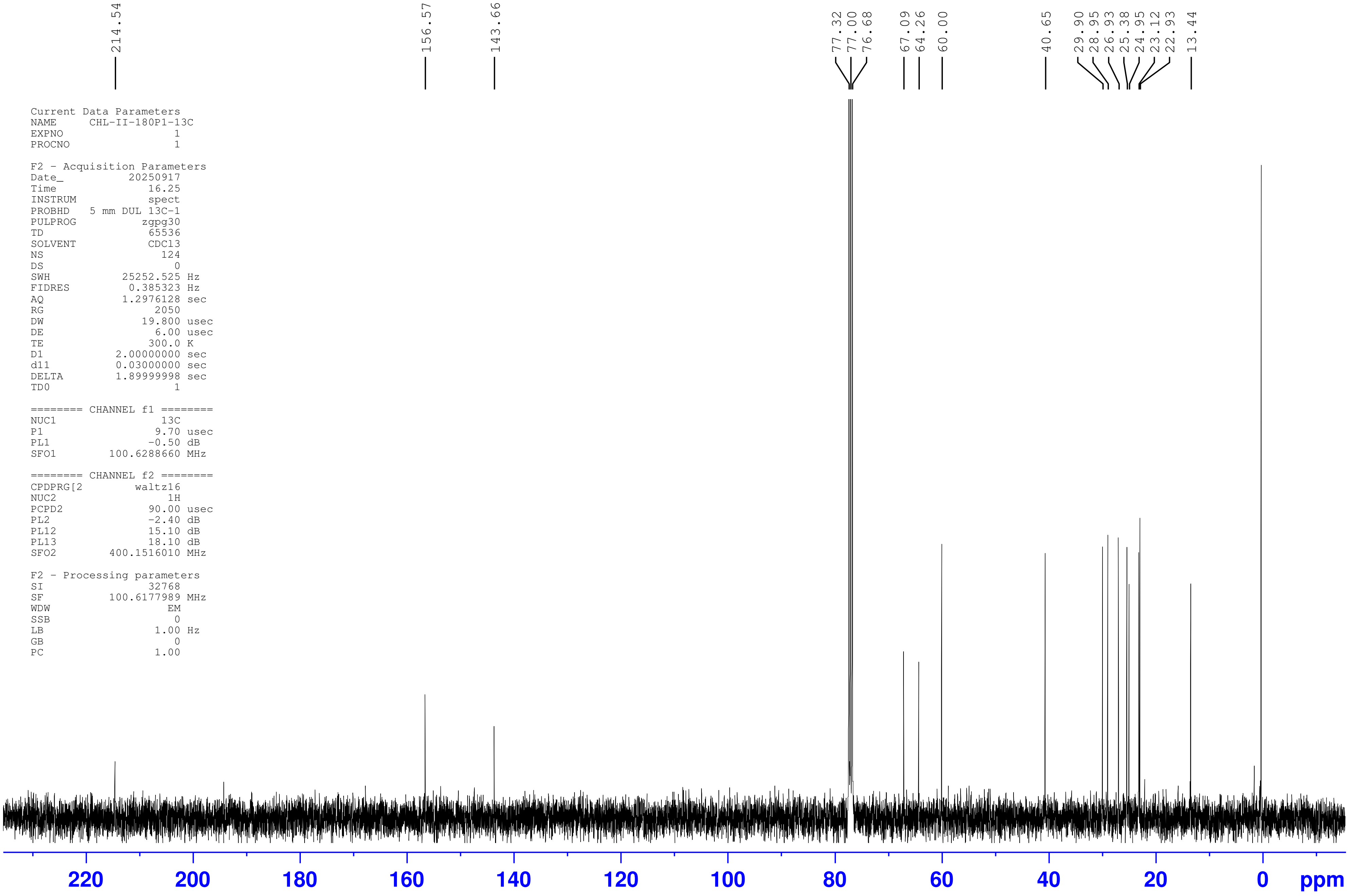

214.54
156.57
143.66
77.32
77.00
76.68
67.09
64.26
60.00
40.65
29.90
28.95
26.93
25.38
24.95
23.12
22.93
13.44
Current Data Parameters
NAME CHL-II-180P1-13C
EXPNO 1
PROCNO 1
F2 - Acquisition Parameters
Date_ 20250917
Time 16.25
INSTRUM spect
PROBHD 5 mm DUL 13C-1
PULPROG zgpg30
TD 65536
SOLVENT CDCl3
NS 124
DS 0
SWH 25252.525 Hz
FIDRES 0.385323 Hz
AQ 1.2976128 sec
RG 2050
DW 19.800 usec
DE 6.00 usec
TE 300.0 K
D1 2.00000000 sec
d11 0.03000000 sec
DELTA 1.89999998 sec
TD0 1
======== CHANNEL f1 ========
NUC1 13C
P1 9.70 usec
PL1 -0.50 dB
SFO1 100.6288660 MHz
======== CHANNEL f2 ========
CPDPRG[2 waltz16
NUC2 1H
PCPD2 90.00 usec
PL2 -2.40 dB
PL12 15.10 dB
PL13 18.10 dB
SFO2 400.1516010 MHz
F2 - Processing parameters
SI 32768
SF 100.6177989 MHz
WDW EM
SSB 0
LB 1.00 Hz
GB 0
PC 1.00
220
200
180
160
140
120
100
80
60
40
20
0
ppm
