# Supplementary material for: Diastereodivergent Access to cis,cis- and cis,trans-Angular Triquinanes: Total Synthesis of (±)-Arnicenone
Source: J Am Chem Soc. 2026 Sep 22;148(39):41724–32. doi: 10.1021/jacs.6c09019 (PMC13651531; doi:10.1021/jacs.6c09019)
Supplement: Supplementary file 5 [file ja6c09019_si_003.zip › FID 29-44/36/HTH-cis555 enone-MS.pdf]

Display Report

Analysis Info

Analysis Name	D:\Data\nctu service\data\2023\20230207\3HTH-cis555 enone_GB7_01_34322.d	Acquisition Date	2/7/2023 3:50:06 PM
Method	Small molecule.m	Operator	NCTU
Sample Name	3HTH-cis555 enone	Instrument	impact HD 1819696.00164
Comment			

Acquisition Parameter

Source Type	ESI	Ion Polarity	Positive	Set Nebulizer	1.0 Bar
Focus	Active	Set Capillary	4500 V	Set Dry Heater	200 °C
Scan Begin	50 m/z	Set End Plate Offset	-500 V	Set Dry Gas	6.0 l/min
Scan End	1500 m/z	Set Charging Voltage	2000 V	Set Divert Valve	Waste
		Set Corona	0 nA	Set APCI Heater	0 °C

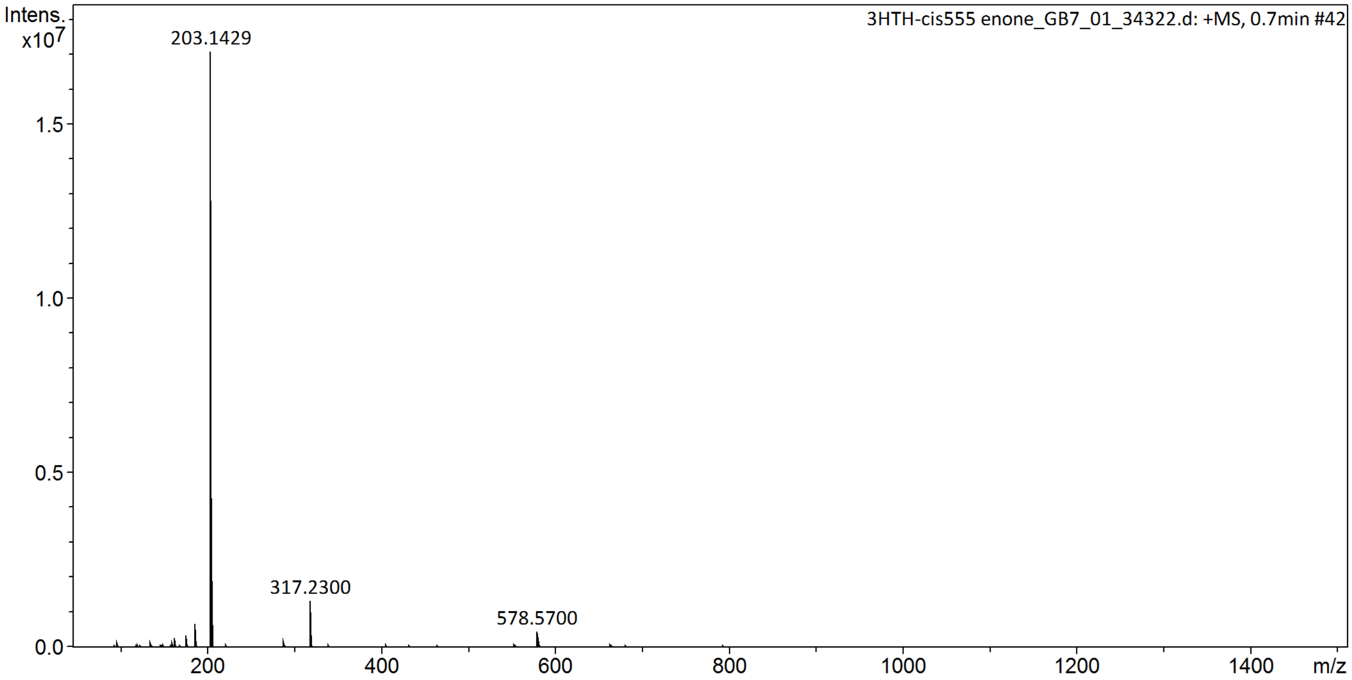

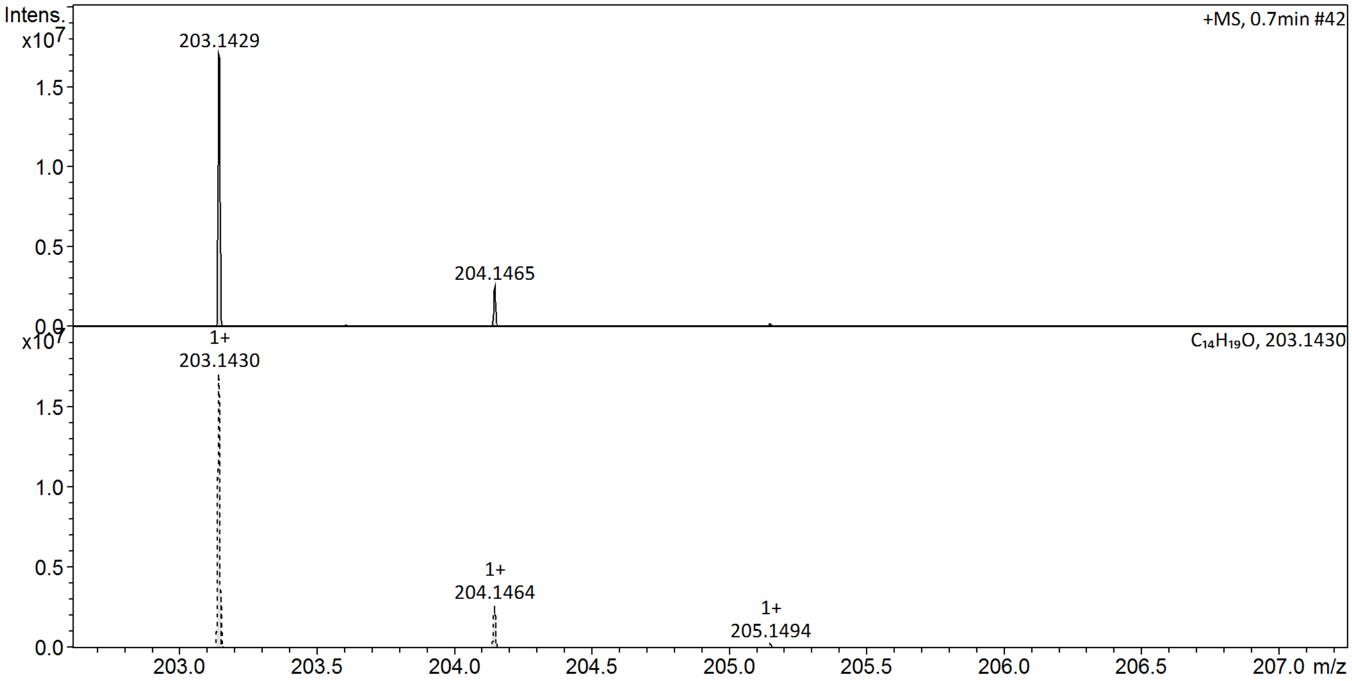

Display Report

Meas. m/z	#	Ion Formula	m/z	err [ppm]	mSigma	# Sigma	Score	rdb	e¯ Conf	N-Rule	Adduct
203.1429	1	C14H19O	203.1430	-0.6	3.9	1	100.00	5.5	even	ok	M+H

+MS, 0.7min #42

#	m/z	Res.	S/N	I	I %	FWHM
1	93.0698	16777	97.9	45610	0.3	0.0055
2	95.0857	16091	250.9	116926	0.7	0.0059
3	105.0701	15663	41.2	19226	0.1	0.0067
4	107.0856	15424	41.6	19450	0.1	0.0069
5	109.1011	17620	50.1	23394	0.1	0.0062
6	116.1068	16046	51.0	23840	0.1	0.0072
7	118.0861	17539	86.2	40280	0.2	0.0067
8	119.0854	17656	173.2	80826	0.5	0.0067
9	122.0963	17647	128.9	60120	0.4	0.0069
10	127.0725	18131	54.4	25336	0.1	0.0070
11	133.1022	16456	227.7	105718	0.6	0.0081
12	135.0802	17836	70.1	32540	0.2	0.0076
13	136.1118	18653	37.2	17248	0.1	0.0073
14	143.0853	19108	50.6	23346	0.1	0.0075
15	145.1006	19318	68.4	31518	0.2	0.0075
16	147.0804	18042	51.1	23522	0.1	0.0082
17	147.1163	19857	71.2	32764	0.2	0.0074
18	149.0230	18510	152.8	70238	0.4	0.0081
19	157.1007	18292	103.5	47530	0.3	0.0086
20	159.1165	18900	251.5	115836	0.7	0.0084
21	161.0957	19496	171.5	78892	0.5	0.0083
22	161.1321	19948	574.4	264274	1.5	0.0081
23	162.1352	19043	76.1	35010	0.2	0.0085
24	163.1112	17076	46.3	21218	0.1	0.0096
25	167.0333	21281	62.0	28318	0.2	0.0078
26	168.5769	22220	58.7	26846	0.2	0.0076
27	175.1112	18455	85.9	39272	0.2	0.0095
28	175.1478	20165	741.9	339182	2.0	0.0087
29	176.1509	18807	103.7	47400	0.3	0.0094
30	185.1322	22626	1459.6	662066	3.9	0.0082
31	186.1355	19554	211.8	95862	0.6	0.0095
32	201.1267	19265	46.6	20370	0.1	0.0104
33	203.1167	9352	90.4	39346	0.2	0.0217
34	203.1429	25437	39217.0	17071560	100.0	0.0080
35	203.1735	10861	62.2	27060	0.2	0.0187
36	203.6077	17512	182.3	79278	0.5	0.0116
37	204.1465	27843	5823.4	2530030	14.8	0.0073
38	205.1494	18984	373.6	161938	0.9	0.0108
39	221.1532	20434	168.6	69830	0.4	0.0108
40	279.1586	20365	72.0	25394	0.1	0.0137
41	282.2790	19198	52.8	18436	0.1	0.0147
42	287.1825	20784	416.6	141848	0.8	0.0138
43	288.1846	20078	105.0	35644	0.2	0.0144
44	307.2080	19044	54.8	17240	0.1	0.0161
45	317.2300	28371	4361.3	1346112	7.9	0.0112
46	318.2323	22710	1049.8	323538	1.9	0.0140
47	319.2315	14170	227.6	70086	0.4	0.0225
48	333.2239	19412	56.4	17150	0.1	0.0172
49	338.3415	22154	220.1	66770	0.4	0.0153
50	405.2787	21093	220.2	68178	0.4	0.0192
51	406.2823	22561	74.1	22984	0.1	0.0180
52	413.2659	21691	58.9	18358	0.1	0.0191
53	419.3153	18941	60.3	18860	0.1	0.0221
54	431.1788	22363	143.3	44882	0.3	0.0193
55	464.3885	20141	87.4	27790	0.2	0.0231
56	466.4042	20433	69.4	22066	0.1	0.0228
57	506.5293	20523	53.1	17298	0.1	0.0247
58	519.3663	20420	56.4	18534	0.1	0.0254
59	552.5530	21960	236.7	79676	0.5	0.0252

#	m/z	Res.	S/N	I	I %	FWHM
1	203.1430	25437		17071560	100.0	0.0080
2	204.1464	25562		2622285	15.4	0.0080
3	205.1494	25688		222461	1.3	0.0080

Display Report

#	m/z	Res.	S/N	I	I %	FWHM
60	553.5562	21428	100.9	33970	0.2	0.0258
61	578.5700	24131	1357.0	461692	2.7	0.0240
62	579.5729	23381	628.9	214156	1.3	0.0248
63	580.5808	17958	350.7	119442	0.7	0.0323
64	581.5845	18300	128.8	43890	0.3	0.0318
65	663.4539	23224	291.2	99280	0.6	0.0286
66	664.4580	20429	123.9	42284	0.2	0.0325
67	680.4809	21903	99.1	33592	0.2	0.0311
68	791.3756	21199	76.6	22986	0.1	0.0373
69	792.3755	20044	103.2	30954	0.2	0.0395
70	794.3771	20112	80.6	24112	0.1	0.0395
