# Supplementary material for: Diastereodivergent Access to cis,cis- and cis,trans-Angular Triquinanes: Total Synthesis of (±)-Arnicenone
Source: J Am Chem Soc. 2026 Sep 22;148(39):41724–32. doi: 10.1021/jacs.6c09019 (PMC13651531; doi:10.1021/jacs.6c09019)
Supplement: Supplementary file 5 [file ja6c09019_si_003.zip › FID 29-44/29b/HTH-desilyl alkyne enone (54)/HTH-desilyl alkyne enone-MS.pdf]

Display Report

Analysis Info

Analysis Name	D:\Data\nctu service\data\2022\20221216\13HTH-desilyl alkyne_GB6_01_33721.d	Acquisition Date	12/19/2022 3:04:55 PM
Method	Small molecule.m	Operator	NCTU
Sample Name	13HTH-desilyl alkyne	Instrument	impact HD 1819696.00164
Comment			

Acquisition Parameter

Source Type	ESI	Ion Polarity	Positive	Set Nebulizer	1.0 Bar
Focus	Active	Set Capillary	4500 V	Set Dry Heater	200 °C
Scan Begin	50 m/z	Set End Plate Offset	-500 V	Set Dry Gas	6.0 l/min
Scan End	1500 m/z	Set Charging Voltage	2000 V	Set Divert Valve	Waste
		Set Corona	0 nA	Set APCI Heater	0 °C

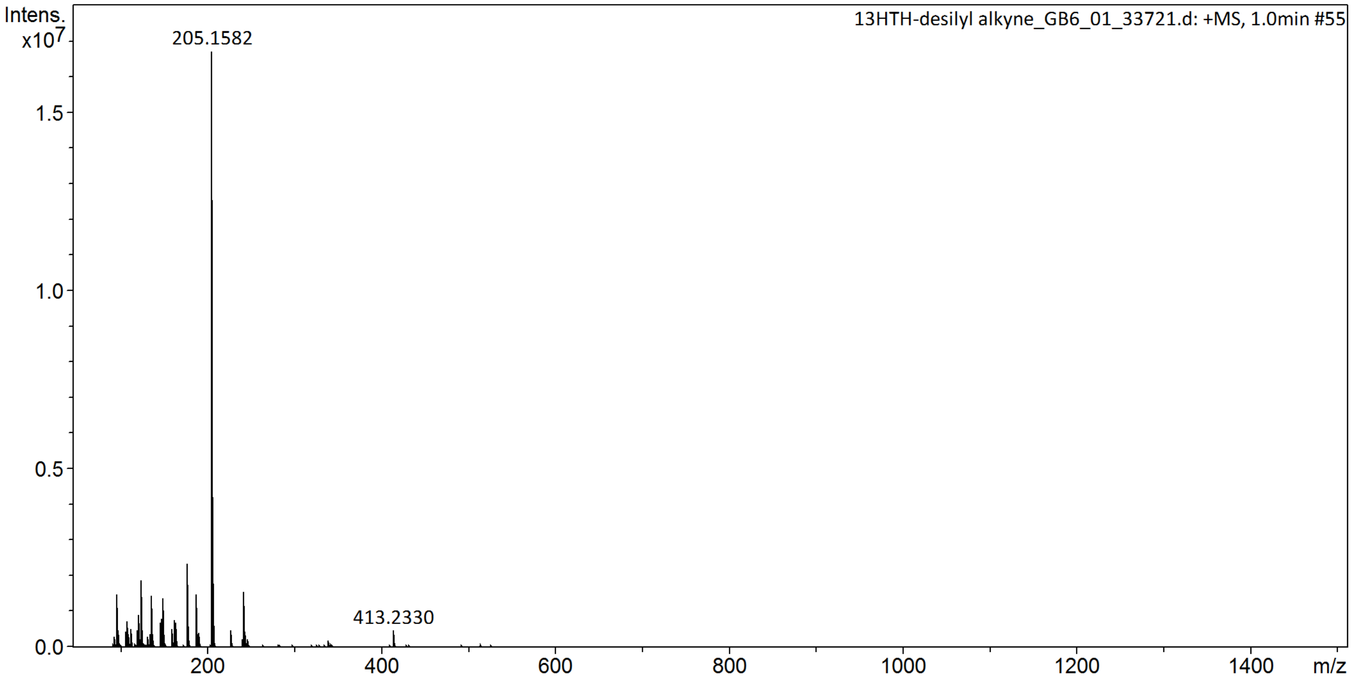

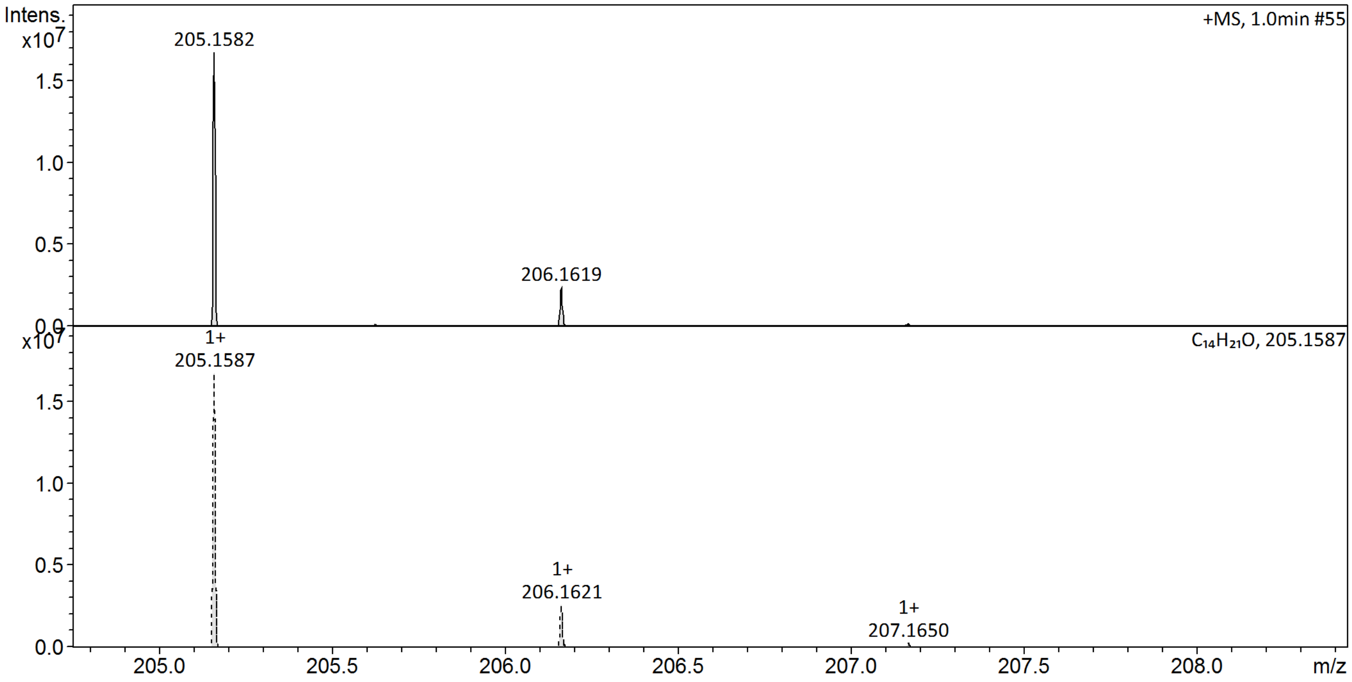

Display Report

Meas. m/z	#	Ion Formula	m/z	err [ppm]	mSigma	# Sigma	Score	rdb	e⁻ Conf	N-Rule	Adduct
205.1582	1	C14H21O	205.1587	2.3	6.8	1	100.00	4.5	even	ok	M+H

+MS, 1.0min #55

#	m/z	Res.	S/N	I	I %	FWHM
1	81.0705	14073	38.9	26766	0.2	0.0058
2	83.0494	15222	36.0	24756	0.1	0.0055
3	91.0542	16701	137.9	93812	0.6	0.0055
4	93.0700	16970	436.9	296448	1.8	0.0055
5	94.0733	15730	35.8	24260	0.1	0.0060
6	95.0491	16153	46.2	31306	0.2	0.0059
7	95.0857	19492	2193.6	1487530	8.9	0.0049
8	96.0889	16699	167.4	113490	0.7	0.0058
9	97.0649	17820	710.1	481394	2.9	0.0054
10	97.1008	16218	77.2	52310	0.3	0.0060
11	98.0679	16240	49.3	33412	0.2	0.0060
12	99.0804	16332	45.2	30708	0.2	0.0061
13	105.0698	17797	629.8	425016	2.5	0.0059
14	106.0732	15770	55.3	37306	0.2	0.0067
15	107.0489	17181	105.9	71404	0.4	0.0062
16	107.0855	18470	1077.5	726754	4.3	0.0058
17	108.0886	17115	97.6	65780	0.4	0.0063
18	109.0646	17644	560.9	377798	2.3	0.0062
19	109.1009	17993	550.4	370734	2.2	0.0061
20	110.0681	15608	45.8	30852	0.2	0.0071
21	110.1043	16331	49.8	33556	0.2	0.0067
22	111.0803	18752	778.5	523780	3.1	0.0059
23	112.0837	16713	57.5	38578	0.2	0.0067
24	116.1066	16715	123.7	82742	0.5	0.0069
25	117.0694	17920	66.4	44378	0.3	0.0065
26	118.0858	16811	32.3	21618	0.1	0.0070
27	119.0853	19064	686.4	458572	2.7	0.0062
28	120.0886	18817	67.8	45250	0.3	0.0064
29	121.0645	19253	473.7	316046	1.9	0.0063
30	121.1010	20831	1368.9	913194	5.5	0.0058
31	122.0679	15501	41.6	27702	0.2	0.0079
32	122.1034	16812	138.7	92398	0.6	0.0073
33	123.0803	22392	2843.5	1892628	11.3	0.0055
34	123.1163	18529	283.7	188854	1.1	0.0066
35	124.0837	17699	240.5	159760	1.0	0.0070
36	124.1198	17582	33.3	22134	0.1	0.0071
37	125.0955	17948	160.0	106130	0.6	0.0070
38	127.0722	18124	105.0	69436	0.4	0.0070
39	129.0461	17195	79.7	52576	0.3	0.0075
40	131.0431	15285	27.5	18106	0.1	0.0086
41	131.0852	19164	458.7	302496	1.8	0.0068
42	132.0888	16627	54.9	36096	0.2	0.0079
43	133.1008	19209	542.1	356244	2.1	0.0069
44	134.1043	17361	62.1	40766	0.2	0.0077
45	135.0802	22495	2192.5	1435226	8.6	0.0060
46	135.1164	19963	537.6	351890	2.1	0.0068
47	136.0838	16764	203.7	133086	0.8	0.0081
48	136.1191	17315	64.5	42158	0.3	0.0079
49	137.0956	18779	391.3	255308	1.5	0.0073
50	138.0988	17913	43.7	28486	0.2	0.0077
51	143.0852	18226	29.6	19224	0.1	0.0079
52	145.1009	20359	1069.9	691450	4.1	0.0071
53	146.1045	17226	137.6	88788	0.5	0.0085
54	147.0866	11788	41.7	26828	0.2	0.0125
55	147.1165	21762	1228.7	790420	4.7	0.0068
56	148.1197	19103	155.3	99670	0.6	0.0078
57	149.0224	17546	28.7	18352	0.1	0.0085
58	149.0959	22732	2123.0	1357378	8.1	0.0066
59	149.1319	18799	382.2	244310	1.5	0.0079

#	m/z	Res.	S/N	I	I %	FWHM
1	205.1587	28137		16712608	100.0	0.0073
2	206.1621	28274		2570992	15.4	0.0073
3	207.1650	28412		218365	1.3	0.0073

Display Report

#	m/z	Res.	S/N	I	I %	FWHM
60	150.0995	17534	232.4	148350	0.9	0.0086
61	150.1343	16037	49.5	31592	0.2	0.0094
62	151.1107	17560	100.4	63854	0.4	0.0086
63	158.1086	17922	39.3	24810	0.1	0.0088
64	159.1164	20820	794.6	503052	3.0	0.0076
65	160.1197	18201	97.4	61706	0.4	0.0088
66	161.0962	16105	51.3	32542	0.2	0.0100
67	161.1322	22548	1220.3	774896	4.6	0.0071
68	162.1033	18334	209.4	132870	0.8	0.0088
69	162.1359	16889	166.5	105670	0.6	0.0096
70	163.1113	21001	1043.4	660640	4.0	0.0078
71	163.1478	21656	1097.0	694494	4.2	0.0075
72	164.1155	16489	145.4	91994	0.6	0.0100
73	164.1507	17886	135.6	85792	0.5	0.0092
74	172.1241	18231	65.3	40884	0.2	0.0094
75	175.1118	17786	35.2	21906	0.1	0.0098
76	175.1476	18332	35.6	22146	0.1	0.0096
77	176.1185	20083	107.2	66604	0.4	0.0088
78	177.1271	26325	3762.0	2335386	14.0	0.0067
79	177.1635	22159	1128.8	700768	4.2	0.0080
80	178.1303	19586	428.2	265850	1.6	0.0091
81	178.1663	18442	161.6	100368	0.6	0.0097
82	179.1340	17120	35.5	22014	0.1	0.0105
83	185.1319	18888	35.7	21934	0.1	0.0098
84	187.1479	24963	2416.5	1481072	8.9	0.0075
85	188.1511	20103	345.9	211538	1.3	0.0094
86	189.1543	18566	28.0	17074	0.1	0.0102
87	190.1348	21535	653.8	397072	2.4	0.0088
88	191.1384	18354	92.9	56248	0.3	0.0104
89	203.1423	20508	68.8	40084	0.2	0.0099
90	205.1322	11426	62.8	36406	0.2	0.0180
91	205.1582	28137	28851.2	16712608	100.0	0.0073
92	205.1887	11708	64.5	37384	0.2	0.0175
93	205.6244	25086	184.9	106998	0.6	0.0082
94	206.1619	27423	4132.5	2389362	14.3	0.0075
95	207.1647	20369	281.2	162152	1.0	0.0102
96	227.1403	22469	884.9	477282	2.9	0.0101
97	228.1433	19809	137.9	74072	0.4	0.0115
98	239.1194	21668	459.5	237606	1.4	0.0110
99	240.1227	18697	67.5	34772	0.2	0.0128
100	241.1352	26894	3012.7	1546660	9.3	0.0090
101	242.1382	21003	432.1	220932	1.3	0.0115
102	243.1324	21613	889.7	453066	2.7	0.0112
103	244.1355	19282	133.5	67626	0.4	0.0127
104	245.1507	20912	473.1	238644	1.4	0.0117
105	246.1539	20087	79.2	39738	0.2	0.0123
106	247.1511	20095	122.6	61174	0.4	0.0123
107	263.1164	19249	72.1	34164	0.2	0.0137
108	281.1894	19737	70.3	30166	0.2	0.0142
109	282.2783	20498	53.1	22652	0.1	0.0138
110	283.2050	20254	119.9	51006	0.3	0.0140
111	297.2206	21258	70.7	27654	0.2	0.0140
112	301.1411	19631	56.3	21488	0.1	0.0153
113	319.2446	20990	87.8	31220	0.2	0.0152
114	325.1989	17199	142.0	49758	0.3	0.0189
115	328.2181	22403	83.0	28878	0.2	0.0147
116	334.1934	20400	81.2	27758	0.2	0.0164
117	338.3414	22243	123.9	41774	0.2	0.0152
118	339.1379	20389	111.9	37664	0.2	0.0166
119	339.2136	21774	585.7	197054	1.2	0.0156
120	340.2161	20441	165.6	55516	0.3	0.0166
121	342.2151	21638	226.0	75280	0.5	0.0158
122	342.7171	22461	108.5	36116	0.2	0.0153
123	361.1977	19052	66.9	21464	0.1	0.0190
124	373.1740	19581	56.7	17910	0.1	0.0191
125	407.2944	21490	70.2	21878	0.1	0.0190

Display Report

#	m/z	Res.	S/N	I	I %	FWHM
126	409.3100	19663	129.4	40310	0.2	0.0208
127	413.2330	24524	1476.5	458442	2.7	0.0168
128	414.2347	20898	507.3	157338	0.9	0.0198
129	415.2330	17908	172.5	53482	0.3	0.0232
130	428.2826	20762	182.9	56432	0.3	0.0206
131	428.7846	21706	104.9	32340	0.2	0.0198
132	431.2924	18967	100.1	30798	0.2	0.0227
133	437.2703	21318	85.0	26066	0.2	0.0205
134	491.2788	20032	113.3	34694	0.2	0.0245
135	513.2616	21666	303.5	93078	0.6	0.0237
136	514.2639	22265	131.9	40432	0.2	0.0231
137	525.2794	19479	99.5	30518	0.2	0.0270
138	525.3531	22313	66.8	20500	0.1	0.0235
