# Supplementary material for: Diastereodivergent Access to cis,cis- and cis,trans-Angular Triquinanes: Total Synthesis of (±)-Arnicenone
Source: J Am Chem Soc. 2026 Sep 22;148(39):41724–32. doi: 10.1021/jacs.6c09019 (PMC13651531; doi:10.1021/jacs.6c09019)
Supplement: Supplementary file 5 [file ja6c09019_si_003.zip › FID 29-44/29d/HTH-desilyl alkyne (aldehyde)/HTH-desilyl alkyne (aldehyde)-MS.pdf]

Display Report

Analysis Info

Analysis Name	D:\Data\nctu service\data\2023\20230620\HTH-24_GC8_01_36655.d	Acquisition Date	6/19/2023 11:23:24 PM
Method	Small molecule.m	Operator	NCTU
Sample Name	HTH-24	Instrument	impact HD 1819696.00164
Comment			

Acquisition Parameter

Source Type	ESI	Ion Polarity	Positive	Set Nebulizer	1.0 Bar
Focus	Active	Set Capillary	4500 V	Set Dry Heater	200 °C
Scan Begin	50 m/z	Set End Plate Offset	-500 V	Set Dry Gas	6.0 l/min
Scan End	1500 m/z	Set Charging Voltage	2000 V	Set Divert Valve	Waste
		Set Corona	0 nA	Set APCI Heater	0 °C

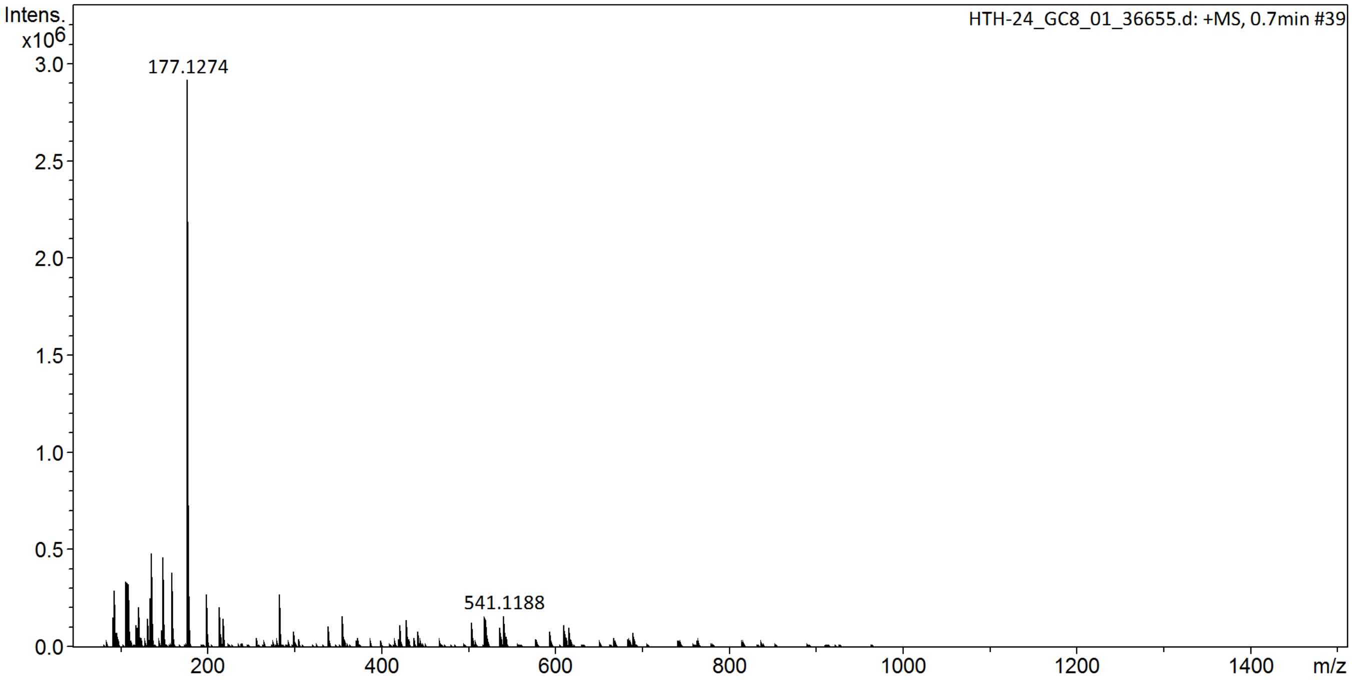

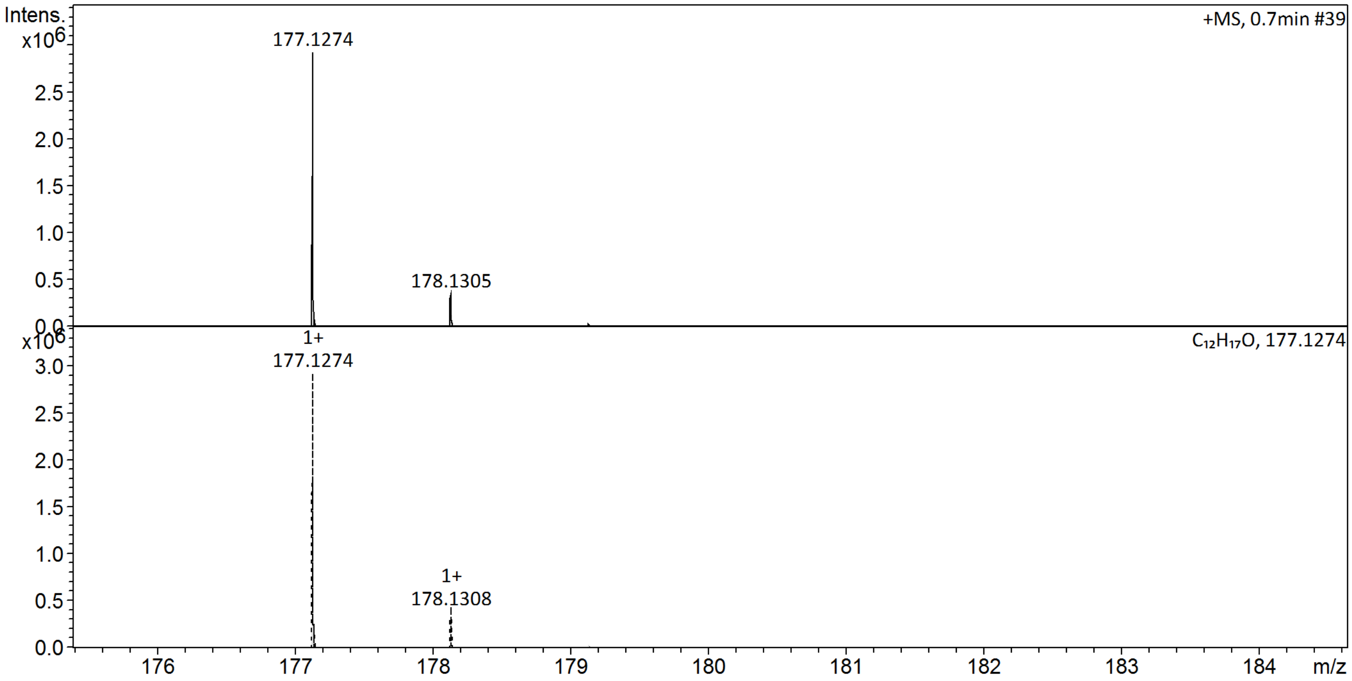

Display Report

Meas. m/z	#	Ion Formula	m/z	err [ppm]	mSigma	# Sigma	Score	rdb	e⁻ Conf	N-Rule	Adduct
177.1274	1	C12H17O	177.1274	-0.1	7.3	1	100.00	4.5	even	ok	M+H

+MS, 0.7min #39

#	m/z	Res.	S/N	I	I %	FWHM
1	81.0702	14970	36.2	11138	0.4	0.0054
2	83.0492	14761	59.9	18442	0.6	0.0056
3	91.0540	16070	501.2	153324	5.3	0.0057
4	92.0575	16469	47.6	14538	0.5	0.0056
5	93.0697	16811	946.0	289306	9.9	0.0055
6	94.0730	15702	80.5	24614	0.8	0.0060
7	95.0489	16791	134.9	41200	1.4	0.0057
8	95.0853	17043	243.6	74434	2.6	0.0056
9	96.0889	15030	19.0	5798	0.2	0.0064
10	97.0646	17678	141.2	43048	1.5	0.0055
11	103.0541	16463	17.0	5164	0.2	0.0063
12	105.0697	17642	1121.2	339396	11.6	0.0060
13	106.0733	17080	93.3	28242	1.0	0.0062
14	107.0489	18143	535.5	161924	5.6	0.0059
15	107.0853	18625	1103.4	333656	11.4	0.0057
16	108.0523	15828	39.6	11966	0.4	0.0068
17	108.0888	16102	95.5	28840	1.0	0.0067
18	109.0645	18219	1070.6	322870	11.1	0.0060
19	109.1008	17079	48.2	14538	0.5	0.0064
20	110.0677	17325	84.8	25532	0.9	0.0064
21	111.0800	18853	107.9	32460	1.1	0.0059
22	115.0540	17986	27.8	8330	0.3	0.0064
23	117.0696	17724	381.7	113966	3.9	0.0066
24	118.0858	17374	144.1	42972	1.5	0.0068
25	119.0853	18331	338.6	100884	3.5	0.0065
26	120.0888	17678	40.8	12152	0.4	0.0068
27	121.0644	18476	687.1	204296	7.0	0.0066
28	121.1009	17837	407.1	121052	4.2	0.0068
29	121.9656	18412	22.5	6688	0.2	0.0066
30	122.0693	12635	71.7	21296	0.7	0.0097
31	122.1031	15582	44.3	13148	0.5	0.0078
32	123.0802	17759	171.3	50794	1.7	0.0069
33	124.0853	15445	18.5	5484	0.2	0.0080
34	127.0737	14952	19.7	5794	0.2	0.0085
35	127.1478	17797	97.9	28840	1.0	0.0071
36	129.0692	18725	33.0	9720	0.3	0.0069
37	130.0766	17083	23.1	6800	0.2	0.0076
38	131.0851	19493	508.5	149474	5.1	0.0067
39	132.0883	18429	52.8	15506	0.5	0.0072
40	133.0647	16327	19.2	5626	0.2	0.0081
41	133.1009	18949	857.4	251676	8.6	0.0070
42	134.1045	17564	102.9	30176	1.0	0.0076
43	135.0802	19117	810.6	237856	8.2	0.0071
44	135.1167	20702	1644.3	482438	16.5	0.0065
45	136.0851	13650	89.0	26084	0.9	0.0100
46	136.1199	17115	159.2	46668	1.6	0.0080
47	137.0941	12876	22.7	6644	0.2	0.0106
48	140.0681	18927	29.2	8504	0.3	0.0074
49	144.0930	18598	103.8	30092	1.0	0.0077
50	145.0990	13631	21.2	6154	0.2	0.0106
51	147.0805	16723	27.3	7898	0.3	0.0088
52	147.1164	18472	291.1	84112	2.9	0.0080
53	148.0879	17506	67.7	19534	0.7	0.0085
54	148.1200	17017	39.3	11346	0.4	0.0087
55	149.0229	18000	67.6	19468	0.7	0.0083
56	149.0959	20406	1201.1	346082	11.9	0.0073
57	149.1323	21168	1597.5	460268	15.8	0.0070
58	150.0991	18101	119.4	34346	1.2	0.0083
59	150.1355	19318	198.0	56940	2.0	0.0078

#	m/z	Res.	S/N	I	I %	FWHM
1	177.1274	26550		2915516	100.0	0.0067
2	178.1308	26701		384102	13.2	0.0067
3	179.1336	26851		29241	1.0	0.0067

Display Report

#	m/z	Res.	S/N	I	I %	FWHM
60	153.0910	18193	27.9	7992	0.3	0.0084
61	155.5691	20479	24.0	6848	0.2	0.0076
62	157.1008	21189	44.7	12740	0.4	0.0074
63	159.1167	20370	1343.9	382098	13.1	0.0078
64	160.1198	19836	184.9	52508	1.8	0.0081
65	162.1038	18077	63.7	18018	0.6	0.0090
66	167.0331	19820	26.0	7296	0.3	0.0084
67	167.0549	18581	35.6	10012	0.3	0.0090
68	175.1112	19381	64.7	17962	0.6	0.0090
69	177.1274	26550	10544.5	2915516	100.0	0.0067
70	178.1305	21309	1260.9	348552	12.0	0.0084
71	179.1335	18291	88.9	24534	0.8	0.0098
72	192.1382	18418	21.2	5782	0.2	0.0104
73	195.1370	18559	23.6	6408	0.2	0.0105
74	199.1090	21085	1003.4	271766	9.3	0.0094
75	200.1123	20497	128.1	34636	1.2	0.0098
76	200.2002	21306	60.1	16252	0.6	0.0094
77	205.1577	20302	26.9	7240	0.2	0.0101
78	213.1036	20405	760.1	202998	7.0	0.0104
79	213.1456	17898	23.1	6182	0.2	0.0119
80	214.1069	21097	109.0	29128	1.0	0.0101
81	215.1007	19634	252.2	67310	2.3	0.0110
82	216.1034	21873	35.8	9554	0.3	0.0099
83	217.1192	21454	558.9	148840	5.1	0.0101
84	218.1229	19564	77.8	20688	0.7	0.0111
85	223.0630	20413	59.9	15818	0.5	0.0109
86	223.1156	22692	43.6	11506	0.4	0.0098
87	224.1269	20296	47.8	12614	0.4	0.0110
88	225.0429	21914	27.2	7178	0.2	0.0103
89	228.2312	20673	21.5	5670	0.2	0.0110
90	235.0857	19790	51.0	13362	0.5	0.0119
91	239.0936	16022	50.4	13142	0.5	0.0149
92	245.2256	26996	22.7	5912	0.2	0.0091
93	247.1515	17954	24.7	6448	0.2	0.0138
94	247.2410	21639	35.8	9322	0.3	0.0114
95	256.2627	21050	188.3	48818	1.7	0.0122
96	257.1144	19877	19.9	5154	0.2	0.0129
97	257.2481	21372	29.9	7740	0.3	0.0120
98	257.2652	18996	31.2	8078	0.3	0.0135
99	263.2361	18771	42.9	11128	0.4	0.0140
100	265.0805	23119	20.7	5352	0.2	0.0115
101	265.2521	20905	90.1	23330	0.8	0.0127
102	274.2730	19482	41.7	10778	0.4	0.0141
103	275.1632	19953	83.2	21516	0.7	0.0138
104	275.2573	20522	24.8	6426	0.2	0.0134
105	278.2471	19981	19.9	5148	0.2	0.0139
106	280.2628	18839	99.9	25918	0.9	0.0149
107	281.2656	19567	24.8	6418	0.2	0.0144
108	282.2786	22737	1042.0	270040	9.3	0.0124
109	283.2817	19377	196.7	50936	1.7	0.0146
110	284.2924	15378	57.4	14848	0.5	0.0185
111	285.1782	17912	21.1	5454	0.2	0.0159
112	285.2786	18439	26.9	6972	0.2	0.0155
113	286.3092	20531	36.5	9428	0.3	0.0139
114	290.2688	19825	22.7	5858	0.2	0.0146
115	292.1456	25409	28.6	7400	0.3	0.0115
116	293.1726	18644	92.6	23952	0.8	0.0157
117	297.0823	21121	43.2	11172	0.4	0.0141
118	299.0609	21911	308.2	79720	2.7	0.0136
119	299.1619	19845	28.7	7438	0.3	0.0151
120	300.0613	20015	79.7	20610	0.7	0.0150
121	301.0583	17451	44.7	11556	0.4	0.0173
122	301.1402	21270	60.7	15682	0.5	0.0142
123	302.1697	21715	26.5	6846	0.2	0.0139
124	302.2442	21328	43.9	11346	0.4	0.0142
125	304.2607	21850	165.2	42712	1.5	0.0139

Display Report

#	m/z	Res.	S/N	I	I %	FWHM
126	305.2636	19277	29.4	7604	0.3	0.0158
127	309.2025	22458	43.1	11166	0.4	0.0138
128	321.3151	24512	21.0	5432	0.2	0.0131
129	325.2477	20564	51.3	13260	0.5	0.0158
130	333.2197	23106	25.4	6568	0.2	0.0144
131	338.3408	21563	422.4	109532	3.8	0.0157
132	339.1724	15149	24.6	6382	0.2	0.0224
133	339.3432	23567	102.0	26458	0.9	0.0144
134	347.2293	21308	43.2	11214	0.4	0.0163
135	351.2317	20686	41.6	10794	0.4	0.0170
136	355.0688	21766	625.7	162588	5.6	0.0163
137	356.0694	20949	212.4	55188	1.9	0.0170
138	357.0669	19205	127.6	33166	1.1	0.0186
139	358.0678	18741	38.6	10040	0.3	0.0191
140	360.3230	22379	68.2	17744	0.6	0.0161
141	364.2299	26002	20.5	5322	0.2	0.0140
142	371.1001	20854	129.8	33840	1.2	0.0178
143	372.1015	20488	47.1	12278	0.4	0.0182
144	372.2186	22118	35.2	9184	0.3	0.0168
145	372.7208	30677	21.9	5726	0.2	0.0121
146	373.0798	21866	185.3	48378	1.7	0.0171
147	373.2137	21772	33.4	8726	0.3	0.0171
148	374.0805	21664	64.8	16922	0.6	0.0173
149	375.0788	18374	37.5	9802	0.3	0.0204
150	375.2272	14892	29.2	7632	0.3	0.0252
151	387.2127	19772	102.9	27032	0.9	0.0196
152	399.2494	20819	127.0	33606	1.2	0.0192
153	400.2537	20821	27.7	7330	0.3	0.0192
154	409.1937	19044	63.2	16802	0.6	0.0215
155	411.0931	23720	21.9	5818	0.2	0.0173
156	413.2645	17821	32.7	8722	0.3	0.0232
157	415.2102	23338	101.5	27086	0.9	0.0178
158	416.2135	20334	29.9	7986	0.3	0.0205
159	419.3137	22158	152.0	40606	1.4	0.0189
160	420.3170	19916	44.5	11894	0.4	0.0211
161	421.2310	22988	425.5	113664	3.9	0.0183
162	421.3243	21390	21.0	5596	0.2	0.0197
163	422.2345	20494	82.6	22070	0.8	0.0206
164	429.0872	23292	530.5	141848	4.9	0.0184
165	430.0879	20803	207.2	55412	1.9	0.0207
166	431.0851	21223	149.0	39898	1.4	0.0203
167	431.1774	20242	49.6	13278	0.5	0.0213
168	432.0853	18967	44.3	11874	0.4	0.0228
169	437.1923	22015	174.9	46992	1.6	0.0199
170	438.1960	20095	46.9	12582	0.4	0.0218
171	441.2960	22215	306.2	82322	2.8	0.0199
172	442.2991	22134	91.0	24498	0.8	0.0200
173	445.1182	21106	113.8	30688	1.1	0.0211
174	446.1195	19667	49.0	13208	0.5	0.0227
175	447.1056	6772	41.3	11158	0.4	0.0660
176	451.2643	23180	51.0	13778	0.5	0.0195
177	467.1003	21646	105.0	28554	1.0	0.0216
178	468.1012	22966	47.2	12840	0.4	0.0204
179	469.0986	21754	32.7	8902	0.3	0.0216
180	473.2463	18372	20.5	5596	0.2	0.0258
181	473.3422	26821	26.0	7074	0.2	0.0176
182	480.5125	24793	25.6	7004	0.2	0.0194
183	485.1114	21368	19.3	5310	0.2	0.0227
184	494.5634	22166	50.1	13848	0.5	0.0223
185	503.1056	22719	448.4	124248	4.3	0.0221
186	504.1061	21085	215.5	59754	2.0	0.0239
187	505.1037	21703	164.2	45514	1.6	0.0233
188	506.1032	22126	51.5	14272	0.5	0.0229
189	507.1022	21558	25.0	6920	0.2	0.0235
190	507.2884	21329	27.2	7540	0.3	0.0238
191	508.5439	20290	74.6	20706	0.7	0.0251

Display Report

#	m/z	Res.	S/N	I	I %	FWHM
192	509.5461	20483	29.2	8108	0.3	0.0249
193	517.3687	20243	20.6	5752	0.2	0.0256
194	519.1371	22680	559.6	155818	5.3	0.0229
195	520.1375	22142	278.1	77406	2.7	0.0235
196	521.1347	22025	210.6	58622	2.0	0.0237
197	522.1338	19023	73.6	20492	0.7	0.0274
198	522.5942	19251	32.1	8942	0.3	0.0271
199	523.1319	18484	34.4	9568	0.3	0.0283
200	536.1632	22752	361.4	100600	3.5	0.0236
201	536.5739	20738	77.1	21450	0.7	0.0259
202	537.1606	16982	198.3	55158	1.9	0.0316
203	537.5781	22153	31.9	8880	0.3	0.0243
204	538.1594	19141	146.2	40674	1.4	0.0281
205	539.1578	15224	56.2	15618	0.5	0.0354
206	540.1562	17147	24.8	6898	0.2	0.0315
207	541.1188	22118	575.0	159684	5.5	0.0245
208	542.1197	20621	270.4	75054	2.6	0.0263
209	543.1174	20667	201.9	55996	1.9	0.0263
210	544.1176	20840	77.4	21462	0.7	0.0261
211	545.1168	19991	30.5	8444	0.3	0.0273
212	557.0921	22310	43.6	11992	0.4	0.0250
213	558.0933	20729	21.2	5824	0.2	0.0269
214	561.3956	21964	26.4	7264	0.2	0.0256
215	577.1232	23426	140.5	38072	1.3	0.0246
216	578.1247	22541	87.4	23672	0.8	0.0256
217	579.1224	20545	59.2	16028	0.5	0.0282
218	580.1226	19051	20.6	5568	0.2	0.0305
219	593.1553	22102	289.7	77564	2.7	0.0268
220	594.1566	22963	175.2	46862	1.6	0.0259
221	595.1537	19642	124.2	33216	1.1	0.0303
222	596.1528	21055	52.9	14142	0.5	0.0283
223	597.1527	19711	24.1	6452	0.2	0.0303
224	605.4216	22426	23.9	6360	0.2	0.0270
225	610.1813	23316	432.4	114422	3.9	0.0262
226	611.1812	20691	250.1	66164	2.3	0.0295
227	612.1798	20066	182.3	48190	1.7	0.0305
228	613.1802	18321	75.0	19812	0.7	0.0335
229	614.1775	17838	31.2	8238	0.3	0.0344
230	615.1370	21778	374.5	98840	3.4	0.0282
231	616.1378	23520	234.1	61756	2.1	0.0262
232	617.1352	21481	167.8	44216	1.5	0.0287
233	618.1345	21281	74.7	19674	0.7	0.0290
234	619.1332	21521	26.8	7030	0.2	0.0288
235	622.0272	22060	28.2	7402	0.3	0.0282
236	631.1100	21878	40.0	10378	0.4	0.0288
237	632.1125	23244	24.3	6322	0.2	0.0272
238	633.1118	21442	23.4	6072	0.2	0.0295
239	651.1423	22314	82.3	20904	0.7	0.0292
240	652.1431	21743	50.9	12910	0.4	0.0300
241	653.1415	20847	40.4	10242	0.4	0.0313
242	663.4524	19131	41.1	10278	0.4	0.0347
243	667.1738	21090	202.6	50392	1.7	0.0316
244	668.1745	22050	135.8	33704	1.2	0.0303
245	669.1729	21008	107.4	26600	0.9	0.0319
246	670.1727	21405	52.7	13030	0.4	0.0313
247	684.1996	23423	179.9	43272	1.5	0.0292
248	685.1991	20784	128.9	30972	1.1	0.0330
249	685.4319	21690	92.5	22212	0.8	0.0316
250	686.1977	18161	88.8	21288	0.7	0.0378
251	686.4375	19679	37.8	9074	0.3	0.0349
252	687.1957	17784	42.2	10104	0.3	0.0386
253	689.1553	21872	299.2	71434	2.5	0.0315
254	690.1562	20876	184.2	43930	1.5	0.0331
255	691.1540	20170	141.7	33746	1.2	0.0343
256	692.1542	22071	73.4	17454	0.6	0.0314
257	693.1527	21538	31.9	7584	0.3	0.0322

#	m/z	Res.	S/N	I	I %	FWHM
258	705.1308	21939	72.4	16878	0.6	0.0321
259	706.1300	19398	40.5	9422	0.3	0.0364
260	707.1269	23660	46.8	10878	0.4	0.0299
261	741.1921	20946	165.9	36892	1.3	0.0354
262	742.1931	20892	122.5	27212	0.9	0.0355
263	743.1905	22136	109.6	24322	0.8	0.0336
264	744.1922	20063	51.1	11312	0.4	0.0371
265	745.1906	20654	26.6	5892	0.2	0.0361
266	758.2195	20316	65.5	14206	0.5	0.0373
267	759.2175	20475	63.3	13712	0.5	0.0371
268	760.2159	19511	47.0	10170	0.3	0.0390
269	761.2152	17521	28.5	6146	0.2	0.0434
270	763.1746	22038	168.7	36308	1.2	0.0346
271	764.1740	21233	120.2	25796	0.9	0.0360
272	765.1729	21712	102.0	21860	0.7	0.0352
273	766.1709	20785	48.9	10472	0.4	0.0369
274	779.1477	21951	81.9	17322	0.6	0.0355
275	780.1495	23828	67.0	14130	0.5	0.0327
276	781.1483	18966	50.6	10662	0.4	0.0412
277	782.1493	24264	30.6	6440	0.2	0.0322
278	815.2107	23353	117.2	23850	0.8	0.0349
279	816.2114	20314	91.3	18578	0.6	0.0402
280	817.2092	21550	77.7	15788	0.5	0.0379
281	818.2092	18744	39.3	7980	0.3	0.0437
282	832.2402	20208	29.1	5820	0.2	0.0412
283	837.1934	21793	96.9	19220	0.7	0.0384
284	838.1935	20310	73.3	14518	0.5	0.0413
285	839.1918	22363	70.2	13872	0.5	0.0375
286	840.1923	17365	32.8	6484	0.2	0.0484
287	853.1671	23508	61.3	11934	0.4	0.0363
288	854.1664	21951	47.3	9216	0.3	0.0389
289	855.1665	18886	43.7	8498	0.3	0.0453
290	889.2293	21532	62.7	11892	0.4	0.0413
291	890.2322	20073	50.3	9548	0.3	0.0444
292	891.2291	24001	52.5	9964	0.3	0.0371
293	892.2319	18322	27.7	5248	0.2	0.0487
294	911.2123	21286	56.2	10534	0.4	0.0428
295	912.2115	21977	39.7	7438	0.3	0.0415
296	913.2106	20032	39.1	7320	0.3	0.0456
297	922.0067	22394	27.6	5144	0.2	0.0412
298	927.1855	21523	32.0	5948	0.2	0.0431
299	963.2487	21682	32.1	5984	0.2	0.0444
300	965.2465	22109	29.2	5440	0.2	0.0437
