# Supplementary material for: Diastereodivergent Access to cis,cis- and cis,trans-Angular Triquinanes: Total Synthesis of (±)-Arnicenone
Source: J Am Chem Soc. 2026 Sep 22;148(39):41724–32. doi: 10.1021/jacs.6c09019 (PMC13651531; doi:10.1021/jacs.6c09019)
Supplement: Supplementary file 5 [file ja6c09019_si_003.zip › FID 29-44/29d/HTH-desilyl alkyne (aldehyde)/HTH-desilyl alkyne (aldehyde)-1H.pdf]

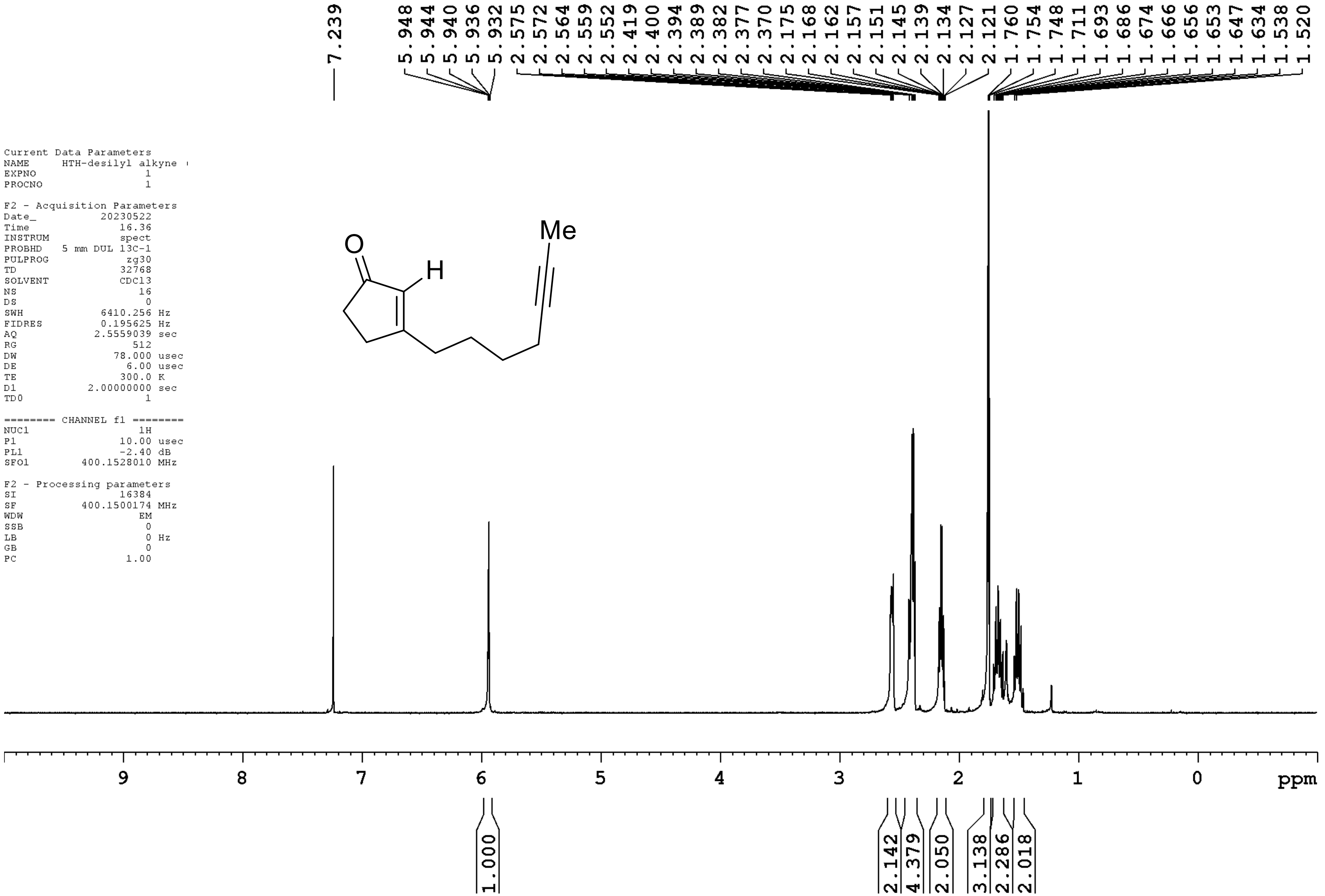

7.239
5.948
5.944
5.940
5.936
5.932
2.575
2.572
2.564
2.559
2.552
2.419
2.400
2.394
2.389
2.382
2.377
2.370
2.175
2.168
2.162
2.157
2.151
2.145
2.139
2.134
2.127
2.121
1.760
1.754
1.748
1.711
1.693
1.686
1.674
1.666
1.656
1.653
1.647
1.634
1.538
1.520
Current Data Parameters
NAME HTH-desilyl alkyne
EXPNO 1
PROCNO 1
F2 - Acquisition Parameters
Date_ 20230522
Time 16.36
INSTRUM spect
PROBHD 5 mm DUL 13C-1
PULPROG zg30
TD 32768
SOLVENT CDCl3
NS 16
DS 0
SWH 6410.256 Hz
FIDRES 0.195625 Hz
AQ 2.5559039 sec
RG 512
DW 78.000 usec
DE 6.00 usec
TE 300.0 K
D1 2.00000000 sec
TD0 1
======== CHANNEL f1 ========
NUC1 1H
P1 10.00 usec
PL1 -2.40 dB
SFO1 400.1528010 MHz
F2 - Processing parameters
SI 16384
SF 400.1500174 MHz
WDW EM
SSB 0
LB 0 Hz
GB 0
PC 1.00
O
H
Me
9
8
7
6
5
4
3
2
1
0
ppm
1.000
2.142
4.379
2.050
3.138
2.286
2.018
