# Supplementary material for: Diastereodivergent Access to cis,cis- and cis,trans-Angular Triquinanes: Total Synthesis of (±)-Arnicenone
Source: J Am Chem Soc. 2026 Sep 22;148(39):41724–32. doi: 10.1021/jacs.6c09019 (PMC13651531; doi:10.1021/jacs.6c09019)

```
Current Data Parameters
NAME      HTH-desilyl alkyne
EXPNO                  2
PROCNO                 1

F2 - Acquisition Parameters
Date_          20230522
Time              16.40
INSTRUM           spect
PROBHD   5 mm DUL 13C-1
PULPROG         zgpg30
TD               65536
SOLVENT          CDCl3
NS                 100
DS                   0
SWH          26041.666 Hz
FIDRES        0.397364 Hz
AQ           1.2582912 sec
RG                1820
DW              19.200 use
DE                6.00 use
TE               300.0 K
D1          2.00000000 sec
d11         0.03000000 sec
DELTA       1.89999998 sec
TD0                  1

======== CHANNEL f1 ========
NUC1               13C
P1                9.70 use
PL1              -0.50 dB
SFO1       100.6288660 MHz

======== CHANNEL f2 ========
CPDPRG[2       waltz16
NUC2                1H
PCPD2            90.00 use
PL2              -2.40 dB
PL12             15.10 dB
PL13             18.10 dB
SFO2       400.1516010 MHz

F2 - Processing parameters
SI               32768
SF         100.6178028 MHz
WDW                 EM
SSB                  0
LB                3.00 Hz
GB                   0
PC                1.00
```

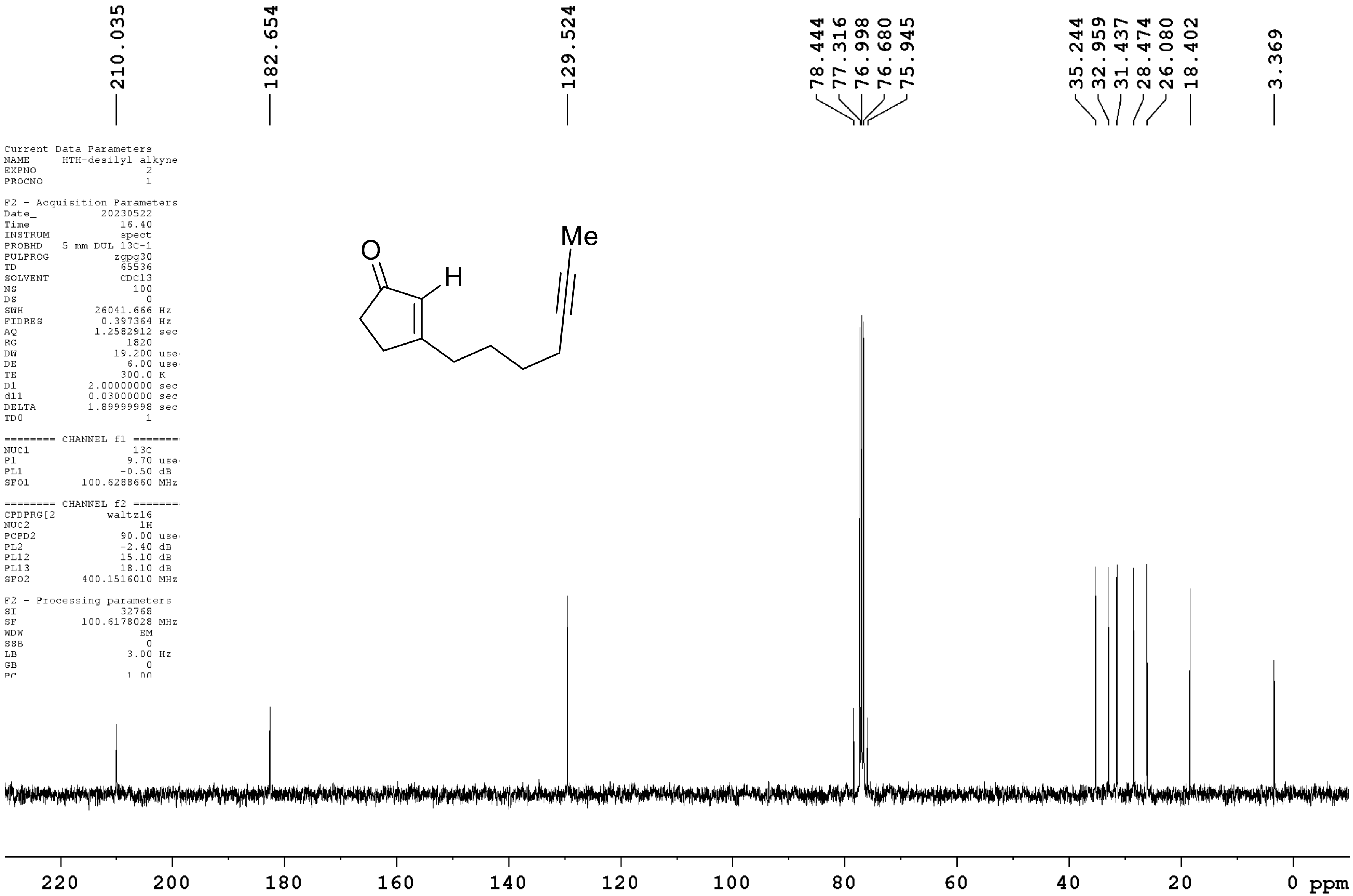

Supplement: Supplementary file 5 [file ja6c09019_si_003.zip › FID 29-44/29d/HTH-desilyl alkyne (aldehyde)/HTH-desilyl alkyne (aldehyde)-13C.pdf]
