# Supplementary material for: Diastereodivergent Access to cis,cis- and cis,trans-Angular Triquinanes: Total Synthesis of (±)-Arnicenone
Source: J Am Chem Soc. 2026 Sep 22;148(39):41724–32. doi: 10.1021/jacs.6c09019 (PMC13651531; doi:10.1021/jacs.6c09019)
Supplement: Supplementary file 5 [file ja6c09019_si_003.zip › FID 29-44/29a/HTH-alpha demthyl desilyl alkyne enone (93)/HTH-alpha demethyl desilyl alkyne enone-MS.pdf]

Display Report

Analysis Info

Analysis Name	D:\Data\nctu service\data\2022\20221216\14HTH-alpha dimethyl desilyl a_GB7_01_33722.d	Acquisition Date	12/19/2022 3:09:13 PM
Method	Small molecule.m	Operator	NCTU
Sample Name	14HTH-alpha dimethyl desilyl a	Instrument	impact HD 1819696.00164
Comment			

Acquisition Parameter

Source Type	ESI	Ion Polarity	Positive	Set Nebulizer	1.0 Bar
Focus	Active	Set Capillary	4500 V	Set Dry Heater	200 °C
Scan Begin	50 m/z	Set End Plate Offset	-500 V	Set Dry Gas	6.0 l/min
Scan End	1500 m/z	Set Charging Voltage	2000 V	Set Divert Valve	Waste
		Set Corona	0 nA	Set APCI Heater	0 °C

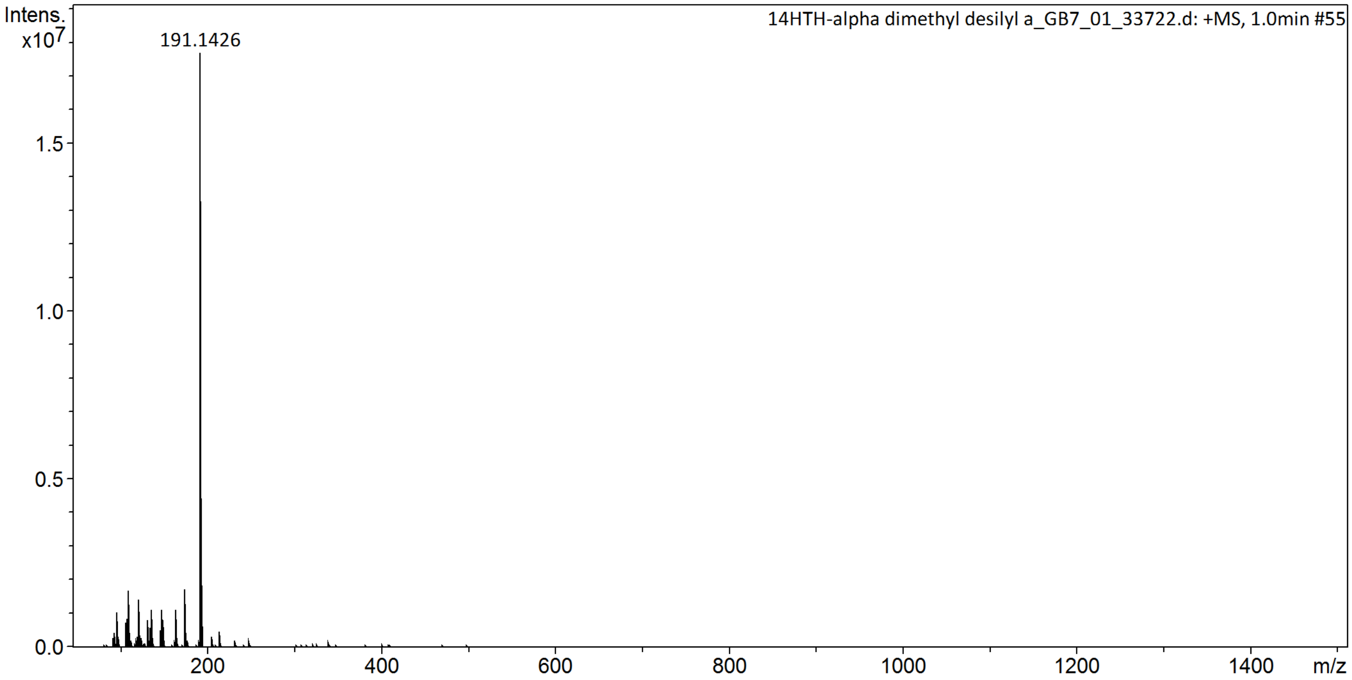

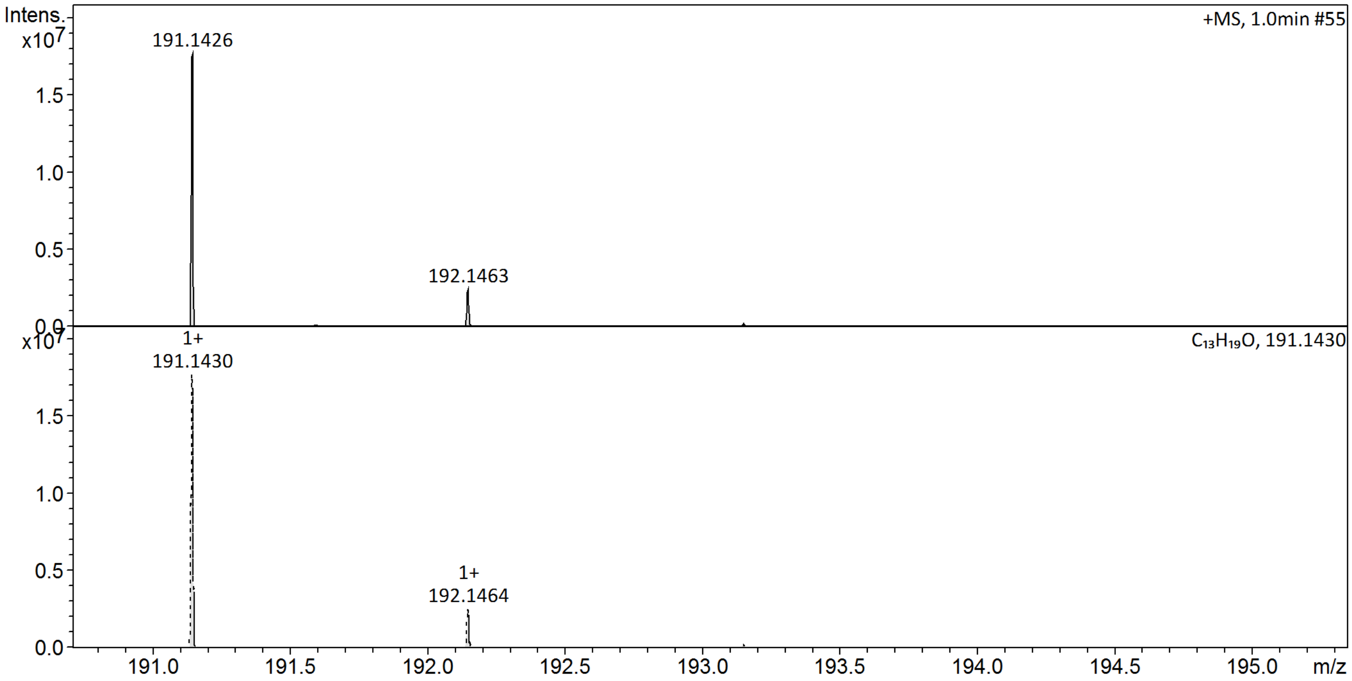

Display Report

Meas. m/z	#	Ion Formula	m/z	err [ppm]	mSigma	# Sigma	Score	rdb	e⁻ Conf	N-Rule	Adduct
191.1426	1	C13H19O	191.1430	2.3	3.0	1	100.00	4.5	even	ok	M+H

+MS, 1.0min #55

#	m/z	Res.	S/N	I	I %	FWHM
1	81.0702	14877	66.7	41836	0.2	0.0054
2	83.0494	14541	97.4	61052	0.3	0.0057
3	91.0542	16545	417.2	259000	1.5	0.0055
4	92.0576	14730	34.6	21434	0.1	0.0062
5	93.0699	17360	694.3	429990	2.4	0.0054
6	94.0731	16110	57.8	35766	0.2	0.0058
7	95.0490	17312	158.3	97950	0.6	0.0055
8	95.0856	18845	1659.1	1026246	5.8	0.0050
9	96.0887	16894	129.8	80290	0.5	0.0057
10	97.0647	17457	533.2	329498	1.9	0.0056
11	97.1009	15456	44.4	27462	0.2	0.0063
12	98.0680	16069	37.9	23410	0.1	0.0061
13	99.0802	16072	33.6	20718	0.1	0.0062
14	105.0698	18544	1209.8	738530	4.2	0.0057
15	106.0730	17298	104.5	63742	0.4	0.0061
16	107.0490	18072	389.6	237238	1.3	0.0059
17	107.0855	18555	1404.9	855438	4.8	0.0058
18	108.0525	14107	29.9	18196	0.1	0.0077
19	108.0886	17386	131.9	80250	0.5	0.0062
20	109.0647	20658	2766.8	1680804	9.5	0.0053
21	109.1007	18011	343.1	208412	1.2	0.0061
22	110.0678	17063	198.8	120424	0.7	0.0065
23	110.1039	16719	35.9	21762	0.1	0.0066
24	111.0800	17302	304.5	184210	1.0	0.0064
25	115.0538	18405	42.2	25298	0.1	0.0063
26	116.1067	17555	146.8	87670	0.5	0.0066
27	117.0695	17589	266.2	158506	0.9	0.0067
28	118.0853	17325	47.8	28436	0.2	0.0068
29	119.0851	18339	534.8	317368	1.8	0.0065
30	120.0886	17111	55.6	32892	0.2	0.0070
31	121.0647	21432	2403.2	1418394	8.0	0.0056
32	121.1008	20143	722.1	426140	2.4	0.0060
33	122.0680	16659	201.3	118490	0.7	0.0073
34	122.1019	13339	85.3	50208	0.3	0.0092
35	123.0801	18578	455.0	267170	1.5	0.0066
36	124.0848	14749	53.9	31556	0.2	0.0084
37	127.0723	18100	127.1	73994	0.4	0.0070
38	129.0693	16935	46.2	26772	0.2	0.0076
39	131.0853	20835	1403.7	807926	4.6	0.0063
40	132.0887	17667	158.2	90764	0.5	0.0075
41	133.1008	20593	996.1	569902	3.2	0.0065
42	134.1042	18111	109.7	62638	0.4	0.0074
43	135.0803	21740	1988.3	1131554	6.4	0.0062
44	135.1163	18922	332.1	188992	1.1	0.0071
45	136.0843	15607	198.8	112748	0.6	0.0087
46	136.1176	9498	34.3	19454	0.1	0.0143
47	137.0949	17411	85.8	48484	0.3	0.0079
48	143.0848	17052	46.0	25688	0.1	0.0084
49	144.0921	17253	44.5	24764	0.1	0.0084
50	145.1008	19811	897.7	498850	2.8	0.0073
51	146.1040	17882	107.2	59634	0.3	0.0082
52	147.0880	13573	68.5	38220	0.2	0.0108
53	147.1165	22580	1981.9	1105210	6.3	0.0065
54	148.0875	17592	74.0	41328	0.2	0.0084
55	148.1199	17964	221.3	123540	0.7	0.0082
56	149.0232	17756	42.3	23602	0.1	0.0084
57	149.0957	20375	863.5	481996	2.7	0.0073
58	149.1321	21532	1475.2	823414	4.7	0.0069
59	150.0999	16357	113.3	63076	0.4	0.0092

#	m/z	Res.	S/N	I	I %	FWHM
1	191.1430	24562		17677002	100.0	0.0078
2	192.1464	24690		2524094	14.3	0.0078
3	193.1493	24819		203050	1.1	0.0078

Display Report

#	m/z	Res.	S/N	I	I %	FWHM
60	150.1349	18151	178.1	99136	0.6	0.0083
61	158.1084	19224	104.4	57166	0.3	0.0082
62	159.1151	16021	36.8	20116	0.1	0.0099
63	161.0964	16588	51.7	28066	0.2	0.0097
64	161.1318	19162	111.5	60580	0.3	0.0084
65	162.1032	19311	204.9	111122	0.6	0.0084
66	163.1114	22712	2087.0	1129366	6.4	0.0072
67	163.1478	21303	1203.7	651308	3.7	0.0077
68	164.1144	19189	233.9	126402	0.7	0.0086
69	164.1506	18229	157.8	85258	0.5	0.0090
70	171.1160	18620	63.5	33758	0.2	0.0092
71	173.1323	24006	3274.0	1731648	9.8	0.0072
72	174.1353	19912	441.4	232766	1.3	0.0087
73	175.1389	16688	33.8	17768	0.1	0.0105
74	176.1190	20227	352.3	184494	1.0	0.0087
75	177.1253	16551	109.9	57396	0.3	0.0107
76	187.1475	19799	78.4	39232	0.2	0.0095
77	189.1267	20003	227.0	112504	0.6	0.0095
78	190.1321	15883	47.8	23550	0.1	0.0120
79	191.1176	8547	80.8	39634	0.2	0.0224
80	191.1426	24561	36025.9	17677002	100.0	0.0078
81	191.1730	11693	77.6	38064	0.2	0.0163
82	191.5933	15698	209.8	102862	0.6	0.0122
83	192.1463	26694	4999.3	2446866	13.8	0.0072
84	193.1489	19404	336.9	164210	0.9	0.0100
85	205.1583	21133	680.0	313036	1.8	0.0097
86	206.1614	18745	102.0	46734	0.3	0.0110
87	209.1530	19123	63.8	28816	0.2	0.0109
88	213.1246	22935	1083.1	480328	2.7	0.0093
89	214.1277	19615	149.2	65892	0.4	0.0109
90	217.1038	21221	41.0	17856	0.1	0.0102
91	219.1739	19329	62.4	26930	0.2	0.0113
92	227.1187	16991	53.4	22238	0.1	0.0134
93	231.1349	20719	495.2	202610	1.1	0.0112
94	232.1379	20263	77.8	31688	0.2	0.0115
95	241.1348	19808	142.3	56332	0.3	0.0122
96	243.1319	19460	54.8	21550	0.1	0.0125
97	247.1508	21550	386.5	149628	0.8	0.0115
98	248.1531	18898	75.7	29240	0.2	0.0131
99	301.1399	20900	112.0	30932	0.2	0.0144
100	307.1941	21244	135.9	36740	0.2	0.0145
101	313.1697	22221	111.8	29806	0.2	0.0141
102	319.2444	18645	71.5	18776	0.1	0.0171
103	321.1917	20942	264.6	69274	0.4	0.0153
104	321.6929	21357	103.9	27176	0.2	0.0151
105	323.1848	9724	78.3	20428	0.1	0.0332
106	325.1219	21008	193.6	50278	0.3	0.0155
107	325.1976	20800	357.5	92876	0.5	0.0156
108	326.2004	21100	102.5	26536	0.2	0.0155
109	338.3411	20490	189.6	48368	0.3	0.0165
110	339.2130	21256	493.1	125700	0.7	0.0160
111	340.2158	21948	143.4	36522	0.2	0.0155
112	347.1796	18498	114.3	28944	0.2	0.0188
113	381.2785	19245	218.6	55104	0.3	0.0198
114	399.2160	19787	92.4	23344	0.1	0.0202
115	400.2508	22006	271.6	68618	0.4	0.0182
116	400.7530	21532	139.9	35366	0.2	0.0186
117	408.2372	21135	134.7	34106	0.2	0.0193
118	408.7387	20596	75.5	19130	0.1	0.0198
119	409.2390	18662	154.6	39164	0.2	0.0219
120	409.7404	22286	85.1	21590	0.1	0.0184
121	410.2371	19992	71.8	18208	0.1	0.0205
122	469.2521	20278	141.8	35964	0.2	0.0231
123	497.2474	20875	143.2	36646	0.2	0.0238
