# Supplementary material for: Diastereodivergent Access to cis,cis- and cis,trans-Angular Triquinanes: Total Synthesis of (±)-Arnicenone
Source: J Am Chem Soc. 2026 Sep 22;148(39):41724–32. doi: 10.1021/jacs.6c09019 (PMC13651531; doi:10.1021/jacs.6c09019)
Supplement: Supplementary file 5 [file ja6c09019_si_003.zip › FID 29-44/29a/HTH-alpha demthyl desilyl alkyne enone (93)/HTH-alpha demethyl desilyl alkyne enone-13C.pdf]

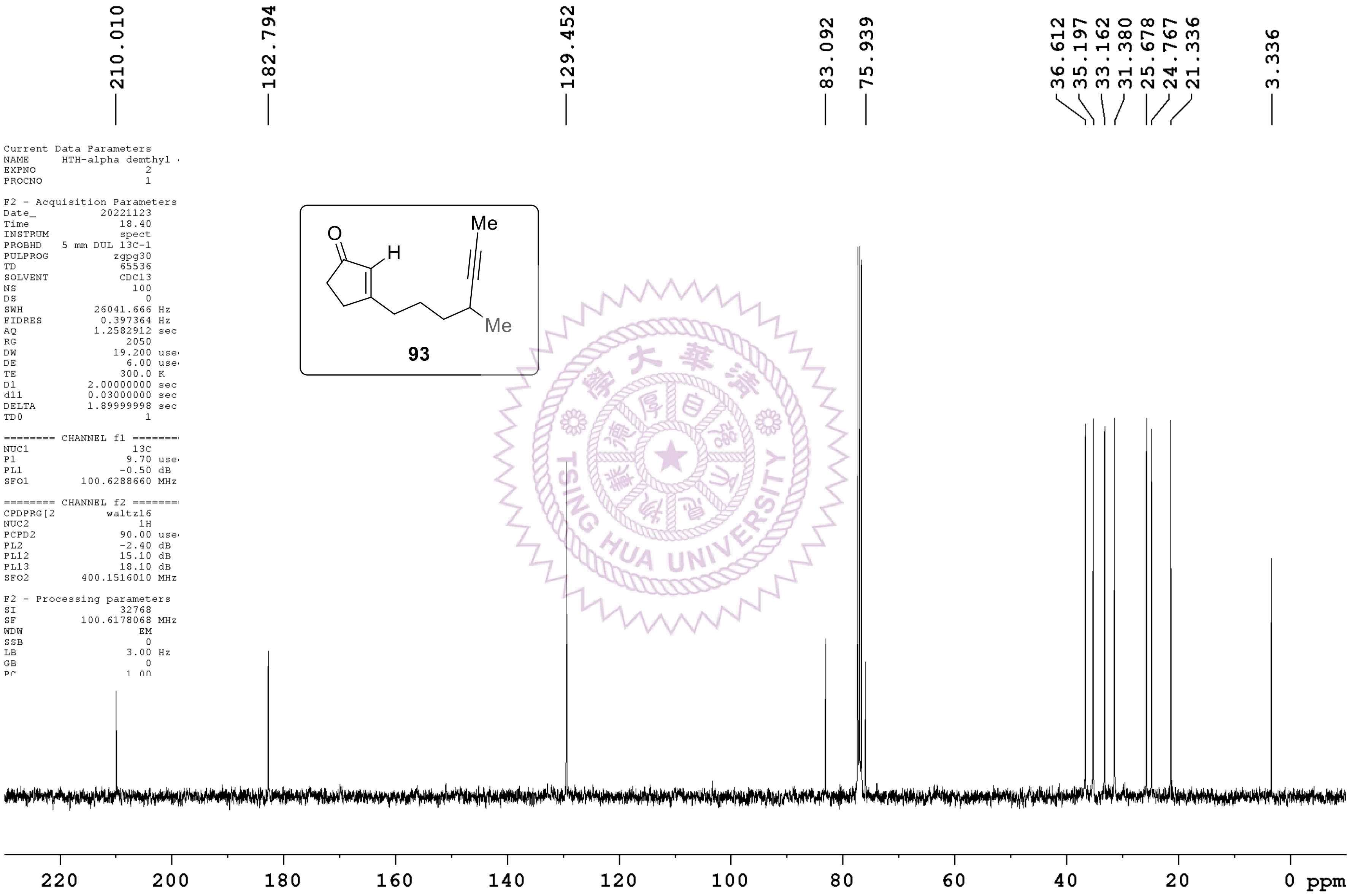
210.010
182.794
129.452
83.092
75.939
36.612
35.197
33.162
31.380
25.678
24.767
21.336
3.336
Current Data Parameters
NAME HTH-alpha demthyl
EXPNO 2
PROCNO 1
F2 - Acquisition Parameters
Date_ 20221123
Time 18.40
INSTRUM spect
PROBHD 5 mm DUL 13C-1
PULPROG zgpg30
TD 65536
SOLVENT CDCl3
NS 100
DS 0
SWH 26041.666 Hz
FIDRES 0.397364 Hz
AQ 1.2582912 sec
RG 2050
DW 19.200 use
DE 6.00 use
TE 300.0 K
D1 2.00000000 sec
d11 0.03000000 sec
DELTA 1.89999998 sec
TD0 1
======== CHANNEL f1 ========
NUC1 13C
P1 9.70 use
PL1 -0.50 dB
SFO1 100.6288660 MHz
======== CHANNEL f2 ========
CPDPRG[2 waltz16
NUC2 1H
PCPD2 90.00 use
PL2 -2.40 dB
PL12 15.10 dB
PL13 18.10 dB
SFO2 400.1516010 MHz
F2 - Processing parameters
SI 32768
SF 100.6178068 MHz
WDW EM
SSB 0
LB 3.00 Hz
GB 0
O
H
Me
Me
93
220
200
180
160
140
120
100
80
60
40
20
0 ppm
