# Supplementary material for: Diastereodivergent Access to cis,cis- and cis,trans-Angular Triquinanes: Total Synthesis of (±)-Arnicenone
Source: J Am Chem Soc. 2026 Sep 22;148(39):41724–32. doi: 10.1021/jacs.6c09019 (PMC13651531; doi:10.1021/jacs.6c09019)
Supplement: Supplementary file 6 [file ja6c09019_si_004.zip › FID SI/SI-3c/HTH-TIPS allene (aldehyde)/HTH-TIPS allene (aldehyde)-MS.pdf]

Display Report

Analysis Info

Analysis Name	D:\Data\nctu service\data\2023\20230620\HTH-46_RB7_01_36677.d	Acquisition Date	6/20/2023 1:01:44 AM
Method	Small molecule.m	Operator	NCTU
Sample Name	HTH-46	Instrument	impact HD 1819696.00164
Comment			

Acquisition Parameter

Source Type	ESI	Ion Polarity	Positive	Set Nebulizer	1.0 Bar
Focus	Active	Set Capillary	4500 V	Set Dry Heater	200 °C
Scan Begin	50 m/z	Set End Plate Offset	-500 V	Set Dry Gas	6.0 l/min
Scan End	1500 m/z	Set Charging Voltage	2000 V	Set Divert Valve	Waste
		Set Corona	0 nA	Set APCI Heater	0 °C

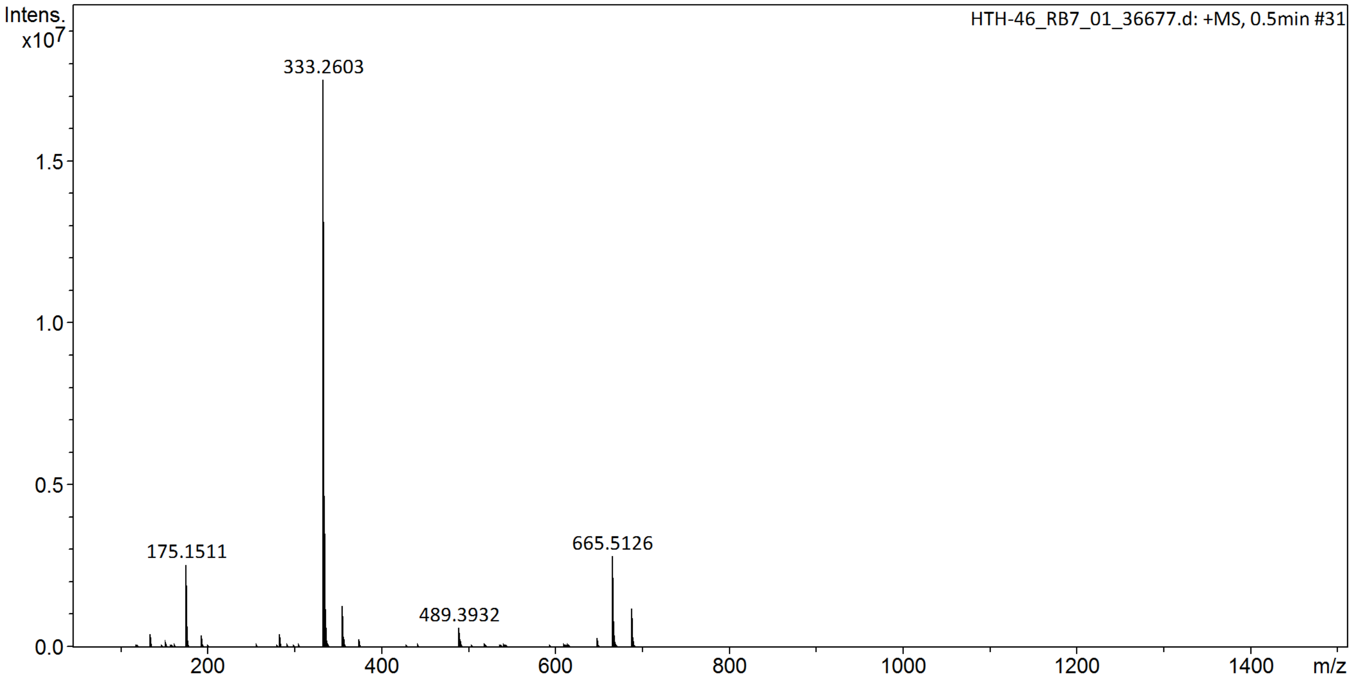

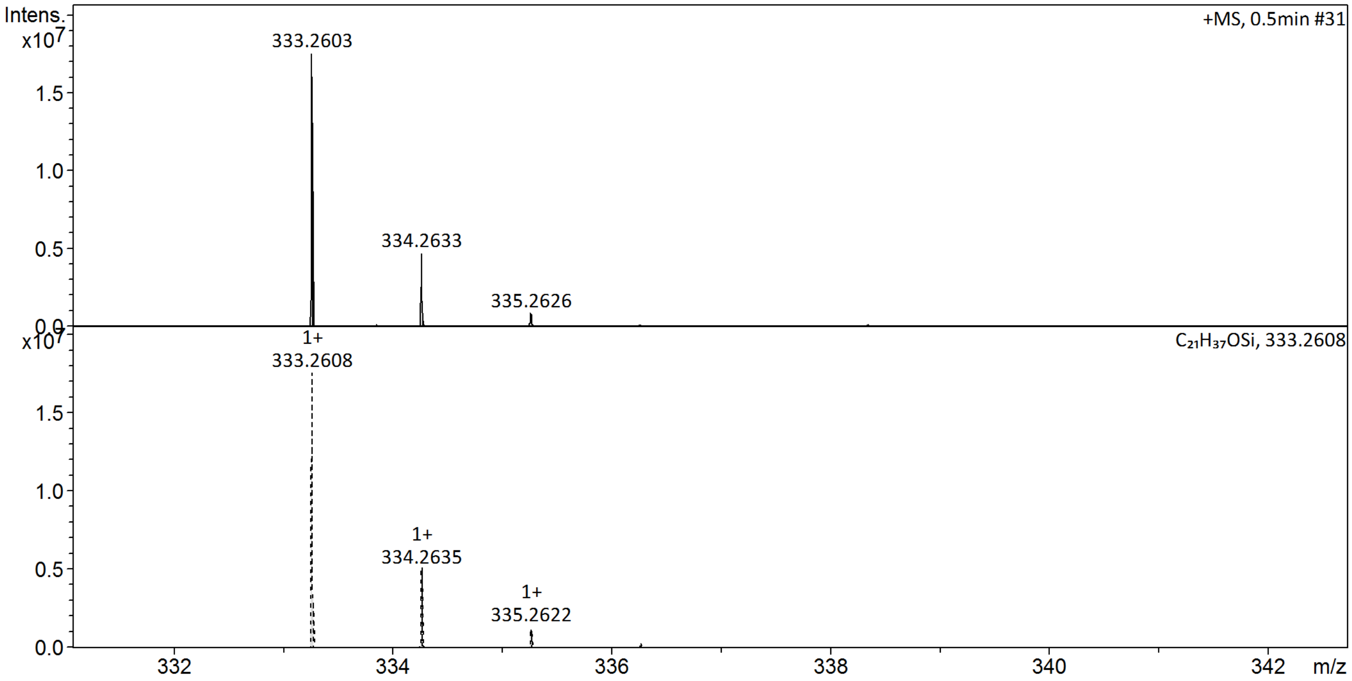

Display Report

Meas. m/z	#	Ion Formula	m/z	err [ppm]	mSigma	# Sigma	Score	rdb	e⁻ Conf	N-Rule	Adduct
333.2603	1	$C_{21}H_{37}OSi$	333.2608	1.5	15.9	1	100.00	4.5	even	ok	M+H

+MS, 0.5min #31

#	m/z	Res.	S/N	I	I %	FWHM
1	118.0858	17436	122.1	40262	0.2	0.0068
2	119.0881	16821	165.3	54676	0.3	0.0071
3	127.1478	19441	53.3	18060	0.1	0.0065
4	133.1040	20074	1136.8	391838	2.2	0.0066
5	134.1057	15458	105.8	36628	0.2	0.0087
6	147.1194	18768	163.2	58400	0.3	0.0078
7	151.1144	19398	367.6	132982	0.8	0.0078
8	157.1403	18016	119.8	44094	0.3	0.0087
9	159.1165	18107	114.0	42090	0.2	0.0088
10	161.1320	20008	187.5	69568	0.4	0.0081
11	175.1511	25991	6647.0	2544022	14.5	0.0067
12	176.1532	18544	767.7	294332	1.7	0.0095
13	177.1493	13991	185.2	71214	0.4	0.0127
14	193.1614	21680	912.5	360402	2.1	0.0089
15	194.1636	18017	127.0	50240	0.3	0.0108
16	200.2002	19857	80.3	32100	0.2	0.0101
17	213.1458	20928	43.9	17806	0.1	0.0102
18	223.0633	19542	57.9	23704	0.1	0.0114
19	256.2629	21808	182.3	76244	0.4	0.0118
20	265.2518	20288	66.4	27844	0.2	0.0131
21	280.2626	21119	128.3	54604	0.3	0.0133
22	282.2787	24174	961.8	409506	2.3	0.0117
23	283.2818	20986	177.0	75422	0.4	0.0135
24	284.2917	15328	50.2	21376	0.1	0.0185
25	291.2130	21549	167.7	71462	0.4	0.0135
26	292.2156	19779	45.6	19438	0.1	0.0148
27	299.0611	20855	80.9	34594	0.2	0.0143
28	301.1401	21148	47.8	20390	0.1	0.0142
29	302.2445	21786	62.2	26466	0.2	0.0139
30	304.2602	20659	189.4	80434	0.5	0.0147
31	305.2630	22622	43.6	18514	0.1	0.0135
32	309.2229	19950	41.6	17712	0.1	0.0155
33	325.2475	19361	43.3	18462	0.1	0.0168
34	333.2239	14180	108.9	46138	0.3	0.0235
35	333.2603	32613	41283.3	17495930	100.0	0.0102
36	333.3034	13997	51.6	21862	0.1	0.0238
37	333.8559	20495	160.1	67808	0.4	0.0163
38	334.2633	35875	11018.6	4665306	26.7	0.0093
39	335.2626	22052	1893.5	800746	4.6	0.0152
40	336.2624	18499	297.5	125710	0.7	0.0182
41	338.3408	20937	225.0	94836	0.5	0.0162
42	339.3440	21511	54.2	22794	0.1	0.0158
43	355.0690	22758	157.2	64676	0.4	0.0156
44	355.2425	30250	3120.9	1283870	7.3	0.0117
45	356.0696	20375	54.9	22566	0.1	0.0175
46	356.2449	23916	763.1	313606	1.8	0.0149
47	357.2437	16622	149.3	61256	0.4	0.0215
48	360.3227	21634	56.5	22996	0.1	0.0167
49	371.1002	21789	58.1	23190	0.1	0.0170
50	373.2523	23809	629.8	250896	1.4	0.0157
51	374.2548	21155	161.2	64120	0.4	0.0177
52	387.2129	19382	72.0	27996	0.2	0.0200
53	409.1953	22417	62.3	23442	0.1	0.0183
54	419.3138	20597	74.1	27258	0.2	0.0204
55	429.0875	22588	136.7	49062	0.3	0.0190
56	430.0876	20229	55.6	19950	0.1	0.0213
57	441.2960	20825	226.1	79512	0.5	0.0212
58	442.2991	20294	65.9	23084	0.1	0.0218
59	489.3932	27509	1841.6	590092	3.4	0.0178

#	m/z	Res.	S/N	I	I %	FWHM
1	333.2608	32613		17495930	100.0	0.0102
2	334.2635	32711		4937117	28.2	0.0102
3	335.2622	32809		1274917	7.3	0.0102
4	336.2633	32907		199839	1.1	0.0102

Display Report

#	m/z	Res.	S/N	I	I %	FWHM
60	490.3953	23634	741.1	237190	1.4	0.0207
61	491.3942	19857	246.0	78672	0.4	0.0247
62	492.3950	19271	58.5	18676	0.1	0.0256
63	494.5632	20684	55.6	17696	0.1	0.0239
64	503.1051	23315	148.2	46928	0.3	0.0216
65	504.1068	21715	69.7	22054	0.1	0.0232
66	508.5429	21110	74.9	23660	0.1	0.0241
67	519.1366	23107	224.5	70646	0.4	0.0225
68	520.1380	22802	109.7	34488	0.2	0.0228
69	521.1361	19523	77.6	24408	0.1	0.0267
70	536.1631	22463	218.5	68574	0.4	0.0239
71	536.5740	22578	87.3	27394	0.2	0.0238
72	537.1625	20509	115.9	36342	0.2	0.0262
73	538.1607	20327	84.9	26616	0.2	0.0265
74	541.1187	22584	341.6	107096	0.6	0.0240
75	542.1193	21899	169.5	53076	0.3	0.0248
76	543.1171	21331	119.5	37448	0.2	0.0255
77	593.1553	21962	111.1	34334	0.2	0.0270
78	594.1560	19435	64.2	19846	0.1	0.0306
79	610.1816	22497	239.6	73676	0.4	0.0271
80	611.1821	22533	142.2	43714	0.2	0.0271
81	612.1799	20855	100.3	30836	0.2	0.0294
82	614.5306	22475	302.7	92822	0.5	0.0273
83	615.1371	23464	148.0	45372	0.3	0.0262
84	615.5334	21641	148.7	45596	0.3	0.0284
85	616.1379	21468	82.1	25160	0.1	0.0287
86	617.1360	19716	57.2	17546	0.1	0.0313
87	647.5012	24311	931.0	283902	1.6	0.0266
88	648.5039	24004	535.6	163210	0.9	0.0270
89	649.5037	19812	192.1	58496	0.3	0.0328
90	665.5126	40470	9344.4	2800886	16.0	0.0164
91	666.5154	34238	4710.1	1410570	8.1	0.0195
92	667.1734	20025	66.3	19834	0.1	0.0333
93	667.5154	25287	1589.3	475370	2.7	0.0264
94	668.5157	20998	437.7	130814	0.7	0.0318
95	669.5120	14933	104.9	31318	0.2	0.0448
96	670.5925	21574	94.6	28238	0.2	0.0311
97	684.2007	21205	87.4	25690	0.1	0.0323
98	685.4322	21069	70.1	20596	0.1	0.0325
99	687.4943	33397	4106.7	1203786	6.9	0.0206
100	688.4969	28324	2134.6	625412	3.6	0.0243
101	689.1559	21369	71.7	20986	0.1	0.0323
102	689.4969	22585	797.4	233442	1.3	0.0305
103	690.4969	20552	213.3	62406	0.4	0.0336

HTH-46_RB7_01_36677.d
