# Supplementary material for: Diastereodivergent Access to cis,cis- and cis,trans-Angular Triquinanes: Total Synthesis of (±)-Arnicenone
Source: J Am Chem Soc. 2026 Sep 22;148(39):41724–32. doi: 10.1021/jacs.6c09019 (PMC13651531; doi:10.1021/jacs.6c09019)
Supplement: Supplementary file 6 [file ja6c09019_si_004.zip › FID SI/SI-3c/HTH-TIPS allene (aldehyde)/HTH-TIPS allene (aldehyde)-13C.pdf]

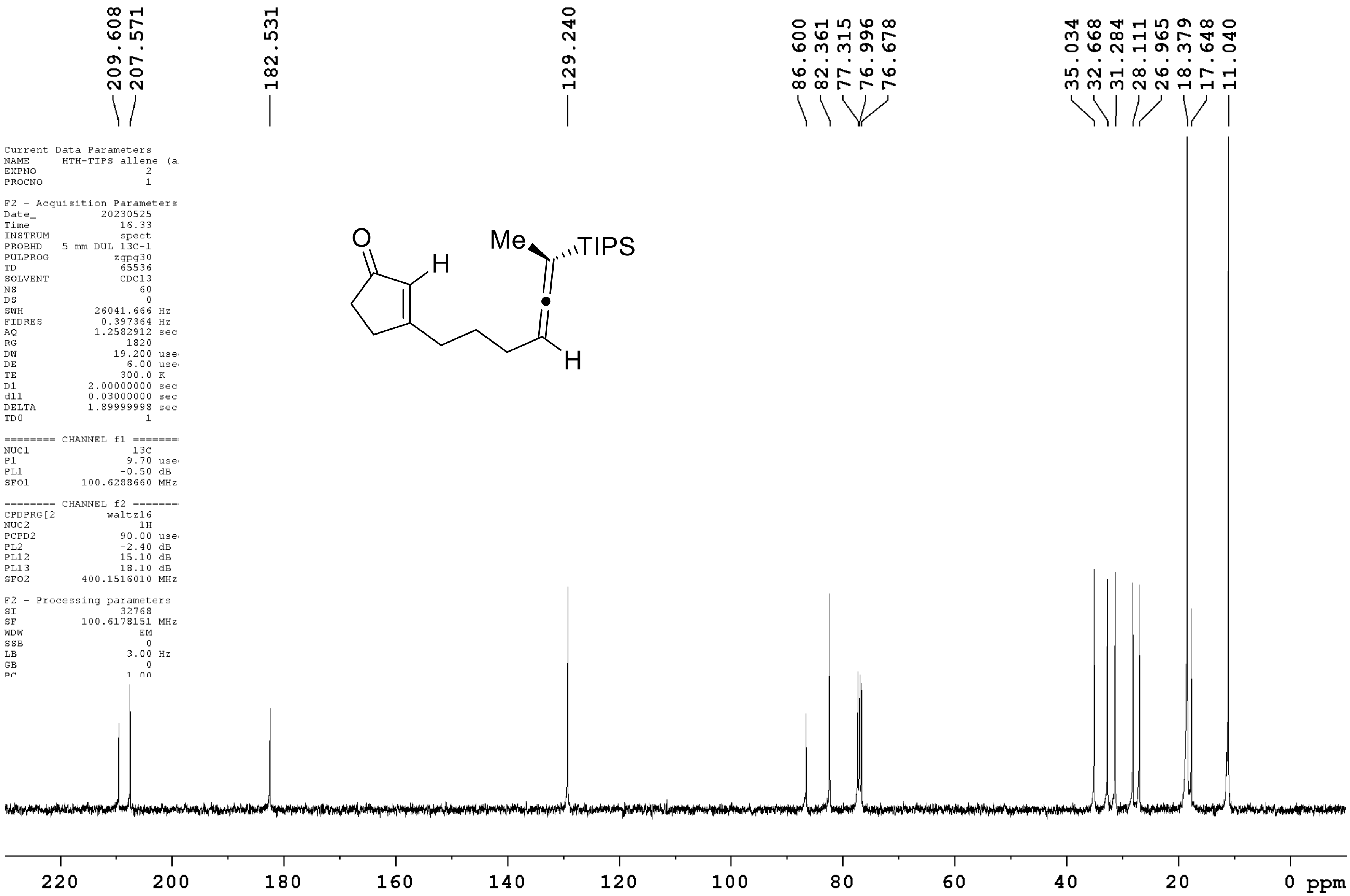

209.608
207.571
182.531
129.240
86.600
82.361
77.315
76.996
76.678
35.034
32.668
31.284
28.111
26.965
18.379
17.648
11.040
Current Data Parameters
NAME HTH-TIPS allene (a
EXPNO 2
PROCNO 1
F2 - Acquisition Parameters
Date_ 20230525
Time 16.33
INSTRUM spect
PROBHD 5 mm DUL 13C-1
PULPROG zgpg30
TD 65536
SOLVENT CDCl3
NS 60
DS 0
SWH 26041.666 Hz
FIDRES 0.397364 Hz
AQ 1.2582912 sec
RG 1820
DW 19.200 use
DE 6.00 use
TE 300.0 K
D1 2.00000000 sec
d11 0.03000000 sec
DELTA 1.89999998 sec
TD0 1
======== CHANNEL f1 ========
NUC1 13C
P1 9.70 use
PL1 -0.50 dB
SFO1 100.6288660 MHz
======== CHANNEL f2 ========
CPDPRG[2 waltz16
NUC2 1H
PCPD2 90.00 use
PL2 -2.40 dB
PL12 15.10 dB
PL13 18.10 dB
SFO2 400.1516010 MHz
F2 - Processing parameters
SI 32768
SF 100.6178151 MHz
WDW EM
SSB 0
LB 3.00 Hz
GB 0
O
H
Me
TIPS
H
220 200 180 160 140 120 100 80 60 40 20 0 ppm
