# Supplementary material for: Diastereodivergent Access to cis,cis- and cis,trans-Angular Triquinanes: Total Synthesis of (±)-Arnicenone
Source: J Am Chem Soc. 2026 Sep 22;148(39):41724–32. doi: 10.1021/jacs.6c09019 (PMC13651531; doi:10.1021/jacs.6c09019)
Supplement: Supplementary file 6 [file ja6c09019_si_004.zip › FID SI/SI-3c/HTH-TIPS allene (aldehyde)/HTH-TIPS allene (aldehyde)-1H.pdf]

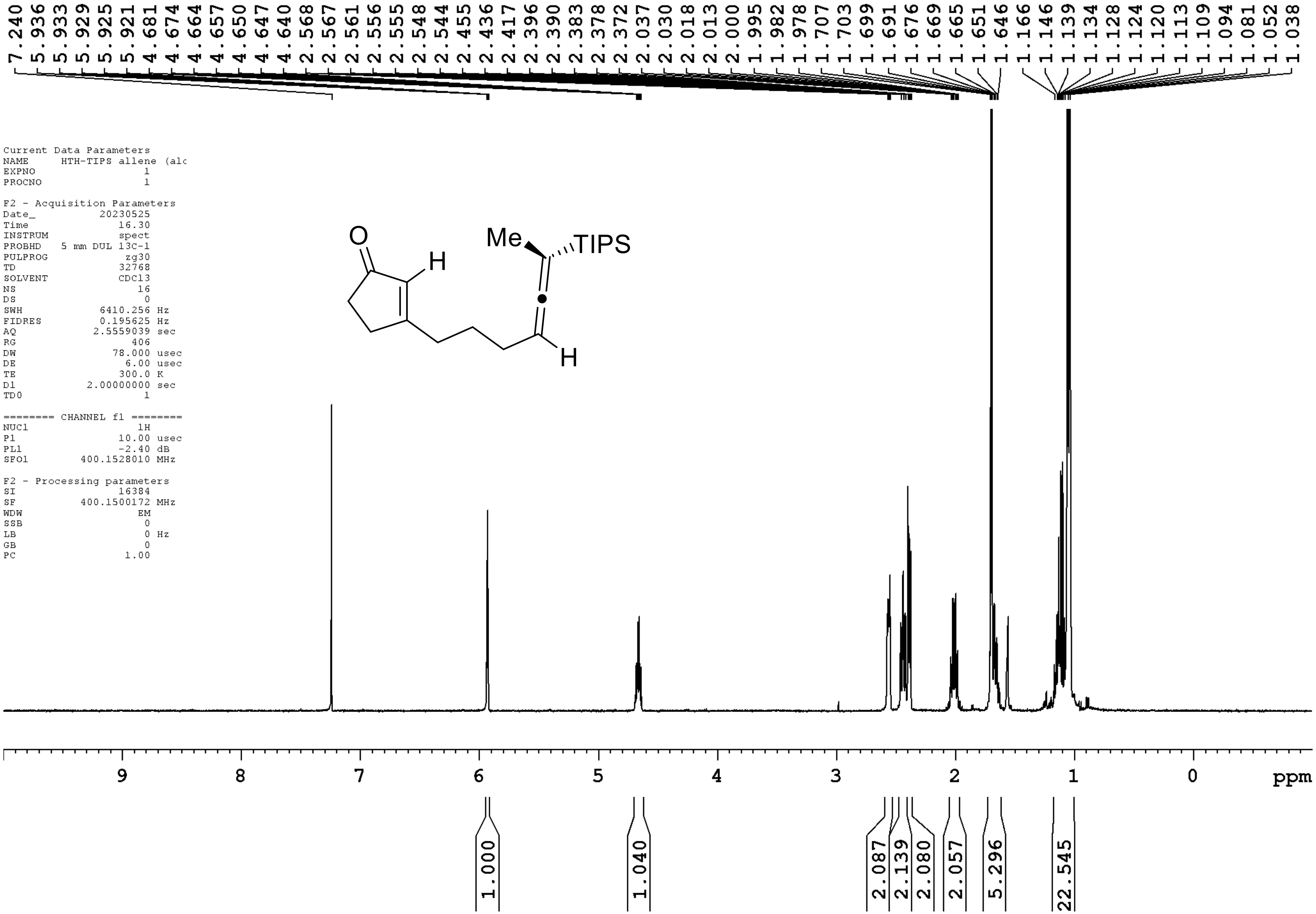

Current Data Parameters
NAME HTH-TIPS allene (alc
EXPNO 1
PROCNO 1

F2 - Acquisition Parameters
Date_ 20230525
Time 16.30
INSTRUM spect
PROBHD 5 mm DUL 13C-1
PULPROG zg30
TD 32768
SOLVENT CDCl3
NS 16
DS 0
SWH 6410.256 Hz
FIDRES 0.195625 Hz
AQ 2.5559039 sec
RG 406
DW 78.000 usec
DE 6.00 usec
TE 300.0 K
D1 2.00000000 sec
TD0 1

======== CHANNEL f1 ========
NUC1 1H
P1 10.00 usec
PL1 -2.40 dB
SFO1 400.1528010 MHz

F2 - Processing parameters
SI 16384
SF 400.1500172 MHz
WDW EM
SSB 0
LB 0 Hz
GB 0
PC 1.00
