# Supplementary material for: Diastereodivergent Access to cis,cis- and cis,trans-Angular Triquinanes: Total Synthesis of (±)-Arnicenone
Source: J Am Chem Soc. 2026 Sep 22;148(39):41724–32. doi: 10.1021/jacs.6c09019 (PMC13651531; doi:10.1021/jacs.6c09019)
Supplement: Supplementary file 6 [file ja6c09019_si_004.zip › FID SI/SI-2/HTH-cyclopentenone acetal/HTH-cyclopentenone acetal-MS.pdf]

Display Report

Analysis Info

Analysis Name	D:\Data\nctu service\data\2023\20230620\HTH-23_GC7_01_36654.d	Acquisition Date	6/19/2023 11:19:00 PM
Method	Small molecule.m	Operator	NCTU
Sample Name	HTH-23	Instrument	impact HD 1819696.00164
Comment			

Acquisition Parameter

Source Type	ESI	Ion Polarity	Positive	Set Nebulizer	1.0 Bar
Focus	Active	Set Capillary	4500 V	Set Dry Heater	200 °C
Scan Begin	50 m/z	Set End Plate Offset	-500 V	Set Dry Gas	6.0 l/min
Scan End	1500 m/z	Set Charging Voltage	2000 V	Set Divert Valve	Waste
		Set Corona	0 nA	Set APCI Heater	0 °C

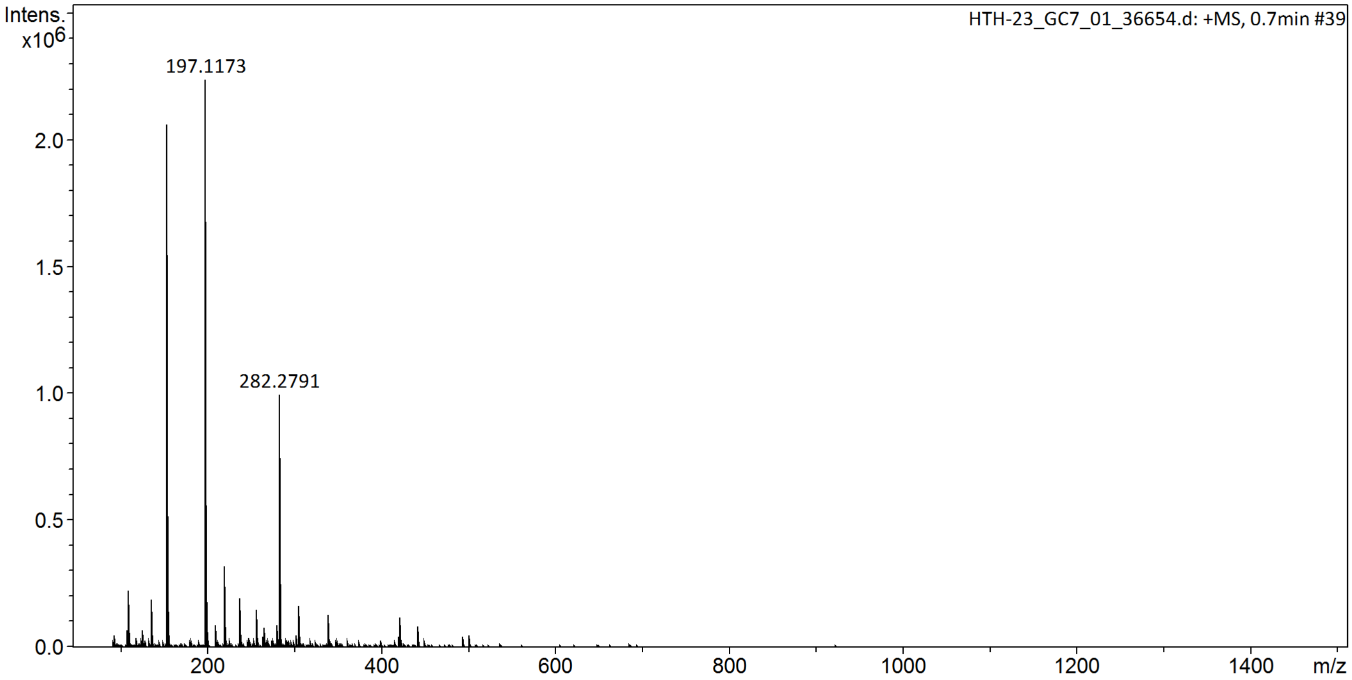

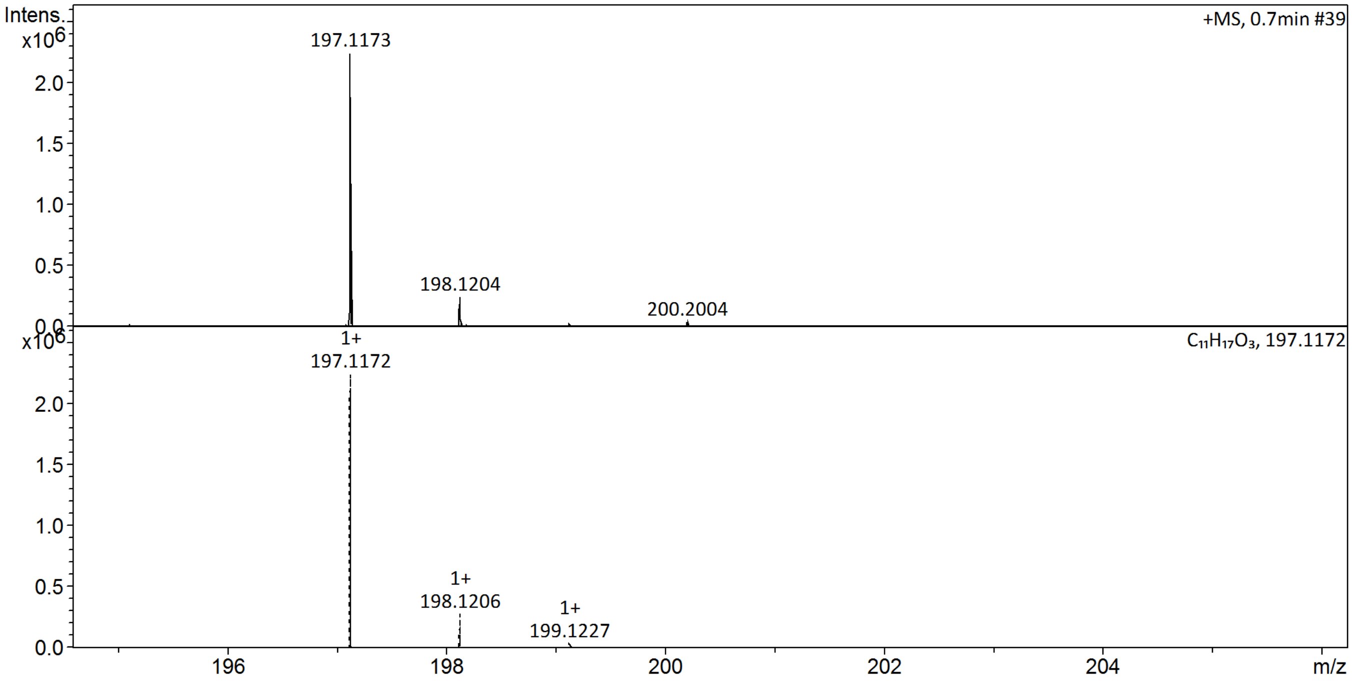

Display Report

Meas. m/z	#	Ion Formula	m/z	err [ppm]	mSigma	# Sigma	Score	rdb	e¯ Conf	N-Rule	Adduct
197.1173	1	C11H17O3	197.1172	0.5	9.0	1	100.00	3.5	even	ok	M+H

+MS, 0.7min #39

#	m/z	Res.	S/N	I	I %	FWHM
1	90.9765	16868	31.0	9278	0.4	0.0054
2	91.0541	15305	62.2	18630	0.8	0.0059
3	93.0697	16429	150.2	44978	2.0	0.0057
4	95.0853	16455	31.5	9444	0.4	0.0058
5	97.0645	18450	43.8	13148	0.6	0.0053
6	97.1010	16324	23.2	6972	0.3	0.0059
7	99.0441	16506	19.3	5814	0.3	0.0060
8	105.0699	17014	17.5	5254	0.2	0.0062
9	107.0491	18802	21.5	6476	0.3	0.0057
10	107.0852	17430	226.5	68154	3.0	0.0061
11	108.0885	15743	20.5	6180	0.3	0.0069
12	109.0646	18086	733.4	220740	9.9	0.0060
13	109.1009	16686	43.7	13152	0.6	0.0065
14	110.0679	16610	58.0	17458	0.8	0.0066
15	111.0800	17970	36.2	10894	0.5	0.0062
16	111.1165	18198	29.7	8958	0.4	0.0061
17	113.0591	19400	20.7	6234	0.3	0.0058
18	114.0911	17582	22.9	6912	0.3	0.0065
19	115.0751	15942	24.2	7298	0.3	0.0072
20	117.0696	19050	79.7	24046	1.1	0.0061
21	118.0861	17671	116.0	35054	1.6	0.0067
22	119.0864	12795	20.6	6234	0.3	0.0093
23	121.1011	17495	33.2	10052	0.4	0.0069
24	122.0965	18774	36.6	11052	0.5	0.0065
25	123.0384	18711	59.9	18118	0.8	0.0066
26	123.0803	18804	67.0	20240	0.9	0.0065
27	123.1168	21392	30.0	9082	0.4	0.0058
28	125.0595	18622	17.4	5264	0.2	0.0067
29	125.0959	17930	229.3	69348	3.1	0.0070
30	127.0367	16828	24.2	7344	0.3	0.0075
31	127.0741	18591	25.5	7740	0.3	0.0068
32	127.1480	19135	86.3	26162	1.2	0.0066
33	128.1069	17682	24.8	7524	0.3	0.0072
34	132.0429	21006	64.4	19556	0.9	0.0063
35	133.1012	16410	32.0	9712	0.4	0.0081
36	134.5457	19103	103.0	31284	1.4	0.0070
37	135.0803	18676	623.2	189218	8.5	0.0072
38	135.1165	18933	32.0	9710	0.4	0.0071
39	136.0836	18956	62.0	18854	0.8	0.0072
40	137.0954	18549	32.2	9796	0.4	0.0074
41	139.0747	18269	25.1	7618	0.3	0.0076
42	139.1114	18506	19.6	5970	0.3	0.0075
43	140.0682	17476	42.5	12914	0.6	0.0080
44	141.0908	15680	18.2	5544	0.2	0.0090
45	142.1222	19795	28.3	8616	0.4	0.0072
46	143.5511	19288	59.4	18062	0.8	0.0074
47	149.0229	19171	54.1	16462	0.7	0.0078
48	149.1320	19767	28.3	8608	0.4	0.0075
49	151.1116	19410	32.2	9792	0.4	0.0078
50	151.1482	19568	21.6	6578	0.3	0.0077
51	153.0557	12298	16.8	5108	0.2	0.0124
52	153.0911	24053	6778.3	2059546	92.0	0.0064
53	154.0942	20052	626.2	190286	8.5	0.0077
54	155.0980	13339	46.9	14244	0.6	0.0116
55	156.1380	21586	25.1	7622	0.3	0.0072
56	158.9641	21975	24.4	7380	0.3	0.0072
57	161.0954	19291	19.7	5976	0.3	0.0084
58	161.1321	19859	17.8	5392	0.2	0.0081
59	163.1474	20945	21.3	6446	0.3	0.0078

#	m/z	Res.	S/N	I	I %	FWHM
1	197.1172	27062		2238120	100.0	0.0073
2	198.1206	27200		273210	12.2	0.0073
3	199.1227	27337		29028	1.3	0.0073

Display Report

#	m/z	Res.	S/N	I	I %	FWHM
60	165.1635	17262	24.5	7414	0.3	0.0096
61	167.0332	18121	25.2	7634	0.3	0.0092
62	167.1063	20404	19.5	5920	0.3	0.0082
63	169.0850	19837	45.9	13868	0.6	0.0085
64	169.1225	17542	28.6	8632	0.4	0.0096
65	170.1539	19894	25.8	7790	0.3	0.0086
66	171.1011	20608	29.9	9036	0.4	0.0083
67	173.0785	20805	30.2	9110	0.4	0.0083
68	179.1064	19351	52.3	15756	0.7	0.0093
69	181.0693	20486	72.8	21922	1.0	0.0088
70	183.1003	20822	29.2	8784	0.4	0.0088
71	184.1693	20645	28.1	8468	0.4	0.0089
72	185.1166	20464	19.7	5932	0.3	0.0090
73	190.0742	23184	57.3	17250	0.8	0.0082
74	192.1376	19955	25.0	7514	0.3	0.0096
75	193.0835	20195	17.3	5212	0.2	0.0096
76	193.1228	21131	16.9	5074	0.2	0.0091
77	194.1159	19142	28.2	8484	0.4	0.0101
78	195.0998	17145	29.2	8768	0.4	0.0114
79	195.1373	19549	22.6	6790	0.3	0.0100
80	197.0798	16706	33.8	10148	0.5	0.0118
81	197.1173	27062	7446.4	2238120	100.0	0.0073
82	198.1204	20990	794.8	238760	10.7	0.0094
83	198.1850	22324	26.6	7976	0.4	0.0089
84	199.1228	19317	81.1	24336	1.1	0.0103
85	200.2004	20715	122.0	36626	1.6	0.0097
86	209.0787	20126	26.9	8026	0.4	0.0104
87	209.1145	20937	297.6	88934	4.0	0.0100
88	210.1172	19019	33.6	10020	0.4	0.0110
89	211.0953	17777	23.0	6866	0.3	0.0119
90	211.1314	20915	49.7	14826	0.7	0.0101
91	212.2002	18860	17.8	5300	0.2	0.0113
92	213.1122	20440	17.3	5140	0.2	0.0104
93	213.1475	17617	20.2	6018	0.3	0.0121
94	215.1258	23356	17.2	5114	0.2	0.0092
95	217.1049	18226	34.5	10254	0.5	0.0119
96	219.0988	22325	1082.9	321212	14.4	0.0098
97	220.1022	20321	131.7	39074	1.7	0.0108
98	223.0941	20012	31.7	9384	0.4	0.0111
99	223.1321	18893	25.9	7684	0.3	0.0118
100	224.1277	18620	37.5	11118	0.5	0.0120
101	225.1095	20347	75.4	22320	1.0	0.0111
102	226.2164	22082	19.6	5792	0.3	0.0102
103	226.9508	21928	20.6	6106	0.3	0.0103
104	227.1264	17726	21.7	6432	0.3	0.0128
105	228.2315	21060	43.6	12876	0.6	0.0108
106	233.1150	19532	20.6	6074	0.3	0.0119
107	235.0941	17781	17.9	5278	0.2	0.0132
108	235.1311	19625	38.0	11180	0.5	0.0120
109	237.1094	21375	651.6	191356	8.5	0.0111
110	237.1461	14760	37.6	11050	0.5	0.0161
111	238.1124	21026	85.5	25114	1.1	0.0113
112	239.0886	17709	33.3	9772	0.4	0.0135
113	239.1237	14474	23.9	7026	0.3	0.0165
114	241.1045	19751	41.2	12084	0.5	0.0122
115	245.1139	22195	28.7	8408	0.4	0.0110
116	245.2262	19987	59.3	17332	0.8	0.0123
117	247.2415	20370	121.1	35414	1.6	0.0121
118	248.2447	19001	24.3	7096	0.3	0.0131
119	249.1099	15229	18.6	5454	0.2	0.0164
120	249.1569	18276	48.0	14036	0.6	0.0136
121	251.0876	19380	24.9	7300	0.3	0.0130
122	251.1260	18705	46.2	13540	0.6	0.0134
123	251.1616	20257	21.1	6188	0.3	0.0124
124	253.1051	19866	24.2	7066	0.3	0.0127
125	253.1408	19562	81.4	23798	1.1	0.0129

Display Report

#	m/z	Res.	S/N	I	I %	FWHM
126	255.1200	18244	22.4	6550	0.3	0.0140
127	256.2630	21035	504.9	147498	6.6	0.0122
128	257.2659	20830	87.9	25666	1.1	0.0124
129	263.1620	21077	20.4	5932	0.3	0.0125
130	263.2361	20485	141.0	41026	1.8	0.0129
131	264.2403	21727	34.2	9950	0.4	0.0122
132	265.1043	19301	38.6	11224	0.5	0.0137
133	265.1409	21020	31.3	9098	0.4	0.0126
134	265.1785	20562	23.4	6802	0.3	0.0129
135	265.2521	20971	259.8	75492	3.4	0.0126
136	266.2561	18851	47.1	13686	0.6	0.0141
137	267.1196	22753	64.2	18672	0.8	0.0117
138	267.1558	19884	26.6	7722	0.3	0.0134
139	269.0998	18101	17.7	5136	0.2	0.0149
140	269.1361	21210	66.7	19374	0.9	0.0127
141	271.1158	18574	30.8	8954	0.4	0.0146
142	274.2734	21888	96.5	28024	1.3	0.0125
143	275.1633	20584	71.2	20672	0.9	0.0134
144	275.2759	19741	19.2	5576	0.2	0.0139
145	277.1043	24050	38.9	11274	0.5	0.0115
146	277.1397	19180	22.0	6388	0.3	0.0144
147	277.1783	20888	31.2	9052	0.4	0.0133
148	278.2462	18671	44.6	12904	0.6	0.0149
149	279.1199	19547	40.6	11746	0.5	0.0143
150	279.1569	19759	46.1	13344	0.6	0.0141
151	280.2629	20982	303.5	87900	3.9	0.0134
152	281.0995	20408	20.6	5952	0.3	0.0138
153	281.1357	19266	32.0	9260	0.4	0.0146
154	281.1719	19905	110.7	32058	1.4	0.0141
155	281.2657	20491	62.6	18124	0.8	0.0137
156	282.1746	18505	22.0	6380	0.3	0.0152
157	282.2791	25922	3445.5	997296	44.6	0.0109
158	283.1159	16917	30.1	8710	0.4	0.0167
159	283.1520	20562	27.7	8004	0.4	0.0138
160	283.2818	21490	638.2	184628	8.2	0.0132
161	284.2912	15945	142.1	41146	1.8	0.0178
162	285.1345	15843	29.7	8590	0.4	0.0180
163	285.2941	13802	24.5	7092	0.3	0.0207
164	287.0856	21562	42.2	12216	0.5	0.0133
165	290.2684	20460	77.5	22416	1.0	0.0142
166	291.1343	21340	65.4	18898	0.8	0.0136
167	292.1351	19373	22.1	6398	0.3	0.0151
168	293.1363	20375	45.2	13056	0.6	0.0144
169	293.1735	21226	85.2	24624	1.1	0.0138
170	294.1766	23566	20.1	5800	0.3	0.0125
171	294.2418	22572	18.8	5442	0.2	0.0130
172	295.1151	21852	50.1	14490	0.6	0.0135
173	295.1512	22051	48.4	13982	0.6	0.0134
174	297.1317	17900	27.8	8030	0.4	0.0166
175	297.1668	18658	24.3	7018	0.3	0.0159
176	299.0797	22067	28.8	8290	0.4	0.0136
177	299.1595	20414	48.7	14024	0.6	0.0147
178	301.1391	18662	42.9	12342	0.6	0.0161
179	302.2449	22296	159.6	45854	2.0	0.0136
180	303.1550	18013	17.8	5114	0.2	0.0168
181	303.2480	18806	30.4	8728	0.4	0.0161
182	304.2603	22421	563.7	161958	7.2	0.0136
183	305.1352	23571	26.0	7472	0.3	0.0129
184	305.1714	21771	70.8	20310	0.9	0.0140
185	305.2641	20825	107.6	30868	1.4	0.0147
186	307.1284	15003	45.0	12900	0.6	0.0205
187	307.1876	21074	30.3	8698	0.4	0.0146
188	309.1302	20239	44.8	12804	0.6	0.0153
189	309.1666	18045	30.2	8646	0.4	0.0171
190	309.2037	21869	41.6	11910	0.5	0.0141
191	310.3095	20098	18.9	5410	0.2	0.0154

Display Report

#	m/z	Res.	S/N	I	I %	FWHM
192	311.1469	14903	43.3	12360	0.6	0.0209
193	311.1841	14905	20.5	5856	0.3	0.0209
194	313.1613	20557	24.6	7032	0.3	0.0152
195	315.1546	23506	25.7	7348	0.3	0.0134
196	318.2393	21058	45.2	12904	0.6	0.0151
197	318.2995	19356	68.5	19542	0.9	0.0164
198	320.2553	16761	27.5	7842	0.4	0.0191
199	321.1315	17665	33.7	9596	0.4	0.0182
200	321.1667	17440	23.1	6576	0.3	0.0184
201	321.3143	22522	24.9	7094	0.3	0.0143
202	323.1459	20031	49.2	14014	0.6	0.0161
203	323.1822	20054	27.0	7694	0.3	0.0161
204	325.1295	9234	18.1	5156	0.2	0.0352
205	325.1617	20977	38.9	11088	0.5	0.0155
206	325.1974	21854	30.9	8806	0.4	0.0149
207	325.2471	19776	43.5	12374	0.6	0.0164
208	327.1423	18072	25.3	7198	0.3	0.0181
209	327.1783	15957	18.8	5366	0.2	0.0205
210	329.1572	17970	34.8	9918	0.4	0.0183
211	333.1664	21600	19.1	5450	0.2	0.0154
212	335.1463	20076	19.7	5602	0.3	0.0167
213	335.1811	17500	25.2	7182	0.3	0.0192
214	337.1625	19180	39.3	11192	0.5	0.0176
215	338.3407	22756	454.0	129416	5.8	0.0149
216	339.1435	16785	29.3	8338	0.4	0.0202
217	339.1755	16193	23.0	6540	0.3	0.0209
218	339.3442	23105	113.7	32354	1.4	0.0147
219	340.2822	19901	27.2	7736	0.3	0.0171
220	340.3490	20169	19.3	5504	0.2	0.0169
221	341.1574	15131	29.0	8254	0.4	0.0225
222	341.2663	21953	58.8	16746	0.7	0.0155
223	343.1856	19145	41.6	11848	0.5	0.0179
224	347.2282	20668	49.0	13978	0.6	0.0168
225	349.1619	21196	28.8	8220	0.4	0.0165
226	349.1973	22003	79.6	22676	1.0	0.0159
227	351.1424	19626	18.7	5340	0.2	0.0179
228	351.1767	17412	32.4	9228	0.4	0.0202
229	351.2121	17331	20.0	5692	0.3	0.0203
230	353.1572	21370	32.4	9222	0.4	0.0165
231	353.1923	15231	25.3	7218	0.3	0.0232
232	353.2633	19313	17.8	5064	0.2	0.0183
233	355.3356	22200	40.0	11400	0.5	0.0160
234	360.3227	19296	80.6	22980	1.0	0.0187
235	361.3258	21429	20.3	5772	0.3	0.0169
236	363.1792	20107	20.2	5738	0.3	0.0181
237	365.1557	13688	22.3	6342	0.3	0.0267
238	365.1921	17546	27.8	7920	0.4	0.0208
239	367.1720	17155	34.3	9760	0.4	0.0214
240	367.2087	15911	19.5	5530	0.2	0.0231
241	369.1884	18906	39.0	11084	0.5	0.0195
242	369.2968	19360	33.6	9558	0.4	0.0191
243	373.3449	22593	64.7	18432	0.8	0.0165
244	379.1735	18055	23.6	6730	0.3	0.0210
245	381.1878	17648	34.9	9952	0.4	0.0216
246	383.1657	18200	19.9	5660	0.3	0.0211
247	383.2033	14948	19.5	5556	0.2	0.0256
248	385.1818	18218	23.0	6558	0.3	0.0211
249	387.2115	14419	20.5	5856	0.3	0.0269
250	391.3559	21690	29.4	8406	0.4	0.0180
251	393.1881	19237	19.7	5624	0.3	0.0204
252	393.2244	19622	41.7	11898	0.5	0.0200
253	395.2041	18893	19.5	5564	0.2	0.0209
254	397.1818	16344	18.3	5230	0.2	0.0243
255	399.2492	20328	98.6	28194	1.3	0.0196
256	400.2530	23150	21.9	6280	0.3	0.0173
257	401.2126	14598	18.6	5320	0.2	0.0275

Display Report

#	m/z	Res.	S/N	I	I %	FWHM
258	407.2063	16983	19.1	5480	0.2	0.0240
259	409.1867	14241	21.6	6192	0.3	0.0287
260	409.2166	15814	25.3	7264	0.3	0.0259
261	412.1996	24871	20.2	5810	0.3	0.0166
262	413.2647	20267	21.5	6184	0.3	0.0204
263	415.2074	19949	55.4	15922	0.7	0.0208
264	419.3137	22393	136.8	39248	1.8	0.0187
265	420.3180	20458	40.6	11658	0.5	0.0205
266	421.2311	22173	406.7	116862	5.2	0.0190
267	422.2345	22406	82.3	23680	1.1	0.0188
268	423.1956	17235	32.6	9386	0.4	0.0246
269	423.2353	27248	33.8	9708	0.4	0.0155
270	425.2137	18631	30.7	8844	0.4	0.0228
271	429.3169	23743	17.7	5084	0.2	0.0181
272	435.2049	15265	18.5	5338	0.2	0.0285
273	437.2113	12336	26.0	7514	0.3	0.0354
274	439.2286	21316	21.8	6304	0.3	0.0206
275	441.2100	18825	19.1	5530	0.2	0.0234
276	441.2961	22854	287.3	83238	3.7	0.0193
277	442.2990	20463	78.7	22828	1.0	0.0216
278	449.3586	21539	70.5	20560	0.9	0.0209
279	450.3609	20722	21.7	6336	0.3	0.0217
280	473.3438	22212	18.8	5558	0.2	0.0213
281	478.3885	23339	22.9	6762	0.3	0.0205
282	493.3845	21738	131.0	39114	1.7	0.0227
283	494.3880	21917	43.4	12950	0.6	0.0226
284	494.5638	22760	17.7	5290	0.2	0.0217
285	495.1253	19544	20.8	6208	0.3	0.0253
286	500.2523	22004	156.3	46920	2.1	0.0227
287	501.2559	19603	49.8	14956	0.7	0.0256
288	508.5444	19221	28.4	8572	0.4	0.0265
289	517.3692	23270	20.1	6074	0.3	0.0222
290	522.2346	27696	26.6	8052	0.4	0.0189
291	536.5745	25500	43.0	13088	0.6	0.0210
292	537.4095	21326	25.4	7730	0.3	0.0252
293	561.3958	19112	22.8	6914	0.3	0.0294
294	622.0251	29854	27.9	8136	0.4	0.0208
295	647.4571	23876	18.0	5108	0.2	0.0271
296	649.4491	21395	21.5	6068	0.3	0.0304
297	663.4509	19468	24.7	6860	0.3	0.0341
298	685.4340	21433	38.8	10454	0.5	0.0320
299	686.4364	23652	19.6	5264	0.2	0.0290
300	922.0066	24307	30.2	5856	0.3	0.0379
