# Supplementary material for: Diastereodivergent Access to cis,cis- and cis,trans-Angular Triquinanes: Total Synthesis of (±)-Arnicenone
Source: J Am Chem Soc. 2026 Sep 22;148(39):41724–32. doi: 10.1021/jacs.6c09019 (PMC13651531; doi:10.1021/jacs.6c09019)
Supplement: Supplementary file 6 [file ja6c09019_si_004.zip › FID SI/SI-3a/HTH-TMS allene (aldehyde)/HTH-TMS allene (aldehyde)-MS.pdf]

Display Report

Analysis Info

Analysis Name	D:\Data\nctu service\data\2023\20230620\HTH-18_GC2_01_36649.d	Acquisition Date	6/19/2023 10:57:08 PM
Method	Small molecule.m	Operator	NCTU
Sample Name	HTH-18	Instrument	impact HD 1819696.00164
Comment			

Acquisition Parameter

Source Type	ESI	Ion Polarity	Positive	Set Nebulizer	1.0 Bar
Focus	Active	Set Capillary	4500 V	Set Dry Heater	200 °C
Scan Begin	50 m/z	Set End Plate Offset	-500 V	Set Dry Gas	6.0 l/min
Scan End	1500 m/z	Set Charging Voltage	2000 V	Set Divert Valve	Waste
		Set Corona	0 nA	Set APCI Heater	0 °C

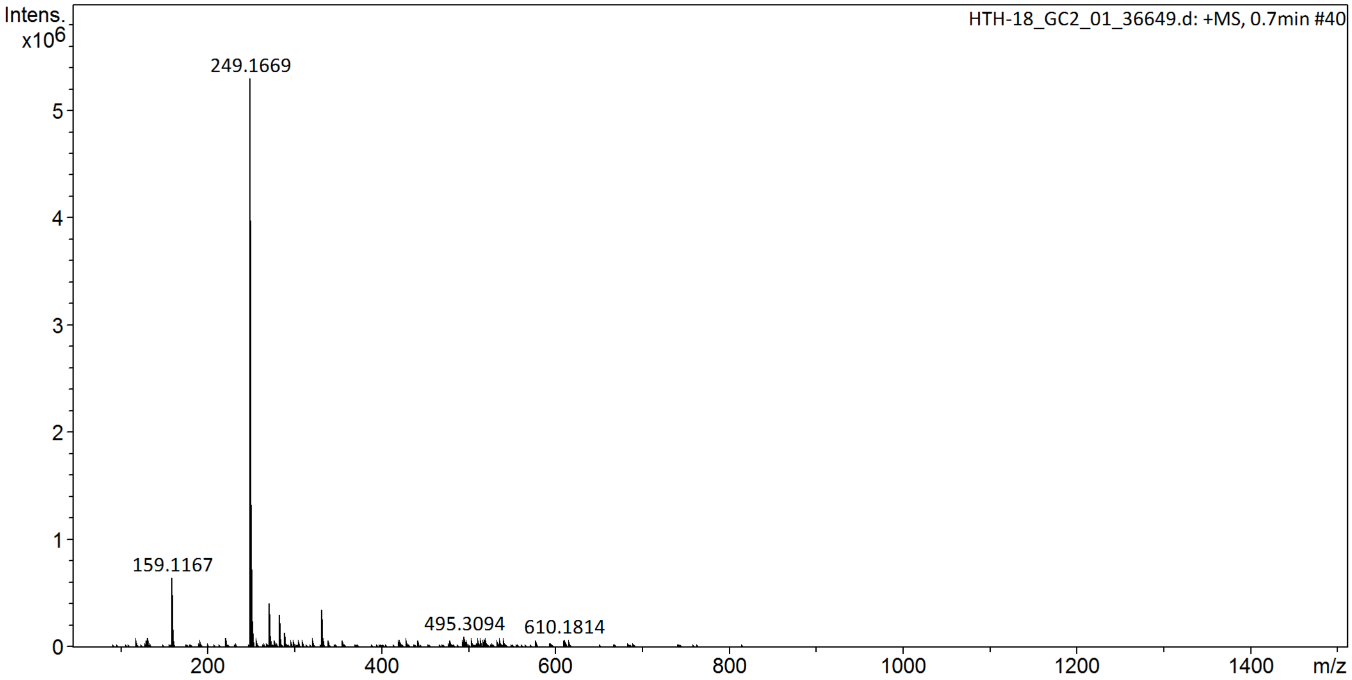

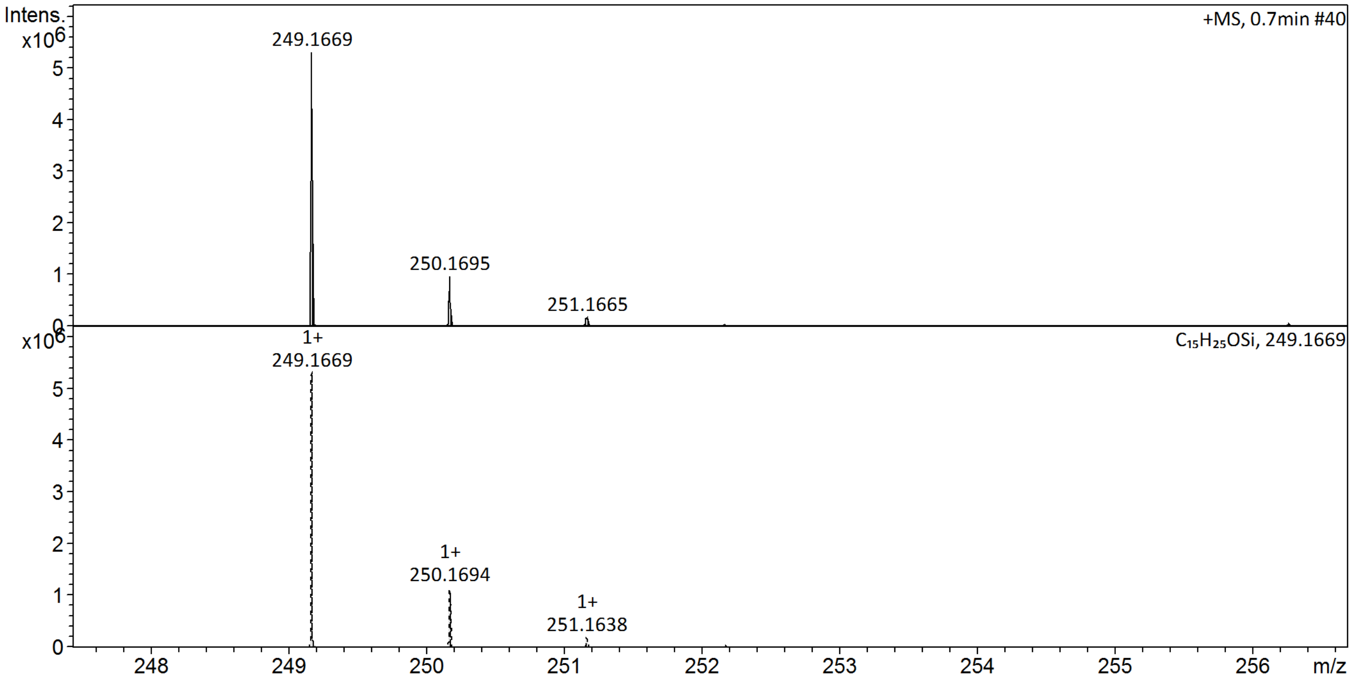

Display Report

Meas. m/z	#	Ion Formula	m/z	err [ppm]	mSigma	# Sigma	Score	rdb	e⁻ Conf	N-Rule	Adduct
249.1669	1	C15H25OSi	249.1669	0.2	20.7	1	100.00	4.5	even	ok	M+H

+MS, 0.7min #40

#	m/z	Res.	S/N	I	I %	FWHM
1	91.0546	13335	29.7	9162	0.2	0.0068
2	93.0697	15192	26.7	8248	0.2	0.0061
3	95.0854	17197	31.5	9740	0.2	0.0055
4	105.0695	17635	63.4	19742	0.4	0.0060
5	109.0647	17655	53.4	16688	0.3	0.0062
6	109.1010	17009	21.5	6712	0.1	0.0064
7	116.1069	17211	22.9	7202	0.1	0.0067
8	117.0698	17552	137.0	43098	0.8	0.0067
9	118.0858	19198	170.1	53520	1.0	0.0062
10	119.0854	16386	65.3	20554	0.4	0.0073
11	123.0801	17723	34.5	10920	0.2	0.0069
12	127.1479	16646	71.4	22666	0.4	0.0076
13	129.0725	17840	205.9	65422	1.2	0.0072
14	130.0748	14332	21.7	6916	0.1	0.0091
15	131.0853	18643	262.5	83502	1.6	0.0070
16	132.0888	17621	30.5	9720	0.2	0.0075
17	133.1012	17289	78.4	24992	0.5	0.0077
18	135.1162	19023	22.8	7278	0.1	0.0071
19	140.0677	18602	25.7	8254	0.2	0.0075
20	145.1003	17993	21.7	6992	0.1	0.0081
21	147.1167	16223	26.0	8354	0.2	0.0091
22	149.0231	18857	51.5	16608	0.3	0.0079
23	149.1319	18061	26.9	8664	0.2	0.0083
24	155.0883	18562	57.2	18510	0.3	0.0084
25	157.1011	19525	34.5	11160	0.2	0.0080
26	159.1167	20935	1984.3	643204	12.2	0.0076
27	160.1197	18900	239.8	77746	1.5	0.0085
28	167.0335	19597	26.2	8522	0.2	0.0085
29	167.0556	18216	21.9	7128	0.1	0.0092
30	169.0862	18629	21.0	6818	0.1	0.0091
31	175.1114	19384	62.0	20146	0.4	0.0090
32	177.1269	18999	59.7	19446	0.4	0.0093
33	180.0813	19333	39.8	12940	0.2	0.0093
34	181.1039	18109	33.2	10814	0.2	0.0100
35	187.1480	18327	23.2	7570	0.1	0.0102
36	189.0865	19389	83.5	27206	0.5	0.0098
37	189.5872	24703	23.7	7710	0.1	0.0077
38	191.1064	17735	21.2	6916	0.1	0.0108
39	191.5890	21857	121.9	39718	0.8	0.0088
40	192.0903	16658	24.9	8112	0.2	0.0115
41	193.1041	20633	38.1	12426	0.2	0.0094
42	200.2005	18672	38.2	12458	0.2	0.0107
43	200.5939	20708	97.9	31894	0.6	0.0097
44	201.0952	18647	23.3	7574	0.1	0.0108
45	205.0853	17647	23.1	7510	0.1	0.0116
46	207.1187	18312	40.6	13182	0.2	0.0113
47	209.1162	18502	24.0	7812	0.1	0.0113
48	213.0881	18565	33.6	10912	0.2	0.0115
49	217.1050	18531	23.5	7638	0.1	0.0117
50	221.1351	20741	269.9	87450	1.7	0.0107
51	222.1381	18279	45.5	14748	0.3	0.0122
52	223.0628	20271	23.2	7506	0.1	0.0110
53	223.0954	17113	23.2	7500	0.1	0.0130
54	224.1275	18832	47.5	15368	0.3	0.0119
55	231.1559	19223	27.8	8988	0.2	0.0120
56	233.1351	21300	65.1	20998	0.4	0.0109
57	239.0926	16803	24.8	7976	0.2	0.0142
58	247.1502	20312	46.6	14906	0.3	0.0122
59	247.2412	20079	37.4	11972	0.2	0.0123

#	m/z	Res.	S/N	I	I %	FWHM
1	249.1669	32009		5292018	100.0	0.0078
2	250.1694	32138		1142610	21.6	0.0078
3	251.1638	32265		179365	3.4	0.0078
4	251.1720	32266		120796	2.3	0.0078
5	252.1671	32394		35258	0.7	0.0078

Display Report

#	m/z	Res.	S/N	I	I %	FWHM
60	249.1380	8445	48.4	15474	0.3	0.0295
61	249.1669	32009	16542.9	5292018	100.0	0.0078
62	250.1695	25402	3025.3	967272	18.3	0.0098
63	251.1665	14820	539.2	172344	3.3	0.0169
64	252.1677	16299	74.8	23902	0.5	0.0155
65	256.2624	21840	148.9	47396	0.9	0.0117
66	257.2483	19517	25.3	8060	0.2	0.0132
67	257.2653	21391	30.7	9762	0.2	0.0120
68	263.1461	20745	47.1	14846	0.3	0.0127
69	263.2367	18211	42.8	13506	0.3	0.0145
70	265.1600	21941	52.2	16418	0.3	0.0121
71	265.2520	21901	88.0	27692	0.5	0.0121
72	267.1765	20978	91.6	28724	0.5	0.0127
73	267.2674	16685	24.6	7712	0.1	0.0160
74	269.1046	16050	31.6	9888	0.2	0.0168
75	269.1336	20650	27.3	8558	0.2	0.0130
76	271.1485	23487	1294.7	404406	7.6	0.0115
77	272.1506	20558	256.1	79926	1.5	0.0132
78	273.1493	13914	51.7	16132	0.3	0.0196
79	274.2737	18621	59.9	18662	0.4	0.0147
80	275.1625	17623	62.3	19384	0.4	0.0156
81	275.2580	20244	25.0	7768	0.1	0.0136
82	277.1979	19919	214.3	66640	1.3	0.0139
83	278.2014	18576	70.6	21936	0.4	0.0150
84	280.2627	21723	99.4	30832	0.6	0.0129
85	282.2788	22908	972.0	301360	5.7	0.0123
86	283.1263	16744	40.7	12598	0.2	0.0169
87	283.2818	20794	187.2	57964	1.1	0.0136
88	284.2911	13986	45.5	14066	0.3	0.0203
89	285.1280	13549	37.8	11670	0.2	0.0210
90	285.2785	21959	49.8	15366	0.3	0.0130
91	287.1437	14116	26.7	8216	0.2	0.0203
92	289.1588	20963	438.3	134802	2.5	0.0138
93	290.1615	20495	95.3	29274	0.6	0.0142
94	290.2681	18312	26.9	8258	0.2	0.0159
95	291.1747	20763	58.9	18098	0.3	0.0140
96	293.1723	17227	66.6	20408	0.4	0.0170
97	295.1546	23368	119.8	36624	0.7	0.0126
98	295.6563	20953	42.1	12876	0.2	0.0141
99	299.0611	21965	109.6	33386	0.6	0.0136
100	299.1763	12207	22.2	6752	0.1	0.0245
101	300.0623	19264	29.0	8820	0.2	0.0156
102	301.1408	19799	36.5	11108	0.2	0.0152
103	302.2456	19851	28.9	8764	0.2	0.0152
104	303.2894	21342	37.9	11476	0.2	0.0142
105	304.1607	22276	27.2	8220	0.2	0.0137
106	304.2603	21981	120.3	36416	0.7	0.0138
107	305.2634	17819	23.8	7212	0.1	0.0171
108	309.1879	17119	113.1	34092	0.6	0.0181
109	310.1907	17573	27.4	8248	0.2	0.0177
110	312.1756	19878	25.4	7658	0.1	0.0157
111	313.1598	17052	28.4	8546	0.2	0.0184
112	318.2995	19426	35.1	10542	0.2	0.0164
113	321.2059	20720	185.7	55554	1.0	0.0155
114	322.2081	19172	50.0	14922	0.3	0.0168
115	323.1985	15637	25.5	7584	0.1	0.0207
116	325.2473	18913	23.1	6880	0.1	0.0172
117	329.1546	23516	33.2	9848	0.2	0.0140
118	329.1914	19792	40.5	12006	0.2	0.0166
119	331.1694	23673	1170.8	347174	6.6	0.0140
120	332.1720	20376	257.7	76354	1.4	0.0163
121	333.1705	15638	71.4	21172	0.4	0.0213
122	338.3407	20751	214.8	63394	1.2	0.0163
123	339.3435	21489	56.6	16682	0.3	0.0158
124	346.2694	24591	36.8	10792	0.2	0.0141
125	347.1433	17697	32.4	9494	0.2	0.0196

Display Report

#	m/z	Res.	S/N	I	I %	FWHM
126	355.0688	21291	212.1	61716	1.2	0.0167
127	355.1689	16976	32.8	9546	0.2	0.0209
128	356.0695	21244	83.2	24184	0.5	0.0168
129	357.0676	19288	51.1	14824	0.3	0.0185
130	360.3237	22426	25.5	7376	0.1	0.0161
131	369.1401	17858	31.2	8968	0.2	0.0207
132	371.1006	20988	49.8	14334	0.3	0.0177
133	373.0797	17062	59.0	16946	0.3	0.0219
134	379.2443	23449	30.1	8634	0.2	0.0162
135	389.2110	19000	52.1	14934	0.3	0.0205
136	392.2180	21087	22.4	6418	0.1	0.0186
137	395.2383	18223	35.6	10200	0.2	0.0217
138	397.2547	20646	63.7	18212	0.3	0.0192
139	399.2027	11384	23.5	6718	0.1	0.0351
140	399.2488	19358	53.1	15194	0.3	0.0206
141	401.2206	15300	48.4	13860	0.3	0.0262
142	401.7251	24664	25.6	7328	0.1	0.0163
143	405.2597	20930	60.3	17274	0.3	0.0194
144	406.2615	21665	23.8	6804	0.1	0.0188
145	407.2741	20312	27.0	7738	0.1	0.0201
146	411.2277	16933	27.8	7988	0.2	0.0243
147	413.2528	14977	32.1	9202	0.2	0.0276
148	419.3138	21921	132.7	38054	0.7	0.0191
149	420.3186	18981	36.4	10414	0.2	0.0221
150	421.2327	15864	130.3	37264	0.7	0.0266
151	422.2366	16557	32.0	9144	0.2	0.0255
152	423.2693	21154	108.7	31070	0.6	0.0200
153	424.2731	21385	41.6	11888	0.2	0.0198
154	425.2131	20783	27.3	7792	0.1	0.0205
155	429.0876	21983	189.7	54106	1.0	0.0195
156	429.2476	12960	26.6	7576	0.1	0.0331
157	430.0878	20335	77.7	22164	0.4	0.0212
158	431.0860	19058	52.8	15066	0.3	0.0226
159	431.2760	18217	24.3	6930	0.1	0.0237
160	437.2098	14175	22.4	6394	0.1	0.0308
161	437.2475	21219	53.1	15138	0.3	0.0206
162	438.2510	25917	22.8	6486	0.1	0.0169
163	439.2631	19216	41.4	11808	0.2	0.0229
164	441.2948	19030	229.8	65478	1.2	0.0232
165	442.2975	19531	68.3	19462	0.4	0.0226
166	445.1188	21482	50.1	14256	0.3	0.0207
167	451.2285	21314	22.7	6460	0.1	0.0212
168	453.2569	14106	49.6	14098	0.3	0.0321
169	455.2608	17820	34.3	9764	0.2	0.0255
170	455.3326	21355	35.7	10156	0.2	0.0213
171	467.1005	20957	31.4	8946	0.2	0.0223
172	469.2376	21292	28.3	8078	0.2	0.0220
173	469.3119	17307	31.5	8992	0.2	0.0271
174	471.3276	20447	66.0	18794	0.4	0.0231
175	477.2989	18771	76.8	21952	0.4	0.0254
176	478.3023	18910	30.7	8782	0.2	0.0253
177	479.3140	21445	233.1	66718	1.3	0.0224
178	480.3167	19780	95.2	27236	0.5	0.0243
179	481.3090	13979	35.2	10076	0.2	0.0344
180	483.2928	16403	42.8	12238	0.2	0.0295
181	485.3060	15184	22.4	6402	0.1	0.0320
182	487.3223	19000	32.8	9370	0.2	0.0256
183	489.3342	29345	31.1	8888	0.2	0.0167
184	493.2962	16741	139.2	39814	0.8	0.0295
185	494.2986	16440	63.2	18078	0.3	0.0301
186	495.3094	22268	330.3	94486	1.8	0.0222
187	496.3111	20168	130.3	37290	0.7	0.0246
188	497.3216	17188	140.8	40318	0.8	0.0289
189	498.3259	16510	50.9	14586	0.3	0.0302
190	499.3269	12782	22.2	6356	0.1	0.0391
191	501.2973	21542	41.7	11954	0.2	0.0233

Display Report

#	m/z	Res.	S/N	I	I %	FWHM
192	503.1059	21937	176.3	50506	1.0	0.0229
193	504.1067	21817	84.4	24162	0.5	0.0231
194	505.1046	20607	59.9	17160	0.3	0.0245
195	506.5278	20804	33.0	9432	0.2	0.0243
196	508.5436	22256	41.9	11998	0.2	0.0228
197	509.2965	13997	74.2	21276	0.4	0.0364
198	510.2964	15105	40.8	11696	0.2	0.0338
199	511.3051	17279	162.7	46684	0.9	0.0296
200	512.3082	18114	77.3	22176	0.4	0.0283
201	513.3181	19764	178.5	51250	1.0	0.0260
202	514.3213	22069	79.2	22784	0.4	0.0233
203	515.3193	14936	28.8	8294	0.2	0.0345
204	516.2961	19428	46.0	13228	0.2	0.0266
205	516.7979	23089	38.4	11038	0.2	0.0224
206	517.2918	20089	126.0	36238	0.7	0.0257
207	518.2952	17788	49.9	14362	0.3	0.0291
208	519.1366	22915	222.8	64082	1.2	0.0227
209	519.3065	21716	195.9	56334	1.1	0.0239
210	520.1380	21361	110.0	31598	0.6	0.0244
211	520.3085	21069	84.0	24144	0.5	0.0247
212	521.1357	19590	82.4	23672	0.4	0.0266
213	521.3107	12636	28.5	8190	0.2	0.0413
214	522.1351	21148	32.6	9350	0.2	0.0247
215	525.2912	14767	47.9	13748	0.3	0.0356
216	527.2992	21478	102.7	29452	0.6	0.0246
217	528.3027	18844	42.6	12216	0.2	0.0280
218	529.3079	11896	29.6	8492	0.2	0.0445
219	531.2802	12548	23.1	6622	0.1	0.0423
220	533.2857	19536	118.4	34002	0.6	0.0273
221	534.2877	20277	49.1	14104	0.3	0.0263
222	535.3008	18326	97.0	27846	0.5	0.0292
223	536.1633	22177	195.3	56054	1.1	0.0242
224	536.3031	19232	41.6	11934	0.2	0.0279
225	536.5747	21043	41.6	11926	0.2	0.0255
226	537.1616	18409	109.1	31308	0.6	0.0292
227	537.3153	15569	36.2	10396	0.2	0.0345
228	537.5782	22368	22.8	6544	0.1	0.0240
229	538.1599	19345	76.4	21916	0.4	0.0278
230	539.1608	17142	34.9	10018	0.2	0.0315
231	539.3332	13310	25.7	7380	0.1	0.0405
232	541.1185	20243	184.9	53132	1.0	0.0267
233	541.2864	13333	22.8	6550	0.1	0.0406
234	542.1195	23031	102.9	29564	0.6	0.0235
235	543.1169	20161	70.3	20222	0.4	0.0269
236	543.2943	17314	40.8	11722	0.2	0.0314
237	544.1179	21954	26.8	7710	0.1	0.0248
238	545.3083	13445	23.4	6740	0.1	0.0406
239	549.2789	19036	60.3	17308	0.3	0.0289
240	550.2836	19075	24.4	7010	0.1	0.0288
241	551.2949	14367	35.7	10228	0.2	0.0384
242	555.3285	18073	54.8	15712	0.3	0.0307
243	556.3340	19267	29.5	8440	0.2	0.0289
244	557.0933	25411	43.4	12420	0.2	0.0219
245	558.0941	22553	22.5	6436	0.1	0.0247
246	561.3164	18985	38.0	10864	0.2	0.0296
247	565.2749	18718	34.0	9732	0.2	0.0302
248	571.3242	17899	31.4	8968	0.2	0.0319
249	577.1238	20872	56.1	15986	0.3	0.0277
250	577.3115	20498	216.1	61590	1.2	0.0282
251	578.1260	21724	31.4	8962	0.2	0.0266
252	578.3145	18838	90.1	25702	0.5	0.0307
253	579.1240	18745	23.2	6604	0.1	0.0309
254	579.3163	12915	55.5	15822	0.3	0.0449
255	581.2810	13639	23.0	6538	0.1	0.0426
256	593.1558	21115	110.2	31320	0.6	0.0281
257	593.3053	19482	105.4	29972	0.6	0.0305

Display Report

#	m/z	Res.	S/N	I	I %	FWHM
258	594.1560	21441	73.3	20842	0.4	0.0277
259	594.3072	17127	41.0	11646	0.2	0.0347
260	595.1548	20919	50.7	14440	0.3	0.0285
261	595.3202	22187	106.1	30190	0.6	0.0268
262	596.3240	18216	40.1	11410	0.2	0.0327
263	609.3000	15734	36.1	10248	0.2	0.0387
264	610.1814	22615	236.1	67080	1.3	0.0270
265	611.1813	20767	135.0	38368	0.7	0.0294
266	611.3099	14907	28.9	8218	0.2	0.0410
267	612.1809	20166	102.2	29034	0.5	0.0304
268	613.1794	18674	39.4	11206	0.2	0.0328
269	615.1367	21040	131.2	37284	0.7	0.0292
270	616.1384	20462	77.2	21934	0.4	0.0301
271	617.1360	20975	57.2	16236	0.3	0.0294
272	618.1357	20886	25.4	7216	0.1	0.0296
273	622.0255	21393	28.3	8036	0.2	0.0291
274	631.1105	19956	23.1	6548	0.1	0.0316
275	651.1436	20714	31.8	8984	0.2	0.0314
276	663.4516	19905	31.2	8776	0.2	0.0333
277	667.1732	19957	70.8	19902	0.4	0.0334
278	668.1747	20269	48.0	13492	0.3	0.0330
279	669.1728	19557	40.2	11296	0.2	0.0342
280	684.1998	20066	87.2	24378	0.5	0.0341
281	685.1984	19905	65.0	18128	0.3	0.0344
282	685.4326	23876	42.2	11774	0.2	0.0287
283	686.1962	17939	45.6	12710	0.2	0.0383
284	687.1957	22257	28.3	7890	0.1	0.0309
285	689.1559	21786	87.0	24236	0.5	0.0316
286	690.1570	21198	60.7	16900	0.3	0.0326
287	691.1561	21719	45.6	12702	0.2	0.0318
288	741.1934	21218	58.0	15916	0.3	0.0349
289	742.1931	21963	44.6	12240	0.2	0.0338
290	743.1917	20274	37.0	10138	0.2	0.0367
291	758.2191	22417	38.2	10414	0.2	0.0338
292	759.2201	20804	34.8	9472	0.2	0.0365
293	760.2158	20797	23.8	6482	0.1	0.0366
294	763.1741	25057	42.7	11622	0.2	0.0305
295	764.1770	20614	27.9	7590	0.1	0.0371
296	765.1733	19951	24.9	6776	0.1	0.0384
297	815.2115	22818	39.9	10720	0.2	0.0357
298	816.2140	20462	31.3	8418	0.2	0.0399
299	817.2125	23339	29.7	7966	0.2	0.0350
300	922.0058	26572	25.6	6604	0.1	0.0347
