# Supplementary material for: Diastereodivergent Access to cis,cis- and cis,trans-Angular Triquinanes: Total Synthesis of (±)-Arnicenone
Source: J Am Chem Soc. 2026 Sep 22;148(39):41724–32. doi: 10.1021/jacs.6c09019 (PMC13651531; doi:10.1021/jacs.6c09019)
Supplement: Supplementary file 6 [file ja6c09019_si_004.zip › FID SI/SI-3b/CHL-II-164P/2/164-13C.pdf]

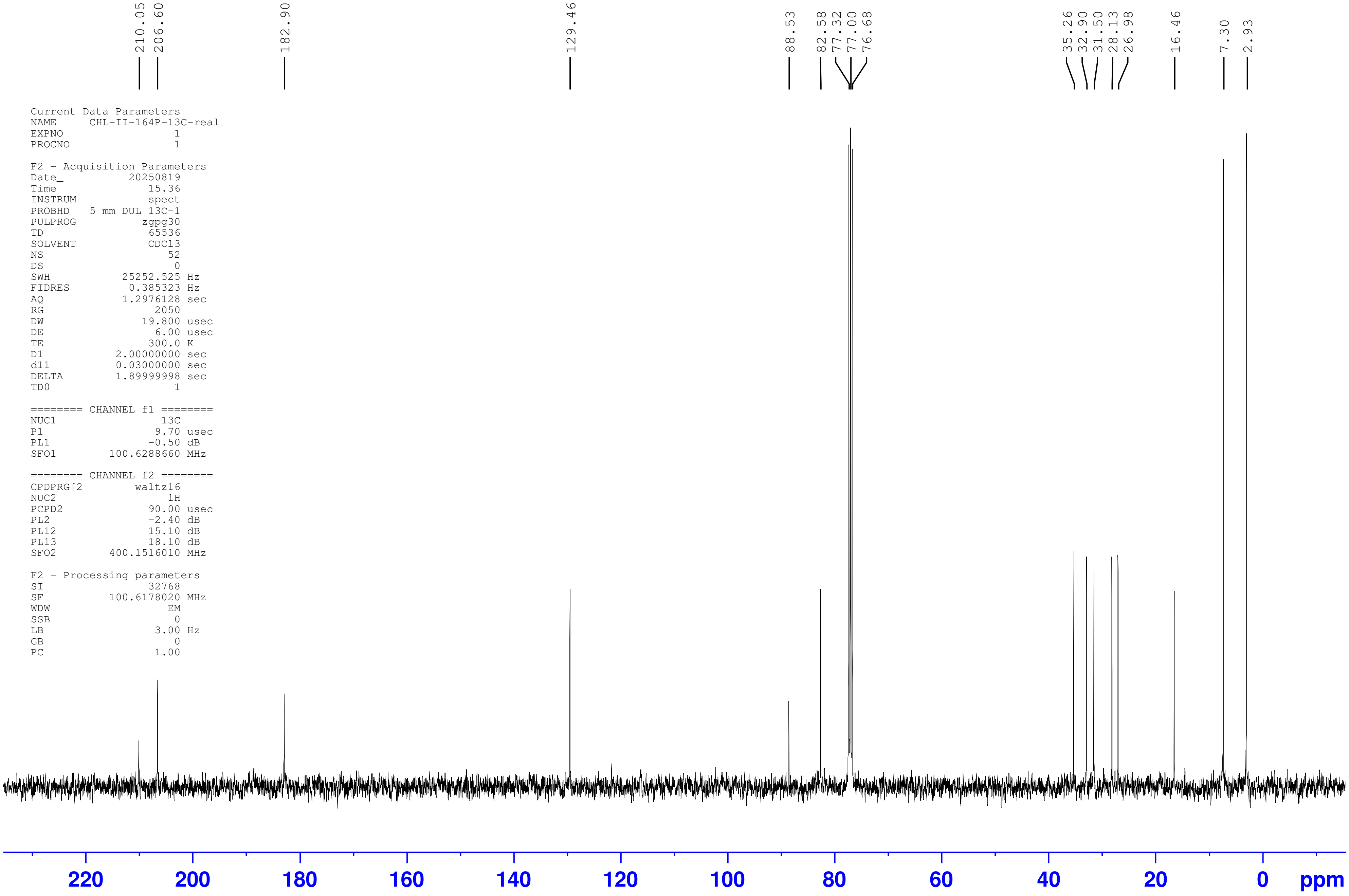

210.05
206.60
182.90
129.46
88.53
82.58
77.32
77.00
76.68
35.26
32.90
31.50
28.13
26.98
16.46
7.30
2.93
Current Data Parameters
NAME CHL-II-164P-13C-real
EXPNO 1
PROCNO 1
F2 - Acquisition Parameters
Date_ 20250819
Time 15.36
INSTRUM spect
PROBHD 5 mm DUL 13C-1
PULPROG zgpg30
TD 65536
SOLVENT CDCl3
NS 52
DS 0
SWH 25252.525 Hz
FIDRES 0.385323 Hz
AQ 1.2976128 sec
RG 2050
DW 19.800 usec
DE 6.00 usec
TE 300.0 K
D1 2.00000000 sec
d11 0.03000000 sec
DELTA 1.89999998 sec
TD0 1
======== CHANNEL f1 ========
NUC1 13C
P1 9.70 usec
PL1 -0.50 dB
SFO1 100.6288660 MHz
======== CHANNEL f2 ========
CPDPRG[2 waltz16
NUC2 1H
PCPD2 90.00 usec
PL2 -2.40 dB
PL12 15.10 dB
PL13 18.10 dB
SFO2 400.1516010 MHz
F2 - Processing parameters
SI 32768
SF 100.6178020 MHz
WDW EM
SSB 0
LB 3.00 Hz
GB 0
PC 1.00
220
200
180
160
140
120
100
80
60
40
20
0
ppm
