# Supplementary material for: Diastereodivergent Access to cis,cis- and cis,trans-Angular Triquinanes: Total Synthesis of (±)-Arnicenone
Source: J Am Chem Soc. 2026 Sep 22;148(39):41724–32. doi: 10.1021/jacs.6c09019 (PMC13651531; doi:10.1021/jacs.6c09019)
Supplement: Supplementary file 7 [file ja6c09019_si_005.zip › FID-arnicenone/HTH-arnicenone-MS.pdf]

Display Report

Analysis Info

Analysis Name	D:\Data\nctu service\data\2023\20230224\HTH-arnicenone _BC7_01_34674.d	Acquisition Date	2/24/2023 4:29:11 PM
Method	Small molecule.m	Operator	NCTU
Sample Name	HTH-arnicenone	Instrument	impact HD 1819696.00164
Comment			

Acquisition Parameter

Source Type	ESI	Ion Polarity	Positive	Set Nebulizer	1.0 Bar
Focus	Active	Set Capillary	4500 V	Set Dry Heater	200 °C
Scan Begin	50 m/z	Set End Plate Offset	-500 V	Set Dry Gas	6.0 l/min
Scan End	1500 m/z	Set Charging Voltage	2000 V	Set Divert Valve	Waste
		Set Corona	0 nA	Set APCI Heater	0 °C

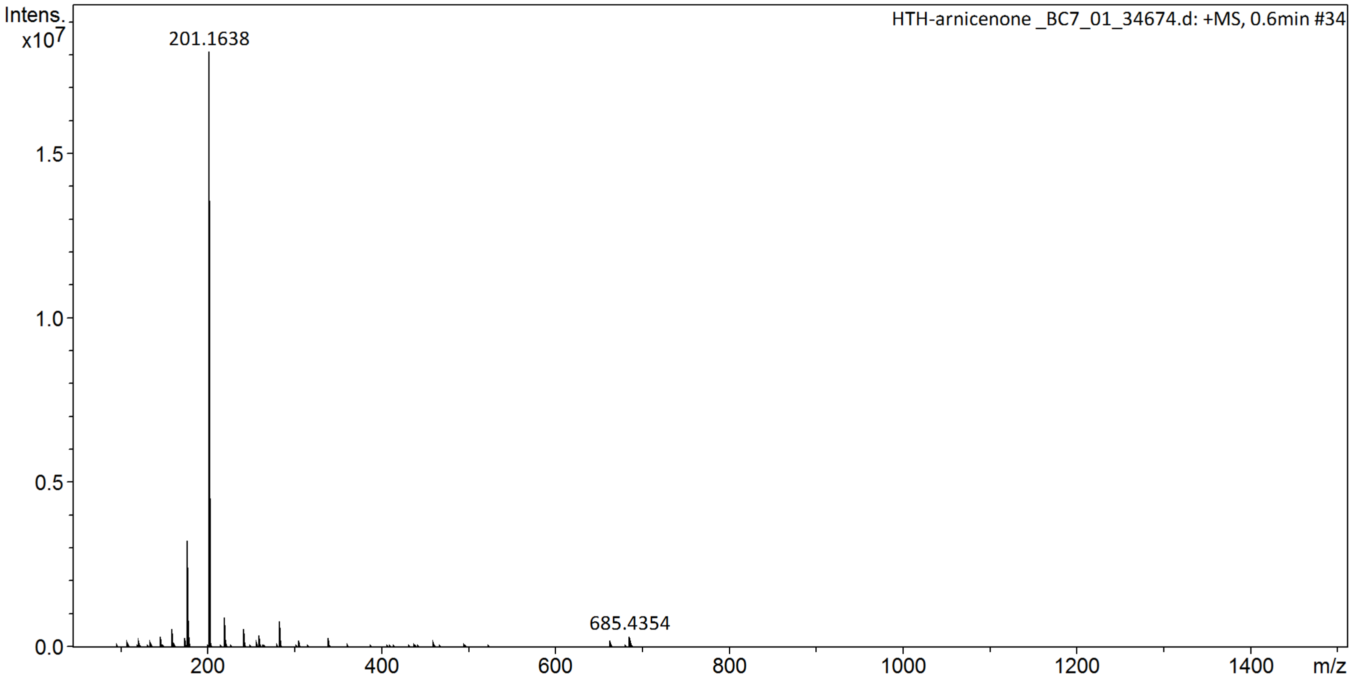

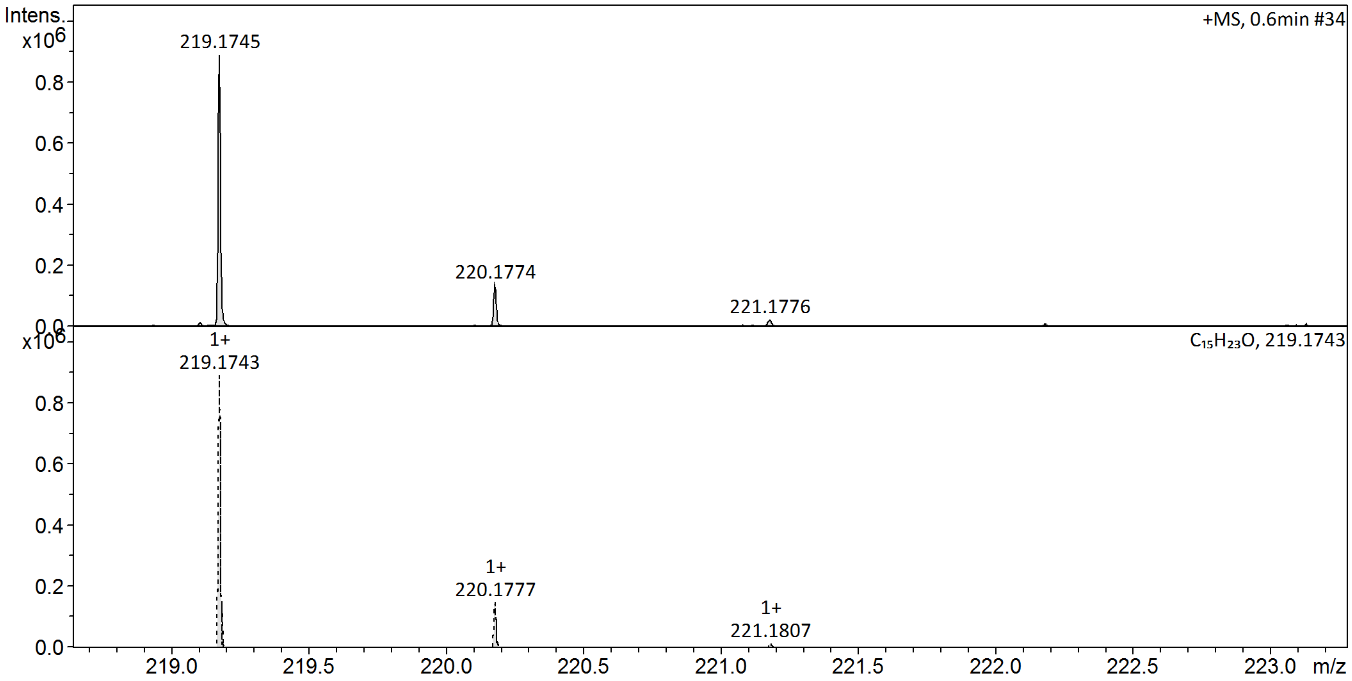

Display Report

Meas. m/z	#	Ion Formula	m/z	err [ppm]	mSigma	# Sigma	Score	rdb	e¯ Conf	N-Rule	Adduct
219.1745	1	C15H23O	219.1743	-0.7	9.0	1	100.00	4.5	even	ok	M+H

+MS, 0.6min #34

#	m/z	Res.	S/N	I	I %	FWHM
1	90.9765	15233	45.3	22118	0.1	0.0060
2	91.0541	16279	41.1	20038	0.1	0.0056
3	93.0697	15978	57.3	27982	0.2	0.0058
4	95.0853	16944	195.4	95352	0.5	0.0056
5	107.0854	17755	289.1	140140	0.8	0.0060
6	109.1010	17013	206.1	99918	0.6	0.0064
7	119.0854	17827	127.8	61774	0.3	0.0067
8	121.1010	17694	328.4	158708	0.9	0.0068
9	122.0980	12316	72.9	35196	0.2	0.0099
10	125.0956	18200	42.1	20308	0.1	0.0069
11	131.0852	18481	70.4	33862	0.2	0.0071
12	133.1010	18660	227.7	109400	0.6	0.0071
13	135.1167	17561	166.9	79998	0.4	0.0077
14	145.1011	19195	638.4	304216	1.7	0.0076
15	146.1048	16811	77.2	36776	0.2	0.0087
16	147.1164	17954	122.0	58034	0.3	0.0082
17	149.0230	19235	48.0	22818	0.1	0.0077
18	149.1322	19039	75.4	35818	0.2	0.0078
19	158.9641	19353	74.6	35446	0.2	0.0082
20	159.1168	21380	1148.0	545014	3.0	0.0074
21	160.1205	17909	148.5	70558	0.4	0.0089
22	161.1320	19010	190.8	90474	0.5	0.0085
23	173.1324	20311	634.7	298382	1.6	0.0085
24	174.1358	18412	84.0	39504	0.2	0.0095
25	177.1639	26178	6902.0	3235724	17.9	0.0068
26	178.1671	21119	869.6	407320	2.3	0.0084
27	179.1710	16611	55.7	26042	0.1	0.0108
28	185.1147	18495	42.9	19874	0.1	0.0100
29	186.1402	18740	42.4	19566	0.1	0.0099
30	194.1152	18638	44.7	20330	0.1	0.0104
31	200.2007	19579	133.9	60024	0.3	0.0102
32	201.1380	11687	74.9	33506	0.2	0.0172
33	201.1638	26051	40424.1	18084514	100.0	0.0077
34	201.6261	22434	172.2	76964	0.4	0.0090
35	202.1674	27755	6231.8	2782462	15.4	0.0073
36	203.1704	21090	378.3	168524	0.9	0.0096
37	215.1792	20687	72.2	31142	0.2	0.0104
38	219.1745	23699	2084.5	889446	4.9	0.0092
39	220.1774	19969	321.4	136642	0.8	0.0110
40	221.1776	14701	53.0	22514	0.1	0.0150
41	226.1798	19010	51.3	21464	0.1	0.0119
42	226.9511	19716	140.5	58712	0.3	0.0115
43	241.1563	23931	1425.8	573324	3.2	0.0101
44	242.1593	21734	229.5	91992	0.5	0.0111
45	247.2417	19599	72.4	28664	0.2	0.0126
46	249.1566	20090	125.3	49390	0.3	0.0124
47	250.1773	17241	62.0	24394	0.1	0.0145
48	256.2629	21332	360.6	139482	0.8	0.0120
49	257.2666	18594	60.6	23392	0.1	0.0138
50	259.1667	22940	955.5	366996	2.0	0.0113
51	260.1696	21097	148.0	56770	0.3	0.0123
52	263.2363	20713	97.3	37058	0.2	0.0127
53	265.0806	21363	79.2	30028	0.2	0.0124
54	265.2521	21210	166.7	63158	0.3	0.0125
55	275.1620	20107	66.3	24080	0.1	0.0137
56	278.2458	19732	60.9	21918	0.1	0.0141
57	280.2630	20906	224.3	80202	0.4	0.0134
58	282.2790	25587	2190.0	781268	4.3	0.0110
59	283.2819	20316	387.8	137942	0.8	0.0139

#	m/z	Res.	S/N	I	I %	FWHM
1	219.1743	23699		889446	100.0	0.0092
2	220.1777	23807		146653	16.5	0.0092
3	221.1807	23916		13134	1.5	0.0092

Display Report

#	m/z	Res.	S/N	I	I %	FWHM
60	284.2908	14877	87.6	31040	0.2	0.0191
61	301.1406	20804	111.7	36782	0.2	0.0145
62	302.2445	21557	197.4	64858	0.4	0.0140
63	304.2605	22060	610.2	199462	1.1	0.0138
64	305.2636	20406	120.7	39442	0.2	0.0150
65	315.2493	20992	95.5	30588	0.2	0.0150
66	338.3413	22687	893.9	280666	1.6	0.0149
67	339.3442	20989	207.3	65064	0.4	0.0162
68	357.2391	22002	82.3	25660	0.1	0.0162
69	360.3225	21331	242.6	75542	0.4	0.0169
70	362.9256	21332	58.8	18272	0.1	0.0170
71	387.2133	21480	136.0	42442	0.2	0.0180
72	406.3145	17152	112.2	35360	0.2	0.0237
73	408.3082	18304	62.0	19598	0.1	0.0223
74	409.1950	21085	161.7	51088	0.3	0.0194
75	413.2651	22155	198.2	62652	0.3	0.0187
76	419.3133	21590	60.9	19394	0.1	0.0194
77	431.1775	19844	96.8	31190	0.2	0.0217
78	437.1922	22080	66.2	21448	0.1	0.0198
79	437.3406	22050	293.2	94948	0.5	0.0198
80	438.3428	20603	97.1	31400	0.2	0.0213
81	441.2964	21596	156.8	50858	0.3	0.0204
82	447.3431	19288	60.9	19930	0.1	0.0232
83	459.3223	21904	326.8	108020	0.6	0.0210
84	460.3253	20340	106.1	35114	0.2	0.0226
85	466.5336	22515	110.6	36762	0.2	0.0207
86	475.3245	20085	66.5	22342	0.1	0.0237
87	494.5646	22560	276.9	94710	0.5	0.0219
88	495.1246	20942	55.5	18986	0.1	0.0236
89	495.4870	21451	75.1	25702	0.1	0.0231
90	495.5677	22665	109.5	37496	0.2	0.0219
91	522.5960	21185	193.5	67048	0.4	0.0247
92	523.6002	23382	76.8	26640	0.1	0.0224
93	536.1642	21815	79.3	27454	0.2	0.0246
94	553.4590	21497	59.4	20482	0.1	0.0257
95	663.4534	23792	619.5	189044	1.0	0.0279
96	664.4565	21652	275.9	84026	0.5	0.0307
97	665.4598	20412	76.2	23164	0.1	0.0326
98	680.4798	19977	128.7	37996	0.2	0.0341
99	681.4820	20216	67.7	19964	0.1	0.0337
100	685.4354	24498	1147.1	335992	1.9	0.0280
101	686.4388	22560	521.7	152572	0.8	0.0304
102	687.4411	21590	129.0	37656	0.2	0.0318
103	701.4086	19529	79.3	22594	0.1	0.0359

HTH-arnicenone _BC7_01_34674.d
