# Supplementary material for: Diastereodivergent Access to cis,cis- and cis,trans-Angular Triquinanes: Total Synthesis of (±)-Arnicenone
Source: J Am Chem Soc. 2026 Sep 22;148(39):41724–32. doi: 10.1021/jacs.6c09019 (PMC13651531; doi:10.1021/jacs.6c09019)
Supplement: Supplementary file 7 [file ja6c09019_si_005.zip › FID-arnicenone/HTH-arnicenone-COSY.pdf]

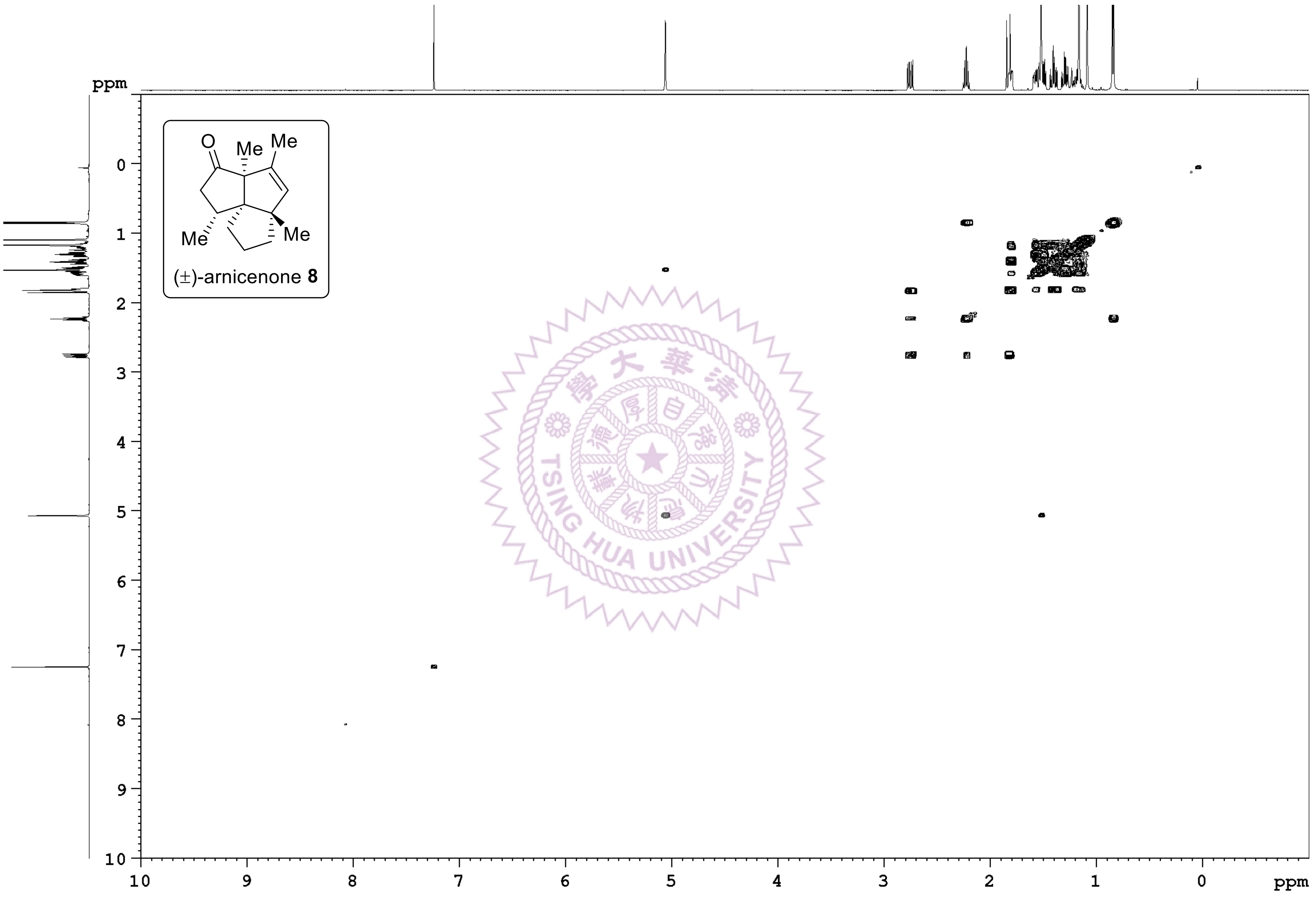

TSING HUA UNIVERSITY
