# Supplementary material for: Diastereodivergent Access to cis,cis- and cis,trans-Angular Triquinanes: Total Synthesis of (±)-Arnicenone
Source: J Am Chem Soc. 2026 Sep 22;148(39):41724–32. doi: 10.1021/jacs.6c09019 (PMC13651531; doi:10.1021/jacs.6c09019)
Supplement: Supplementary file 7 [file ja6c09019_si_005.zip › FID-arnicenone/HTH-arnicenone-13C.pdf]

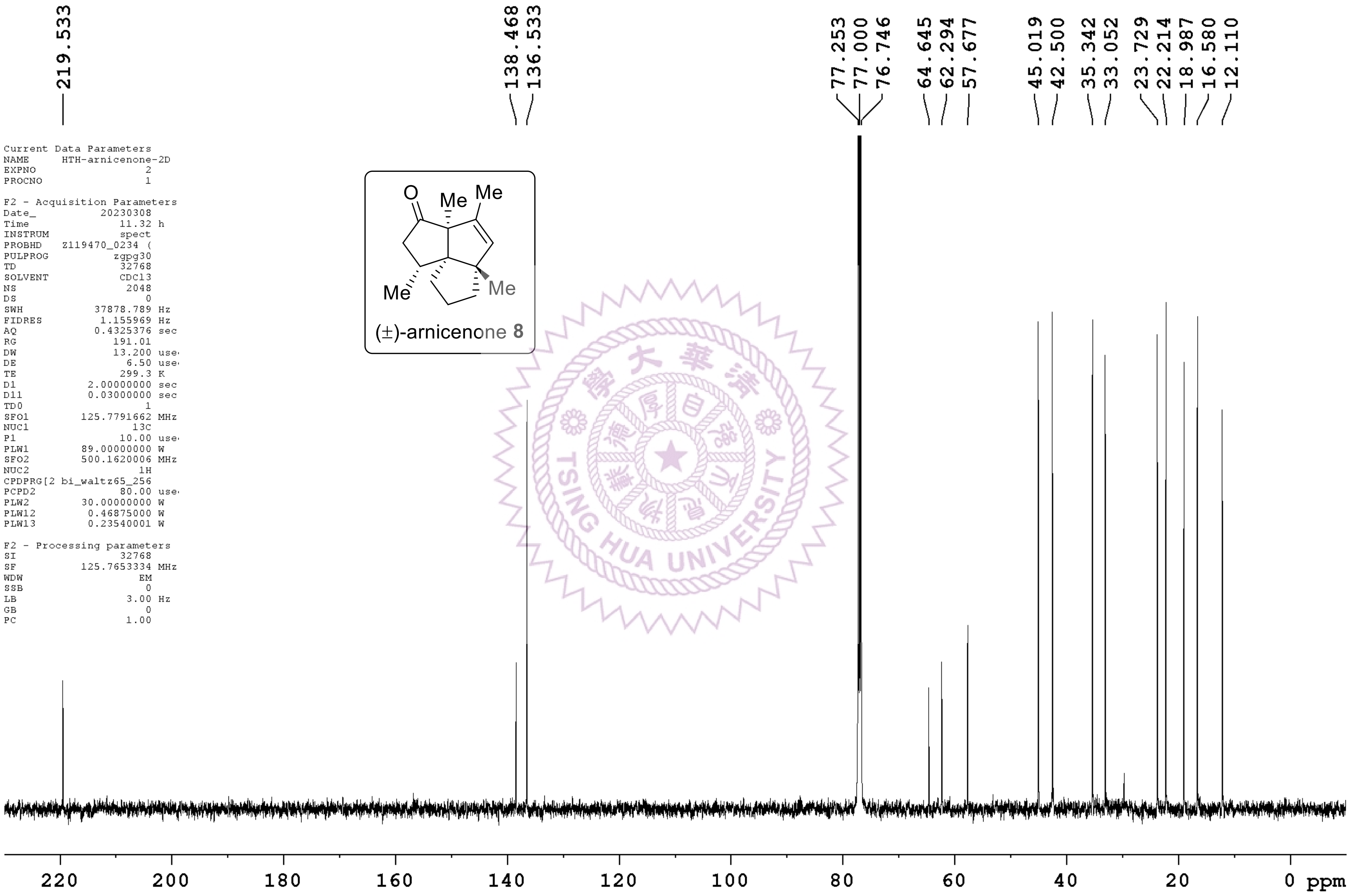
219.533
138.468
136.533
77.253
77.000
76.746
64.645
62.294
57.677
45.019
42.500
35.342
33.052
23.729
22.214
18.987
16.580
12.110
Current Data Parameters
NAME HTH-arnicenone-2D
EXPNO 2
PROCNO 1
F2 - Acquisition Parameters
Date_ 20230308
Time 11.32 h
INSTRUM spect
PROBHD Z119470_0234 (
PULPROG zgpg30
TD 32768
SOLVENT CDCl3
NS 2048
DS 0
SWH 37878.789 Hz
FIDRES 1.155969 Hz
AQ 0.4325376 sec
RG 191.01
DW 13.200 use
DE 6.50 use
TE 299.3 K
D1 2.00000000 sec
D11 0.03000000 sec
TD0 1
SFO1 125.7791662 MHz
NUC1 13C
P1 10.00 use
PLW1 89.00000000 W
SFO2 500.1620006 MHz
NUC2 1H
CPDPRG[2 bi_waltz65_256
PCPD2 80.00 use
PLW2 30.00000000 W
PLW12 0.46875000 W
PLW13 0.23540001 W
F2 - Processing parameters
SI 32768
SF 125.7653334 MHz
WDW EM
SSB 0
LB 3.00 Hz
GB 0
PC 1.00
O
Me
Me
Me
Me
(±)-arnicenone 8
220
200
180
160
140
120
100
80
60
40
20
0 ppm
