# Supplementary material for: Diastereodivergent Access to cis,cis- and cis,trans-Angular Triquinanes: Total Synthesis of (±)-Arnicenone
Source: J Am Chem Soc. 2026 Sep 22;148(39):41724–32. doi: 10.1021/jacs.6c09019 (PMC13651531; doi:10.1021/jacs.6c09019)
Supplement: Supplementary file 7 [file ja6c09019_si_005.zip › FID-arnicenone/HTH-arnicenone-1H.pdf]

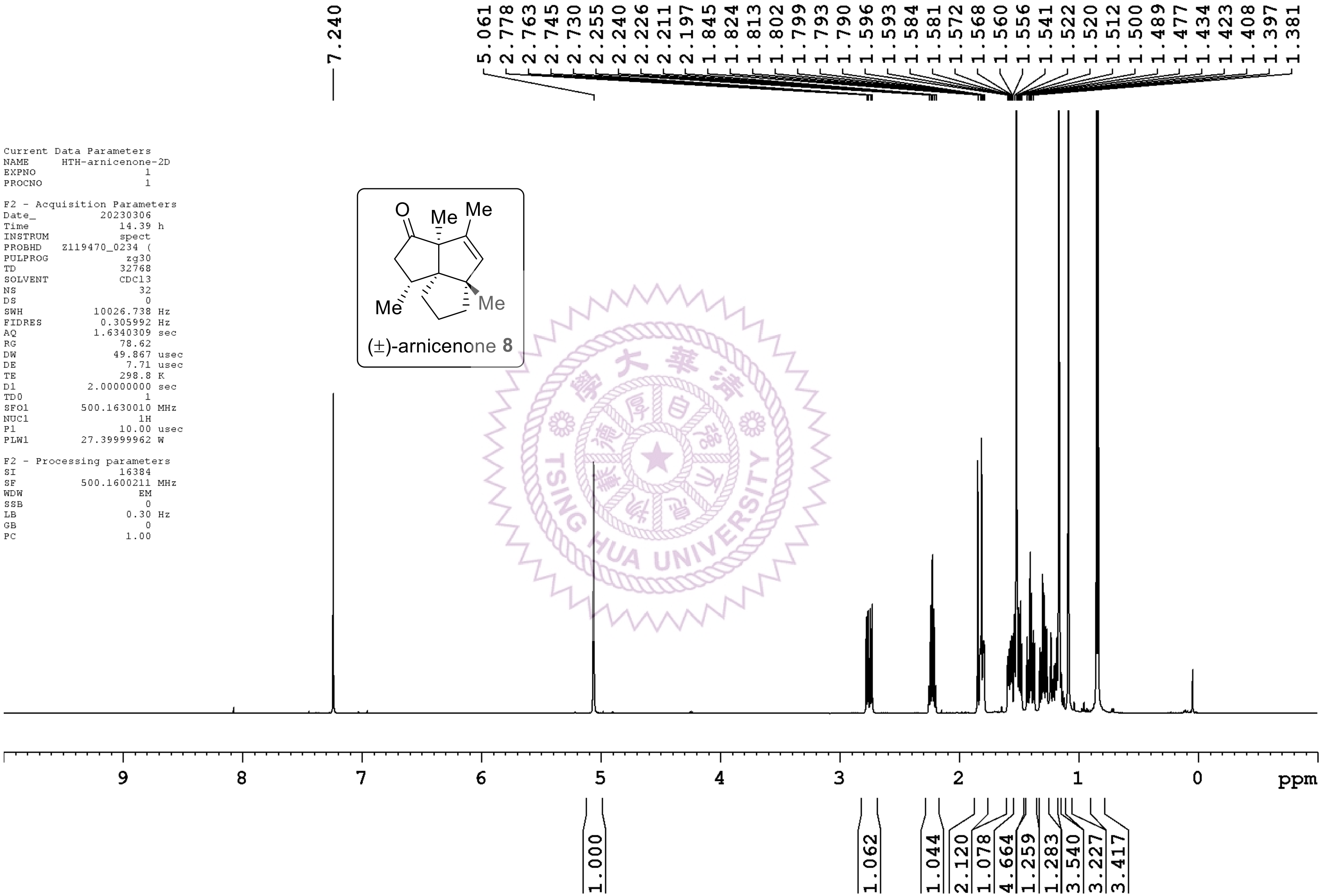
Current Data Parameters
NAME HTH-arnicenone-2D
EXPNO 1
PROCNO 1
F2 - Acquisition Parameters
Date_ 20230306
Time 14.39 h
INSTRUM spect
PROBHD Z119470_0234 (
PULPROG zg30
TD 32768
SOLVENT CDCl3
NS 32
DS 0
SWH 10026.738 Hz
FIDRES 0.305992 Hz
AQ 1.6340309 sec
RG 78.62
DW 49.867 usec
DE 7.71 usec
TE 298.8 K
D1 2.00000000 sec
TD0 1
SFO1 500.1630010 MHz
NUC1 1H
P1 10.00 usec
PLW1 27.39999962 W
F2 - Processing parameters
SI 16384
SF 500.1600211 MHz
WDW EM
SSB 0
LB 0.30 Hz
GB 0
PC 1.00
(±)-arnicenone 8
O
Me
Me
Me
Me
7.240
5.061 2.778 2.763 2.745 2.730 2.255 2.240 2.226 2.211 2.197 1.845 1.824 1.813 1.802 1.799 1.793 1.790 1.596 1.593 1.584 1.581 1.572 1.568 1.560 1.556 1.541 1.522 1.520 1.512 1.500 1.489 1.477 1.434 1.423 1.408 1.397 1.381
9 8 7 6 5 4 3 2 1 0 ppm
1.000 1.062 1.044 2.120 1.078 4.664 1.259 1.283 3.540 3.227 3.417
